# Supplementary material for: Targeting cIAP2 in a novel senolytic strategy prevents glioblastoma recurrence after radiotherapy
Source: EMBO Mol Med. 2025 Feb 19;17(4):645–78. doi: 10.1038/s44321-025-00201-x (PMC11982261; doi:10.1038/s44321-025-00201-x)
Supplement: Supplementary file 1 — Appendix [file 44321_2025_201_MOESM1_ESM.pdf]

Appendix

Targeting cIAP2 in a novel senolytic strategy prevents glioblastoma recurrence after radiotherapy

Tomimatsu et al.

Contents:

**Appendix Figure S1**	Cell death after birinapant treatment or cIAP2 knockdown, related to Figure 4	Page 2
**Appendix Figure S2**	BrdU incorporation in conditioned media-treated PDX cultures, related to Figure EV5	Page 4
**Appendix Figure S3**	Low cIAP1 induction in senescent PDX cultures, related to Figure 6	Page 5
**Appendix Figure S4**	Birinapant resistance of normal human astrocytes, related to Figures 6 and EV5	Page 6
**Appendix Figure S5**	Supporting data for mouse tumor studies, related to Figure 7	Page 7
**Appendix Table S1**	Cytokine levels in conditioned media	Page 9
**Appendix Table S2**	Clinical and molecular information of GBM Patient-Derived Xenografts	Page 10
**Appendix Table S3**	Antibody information	Page 11
**Appendix Table S4**	Exact p values related to Figures 1-d, 5-c and EV2-b	Page 12

Appendix Figure S1: Cell death after birinapant treatment or cIAP2 knockdown, related to Figure 4.

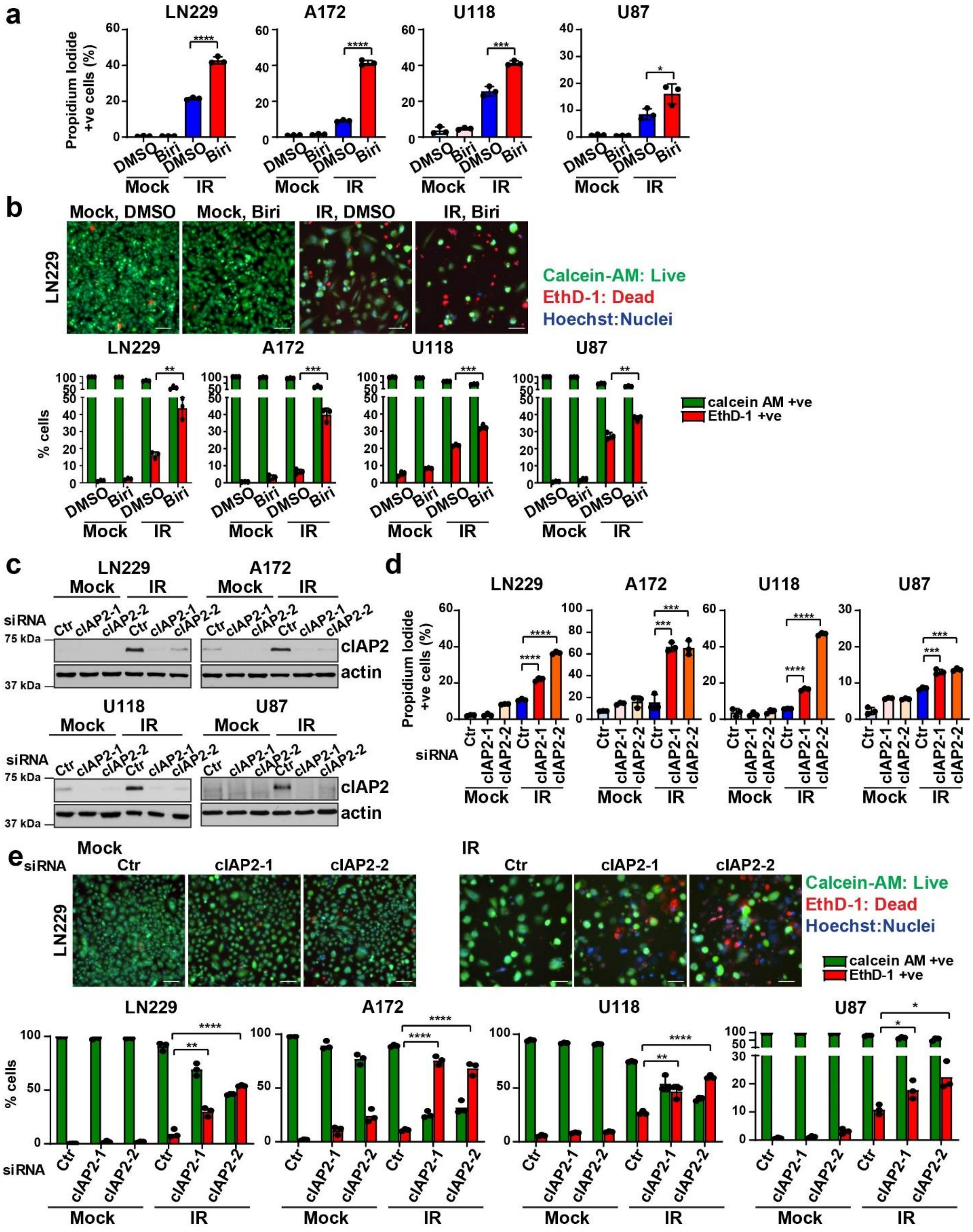

(a) Mock-irradiated (Mock) or irradiated (IR) GBM cells were treated with birinapant (Biri) and analyzed 72 hours post-treatment. Live and dead cells were quantified by flow cytometry based on propidium iodide exclusion (live cells) versus uptake (dead cells). The plots show the percentages of propidium iodide-positive (dead) cells for each treatment condition. **(b)** Mock-irradiated or irradiated GBM cells were treated with birinapant and analyzed 72 hours post-treatment by fluorescence microscopy. Live and dead cells were identified by staining with Calcein-AM (green; live cells) and Ethidium Homodimer-1 (red; dead cells), along with nuclear counterstaining with Hoechst 33342. Images show representative LIVE/DEAD^{TM} staining of LN229 cells. The plots show the percentages of Calcein-AM-positive (live) and Ethidium Homodimer-1-positive (dead) cells for the different treatment conditions. **(c)** Whole cell extracts from mock irradiated or irradiated GBM cell lines 72h after transfection with siRNAs targeting cIAP2 or control siRNA (Ctr) were Western blotted with anti-cIAP2 antibody. Actin serves as loading control. **(d)** The plots show the percentages of propidium iodide-positive (dead) cells 9 days after transfection with the indicated siRNAs. **(e)** Images show representative LIVE/DEAD^{TM} staining of LN229 cells. The plots show the percentages of Calcein-AM-positive (live) and Ethidium Homodimer-1-positive (dead) cells 9 days after transfection with the indicated siRNAs. Scale bar, 100 μm; Error bars, SD; *, $p < 0.05$; **, $p < 0.01$; ***, $p < 0.001$; ****, $p < 0.0001$.

Appendix Figure S2: BrdU incorporation in conditioned media-treated PDX cultures, related to Figure EV5.

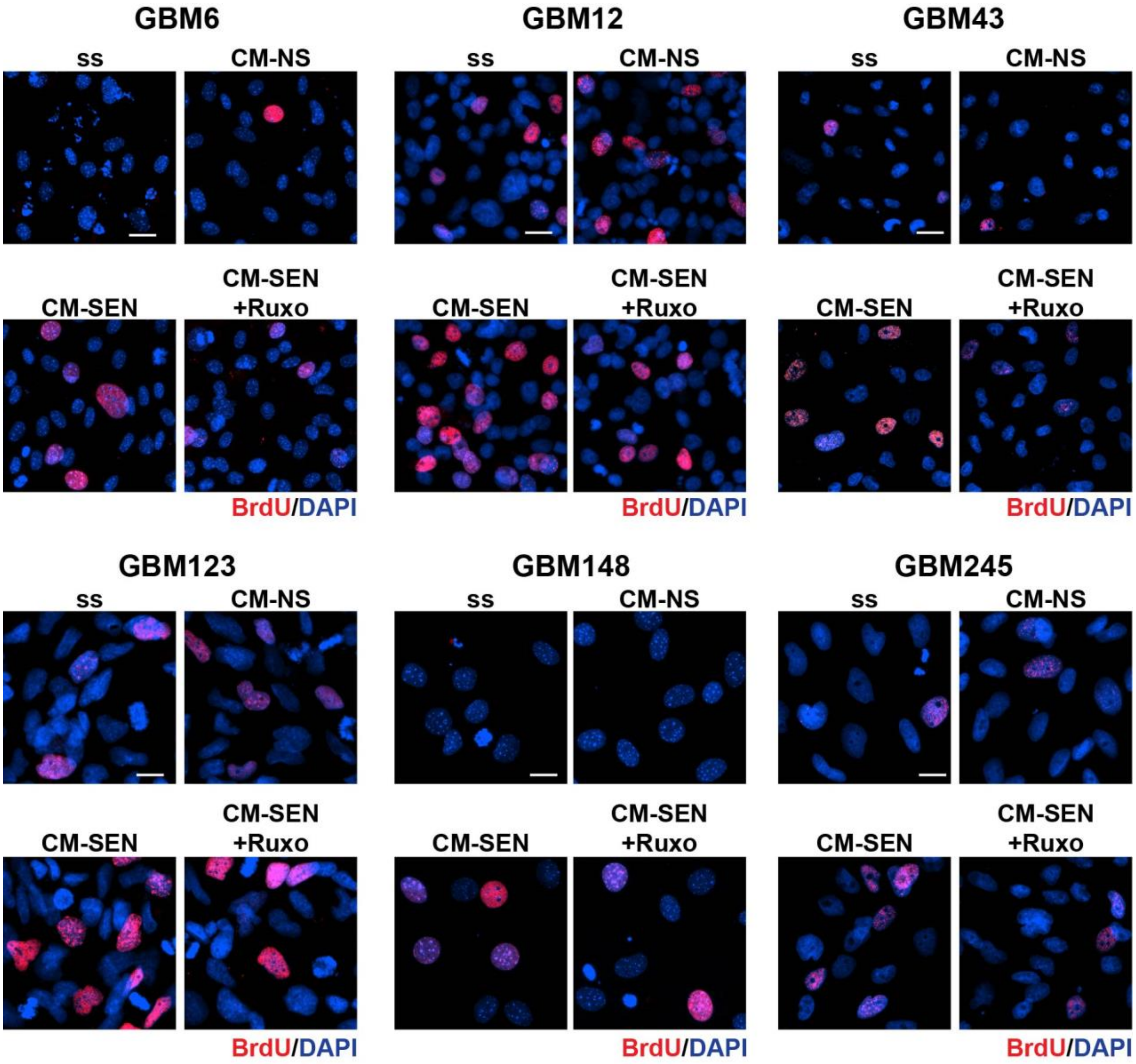

Serum starved (ss) GBM PDX cultures were pulsed with BrdU after exposure to conditioned media from senescent (CM-SEN) or non-senescent (CM-NS) cultures in the absence or presence of ruxolitinib (CM-SEN+Ruxo), and immunofluorescence stained with anti-BrdU antibody (red), as indicated. Nuclei are stained with DAPI (blue). Scale bar, 50 μm.

Appendix Figure S3: Low cIAP1 induction in senescent PDX cultures, related to Figure 6.

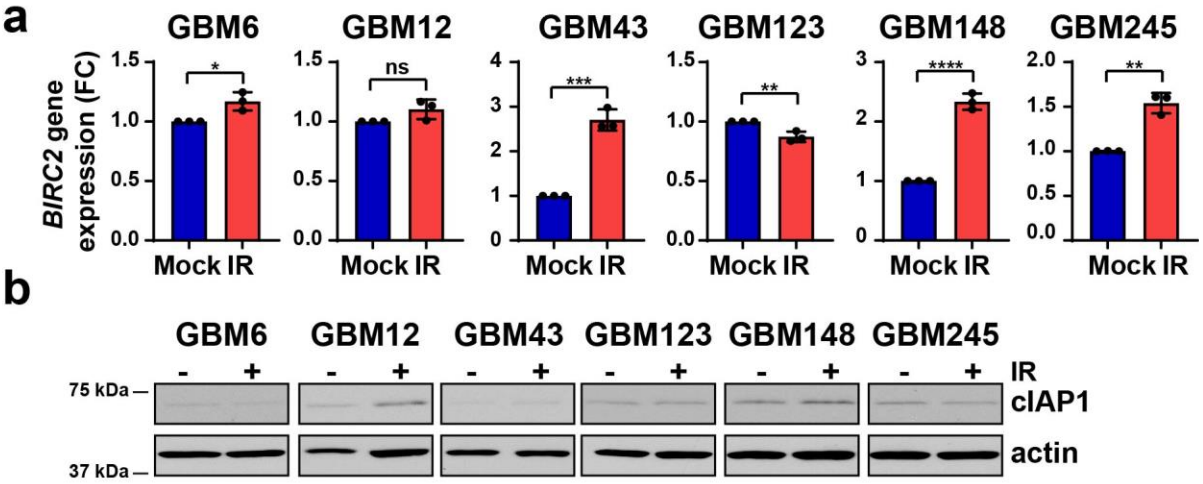

(a) Plots show relative expression of *BIRC2* in mock-irradiated (Mock) *vs.* irradiated (IR) PDX cells 10 days after exposure to 10 Gy of X-rays, as assessed by qRT-PCR. **(b)** Whole cell extracts from mock-irradiated or irradiated GBM PDX cultures were Western blotted with anti-cIAP1 antibody. Actin serves as loading control. n=3; Error bars, SD; *, $p < 0.05$; **, $p < 0.01$; ***, $p < 0.001$; ****, $p < 0.0001$; ns, not significant.

Appendix Figure S4: Birinapant resistance of normal human astrocytes, related to Figures 6 and EV5.

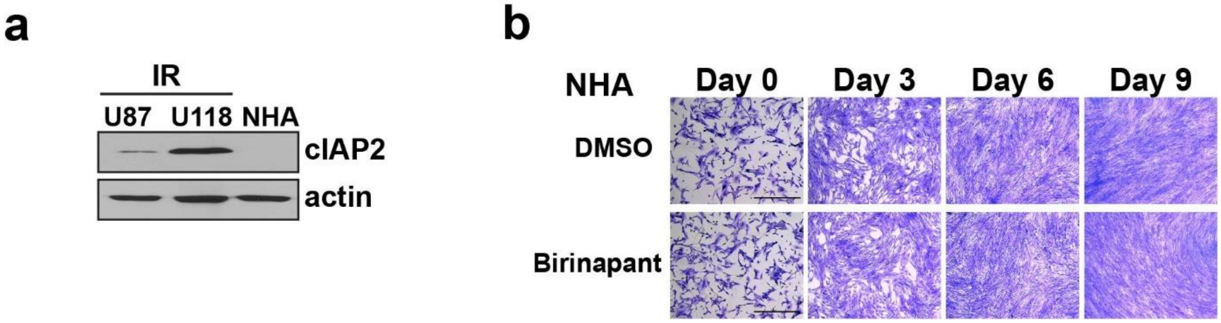

(a) Undetectable levels of cIAP2 in extracts from normal human astrocytes (NHA) as assessed by Western blotting. Extracts from irradiated (IR) U87 and U118 were run alongside for comparison. Actin serves as loading control. **(b)** NHAs were treated with birinapant or DMSO as control, and the surviving cells were visualized by staining with crystal violet at the indicated times. Scale bar, 500 μm.

Appendix Figure S5: Supporting data for mouse tumor studies, related to Figure 7.

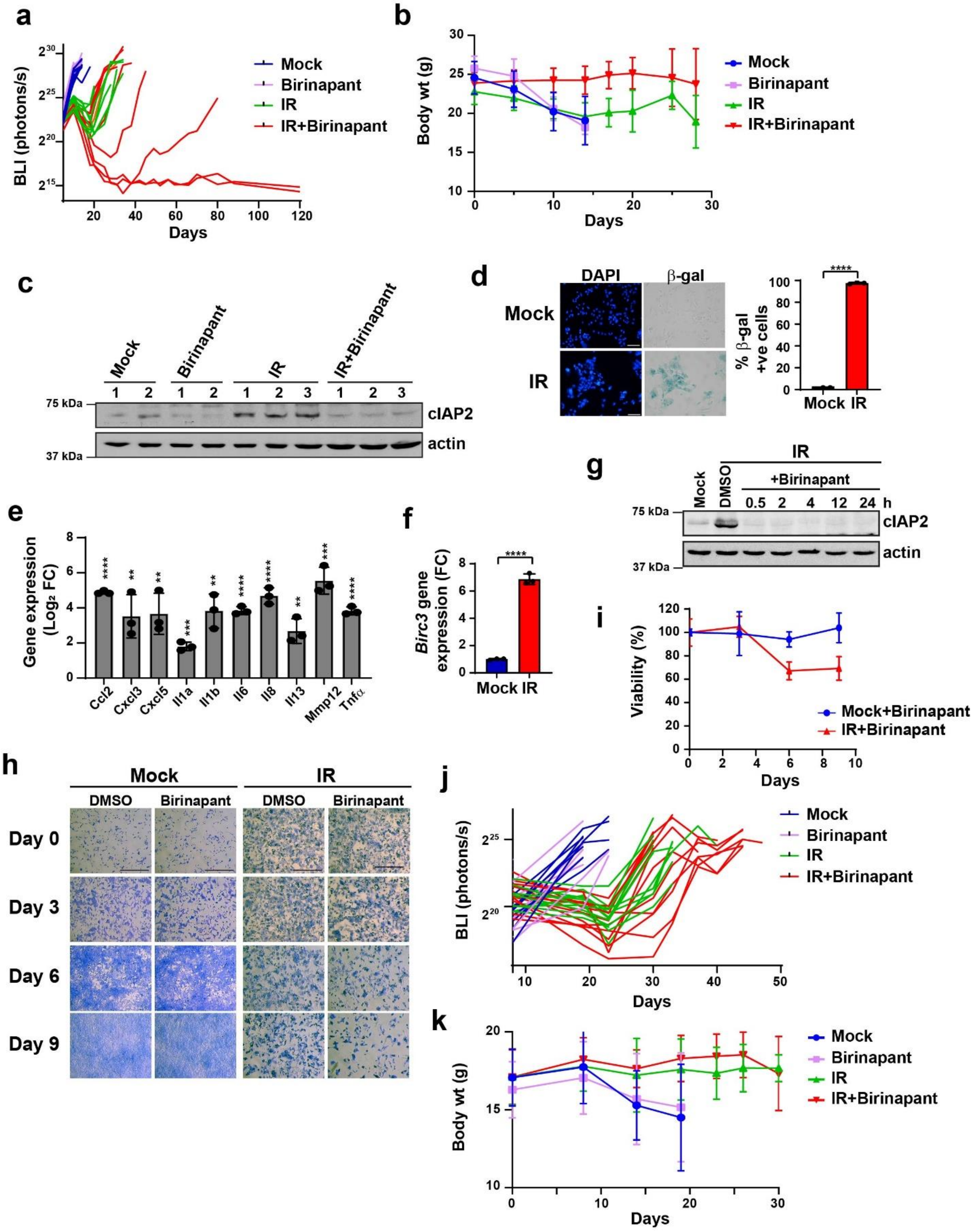

(a) GBM12 cells expressing firefly luciferase were injected intracranially in nude mice to generate orthotopic brain tumors that were monitored by BLI (9 mice per treatment group). Mice with established tumors were randomized into the following treatment groups: i) mock treatment (Mock), ii) birinapant alone (Birinapant), iii) ionizing radiation alone (IR), and iv) IR with adjuvant birinapant (IR+Birinapat). Plot shows quantification of BLI measurements plotted on a Log_2 scale. Each line represents BLI intensity (photons per second) of an individual brain tumor over time. **(b)** Plot shows average body weight of mice for the four treatment arms from the day of inoculation. **(c)** Mice with GBM12 tumors were cranially irradiated with X-rays and treated 2 days later with 5 cycles of birinapant or vehicle, and sacrificed 24 hours after the last treatment. Tumors were excised and cIAP2 levels assessed by western blotting (numbers denote replicate mice). Actin serves as loading control. **(d)** Representative images of SA-β-gal staining of GL261 cells mock-irradiated (Mock) or irradiated (IR) with 10 Gy of X-rays and then allowed to recover for 10 days. Scale bar, 50 μm. **(e)** Total RNA was isolated from GL261 cells 10 days after irradiation with 10 Gy of X-rays or from mock-irradiated cells and expression of SASP genes assessed by qRT-PCR. Plots show fold change (Log_2 FC) in gene expression of SASP-related genes in irradiated GL261 cells relative to mock-irradiated cells. **(f)** Plot shows relative expression of *BIRC3* in mock-irradiated (Mock) *vs.* irradiated (IR) GL261 cells 10 days after exposure to 10 Gy of X-rays, as assessed by qRT-PCR. **(g)** GL261 cells were irradiated with 10 Gy of X-rays (IR) and treated with the cIAP2 inhibitor birinapant (or DMSO as control) after 10 days for the indicated times, and cIAP2 levels were assessed by Western blotting. Actin serves as loading control. **(h)** Mock-irradiated or irradiated GL261 cells were treated with birinapant or DMSO as control, and the surviving cells were visualized by staining with crystal violet at the indicated times (Scale bar, 500 μm), and **(i)** viability was quantified by the MTT assay (values are normalized to that of DMSO-treated cells). The drug was replaced every 72 hours. **(j)** GL261 cells expressing firefly luciferase were injected intracranially in C57BL/6J mice to generate orthotopic brain tumors that were monitored by BLI (12 mice per treatment group). Mice with established tumors were randomized into the indicated treatment groups. Plot shows quantification of BLI measurements plotted on a Log_2 scale. Each line represents BLI intensity (photons per second) of an individual brain tumor over time. **(k)** Plot shows average body weight of mice for the four treatment arms from the day of inoculation. Error bars, SD; **, $p < 0.01$; ***, $p < 0.001$; ****, $p < 0.0001$.

Appendix Table S1: Cytokine levels in conditioned media

	A172			LN229			U87			U118		
	mock (pg/10^6 cells)	IR(pg/10^6 cells)	p_value	mock (pg/10^6 cells)	IR(pg/10^6 cells)	p_value	mock (pg/10^6 cells)	IR(pg/10^6 cells)	p_value	mock (pg/10^6 cells)	IR(pg/10^6 cells)	p_value
**IP-10/CXCL10**	0.27 ± 0.07	110.56 ± 27.8	0.00235	0.34 ± 0.26	15.53 ± 4.62	0.00473	0.33 ± 0.3	4.33 ± 0.42	0.000181666	0.28 ± 0.02	3.13 ± 0.67	0.001781088
**ENA-78/CXCL5**	1.3 ± 1.78	29.49 ± 5.78	0.00128	1.23 ± 2.14	77.83 ± 2.67	0.00000	0 ± 0	123.8 ± 12.48	6.72876E-05	0 ± 0	63.18 ± 4.85	2.28986E-05
**G-CSF**	0 ± 0	18.87 ± 6.18	0.00614	0 ± 0	22.95 ± 4.58	0.00097	17.34 ± 4.58	11519.22 ± 1361.25	0.000126822	0 ± 0	45.36 ± 7.68	0.00051512
**GM-CSF**	0.99 ± 0.25	995.39 ± 257.99	0.00262	0.9 ± 0.23	11.17 ± 1.14	0.00011	0.79 ± 0.2	320.44 ± 46.2	0.000277991	0.7 ± 0.12	80.63 ± 6.6	3.05156E-05
**IFN-γ**	0.04 ± 0.07	6.5 ± 1.49	0.00166	0.12 ± 0.2	1.57 ± 0.15	0.00056	0 ± 0	10.12 ± 1.2	0.000127706	0 ± 0	1.81 ± 0.43	0.001811827
**IL-8/CXCL8**	0.43 ± 0.12	801.99 ± 250.54	0.00519	6.63 ± 2.89	6291.07 ± 132.93	0.00000	1.43 ± 0.76	8219.54 ± 1289.16	0.000382526	5.28 ± 3.56	5965.84 ± 753.82	0.00016465
**MCP-1/CCL2**	450.79 ± 86.15	4362.2 ± 1053.38	0.00304	574.34 ± 165.6	4040.53 ± 102.53	0.00001	10.97 ± 2.92	464.87 ± 46.82	7.42631E-05	5.18 ± 1.22	1754.75 ± 229.1	0.000188759
**RANTES/CCL5**	0 ± 0	263.34 ± 51.92	0.00093	0 ± 0	594.4 ± 51.98	0.00004	0 ± 0	51.53 ± 3.75	1.85243E-05	0 ± 0	12.55 ± 0.39	6.30676E-07
**IL-6 HS**	0.24 ± 0.05	60.98 ± 18.15	0.00441	0.15 ± 0.04	169.12 ± 24.82	0.00030	3.91 ± 1.06	5677.05 ± 778.71	0.000227067	0.27 ± 0.06	196.88 ± 27.93	0.000259863
**MMP-9**	19.03 ± 4.56	243.2 ± 60.75	0.00311	17.13 ± 5.24	28.81 ± 3.48	0.03236	8.94 ± 4.04	60.48 ± 14.65	0.004190431	15.05 ± 0.82	133.74 ± 22.03	0.000736622
**MMP-3**	14.09 ± 2.65	72.45 ± 20.91	0.00868	3.74 ± 2.11	32.93 ± 6.64	0.00191	21.64 ± 5.34	8577.79 ± 757.15	4.01821E-05	135.53 ± 56.36	21869.65 ± 2839.18	0.000187134
**MMP-1**	24.36 ± 0.92	1696.48 ± 658.33	0.01170	0 ± 0	28.16 ± 7.02	0.00225	1.28 ± 0.43	561.27 ± 30.56	5.87702E-06	436.05 ± 121.85	19212.22 ± 2488.11	0.000198719
**IGFBP2**	520.12 ± 89.36	9152 ± 951.56	0.00010	877.37 ± 493.19	10422.07 ± 2821.64	0.00447	349.8 ± 96.29	1969.11 ± 291.82	0.000799493	627.29 ± 131.34	1784.14 ± 204.68	0.001183488
**IL-1a**	2.33 ± 0.84	17.79 ± 4.57	0.00448	0.82 ± 0.31	43.13 ± 2.24	0.00001	1.26 ± 0.43	40.63 ± 5.01	0.000170749	2.33 ± 0.16	19.84 ± 1.64	5.12035E-05
**SERPIN E1/PAI-1**	2607.3 ± 851.99	29765.83 ± 8261.79	0.00479	11.05 ± 4.13	504.89 ± 124.59	0.00236	684.05 ± 250.22	44720.53 ± 4986.36	0.000107071	1441.34 ± 533.1	34955.9 ± 4771.59	0.000268442
**FRACTALKINE/CX3CL1**	767.04 ± 169.14	3556.98 ± 920.05	0.00667	19.49 ± 33.76	1260.32 ± 111.77	0.00005	262.57 ± 109.08	4832.41 ± 553.23	0.000149454	506.15 ± 114.25	4370.16 ± 579.57	0.000345998
**M-CSF**	413.41 ± 61.85	3643.88 ± 769.06	0.00192	96.52 ± 39.13	2201.5 ± 189.01	0.00005	213.97 ± 51.91	2732.29 ± 333.63	0.000207079	403.57 ± 123.27	4395.02 ± 574.56	0.000298663
**ICAM-1/CD54**	7743.53 ± 1771.04	28167.33 ± 6138.6	0.00520	872.01 ± 412.86	8072.1 ± 1362.07	0.00094	3542.1 ± 1095.24	41159.06 ± 4433.07	0.000140141	6923.7 ± 2148.49	34598.13 ± 4360.45	0.000593335
**TARC/CCL17**	52.35 ± 22.07	224.73 ± 52.27	0.00625	28.15 ± 11.19	53.6 ± 6.36	0.02664	27.29 ± 13.27	292.11 ± 33.24	0.000213658	45.52 ± 21.25	267.94 ± 38.32	0.000922892
**TNF RII**	1.97 ± 0.42	13.09 ± 3.72	0.00676	0 ± 0	1.44 ± 0.03	0.00000	1.81 ± 0.4	37.9 ± 3.5	5.93599E-05	3.74 ± 1.22	62.13 ± 6.66	0.000117311
**HGF**	6.35 ± 2.22	39.11 ± 10.04	0.00527	0.22 ± 0.38	187.39 ± 19.45	0.00008	9.06 ± 2.42	187.29 ± 13.37	2.22067E-05	98.35 ± 23.19	4493.57 ± 643.87	0.000293661
**GROa/CXCL1**	108.96 ± 6.33	287.95 ± 63.59	0.00833	61.75 ± 32.79	20226.38 ± 4412.89	0.00138	63.39 ± 16.82	823.6 ± 52.61	1.83544E-05	103.74 ± 31.19	345.76 ± 46.44	0.001697111
**GDF-15**	0.82 ± 0.26	8160.58 ± 1782.21	0.00137	1.08 ± 0.09	1392.72 ± 403	0.00393	1.07 ± 0.3	1551.03 ± 108.54	1.58602E-05	1.14 ± 0.2	25 ± 2.23	5.03032E-05
**Fas/CD95**	5.8 ± 1.13	362.35 ± 74.58	0.00116	4.62 ± 4.67	261.57 ± 27.7	0.00009	8.09 ± 2.2	278.99 ± 29.77	9.57297E-05	6.93 ± 2.87	511.2 ± 44.64	4.05634E-05
Eotaxin/CCL11	21.89 ± 4	31.39 ± 6.88	0.10765	31.26 ± 1.94	24.24 ± 2.23	0.01477	20.21 ± 4.29	37.63 ± 8.33	0.032237472	34.52 ± 10.22	27.52 ± 1.17	0.303782008
MIP-3b/CCL19	0 ± 0	0 ± 0	-	0 ± 0	0 ± 0	-	0 ± 0	0 ± 0	-	0 ± 0	0 ± 0	-
MIP-3a/CCL20	0 ± 0	1.53 ± 1.38	0.12696	0 ± 0	9.15 ± 4.19	0.01931	0 ± 0	14.72 ± 2.98	0.00102136	0 ± 0	9.94 ± 1.67	0.000495775
GROb/CXCL2	0 ± 0	54.63 ± 13.13	0.00196	0 ± 0	0.25 ± 0.25	0.15401	0 ± 0	1072.21 ± 201.25	0.000766472	0 ± 0	45.96 ± 5.43	0.000126193
IL-1ra	1.06 ± 1.83	4.18 ± 2.79	0.17958	10.7 ± 18.54	0 ± 0	0.37390	9.27 ± 16	2066.58 ± 253.8	0.000150486	0.54 ± 0.93	243.97 ± 25.27	7.58191E-05
PDGF-AB/BB	0 ± 0	19.58 ± 4.17	0.00124	0 ± 0	0 ± 0	-	0 ± 0	0 ± 0	-	0 ± 0	0 ± 0	-
TRAIL	4.02 ± 1.18	5.92 ± 0.87	0.08877	4.61 ± 1.26	4.75 ± 1.34	0.90101	3.77 ± 1.14	9.67 ± 1.16	0.003290039	4.53 ± 1.06	5.1 ± 0.66	0.479480859
VEGF	29.48 ± 5.11	845.94 ± 162.79	0.00097	3.46 ± 0.71	7.48 ± 2.83	0.07575	162.88 ± 41.13	3704.27 ± 304.4	3.71078E-05	114.77 ± 33.2	3039.9 ± 353.08	0.000139447
IL-10	0.87 ± 0.11	1.48 ± 0.36	0.04782	1.31 ± 0.21	1.36 ± 0.05	0.74675	0.76 ± 0.16	2.95 ± 0.68	0.005710688	1.26 ± 0.19	1.23 ± 0.1	0.803532779
TNF-a HS	0 ± 0	0.48 ± 0.22	0.01902	0 ± 0	0.01 ± 0.01	0.37390	0 ± 0	1.18 ± 0.21	0.000676002	0 ± 0	0.07 ± 0.06	0.13332299
IL-1b	0.11 ± 0.02	0.62 ± 0.18	0.00911	0.16 ± 0.03	10.88 ± 1.09	0.00007	0.14 ± 0.04	64.52 ± 3.26	4.35553E-06	0.15 ± 0.04	0.2 ± 0.02	0.103139062
IL-13 HS	1.9 ± 0.25	2.33 ± 0.57	0.29908	2.83 ± 0.37	2.48 ± 0.11	0.18088	1.72 ± 0.33	8.75 ± 1.44	0.001185902	2.77 ± 0.53	2.3 ± 0.34	0.257361498
IL-23 HS	0.1 ± 0.08	0 ± 0	0.11861	0.33 ± 0.39	0.15 ± 0.26	0.54974	0.1 ± 0.17	7.65 ± 1.53	0.001043882	0.26 ± 0.24	0 ± 0	0.125541339
MDC/CCL22	44.01 ± 7.39	50.49 ± 12.49	0.48236	67.42 ± 9.53	51.73 ± 2.59	0.05128	39.12 ± 8.53	68.5 ± 12.89	0.030177081	61.91 ± 12.39	49.89 ± 7.81	0.22808141
RAGE/ANGER	3.77 ± 1.51	16.48 ± 3.43	0.00419	7.16 ± 2.28	13.5 ± 5.85	0.15485	2.11 ± 1.04	13.86 ± 1.06	0.000165344	4.08 ± 1.02	14.68 ± 3.01	0.004478718
VCAM-1/CD106	0 ± 0	0 ± 0	-	0 ± 0	0 ± 0	-	0 ± 0	0 ± 0	-	0 ± 0	4927.14 ± 528.77	8.62092E-05
CRP	6.98 ± 0.99	9.08 ± 1.7	0.13897	10.14 ± 1.51	8.43 ± 0.18	0.12121	6.22 ± 1.51	10.94 ± 1.71	0.023263445	9.69 ± 1.92	8.09 ± 1.16	0.284725067

Appendix Table S2: Clinical and molecular information of GBM Patient-Derived Xenografts

	SEX	Age	Primary or Recurrent	Subtype	MGMT methylation	CDKN2A status	PTEN	TP53 status	EGFR	Met	Tert Prom
**6**	Male	65	Primary	C	No	Homodel	loss	Mut	A (viii)	A	Mut (C228T)
**12**	Male	68	Primary	M	Yes	Homodel	-	Mut	A	-	Mut (C250T)
**43**	Male	69	Primary	M	No	Homodel	-	Mut		-	Mut (C228T)
**123**	Female	62	Primary	C	No	-	-	Mut	A	-	Mut (C250T)
**148**	Female	84	Primary	P	No	Deepdel	Mut	-	gain	-	Mut (C228T)
**245**	Male	54	Primary	NA	NA	-	-	Mut	A	-	NA

Mut = mutation
A = amplification
Homodel = homozygous deletion
Deepdel = deep deletion
C = Classical
M = Mesenchymal
P = Proneural
NA = not available
- = wild type

Appendix Table S3: Antibody information

Antibody	Vendor	Cat#	WB	IF
Lamin B1	Abcam	ab16048		1:4000
Ki67	Abcam	ab15580		1:1000
BrdU	Becton Dickinson	347580		1:200
p21	Cell Signaling Technology	2947	1:1000	
p16	Cell Signaling Technology	80772	1:1000	
Actin	Proteintech	HRP-66009	1:5000	
phospho-p65 S536	Cell Signaling Technology	3033	1:1000	
p65	Cell Signaling Technology	8242	1:1000	
phospho-STAT3 Y705	Cell Signaling Technology	9145	1:2000	
STAT3	Cell Signaling Technology	12640	1:1000	
cIAP2	Abcam	ab23423	1:1000	
cIAP2	Cell Signaling Technology	3130	1:1000	
cIAP1	Cell Signaling Technology	7065	1:1000	
Caspase-8	Cell Signaling Technology	9746	1:1000	
HRP-conjugated secondary antibodies (mouse)	Cell Signaling Technology	7076	1:3000	
HRP-conjugated secondary antibodies (rabbit)	Cell Signaling Technology	7074	1:3000	
Alexa488-conjugated secondary antibodies (rabbit)	Invitrogen	A11034		1:1000
Alexa568-conjugated secondary antibodies (mouse)	Invitrogen	A11004		1:1000

Appendix Table S4: Exact p values

Fig.1-d	LN229	A172	U118	U87
CCL2	0.00017926	0.000093	0.000002	0.0453275
CXCL3	0.00003661	0.000040	0.000012	0.0004319
CXCL5	0.00189908	0.001664	0.000974	0.0000008
IL1A	0.00016140	0.000343	0.000015	0.0235268
IL1B	0.00000327	0.000001	0.000044	0.0001346
IL6	0.00102839	0.000204	0.000046	0.0089460
IL8	0.00000008	0.000026	0.000022	0.0014614
IL13	0.00000817	0.000026	0.000022	0.0034624
MMP12	0.00059966	0.005851	0.000254	0.0000431
TNFα	0.00084051	0.000036	0.001112	0.0010751

Fig.5-c	GBM6	GBM12	GBM43	GBM123	GBM148	GBM245
CCL2	0.000034	0.0063027	0.0043447	0.00158	0.001872	0.0003980
CXCL3	0.000010	0.0001193	0.0000123	0.00749	0.000175	0.0000731
CXCL5	0.009358	0.0024874	0.0000019	0.00011	0.001952	0.0003280
IL1A	0.068374	0.0001976	0.0000051	0.91957	0.000070	0.0000250
IL1B	0.000005	0.0001859	0.0000008	0.02618	0.000185	0.0000003
IL6	0.000024	0.0000011	0.0000116	0.00004	0.000553	0.0000712
IL8	0.000010	0.0000006	0.0000038	0.00034	0.000061	0.0003710
IL13	0.002964	0.0003212	0.0238809	0.01157	0.000037	0.0277351
MMP12	0.007338	0.0012494	0.0000114	0.00114	0.000002	0.0000155
TNFα	0.008513	0.0120294	0.1146405	0.00344	0.001310	0.0149686

EV2.b	LN229	A172	U118	U87
CCL2	0.0000029	0.000055999	0.000013	0.000003
CXCL3	0.0000009	0.000000006	0.000003	0.000006
CXCL5	0.0081609	0.529722640	0.065743	0.007000
IL1A	0.0000104	0.000003186	0.000099	0.001991
IL1B	0.0000034	0.011488964	0.398208	0.003772
IL6	0.0000003	0.000010399	0.016130	0.004992
IL8	0.0000061	0.000000028	0.000010	0.000004
IL13	0.7500332	0.117342713	0.458628	0.366142
MMP12	0.6141445	0.589831691	0.022948	0.099648
TNFα	0.0036736	0.004577963	0.079307	0.028493
