# Supplementary figures and images for: Targeting cIAP2 in a novel senolytic strategy prevents glioblastoma recurrence after radiotherapy (part 1 of 4)
Source: EMBO Mol Med. 2025 Feb 19;17(4):645–78. doi: 10.1038/s44321-025-00201-x (PMC11982261; doi:10.1038/s44321-025-00201-x)

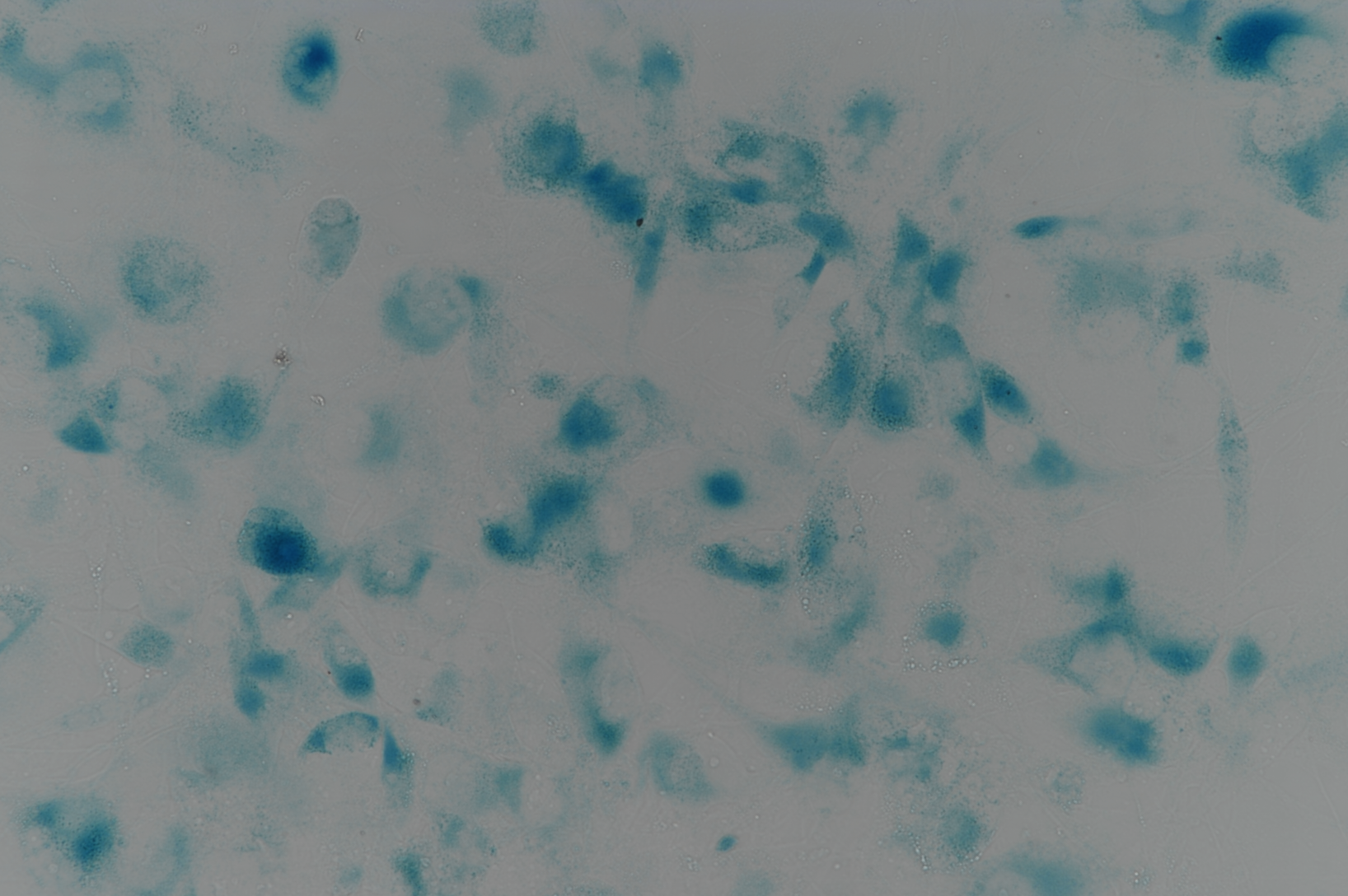

Supplement: Supplementary file 3 — Source data Fig. 1 [file 44321_2025_201_MOESM3_ESM.zip › Fig1/Fig1a/a172 10gyd10_004c1.tif]

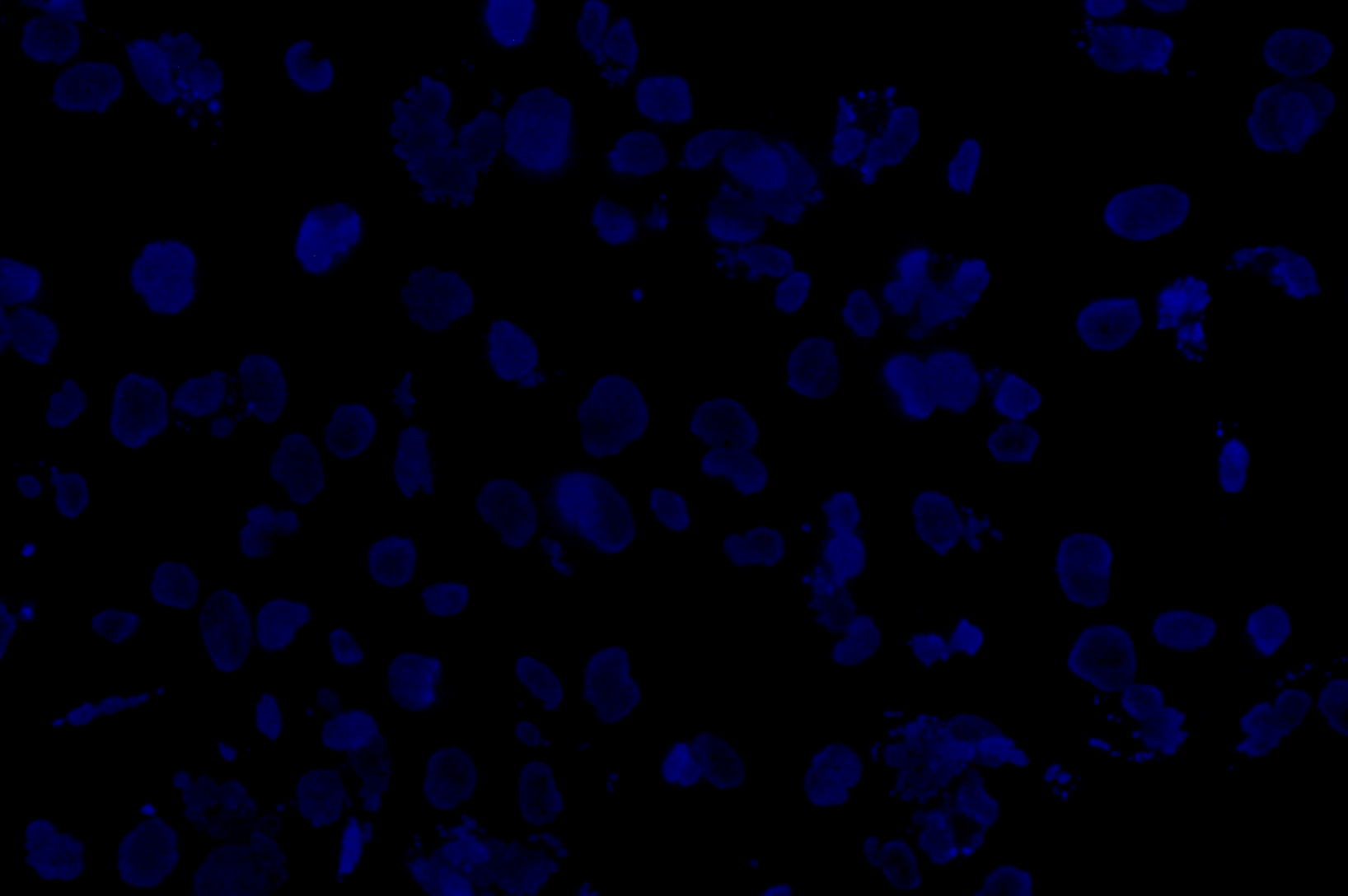

Supplement: Supplementary file 3 — Source data Fig. 1 [file 44321_2025_201_MOESM3_ESM.zip › Fig1/Fig1a/a172 10gyd10_004c2.tif]

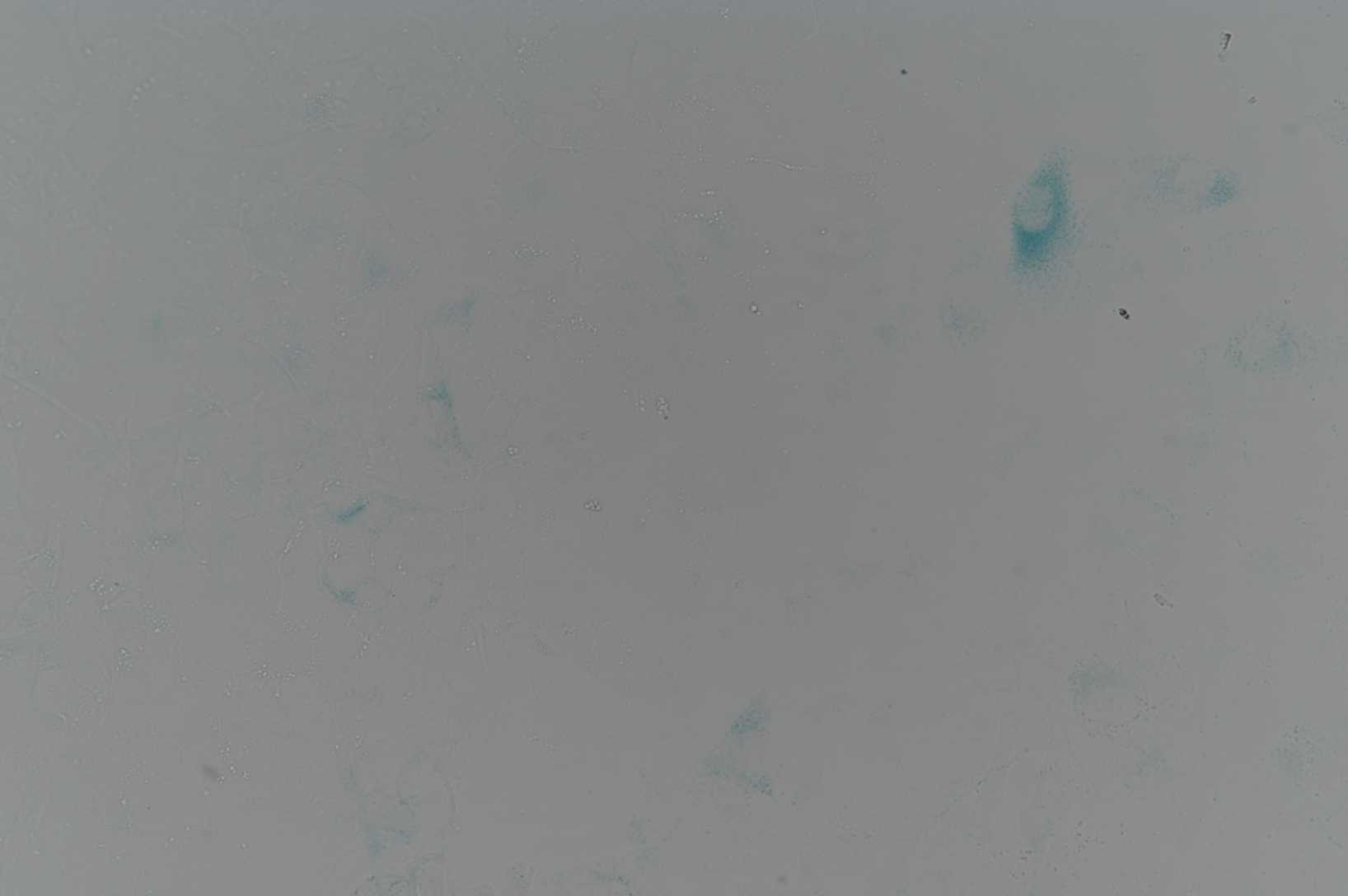

Supplement: Supplementary file 3 — Source data Fig. 1 [file 44321_2025_201_MOESM3_ESM.zip › Fig1/Fig1a/a172 mock_004c1.tif]

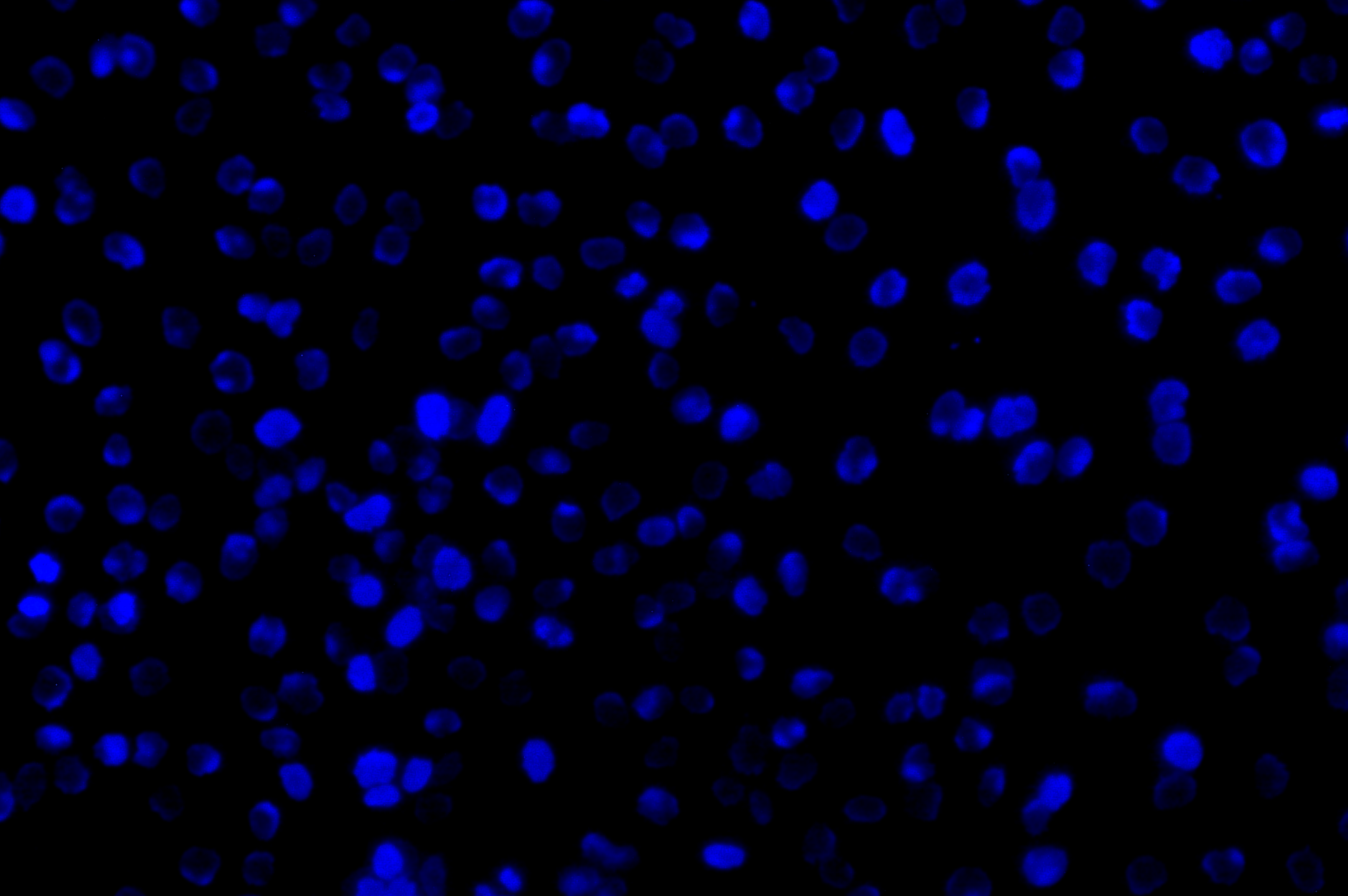

Supplement: Supplementary file 3 — Source data Fig. 1 [file 44321_2025_201_MOESM3_ESM.zip › Fig1/Fig1a/a172 mock_004c2.tif]

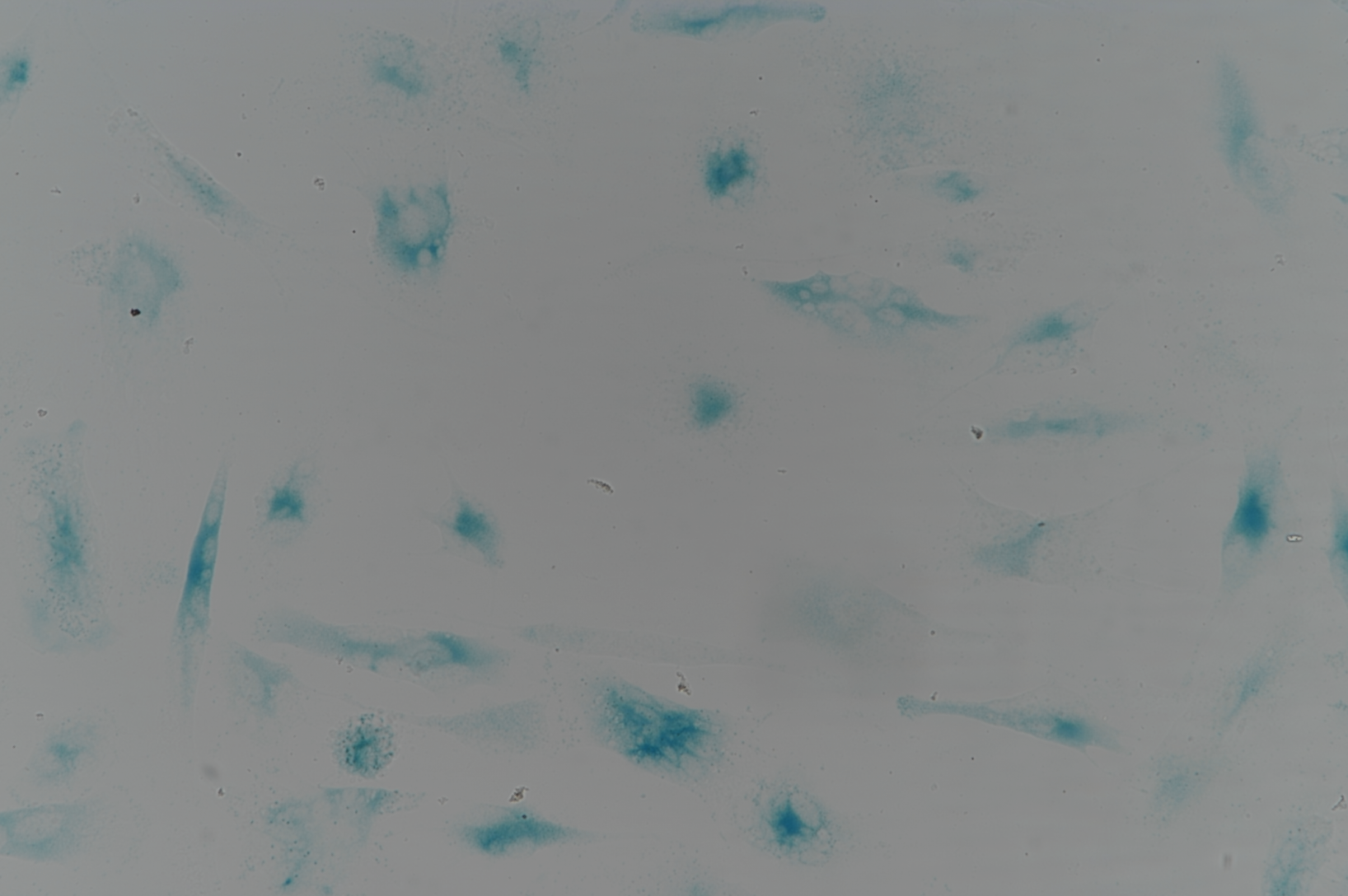

Supplement: Supplementary file 3 — Source data Fig. 1 [file 44321_2025_201_MOESM3_ESM.zip › Fig1/Fig1a/ln229 10gyd10_004c1.tif]

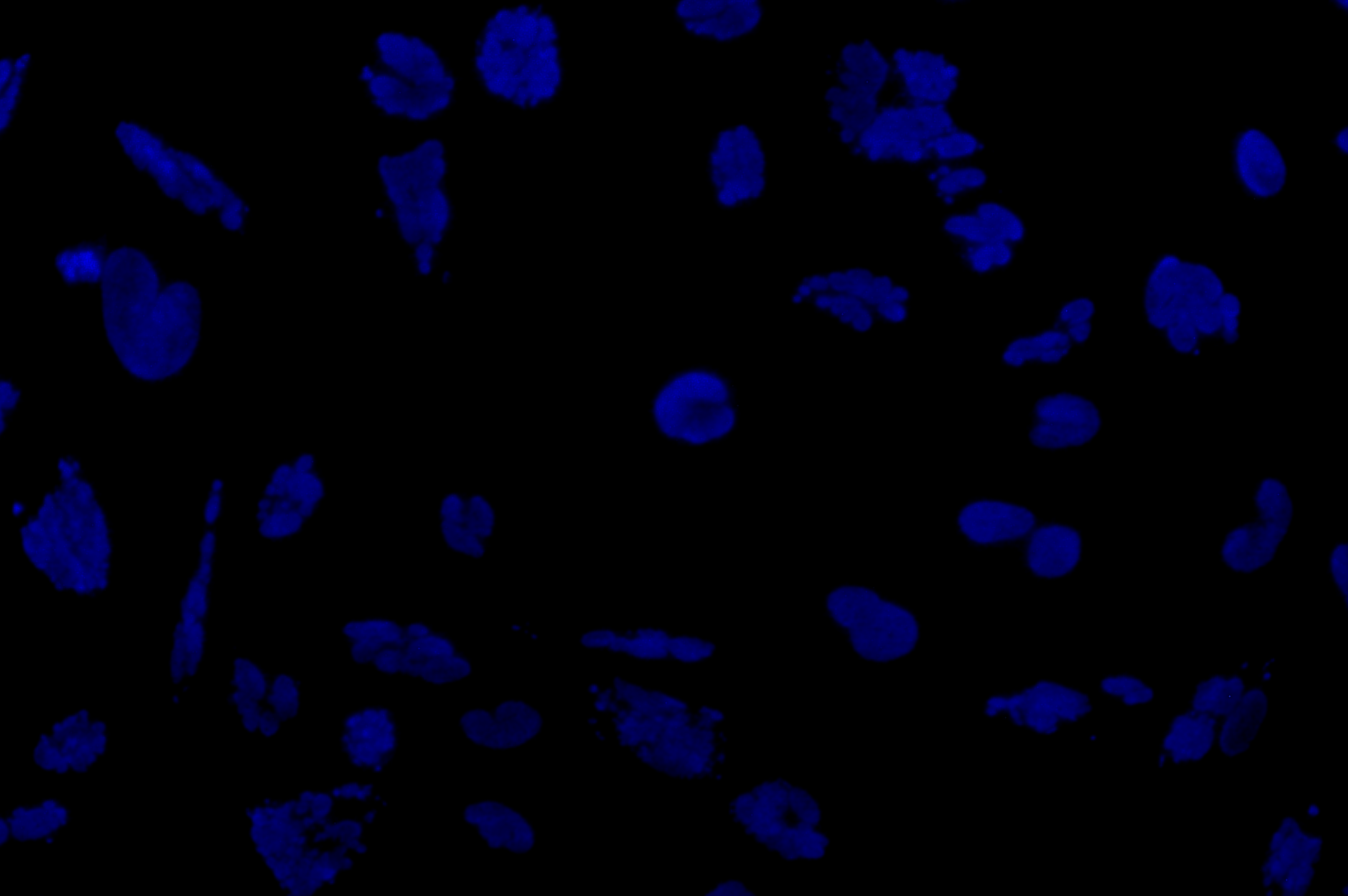

Supplement: Supplementary file 3 — Source data Fig. 1 [file 44321_2025_201_MOESM3_ESM.zip › Fig1/Fig1a/ln229 10gyd10_004c2.tif]

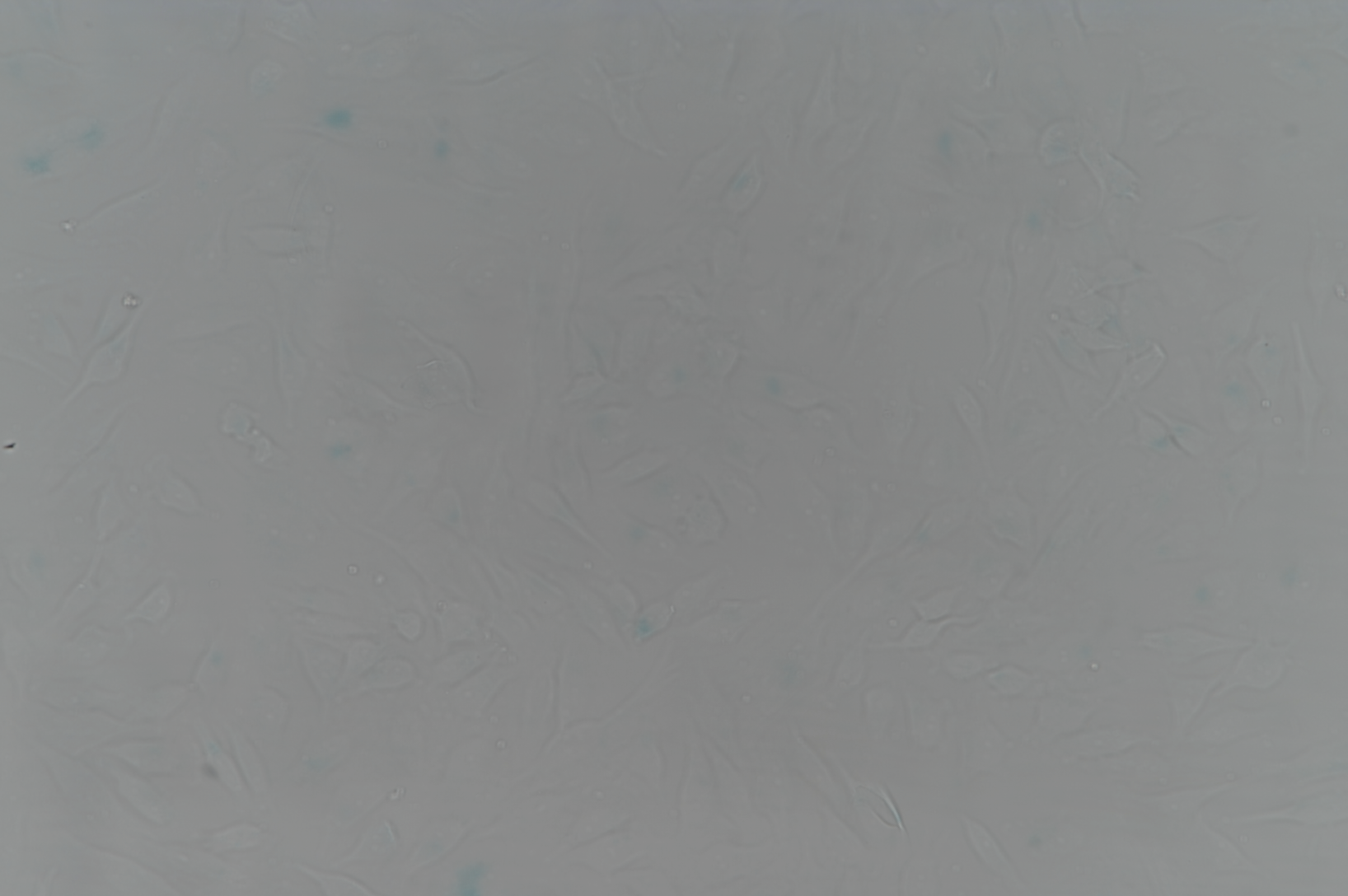

Supplement: Supplementary file 3 — Source data Fig. 1 [file 44321_2025_201_MOESM3_ESM.zip › Fig1/Fig1a/ln229 mock_004c1.tif]

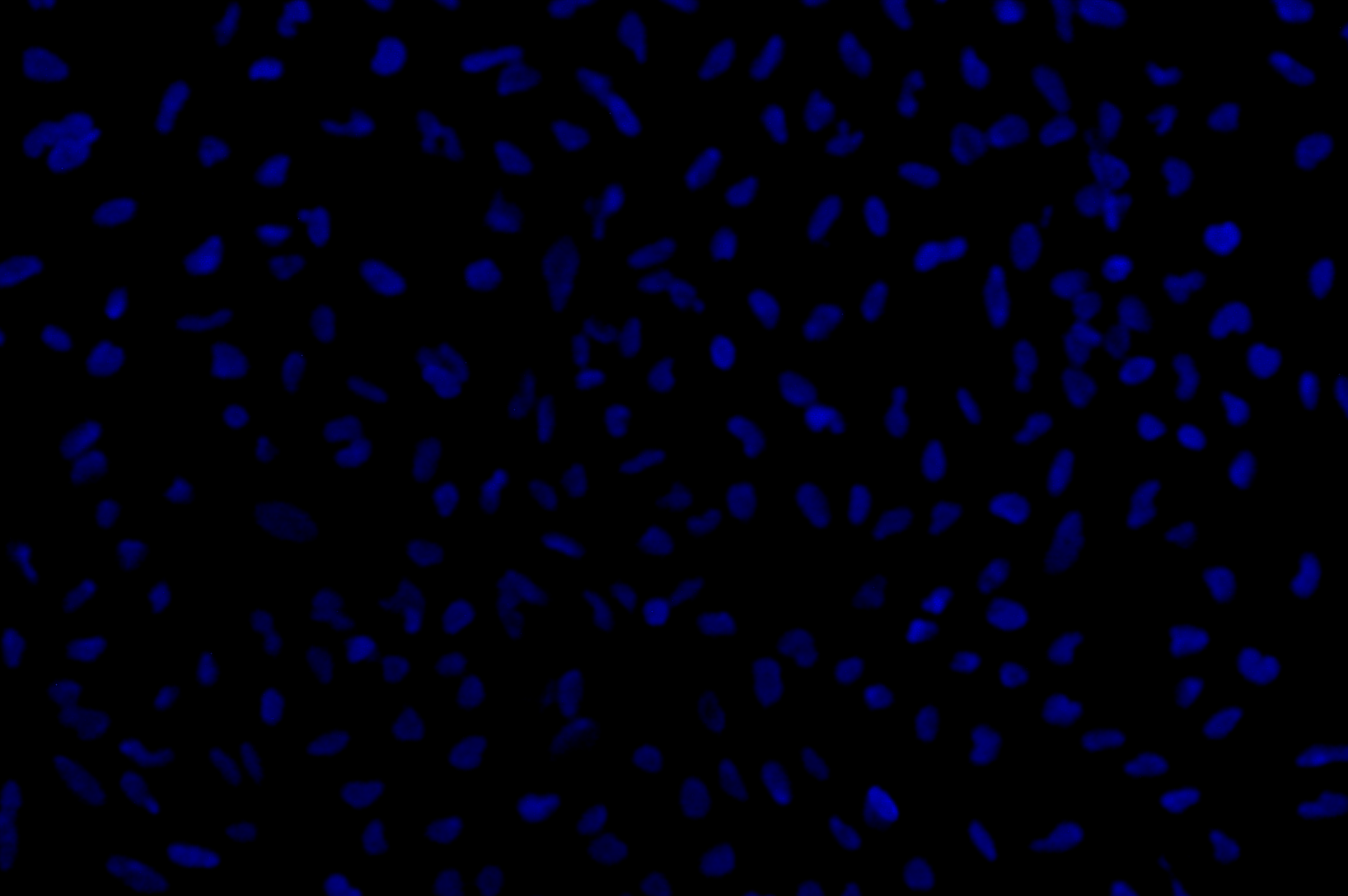

Supplement: Supplementary file 3 — Source data Fig. 1 [file 44321_2025_201_MOESM3_ESM.zip › Fig1/Fig1a/ln229 mock_004c2.tif]

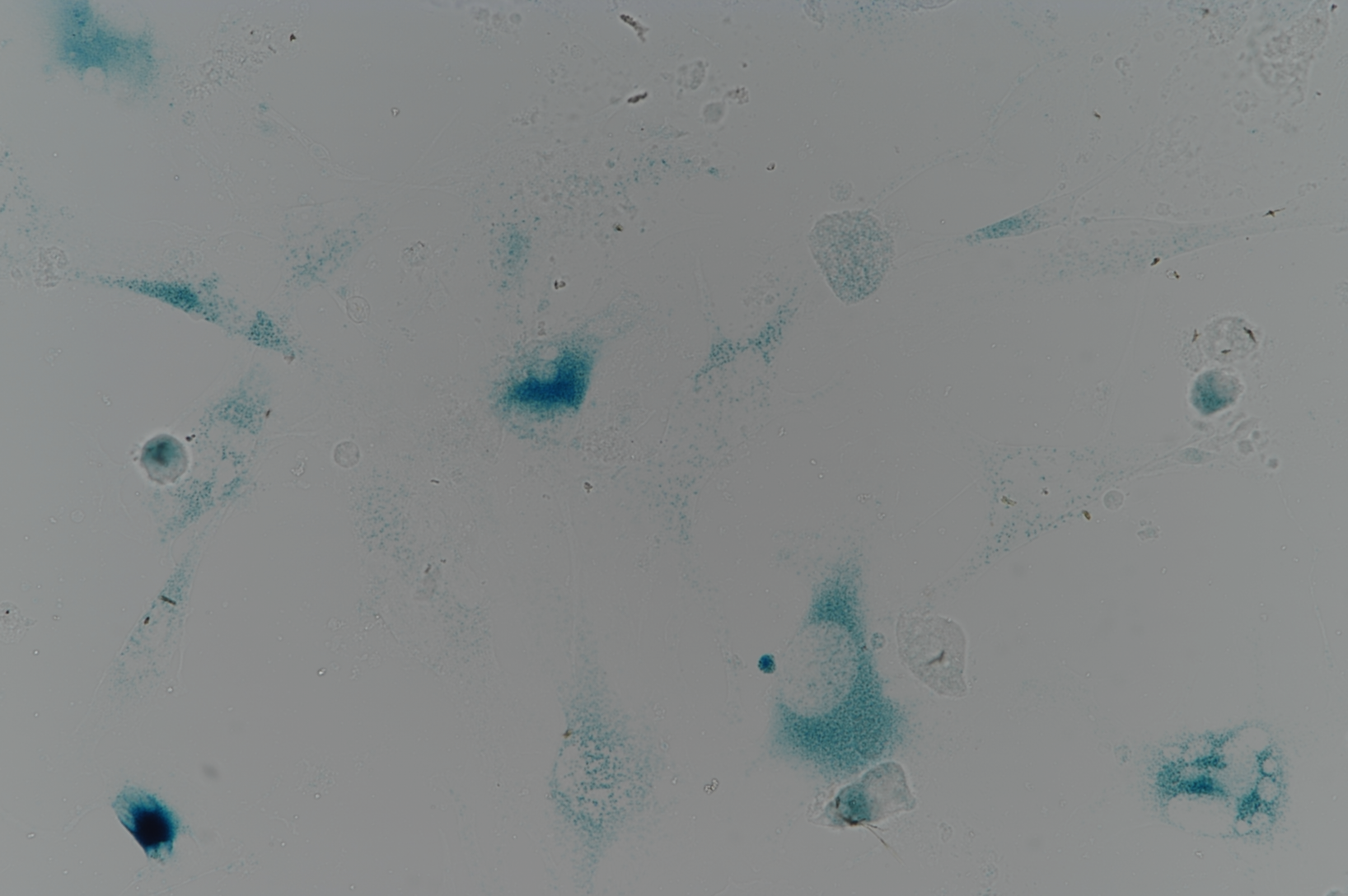

Supplement: Supplementary file 3 — Source data Fig. 1 [file 44321_2025_201_MOESM3_ESM.zip › Fig1/Fig1a/u118 10gyd10_ (4)c1.tif]

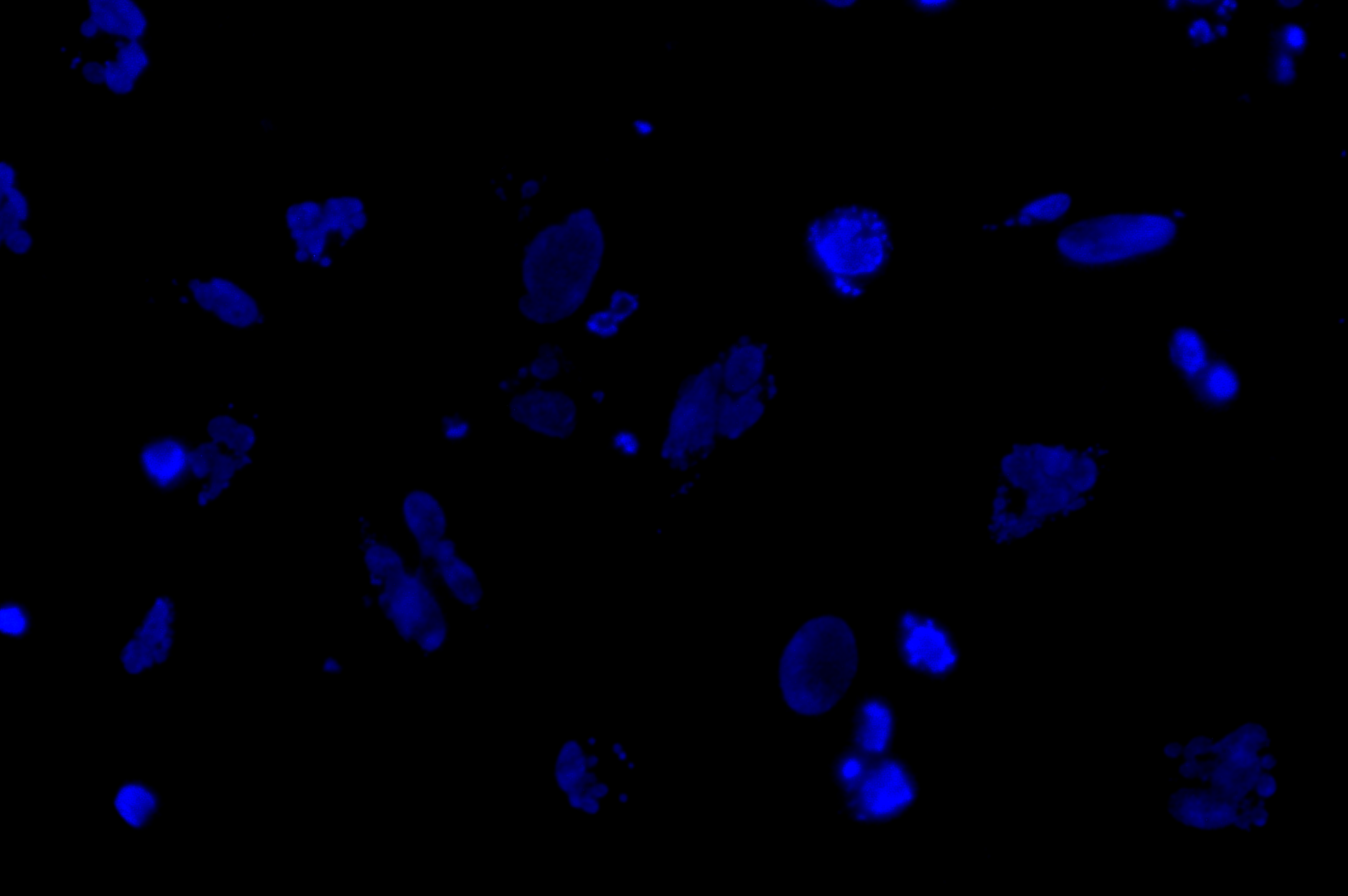

Supplement: Supplementary file 3 — Source data Fig. 1 [file 44321_2025_201_MOESM3_ESM.zip › Fig1/Fig1a/u118 10gyd10_ (4)c2.tif]

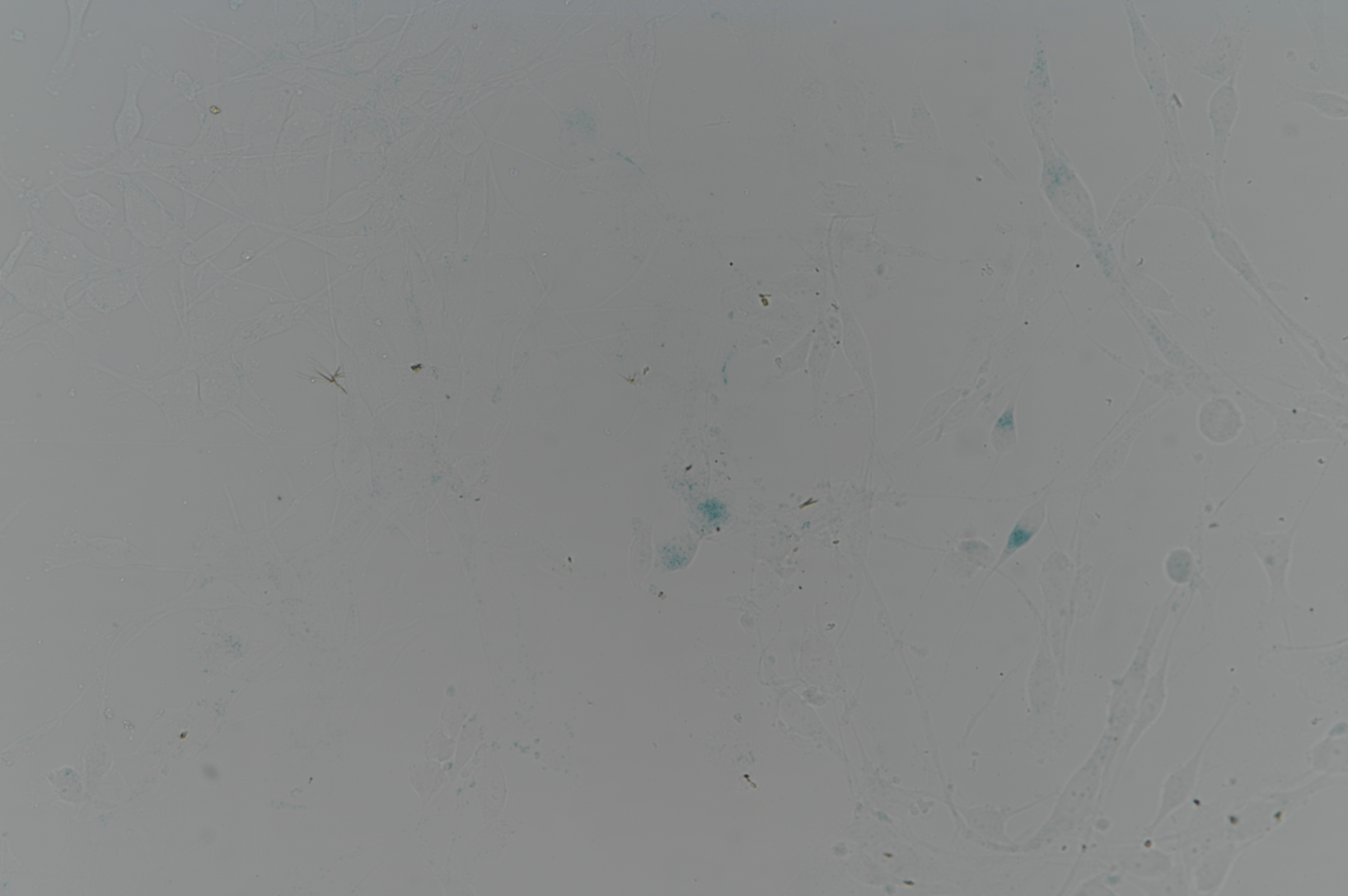

Supplement: Supplementary file 3 — Source data Fig. 1 [file 44321_2025_201_MOESM3_ESM.zip › Fig1/Fig1a/u118 mock_003c1.tif]

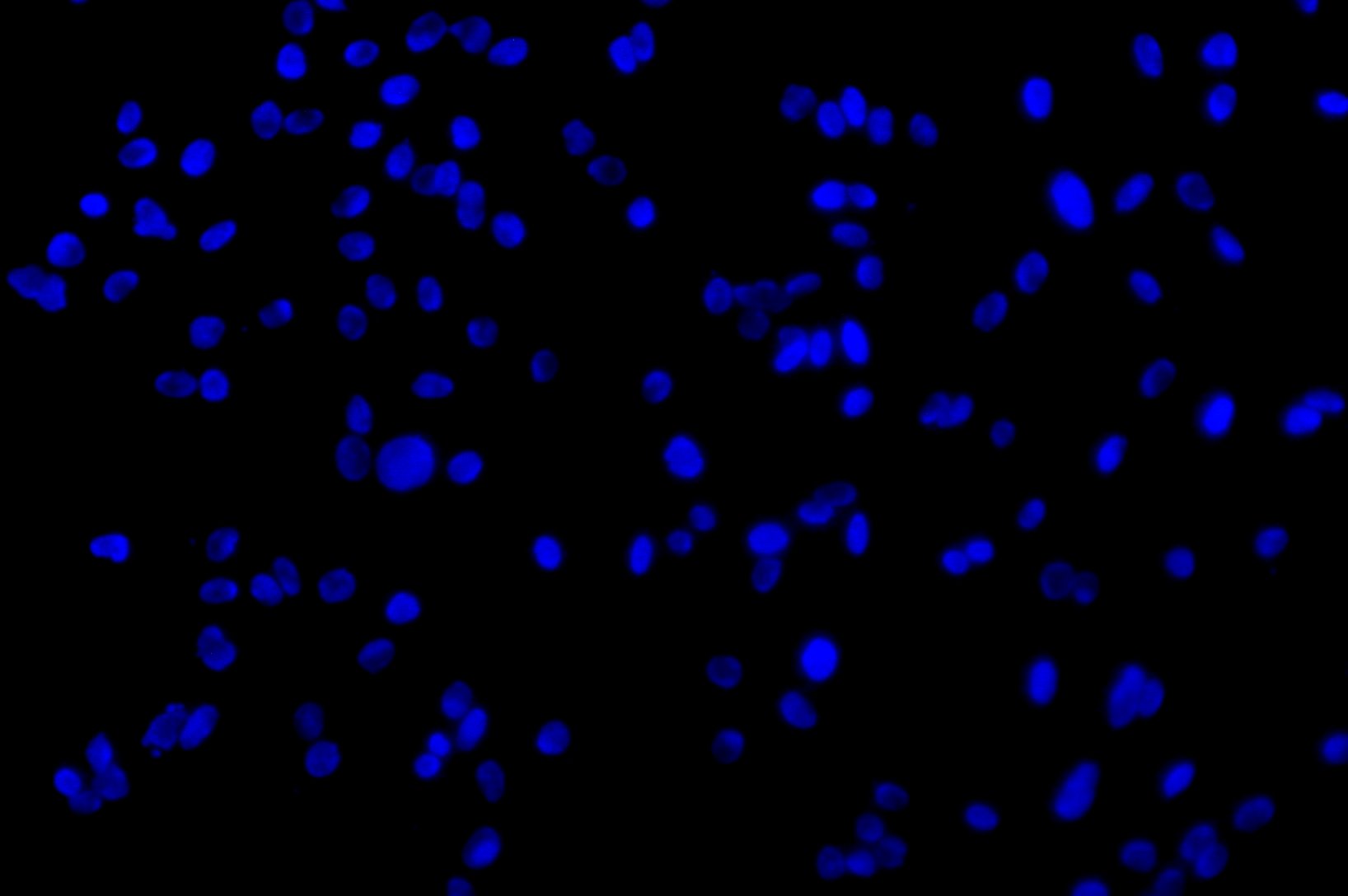

Supplement: Supplementary file 3 — Source data Fig. 1 [file 44321_2025_201_MOESM3_ESM.zip › Fig1/Fig1a/u118 mock_003c2.tif]

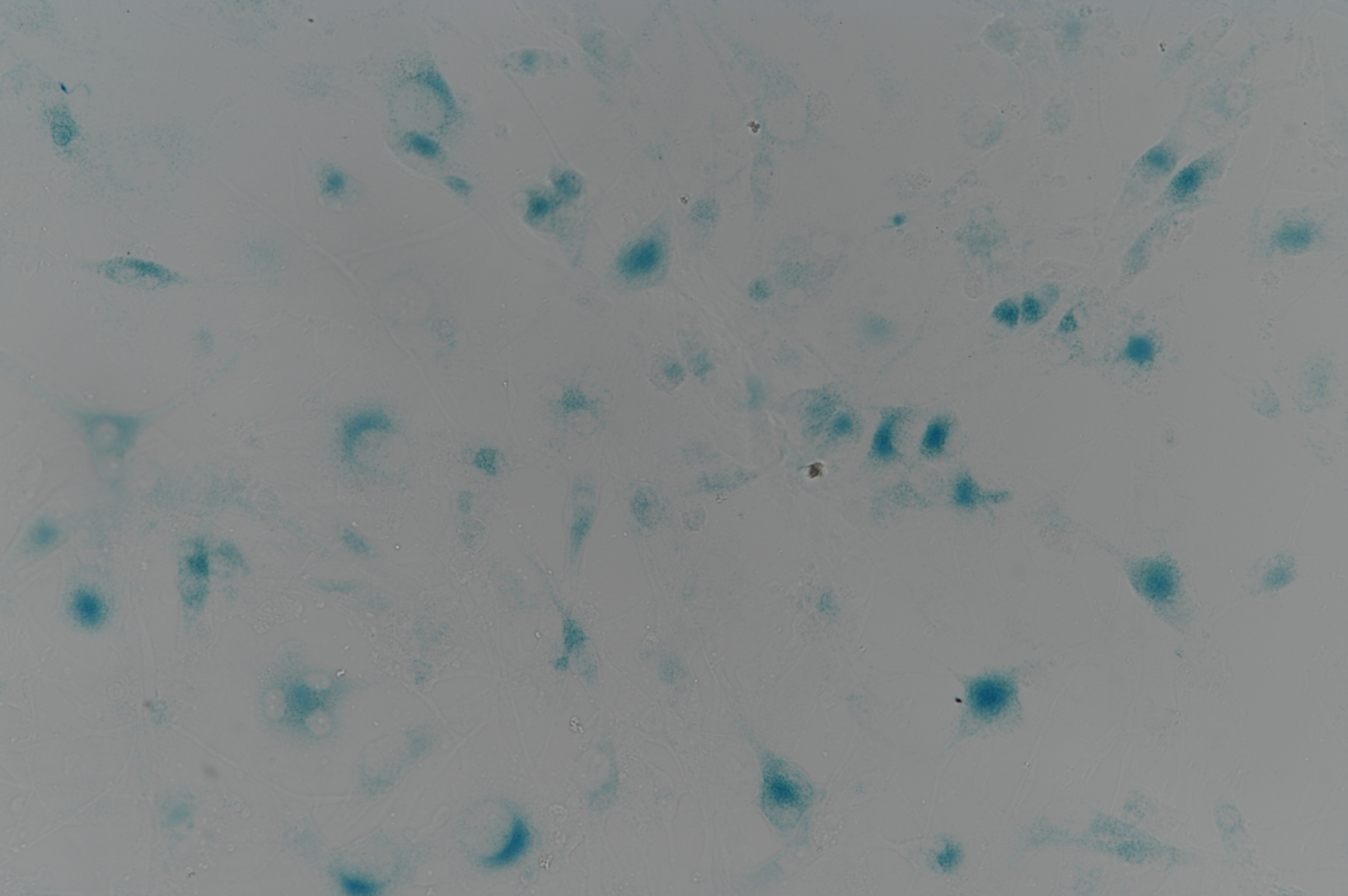

Supplement: Supplementary file 3 — Source data Fig. 1 [file 44321_2025_201_MOESM3_ESM.zip › Fig1/Fig1a/u87 10gyd10_004c1.tif]

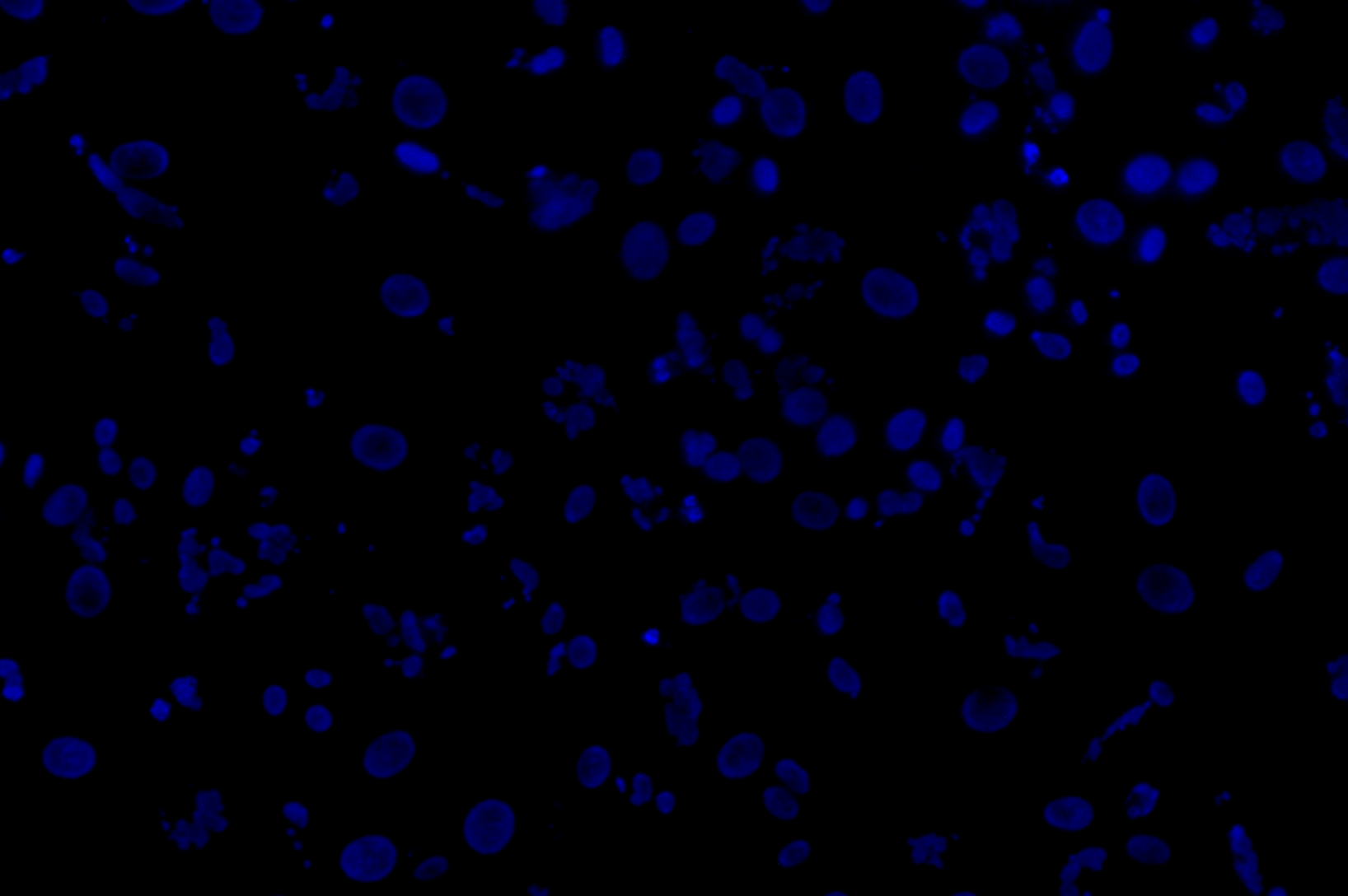

Supplement: Supplementary file 3 — Source data Fig. 1 [file 44321_2025_201_MOESM3_ESM.zip › Fig1/Fig1a/u87 10gyd10_004c2.tif]

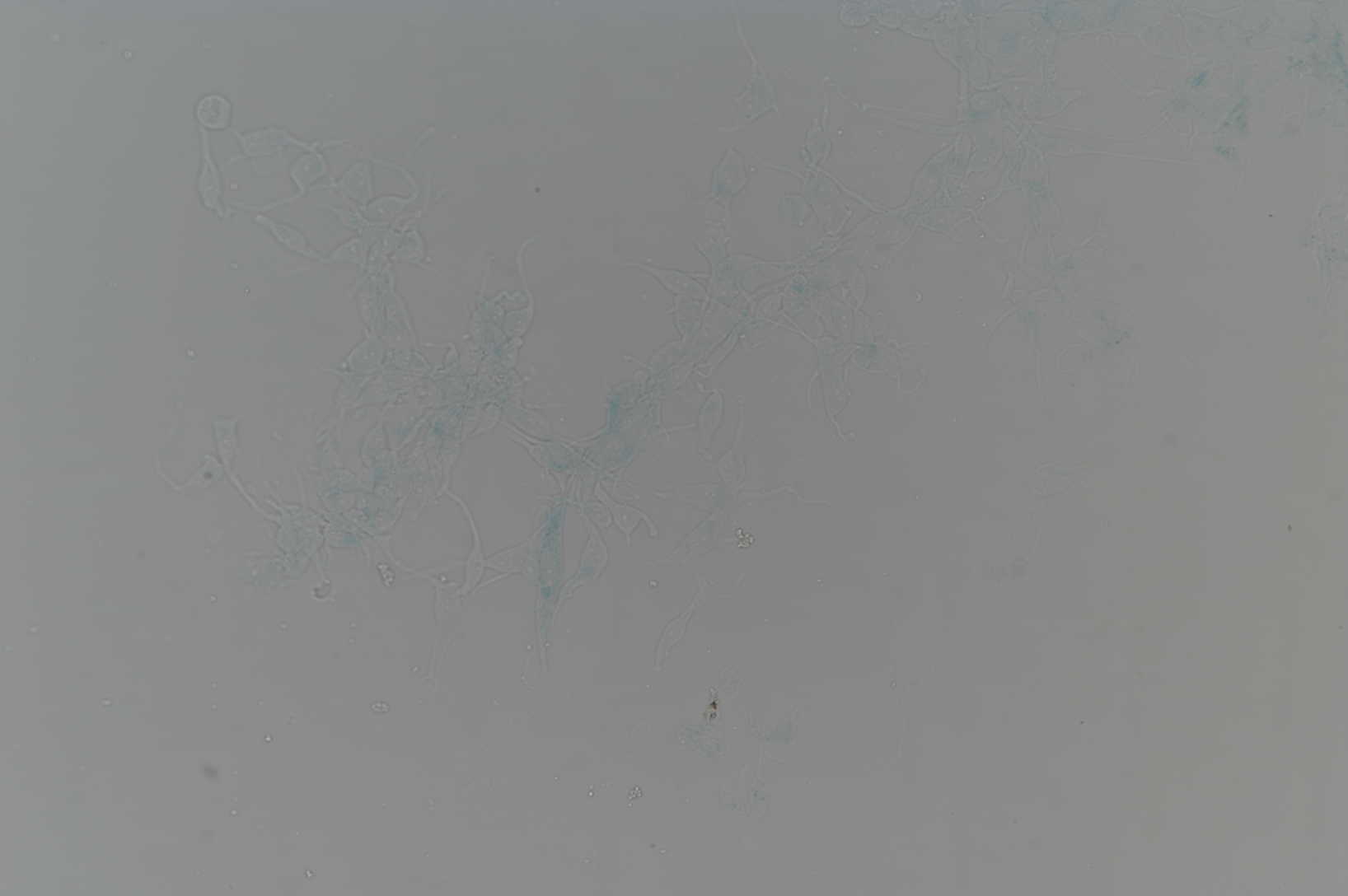

Supplement: Supplementary file 3 — Source data Fig. 1 [file 44321_2025_201_MOESM3_ESM.zip › Fig1/Fig1a/u87 mock_004c1.tif]

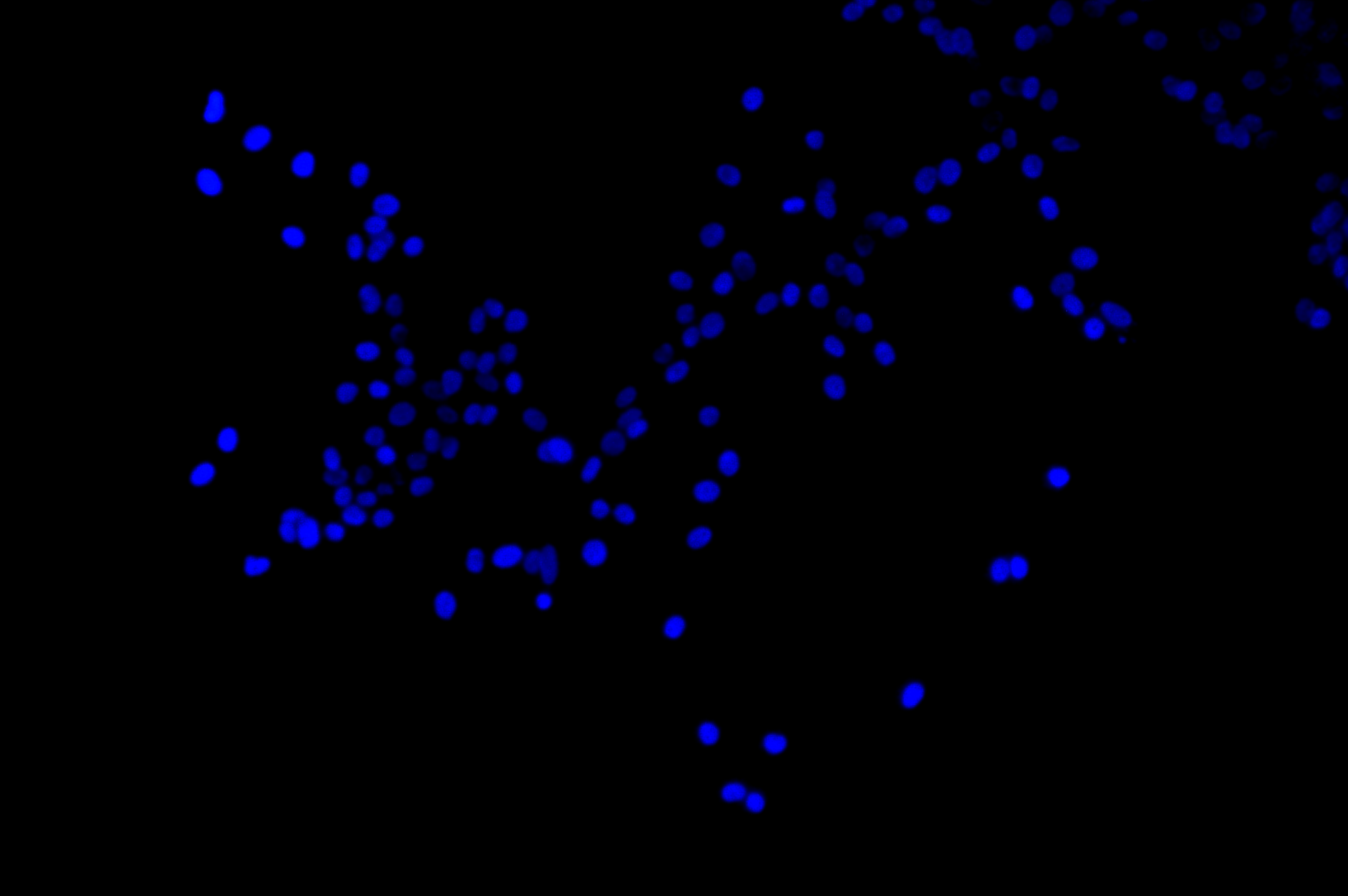

Supplement: Supplementary file 3 — Source data Fig. 1 [file 44321_2025_201_MOESM3_ESM.zip › Fig1/Fig1a/u87 mock_004c2.tif]

Figure 1

b

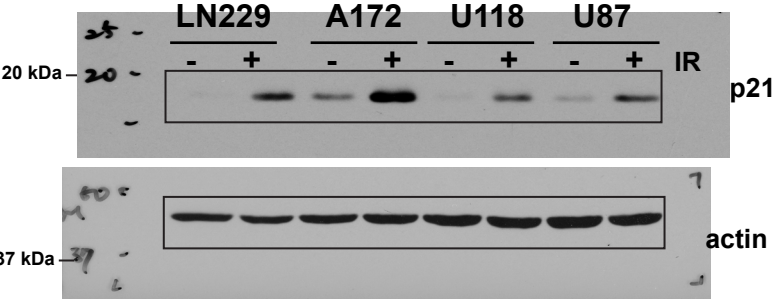

Supplement: Supplementary file 3 — Source data Fig. 1 [file 44321_2025_201_MOESM3_ESM.zip › Fig1/Fig1B-pic.pdf]

**a**

**a**

# LN229

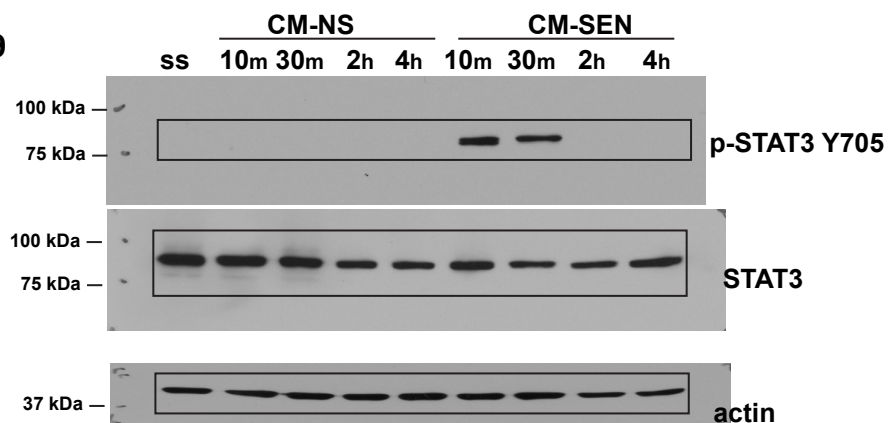

# A172

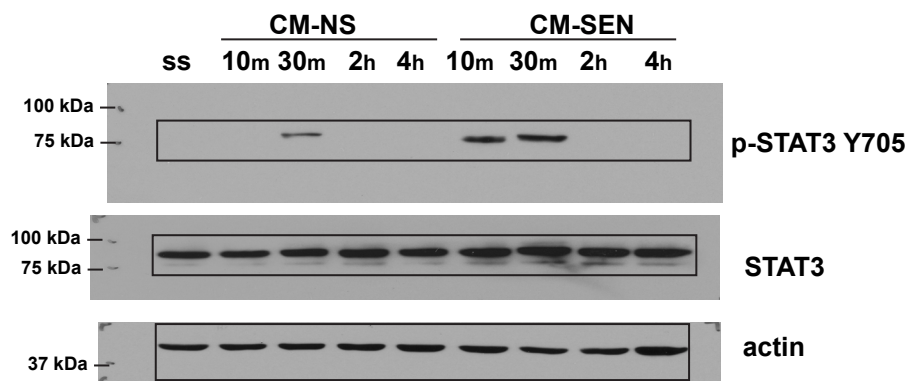

**U118**

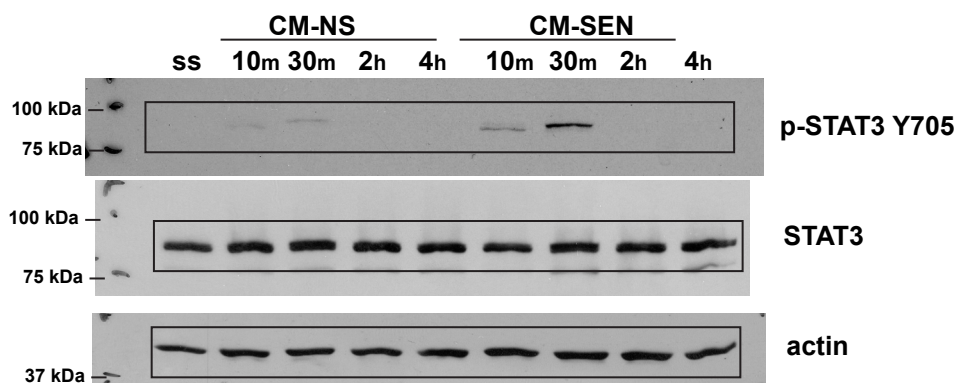

**U87**

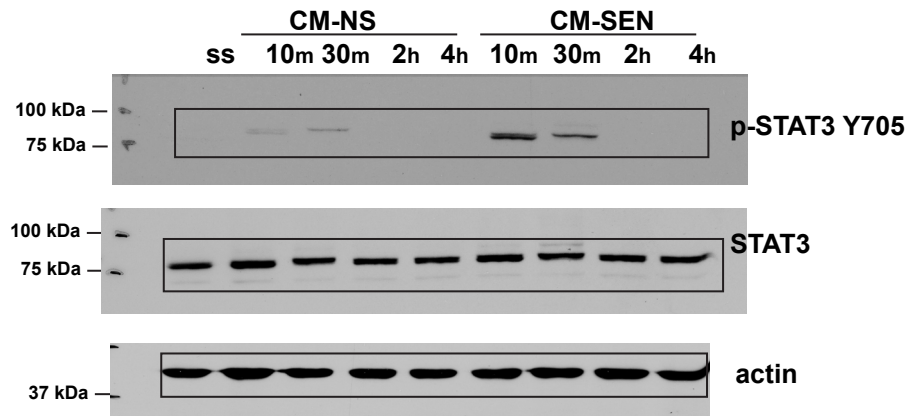

Supplement: Supplementary file 4 — Source data Fig. 2 [file 44321_2025_201_MOESM4_ESM.zip › Fig2/Fig2a-121024.pdf]

Figure 2

b

LN229

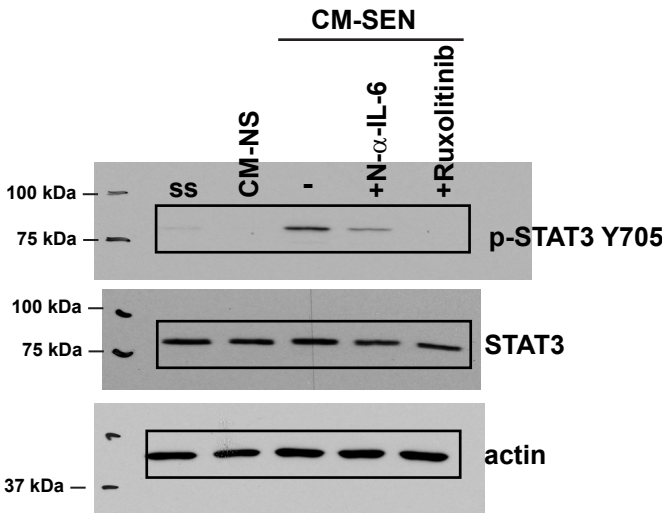

A172

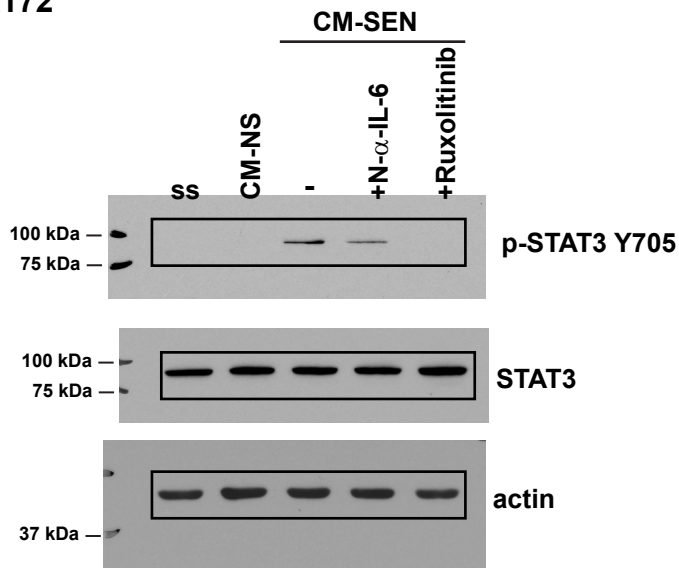

U118

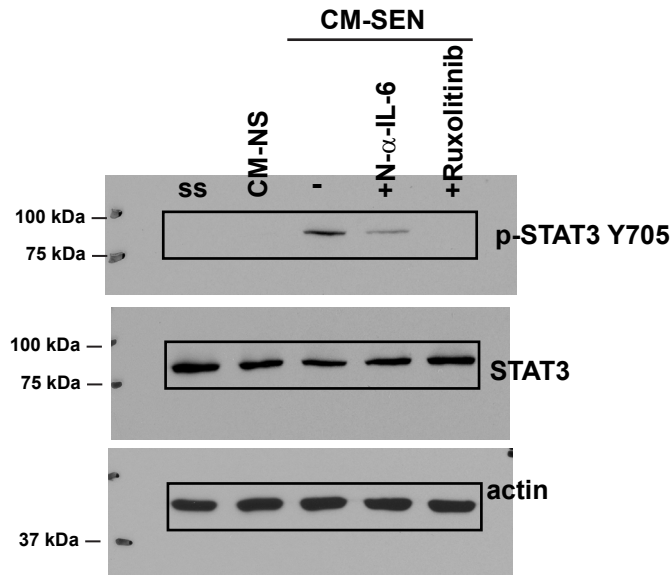

U87

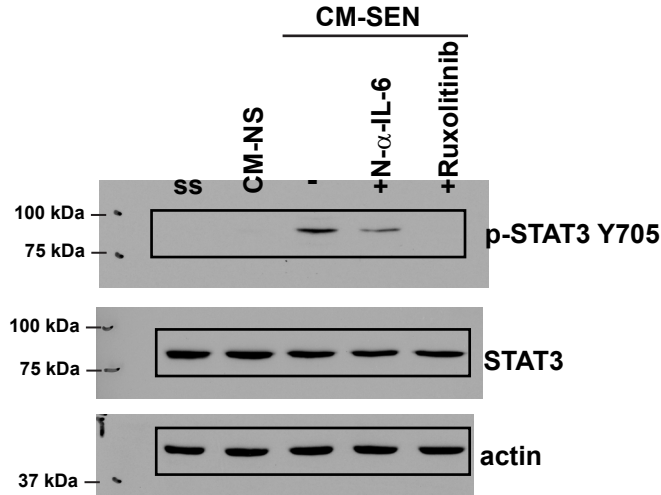

Supplement: Supplementary file 4 — Source data Fig. 2 [file 44321_2025_201_MOESM4_ESM.zip › Fig2/Fig2b.pdf]

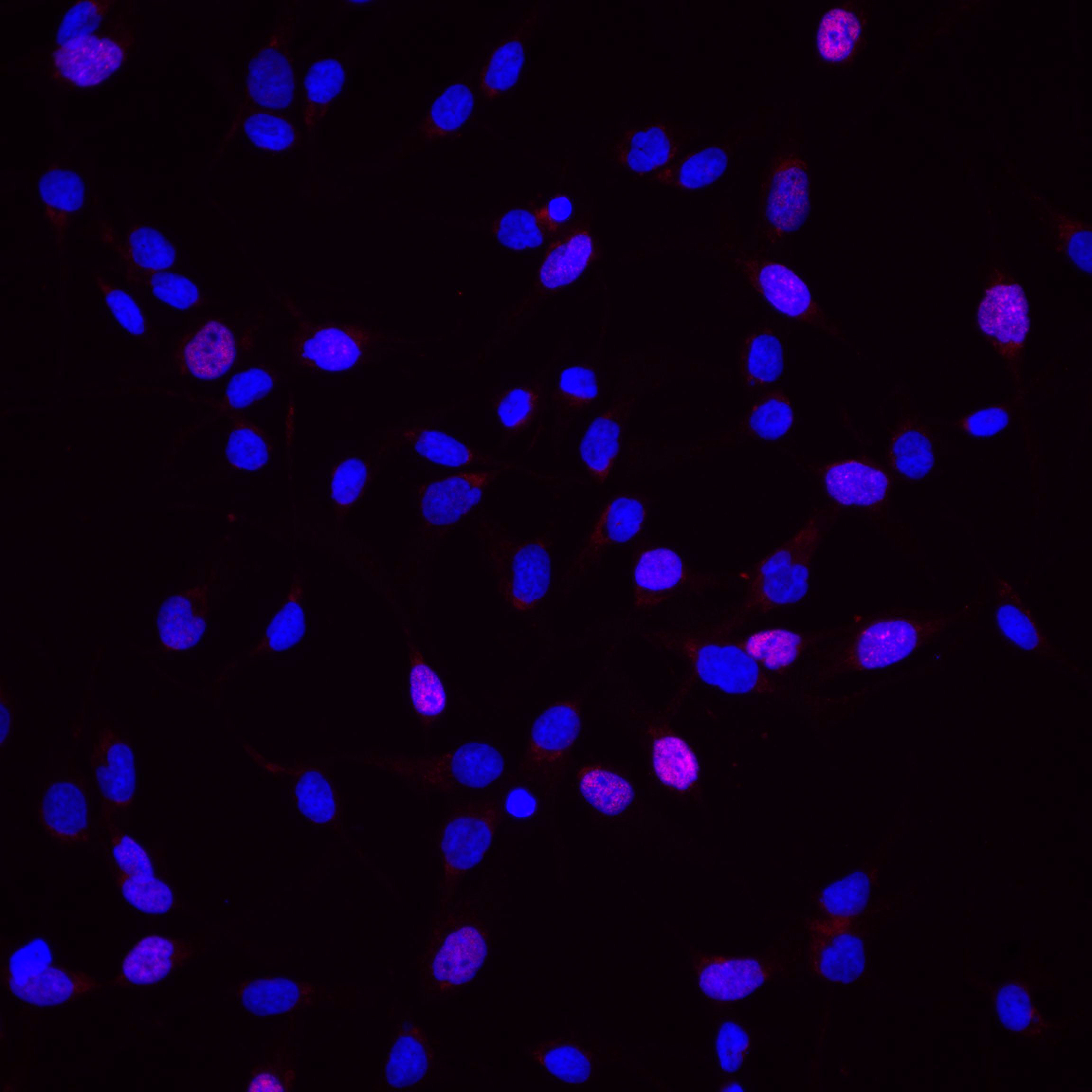

Supplement: Supplementary file 4 — Source data Fig. 2 [file 44321_2025_201_MOESM4_ESM.zip › Fig2/Fig2c BrdU IF/A172/m-A172-3-CM-NS-001.jpg]

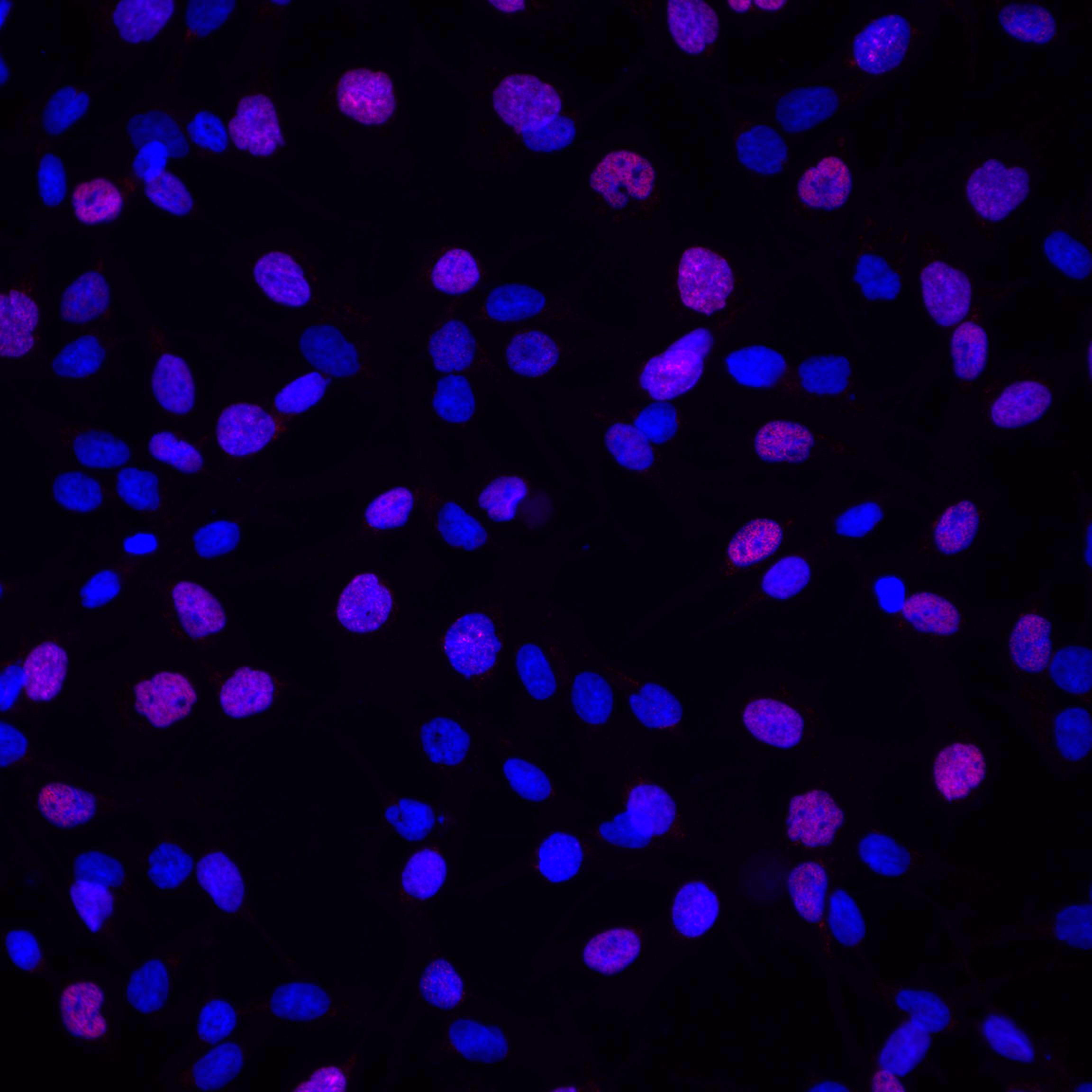

Supplement: Supplementary file 4 — Source data Fig. 2 [file 44321_2025_201_MOESM4_ESM.zip › Fig2/Fig2c BrdU IF/A172/m-A172-3-CM-SEN-004.jpg]

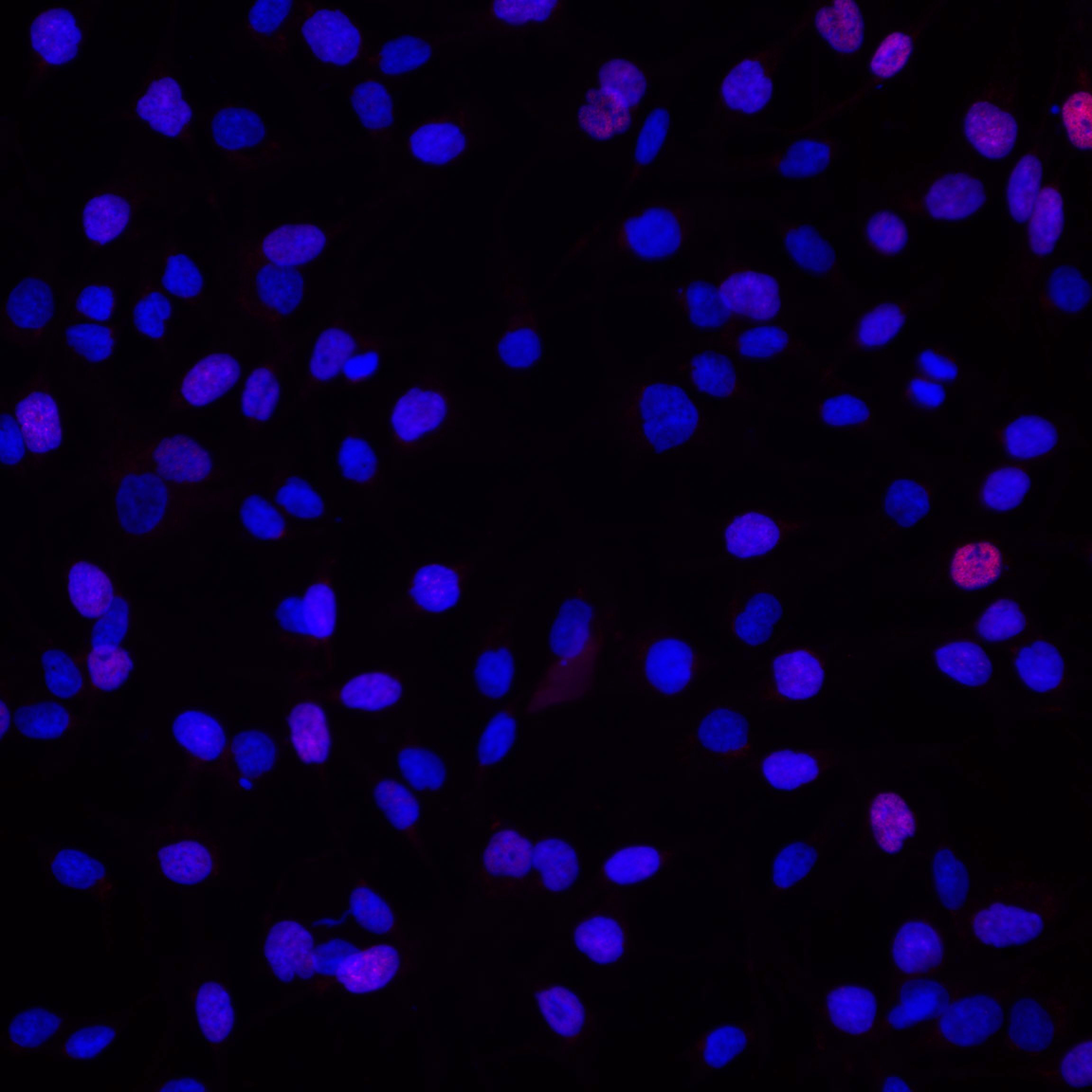

Supplement: Supplementary file 4 — Source data Fig. 2 [file 44321_2025_201_MOESM4_ESM.zip › Fig2/Fig2c BrdU IF/A172/m-A172-3-CM-SEN-Ruxo-006.jpg]

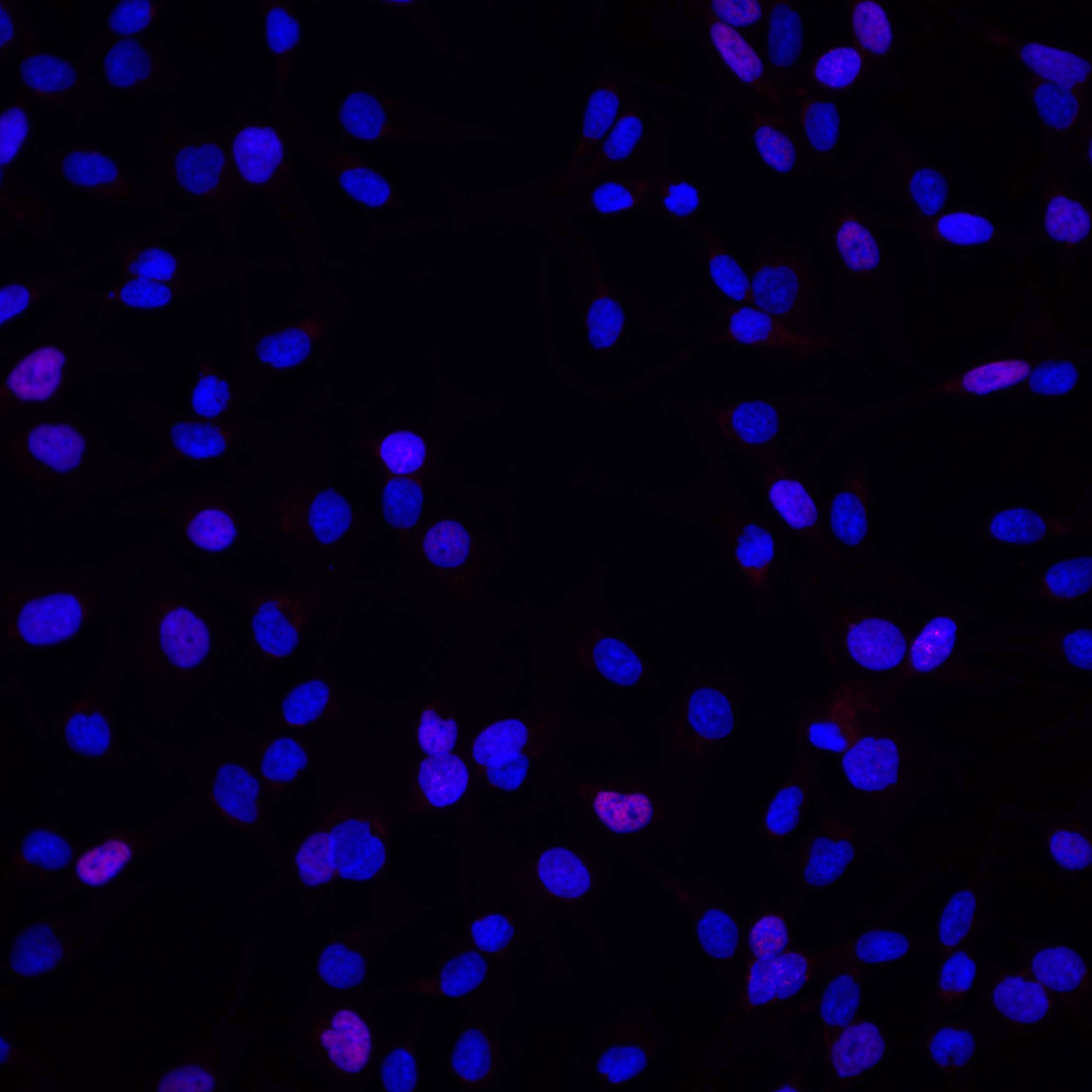

Supplement: Supplementary file 4 — Source data Fig. 2 [file 44321_2025_201_MOESM4_ESM.zip › Fig2/Fig2c BrdU IF/A172/m-A172-3-ss-004.jpg]

Figure 2

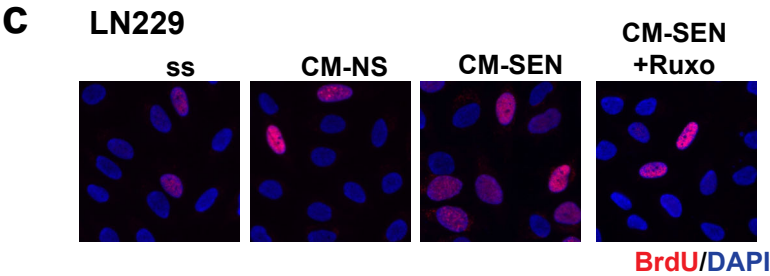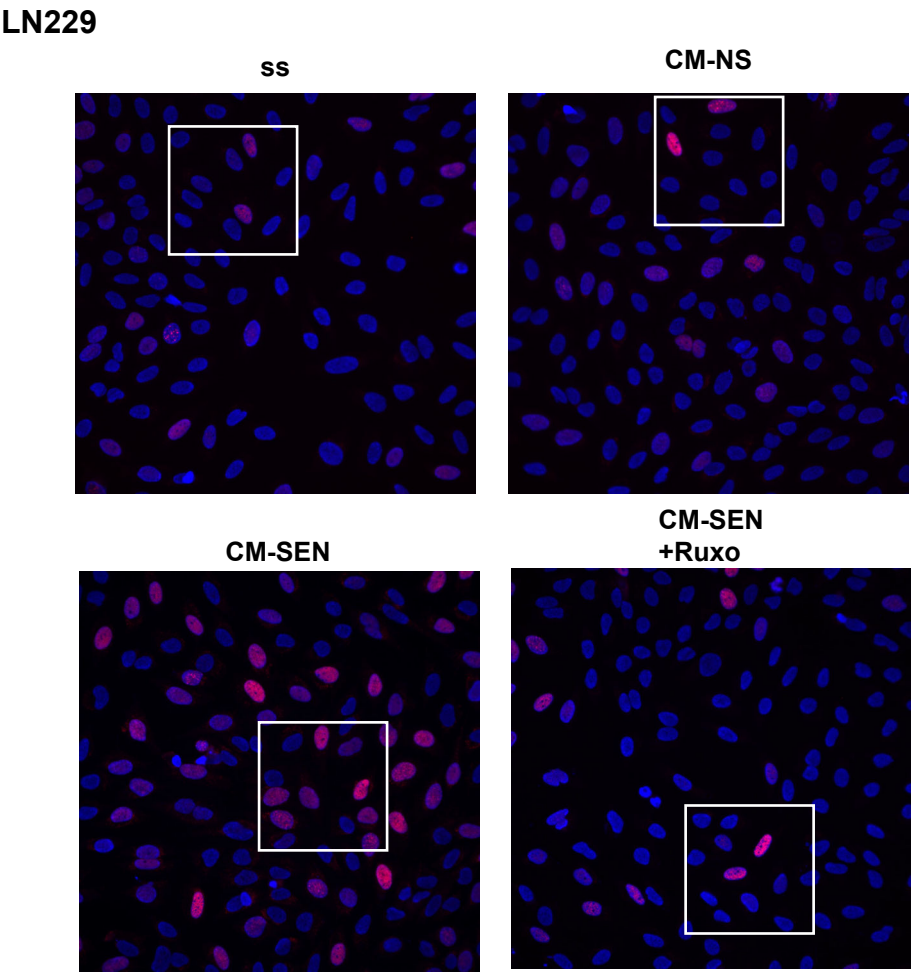

Figure 2

C

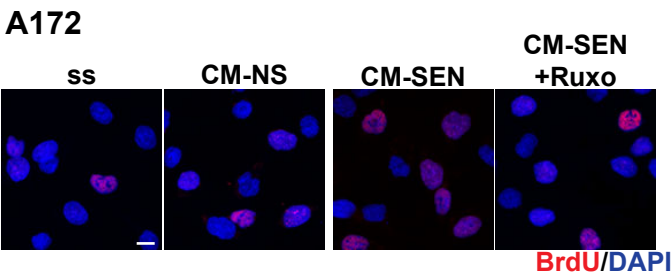

A172

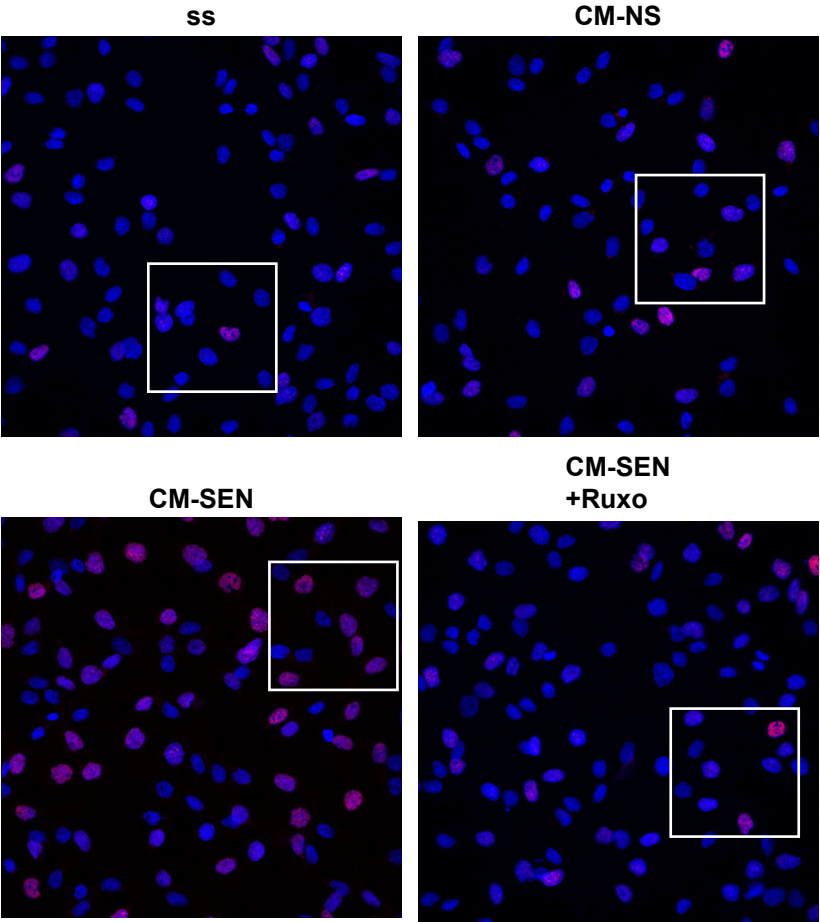

Figure 2

c

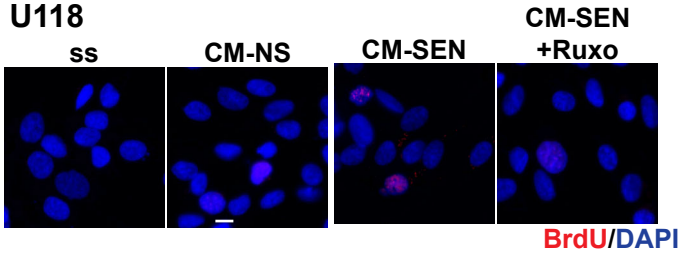

U118

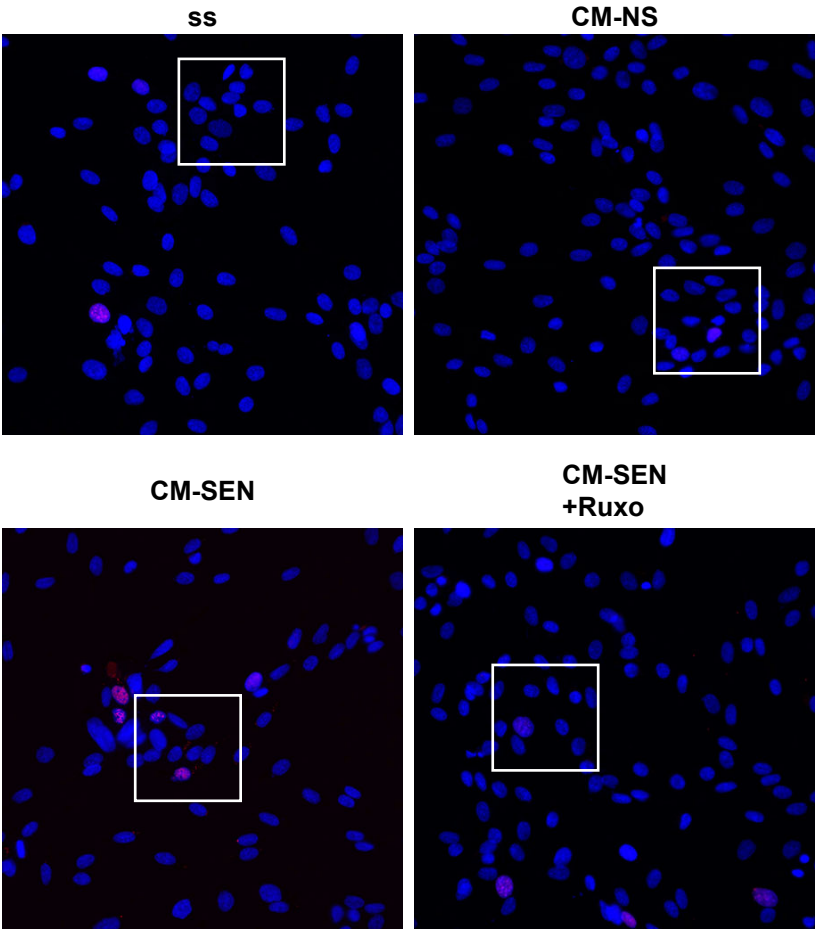

Figure 2

C

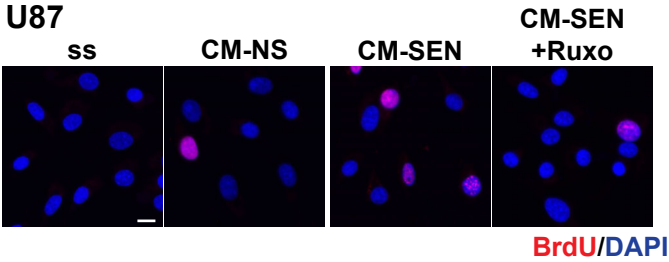

U87

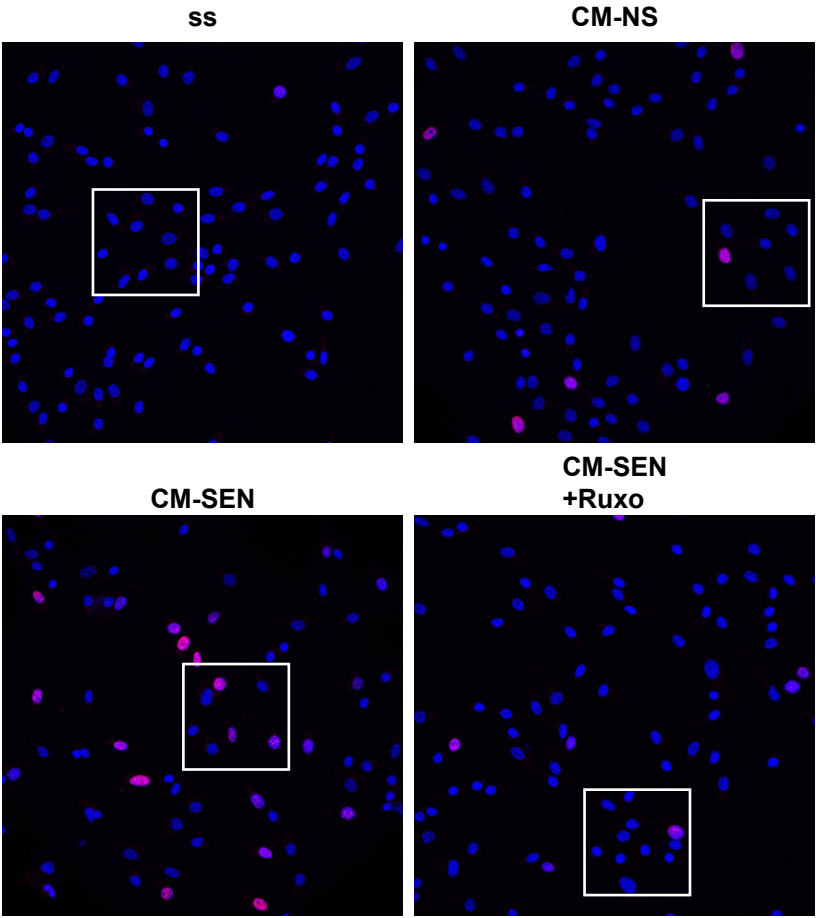

Supplement: Supplementary file 4 — Source data Fig. 2 [file 44321_2025_201_MOESM4_ESM.zip › Fig2/Fig2c BrdU IF/Fig2c.pdf]

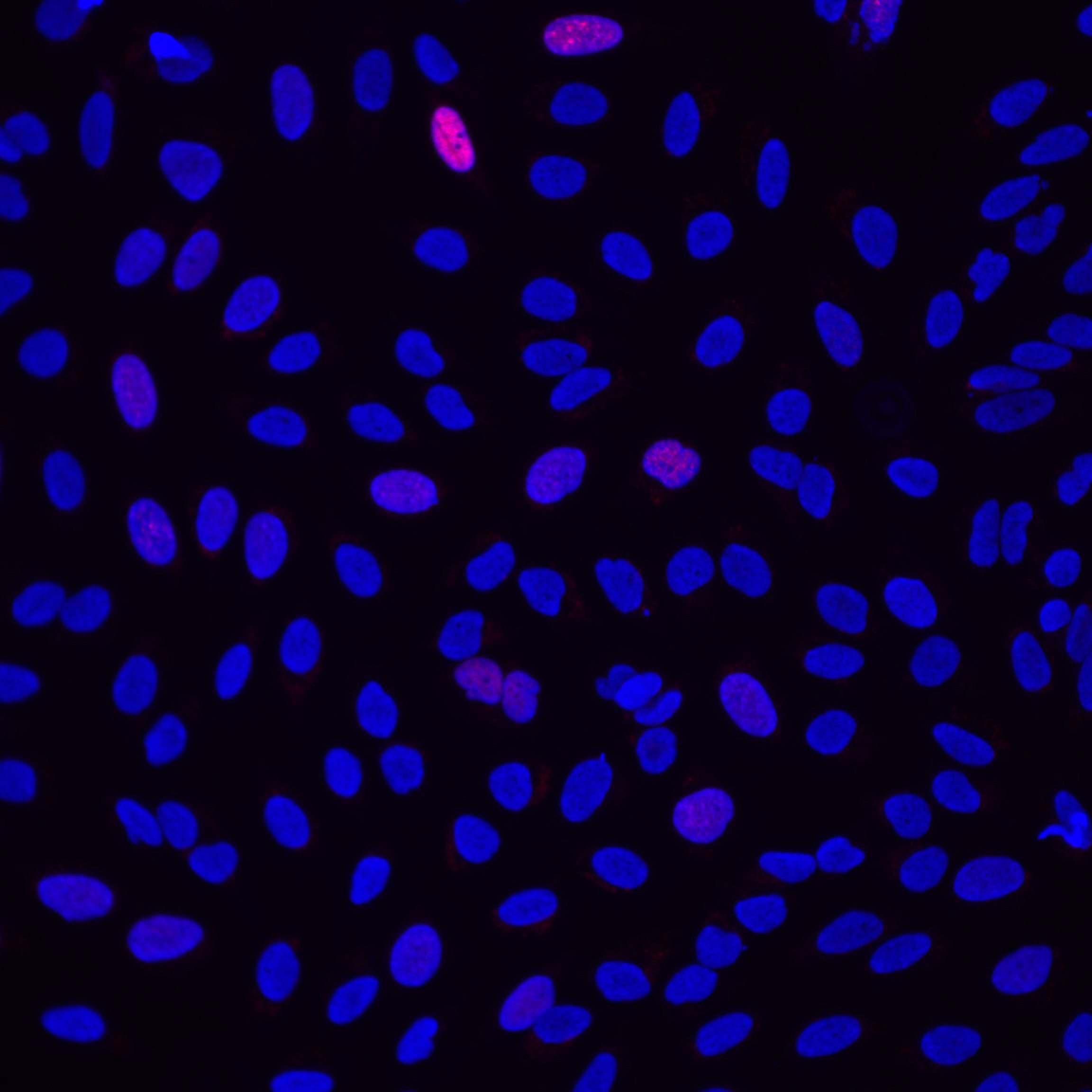

Supplement: Supplementary file 4 — Source data Fig. 2 [file 44321_2025_201_MOESM4_ESM.zip › Fig2/Fig2c BrdU IF/LN229/CM-NS.jpg]

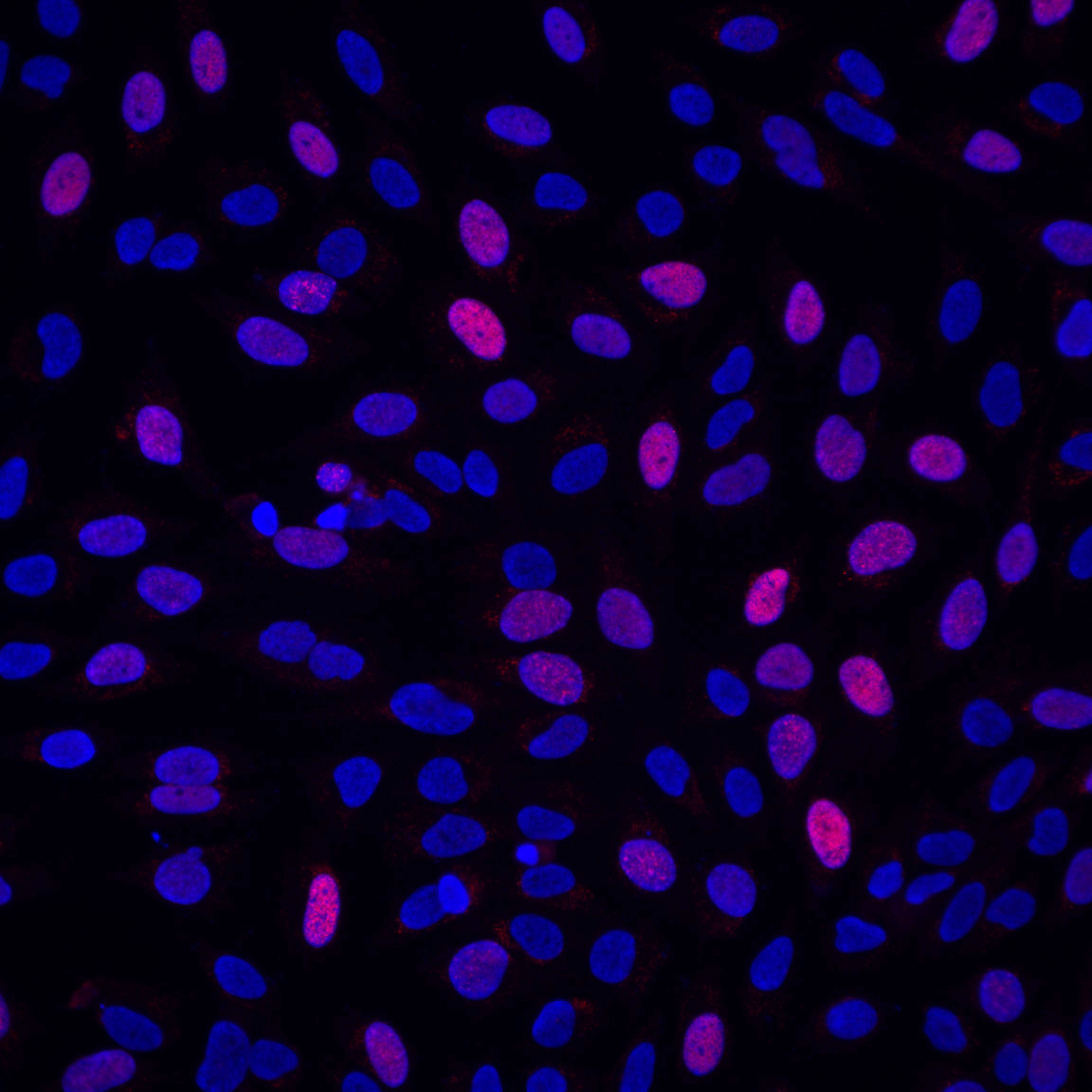

Supplement: Supplementary file 4 — Source data Fig. 2 [file 44321_2025_201_MOESM4_ESM.zip › Fig2/Fig2c BrdU IF/LN229/CM-SEN-1.jpg]

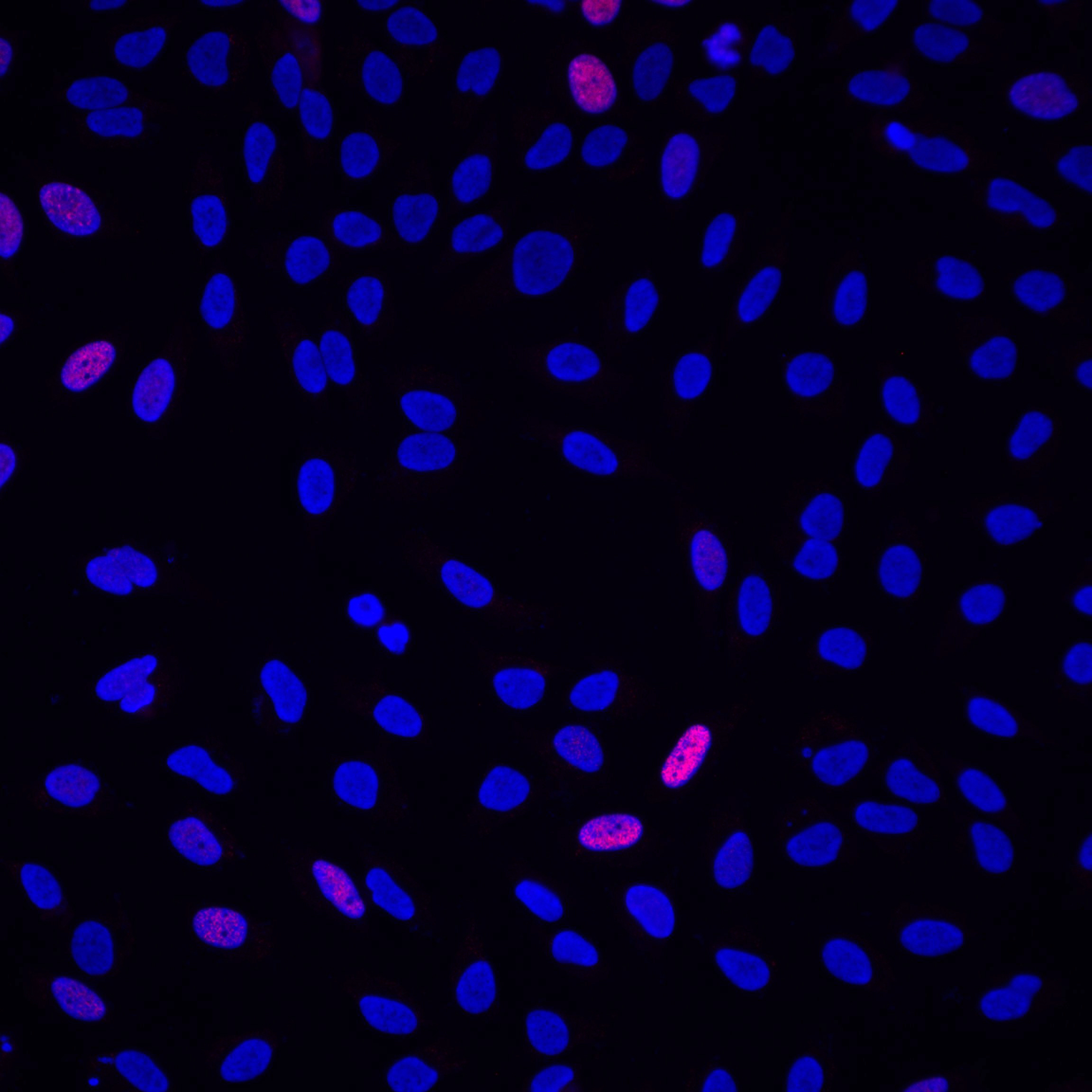

Supplement: Supplementary file 4 — Source data Fig. 2 [file 44321_2025_201_MOESM4_ESM.zip › Fig2/Fig2c BrdU IF/LN229/RUXO.jpg]

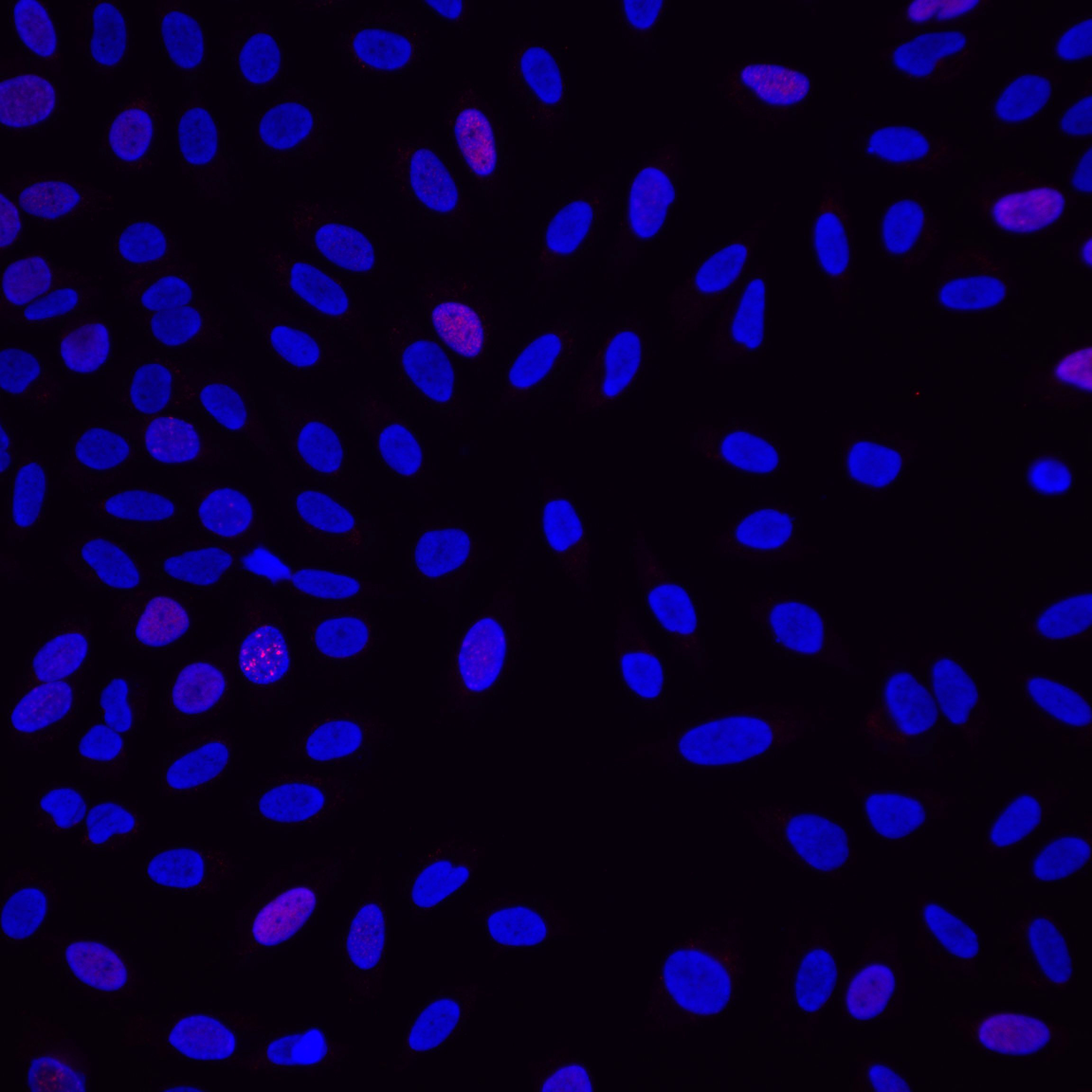

Supplement: Supplementary file 4 — Source data Fig. 2 [file 44321_2025_201_MOESM4_ESM.zip › Fig2/Fig2c BrdU IF/LN229/ss.jpg]

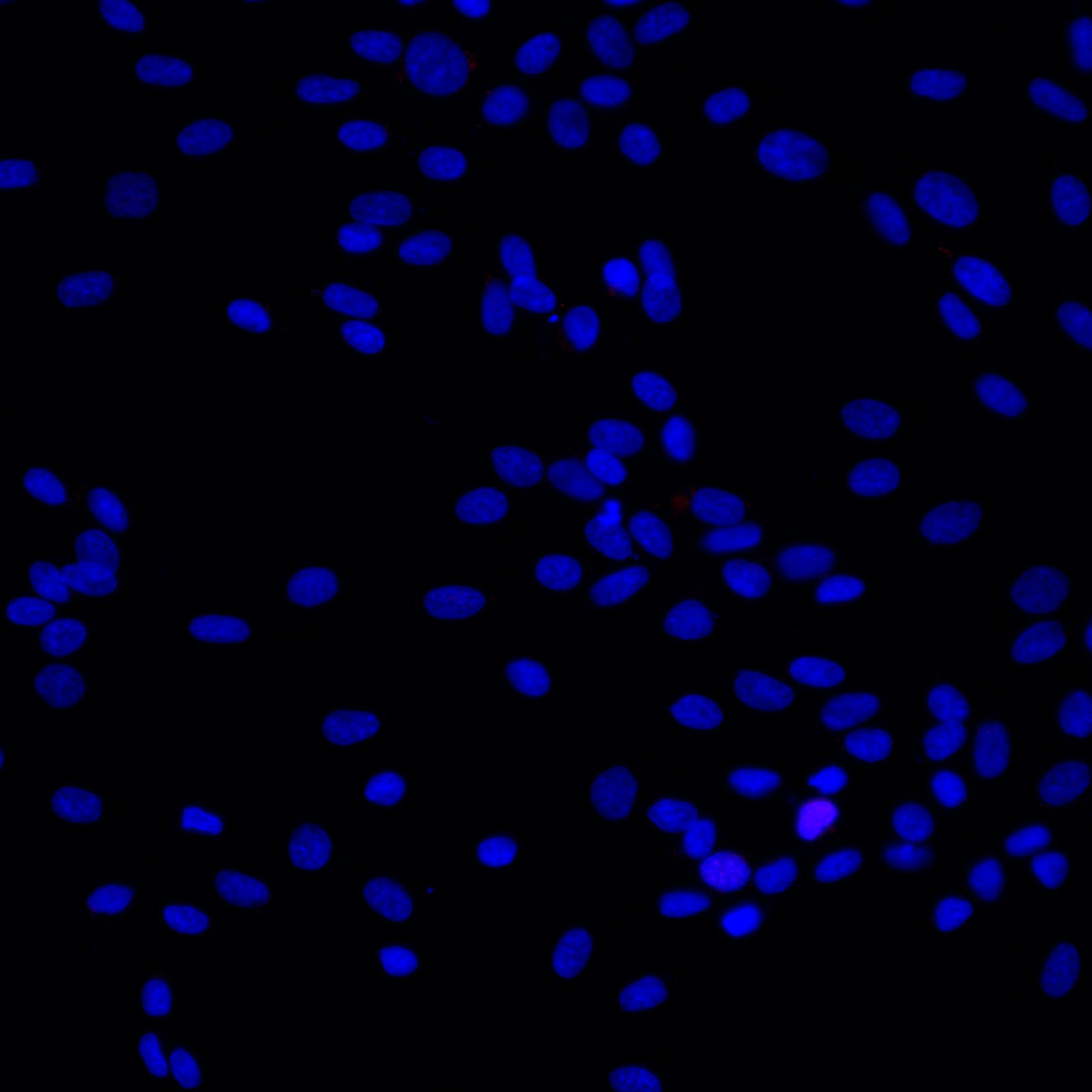

Supplement: Supplementary file 4 — Source data Fig. 2 [file 44321_2025_201_MOESM4_ESM.zip › Fig2/Fig2c BrdU IF/U118/m-118-3-CM-NS-005.nd2 - C=1.jpg]

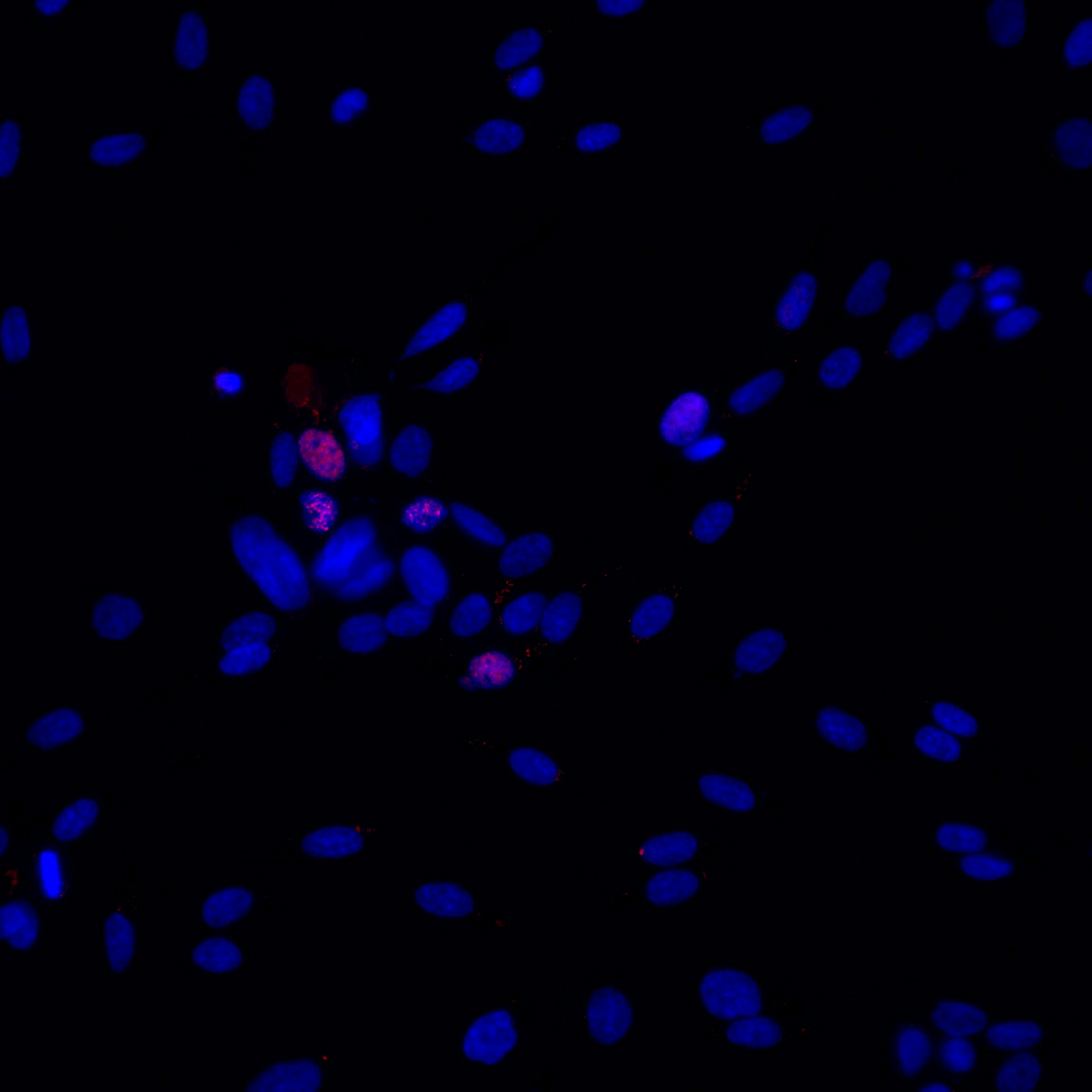

Supplement: Supplementary file 4 — Source data Fig. 2 [file 44321_2025_201_MOESM4_ESM.zip › Fig2/Fig2c BrdU IF/U118/m-118-3-CM-SEN-.nd2 - C=0.jpg]

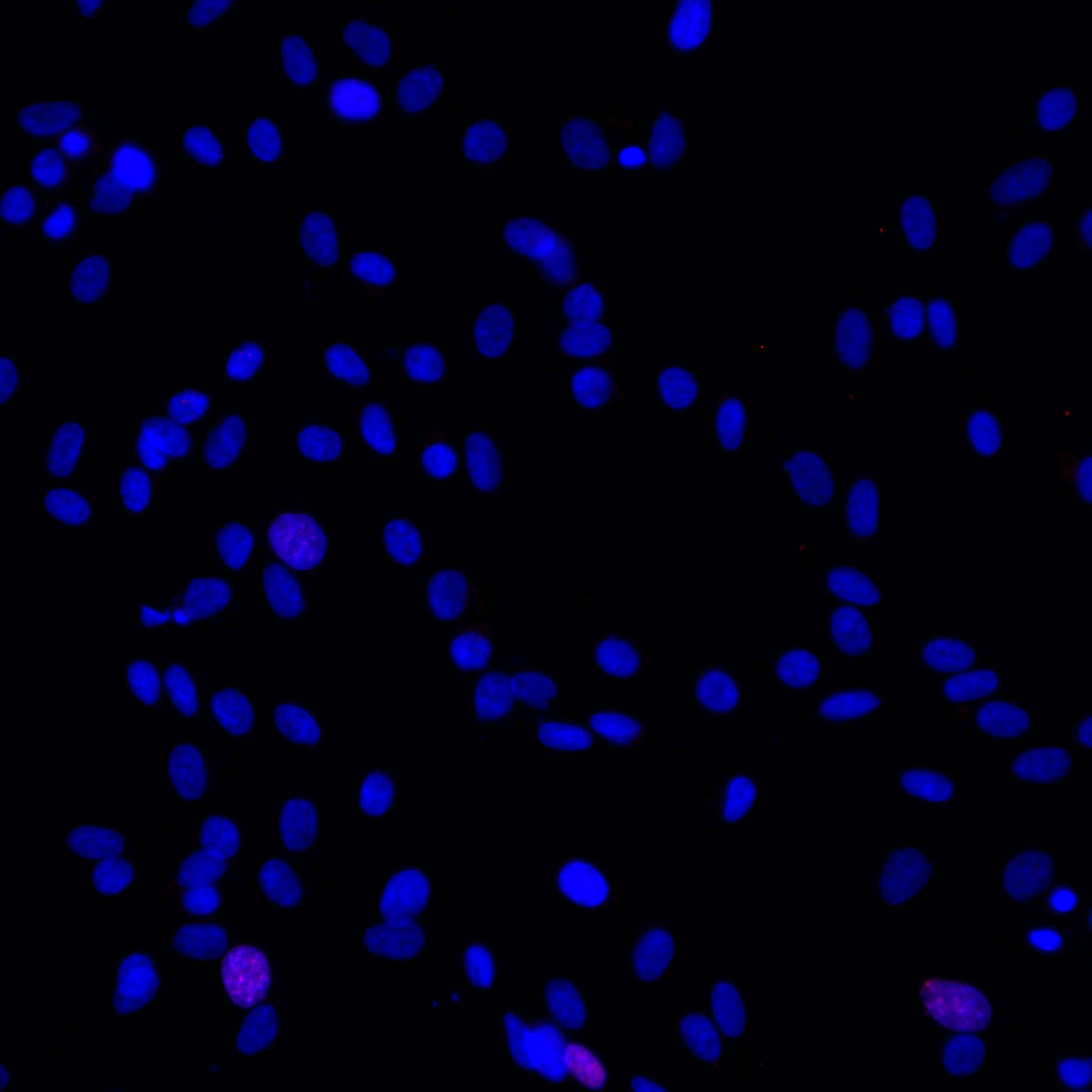

Supplement: Supplementary file 4 — Source data Fig. 2 [file 44321_2025_201_MOESM4_ESM.zip › Fig2/Fig2c BrdU IF/U118/m-118-3-CM-SEN-RUXO-.nd2 - C=0.jpg]

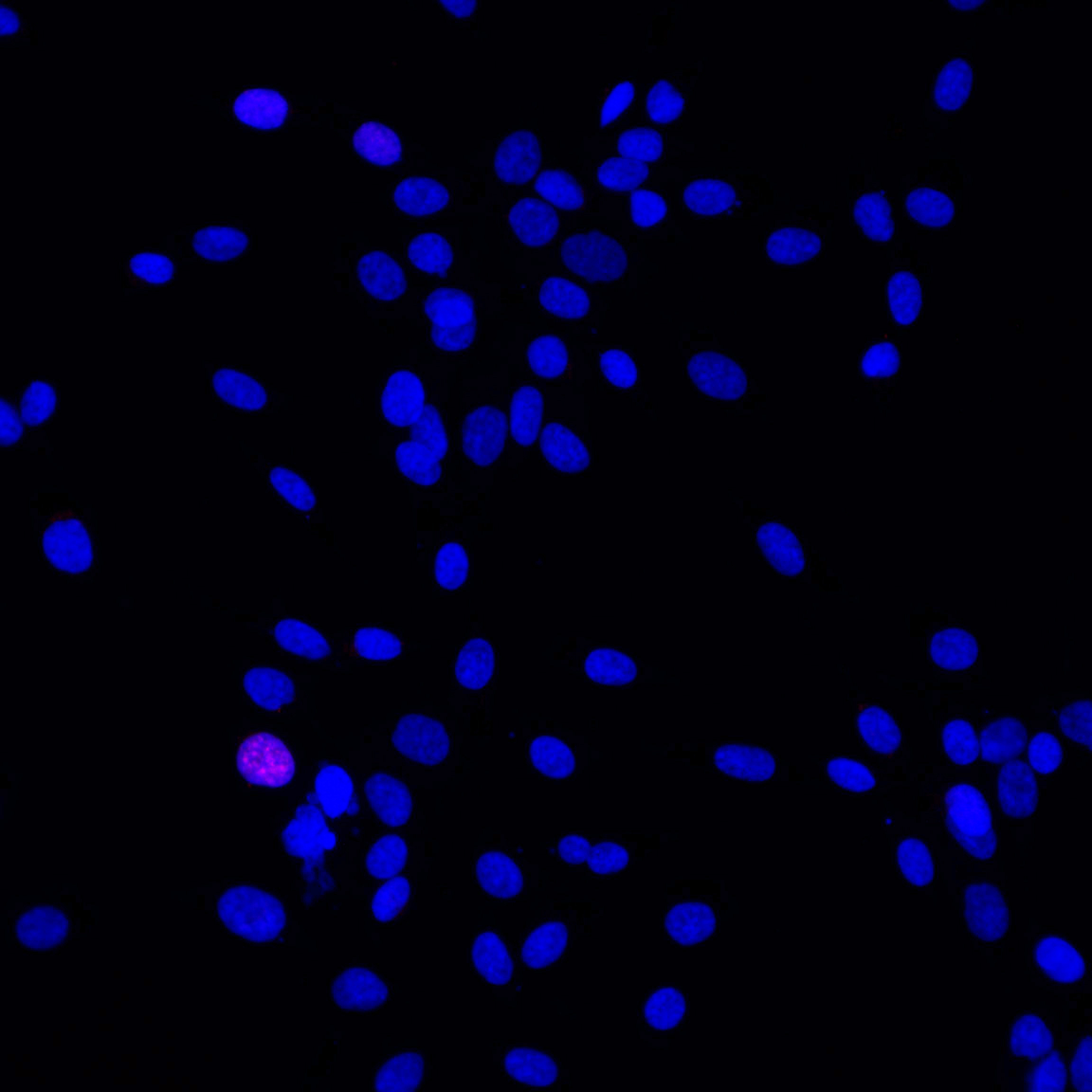

Supplement: Supplementary file 4 — Source data Fig. 2 [file 44321_2025_201_MOESM4_ESM.zip › Fig2/Fig2c BrdU IF/U118/m-118-3-ss-004.jpg]

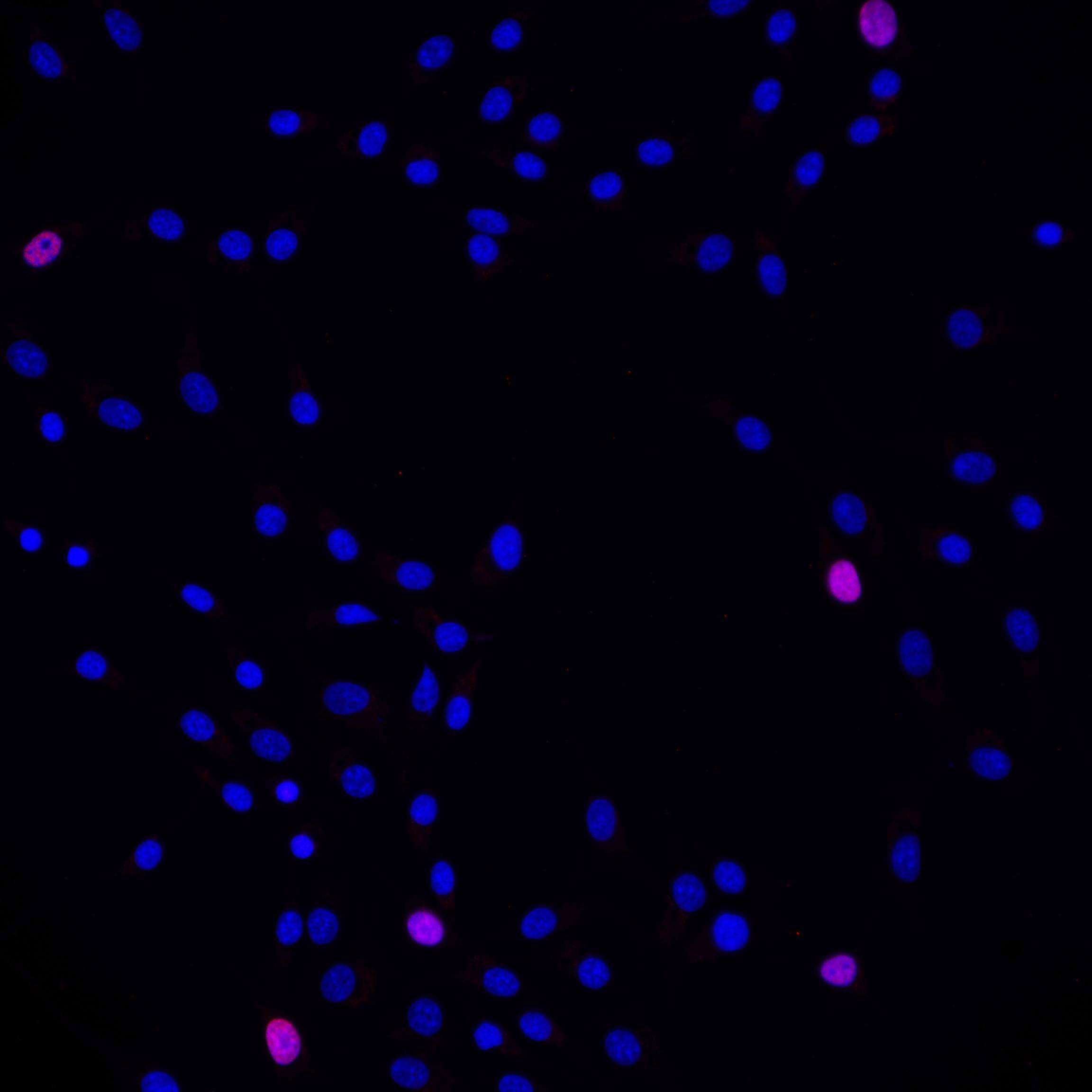

Supplement: Supplementary file 4 — Source data Fig. 2 [file 44321_2025_201_MOESM4_ESM.zip › Fig2/Fig2c BrdU IF/U87/m-U87-2-CM-NS-005.jpg]

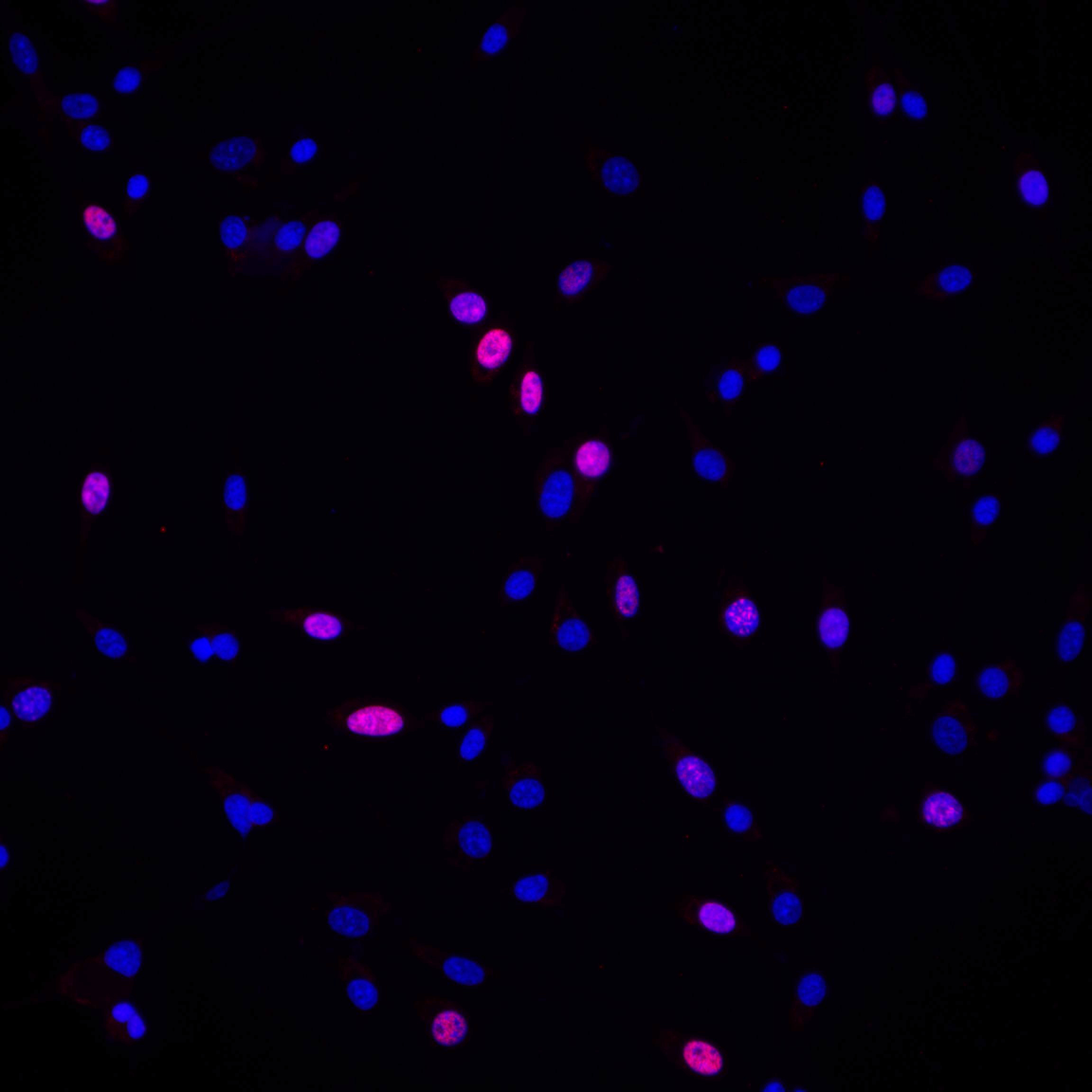

Supplement: Supplementary file 4 — Source data Fig. 2 [file 44321_2025_201_MOESM4_ESM.zip › Fig2/Fig2c BrdU IF/U87/m-U87-2-CM-SEN-005.jpg]

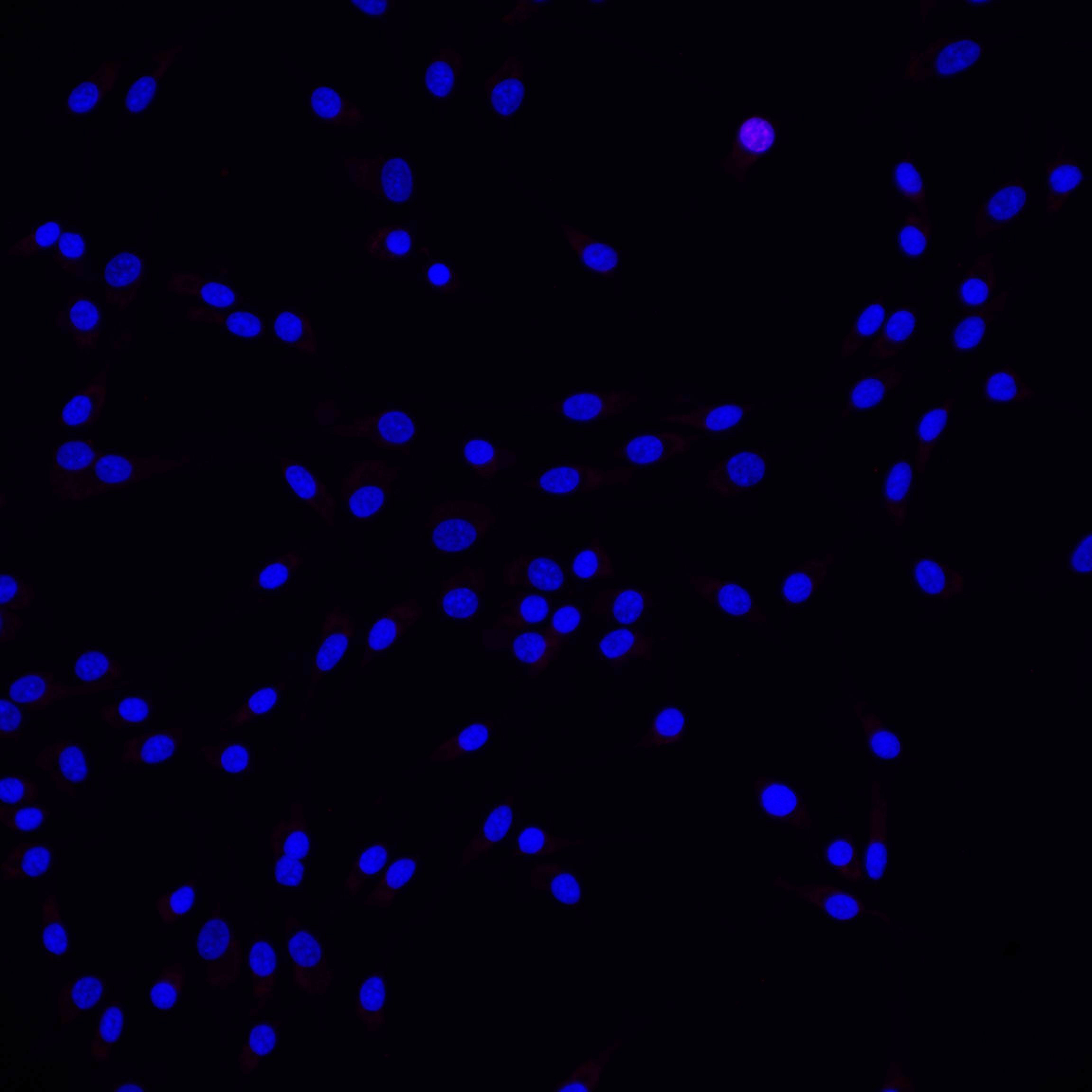

Supplement: Supplementary file 4 — Source data Fig. 2 [file 44321_2025_201_MOESM4_ESM.zip › Fig2/Fig2c BrdU IF/U87/m-U87-2-ss-001.jpg]

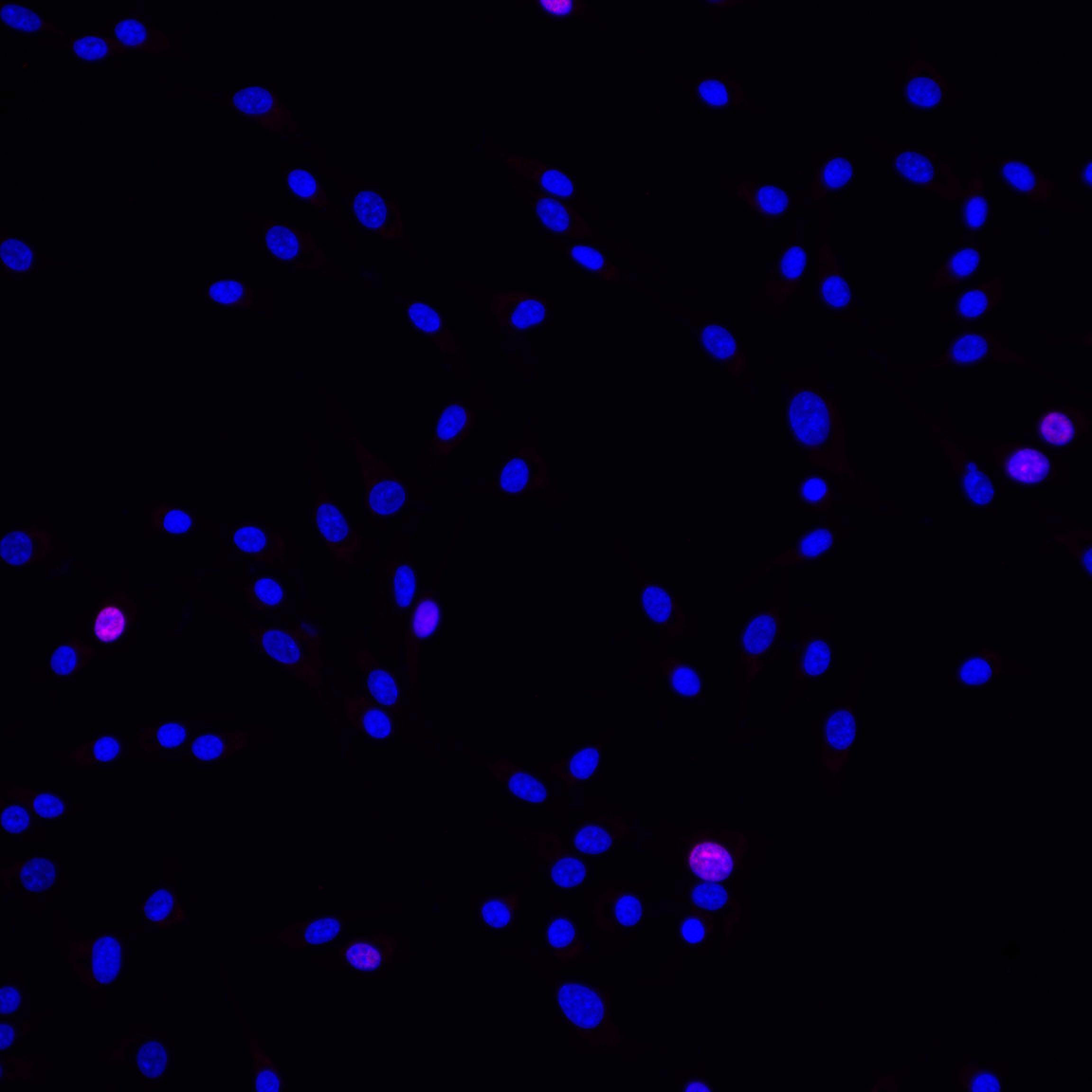

Supplement: Supplementary file 4 — Source data Fig. 2 [file 44321_2025_201_MOESM4_ESM.zip › Fig2/Fig2c BrdU IF/U87/U87-Ruxo.jpg]

Fig3

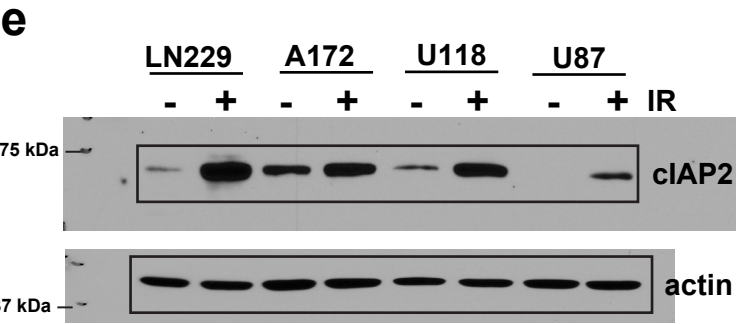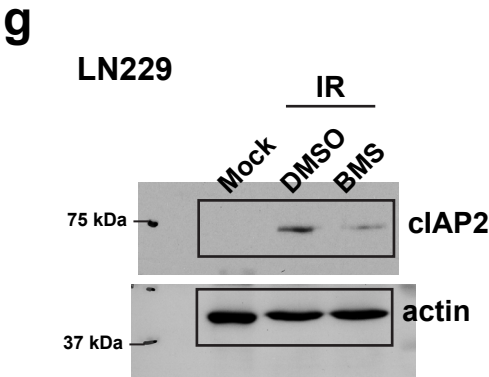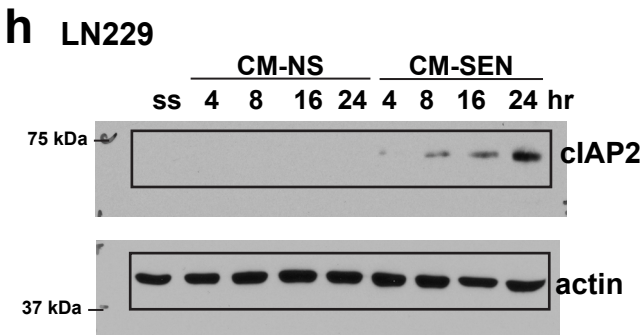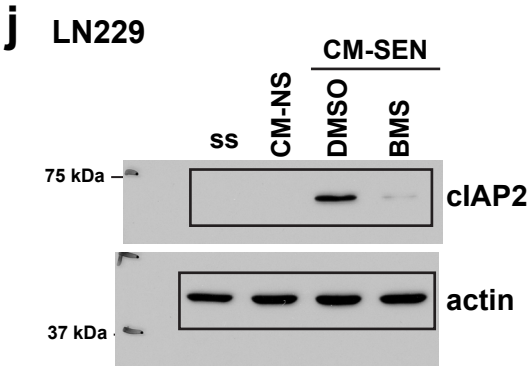

Supplement: Supplementary file 5 — Source data Fig. 3 [file 44321_2025_201_MOESM5_ESM.zip › Fig3/Fig3-WBe-g-h-j.pdf]

Figure 4

a

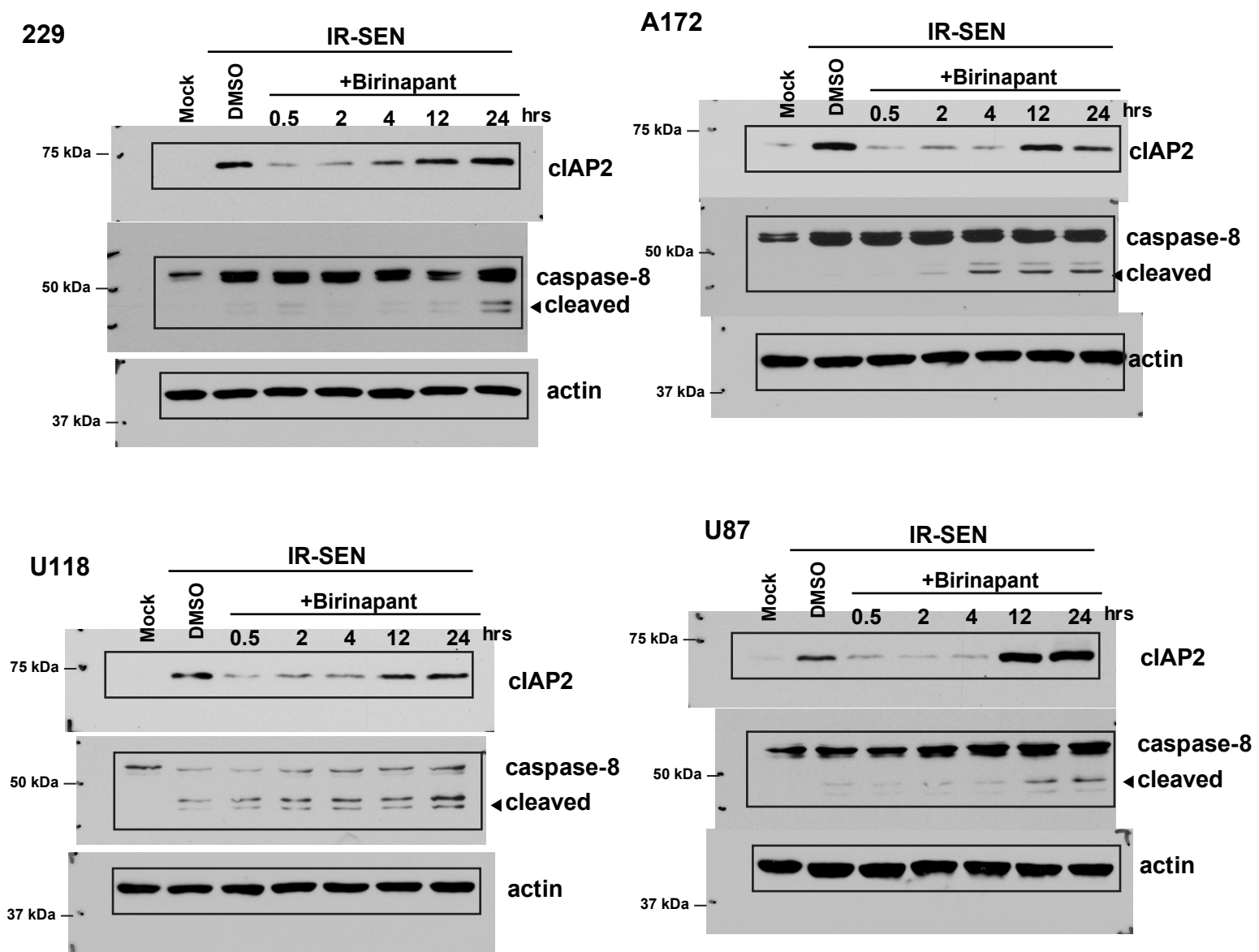

Supplement: Supplementary file 6 — Source data Fig. 4 [file 44321_2025_201_MOESM6_ESM.zip › Fig4/Fig4a.pdf]

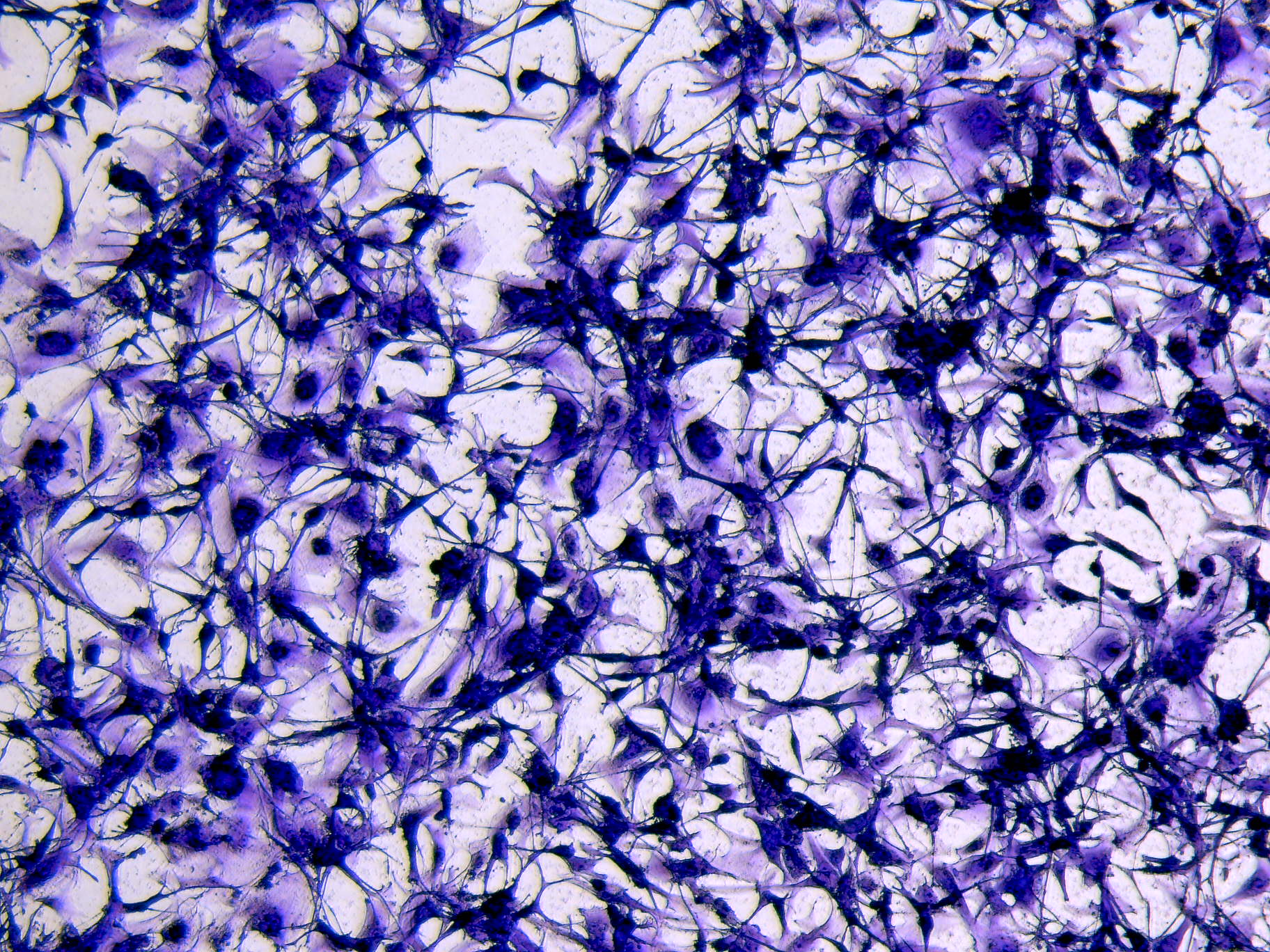

Supplement: Supplementary file 6 — Source data Fig. 4 [file 44321_2025_201_MOESM6_ESM.zip › Fig4/Fig4b CV/A172/IR/Biri-D0.JPG]

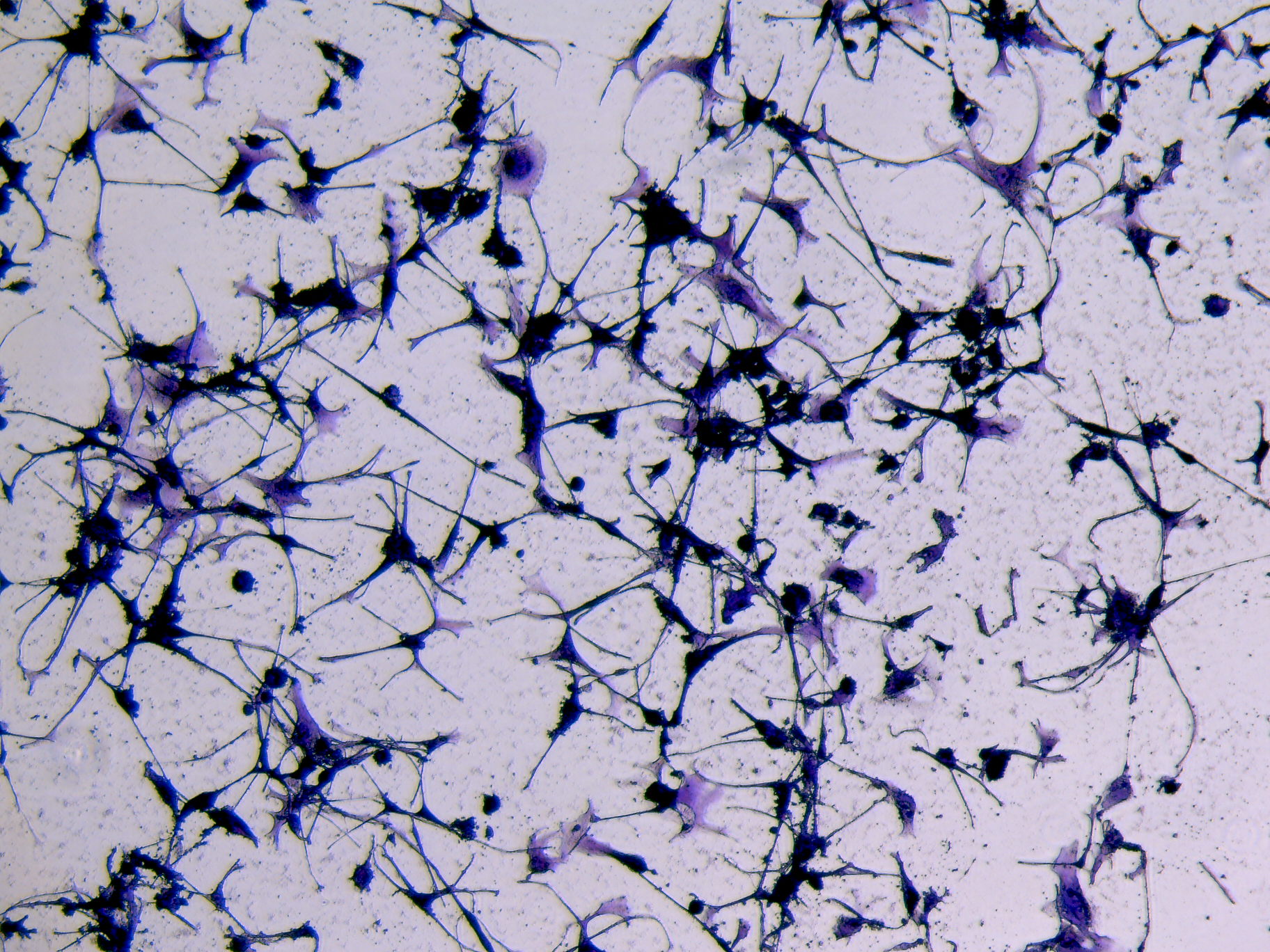

Supplement: Supplementary file 6 — Source data Fig. 4 [file 44321_2025_201_MOESM6_ESM.zip › Fig4/Fig4b CV/A172/IR/Biri-D3.JPG]

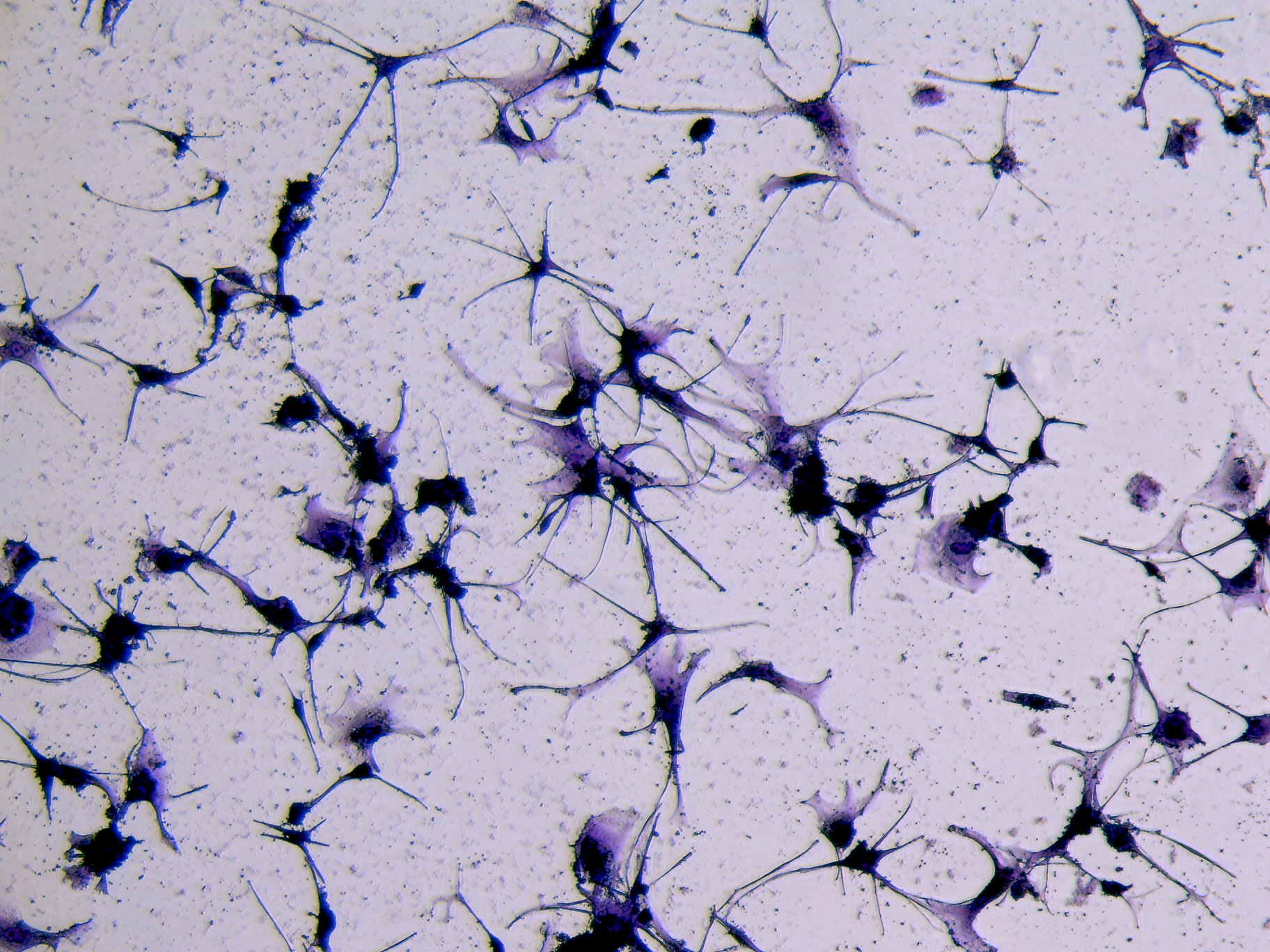

Supplement: Supplementary file 6 — Source data Fig. 4 [file 44321_2025_201_MOESM6_ESM.zip › Fig4/Fig4b CV/A172/IR/Biri-D6.JPG]

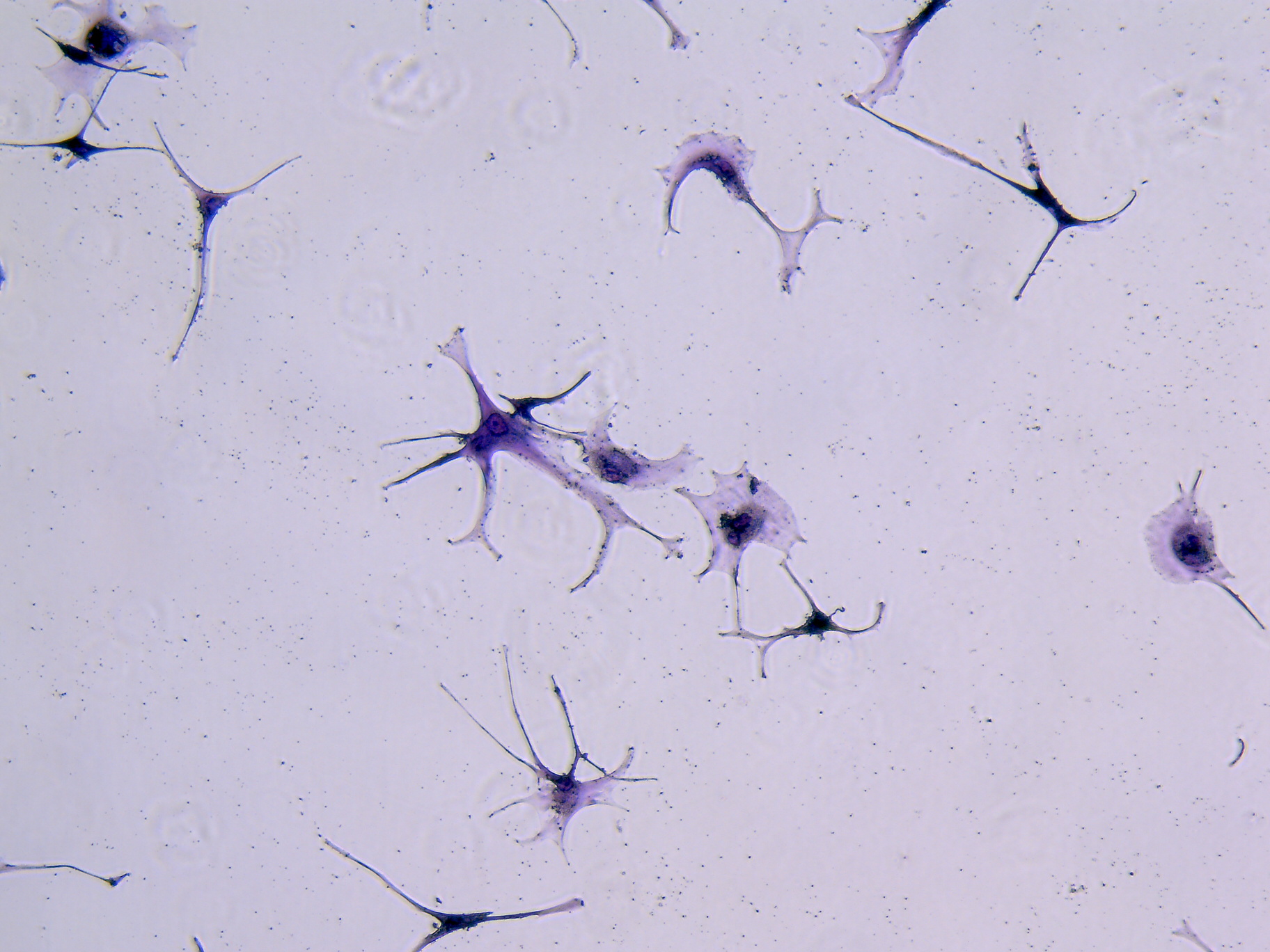

Supplement: Supplementary file 6 — Source data Fig. 4 [file 44321_2025_201_MOESM6_ESM.zip › Fig4/Fig4b CV/A172/IR/Biri-D9.JPG]

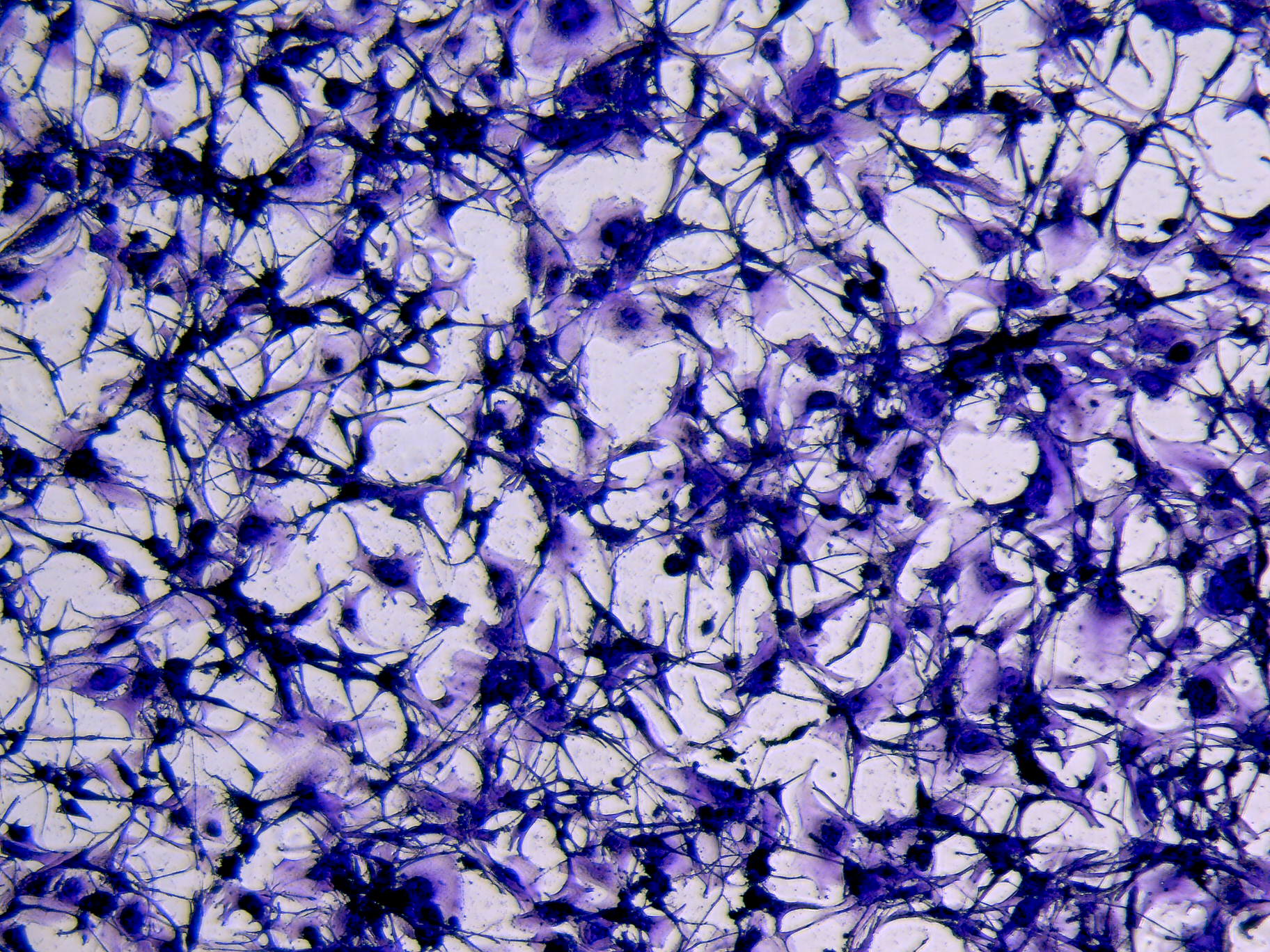

Supplement: Supplementary file 6 — Source data Fig. 4 [file 44321_2025_201_MOESM6_ESM.zip › Fig4/Fig4b CV/A172/IR/DMSO-D0.JPG]

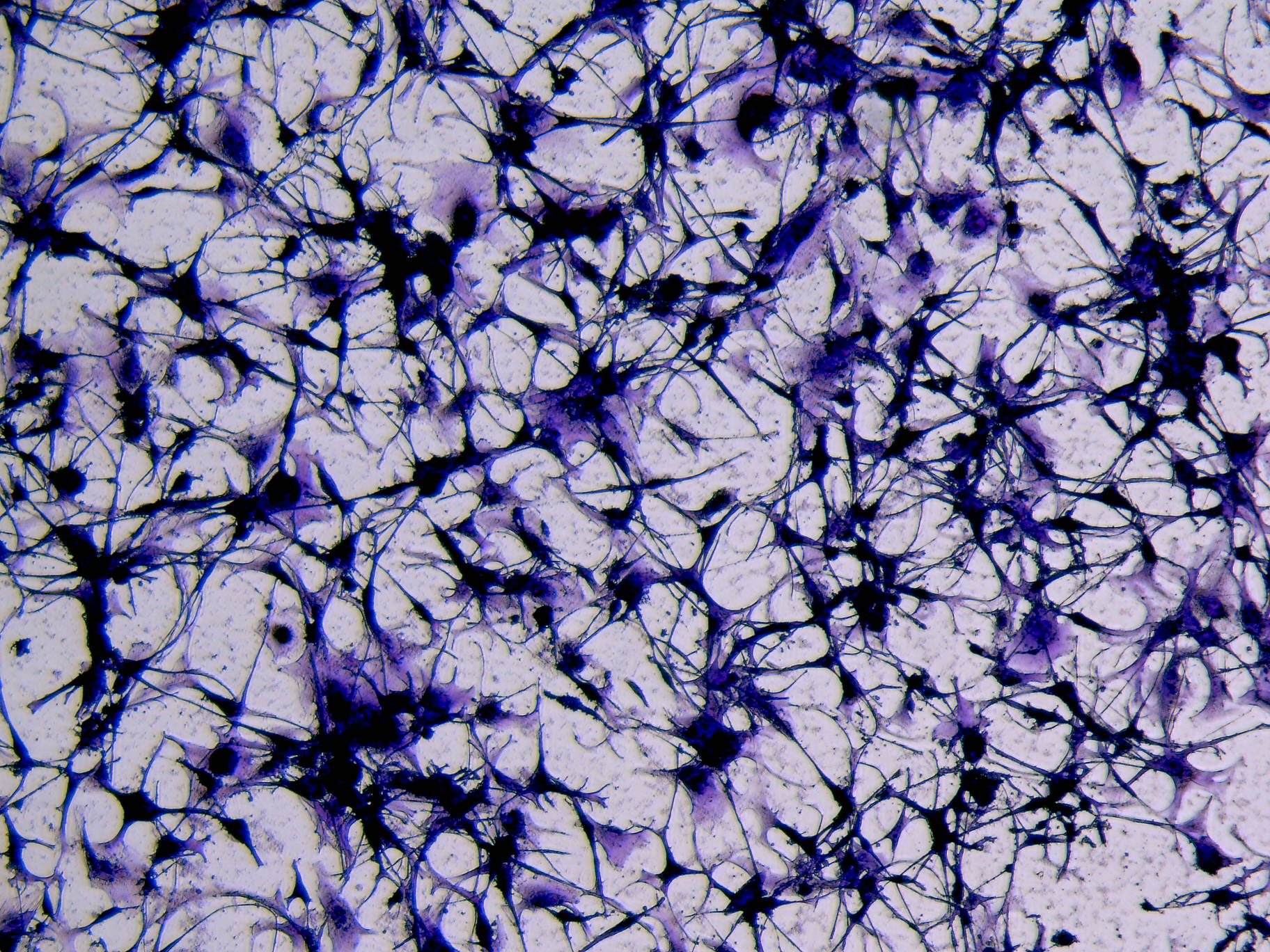

Supplement: Supplementary file 6 — Source data Fig. 4 [file 44321_2025_201_MOESM6_ESM.zip › Fig4/Fig4b CV/A172/IR/DMSO-D3.JPG]

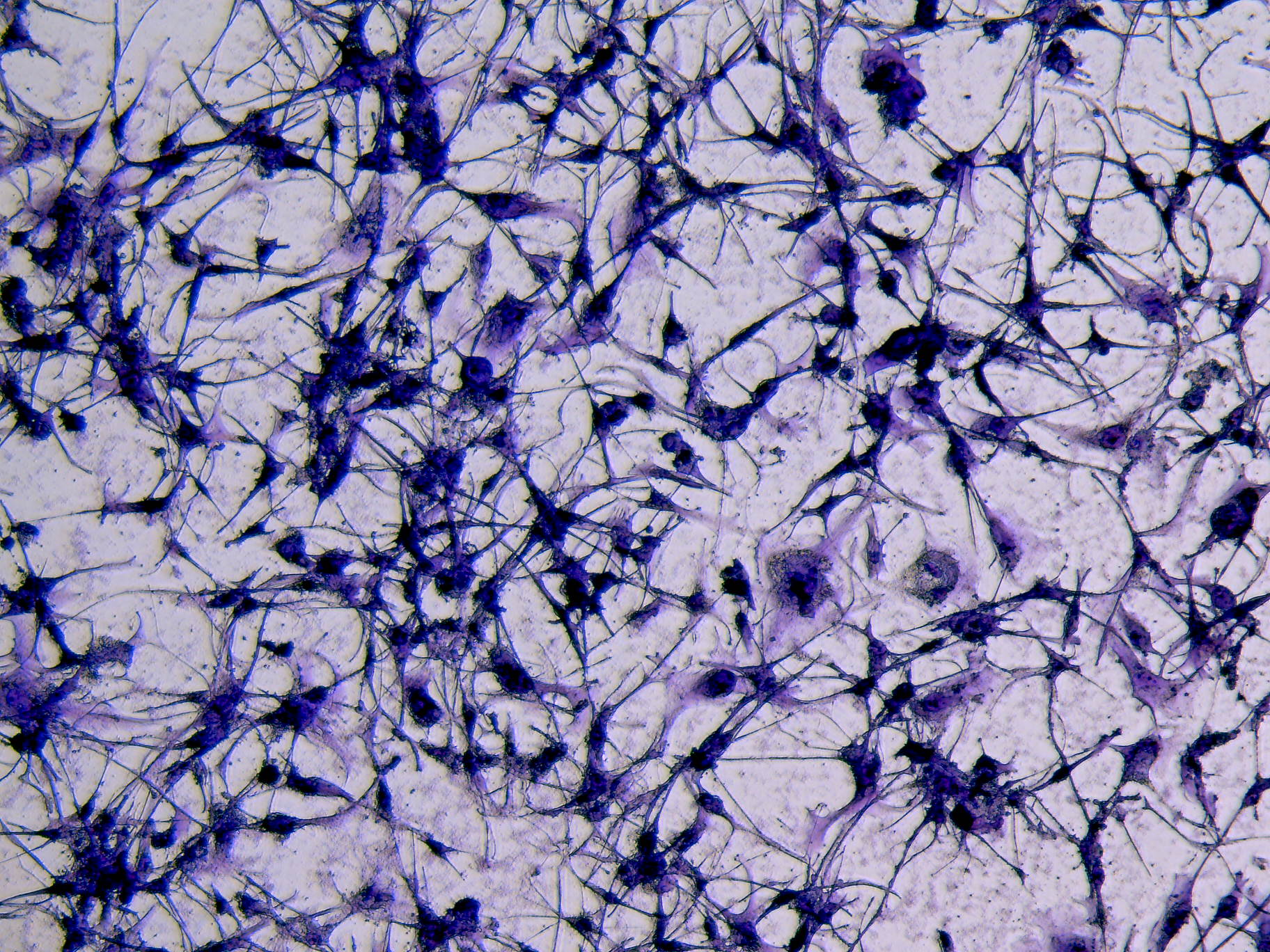

Supplement: Supplementary file 6 — Source data Fig. 4 [file 44321_2025_201_MOESM6_ESM.zip › Fig4/Fig4b CV/A172/IR/DMSO-D6.JPG]

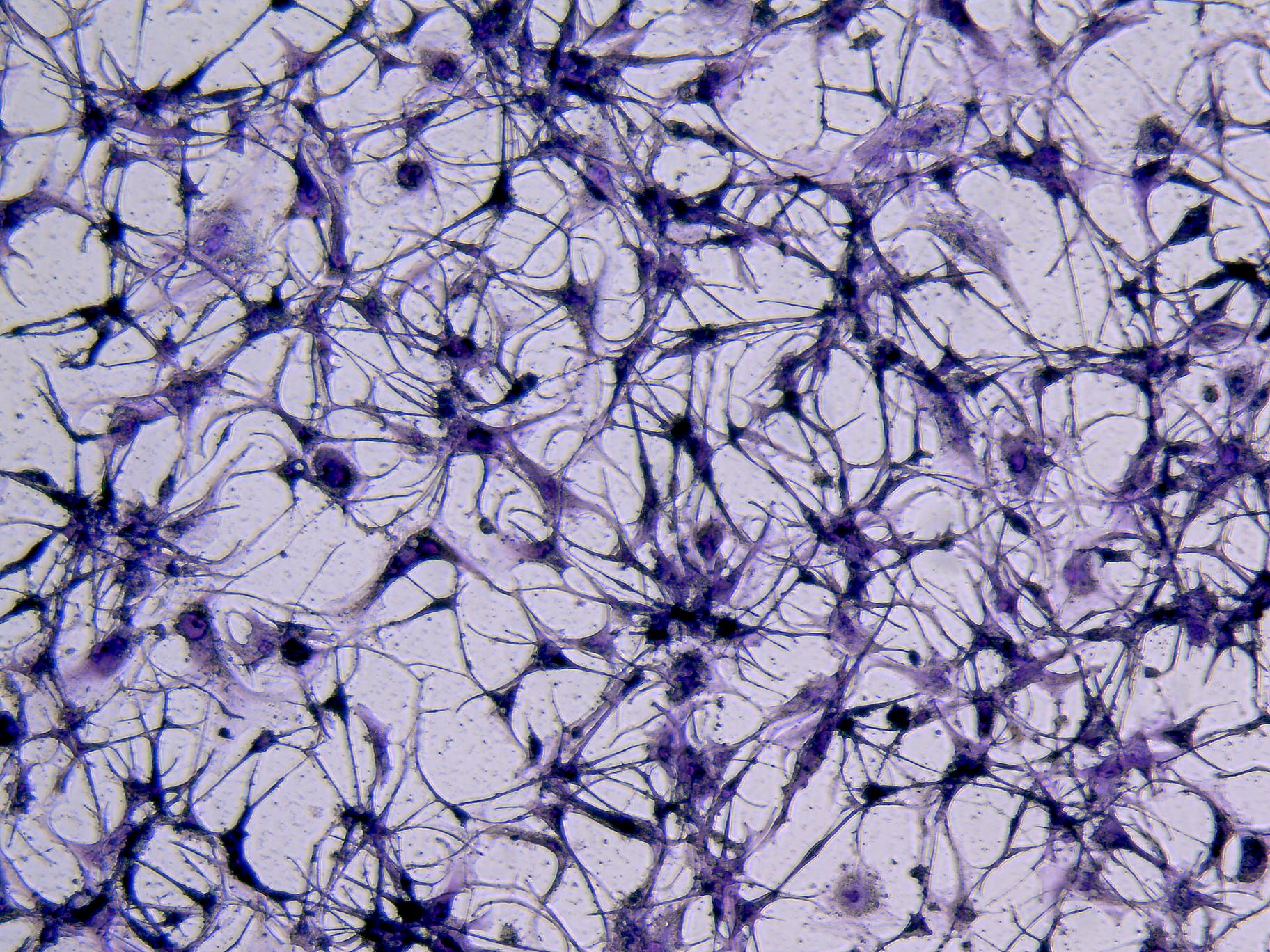

Supplement: Supplementary file 6 — Source data Fig. 4 [file 44321_2025_201_MOESM6_ESM.zip › Fig4/Fig4b CV/A172/IR/DMSO-D9.JPG]

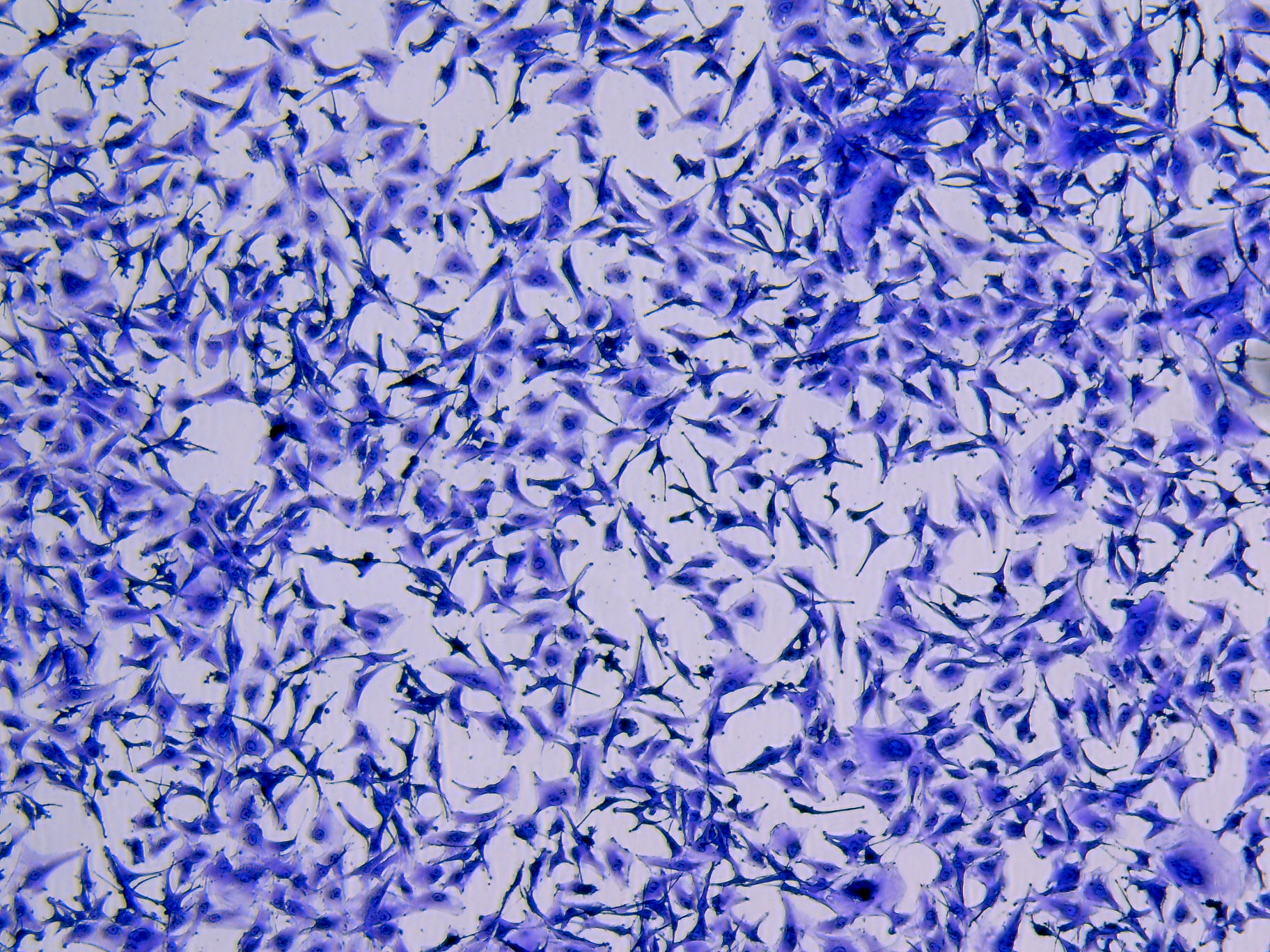

Supplement: Supplementary file 6 — Source data Fig. 4 [file 44321_2025_201_MOESM6_ESM.zip › Fig4/Fig4b CV/A172/mock/Biri-D0.JPG]

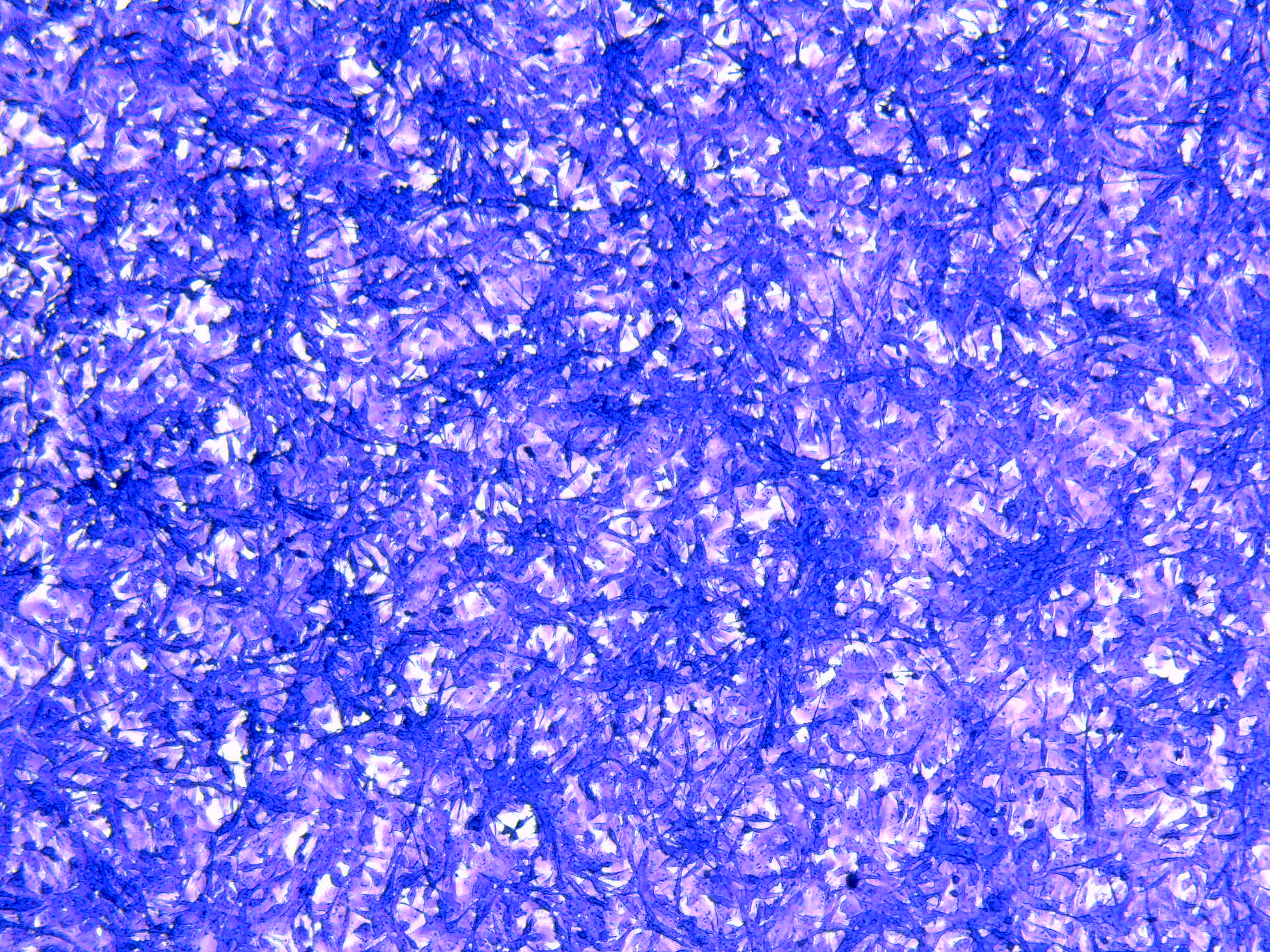

Supplement: Supplementary file 6 — Source data Fig. 4 [file 44321_2025_201_MOESM6_ESM.zip › Fig4/Fig4b CV/A172/mock/Biri-D3.JPG]

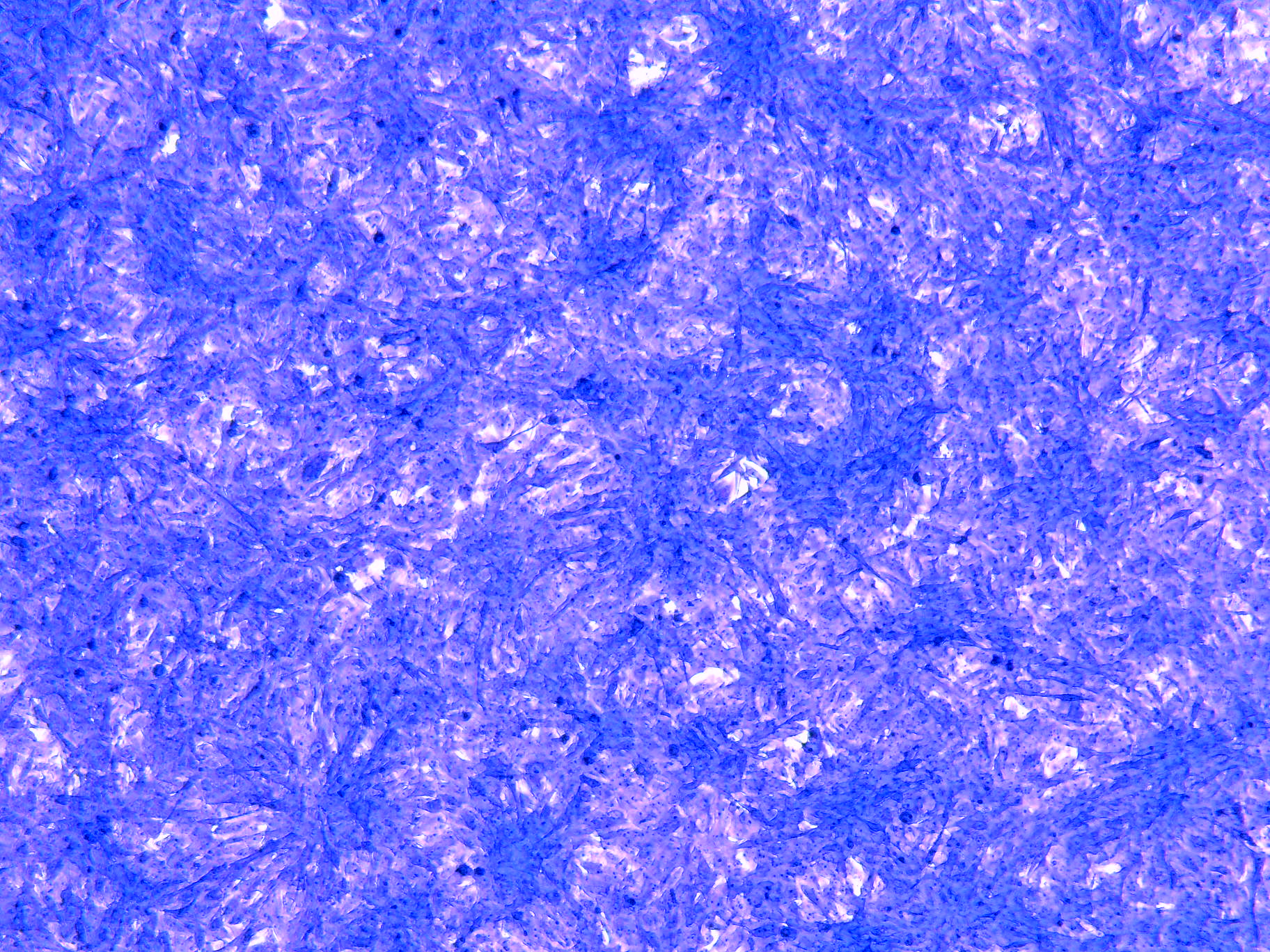

Supplement: Supplementary file 6 — Source data Fig. 4 [file 44321_2025_201_MOESM6_ESM.zip › Fig4/Fig4b CV/A172/mock/Biri-D6.JPG]

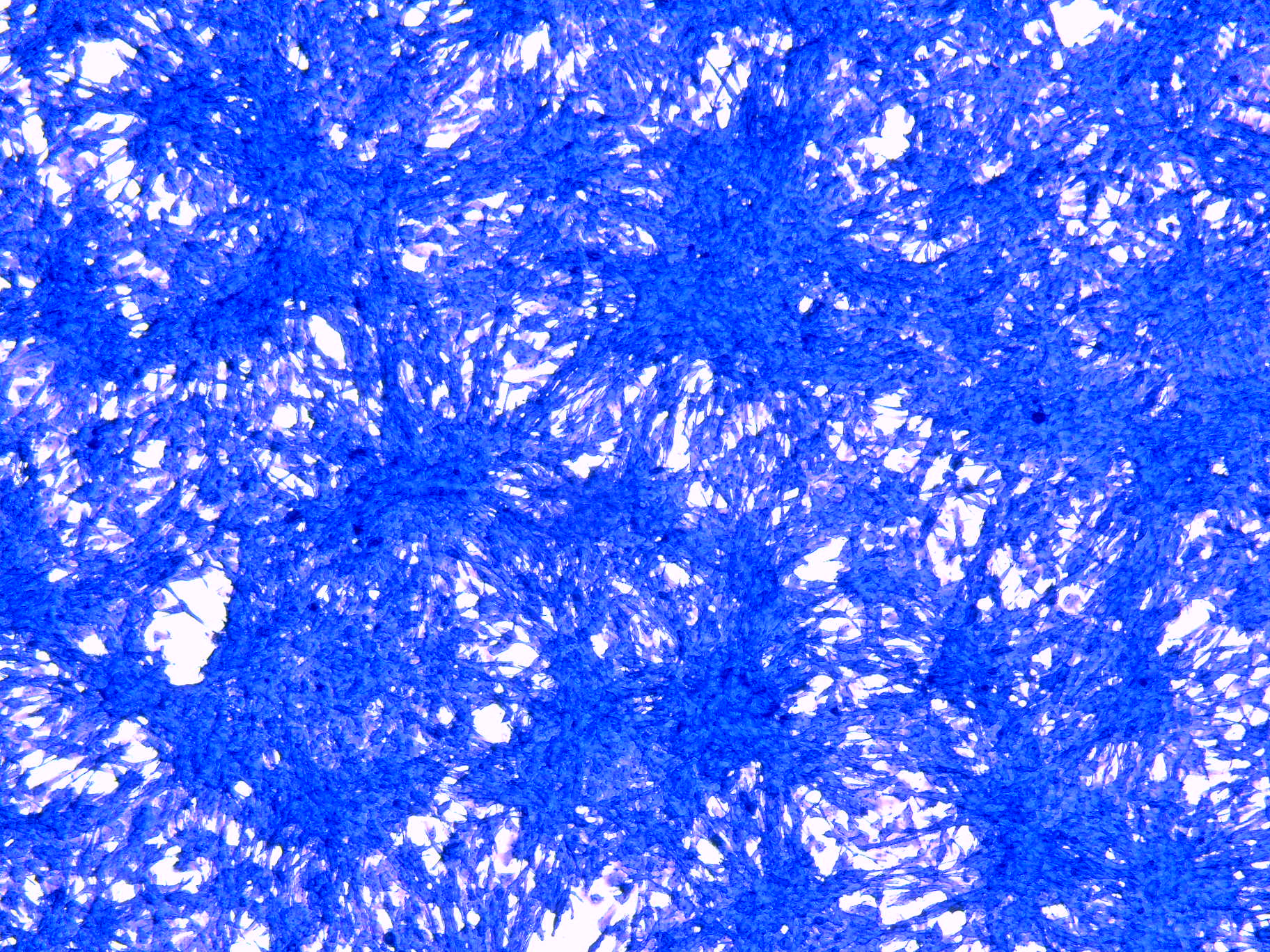

Supplement: Supplementary file 6 — Source data Fig. 4 [file 44321_2025_201_MOESM6_ESM.zip › Fig4/Fig4b CV/A172/mock/Biri-D9.JPG]

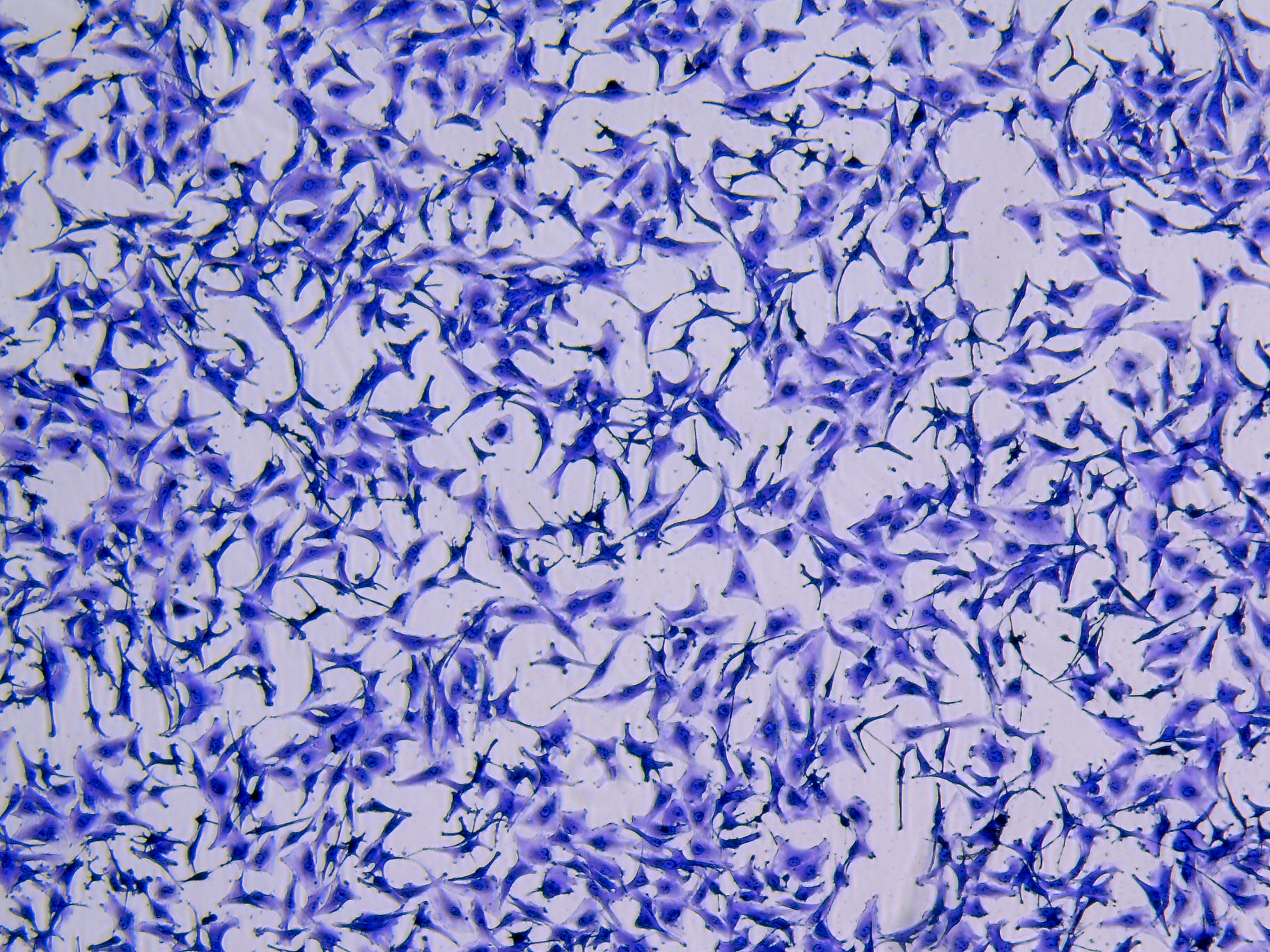

Supplement: Supplementary file 6 — Source data Fig. 4 [file 44321_2025_201_MOESM6_ESM.zip › Fig4/Fig4b CV/A172/mock/DMSO-D0.JPG]

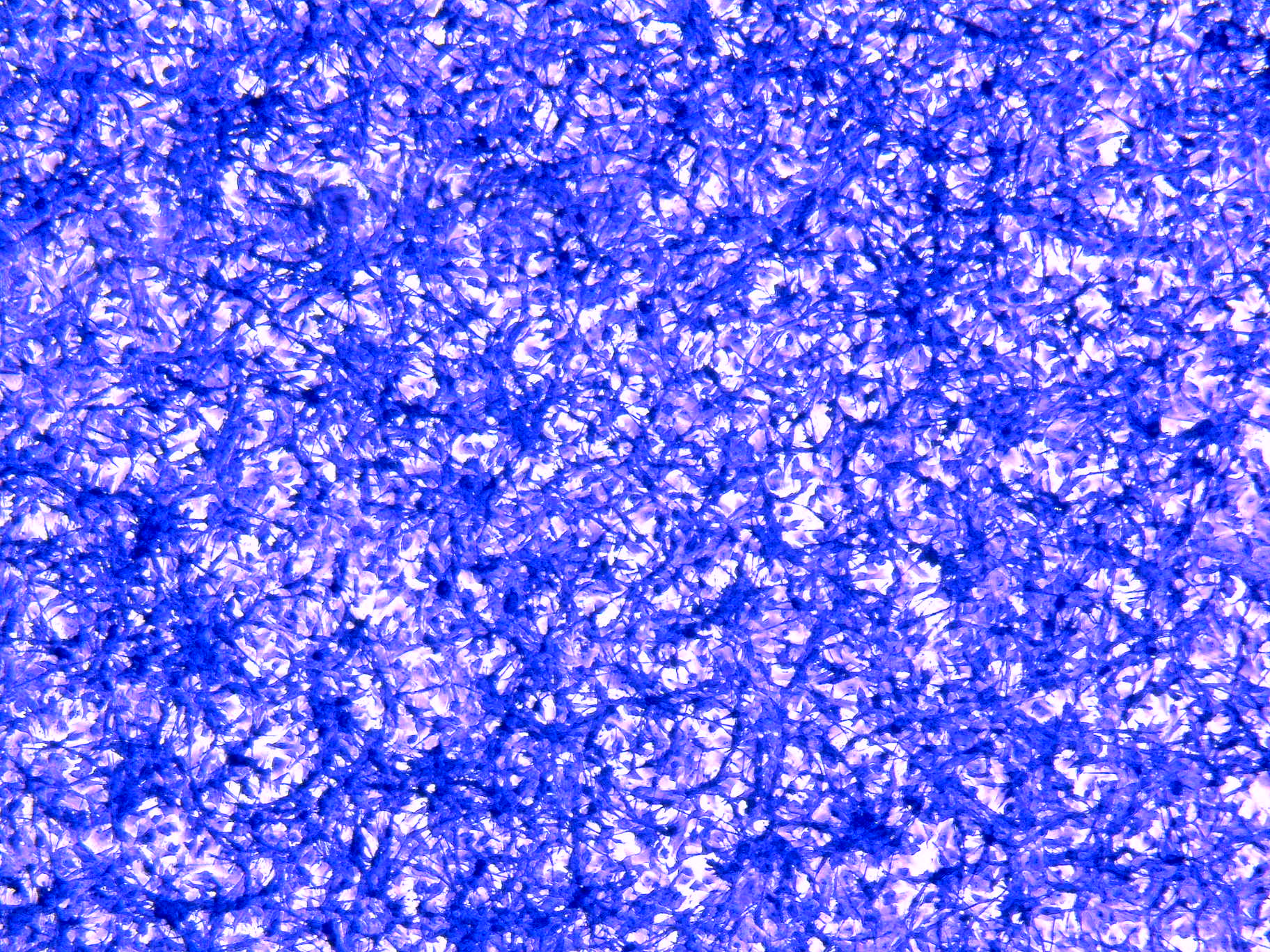

Supplement: Supplementary file 6 — Source data Fig. 4 [file 44321_2025_201_MOESM6_ESM.zip › Fig4/Fig4b CV/A172/mock/DMSO-D3.JPG]

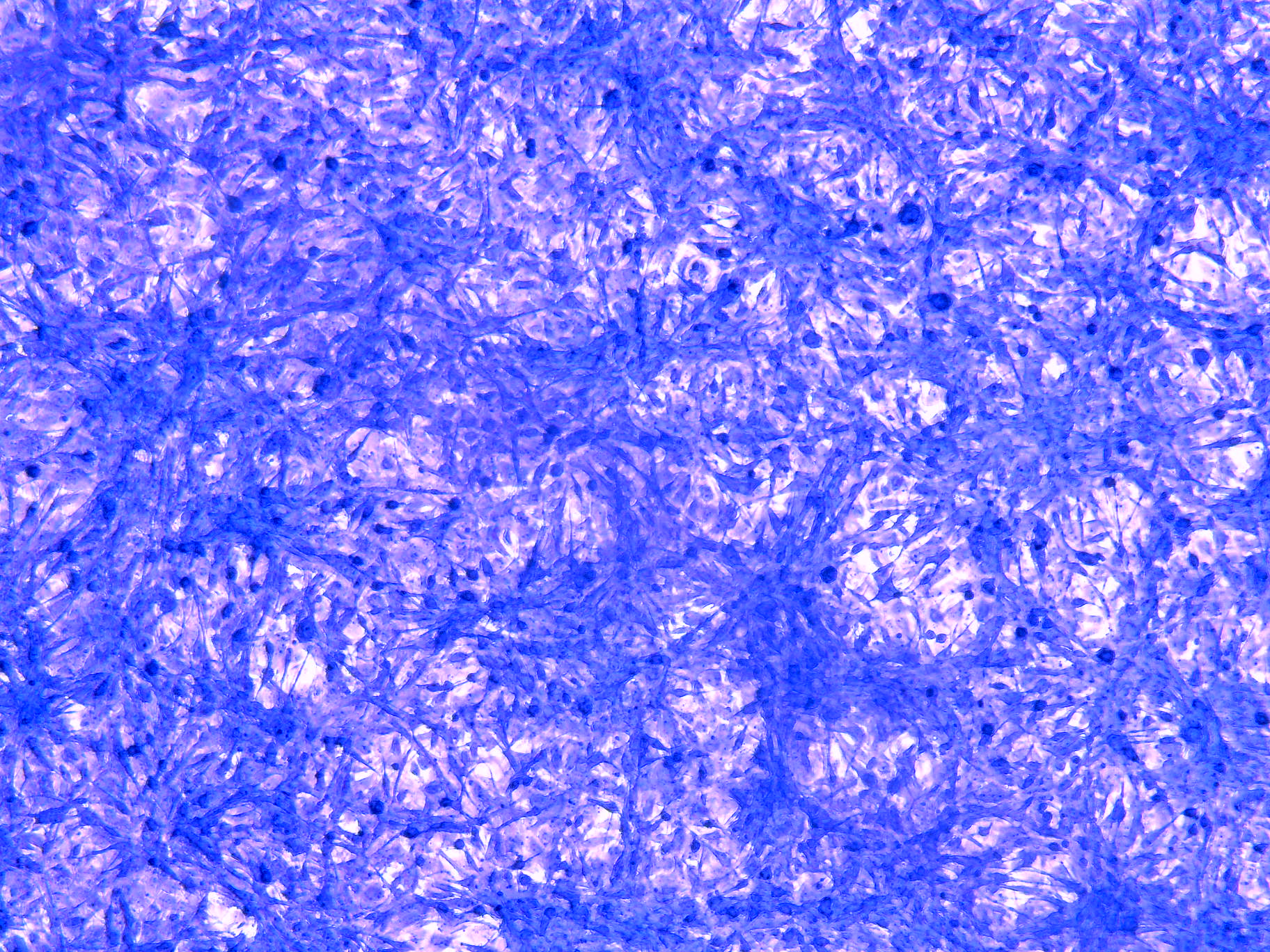

Supplement: Supplementary file 6 — Source data Fig. 4 [file 44321_2025_201_MOESM6_ESM.zip › Fig4/Fig4b CV/A172/mock/DMSO-D6.JPG]

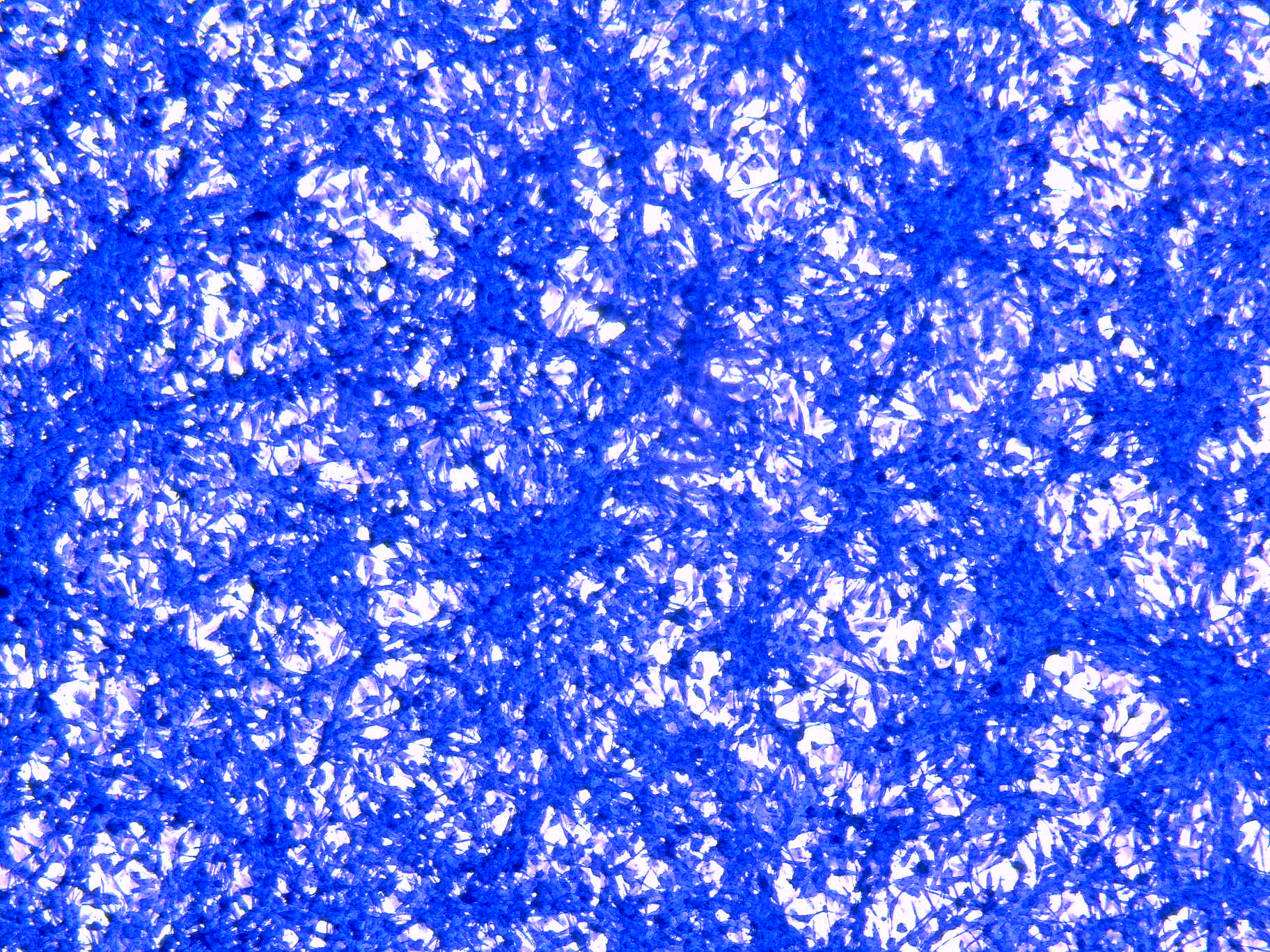

Supplement: Supplementary file 6 — Source data Fig. 4 [file 44321_2025_201_MOESM6_ESM.zip › Fig4/Fig4b CV/A172/mock/DMSO-D9.JPG]

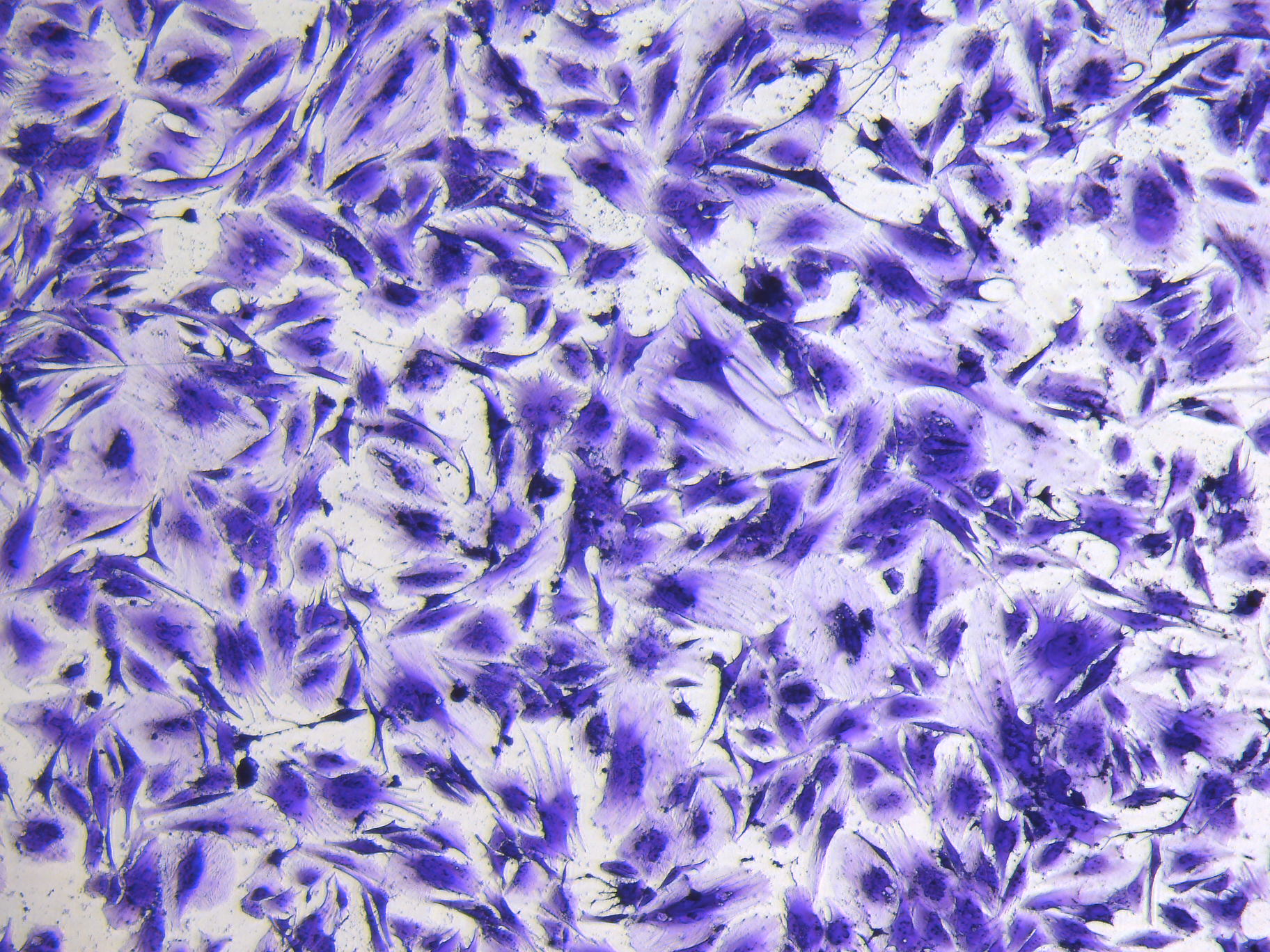

Supplement: Supplementary file 6 — Source data Fig. 4 [file 44321_2025_201_MOESM6_ESM.zip › Fig4/Fig4b CV/LN229/IR/Biri-D0.JPG]

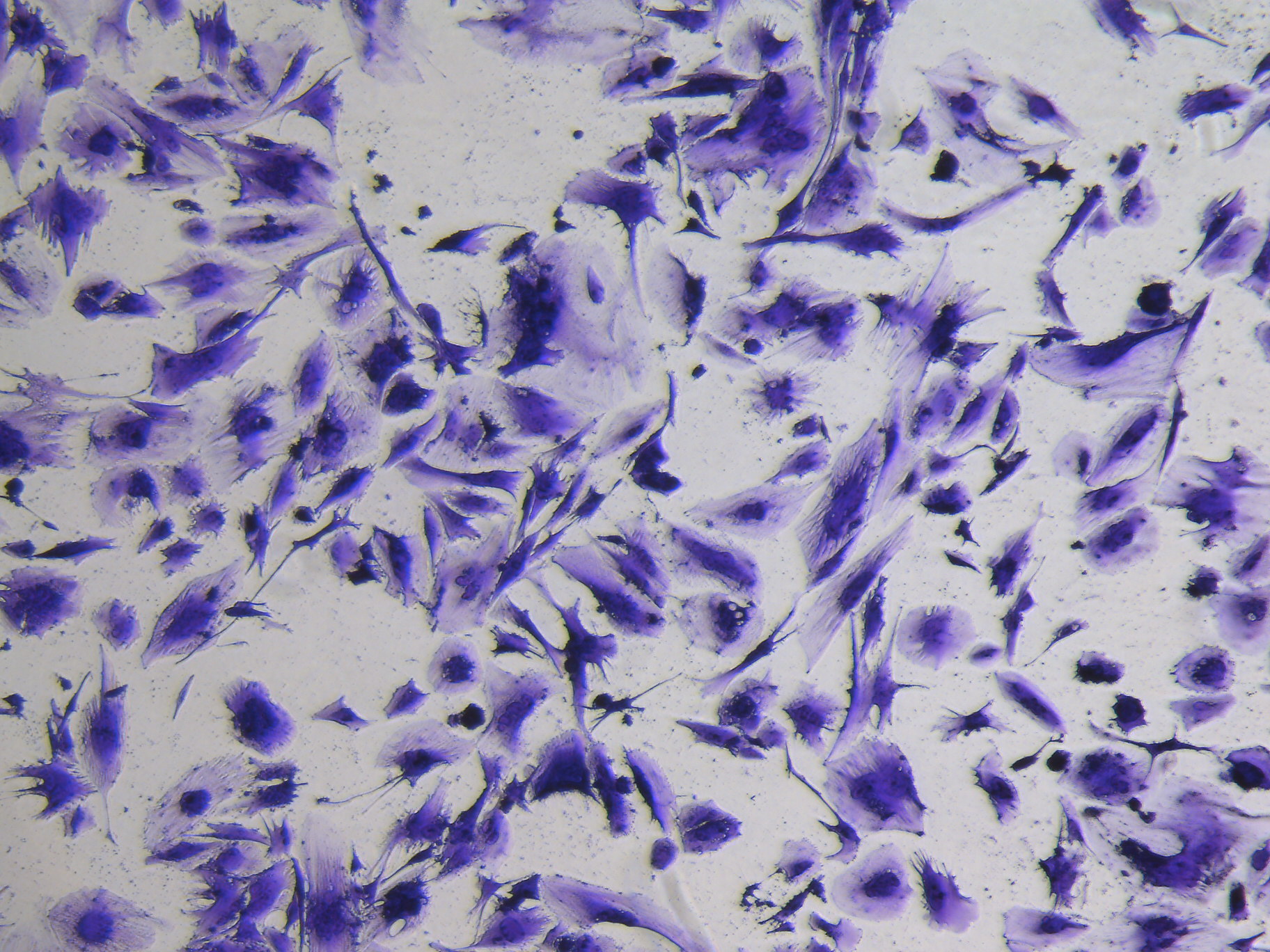

Supplement: Supplementary file 6 — Source data Fig. 4 [file 44321_2025_201_MOESM6_ESM.zip › Fig4/Fig4b CV/LN229/IR/Biri-D3.JPG]

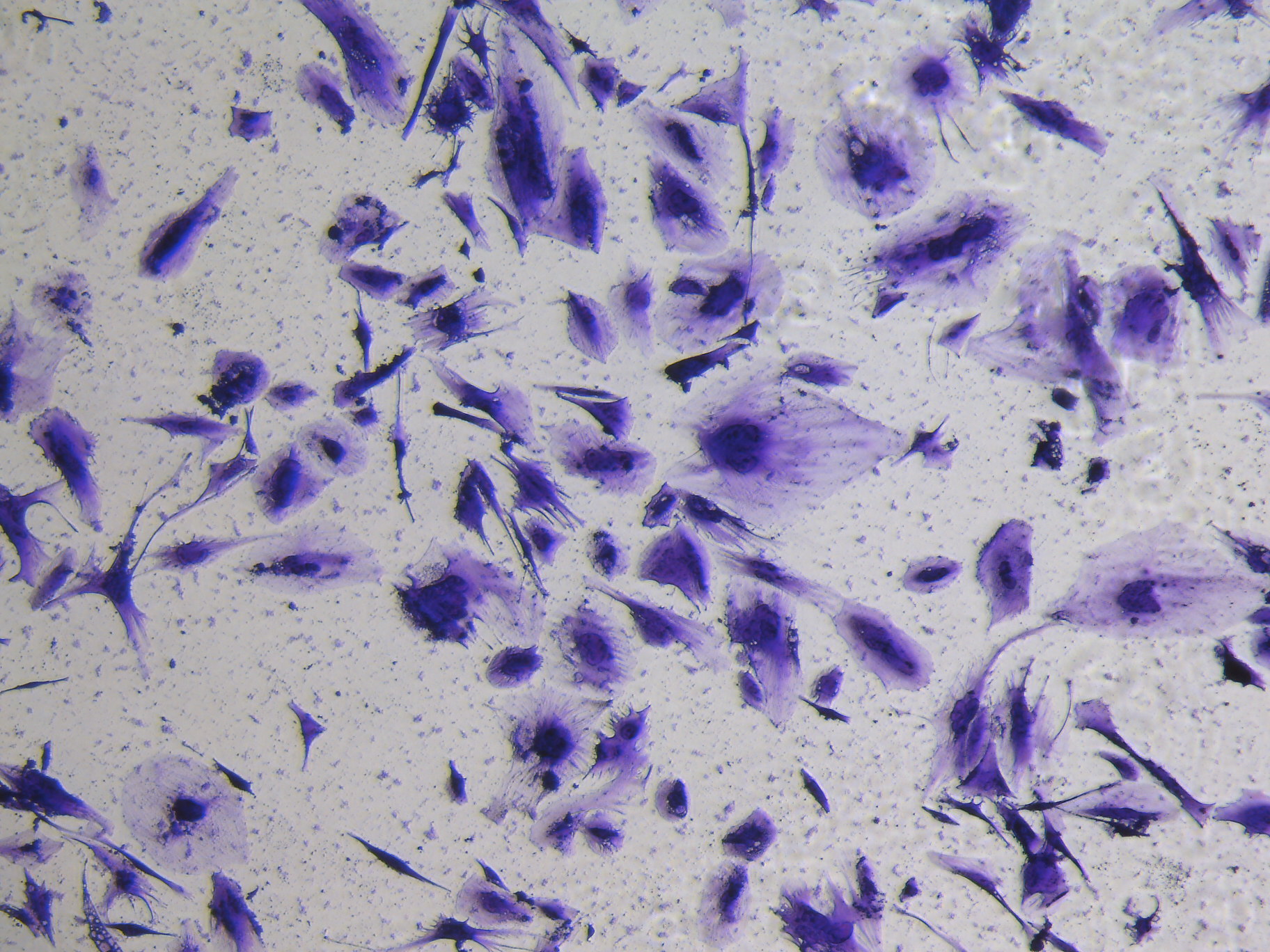

Supplement: Supplementary file 6 — Source data Fig. 4 [file 44321_2025_201_MOESM6_ESM.zip › Fig4/Fig4b CV/LN229/IR/Biri-D6.JPG]

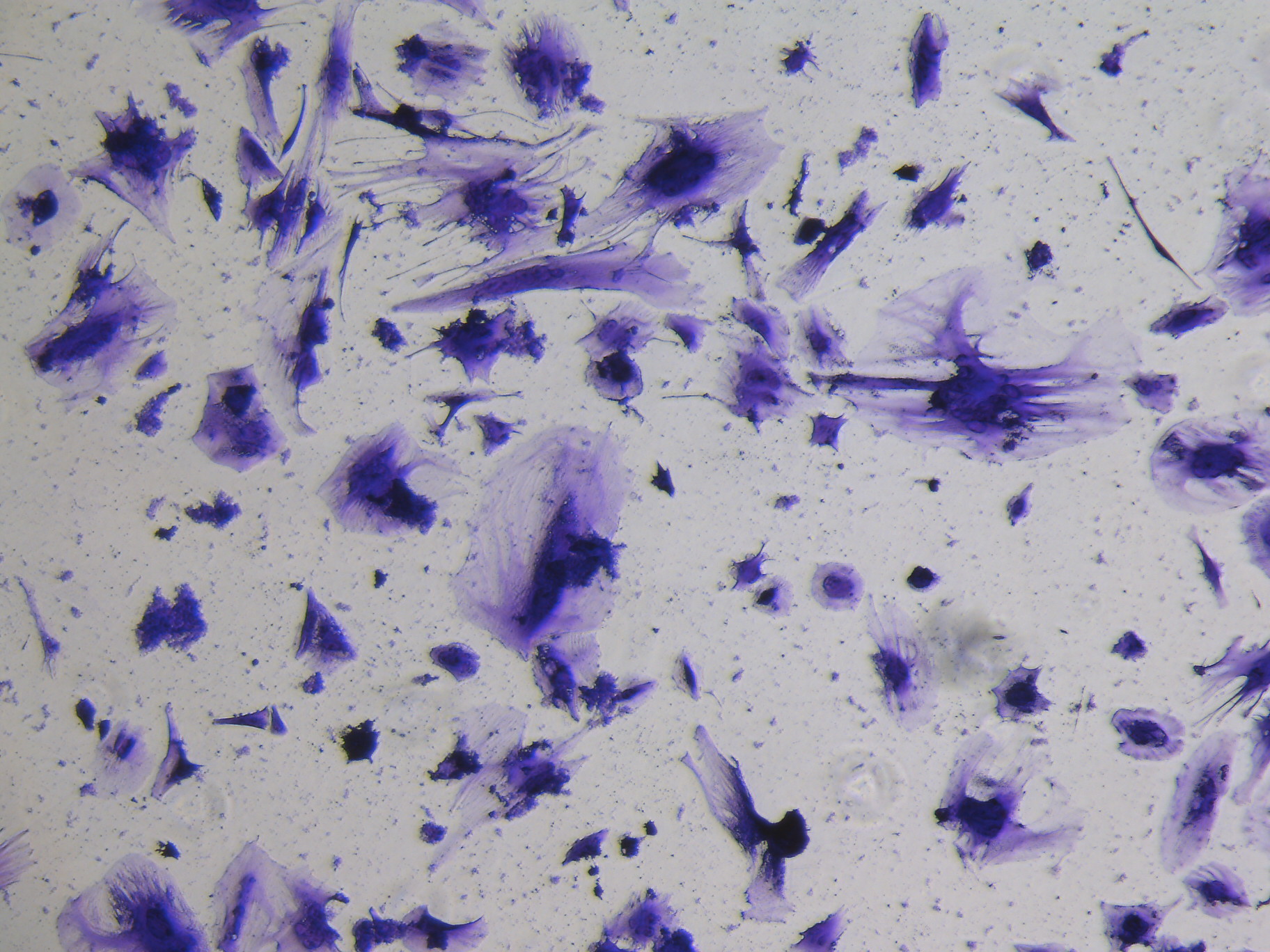

Supplement: Supplementary file 6 — Source data Fig. 4 [file 44321_2025_201_MOESM6_ESM.zip › Fig4/Fig4b CV/LN229/IR/Biri-D9.JPG]

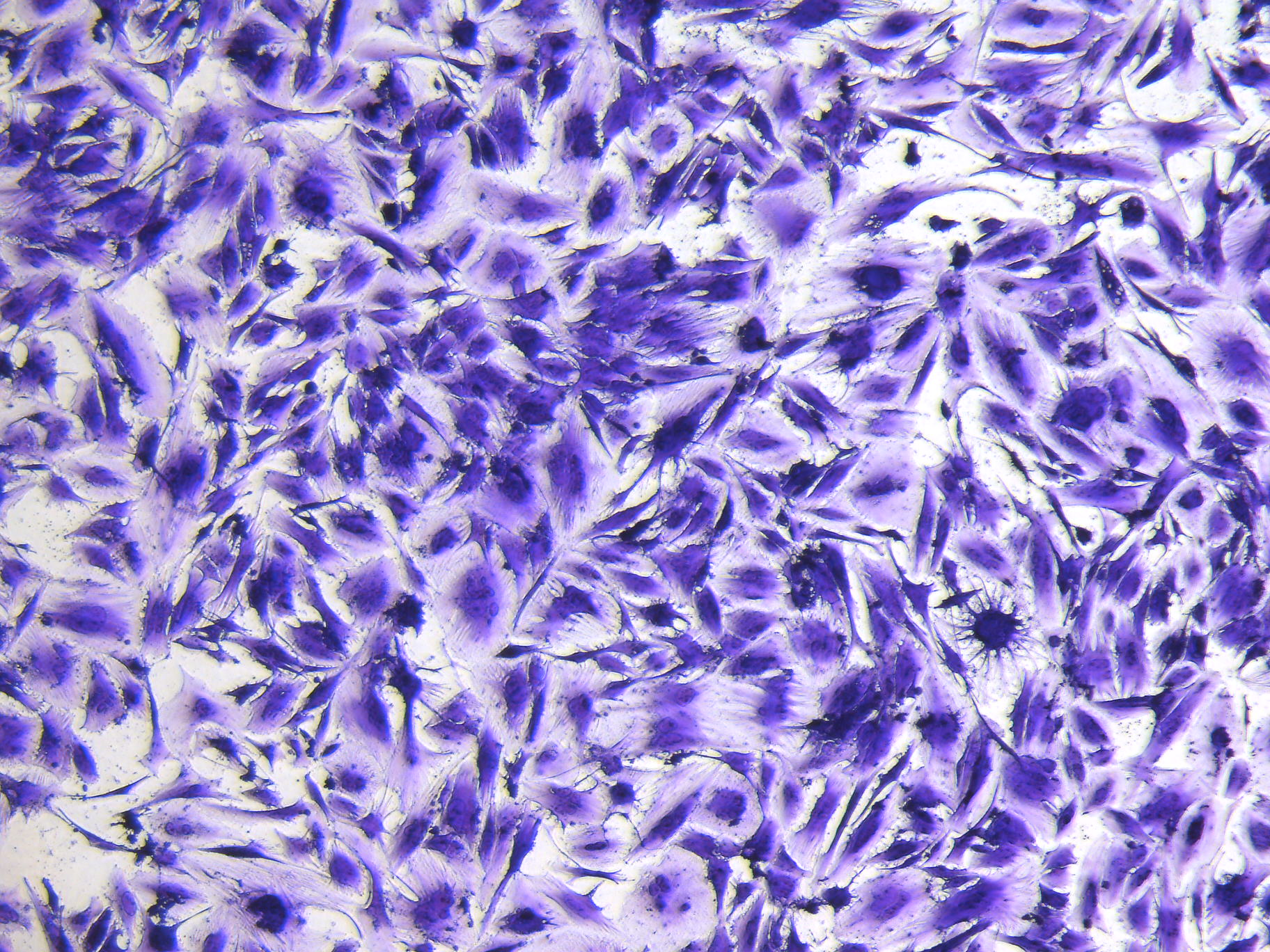

Supplement: Supplementary file 6 — Source data Fig. 4 [file 44321_2025_201_MOESM6_ESM.zip › Fig4/Fig4b CV/LN229/IR/DMSO-D0.JPG]

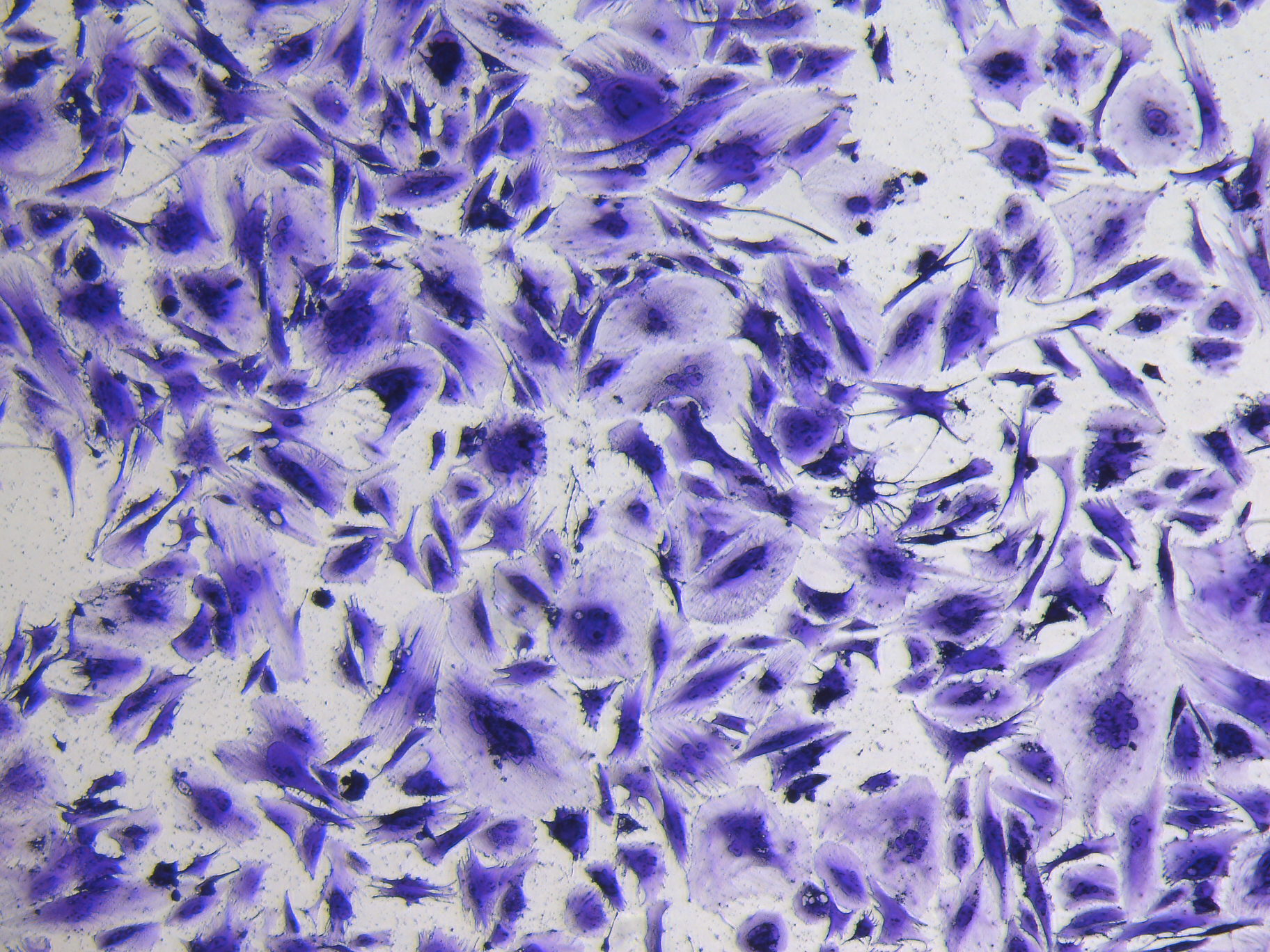

Supplement: Supplementary file 6 — Source data Fig. 4 [file 44321_2025_201_MOESM6_ESM.zip › Fig4/Fig4b CV/LN229/IR/DMSO-D3.JPG]

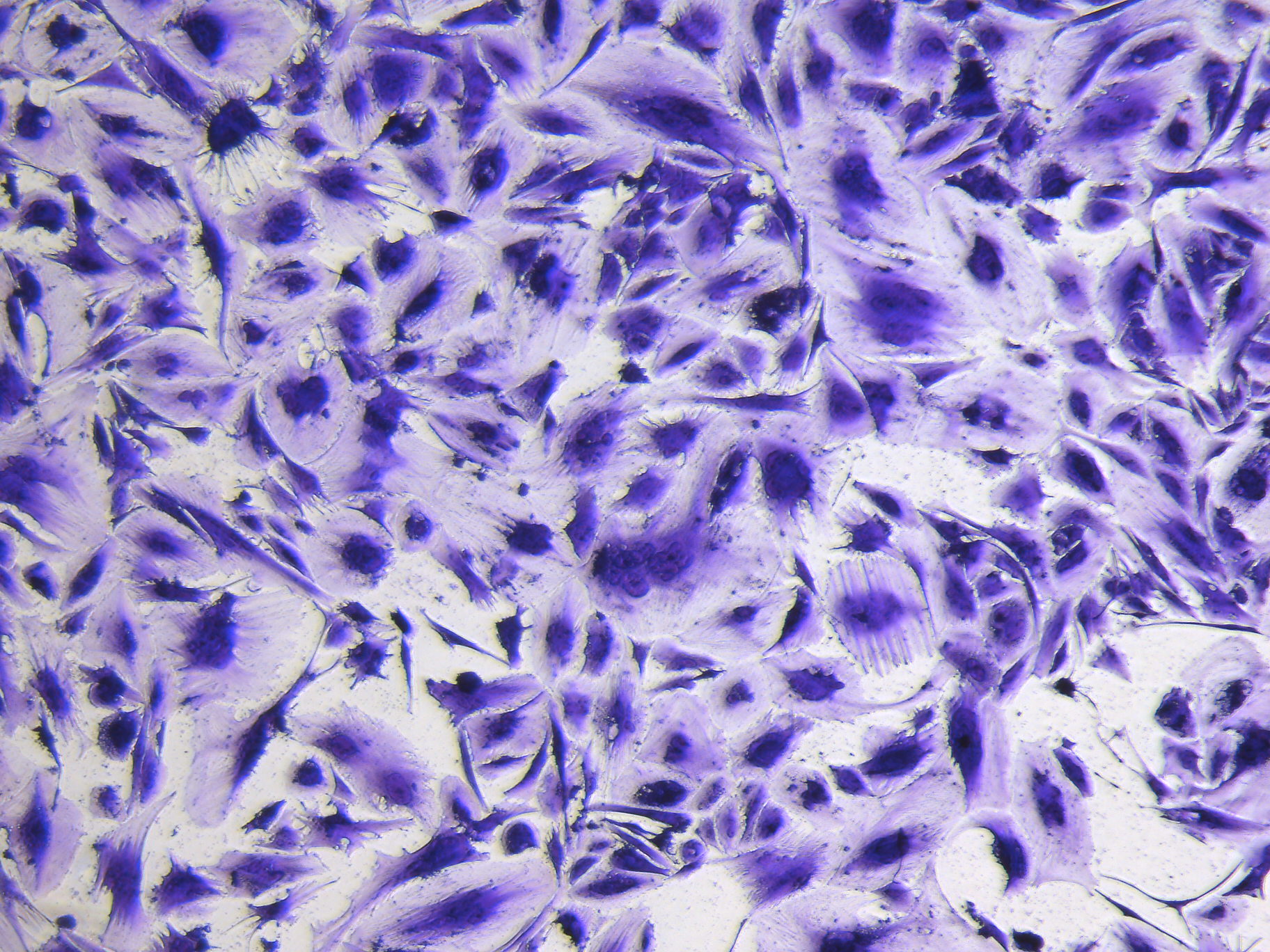

Supplement: Supplementary file 6 — Source data Fig. 4 [file 44321_2025_201_MOESM6_ESM.zip › Fig4/Fig4b CV/LN229/IR/DMSO-D6.JPG]

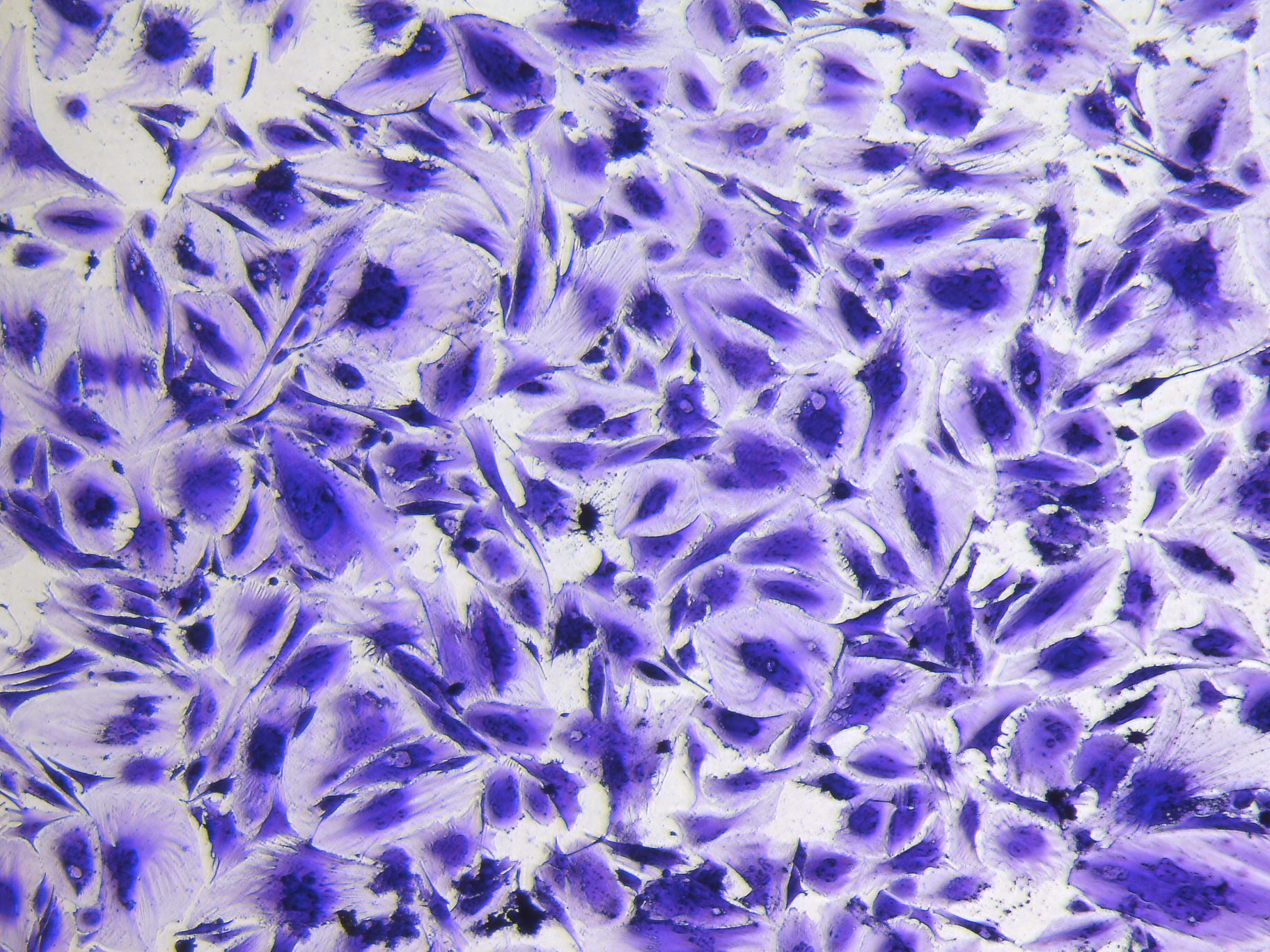

Supplement: Supplementary file 6 — Source data Fig. 4 [file 44321_2025_201_MOESM6_ESM.zip › Fig4/Fig4b CV/LN229/IR/DMSO-D9.JPG]

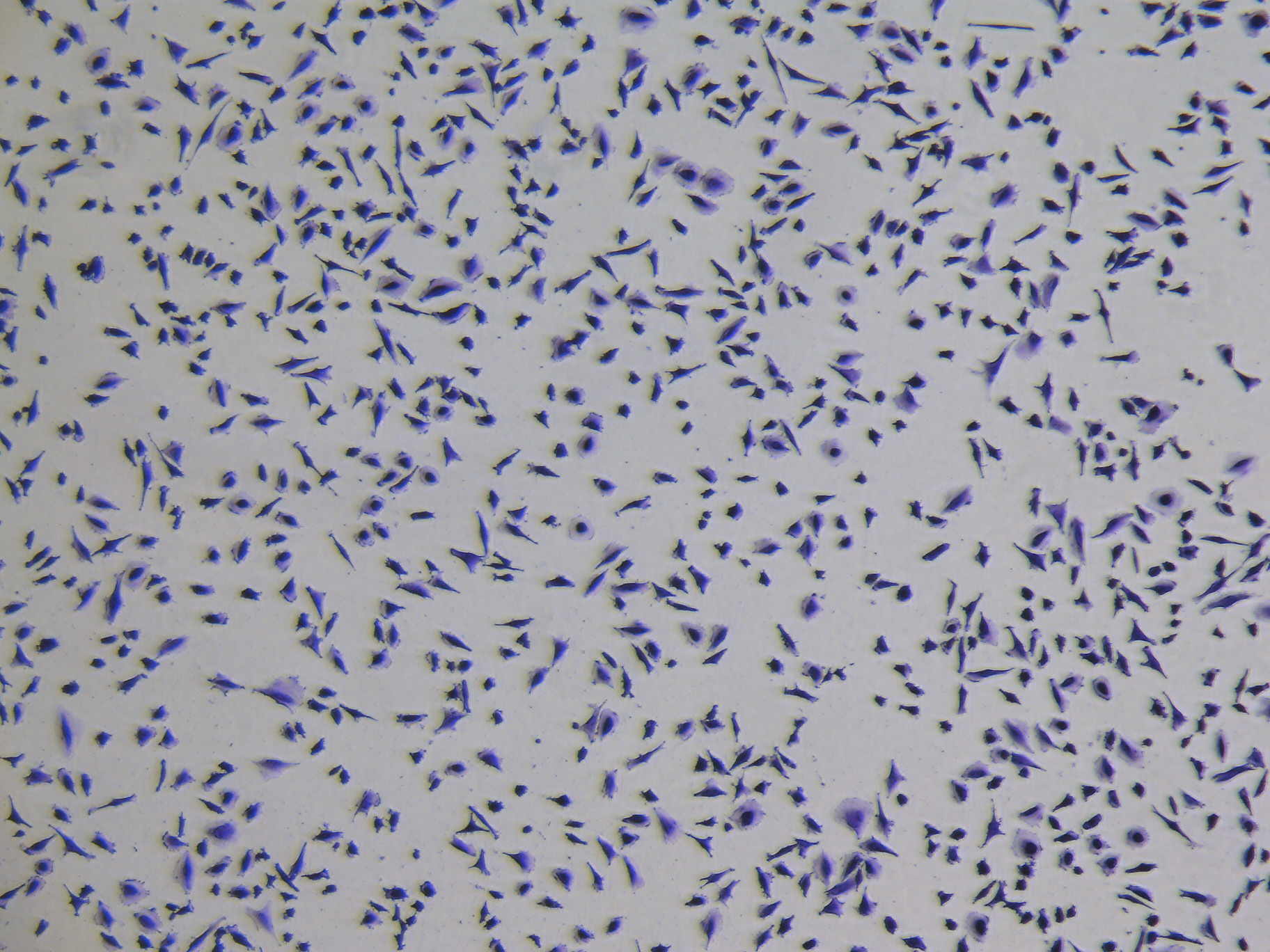

Supplement: Supplementary file 6 — Source data Fig. 4 [file 44321_2025_201_MOESM6_ESM.zip › Fig4/Fig4b CV/LN229/mock/Biri-D0.JPG]

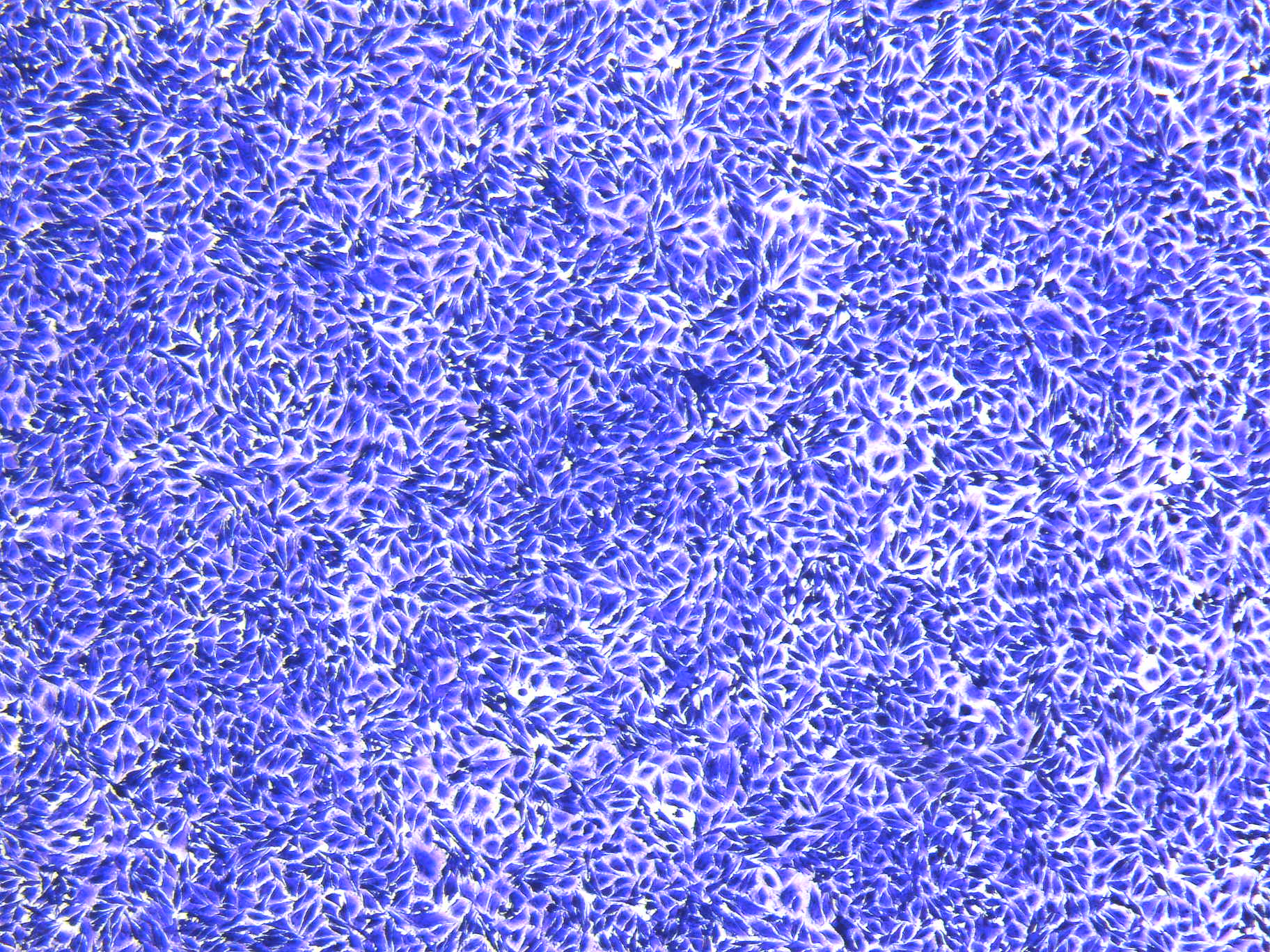

Supplement: Supplementary file 6 — Source data Fig. 4 [file 44321_2025_201_MOESM6_ESM.zip › Fig4/Fig4b CV/LN229/mock/Biri-D3.JPG]

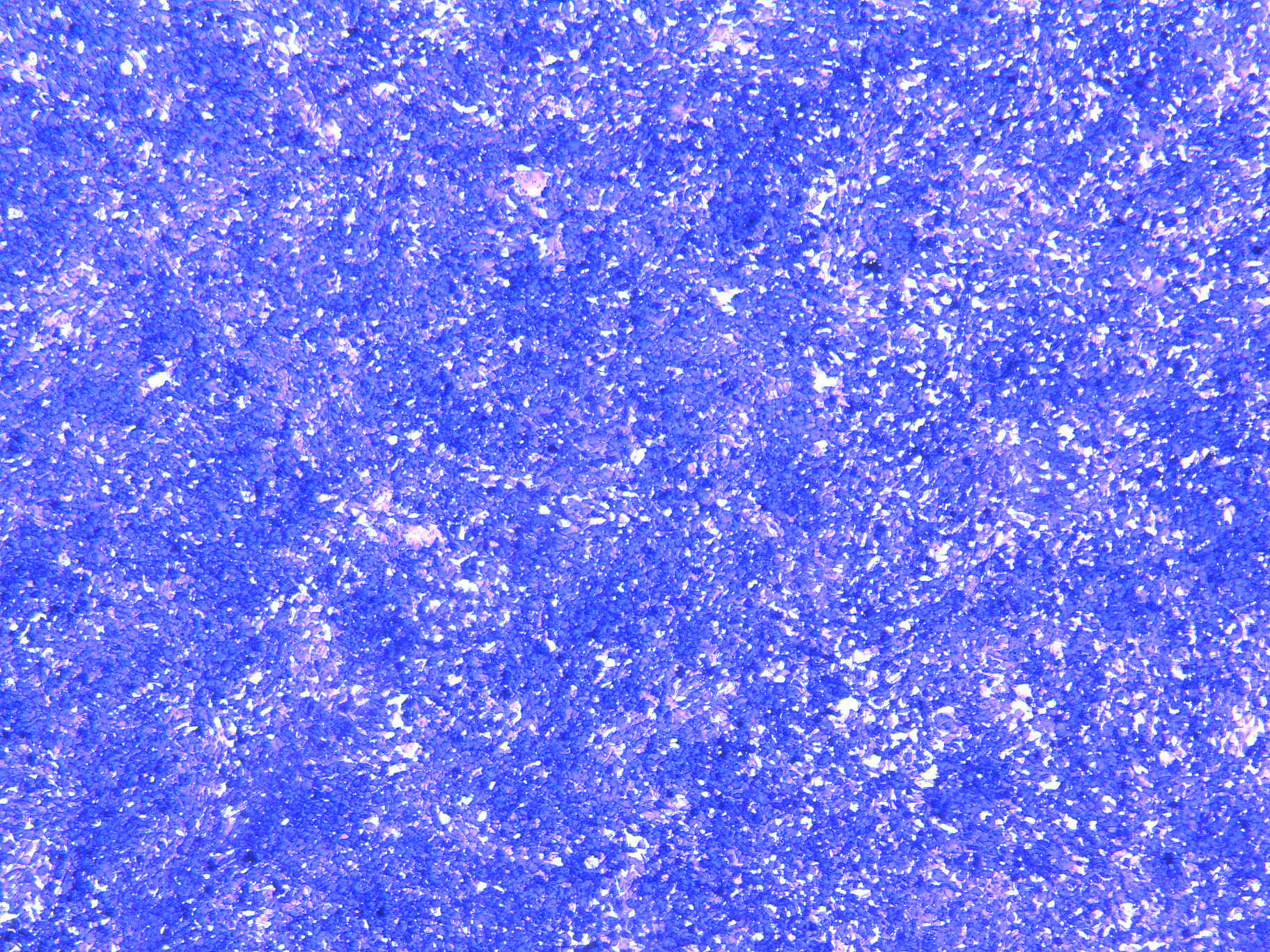

Supplement: Supplementary file 6 — Source data Fig. 4 [file 44321_2025_201_MOESM6_ESM.zip › Fig4/Fig4b CV/LN229/mock/Biri-D6.JPG]

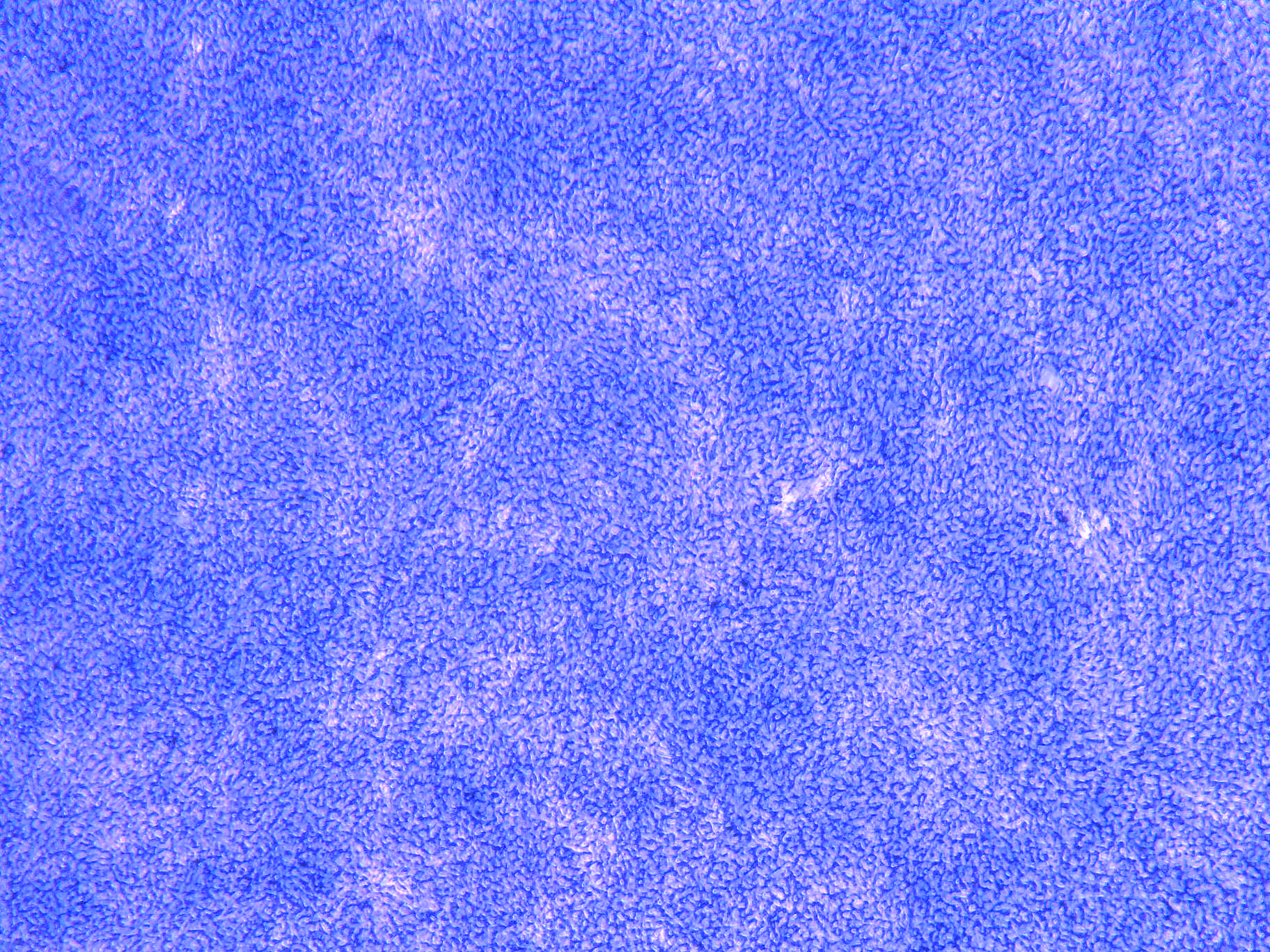

Supplement: Supplementary file 6 — Source data Fig. 4 [file 44321_2025_201_MOESM6_ESM.zip › Fig4/Fig4b CV/LN229/mock/Biri-D9.JPG]

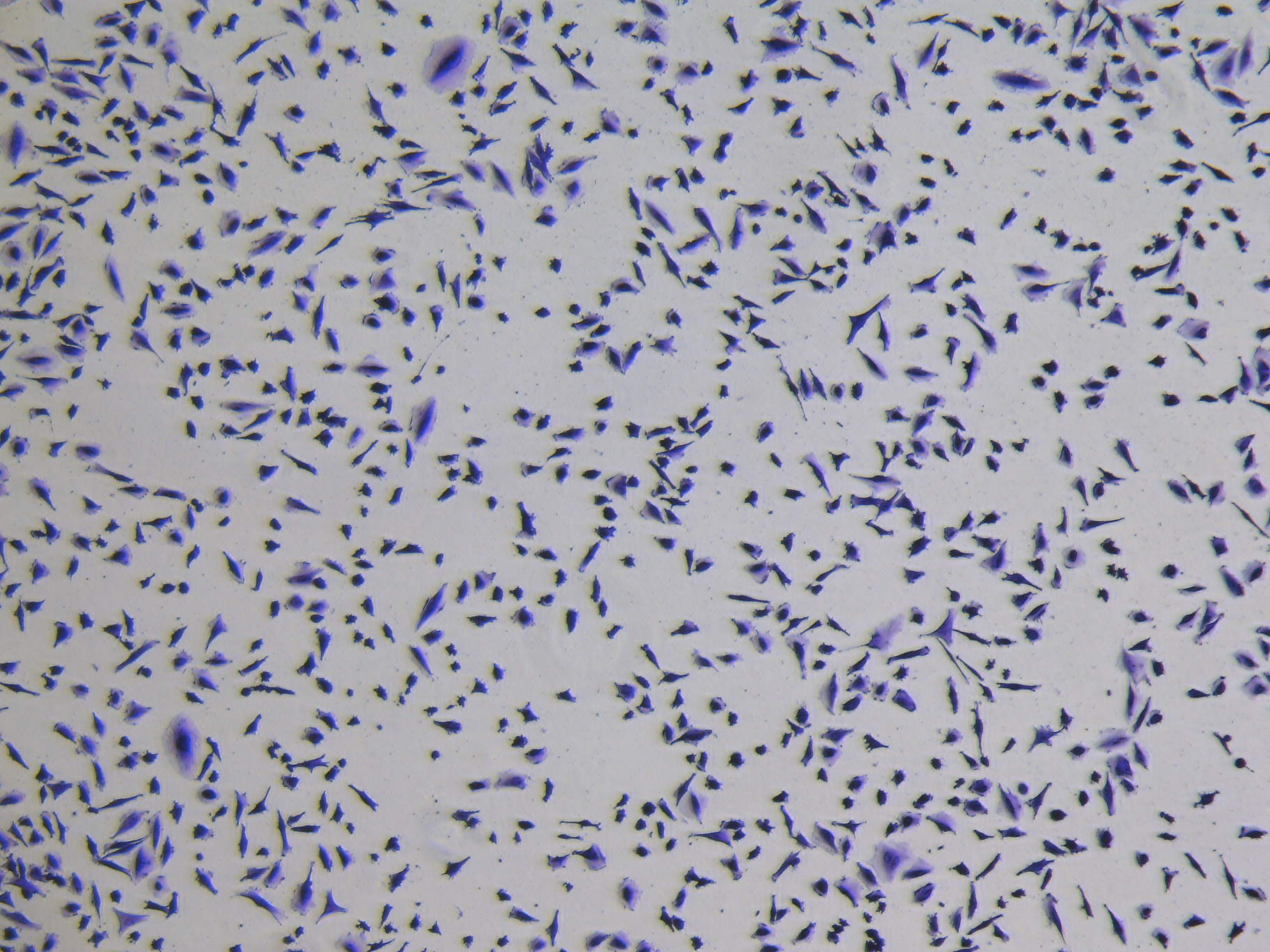

Supplement: Supplementary file 6 — Source data Fig. 4 [file 44321_2025_201_MOESM6_ESM.zip › Fig4/Fig4b CV/LN229/mock/DMSO-D0.JPG]

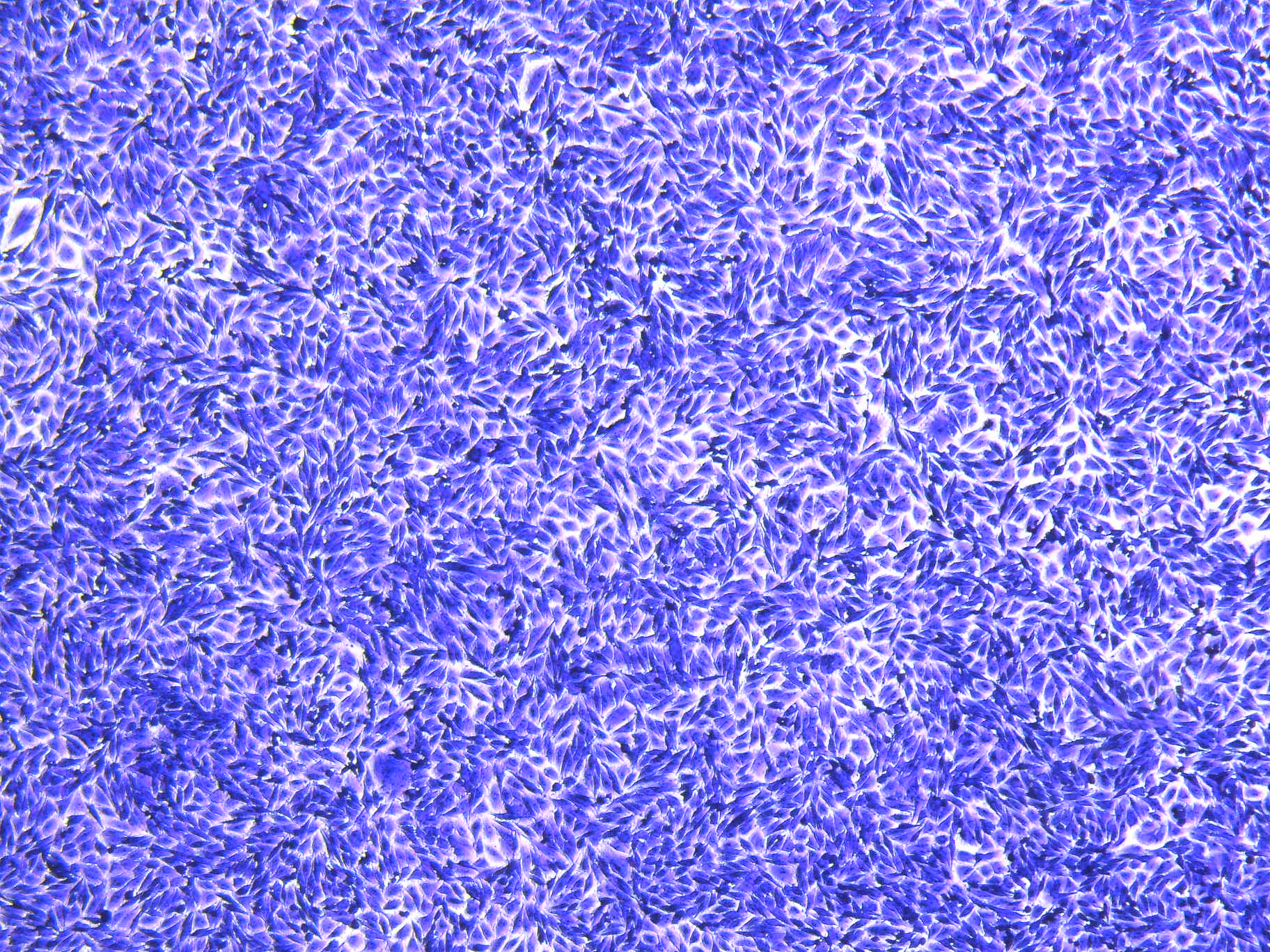

Supplement: Supplementary file 6 — Source data Fig. 4 [file 44321_2025_201_MOESM6_ESM.zip › Fig4/Fig4b CV/LN229/mock/DMSO-D3.JPG]

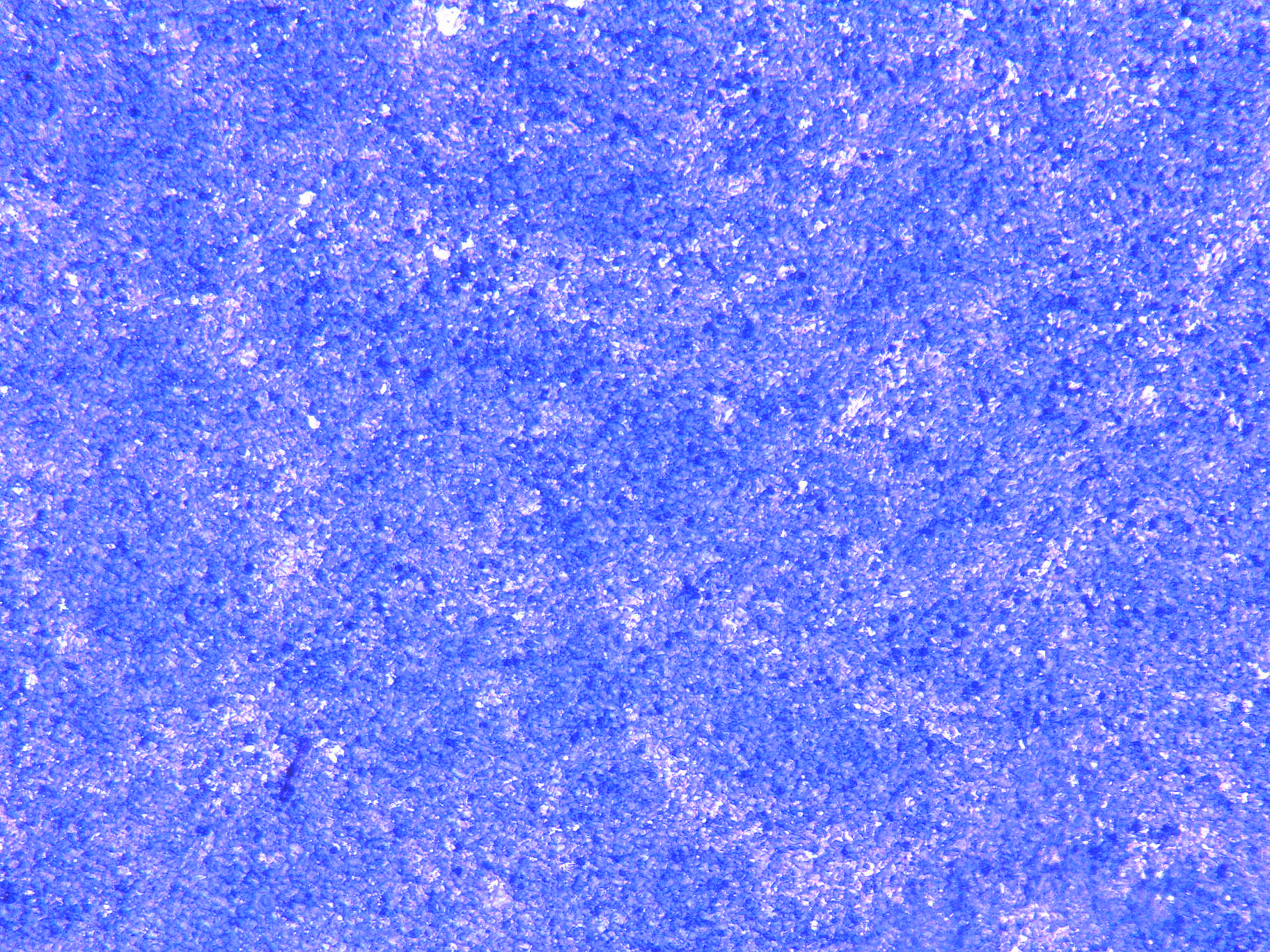

Supplement: Supplementary file 6 — Source data Fig. 4 [file 44321_2025_201_MOESM6_ESM.zip › Fig4/Fig4b CV/LN229/mock/DMSO-D6.JPG]

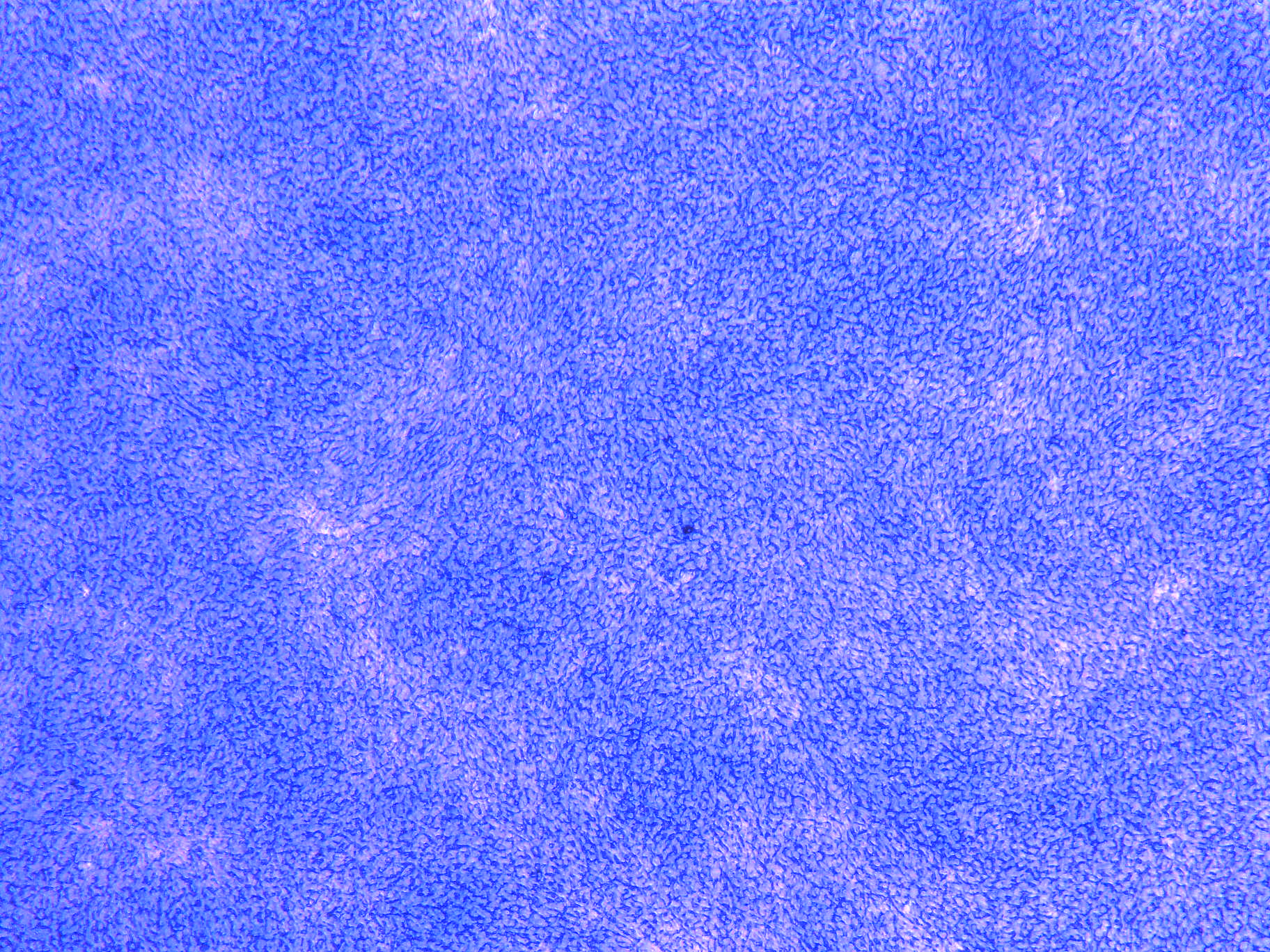

Supplement: Supplementary file 6 — Source data Fig. 4 [file 44321_2025_201_MOESM6_ESM.zip › Fig4/Fig4b CV/LN229/mock/DMSO-D9.JPG]

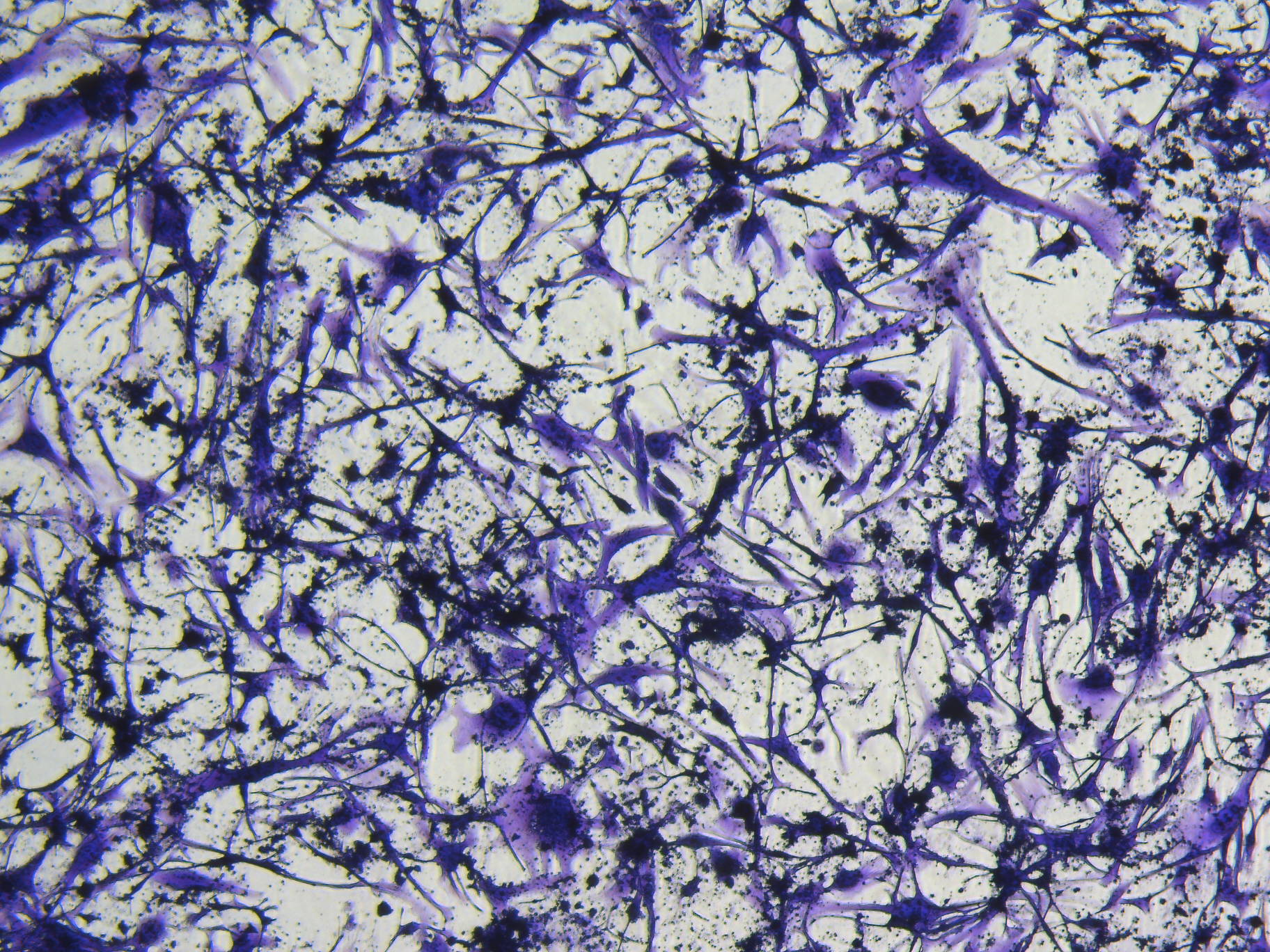

Supplement: Supplementary file 6 — Source data Fig. 4 [file 44321_2025_201_MOESM6_ESM.zip › Fig4/Fig4b CV/U118/IR/Biri-D0.JPG]

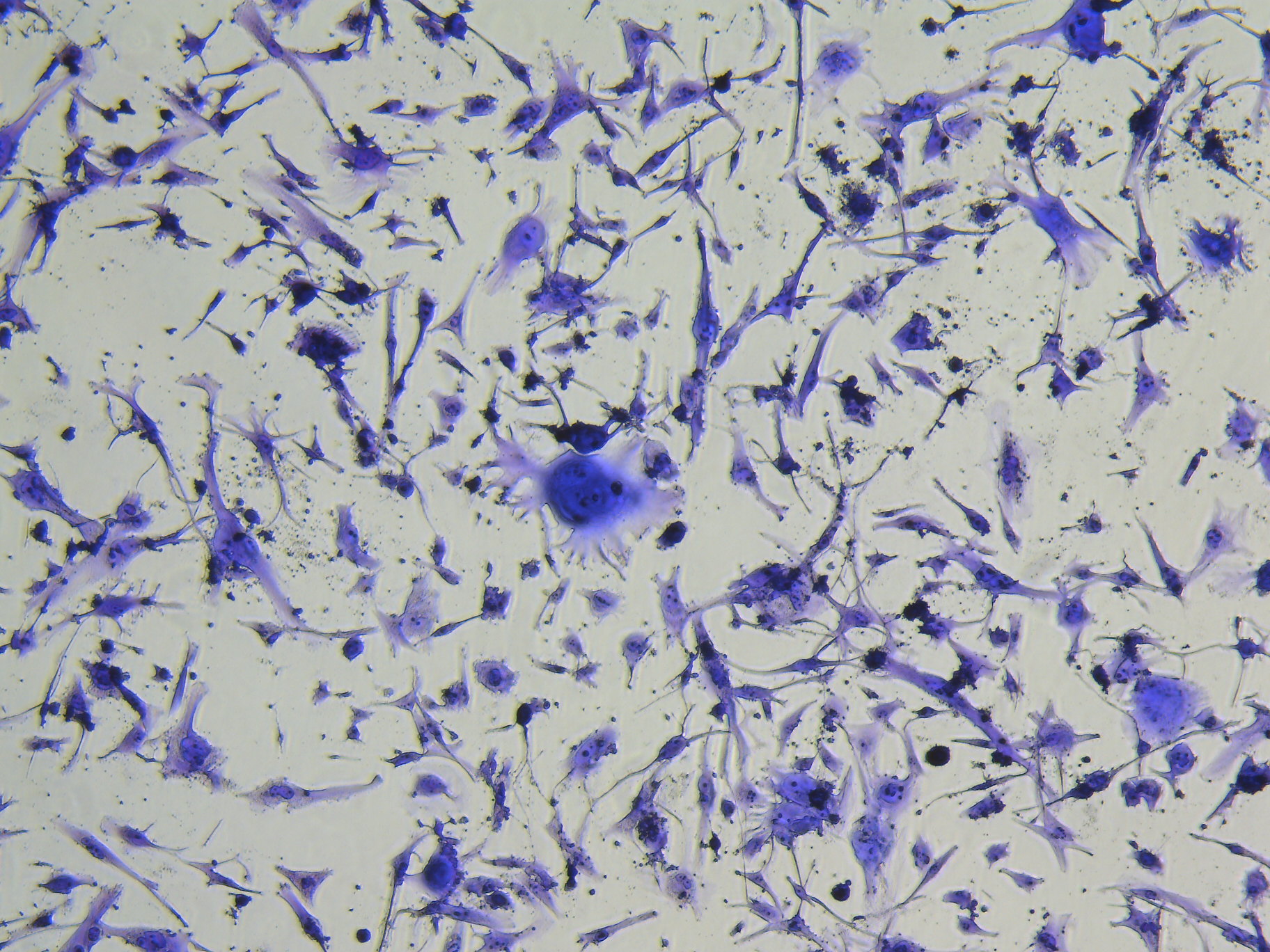

Supplement: Supplementary file 6 — Source data Fig. 4 [file 44321_2025_201_MOESM6_ESM.zip › Fig4/Fig4b CV/U118/IR/Biri-D3.JPG]

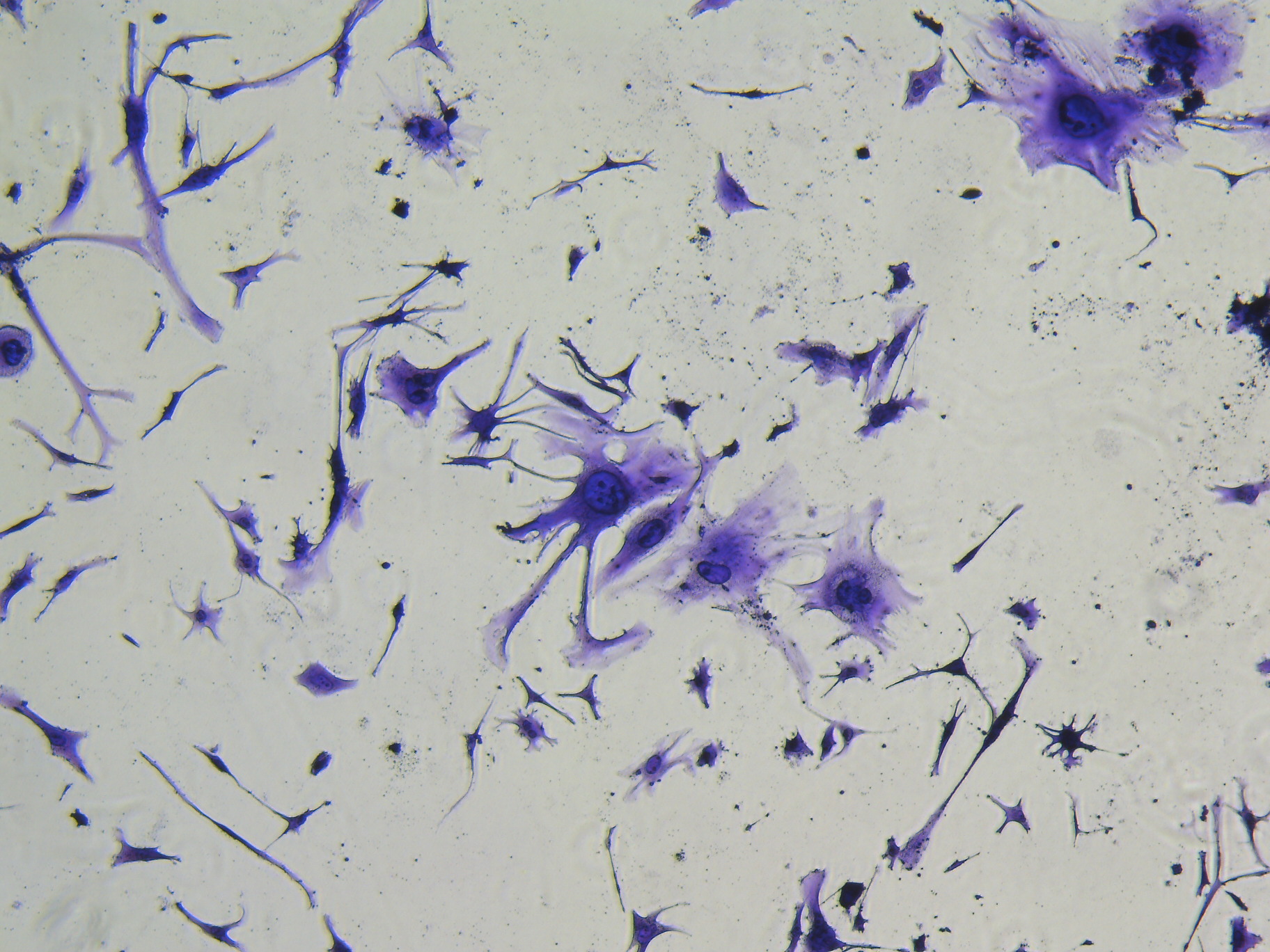

Supplement: Supplementary file 6 — Source data Fig. 4 [file 44321_2025_201_MOESM6_ESM.zip › Fig4/Fig4b CV/U118/IR/Biri-D6.JPG]

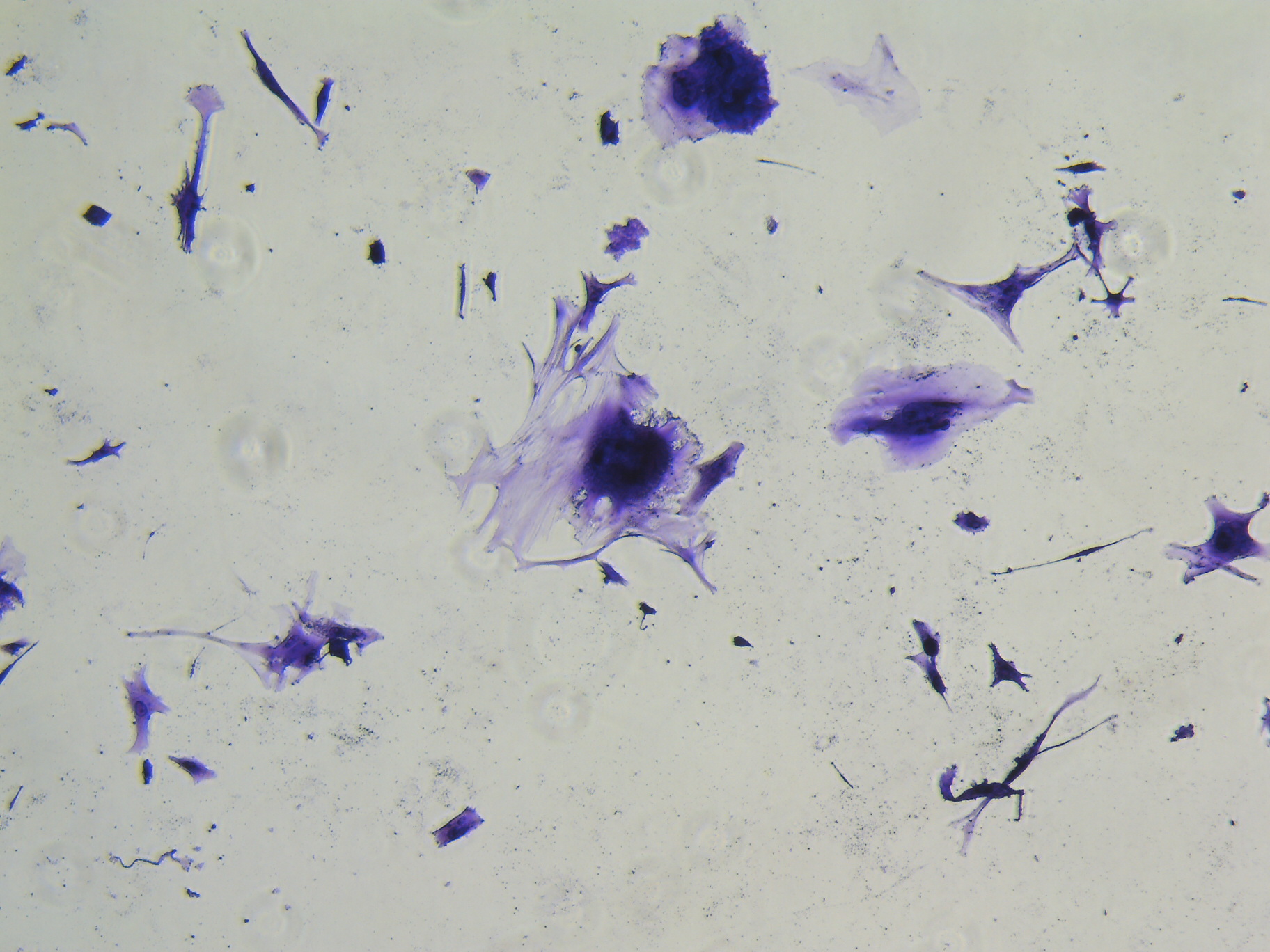

Supplement: Supplementary file 6 — Source data Fig. 4 [file 44321_2025_201_MOESM6_ESM.zip › Fig4/Fig4b CV/U118/IR/Biri-D9.JPG]

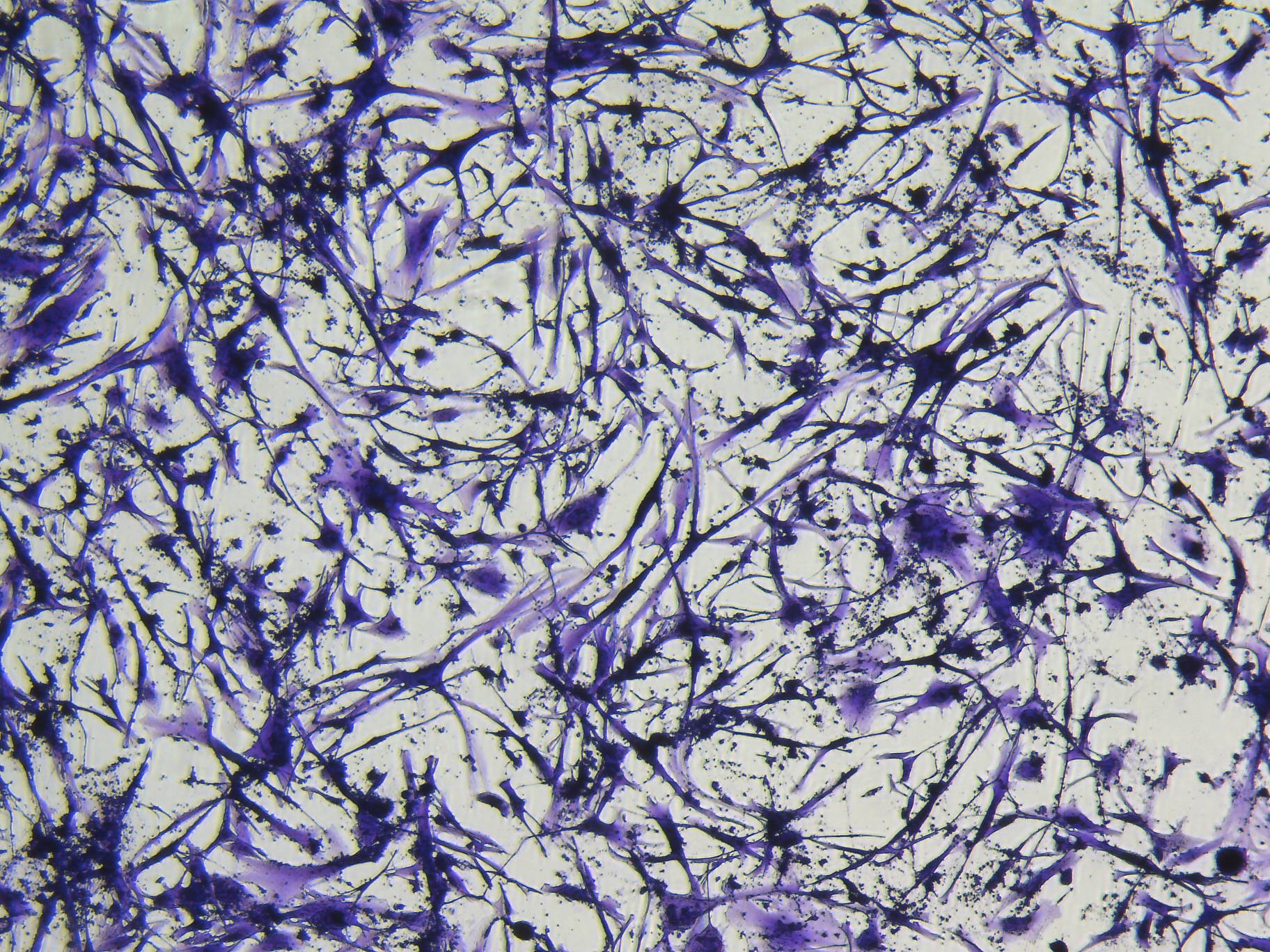

Supplement: Supplementary file 6 — Source data Fig. 4 [file 44321_2025_201_MOESM6_ESM.zip › Fig4/Fig4b CV/U118/IR/DMSO-D0.JPG]

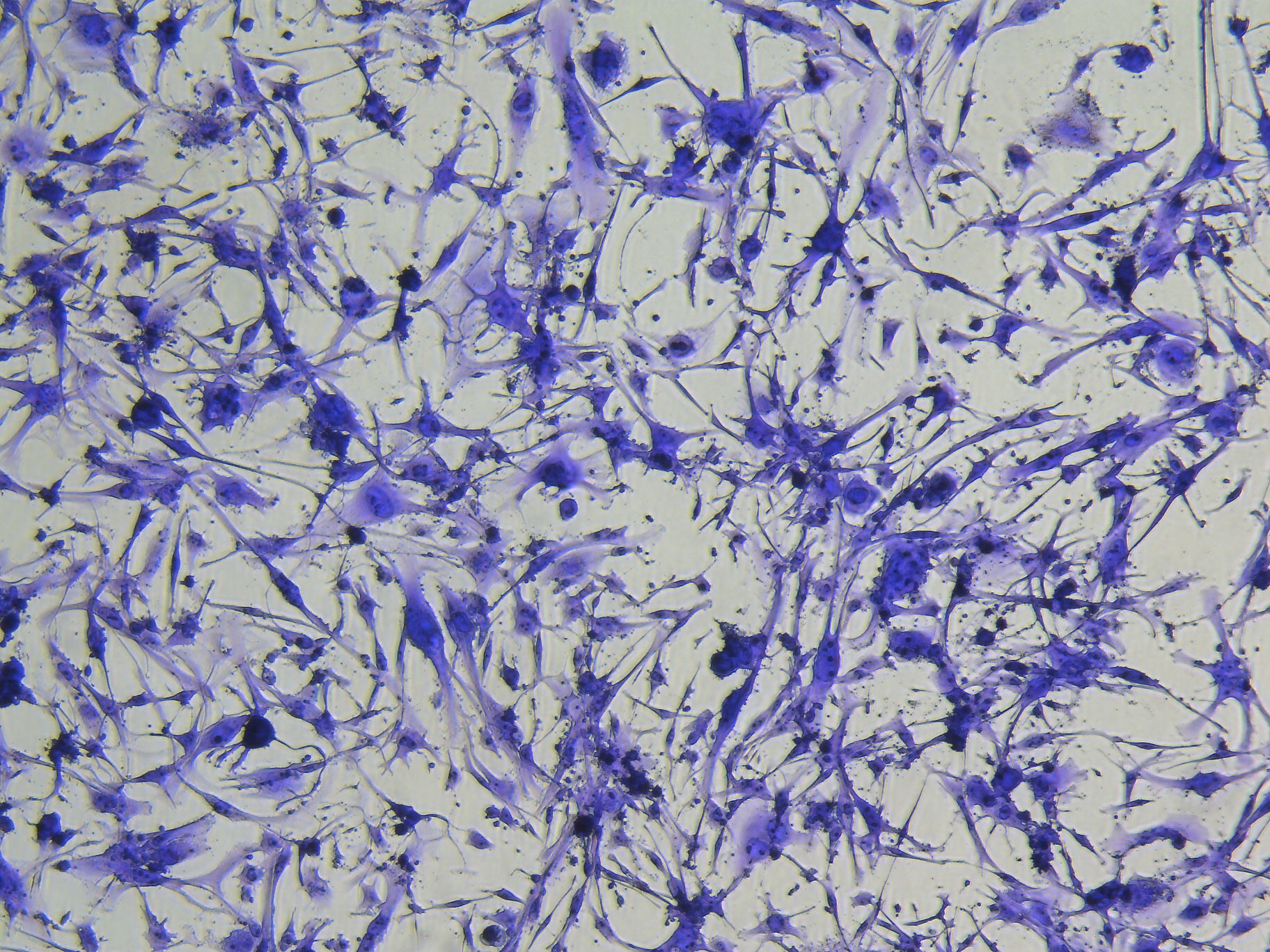

Supplement: Supplementary file 6 — Source data Fig. 4 [file 44321_2025_201_MOESM6_ESM.zip › Fig4/Fig4b CV/U118/IR/DMSO-D3.JPG]

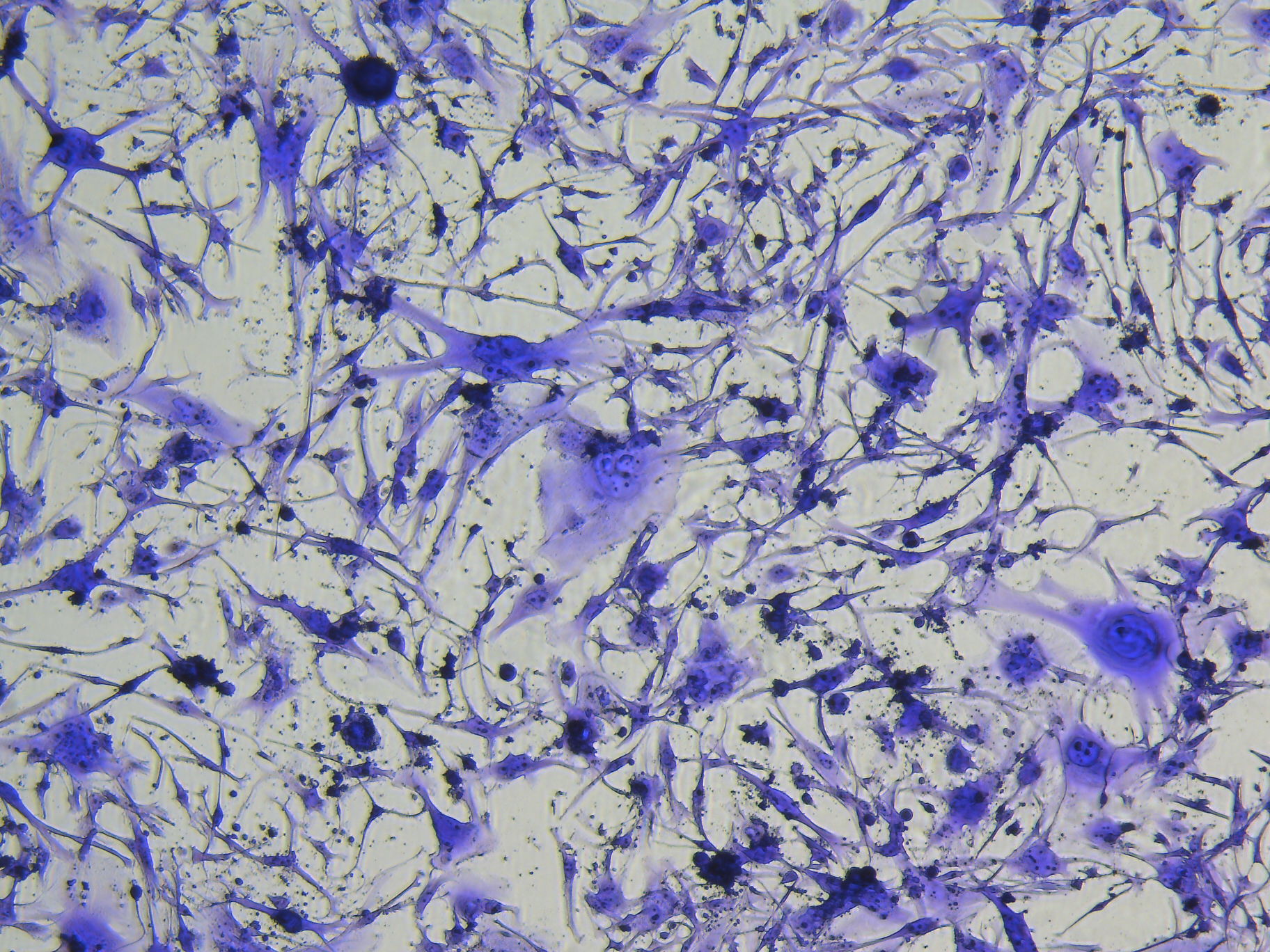

Supplement: Supplementary file 6 — Source data Fig. 4 [file 44321_2025_201_MOESM6_ESM.zip › Fig4/Fig4b CV/U118/IR/DMSO-D6.JPG]

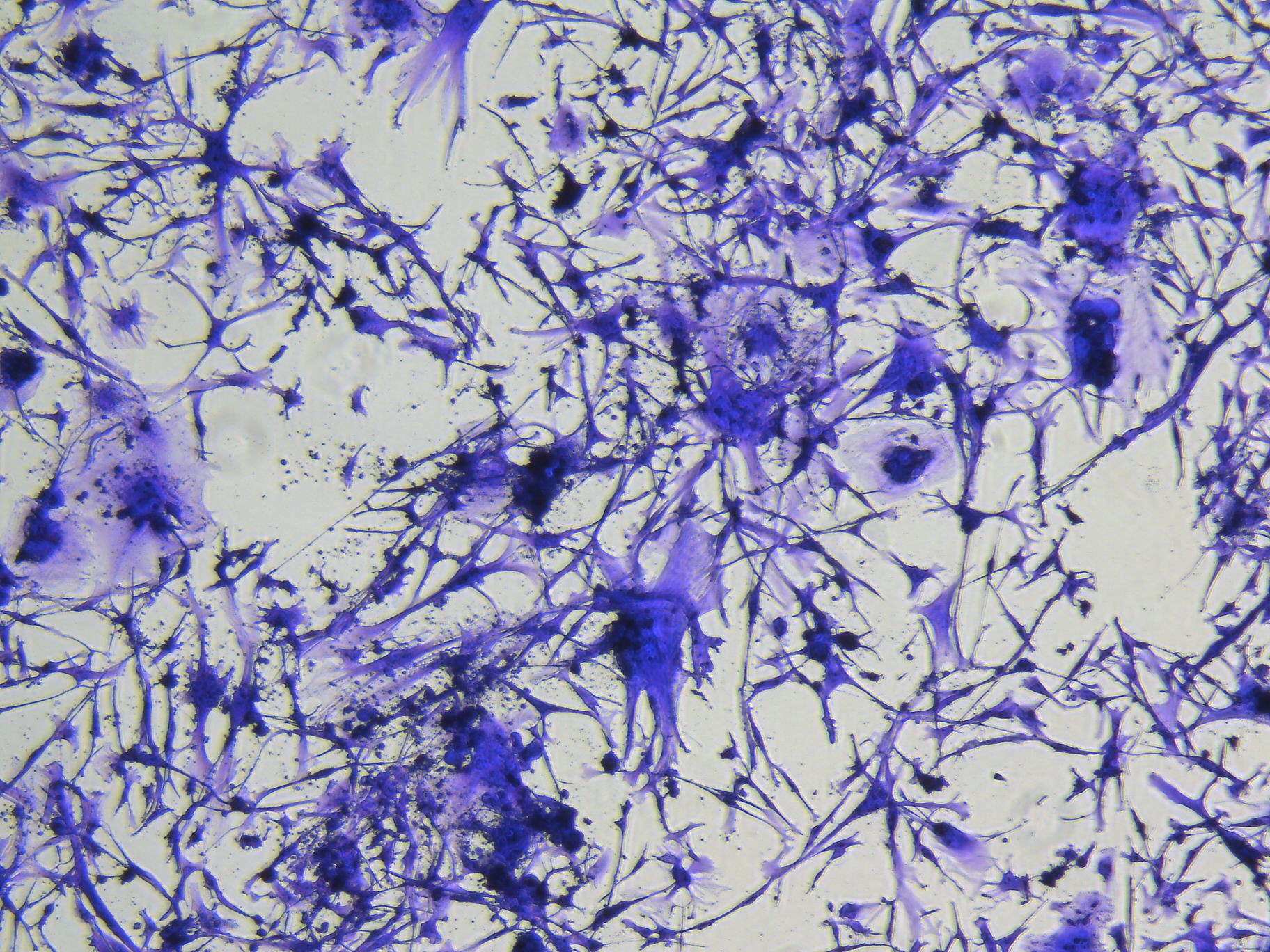

Supplement: Supplementary file 6 — Source data Fig. 4 [file 44321_2025_201_MOESM6_ESM.zip › Fig4/Fig4b CV/U118/IR/DMSO-D9.JPG]

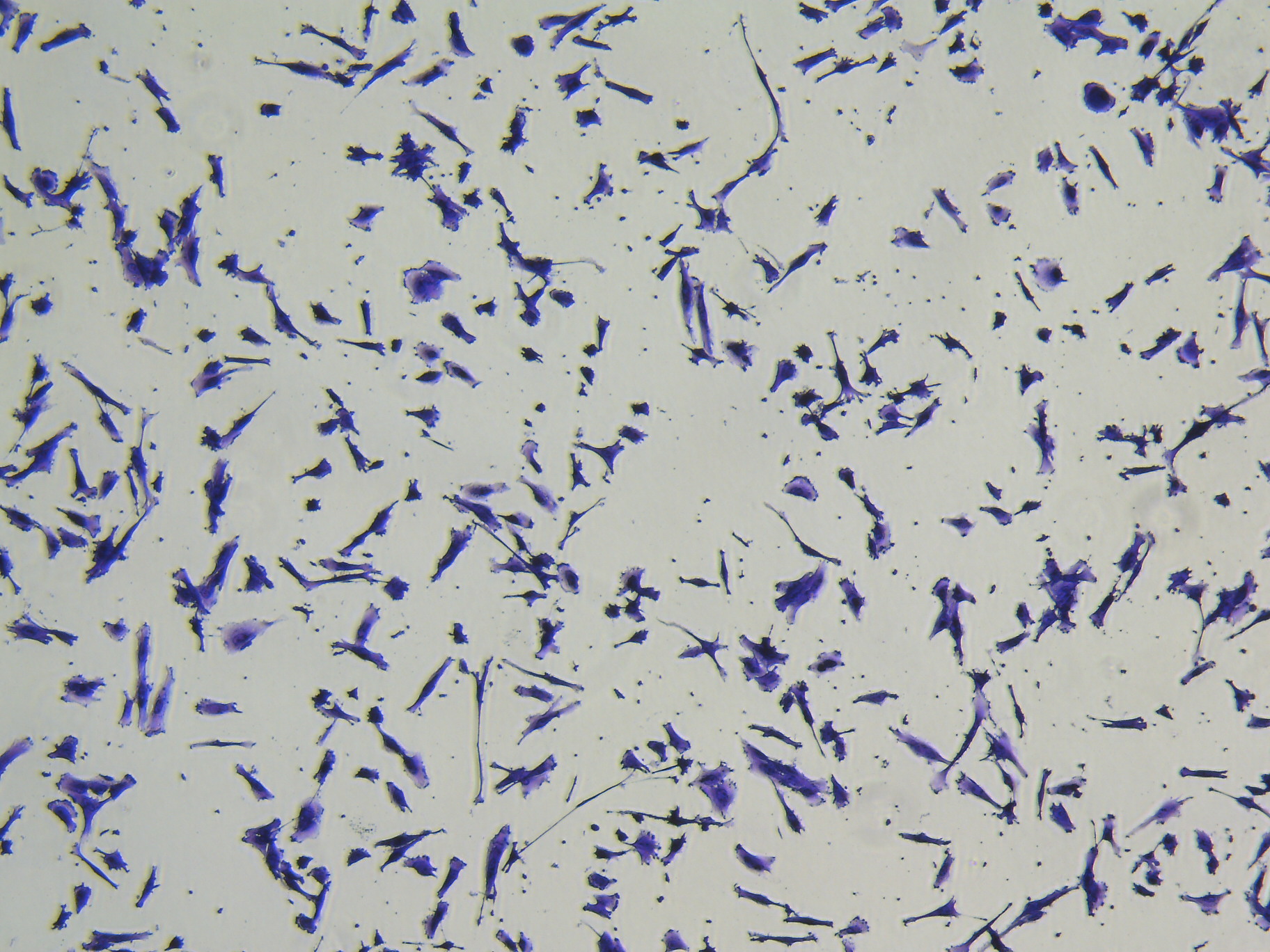

Supplement: Supplementary file 6 — Source data Fig. 4 [file 44321_2025_201_MOESM6_ESM.zip › Fig4/Fig4b CV/U118/mock/Biri-D0.JPG]

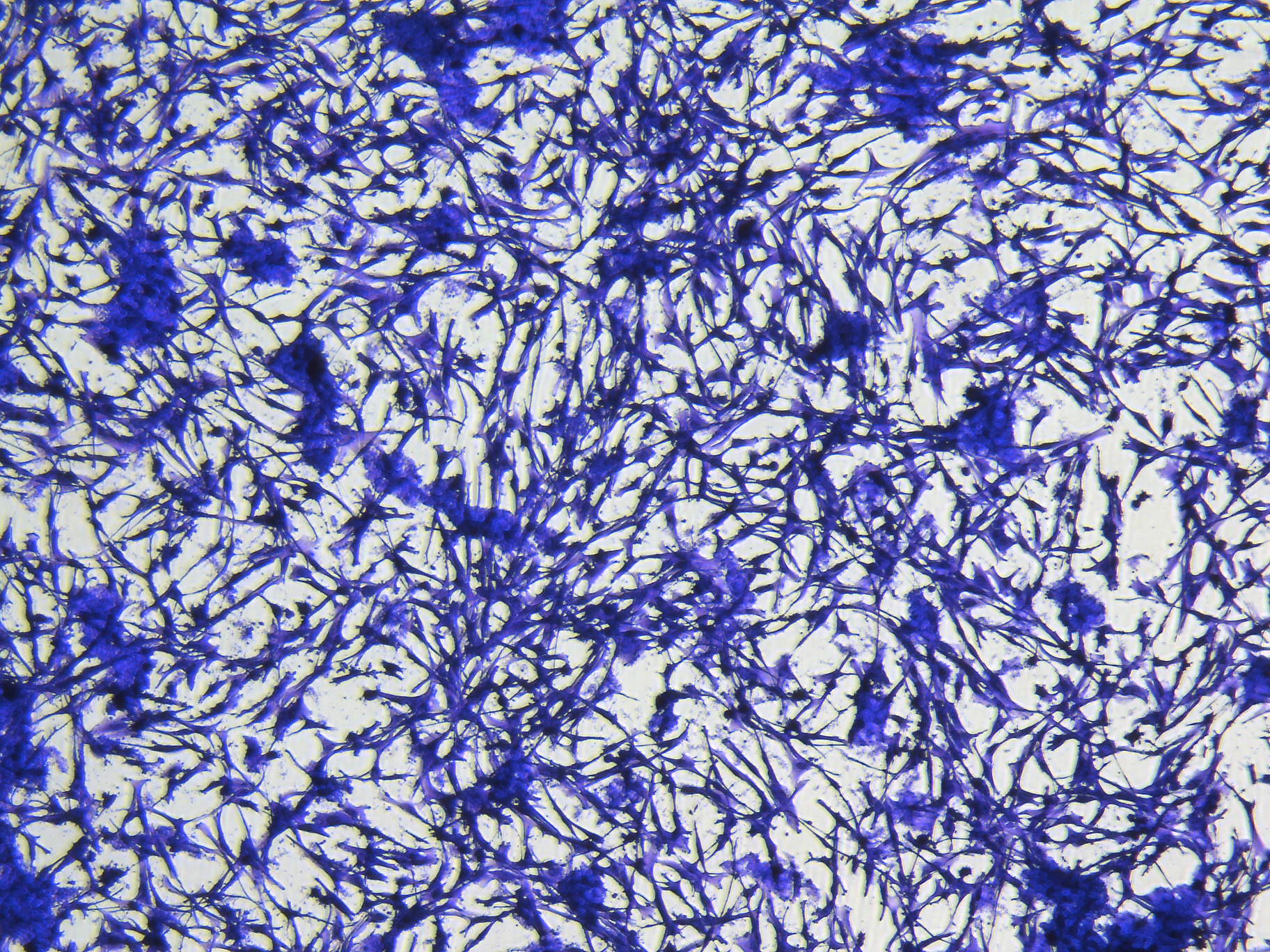

Supplement: Supplementary file 6 — Source data Fig. 4 [file 44321_2025_201_MOESM6_ESM.zip › Fig4/Fig4b CV/U118/mock/Biri-D3.JPG]

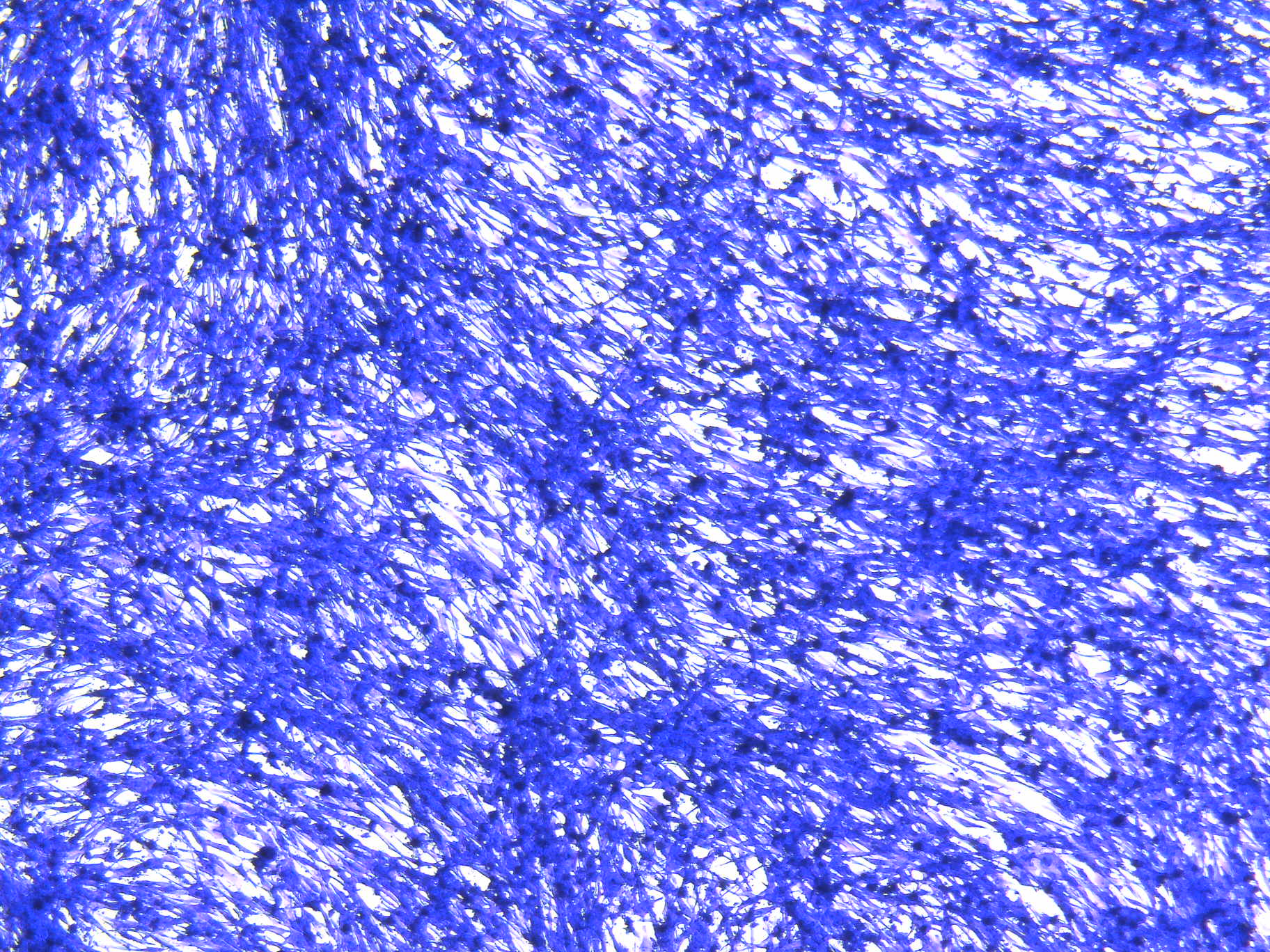

Supplement: Supplementary file 6 — Source data Fig. 4 [file 44321_2025_201_MOESM6_ESM.zip › Fig4/Fig4b CV/U118/mock/Biri-D6.JPG]

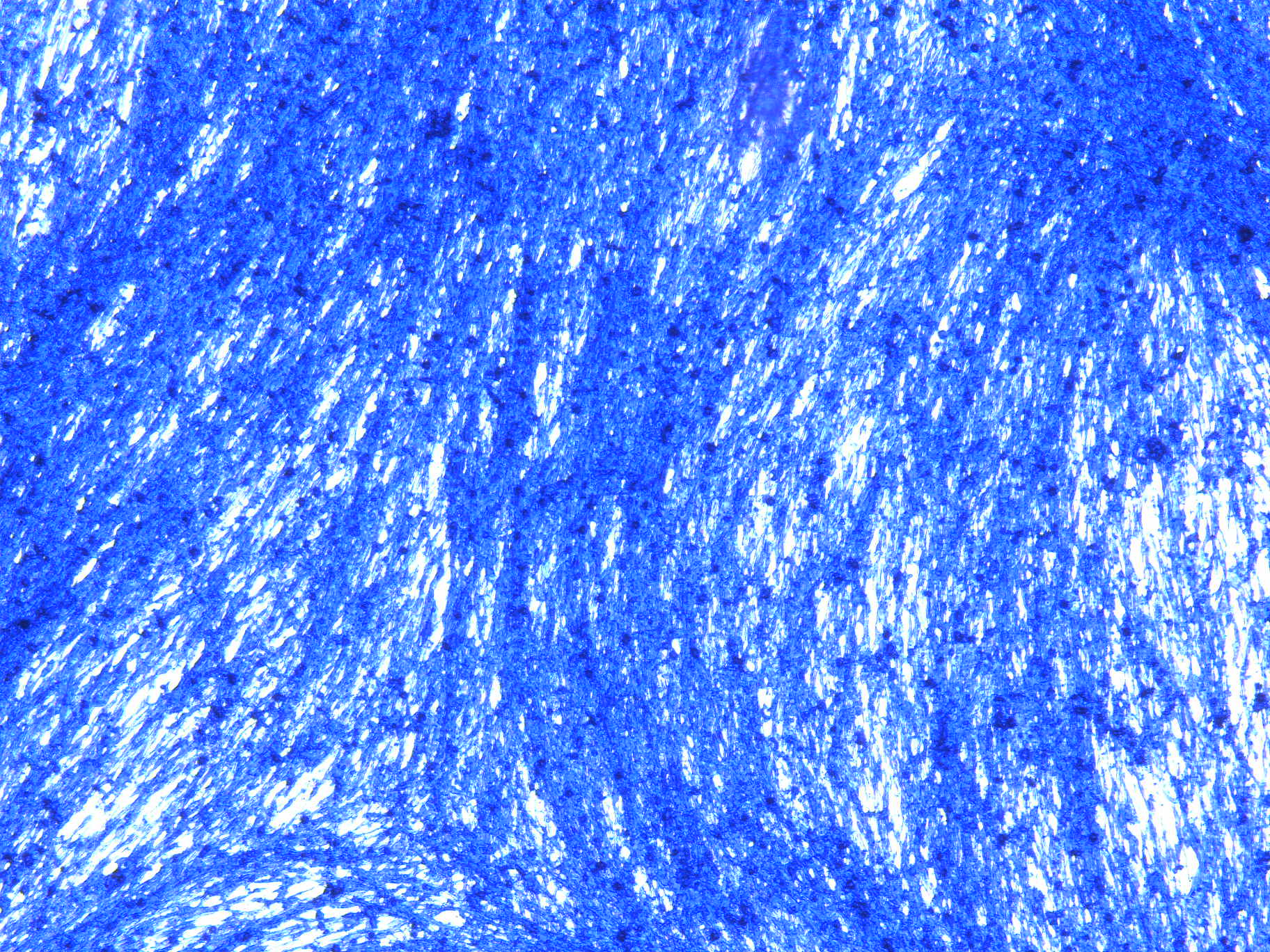

Supplement: Supplementary file 6 — Source data Fig. 4 [file 44321_2025_201_MOESM6_ESM.zip › Fig4/Fig4b CV/U118/mock/Biri-D9.JPG]

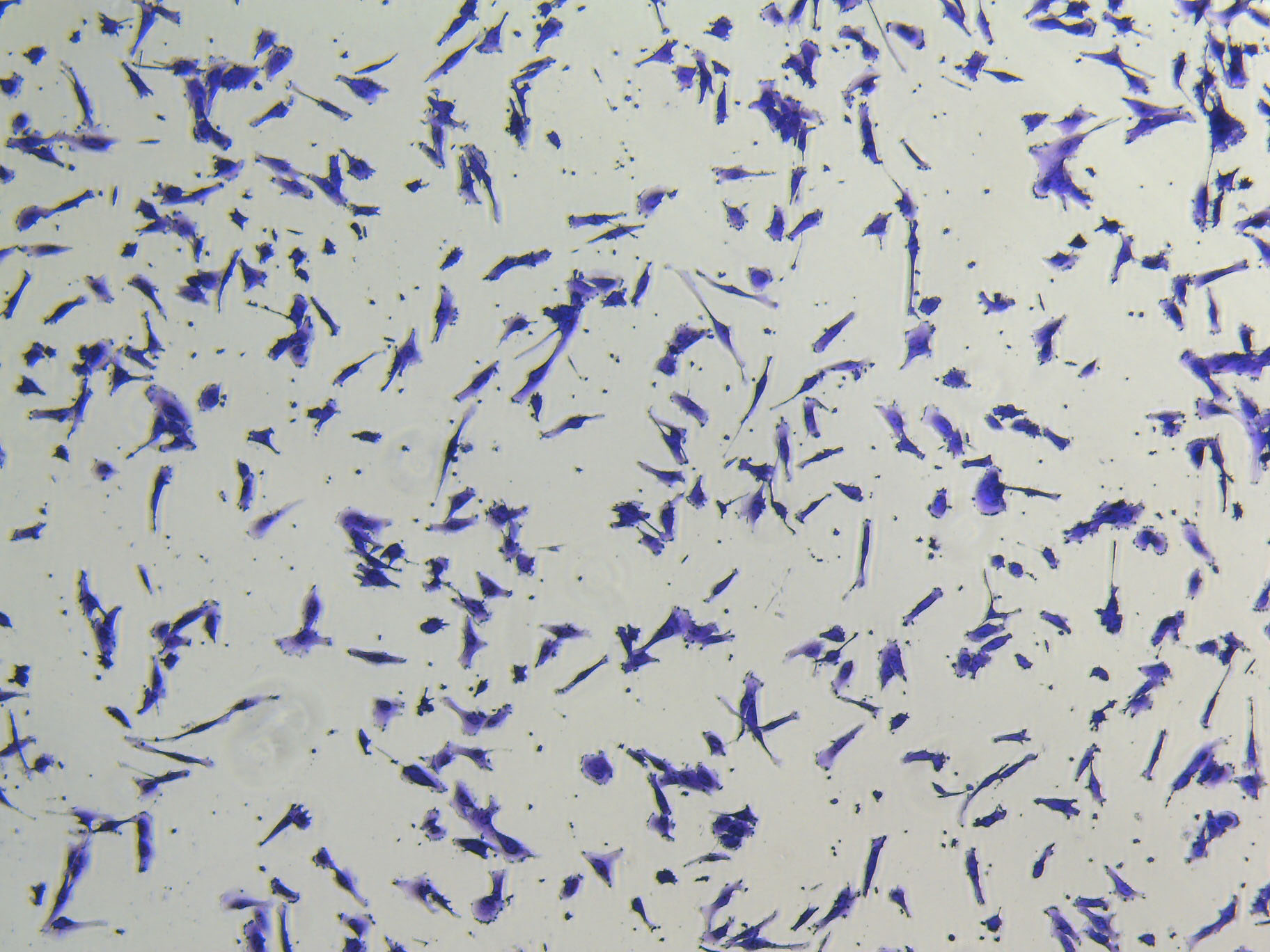

Supplement: Supplementary file 6 — Source data Fig. 4 [file 44321_2025_201_MOESM6_ESM.zip › Fig4/Fig4b CV/U118/mock/DMSO-D0.JPG]

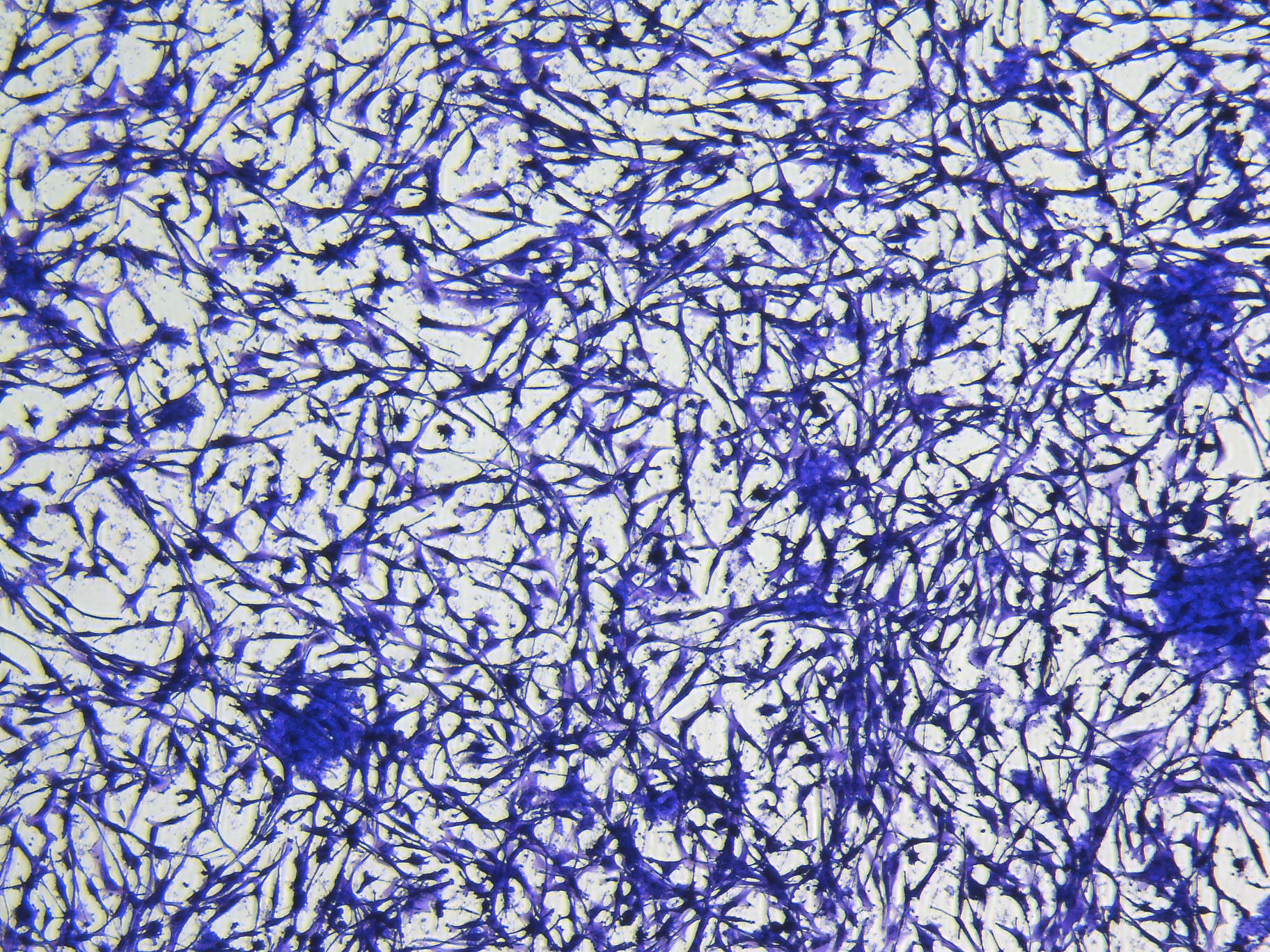

Supplement: Supplementary file 6 — Source data Fig. 4 [file 44321_2025_201_MOESM6_ESM.zip › Fig4/Fig4b CV/U118/mock/DMSO-D3.JPG]

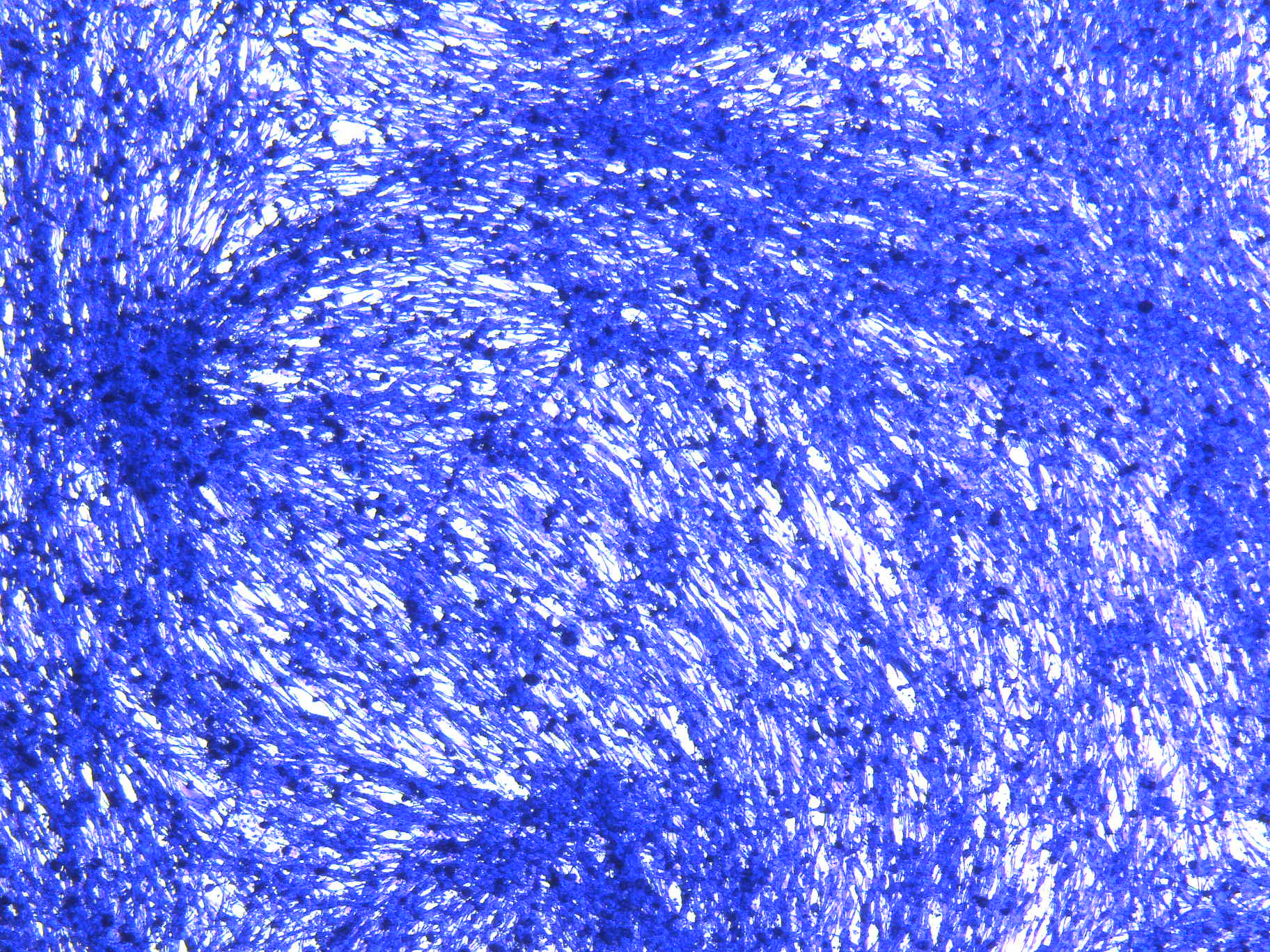

Supplement: Supplementary file 6 — Source data Fig. 4 [file 44321_2025_201_MOESM6_ESM.zip › Fig4/Fig4b CV/U118/mock/DMSO-D6.JPG]

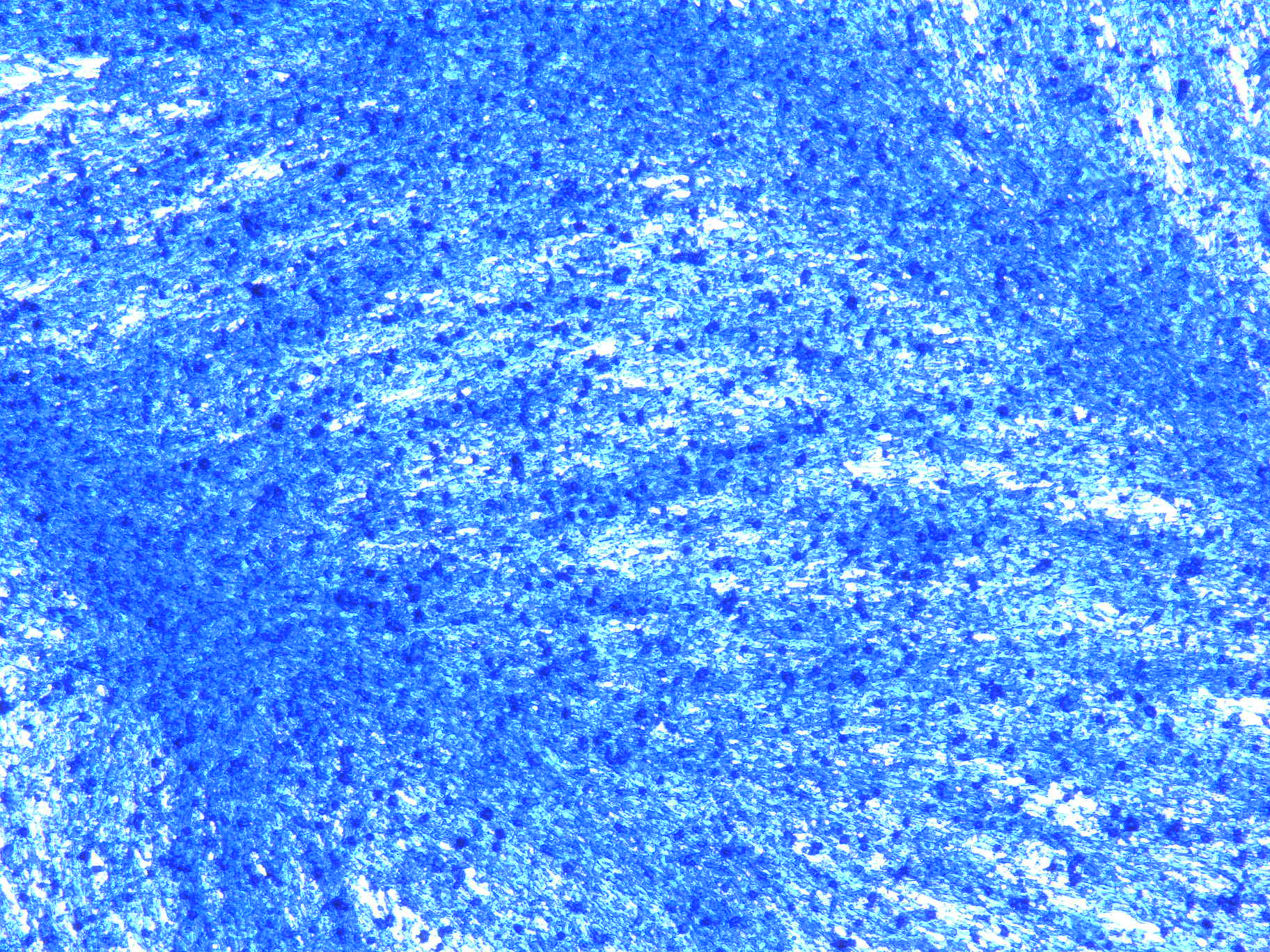

Supplement: Supplementary file 6 — Source data Fig. 4 [file 44321_2025_201_MOESM6_ESM.zip › Fig4/Fig4b CV/U118/mock/DMSO-D9.JPG]

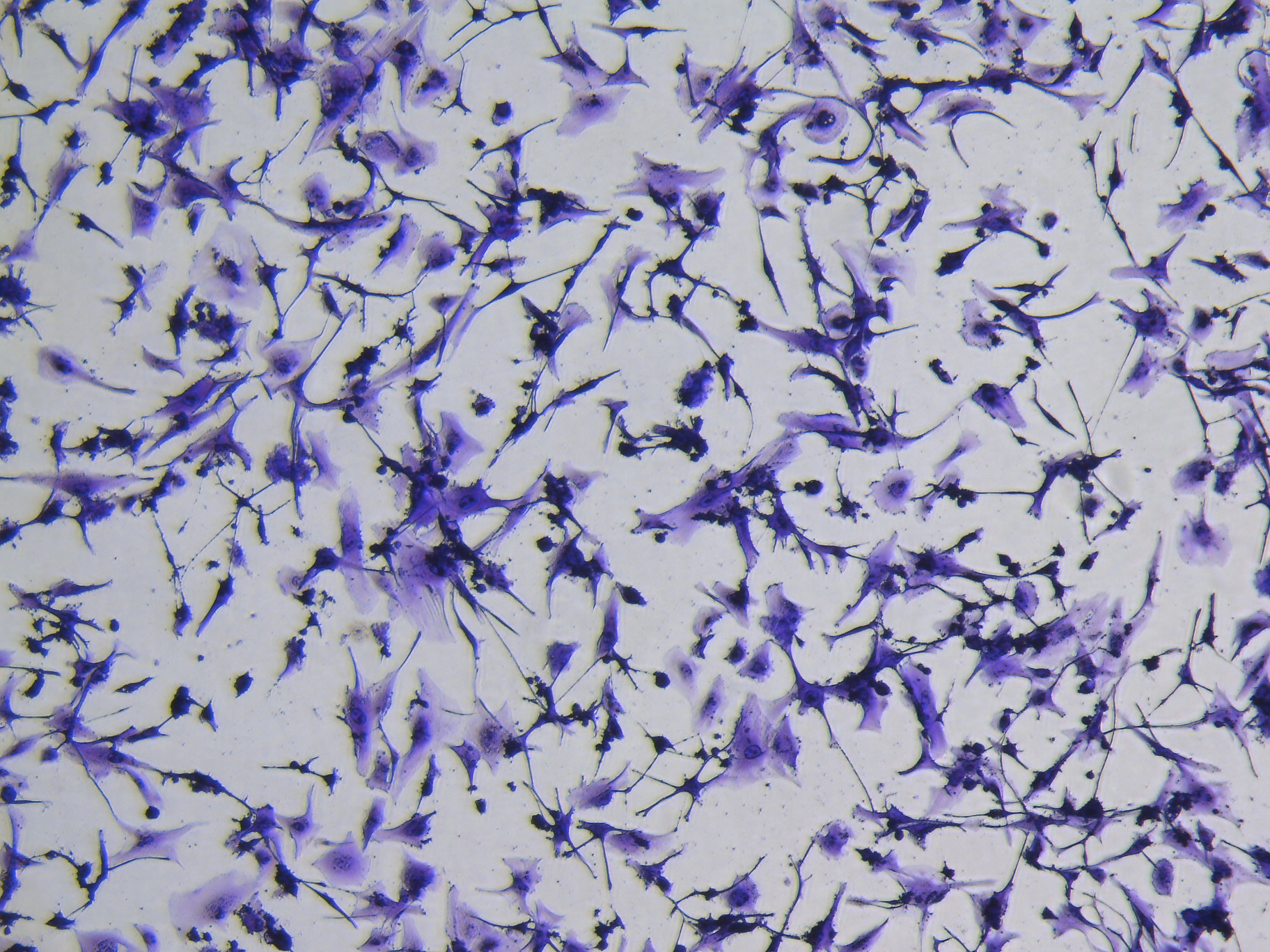

Supplement: Supplementary file 6 — Source data Fig. 4 [file 44321_2025_201_MOESM6_ESM.zip › Fig4/Fig4b CV/U87/IR/Biri-D0.JPG]

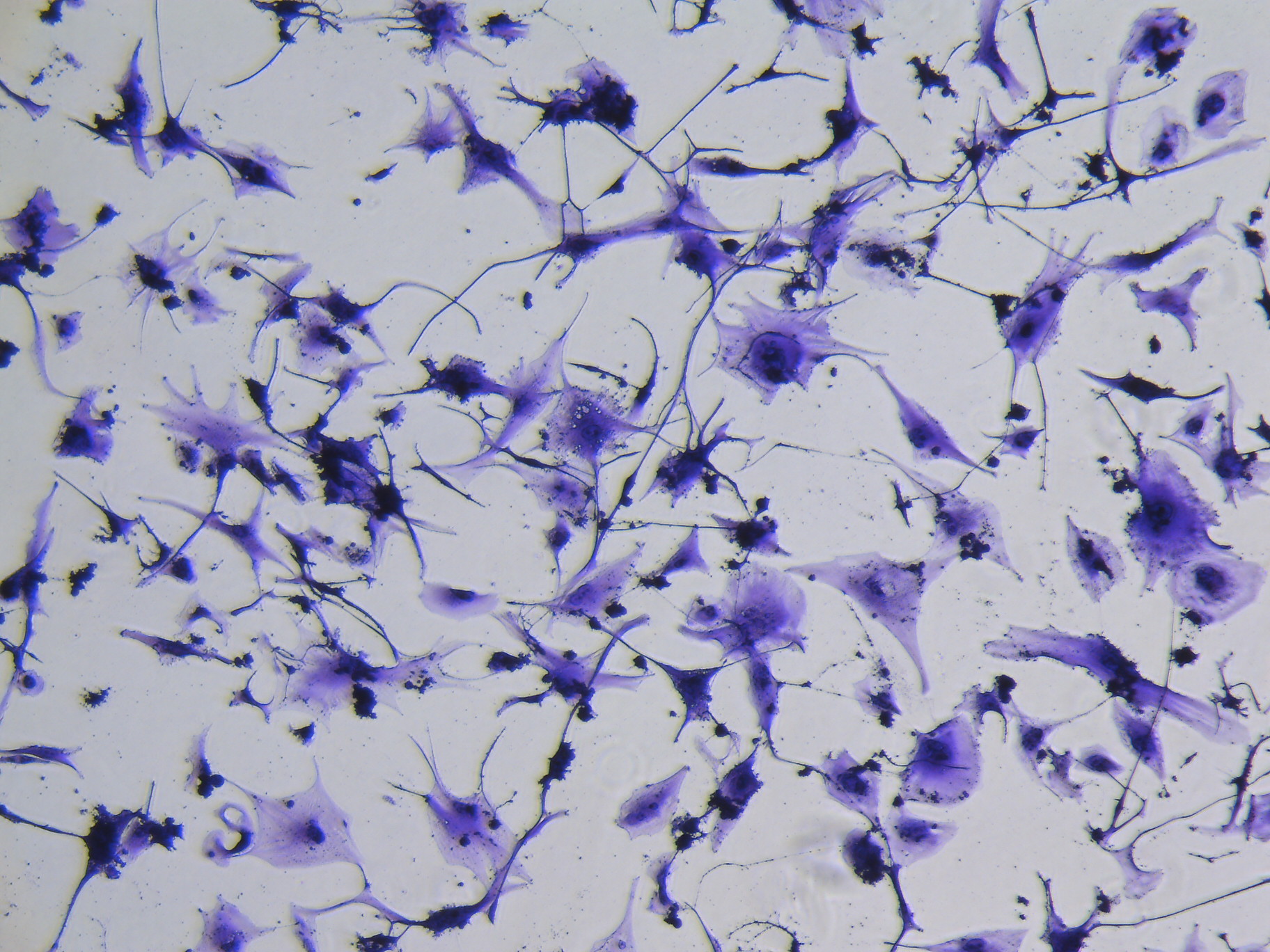

Supplement: Supplementary file 6 — Source data Fig. 4 [file 44321_2025_201_MOESM6_ESM.zip › Fig4/Fig4b CV/U87/IR/Biri-D3.JPG]

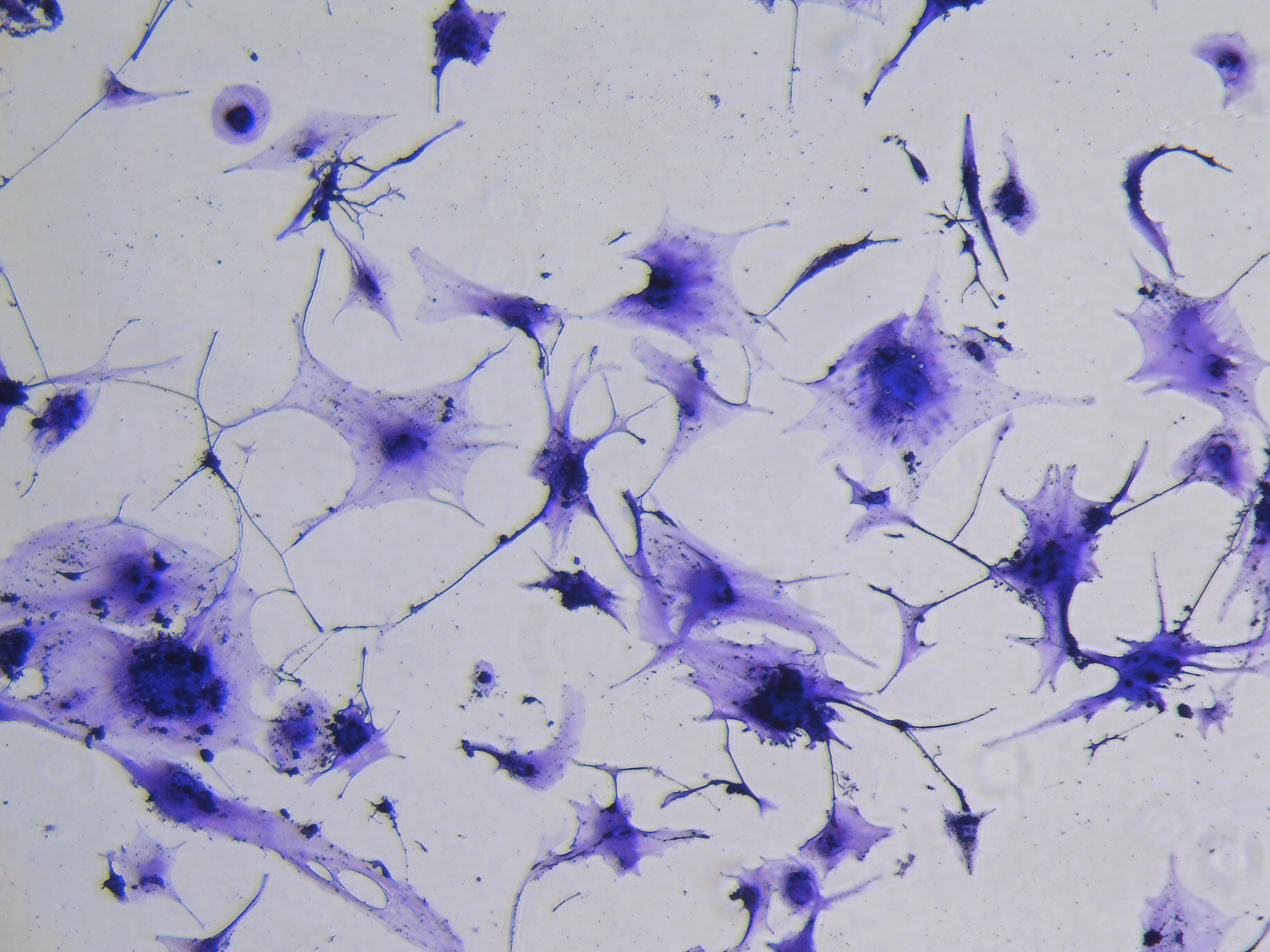

Supplement: Supplementary file 6 — Source data Fig. 4 [file 44321_2025_201_MOESM6_ESM.zip › Fig4/Fig4b CV/U87/IR/Biri-D6.JPG]

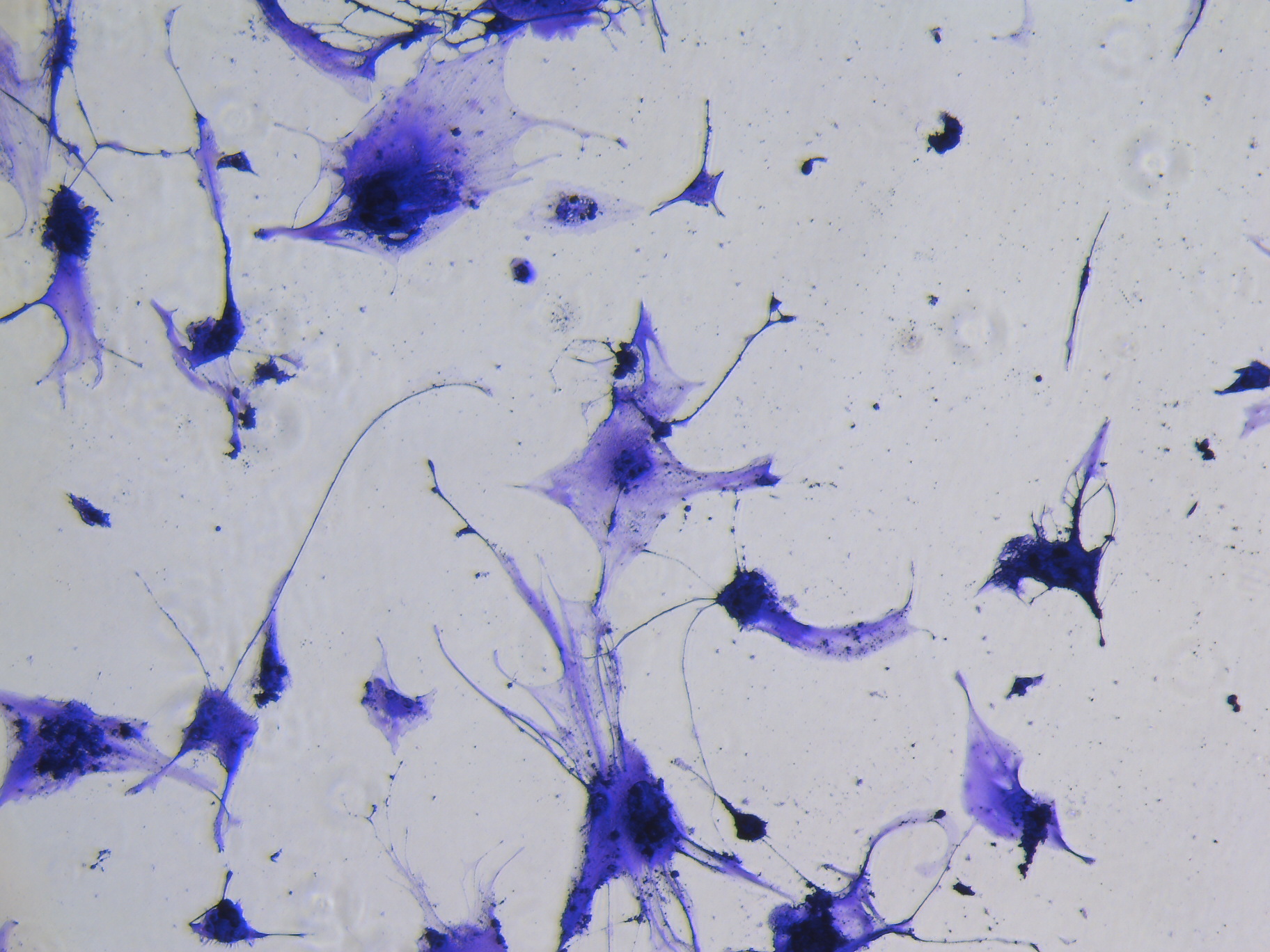

Supplement: Supplementary file 6 — Source data Fig. 4 [file 44321_2025_201_MOESM6_ESM.zip › Fig4/Fig4b CV/U87/IR/Biri-D9.JPG]

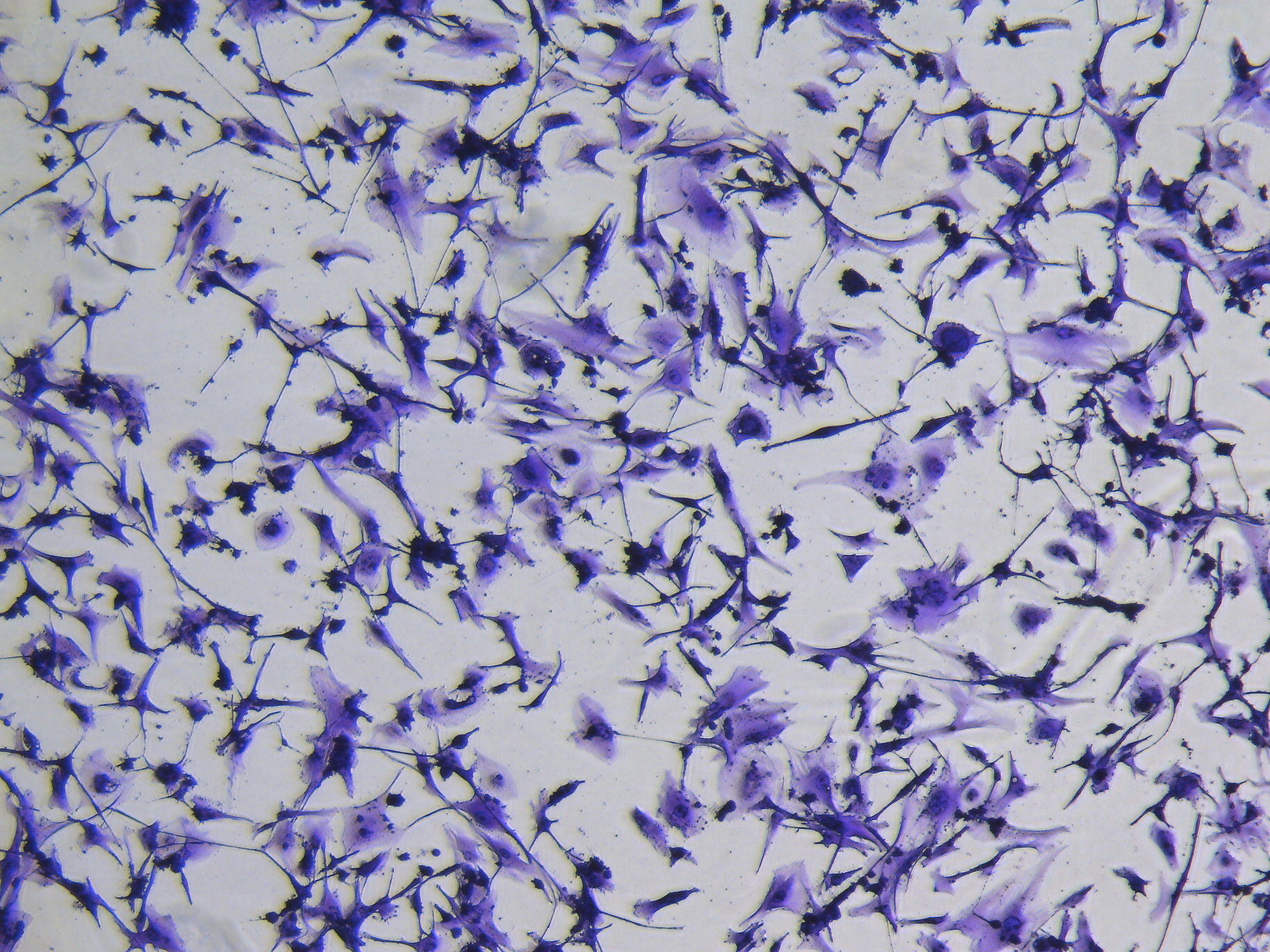

Supplement: Supplementary file 6 — Source data Fig. 4 [file 44321_2025_201_MOESM6_ESM.zip › Fig4/Fig4b CV/U87/IR/DMSO-D0.JPG]

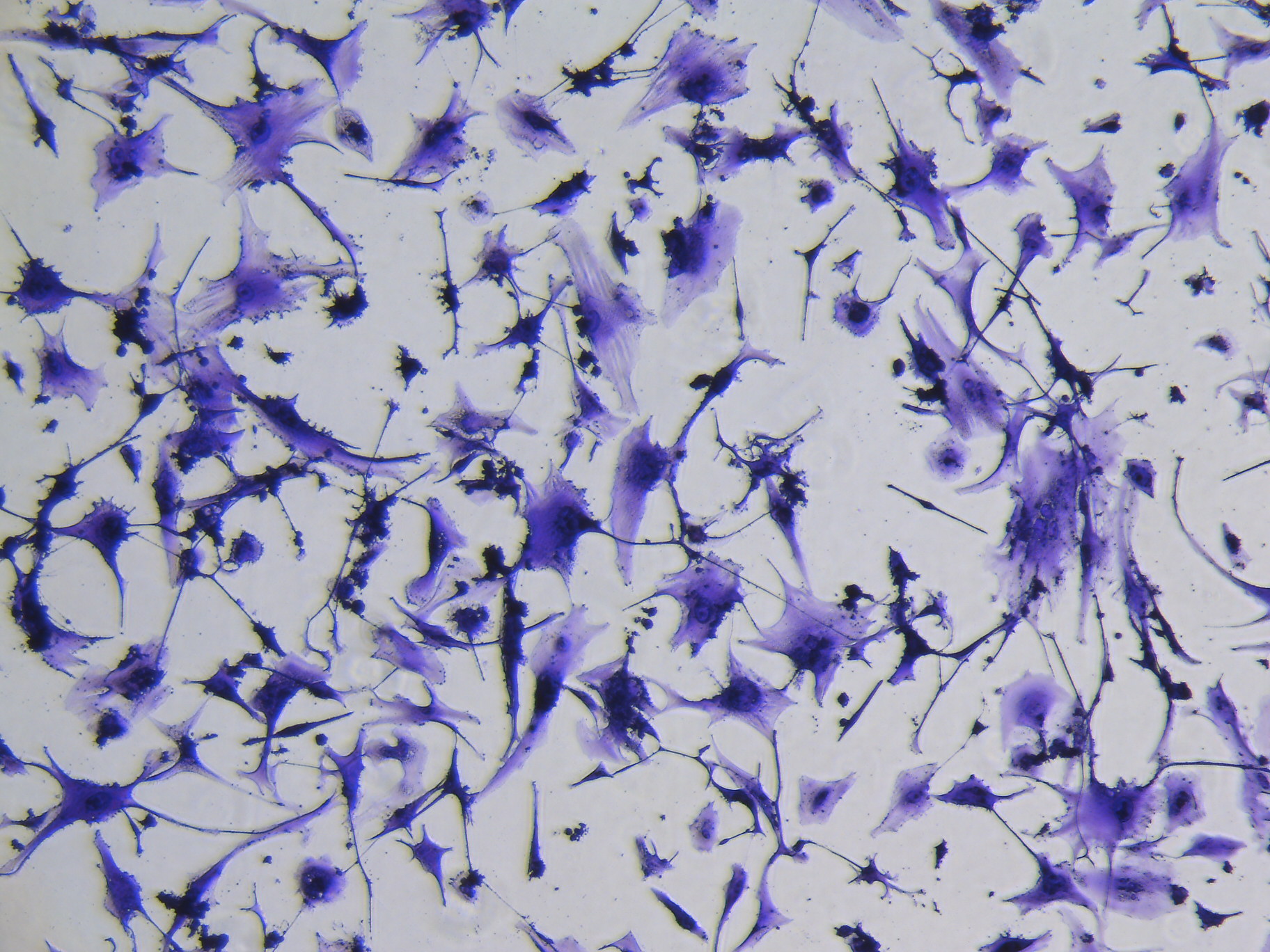

Supplement: Supplementary file 6 — Source data Fig. 4 [file 44321_2025_201_MOESM6_ESM.zip › Fig4/Fig4b CV/U87/IR/DMSO-D3.JPG]

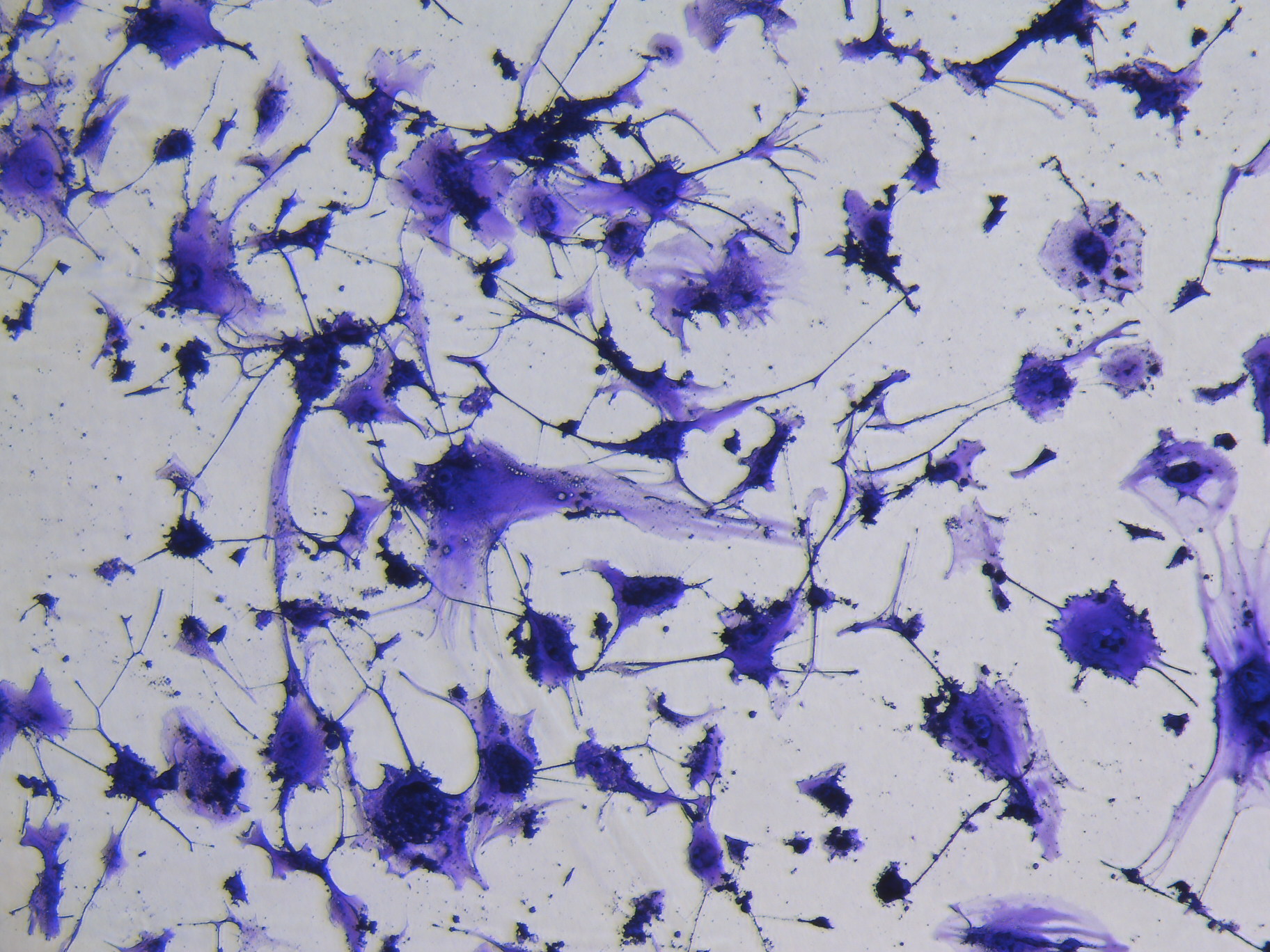

Supplement: Supplementary file 6 — Source data Fig. 4 [file 44321_2025_201_MOESM6_ESM.zip › Fig4/Fig4b CV/U87/IR/DMSO-D6.JPG]

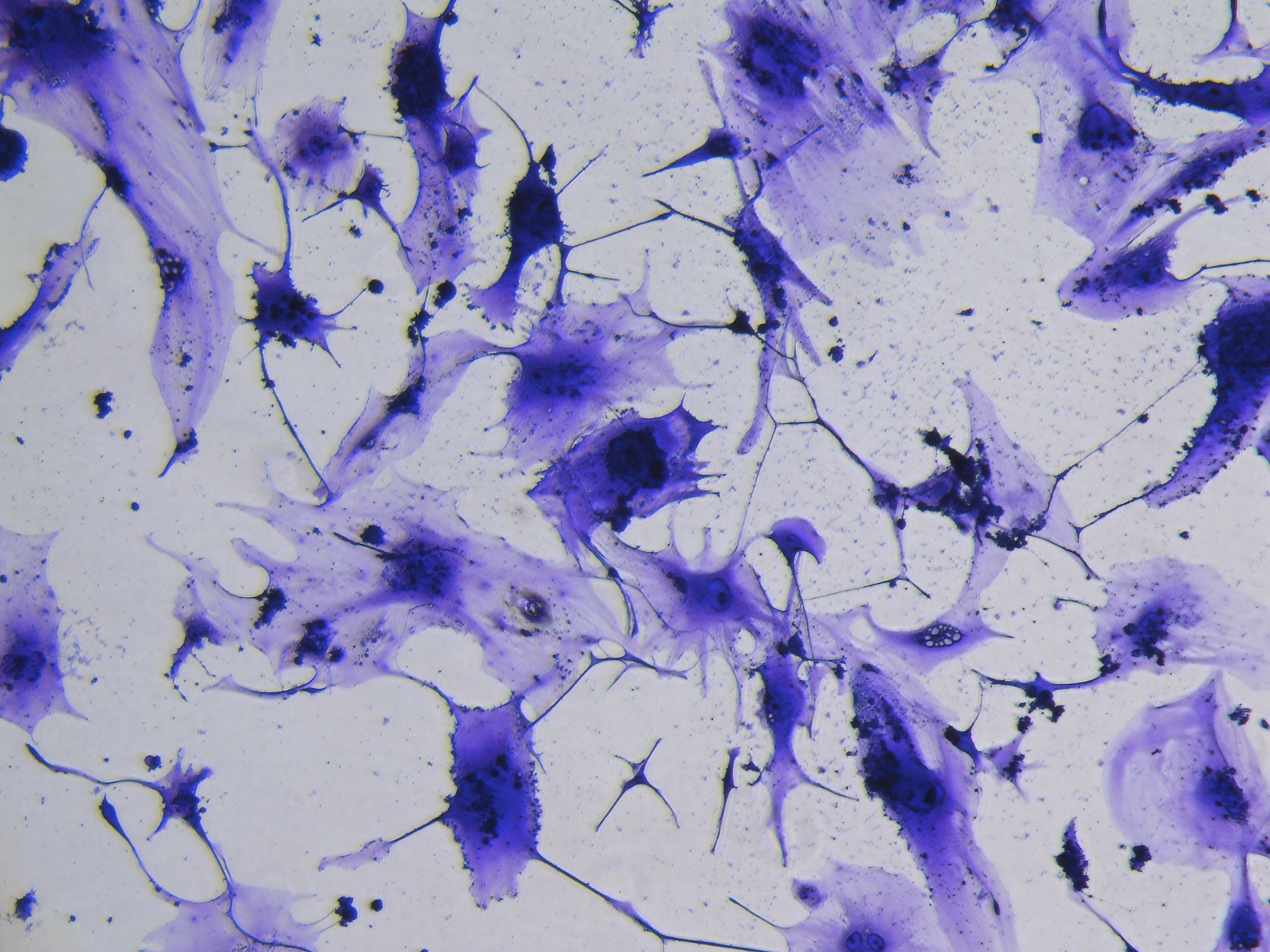

Supplement: Supplementary file 6 — Source data Fig. 4 [file 44321_2025_201_MOESM6_ESM.zip › Fig4/Fig4b CV/U87/IR/DMSO-D9.JPG]

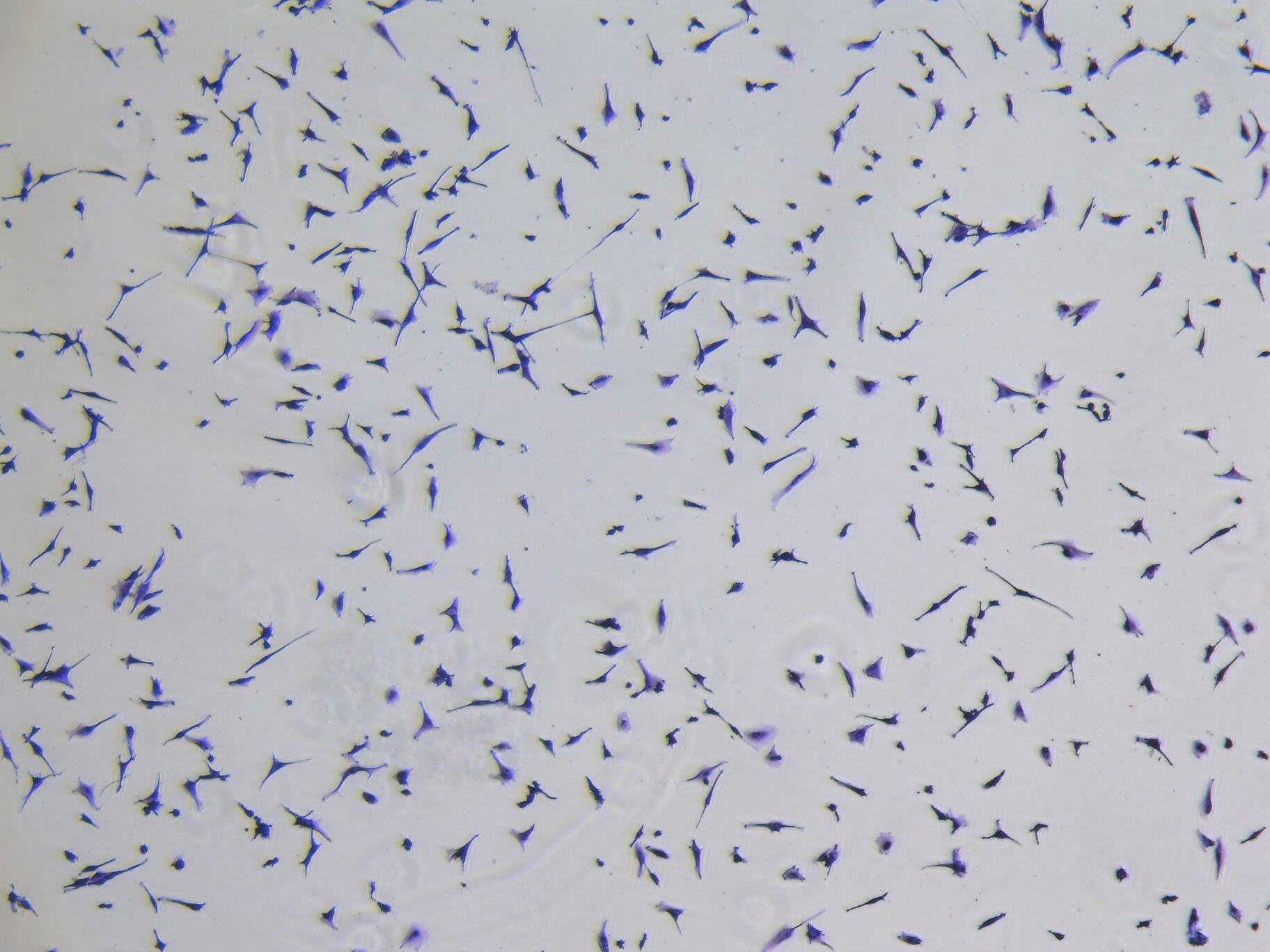

Supplement: Supplementary file 6 — Source data Fig. 4 [file 44321_2025_201_MOESM6_ESM.zip › Fig4/Fig4b CV/U87/mock/Biri-D0.JPG]

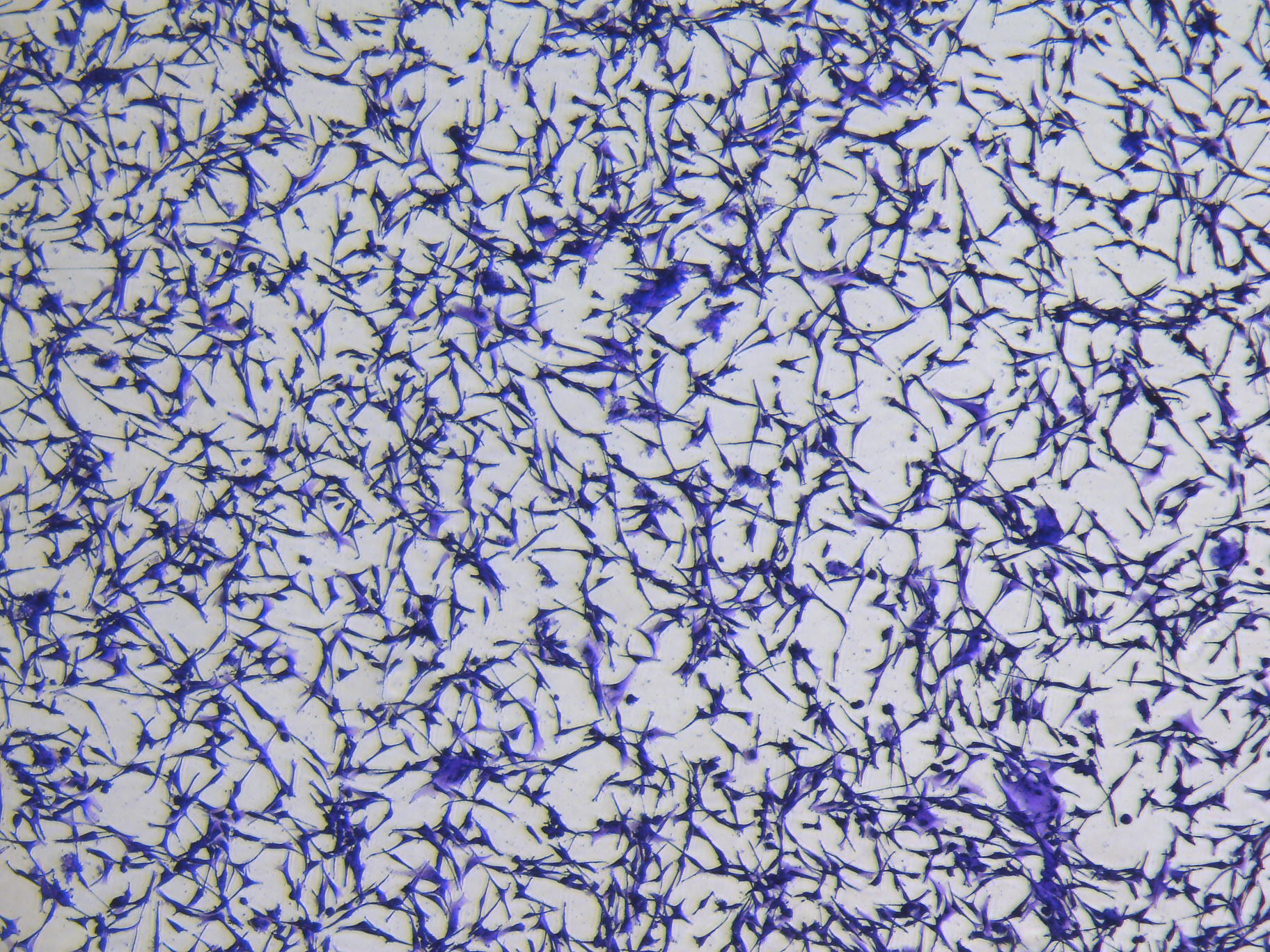

Supplement: Supplementary file 6 — Source data Fig. 4 [file 44321_2025_201_MOESM6_ESM.zip › Fig4/Fig4b CV/U87/mock/Biri-D3.JPG]

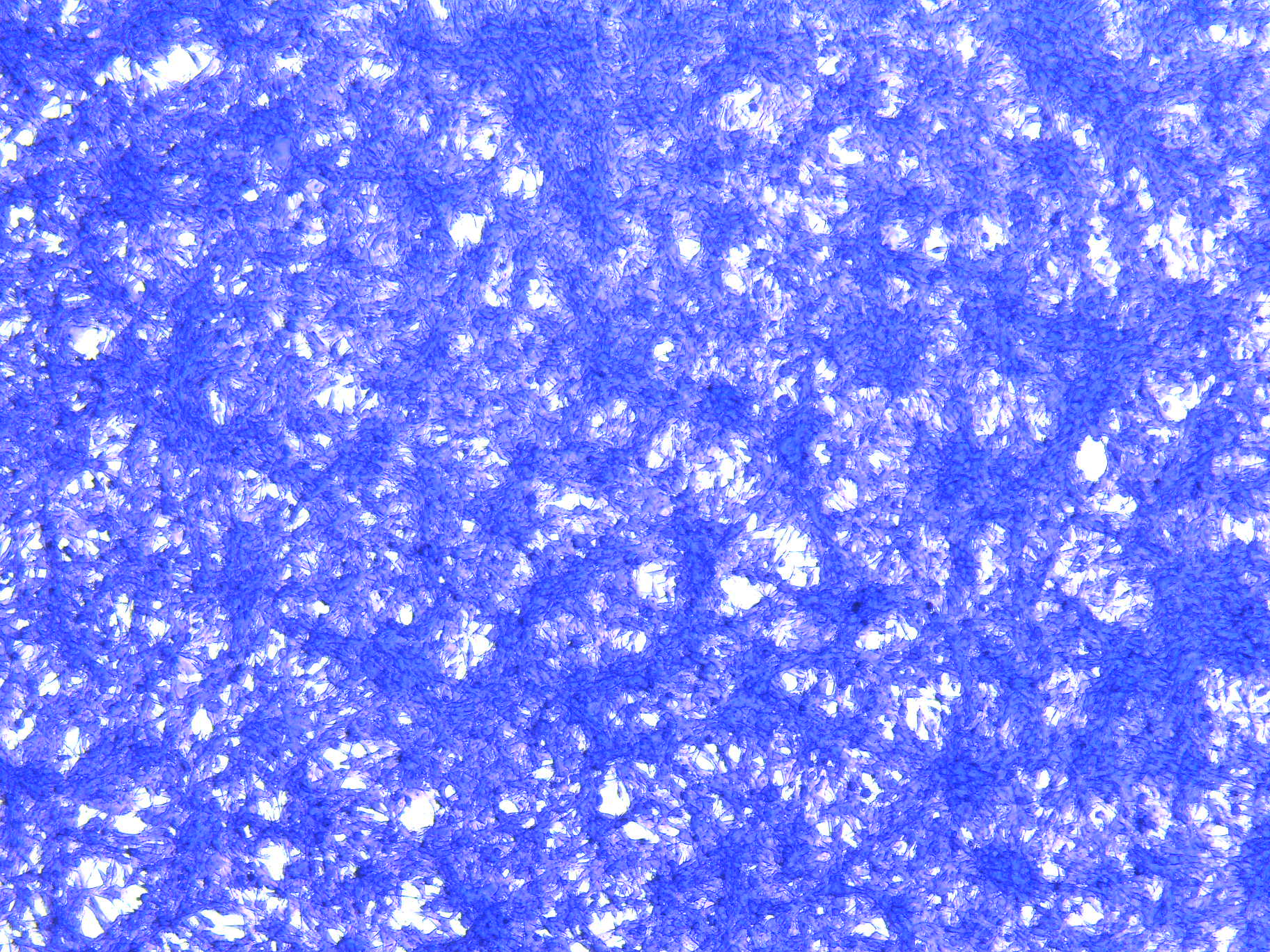

Supplement: Supplementary file 6 — Source data Fig. 4 [file 44321_2025_201_MOESM6_ESM.zip › Fig4/Fig4b CV/U87/mock/Biri-D6.JPG]

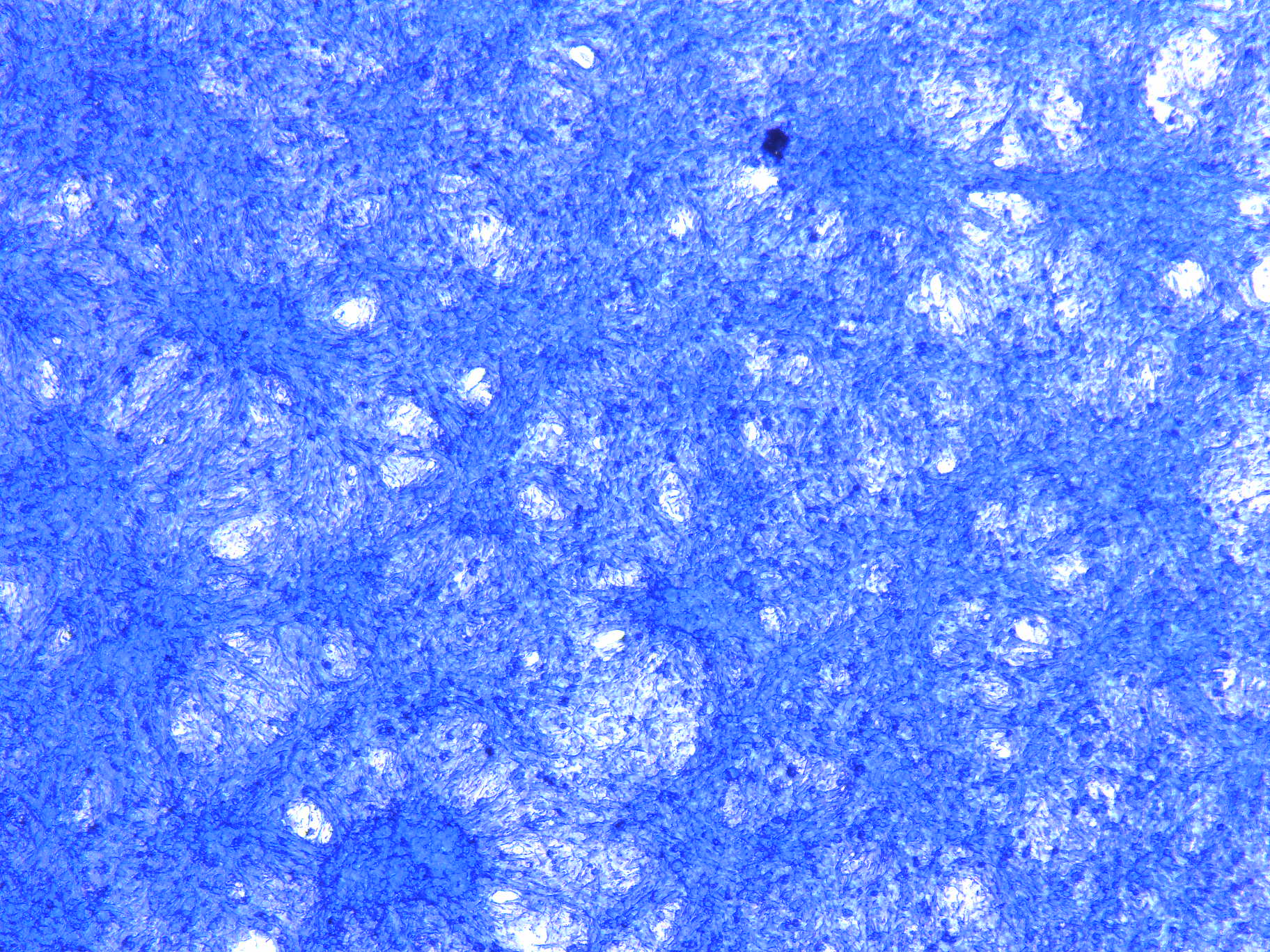

Supplement: Supplementary file 6 — Source data Fig. 4 [file 44321_2025_201_MOESM6_ESM.zip › Fig4/Fig4b CV/U87/mock/Biri-D9.JPG]

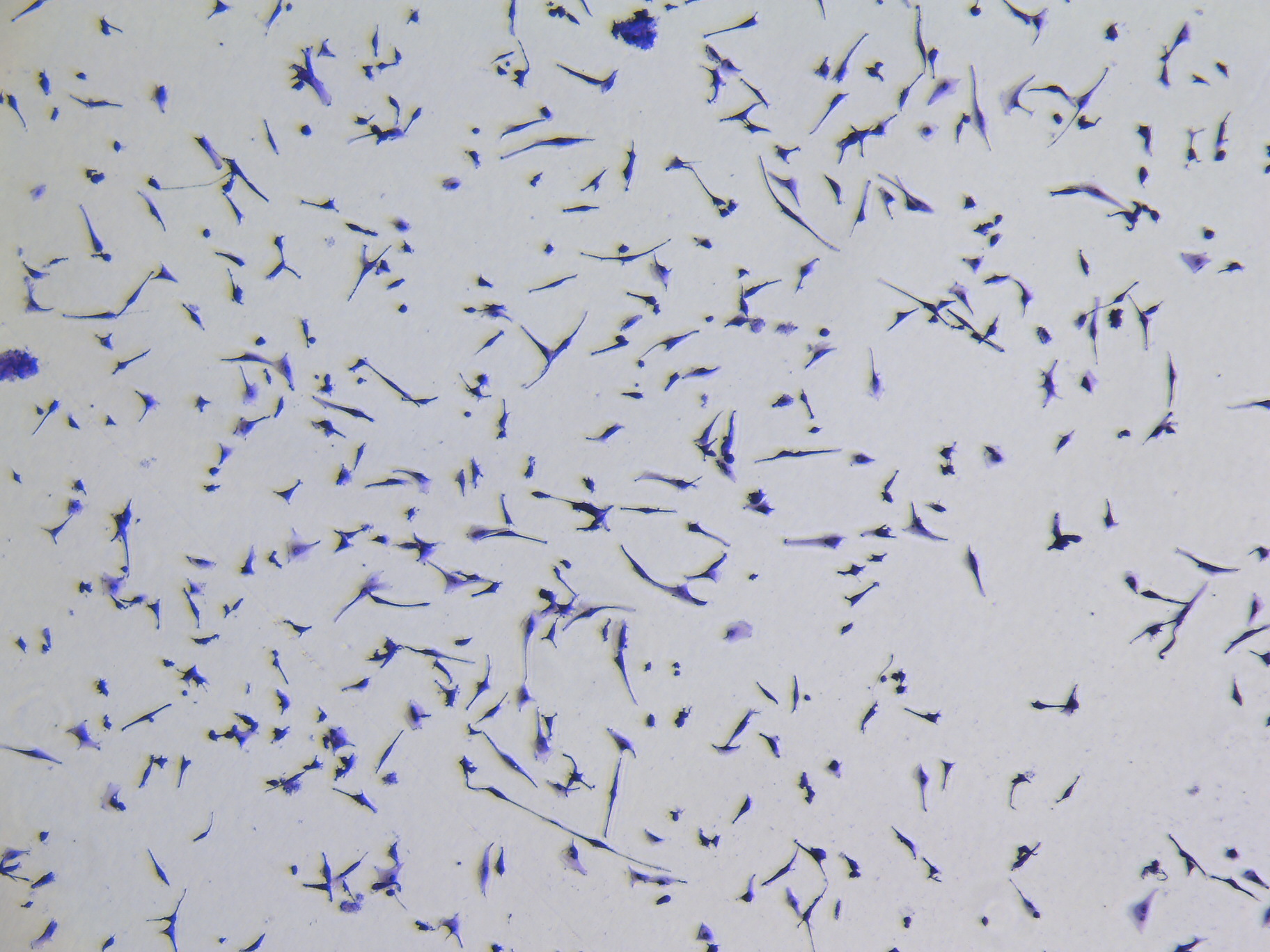

Supplement: Supplementary file 6 — Source data Fig. 4 [file 44321_2025_201_MOESM6_ESM.zip › Fig4/Fig4b CV/U87/mock/DMSO-D0.JPG]

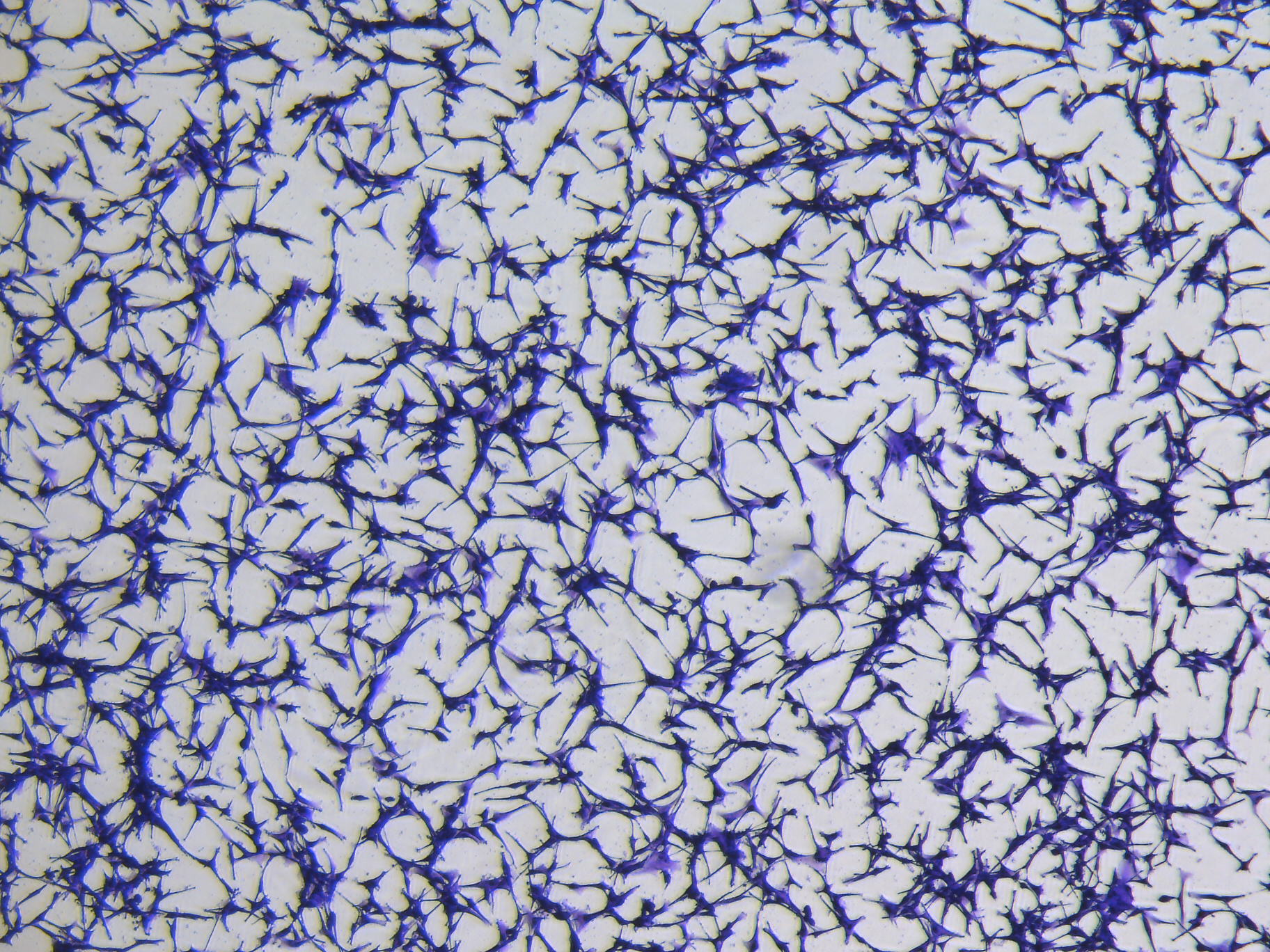

Supplement: Supplementary file 6 — Source data Fig. 4 [file 44321_2025_201_MOESM6_ESM.zip › Fig4/Fig4b CV/U87/mock/DMSO-D3.JPG]
