# Supplementary figures and images for: Targeting cIAP2 in a novel senolytic strategy prevents glioblastoma recurrence after radiotherapy (part 2 of 4)
Source: EMBO Mol Med. 2025 Feb 19;17(4):645–78. doi: 10.1038/s44321-025-00201-x (PMC11982261; doi:10.1038/s44321-025-00201-x)

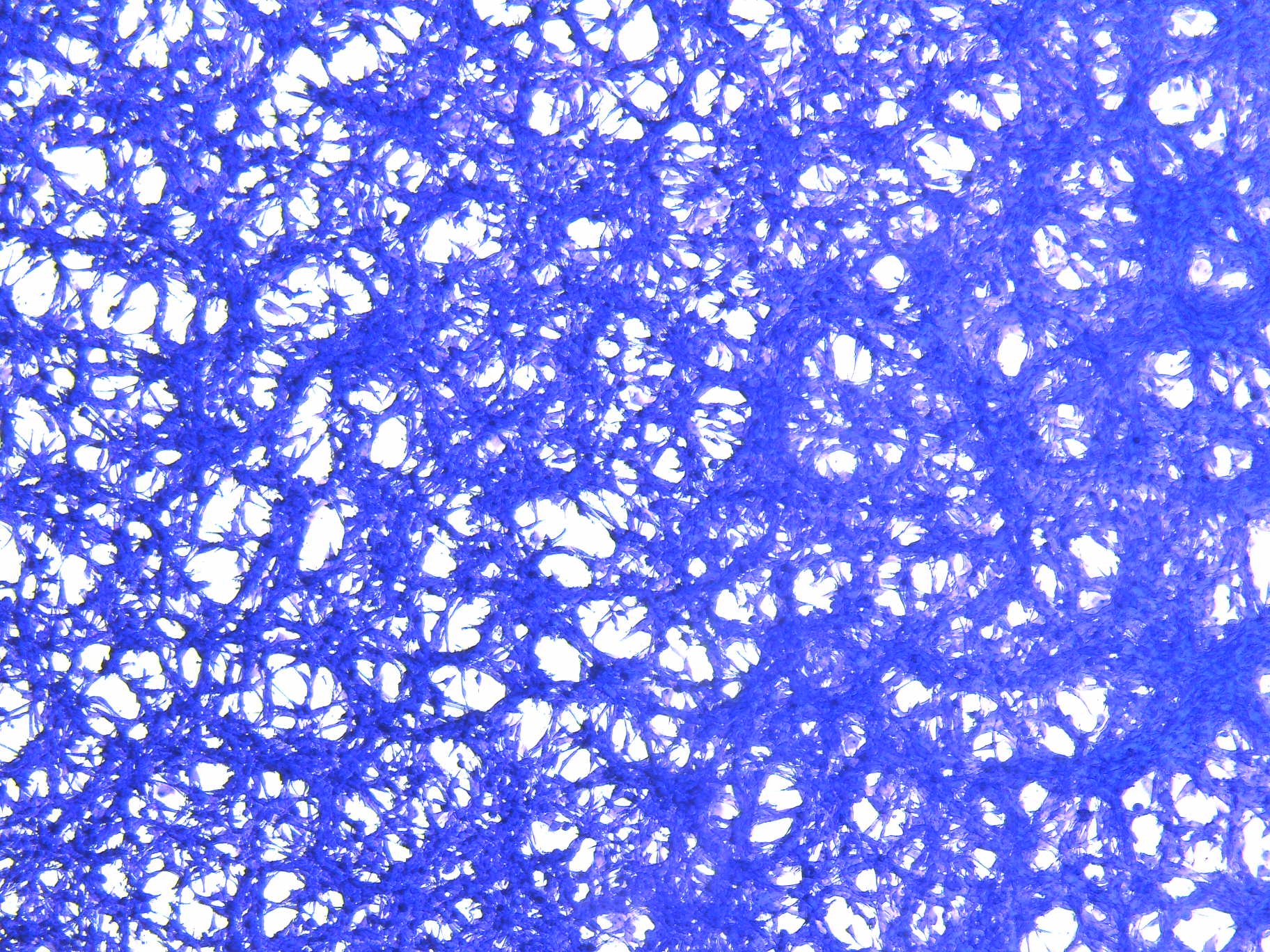

Supplement: Supplementary file 6 — Source data Fig. 4 [file 44321_2025_201_MOESM6_ESM.zip › Fig4/Fig4b CV/U87/mock/DMSO-D6.JPG]

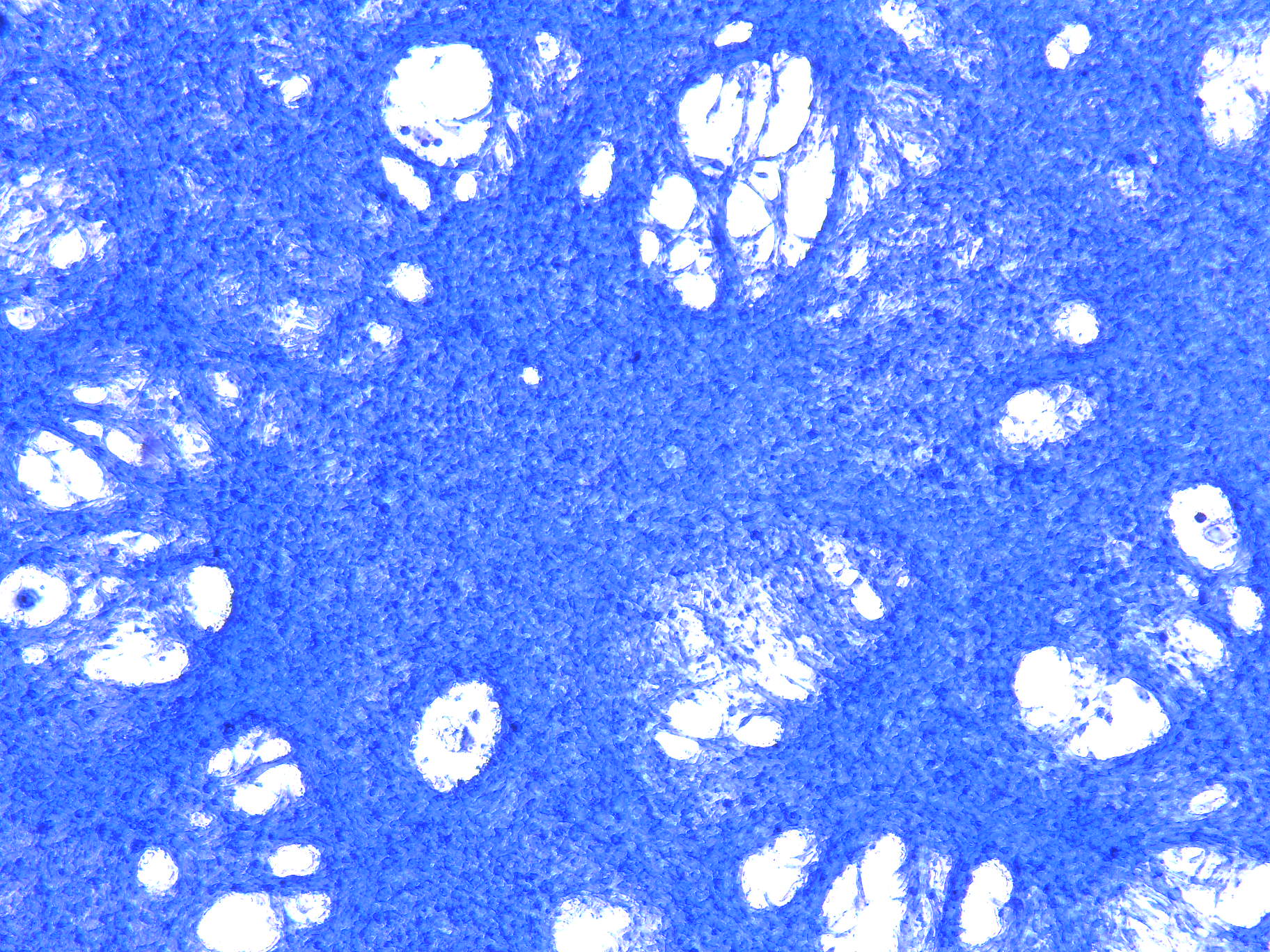

Supplement: Supplementary file 6 — Source data Fig. 4 [file 44321_2025_201_MOESM6_ESM.zip › Fig4/Fig4b CV/U87/mock/DMSO-D9.JPG]

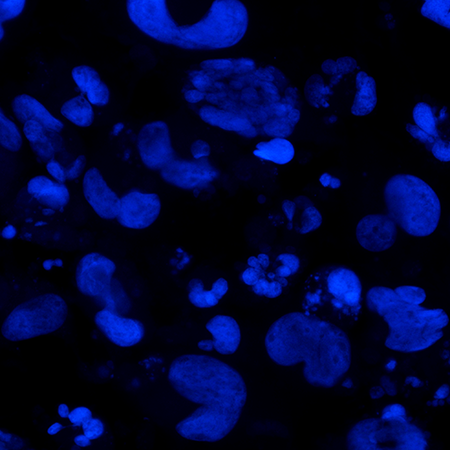

Supplement: Supplementary file 7 — Source data Fig. 5 [file 44321_2025_201_MOESM7_ESM.zip › Fig5/Fig5a bgal/12-IR-DAPI.tif]

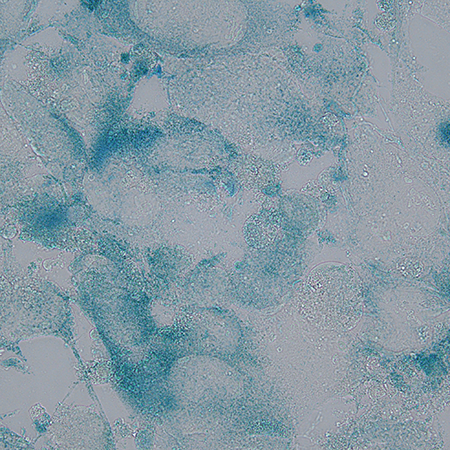

Supplement: Supplementary file 7 — Source data Fig. 5 [file 44321_2025_201_MOESM7_ESM.zip › Fig5/Fig5a bgal/12-IR.tif]

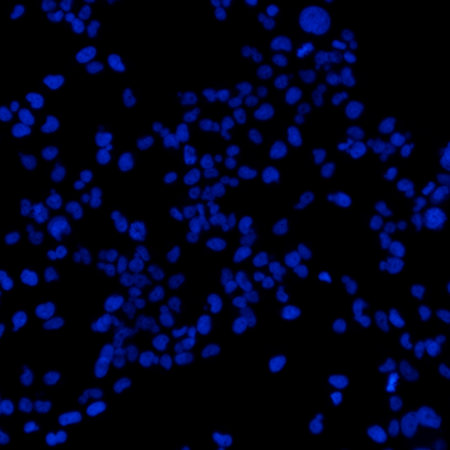

Supplement: Supplementary file 7 — Source data Fig. 5 [file 44321_2025_201_MOESM7_ESM.zip › Fig5/Fig5a bgal/12-mock-DAPI.tif]

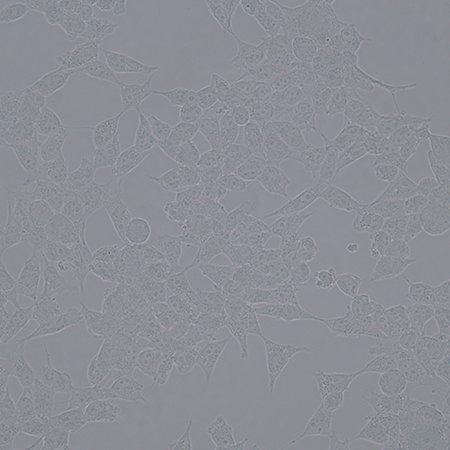

Supplement: Supplementary file 7 — Source data Fig. 5 [file 44321_2025_201_MOESM7_ESM.zip › Fig5/Fig5a bgal/12-mock.tif]

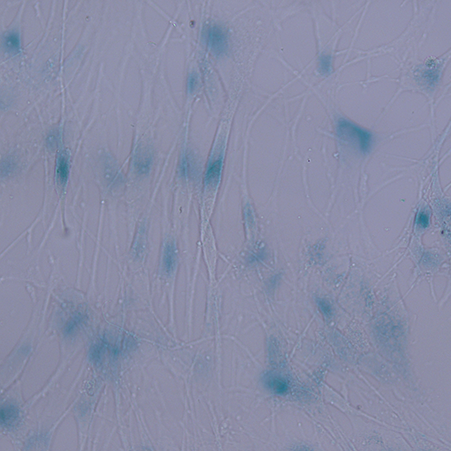

Supplement: Supplementary file 7 — Source data Fig. 5 [file 44321_2025_201_MOESM7_ESM.zip › Fig5/Fig5a bgal/123-IR-004c1.tif]

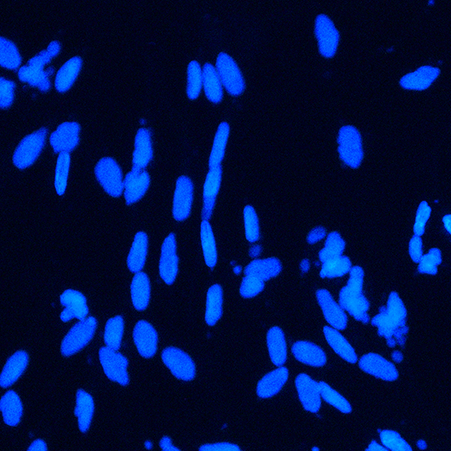

Supplement: Supplementary file 7 — Source data Fig. 5 [file 44321_2025_201_MOESM7_ESM.zip › Fig5/Fig5a bgal/123-IR-004c2.tif]

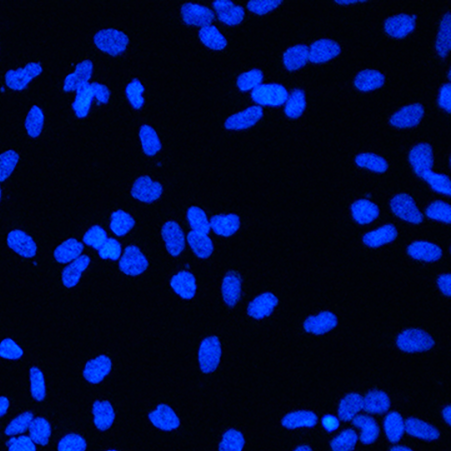

Supplement: Supplementary file 7 — Source data Fig. 5 [file 44321_2025_201_MOESM7_ESM.zip › Fig5/Fig5a bgal/123-mock-003-c2.tif]

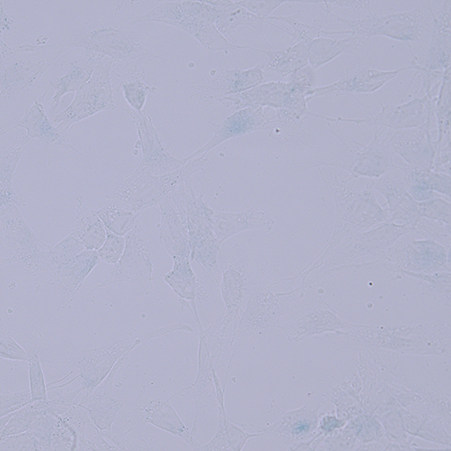

Supplement: Supplementary file 7 — Source data Fig. 5 [file 44321_2025_201_MOESM7_ESM.zip › Fig5/Fig5a bgal/123-mock-003c1.tif]

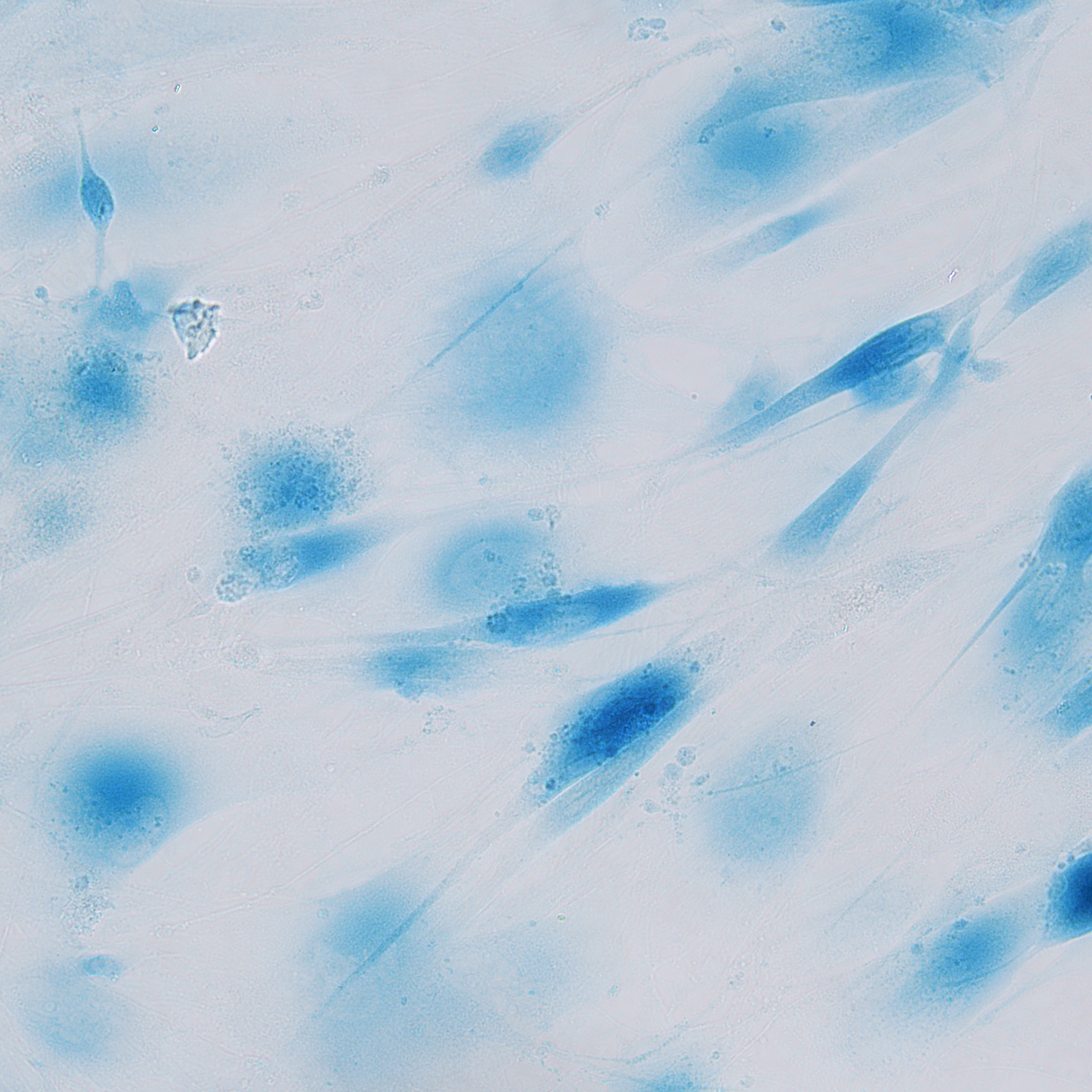

Supplement: Supplementary file 7 — Source data Fig. 5 [file 44321_2025_201_MOESM7_ESM.zip › Fig5/Fig5a bgal/148-1-IR-c1.tif]

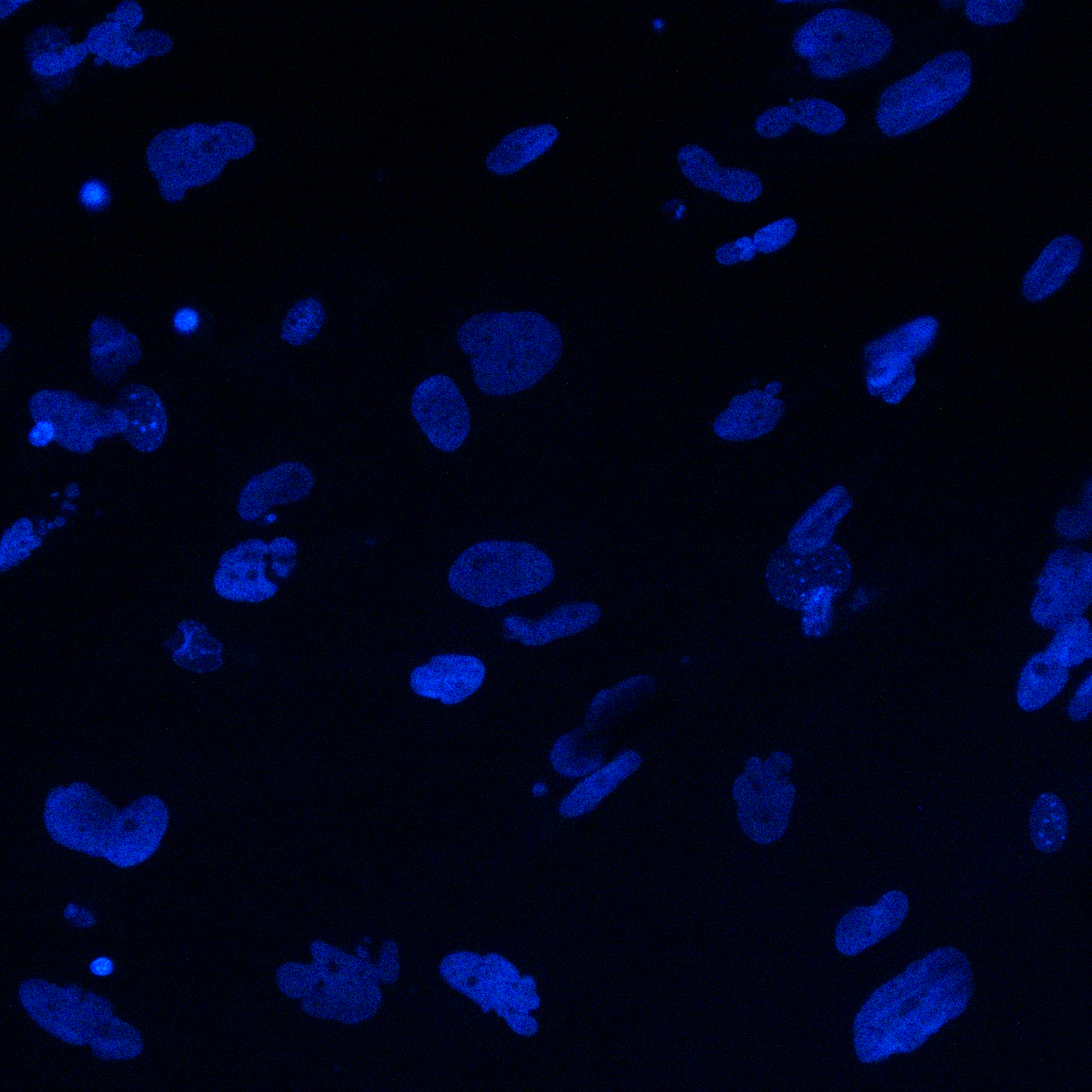

Supplement: Supplementary file 7 — Source data Fig. 5 [file 44321_2025_201_MOESM7_ESM.zip › Fig5/Fig5a bgal/148-1-IR-c2.tif]

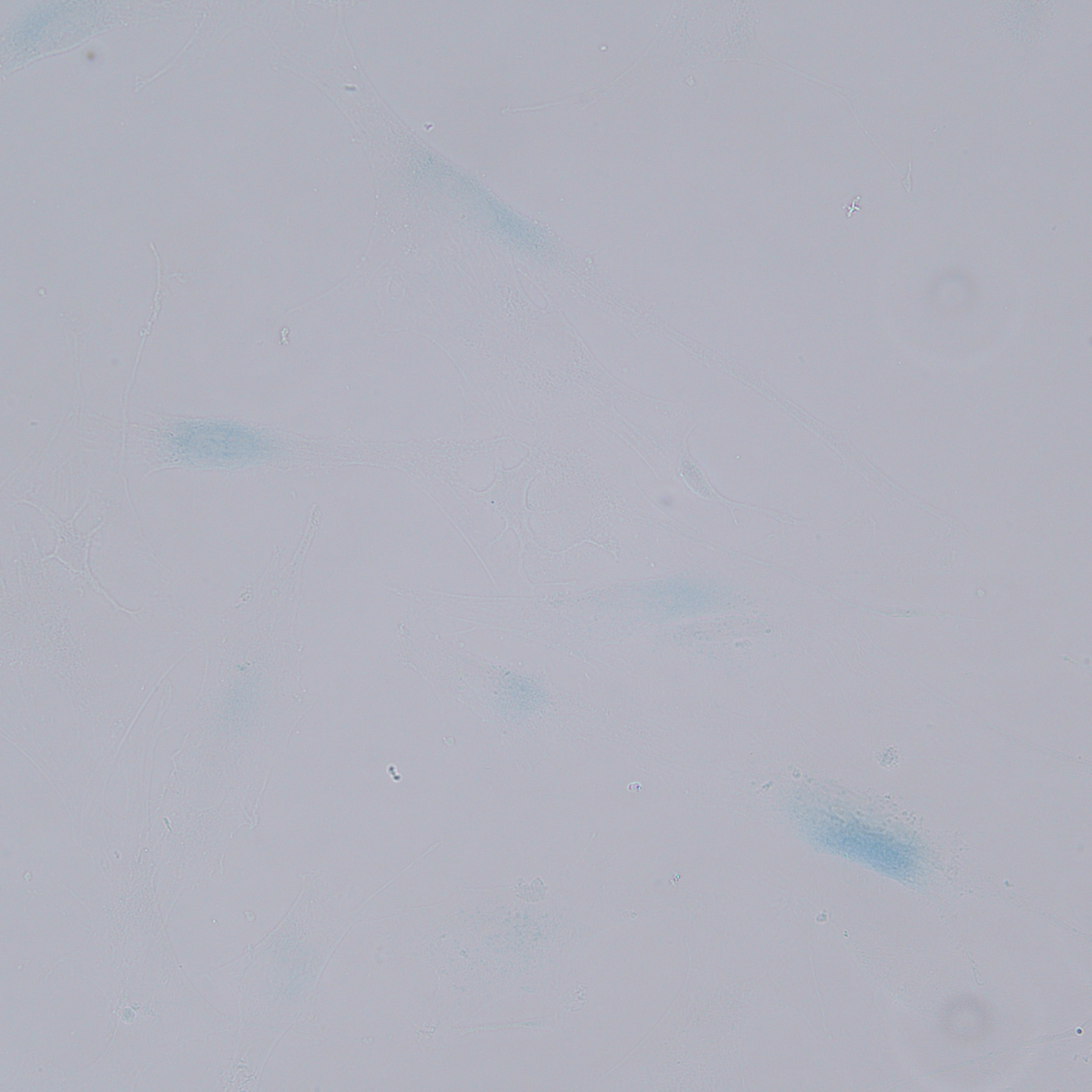

Supplement: Supplementary file 7 — Source data Fig. 5 [file 44321_2025_201_MOESM7_ESM.zip › Fig5/Fig5a bgal/148-mock-003c1.tif]

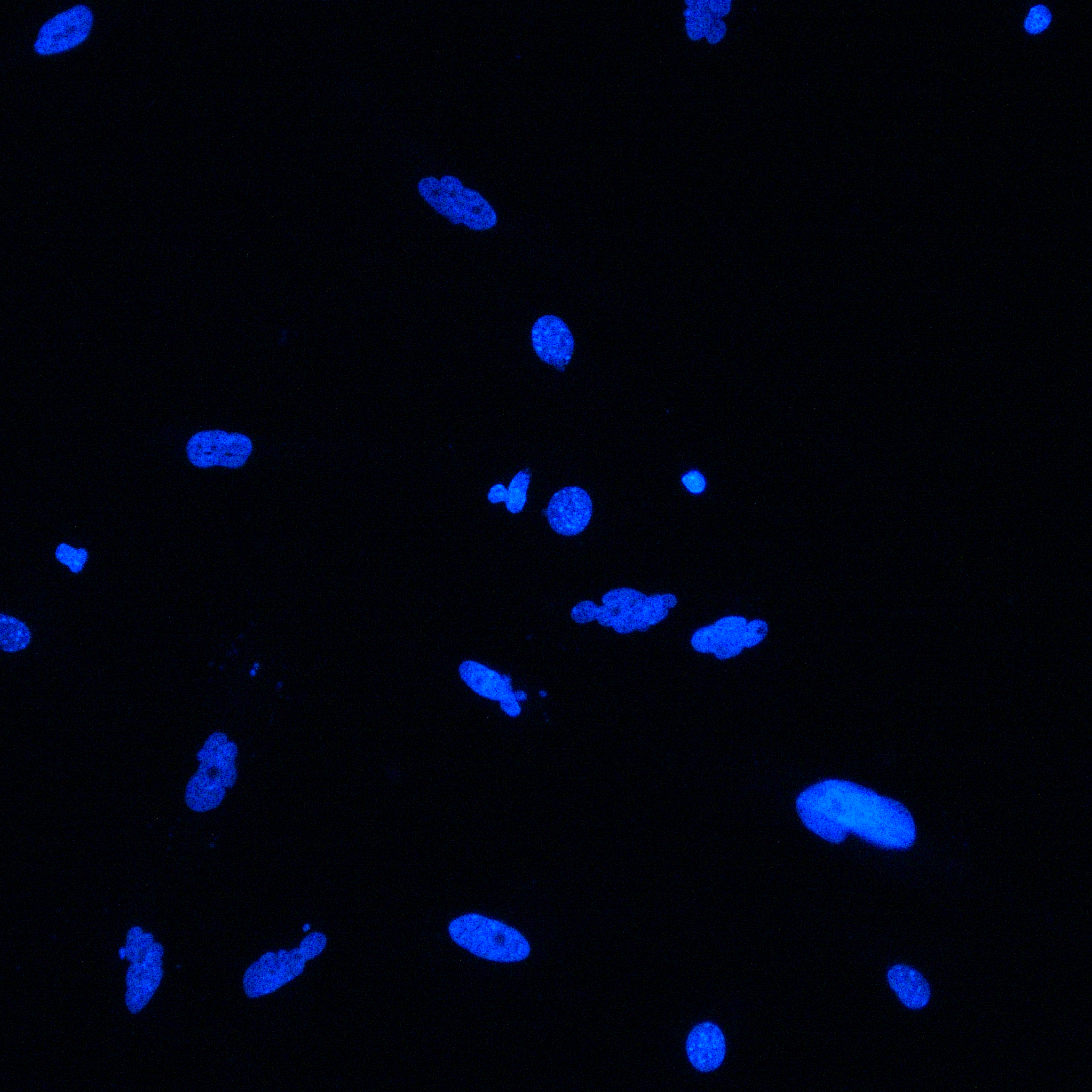

Supplement: Supplementary file 7 — Source data Fig. 5 [file 44321_2025_201_MOESM7_ESM.zip › Fig5/Fig5a bgal/148-mock-003c2.tif]

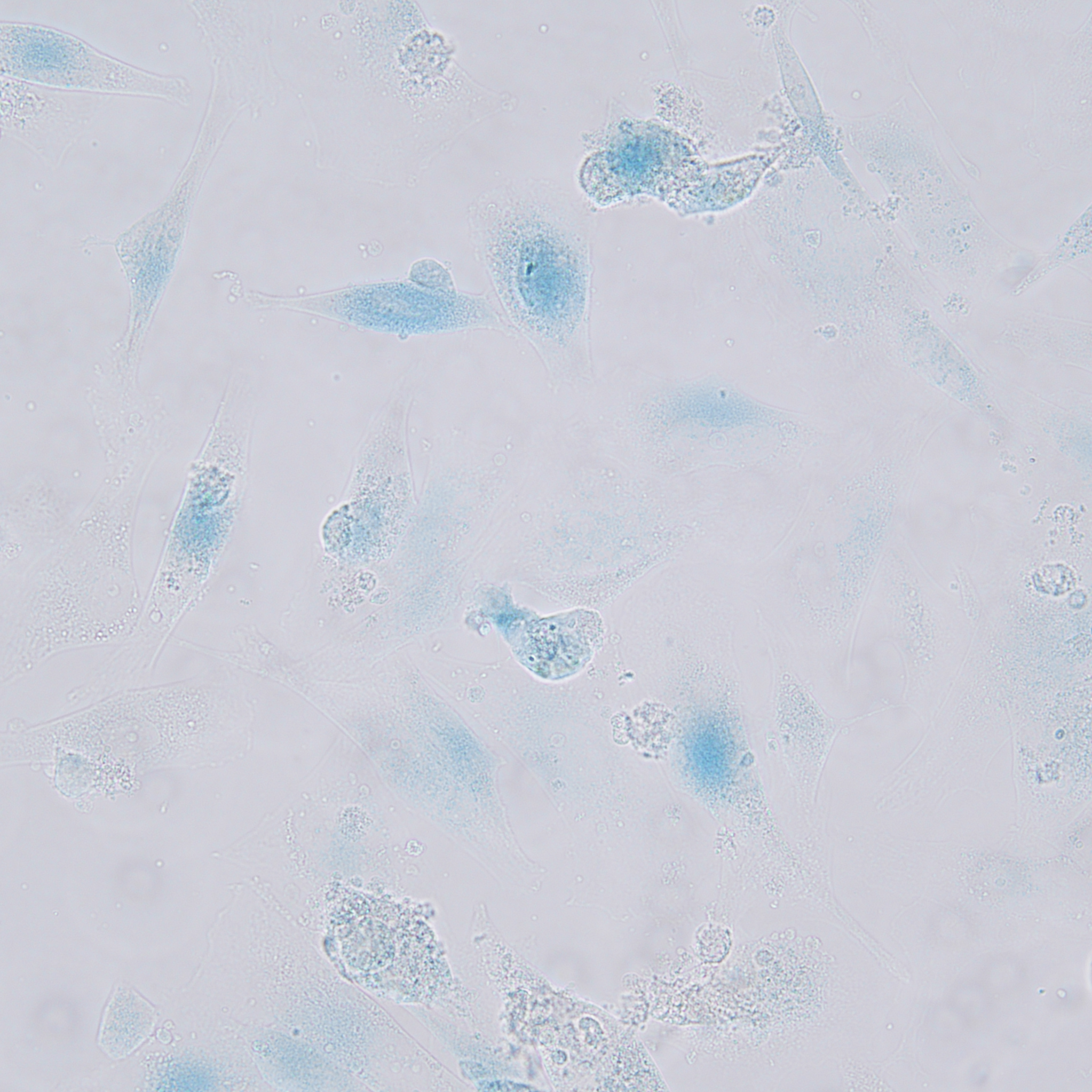

Supplement: Supplementary file 7 — Source data Fig. 5 [file 44321_2025_201_MOESM7_ESM.zip › Fig5/Fig5a bgal/245-3-IR-002c1.tif]

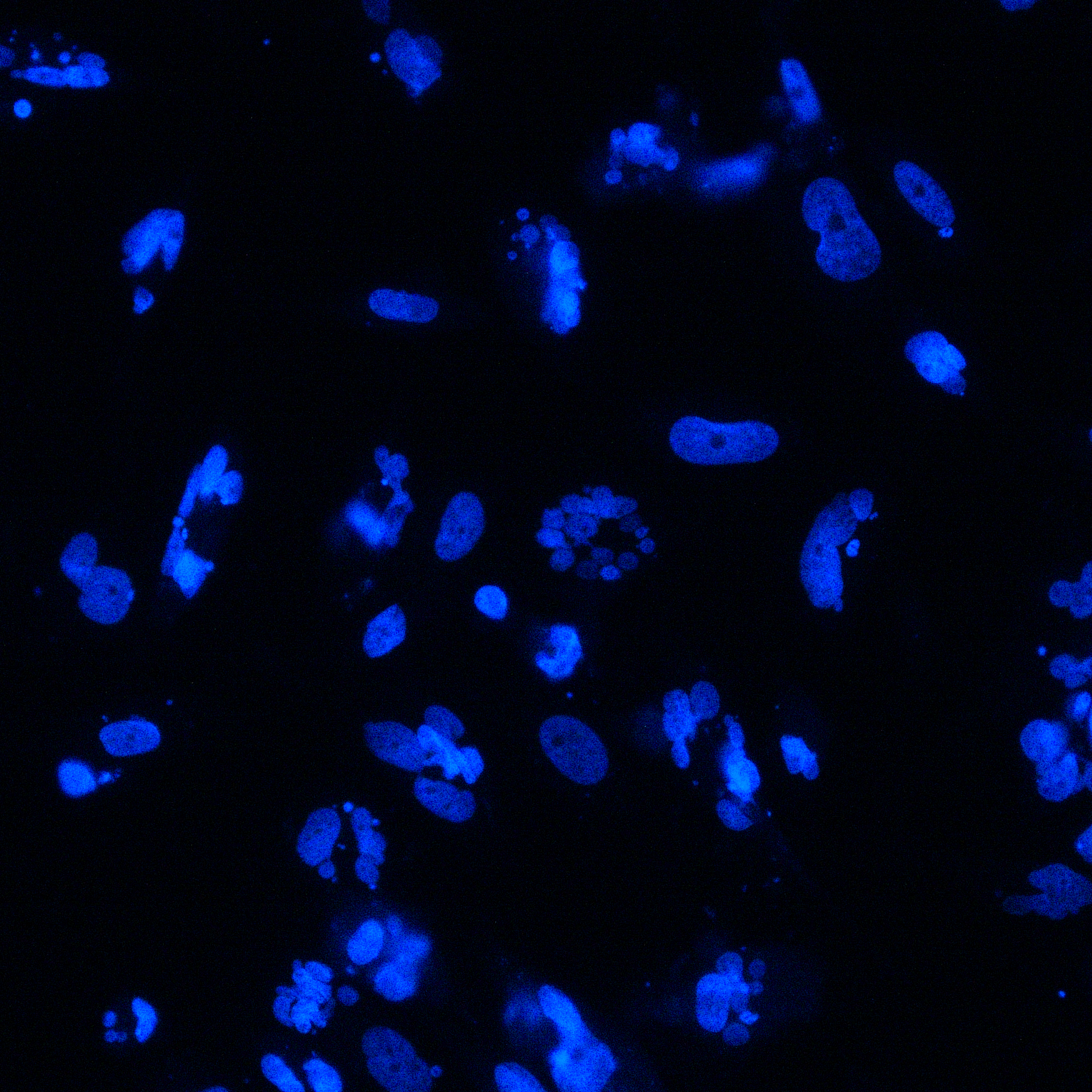

Supplement: Supplementary file 7 — Source data Fig. 5 [file 44321_2025_201_MOESM7_ESM.zip › Fig5/Fig5a bgal/245-3-IR-002c2.tif]

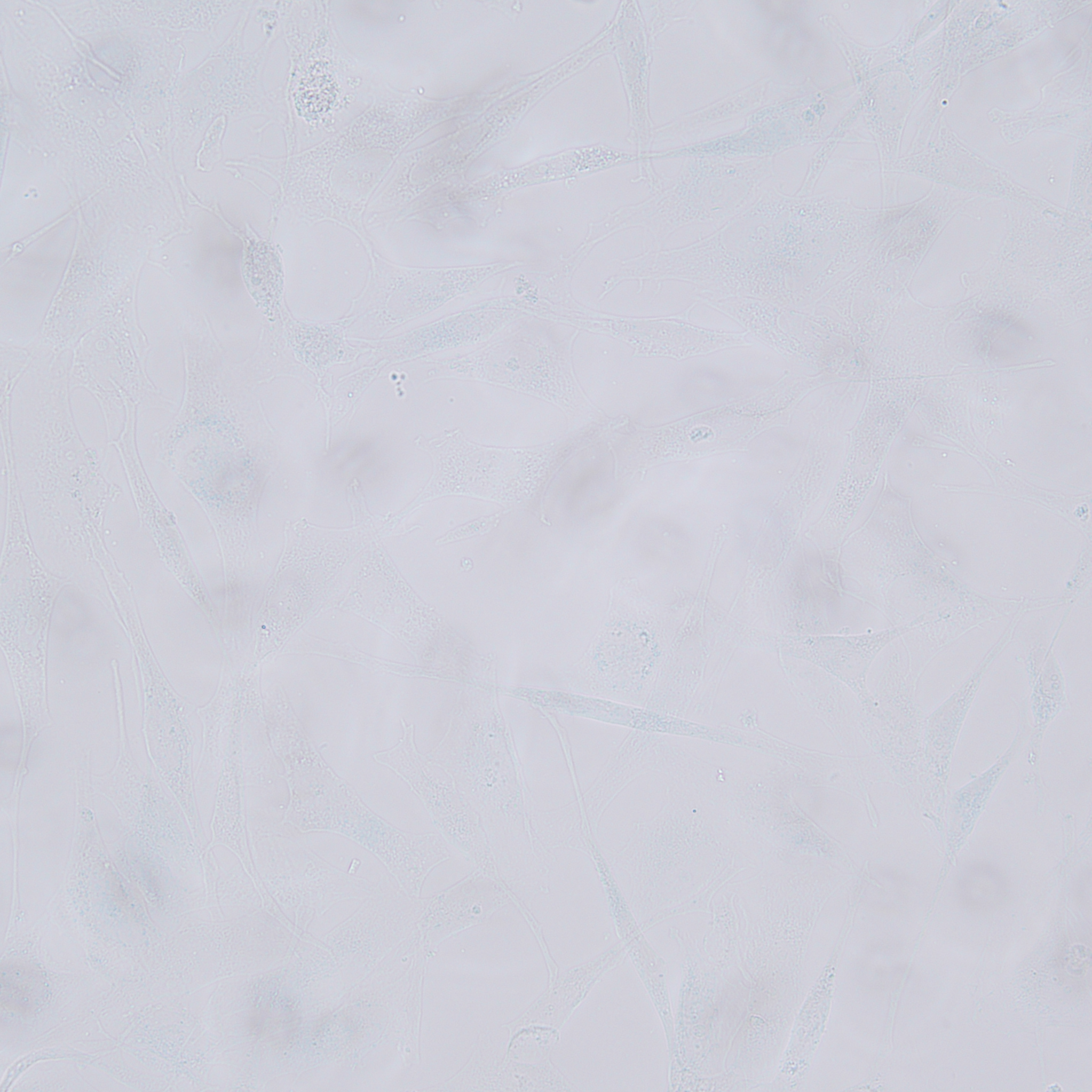

Supplement: Supplementary file 7 — Source data Fig. 5 [file 44321_2025_201_MOESM7_ESM.zip › Fig5/Fig5a bgal/245-3-mock-c1.tif]

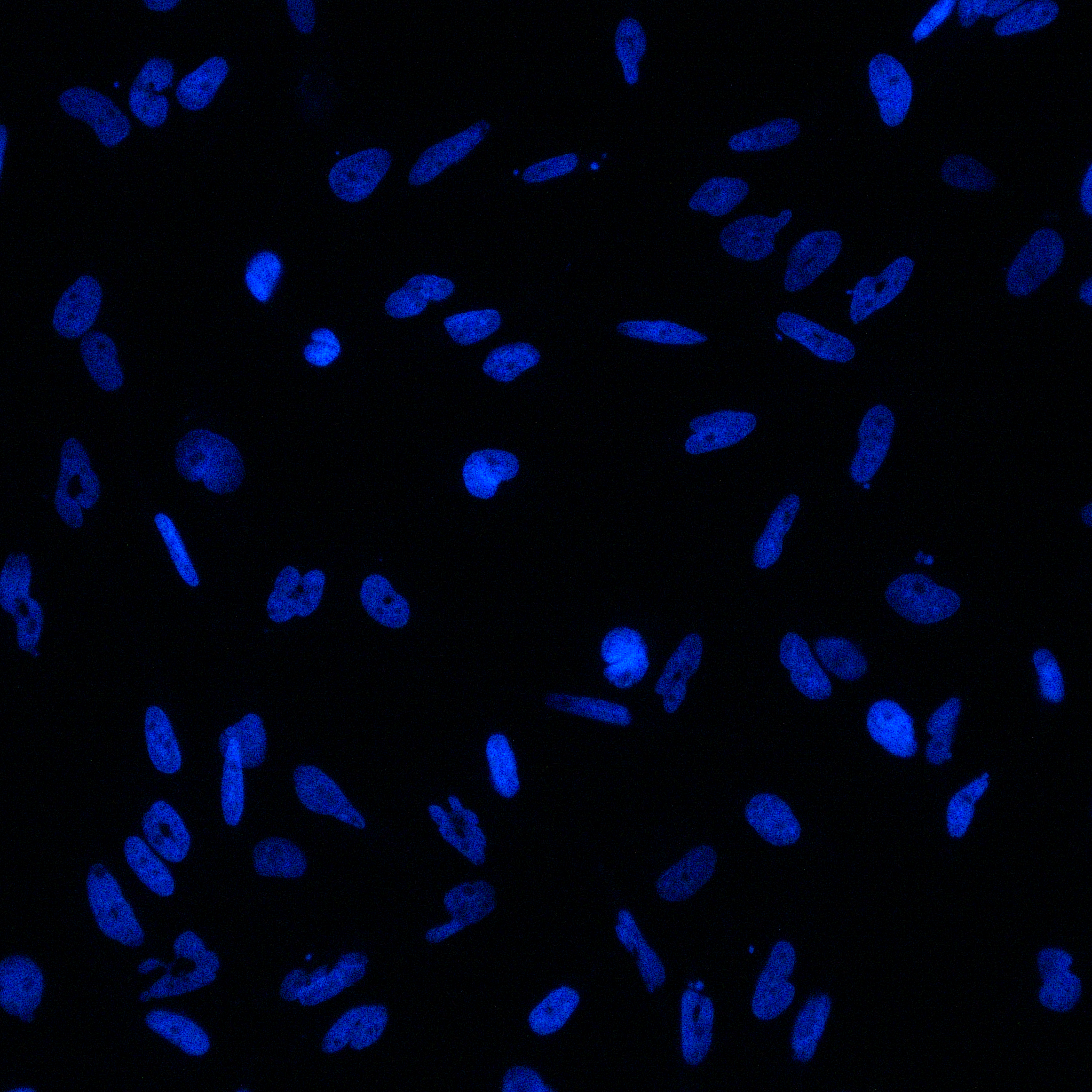

Supplement: Supplementary file 7 — Source data Fig. 5 [file 44321_2025_201_MOESM7_ESM.zip › Fig5/Fig5a bgal/245-3-mock-c2.tif]

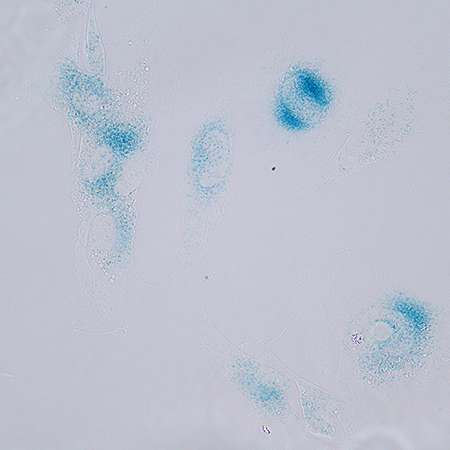

Supplement: Supplementary file 7 — Source data Fig. 5 [file 44321_2025_201_MOESM7_ESM.zip › Fig5/Fig5a bgal/43-3-IR-c1.tif]

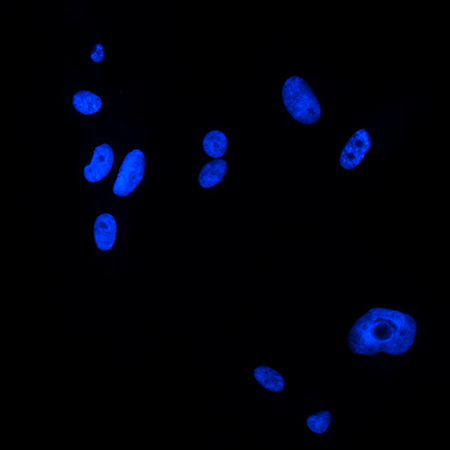

Supplement: Supplementary file 7 — Source data Fig. 5 [file 44321_2025_201_MOESM7_ESM.zip › Fig5/Fig5a bgal/43-3-IR-c2.tif]

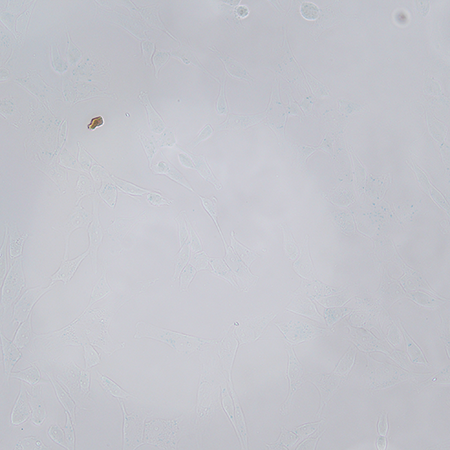

Supplement: Supplementary file 7 — Source data Fig. 5 [file 44321_2025_201_MOESM7_ESM.zip › Fig5/Fig5a bgal/43-3-mock-004c1.tif]

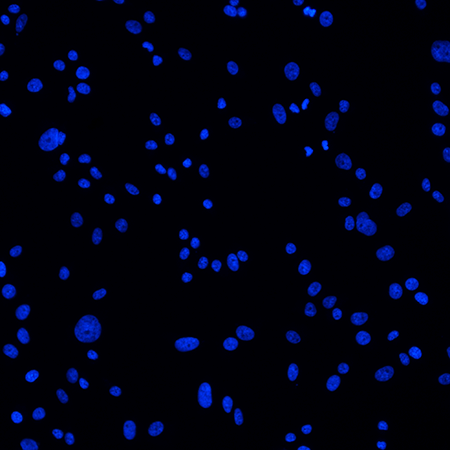

Supplement: Supplementary file 7 — Source data Fig. 5 [file 44321_2025_201_MOESM7_ESM.zip › Fig5/Fig5a bgal/43-3-mock-004c2.tif]

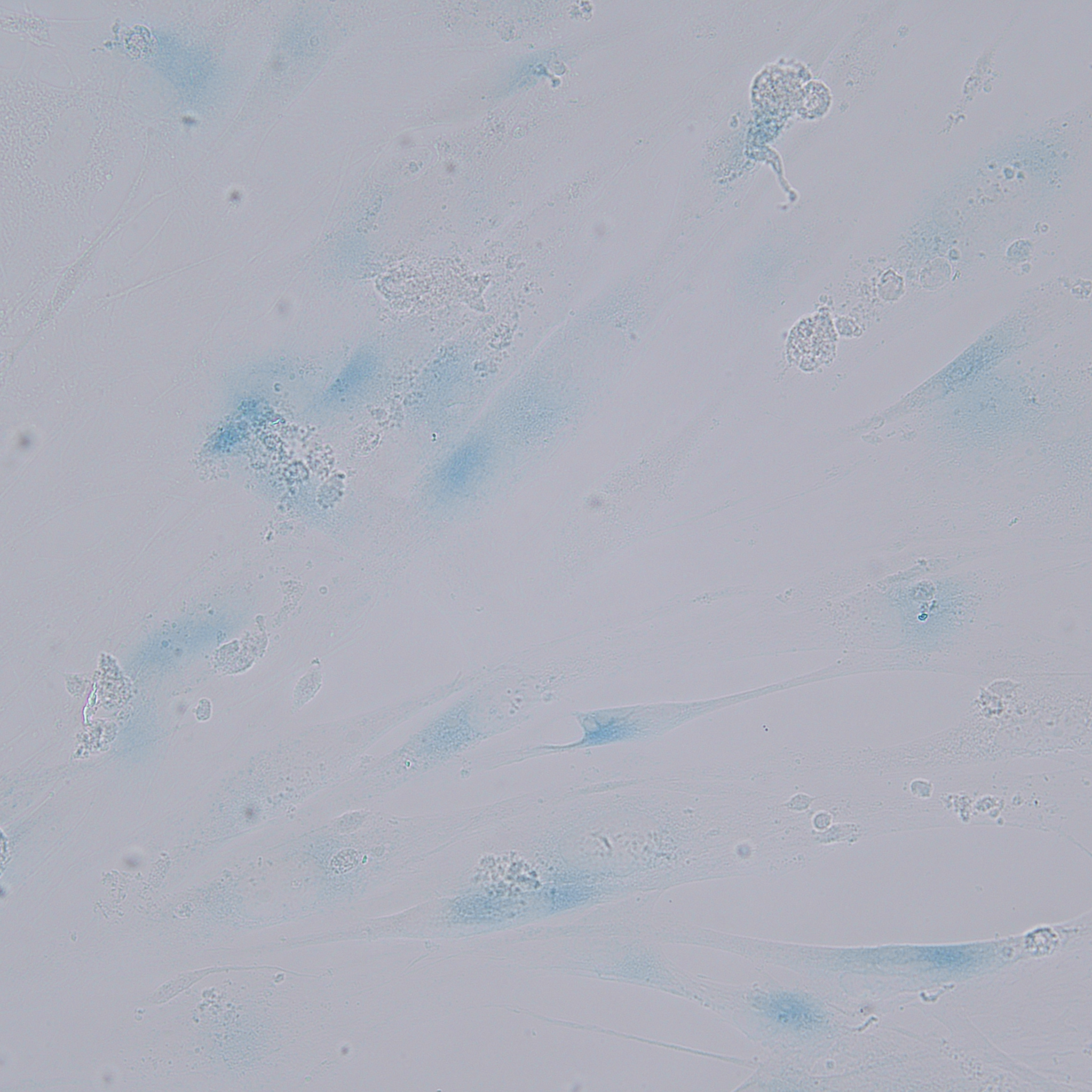

Supplement: Supplementary file 7 — Source data Fig. 5 [file 44321_2025_201_MOESM7_ESM.zip › Fig5/Fig5a bgal/6-ir-c1.tif]

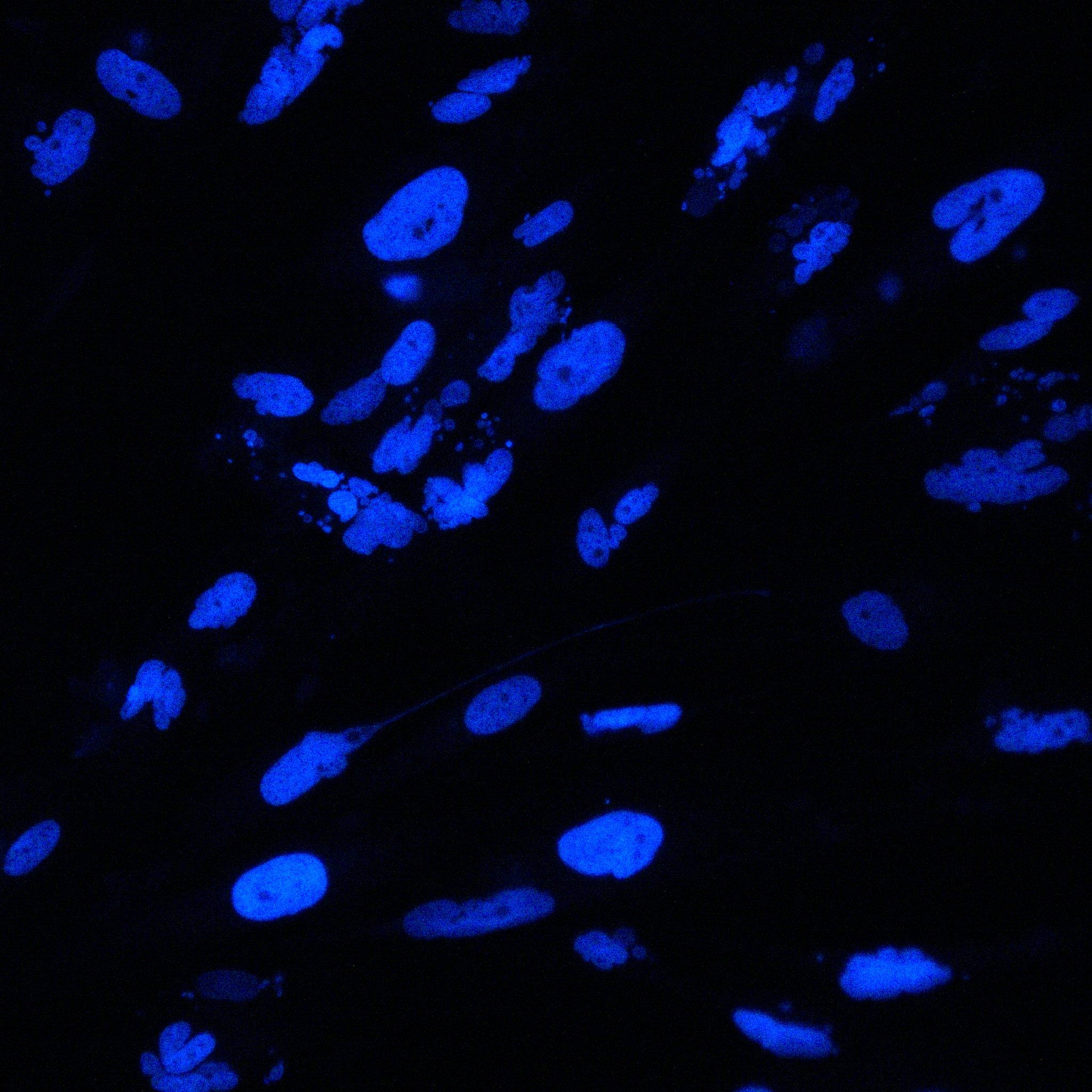

Supplement: Supplementary file 7 — Source data Fig. 5 [file 44321_2025_201_MOESM7_ESM.zip › Fig5/Fig5a bgal/6-ir-c2.tif]

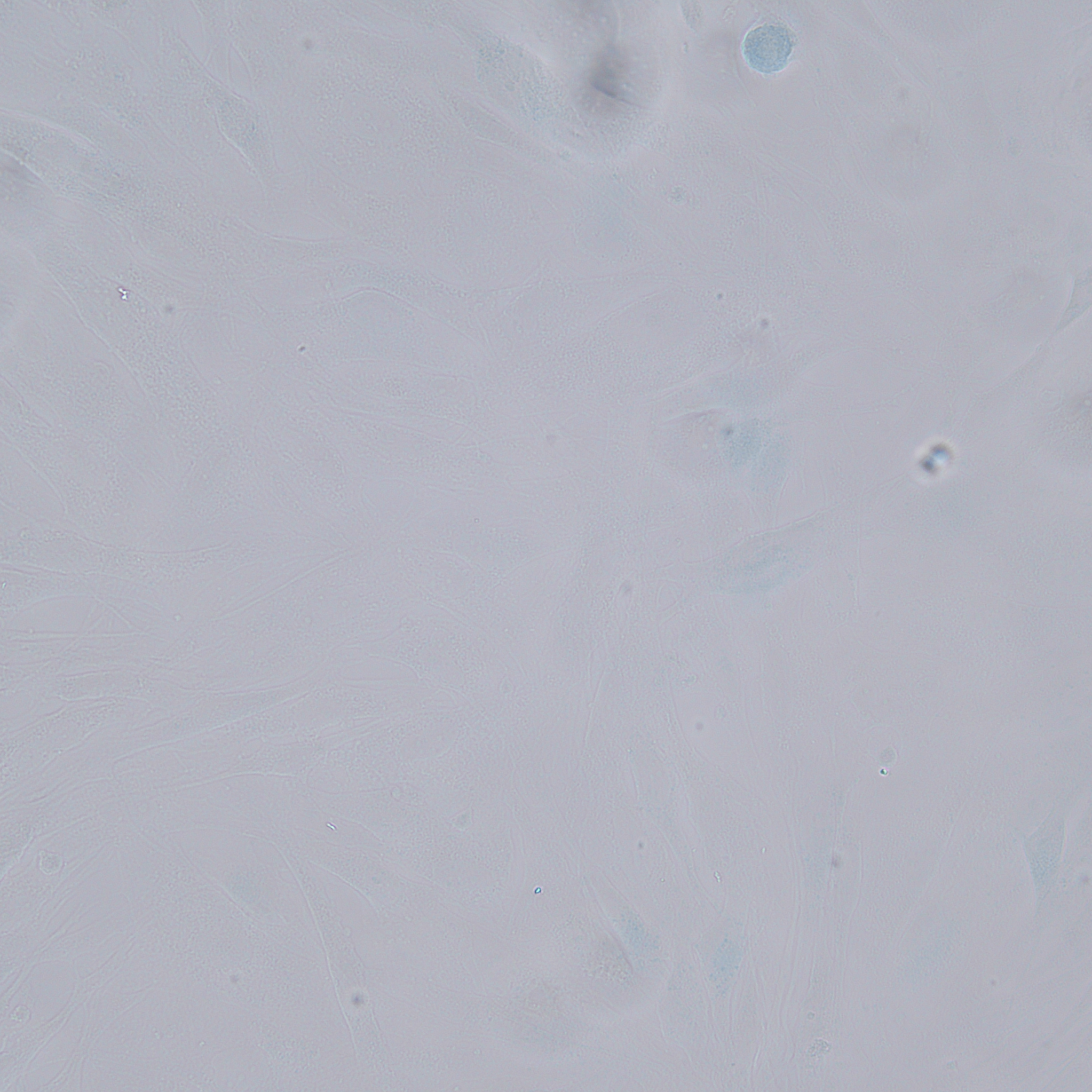

Supplement: Supplementary file 7 — Source data Fig. 5 [file 44321_2025_201_MOESM7_ESM.zip › Fig5/Fig5a bgal/6-mock-001c1.tif]

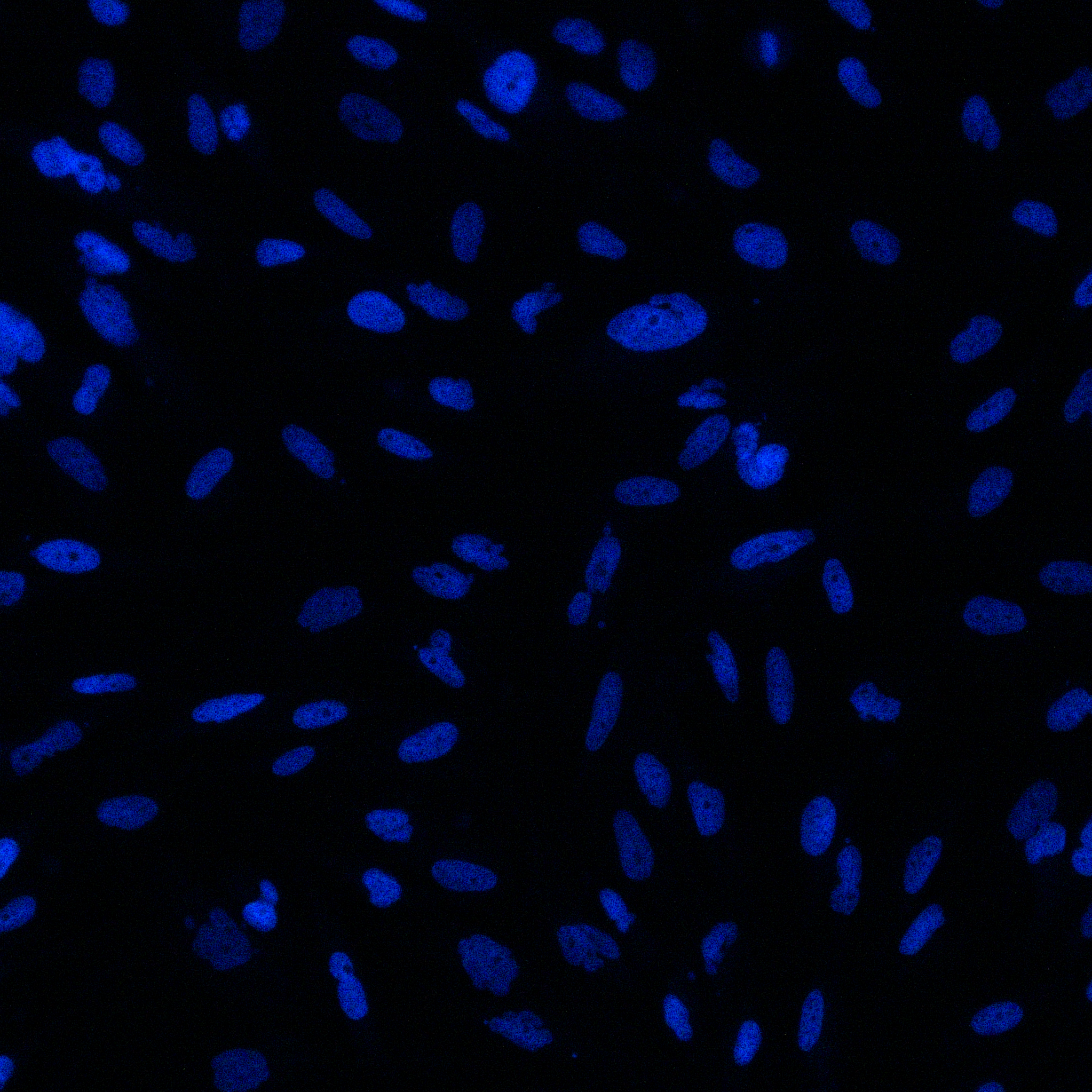

Supplement: Supplementary file 7 — Source data Fig. 5 [file 44321_2025_201_MOESM7_ESM.zip › Fig5/Fig5a bgal/6-mock-001c2.tif]

**Figure 5**

**b**

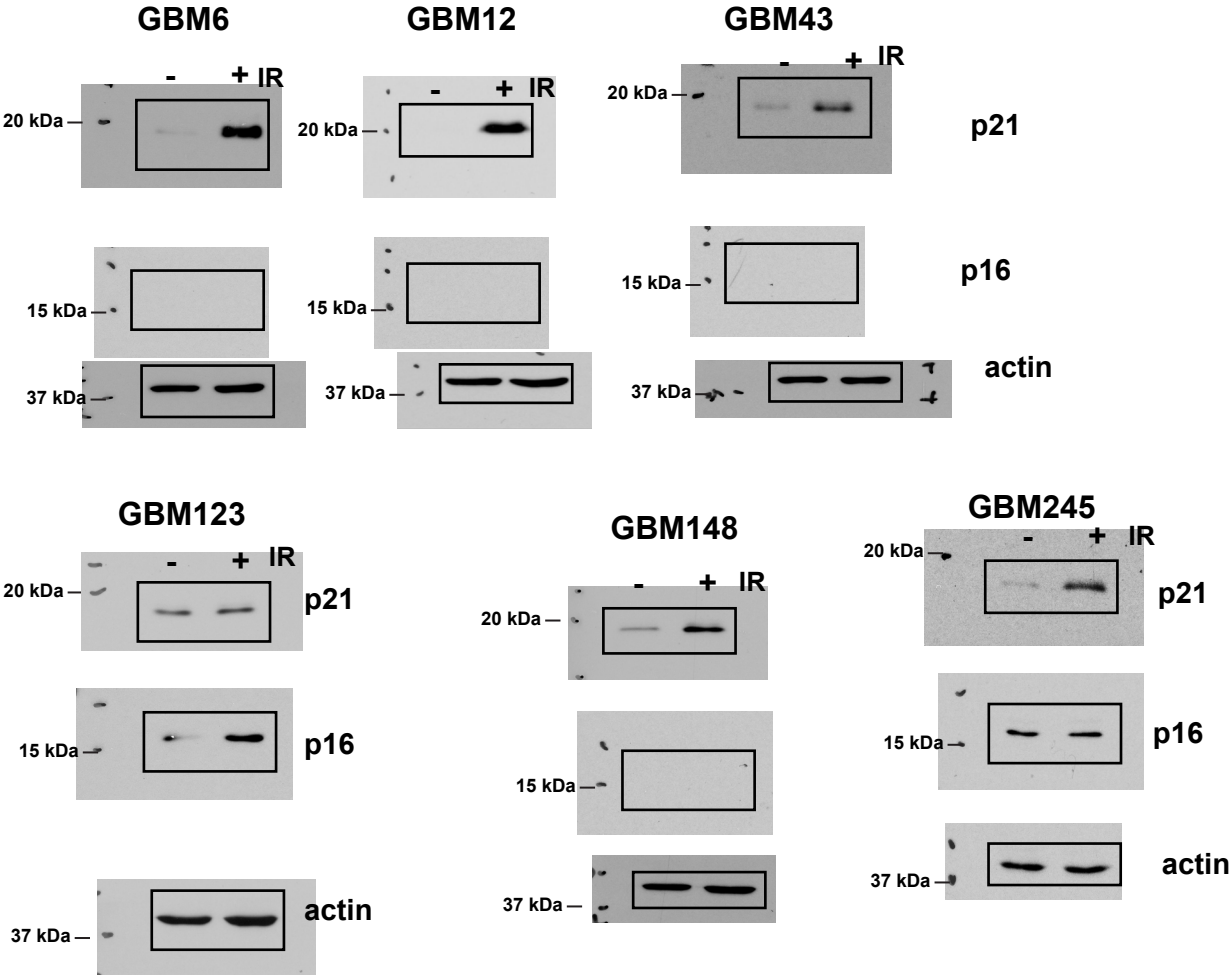

Supplement: Supplementary file 7 — Source data Fig. 5 [file 44321_2025_201_MOESM7_ESM.zip › Fig5/Fig5b WB scan p21.pdf]

Figure 5

d

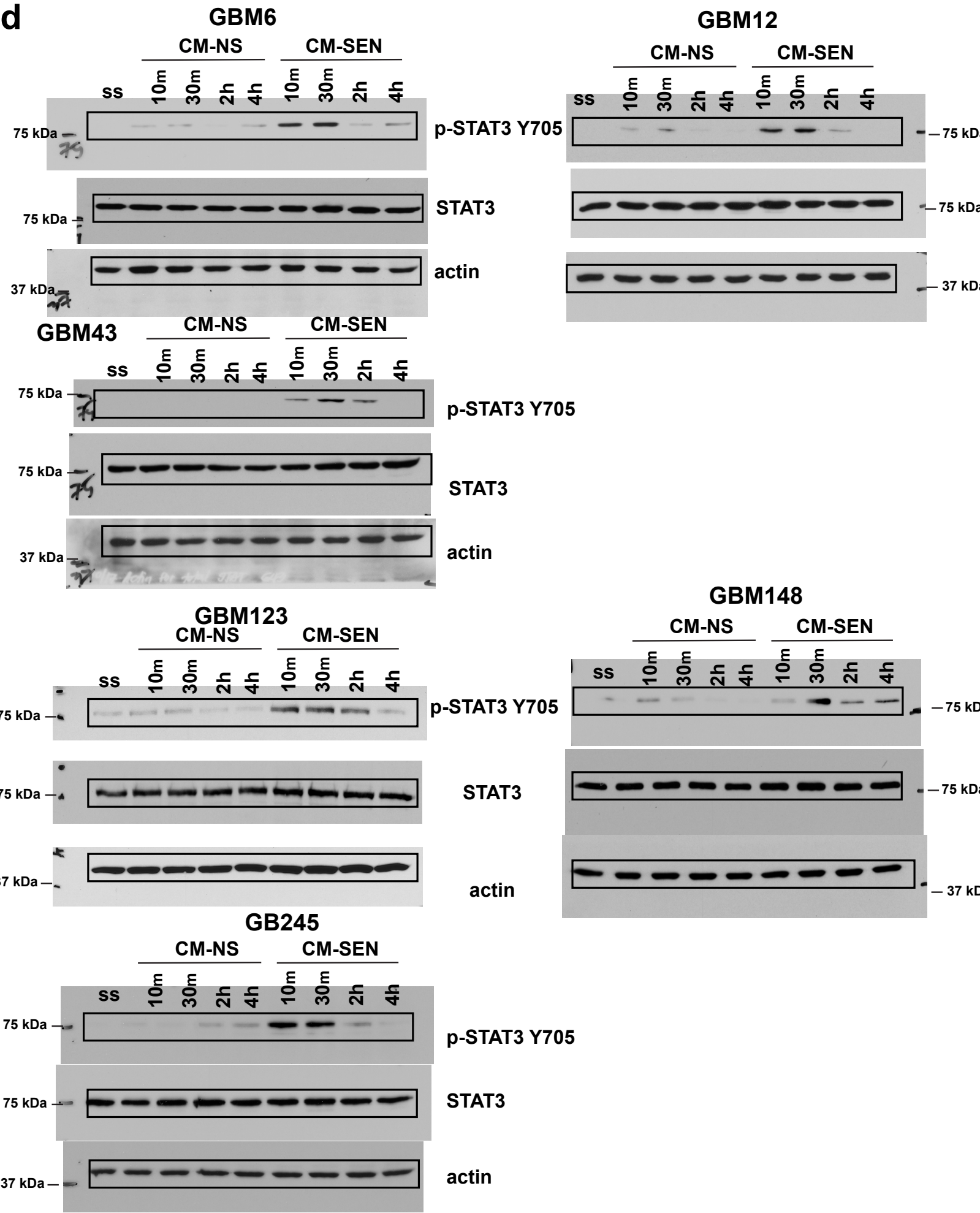

Supplement: Supplementary file 7 — Source data Fig. 5 [file 44321_2025_201_MOESM7_ESM.zip › Fig5/Figure5d PDX pSTAT3-uncropped-121024.pdf]

**Figure 6**  
**b**

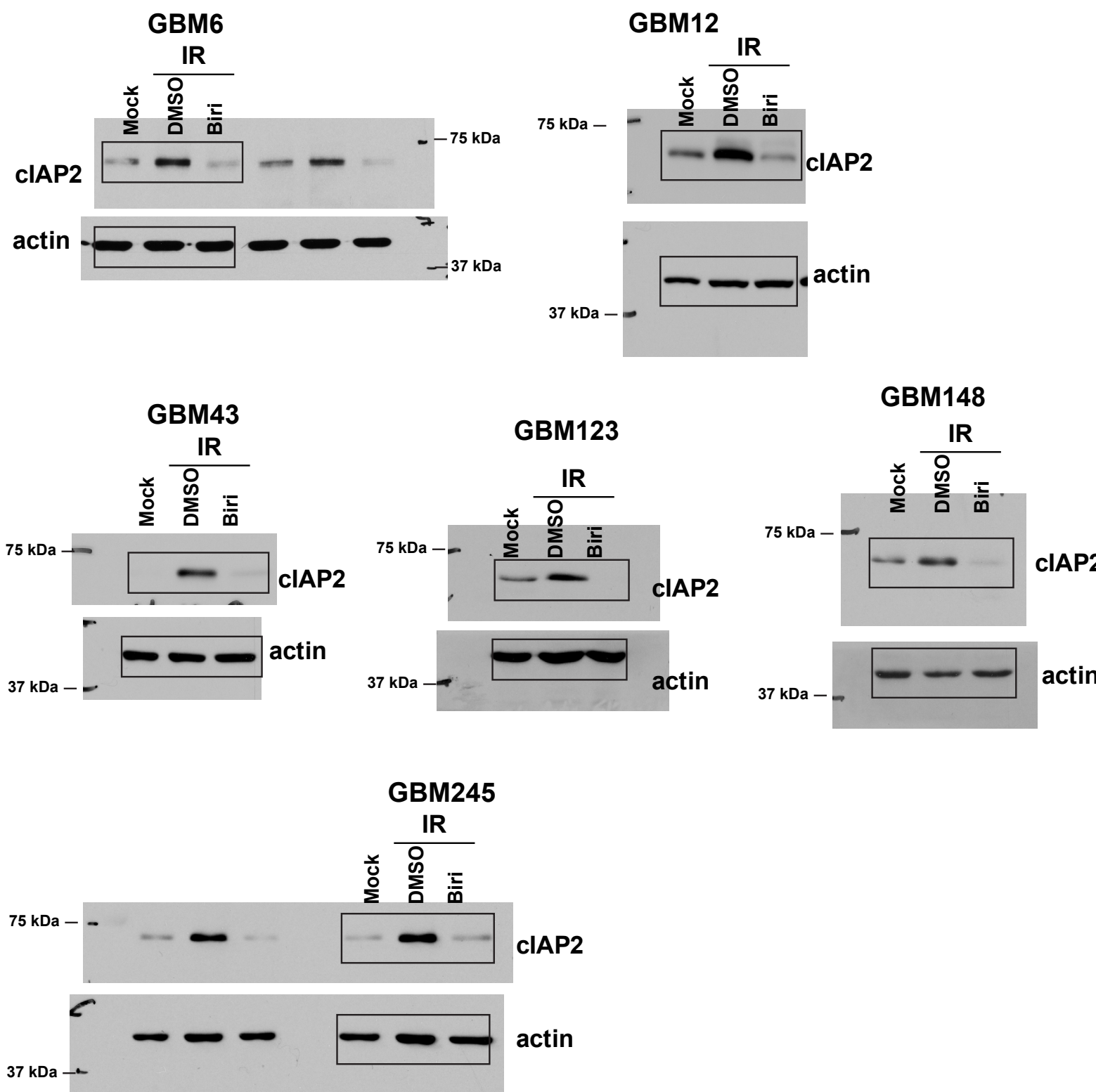

Supplement: Supplementary file 8 — Source data Fig. 6 [file 44321_2025_201_MOESM8_ESM.zip › Fig6/Fig6b PDX Biri.pdf]

Figure 6

d

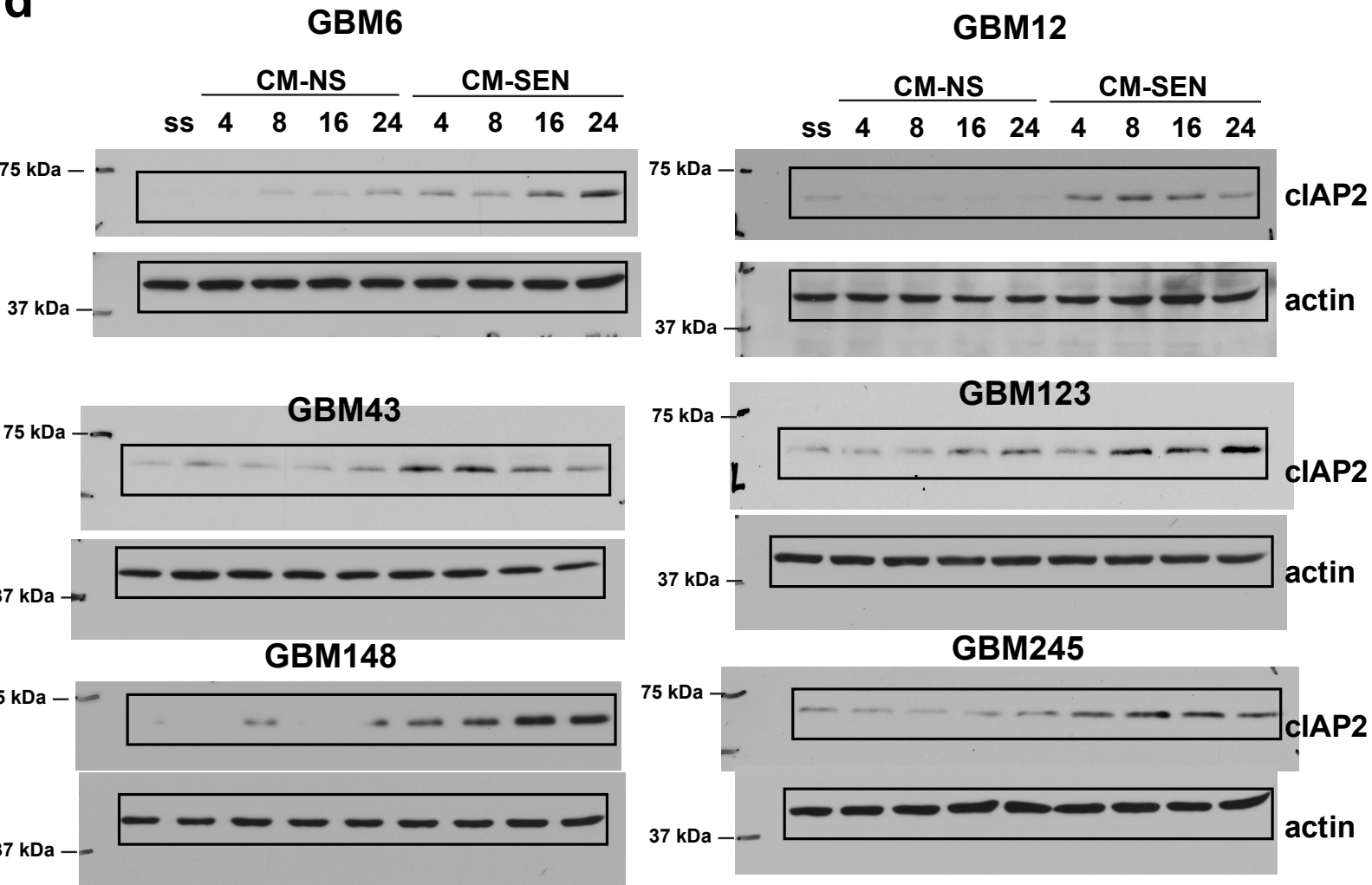

Supplement: Supplementary file 8 — Source data Fig. 6 [file 44321_2025_201_MOESM8_ESM.zip › Fig6/Fig6d CM.pdf]

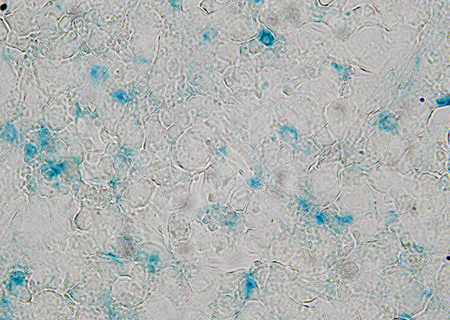

Supplement: Supplementary file 9 — Source data Fig. 7 [file 44321_2025_201_MOESM9_ESM.zip › Fig7/Fig7a-GBM12 Day5/GBM12_2499_IR_D5_40X_003c1.tif]

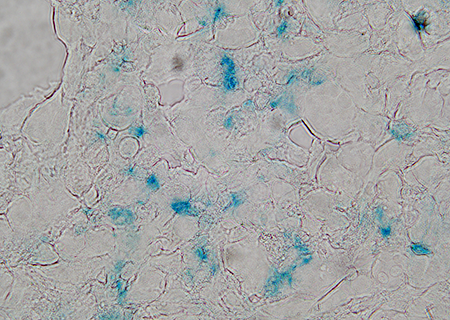

Supplement: Supplementary file 9 — Source data Fig. 7 [file 44321_2025_201_MOESM9_ESM.zip › Fig7/Fig7a-GBM12 Day5/GBM12_2500_IR_D5_40X_005c1.tif]

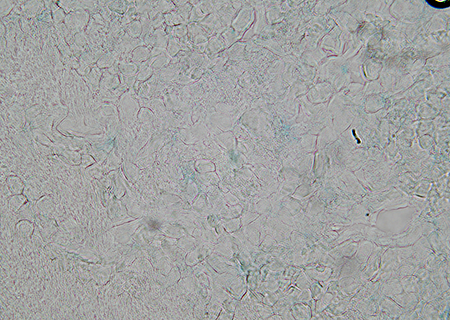

Supplement: Supplementary file 9 — Source data Fig. 7 [file 44321_2025_201_MOESM9_ESM.zip › Fig7/Fig7a-GBM12 Day5/GBM12_2508_mock_ 40x 001c1.tif]

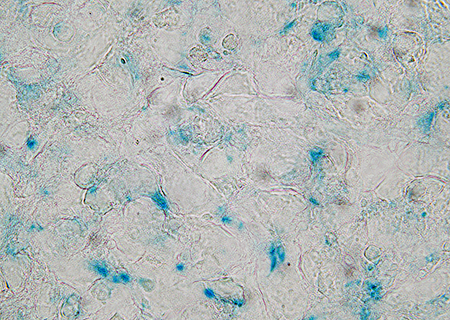

Supplement: Supplementary file 9 — Source data Fig. 7 [file 44321_2025_201_MOESM9_ESM.zip › Fig7/Fig7a-GBM12 Day5/GBM12_2510_IR_D5_40X_007c1.tif]

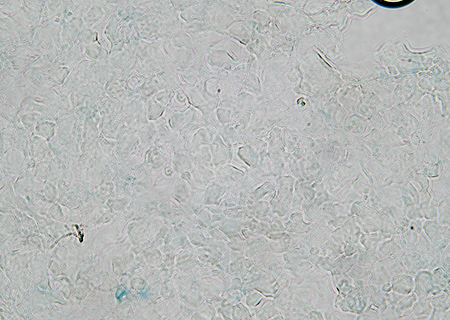

Supplement: Supplementary file 9 — Source data Fig. 7 [file 44321_2025_201_MOESM9_ESM.zip › Fig7/Fig7a-GBM12 Day5/GBM12_2545_mock_40X_005c1.tif]

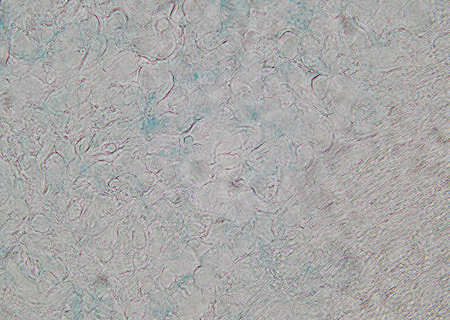

Supplement: Supplementary file 9 — Source data Fig. 7 [file 44321_2025_201_MOESM9_ESM.zip › Fig7/Fig7a-GBM12 Day5/GBM12_2552_mock_ 40x 005c1.tif]

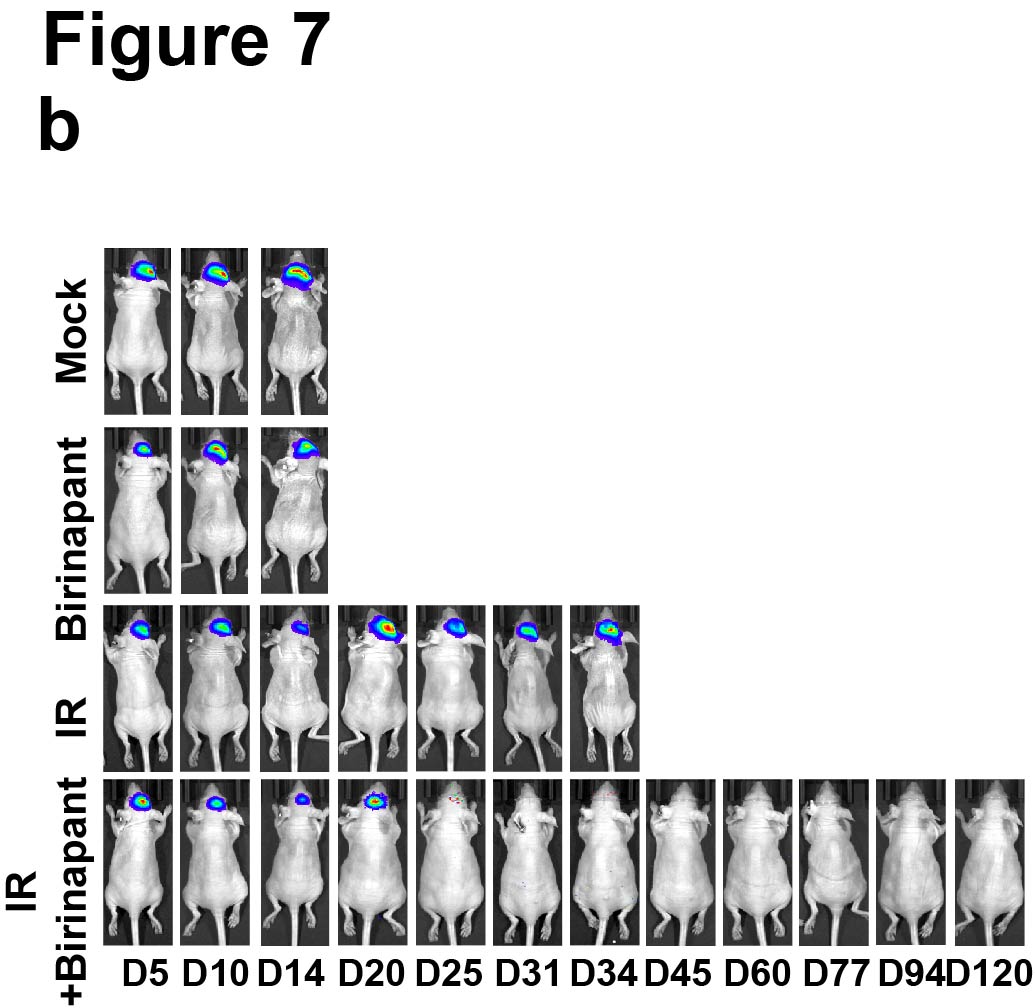

Supplement: Supplementary file 9 — Source data Fig. 7 [file 44321_2025_201_MOESM9_ESM.zip › Fig7/Fig7b-GBM12 BLI.jpg]

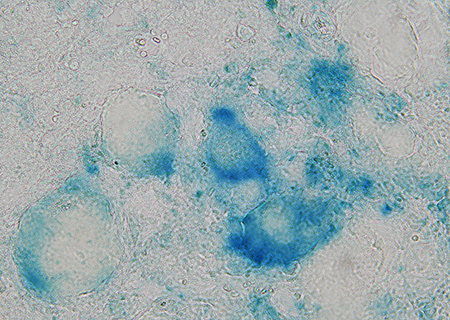

Supplement: Supplementary file 9 — Source data Fig. 7 [file 44321_2025_201_MOESM9_ESM.zip › Fig7/Fig7d-GBM12 endpoint/GBM12_2498_IR_D21_40X_001c1.tif]

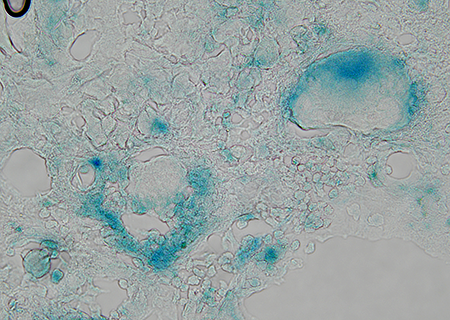

Supplement: Supplementary file 9 — Source data Fig. 7 [file 44321_2025_201_MOESM9_ESM.zip › Fig7/Fig7d-GBM12 endpoint/GBM12_2544_IR_D24_40X_002c1.tif]

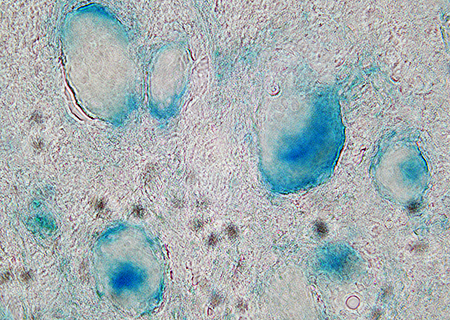

Supplement: Supplementary file 9 — Source data Fig. 7 [file 44321_2025_201_MOESM9_ESM.zip › Fig7/Fig7d-GBM12 endpoint/GBM12_2553_IR_D24_40X_002c1.tif]

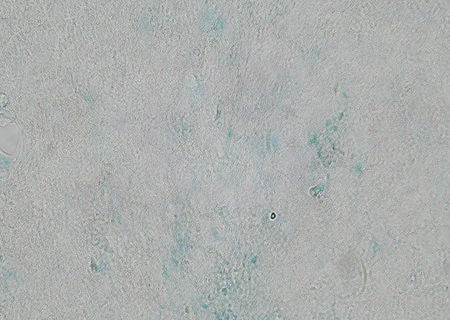

Supplement: Supplementary file 9 — Source data Fig. 7 [file 44321_2025_201_MOESM9_ESM.zip › Fig7/Fig7d-GBM12 endpoint/GBM12_2556_IR+Bir_40X_002c1.tif]

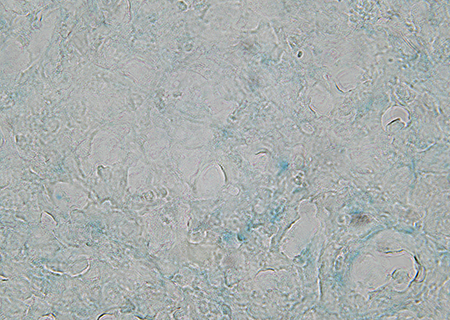

Supplement: Supplementary file 9 — Source data Fig. 7 [file 44321_2025_201_MOESM9_ESM.zip › Fig7/Fig7d-GBM12 endpoint/GBM12_2559_IR+Bir_40X_003c1.tif]

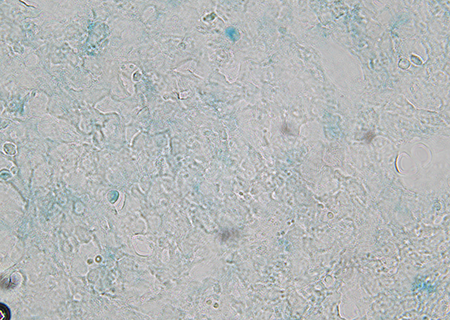

Supplement: Supplementary file 9 — Source data Fig. 7 [file 44321_2025_201_MOESM9_ESM.zip › Fig7/Fig7d-GBM12 endpoint/GBM12_2560_IR+Bir_40X_005c1.tif]

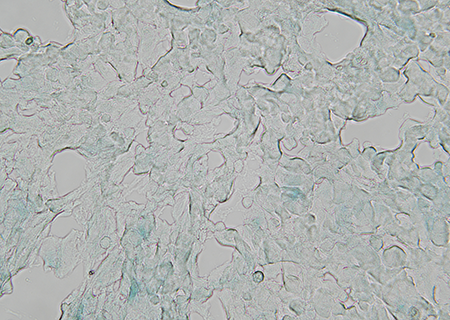

Supplement: Supplementary file 9 — Source data Fig. 7 [file 44321_2025_201_MOESM9_ESM.zip › Fig7/Fig7e-GL261 Day5/2432_mock_40X_004c2.tif]

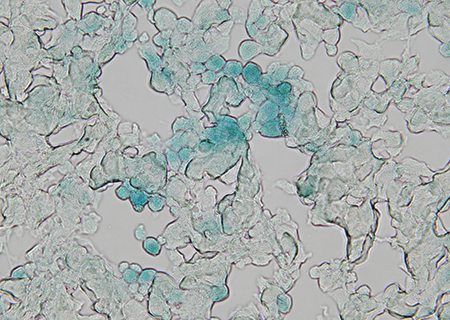

Supplement: Supplementary file 9 — Source data Fig. 7 [file 44321_2025_201_MOESM9_ESM.zip › Fig7/Fig7e-GL261 Day5/2433_IR_day5_40X_002c1.tif]

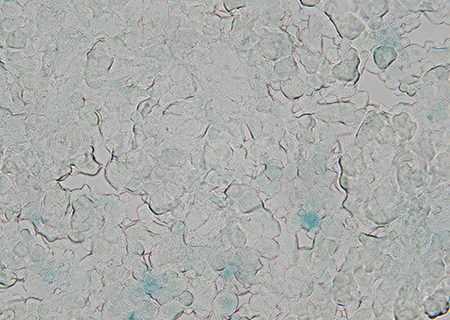

Supplement: Supplementary file 9 — Source data Fig. 7 [file 44321_2025_201_MOESM9_ESM.zip › Fig7/Fig7e-GL261 Day5/2439_mock_40X_003c2.tif]

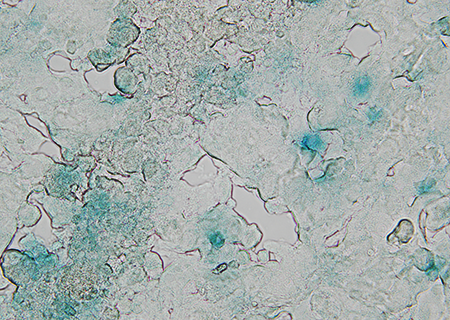

Supplement: Supplementary file 9 — Source data Fig. 7 [file 44321_2025_201_MOESM9_ESM.zip › Fig7/Fig7e-GL261 Day5/2530_IR_day4_40X_005c1.tif]

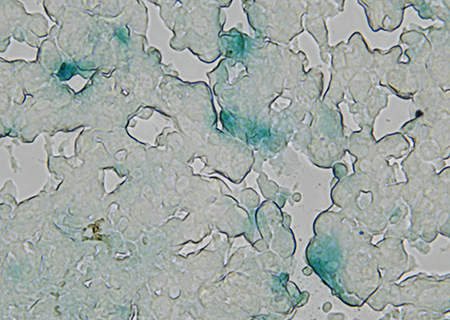

Supplement: Supplementary file 9 — Source data Fig. 7 [file 44321_2025_201_MOESM9_ESM.zip › Fig7/Fig7e-GL261 Day5/2531_IR_day4_40X_002c1.tif]

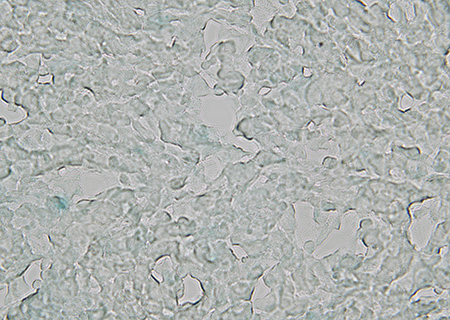

Supplement: Supplementary file 9 — Source data Fig. 7 [file 44321_2025_201_MOESM9_ESM.zip › Fig7/Fig7e-GL261 Day5/2532_mock_40X_003c2.tif]

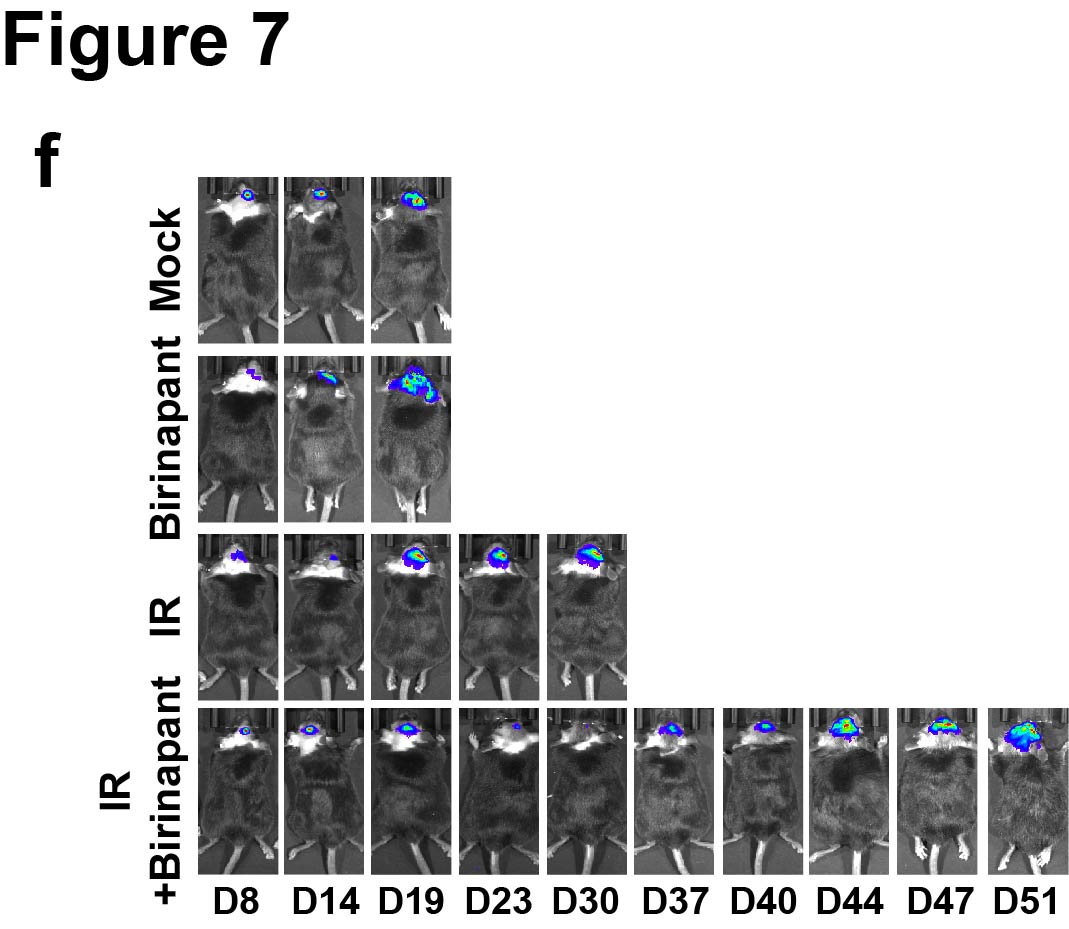

Supplement: Supplementary file 9 — Source data Fig. 7 [file 44321_2025_201_MOESM9_ESM.zip › Fig7/Fig7f-GL261 BLI.jpg]

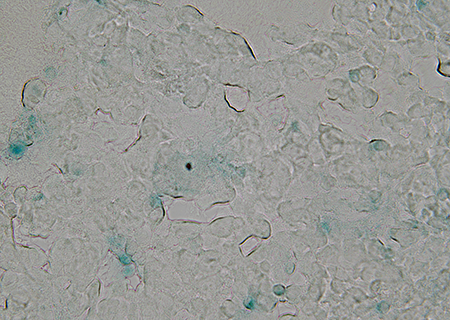

Supplement: Supplementary file 9 — Source data Fig. 7 [file 44321_2025_201_MOESM9_ESM.zip › Fig7/Fig7h-GL261 Birinapant/2567_IR + Bir_40X_003c2.tif]

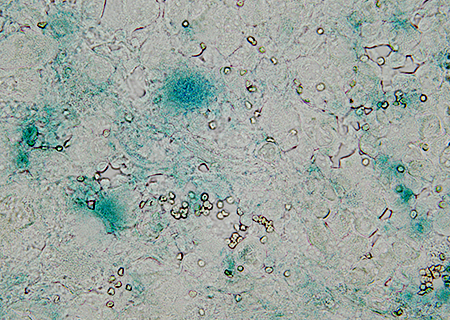

Supplement: Supplementary file 9 — Source data Fig. 7 [file 44321_2025_201_MOESM9_ESM.zip › Fig7/Fig7h-GL261 Birinapant/2568_IR endoint_40X_001c2.tif]

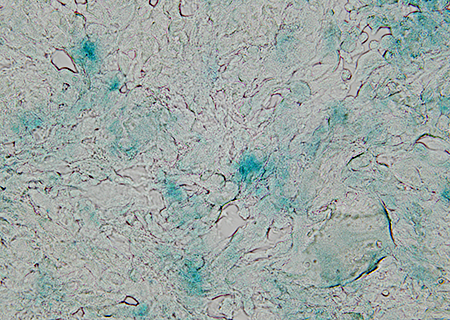

Supplement: Supplementary file 9 — Source data Fig. 7 [file 44321_2025_201_MOESM9_ESM.zip › Fig7/Fig7h-GL261 Birinapant/2569_IR endoint_40X_006c2.tif]

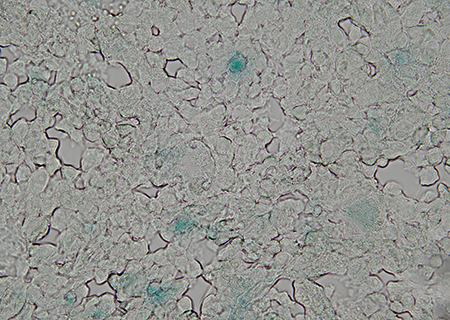

Supplement: Supplementary file 9 — Source data Fig. 7 [file 44321_2025_201_MOESM9_ESM.zip › Fig7/Fig7h-GL261 Birinapant/2570_IR + Bir_40X_005c2.tif]

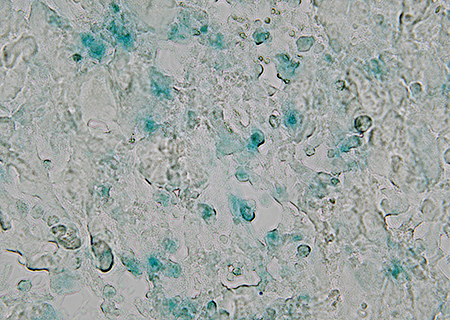

Supplement: Supplementary file 9 — Source data Fig. 7 [file 44321_2025_201_MOESM9_ESM.zip › Fig7/Fig7h-GL261 Birinapant/2574_IR endoint_40X_007c2.tif]

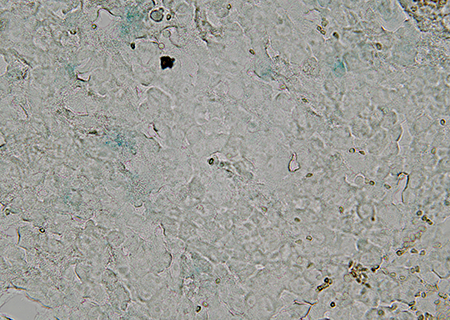

Supplement: Supplementary file 9 — Source data Fig. 7 [file 44321_2025_201_MOESM9_ESM.zip › Fig7/Fig7h-GL261 Birinapant/2578_IR + Bir_40X_007c2.tif]

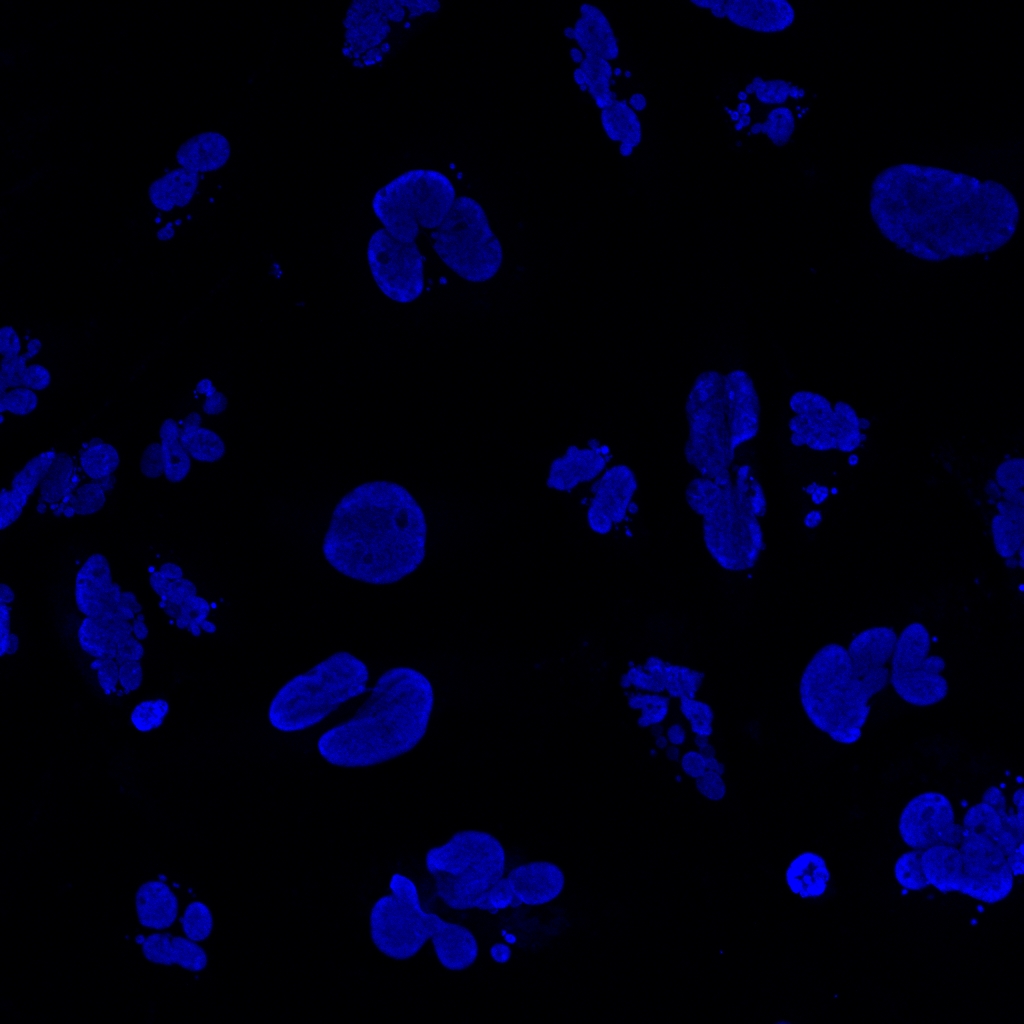

Supplement: Supplementary file 10 — EV figures [file 44321_2025_201_MOESM10_ESM.zip › source data for EV/EV1/EV1 a/229/229 IR-5-Image Export-07_c1.jpg]

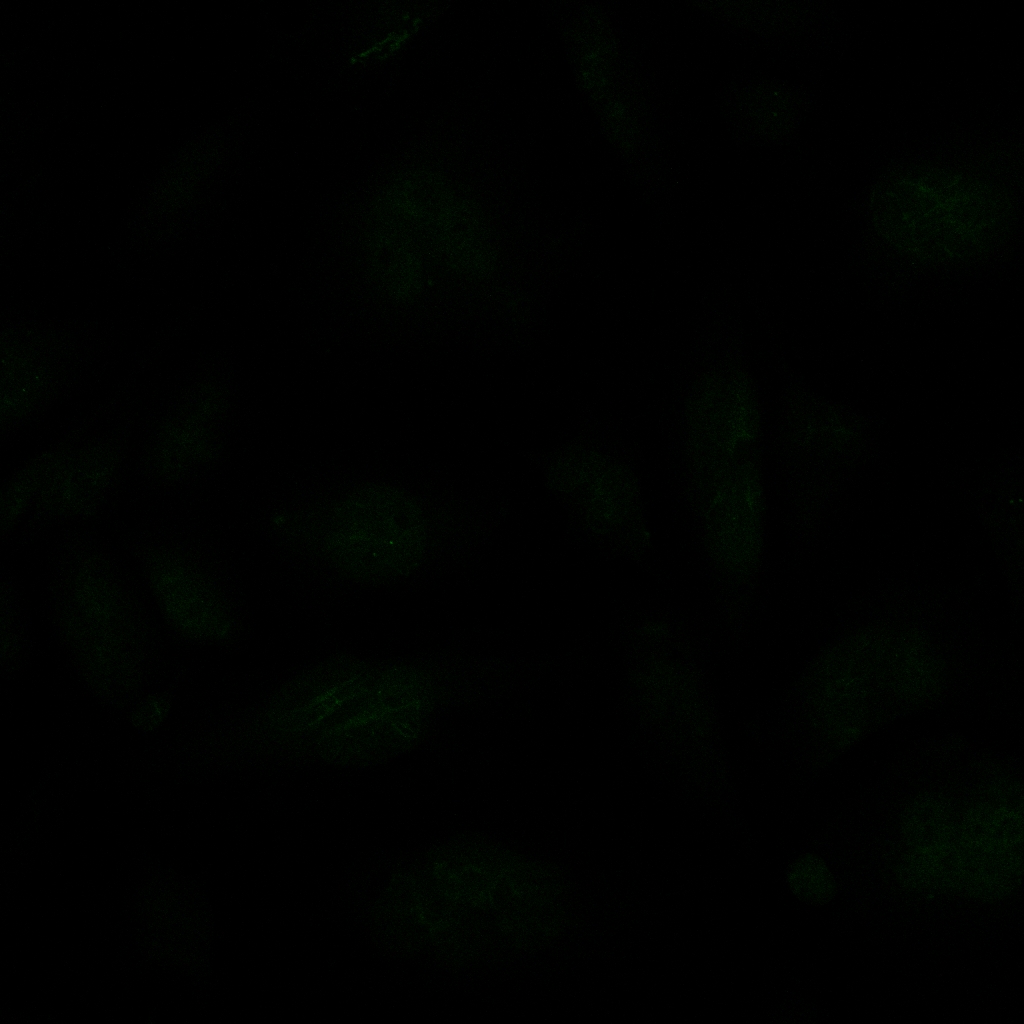

Supplement: Supplementary file 10 — EV figures [file 44321_2025_201_MOESM10_ESM.zip › source data for EV/EV1/EV1 a/229/229 IR-5-Image Export-07_c2.jpg]

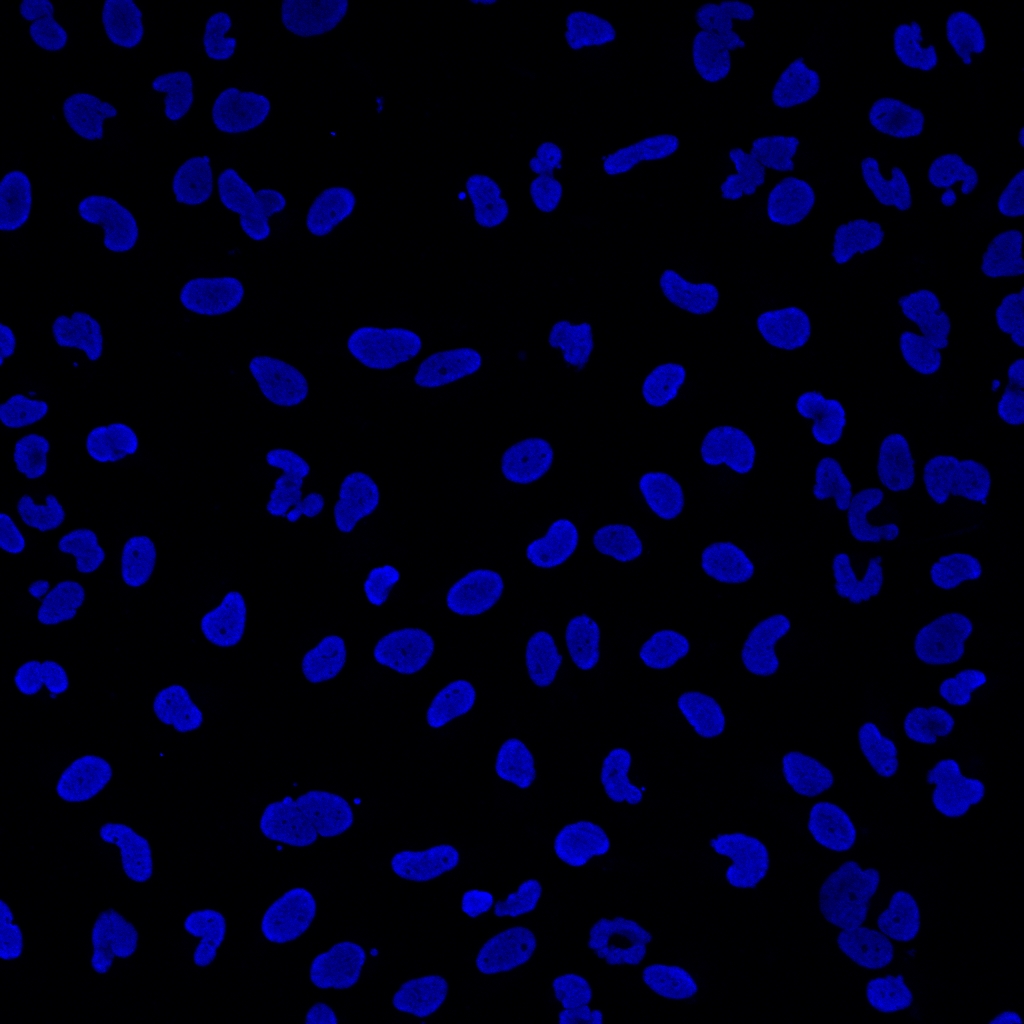

Supplement: Supplementary file 10 — EV figures [file 44321_2025_201_MOESM10_ESM.zip › source data for EV/EV1/EV1 a/229/229 mock-1-Image Export-08_c1.jpg]

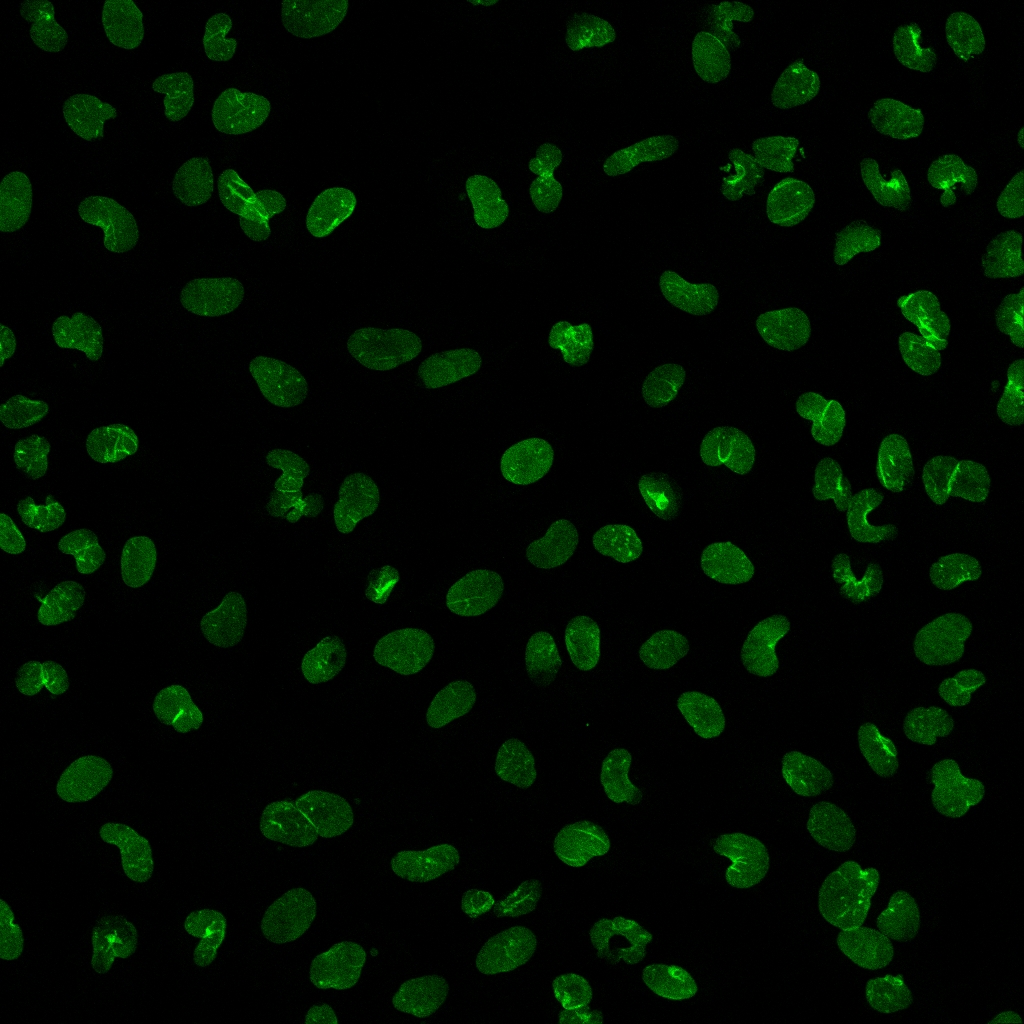

Supplement: Supplementary file 10 — EV figures [file 44321_2025_201_MOESM10_ESM.zip › source data for EV/EV1/EV1 a/229/229 mock-1-Image Export-08_c2.jpg]

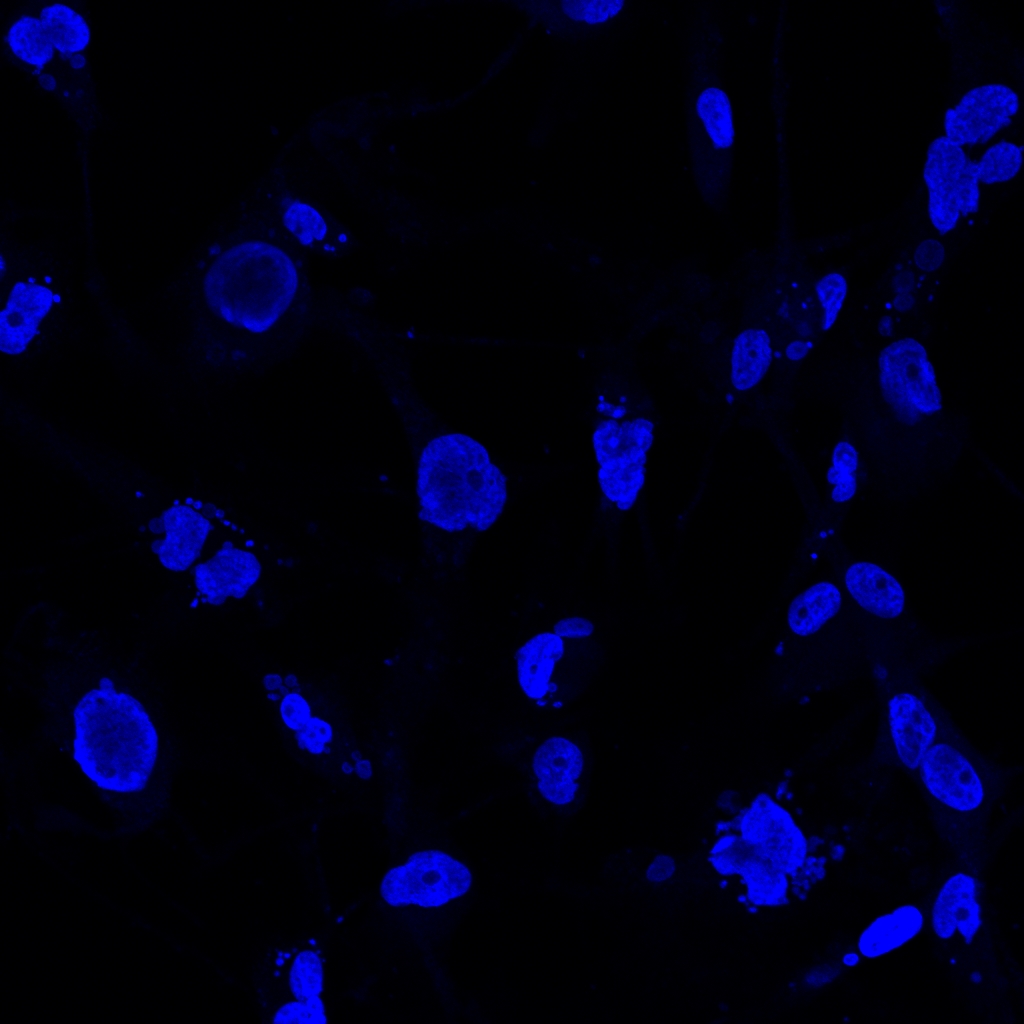

Supplement: Supplementary file 10 — EV figures [file 44321_2025_201_MOESM10_ESM.zip › source data for EV/EV1/EV1 a/A172/A172-IR-4-Image Export-14_c1.jpg]

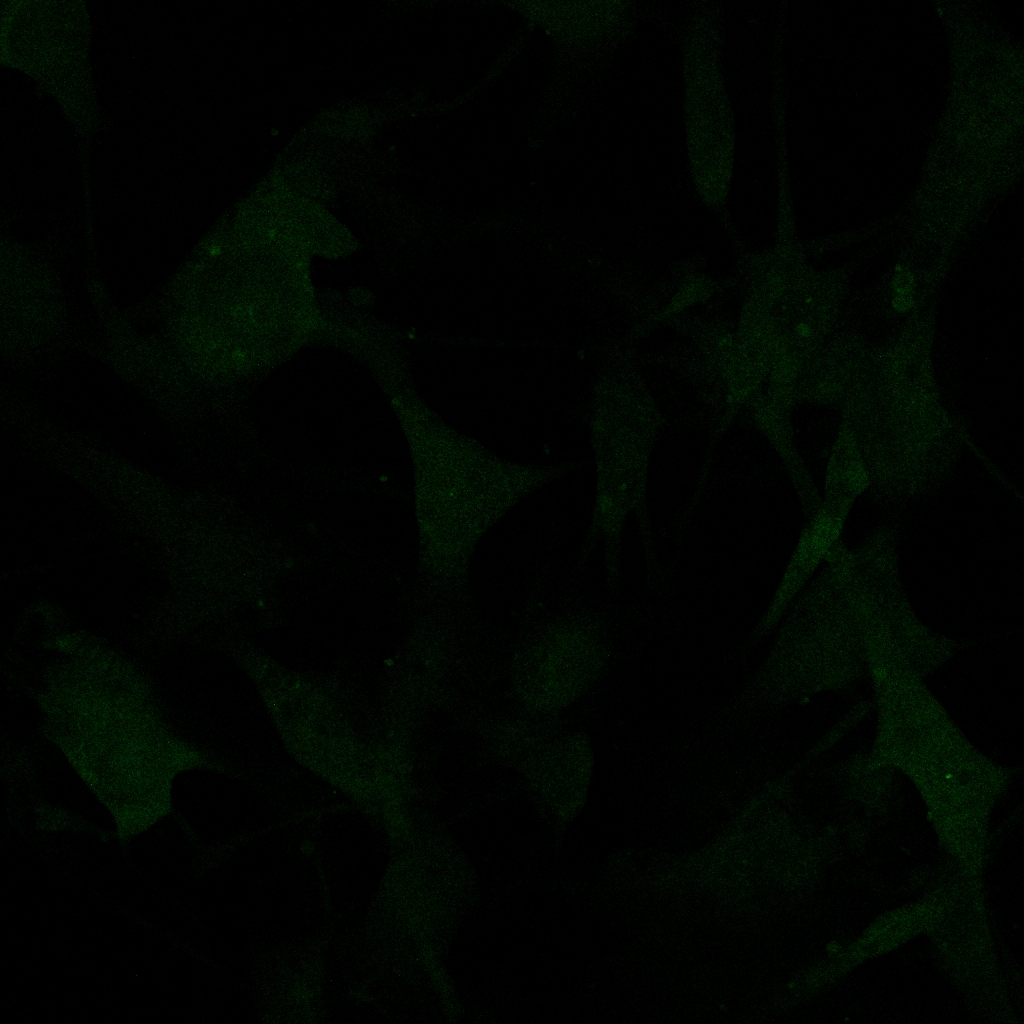

Supplement: Supplementary file 10 — EV figures [file 44321_2025_201_MOESM10_ESM.zip › source data for EV/EV1/EV1 a/A172/A172-IR-4-Image Export-14_c2.jpg]

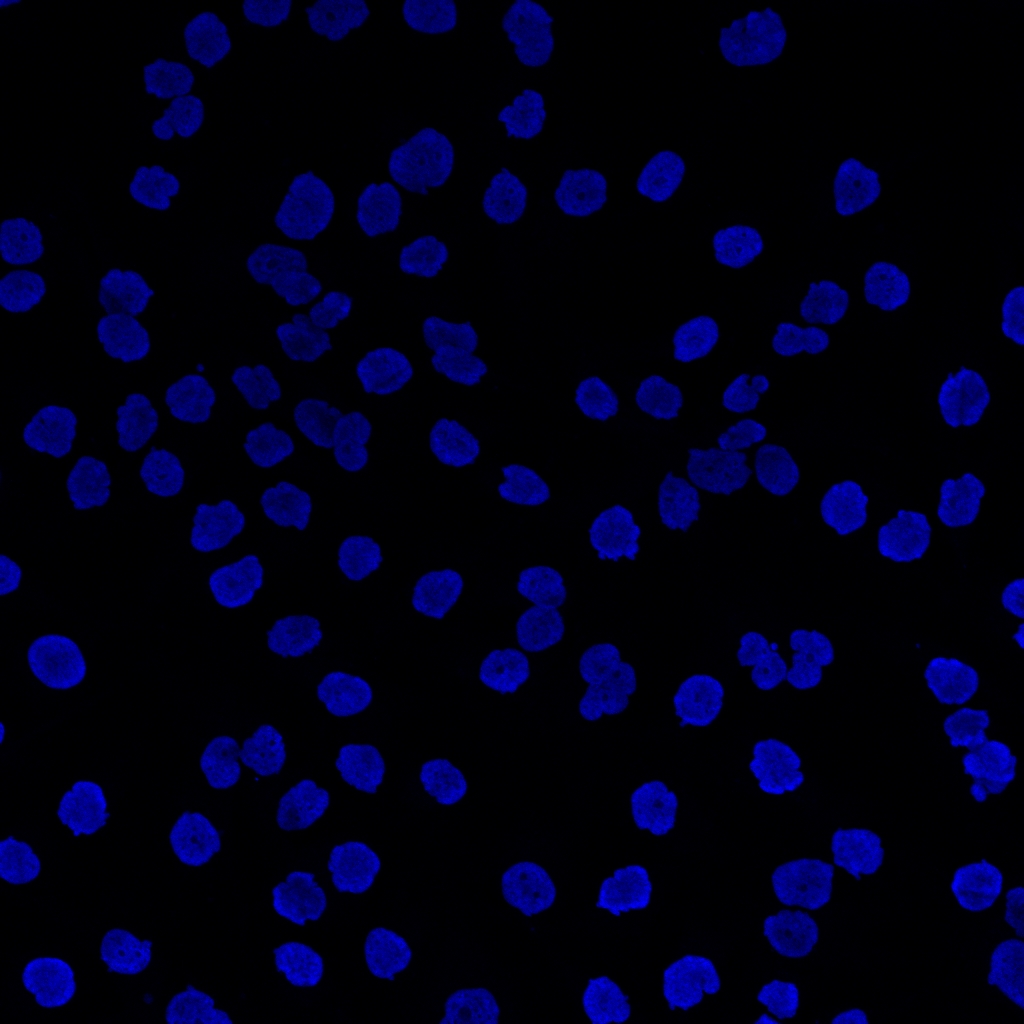

Supplement: Supplementary file 10 — EV figures [file 44321_2025_201_MOESM10_ESM.zip › source data for EV/EV1/EV1 a/A172/A172-mock-1-Image Export-16_c1.jpg]

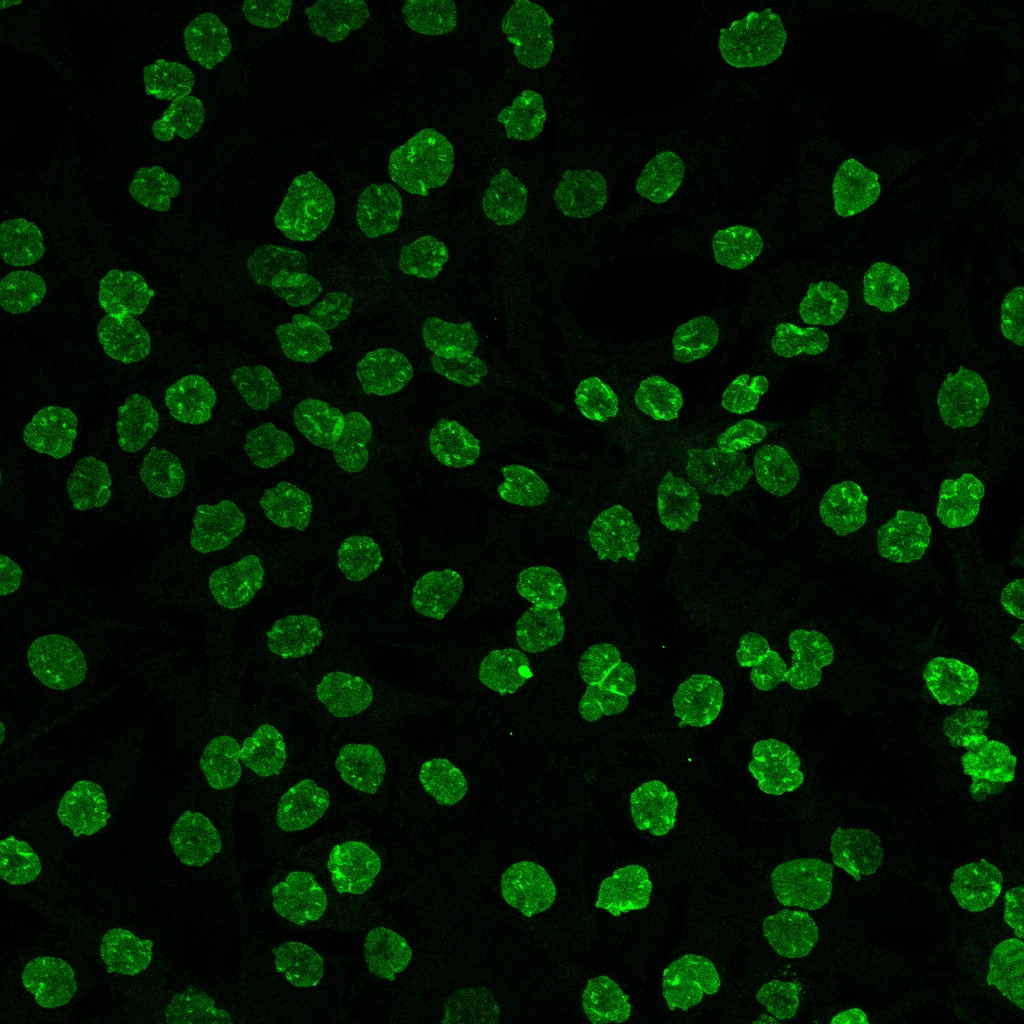

Supplement: Supplementary file 10 — EV figures [file 44321_2025_201_MOESM10_ESM.zip › source data for EV/EV1/EV1 a/A172/A172-mock-1-Image Export-16_c2.jpg]

Mock

IR

DAPI

Lamin B1

DAPI

Lamin B1

LN229

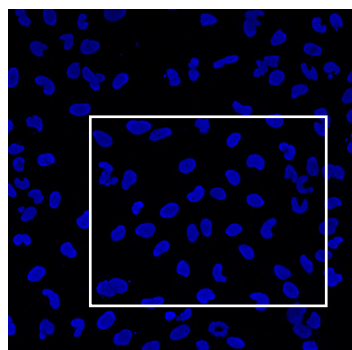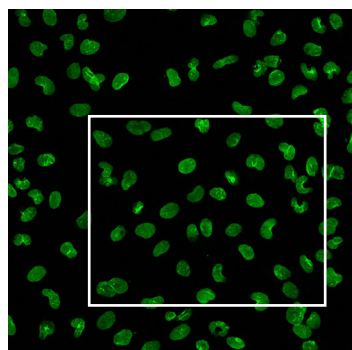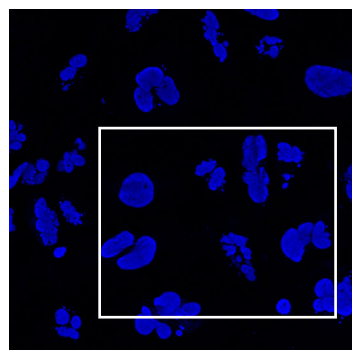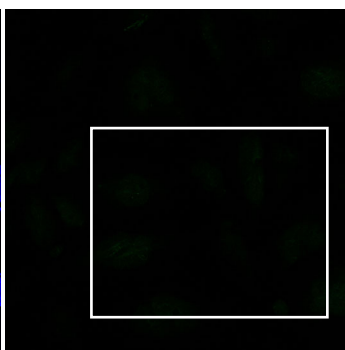

A172

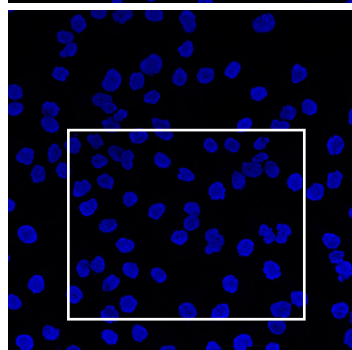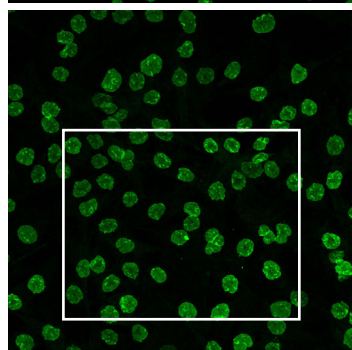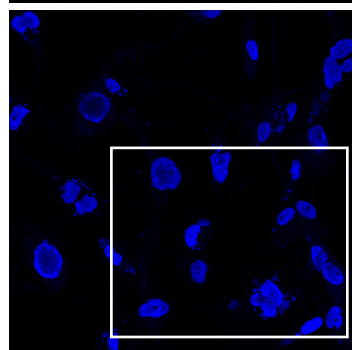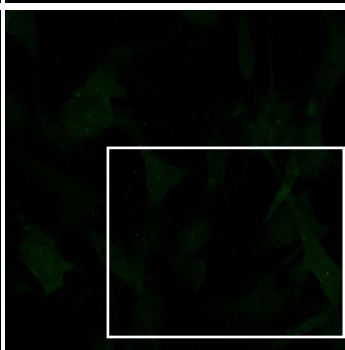

U87

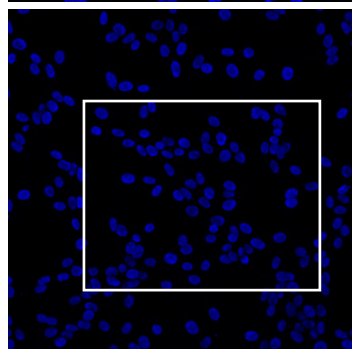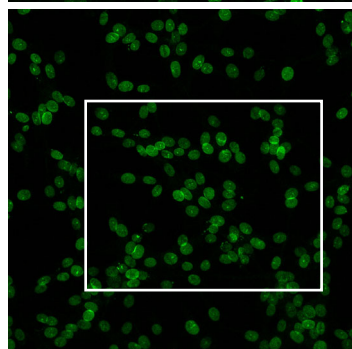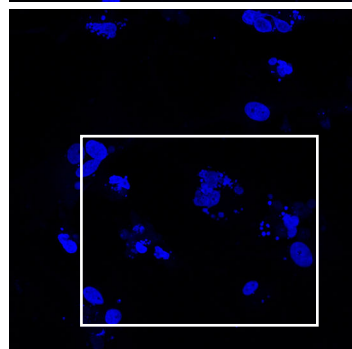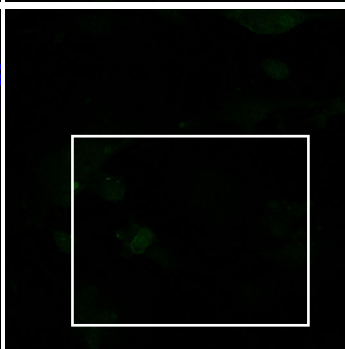

U118

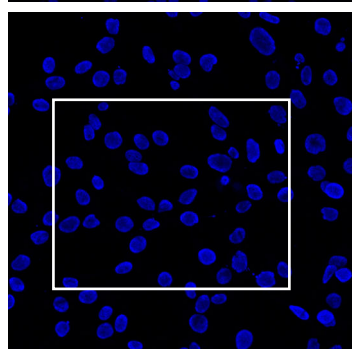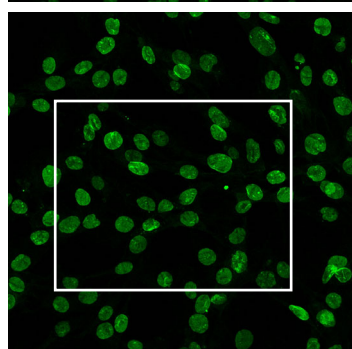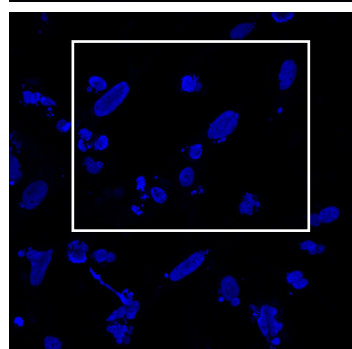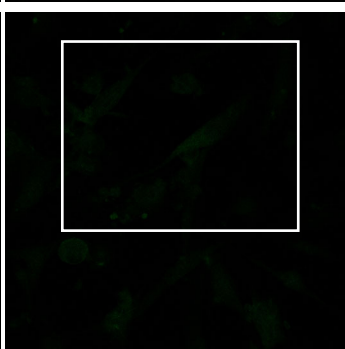

Supplement: Supplementary file 10 — EV figures [file 44321_2025_201_MOESM10_ESM.zip › source data for EV/EV1/EV1 a/pic-Lamin.pdf]

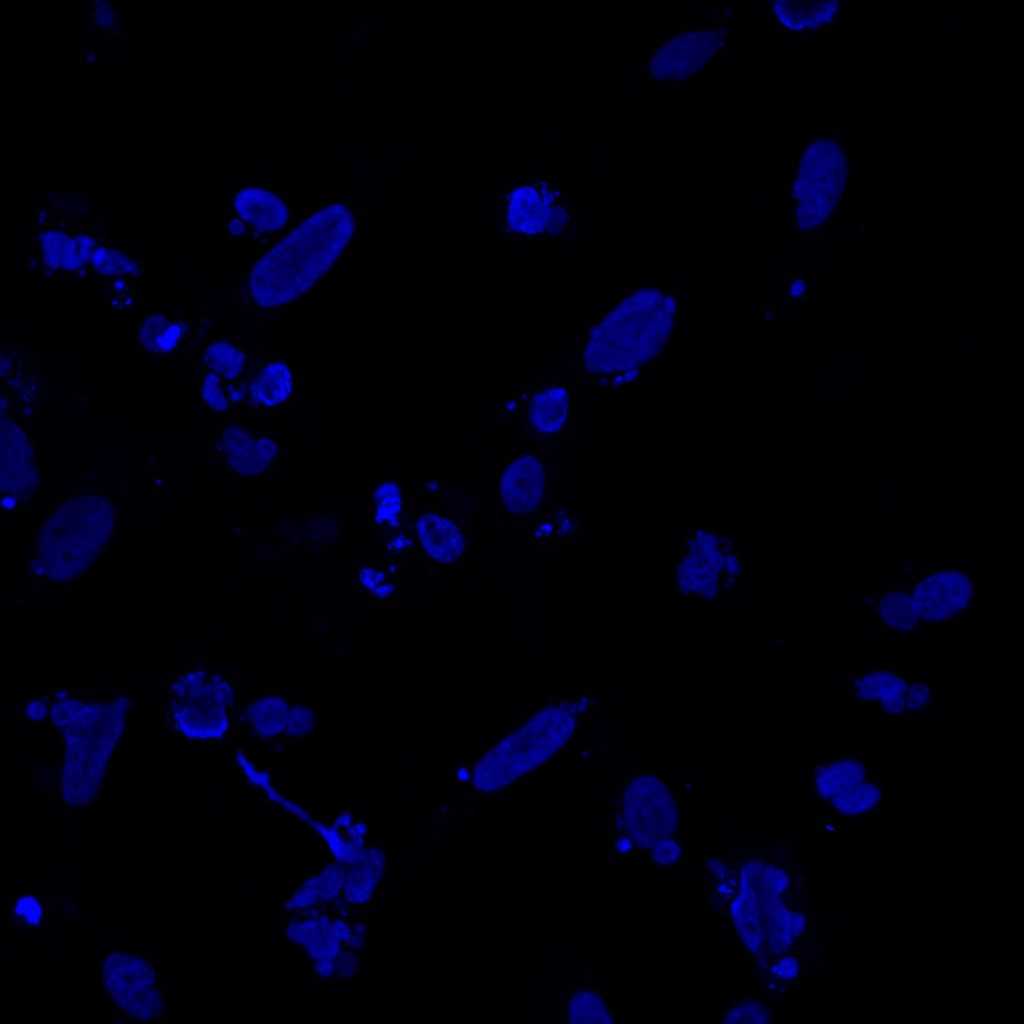

Supplement: Supplementary file 10 — EV figures [file 44321_2025_201_MOESM10_ESM.zip › source data for EV/EV1/EV1 a/U118/U118-IR-5-Image Export-31_c1.jpg]

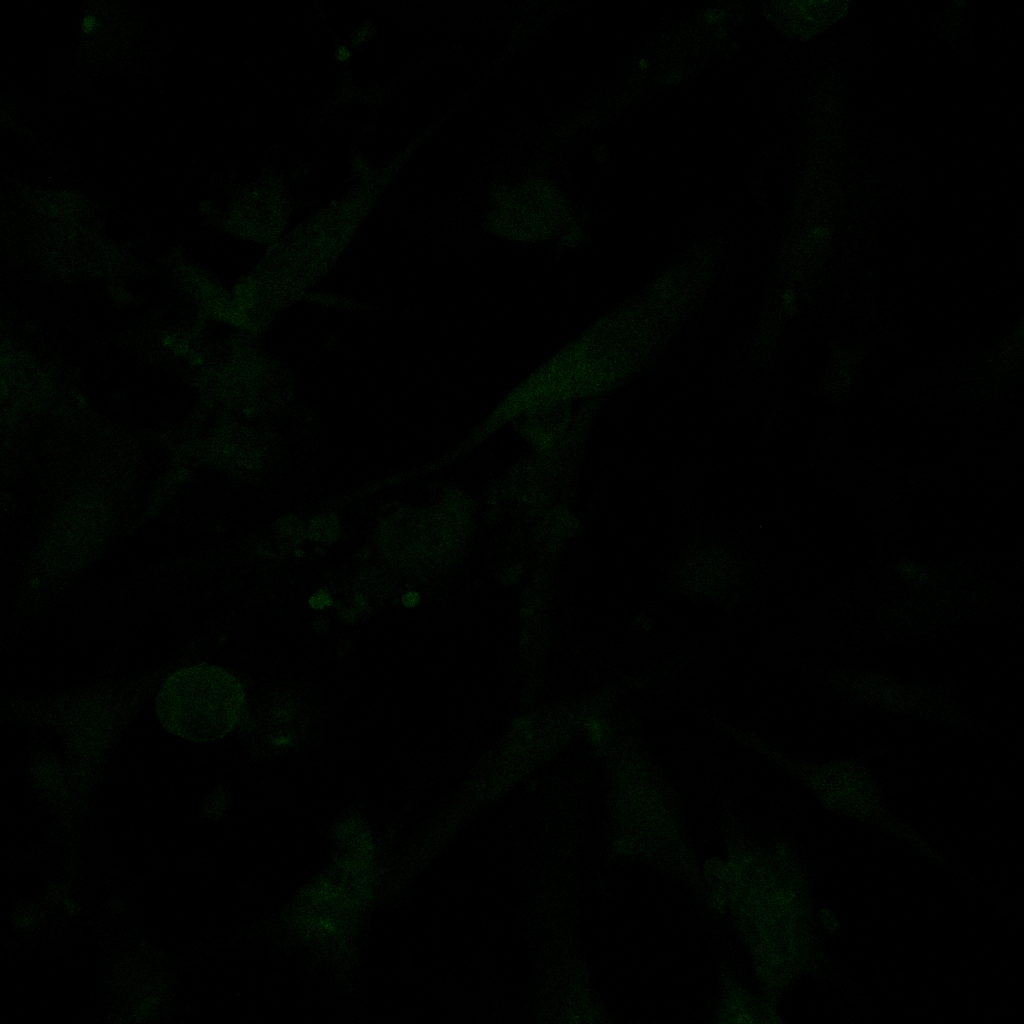

Supplement: Supplementary file 10 — EV figures [file 44321_2025_201_MOESM10_ESM.zip › source data for EV/EV1/EV1 a/U118/U118-IR-5-Image Export-31_c2.jpg]

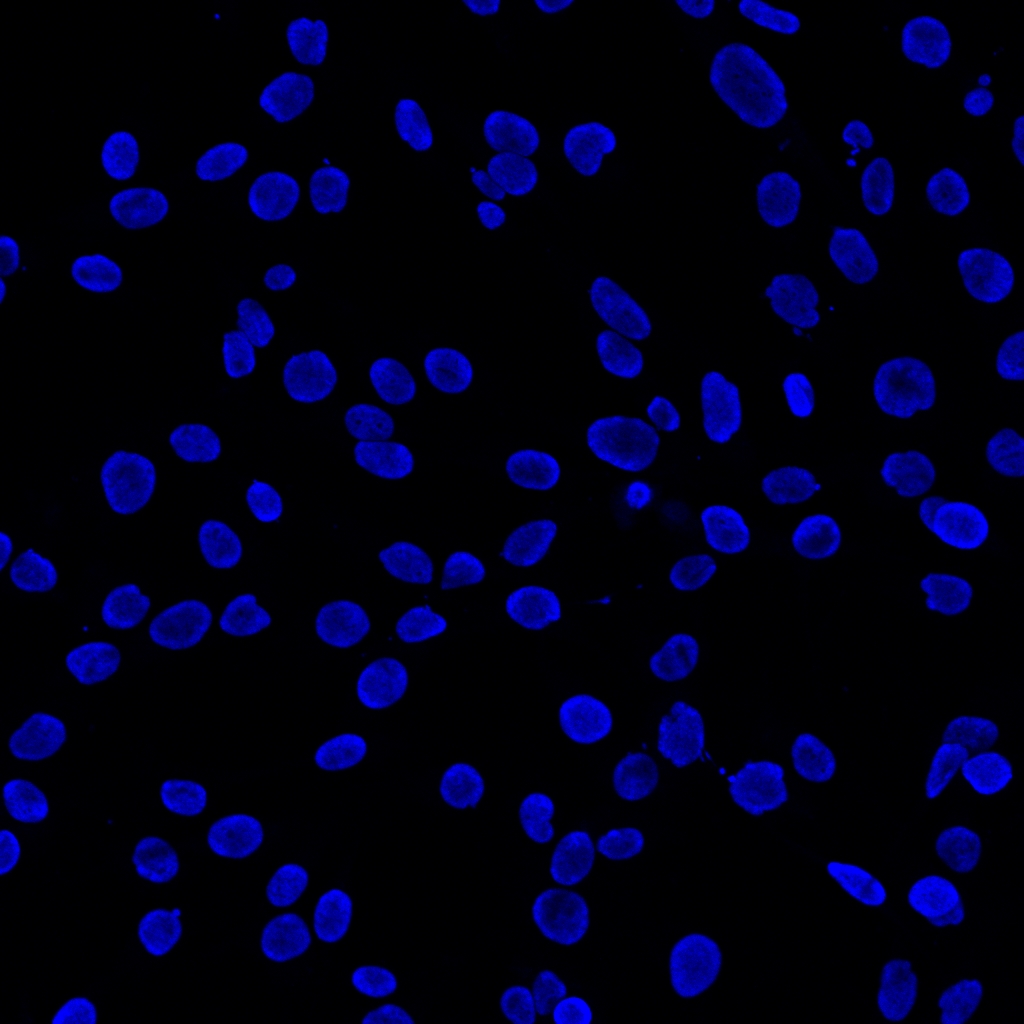

Supplement: Supplementary file 10 — EV figures [file 44321_2025_201_MOESM10_ESM.zip › source data for EV/EV1/EV1 a/U118/U118-mock-1-Image Export-32_c1.jpg]

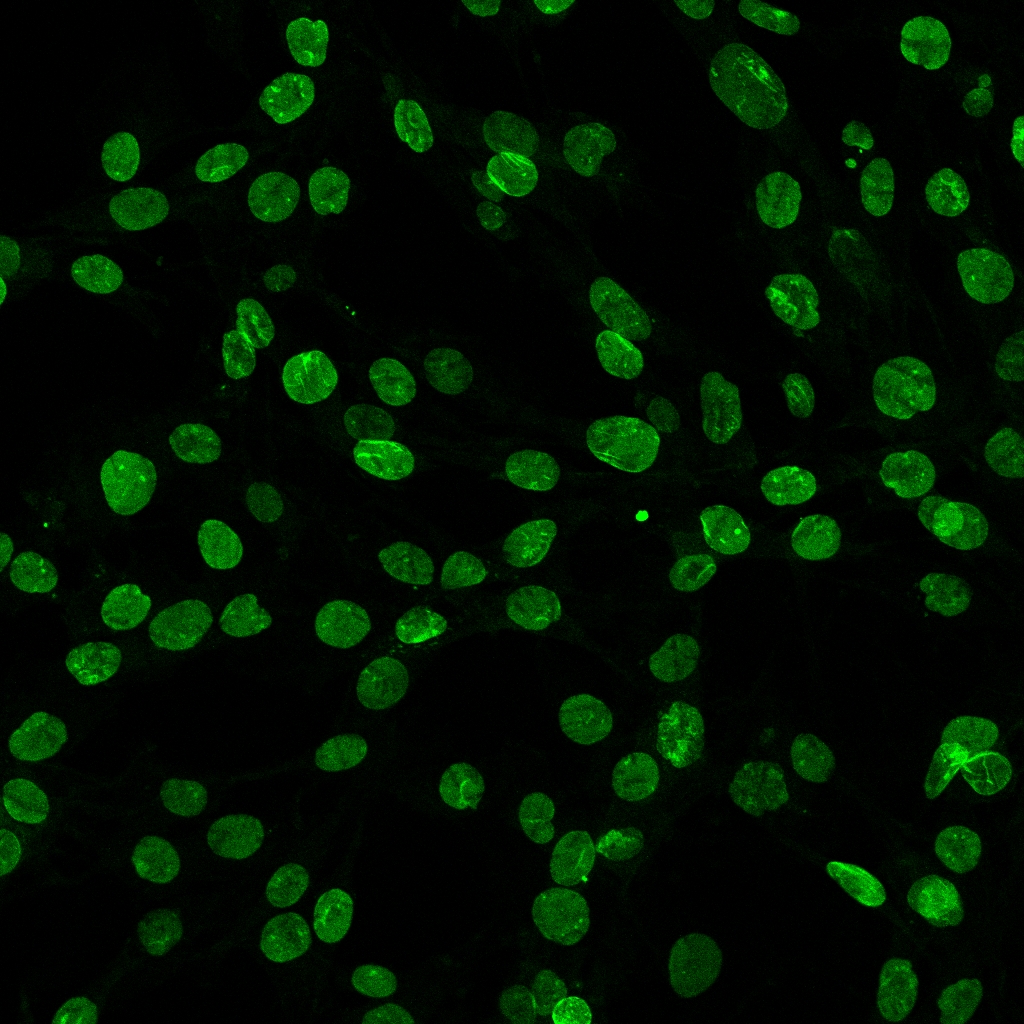

Supplement: Supplementary file 10 — EV figures [file 44321_2025_201_MOESM10_ESM.zip › source data for EV/EV1/EV1 a/U118/U118-mock-1-Image Export-32_c2.jpg]

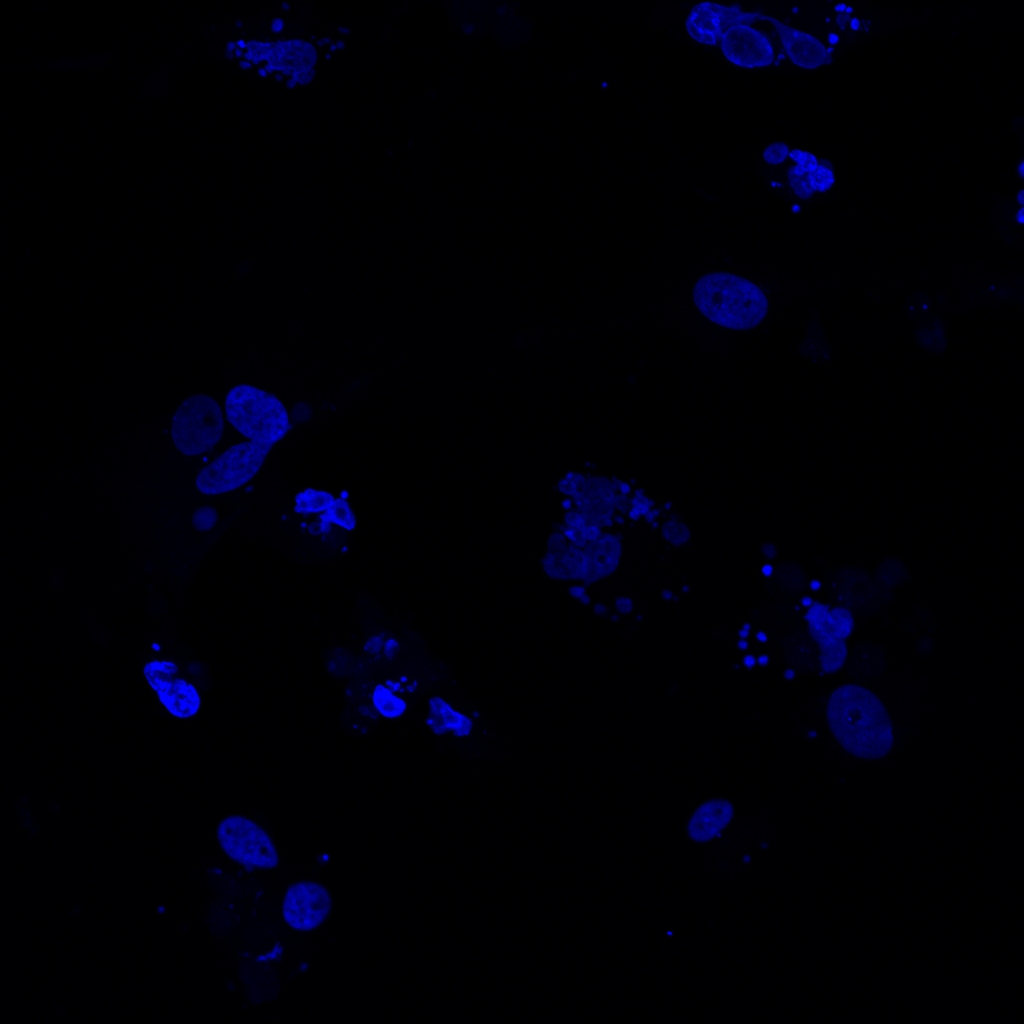

Supplement: Supplementary file 10 — EV figures [file 44321_2025_201_MOESM10_ESM.zip › source data for EV/EV1/EV1 a/U87/U87-IR-4-Image Export-22_c1.jpg]

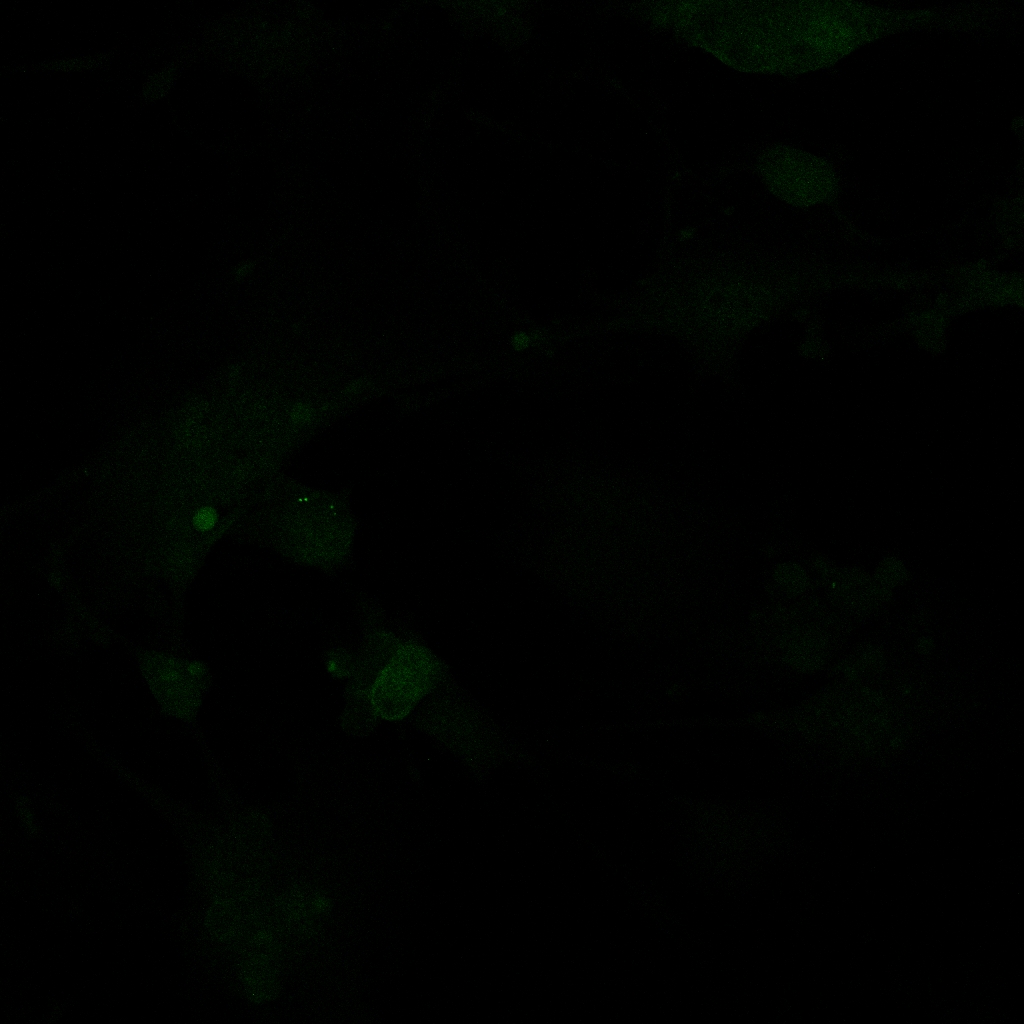

Supplement: Supplementary file 10 — EV figures [file 44321_2025_201_MOESM10_ESM.zip › source data for EV/EV1/EV1 a/U87/U87-IR-4-Image Export-22_c2.jpg]

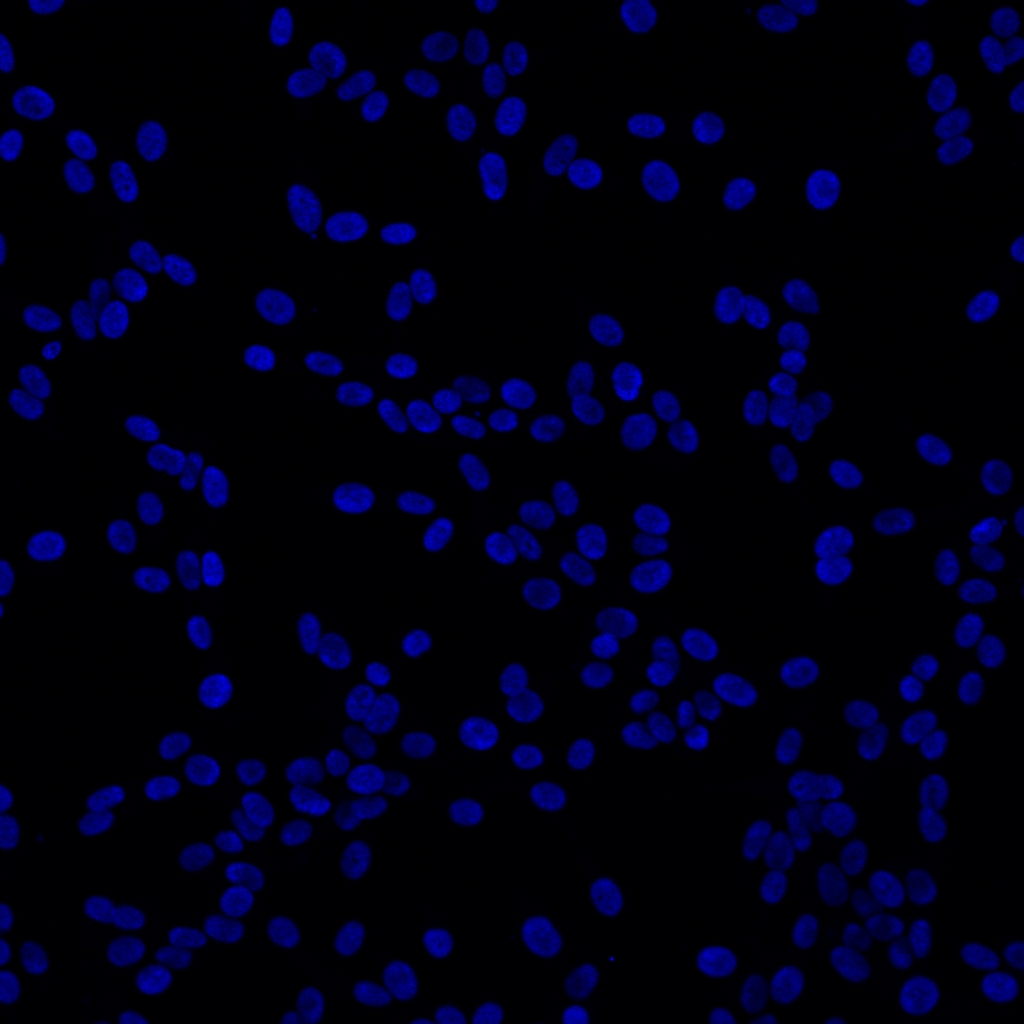

Supplement: Supplementary file 10 — EV figures [file 44321_2025_201_MOESM10_ESM.zip › source data for EV/EV1/EV1 a/U87/U87-mock-1-Image Export-24_c1.jpg]

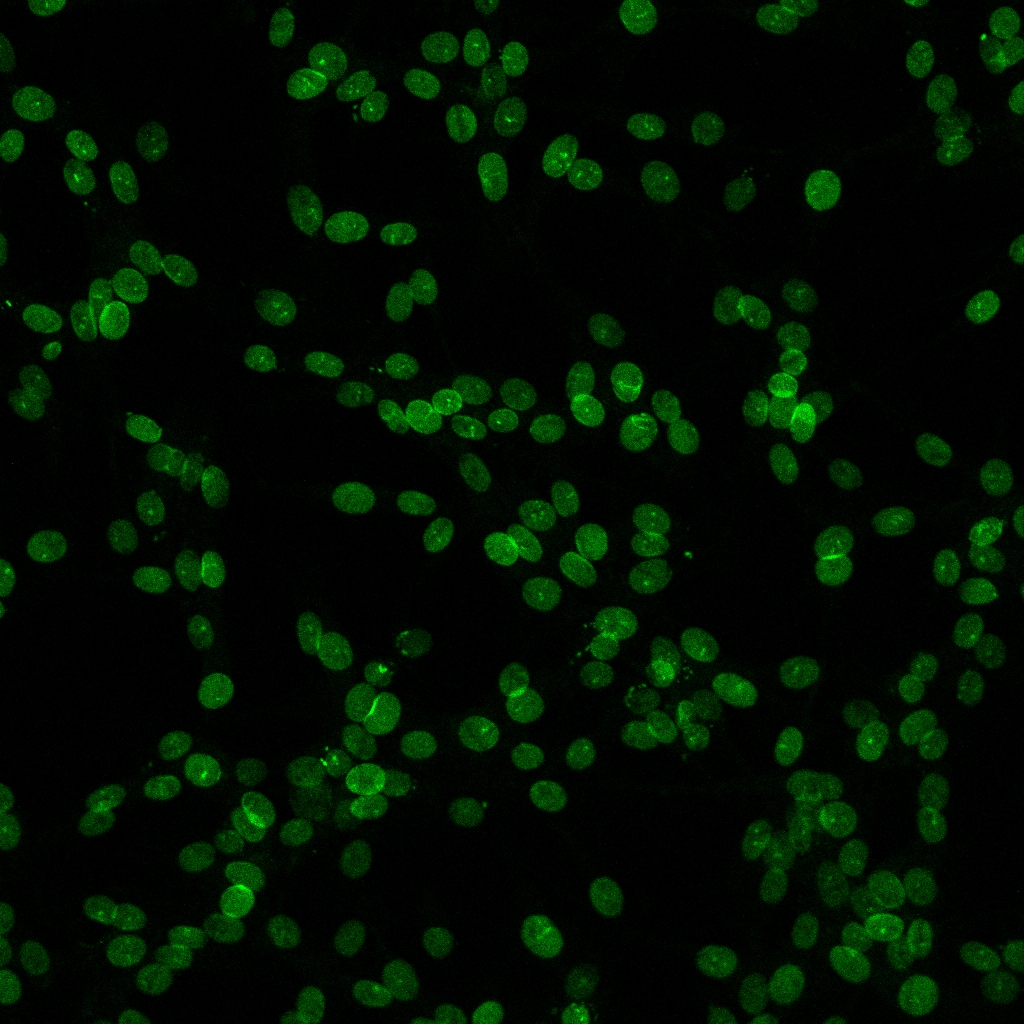

Supplement: Supplementary file 10 — EV figures [file 44321_2025_201_MOESM10_ESM.zip › source data for EV/EV1/EV1 a/U87/U87-mock-1-Image Export-24_c2.jpg]

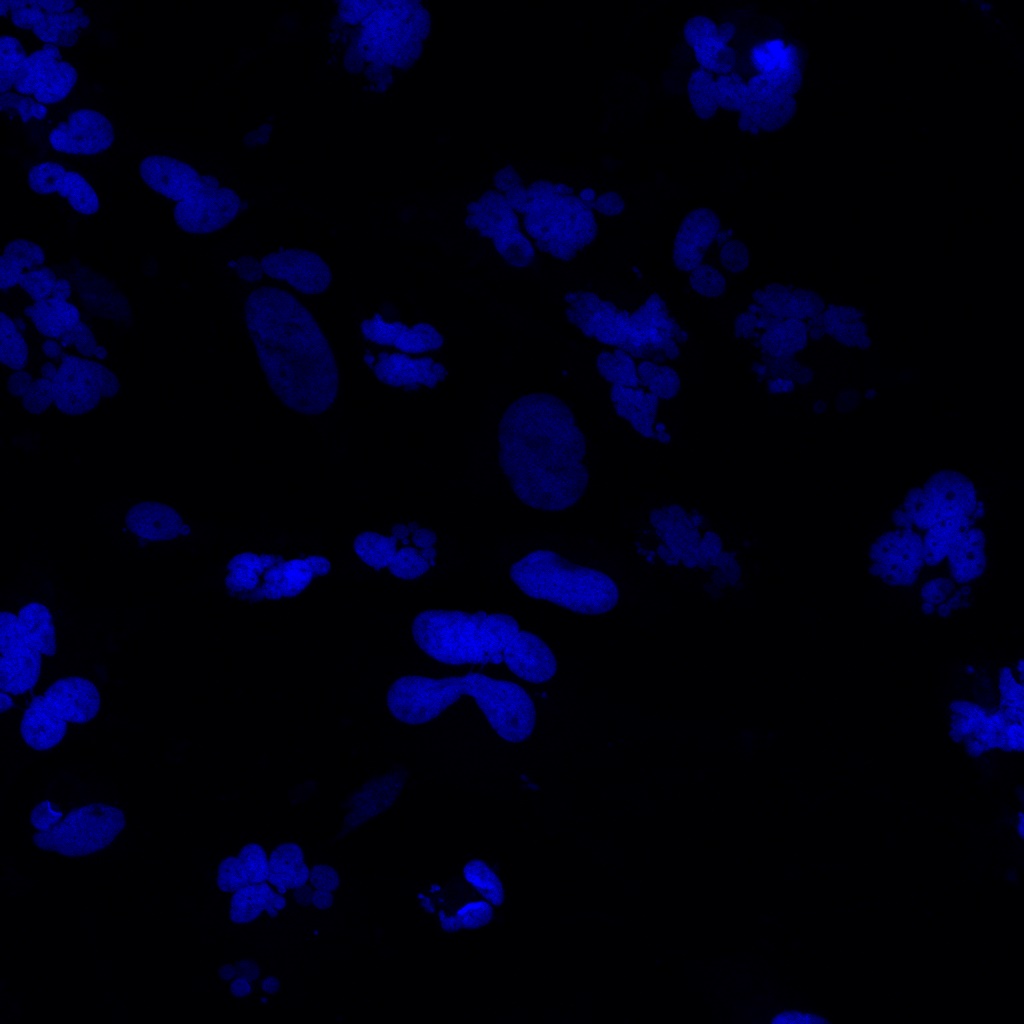

Supplement: Supplementary file 10 — EV figures [file 44321_2025_201_MOESM10_ESM.zip › source data for EV/EV1/EV1 b/229/229-IR-2-Image Export-01_c1.jpg]

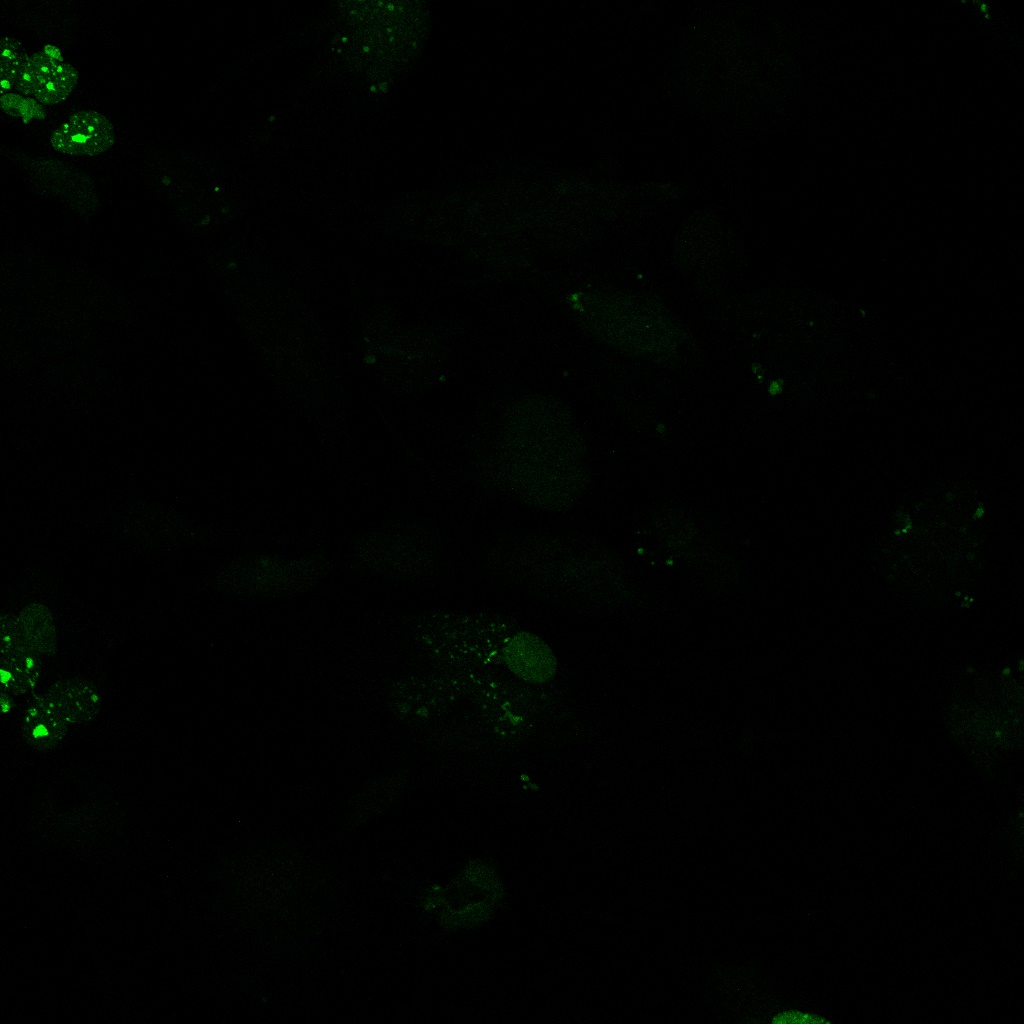

Supplement: Supplementary file 10 — EV figures [file 44321_2025_201_MOESM10_ESM.zip › source data for EV/EV1/EV1 b/229/229-IR-2-Image Export-01_c2.jpg]

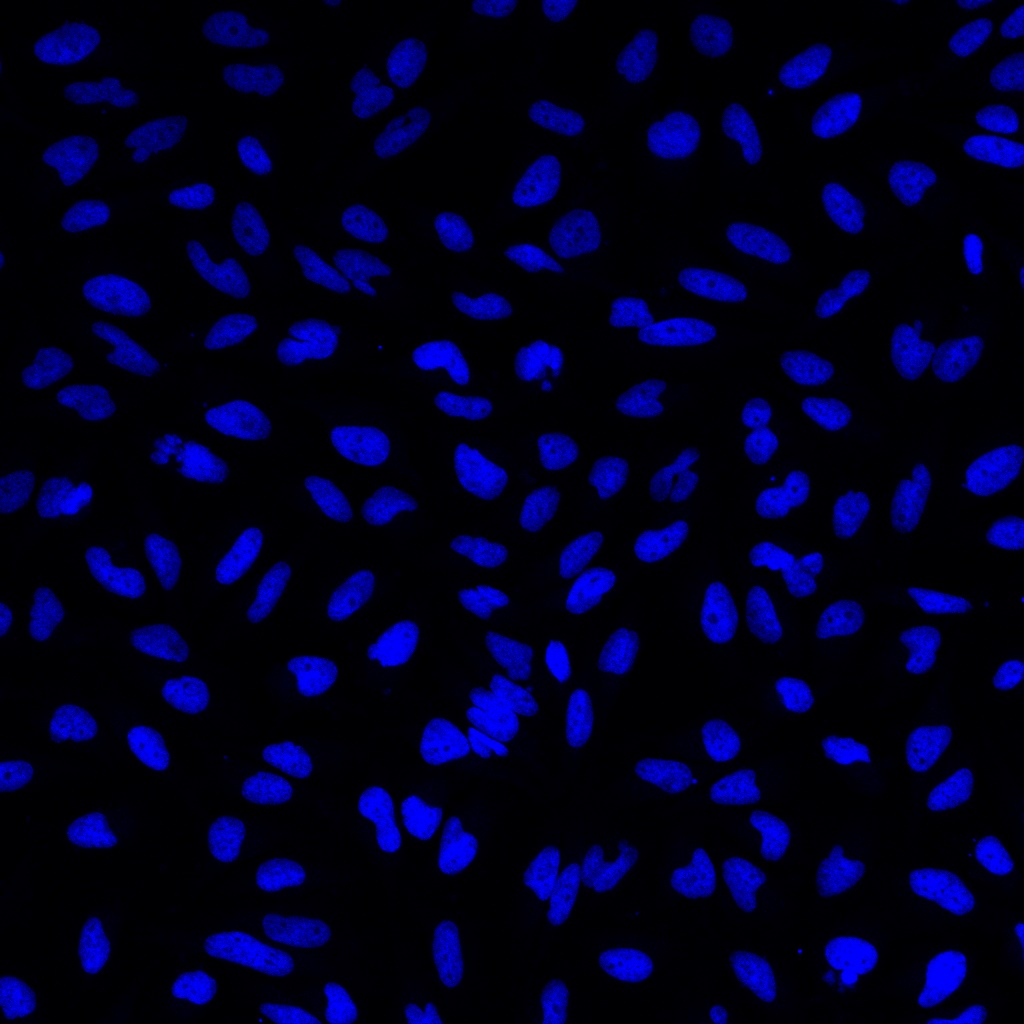

Supplement: Supplementary file 10 — EV figures [file 44321_2025_201_MOESM10_ESM.zip › source data for EV/EV1/EV1 b/229/229-mock-1-Image Export-03_c1.jpg]

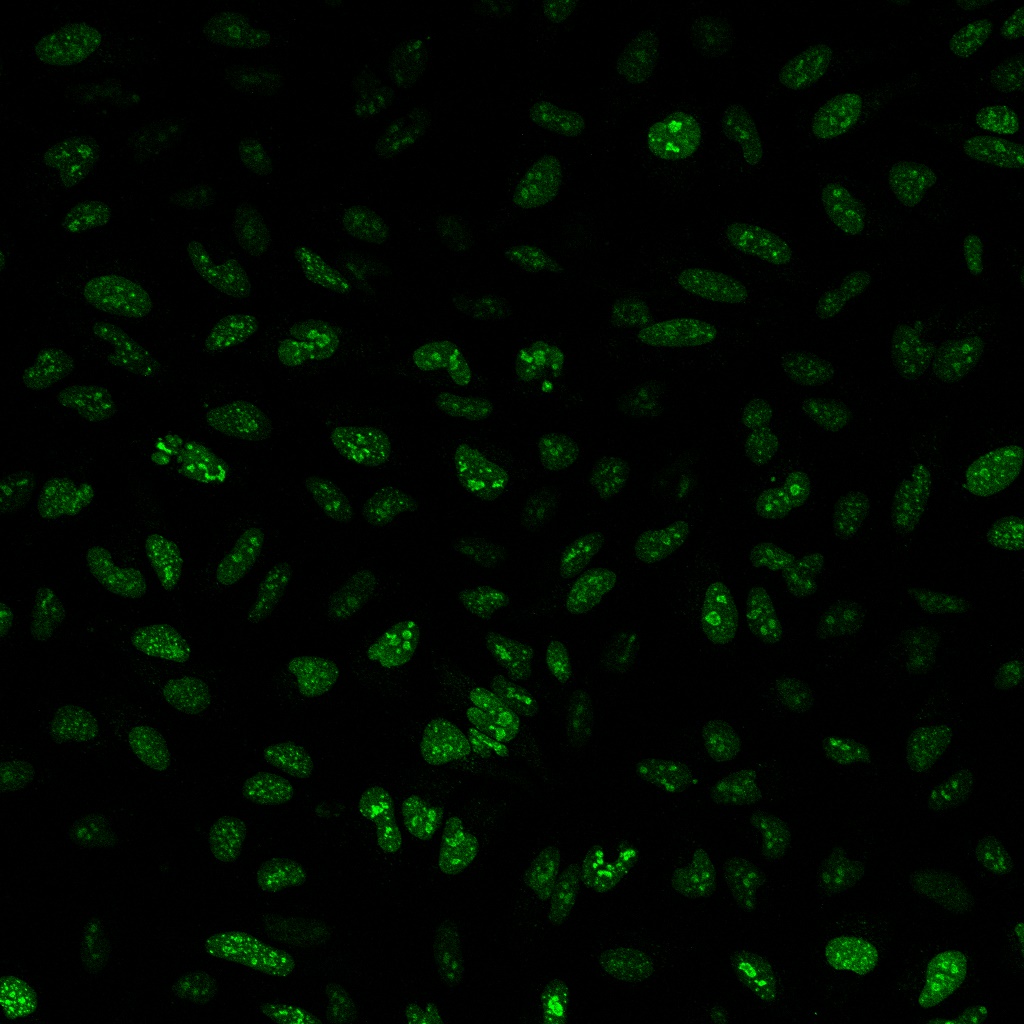

Supplement: Supplementary file 10 — EV figures [file 44321_2025_201_MOESM10_ESM.zip › source data for EV/EV1/EV1 b/229/229-mock-1-Image Export-03_c2.jpg]

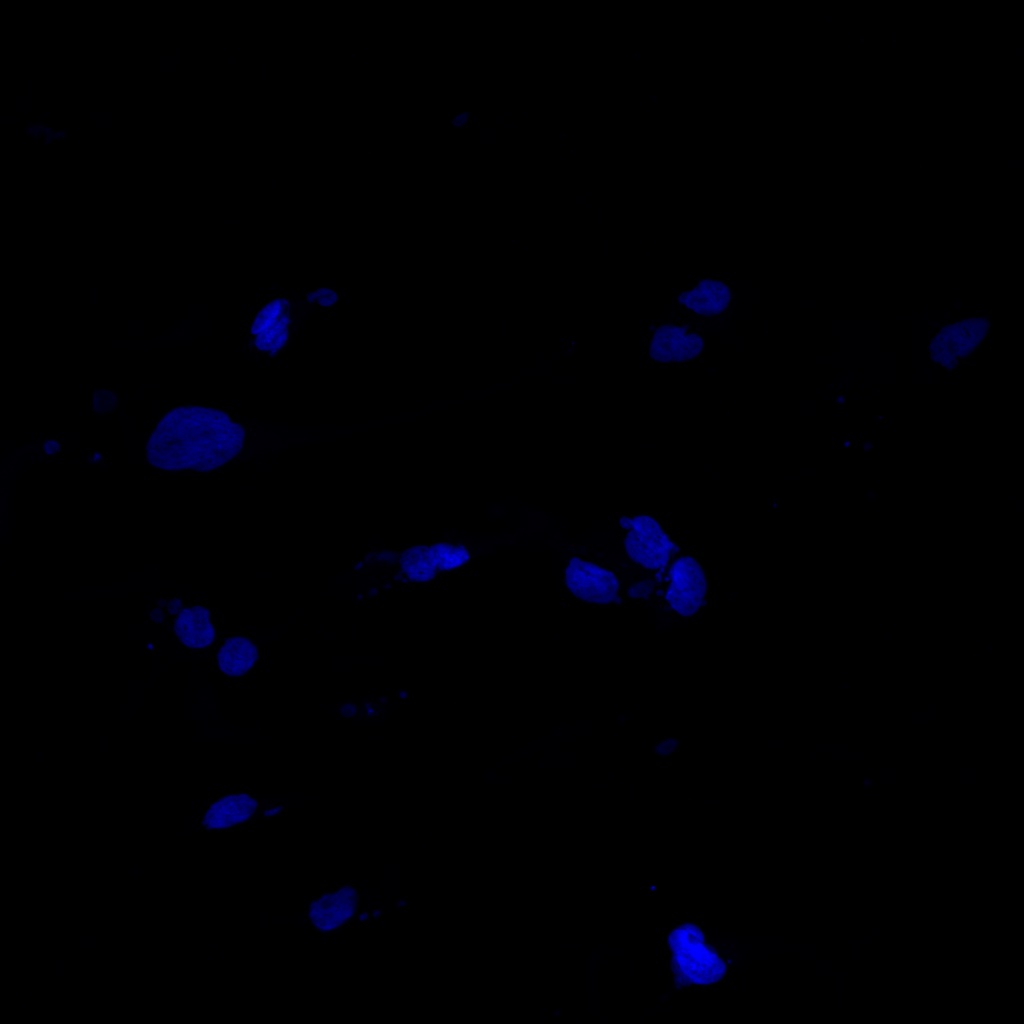

Supplement: Supplementary file 10 — EV figures [file 44321_2025_201_MOESM10_ESM.zip › source data for EV/EV1/EV1 b/A172/A172-IR-4-Image Export-09_c1.jpg]

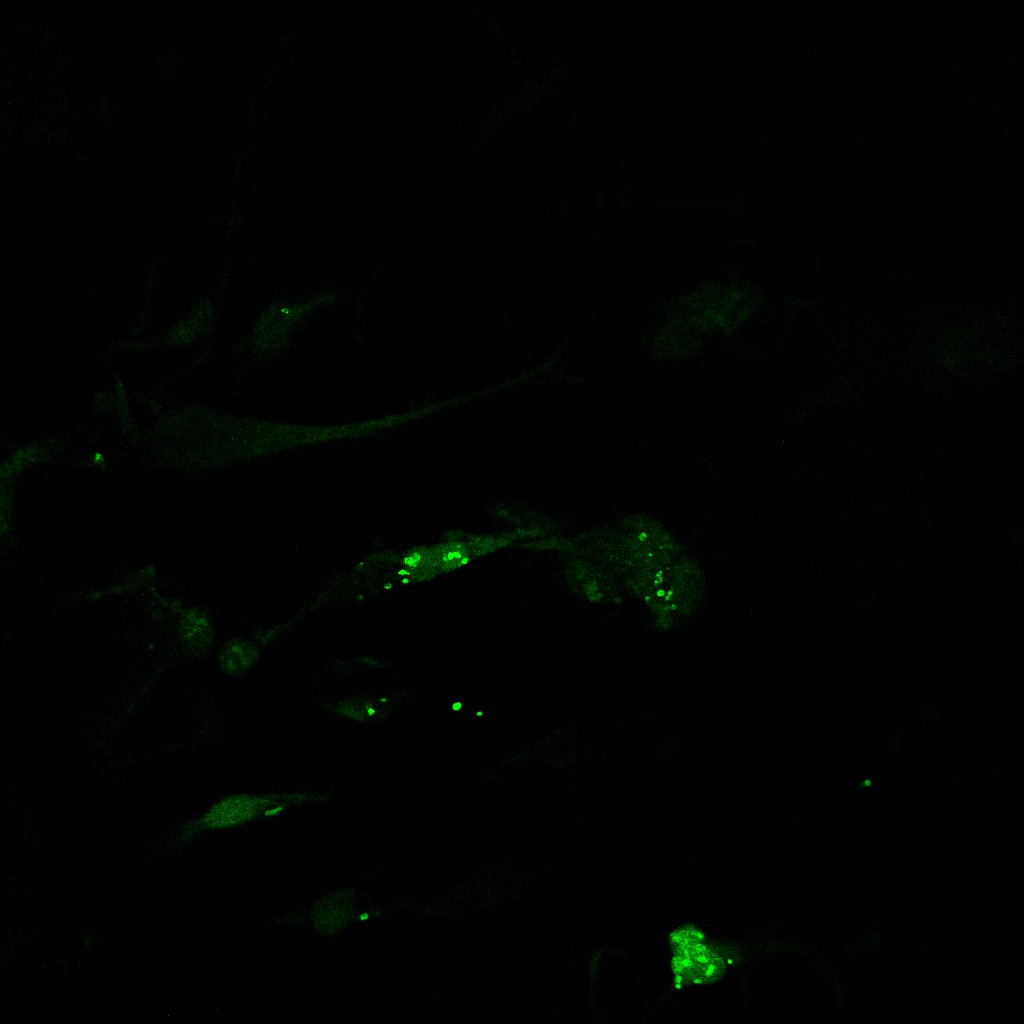

Supplement: Supplementary file 10 — EV figures [file 44321_2025_201_MOESM10_ESM.zip › source data for EV/EV1/EV1 b/A172/A172-IR-4-Image Export-09_c2.jpg]

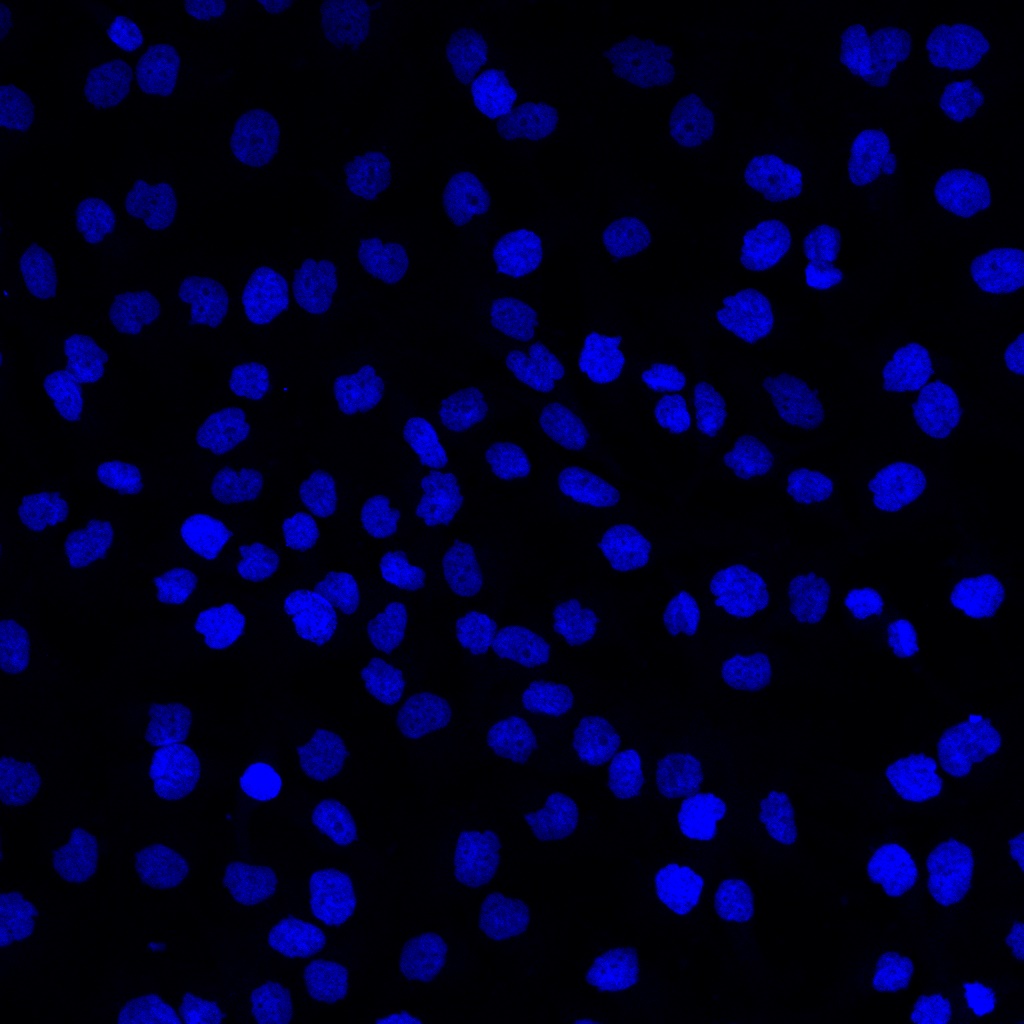

Supplement: Supplementary file 10 — EV figures [file 44321_2025_201_MOESM10_ESM.zip › source data for EV/EV1/EV1 b/A172/A172-mock-1-Image Export-10_c1.jpg]

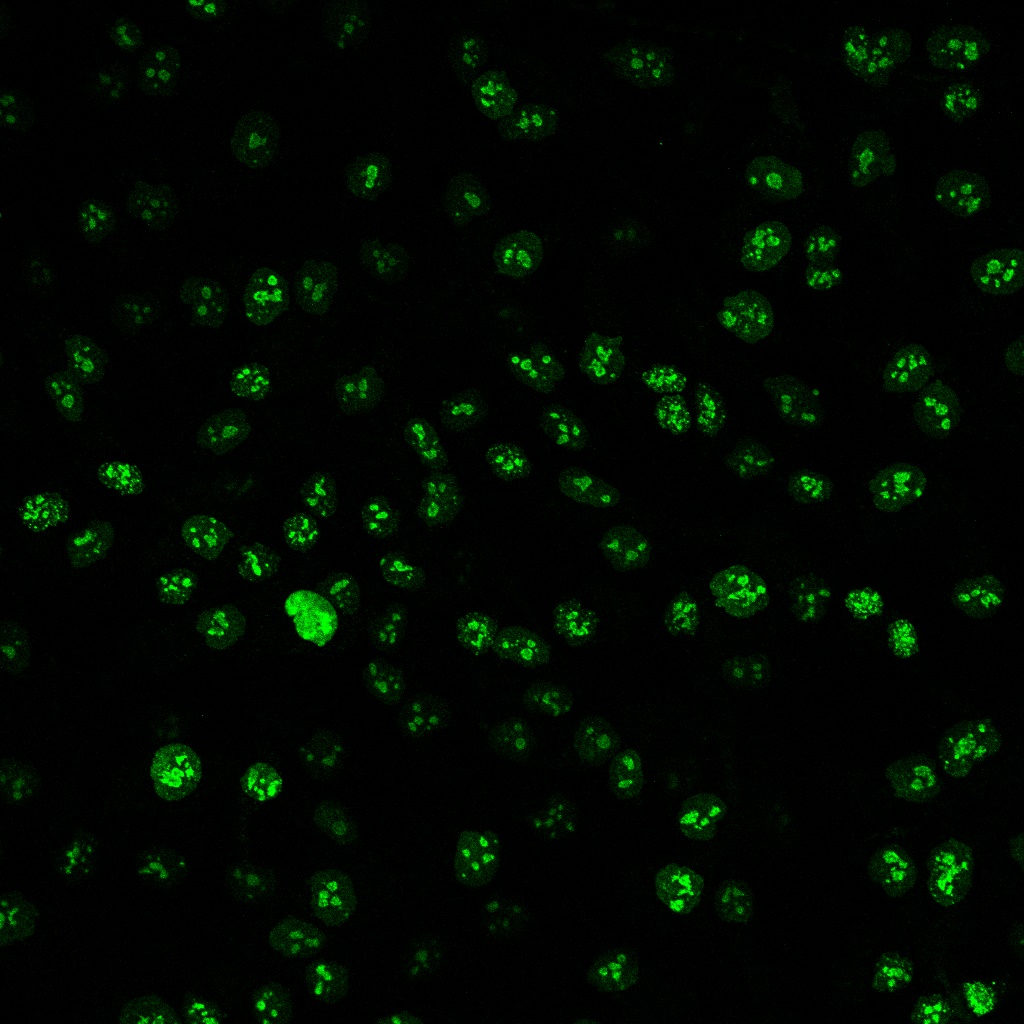

Supplement: Supplementary file 10 — EV figures [file 44321_2025_201_MOESM10_ESM.zip › source data for EV/EV1/EV1 b/A172/A172-mock-1-Image Export-10_c2.jpg]

EV1  
b

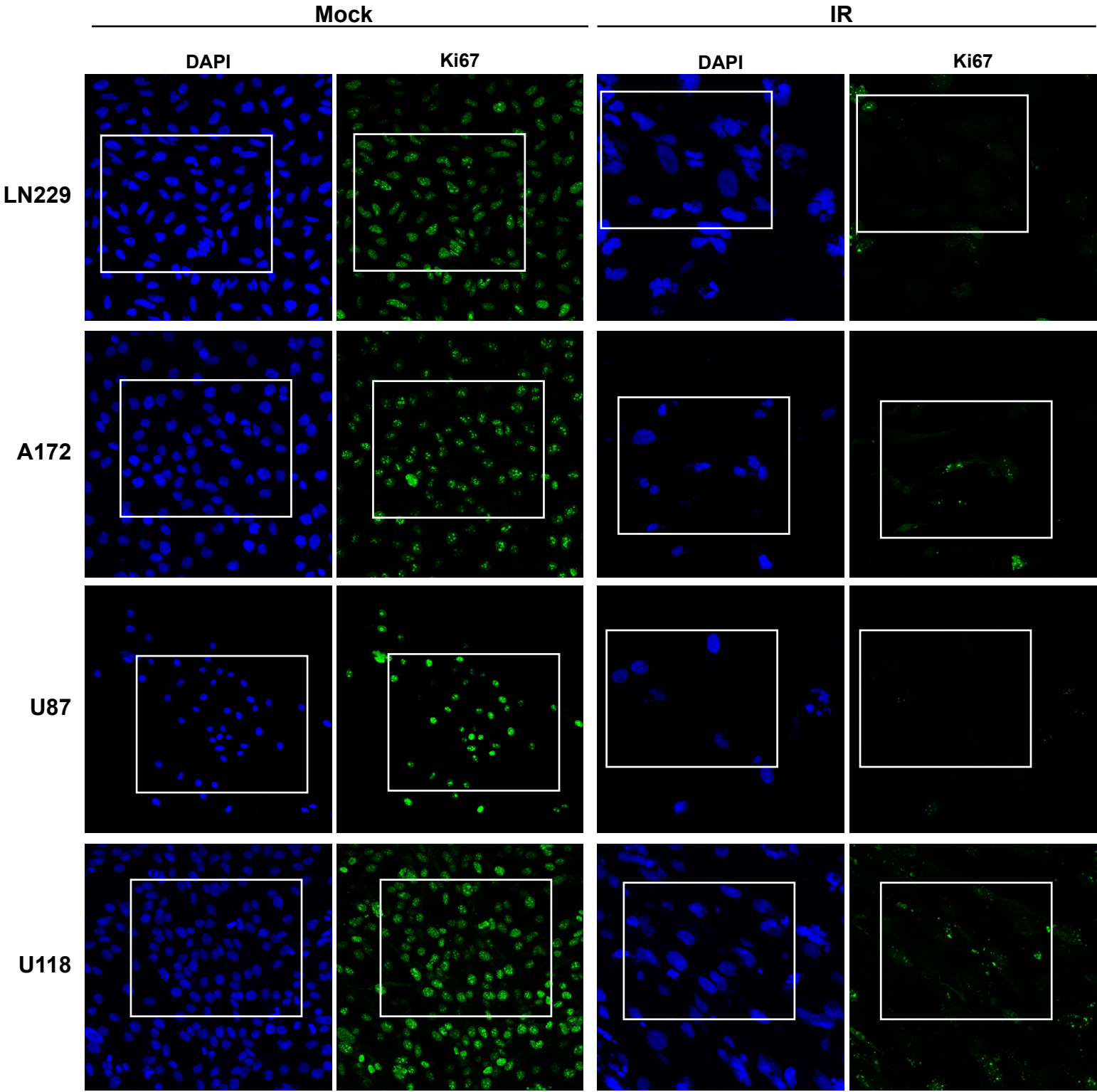

Supplement: Supplementary file 10 — EV figures [file 44321_2025_201_MOESM10_ESM.zip › source data for EV/EV1/EV1 b/pic-ki67.pdf]

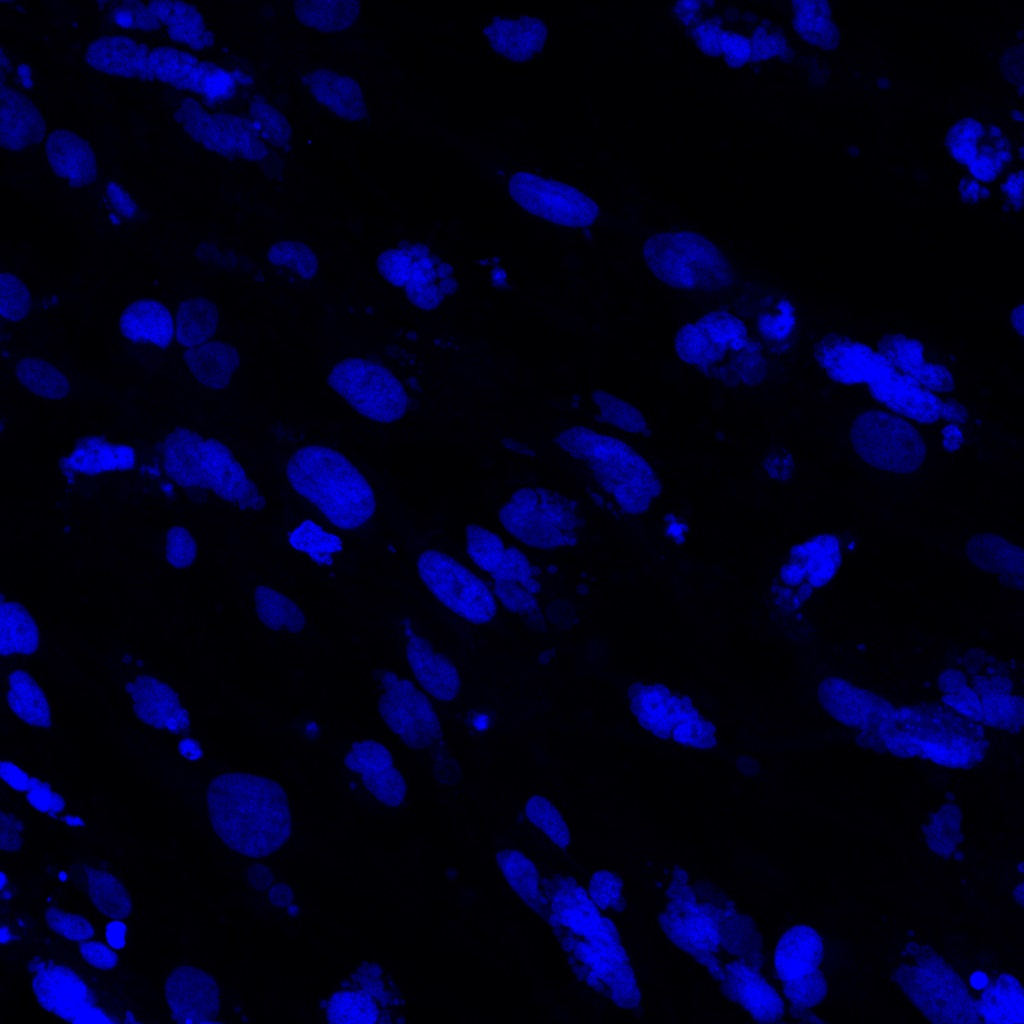

Supplement: Supplementary file 10 — EV figures [file 44321_2025_201_MOESM10_ESM.zip › source data for EV/EV1/EV1 b/U118/U118-IR-3-Image Export-21_c1.jpg]

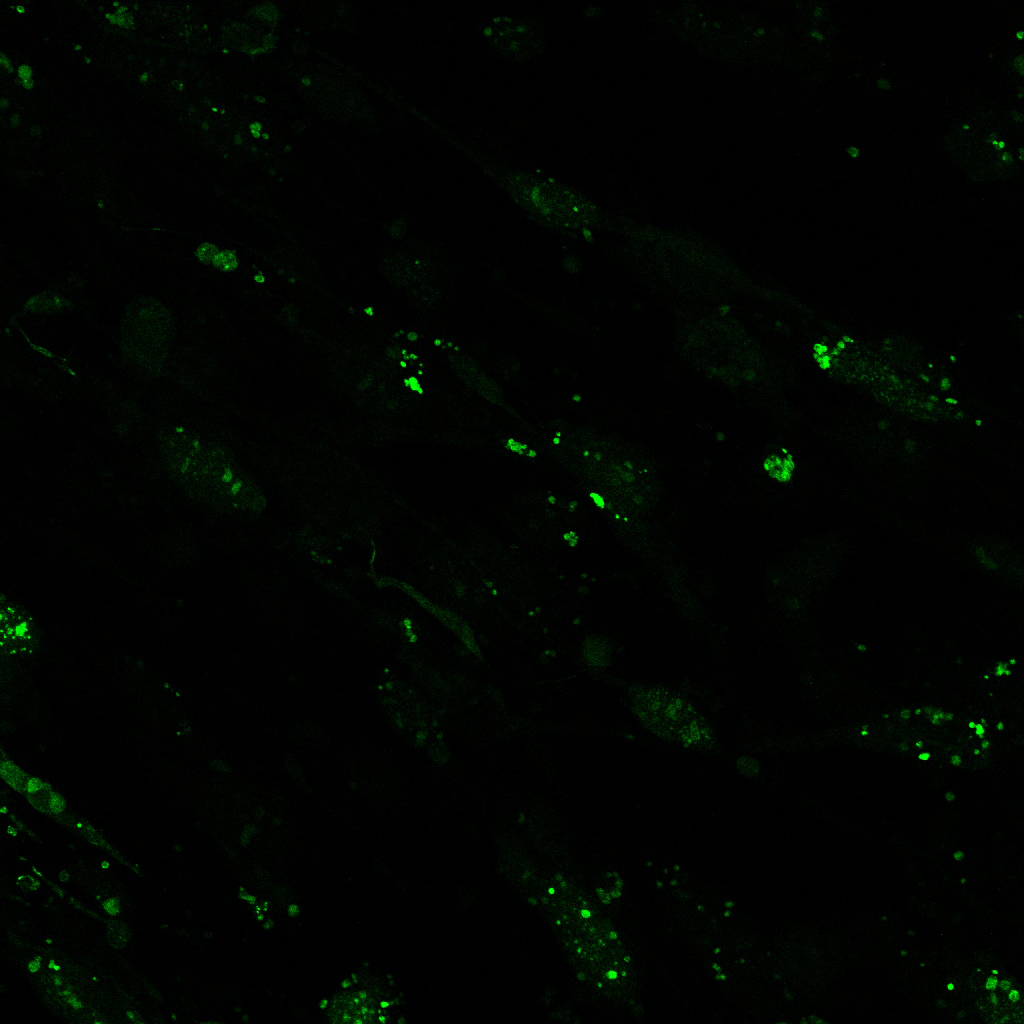

Supplement: Supplementary file 10 — EV figures [file 44321_2025_201_MOESM10_ESM.zip › source data for EV/EV1/EV1 b/U118/U118-IR-3-Image Export-21_c2.jpg]

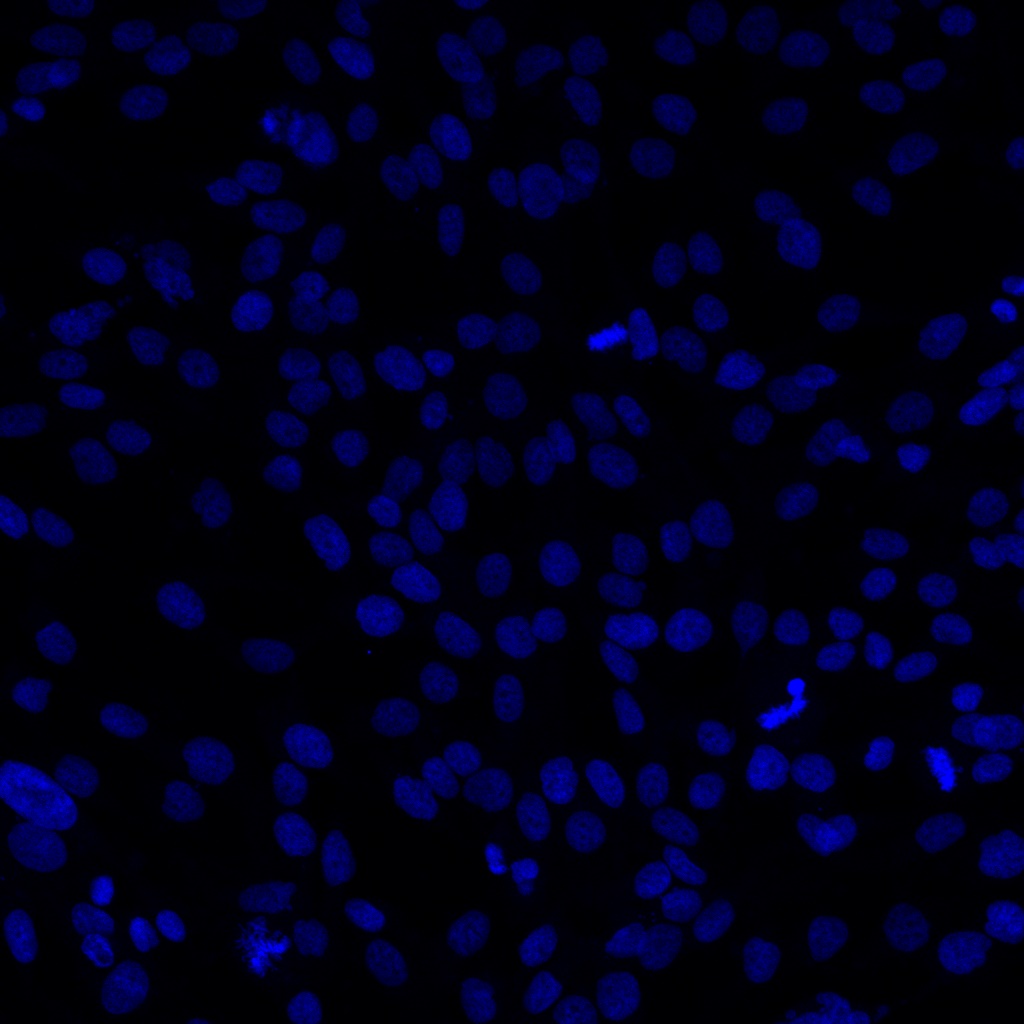

Supplement: Supplementary file 10 — EV figures [file 44321_2025_201_MOESM10_ESM.zip › source data for EV/EV1/EV1 b/U118/U118-mock-2-Image Export-23_c1.jpg]

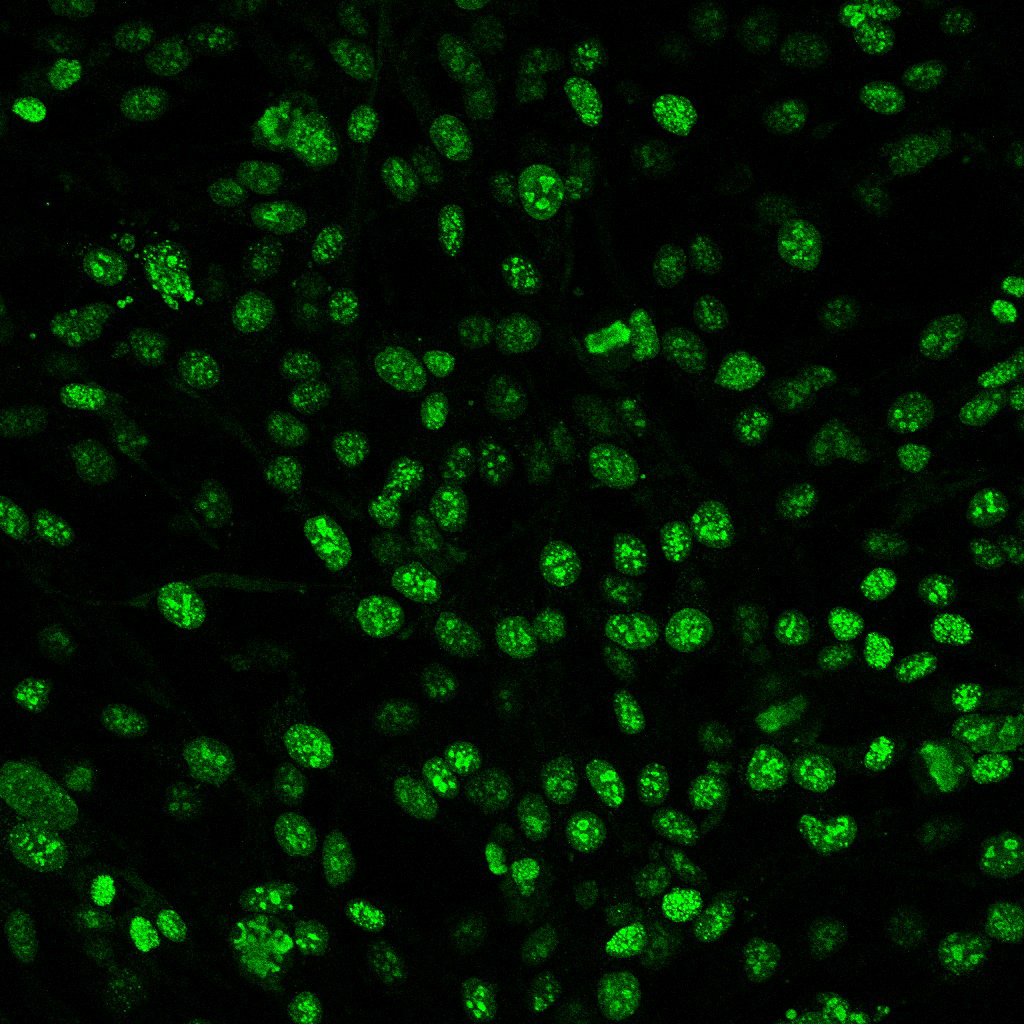

Supplement: Supplementary file 10 — EV figures [file 44321_2025_201_MOESM10_ESM.zip › source data for EV/EV1/EV1 b/U118/U118-mock-2-Image Export-23_c2.jpg]

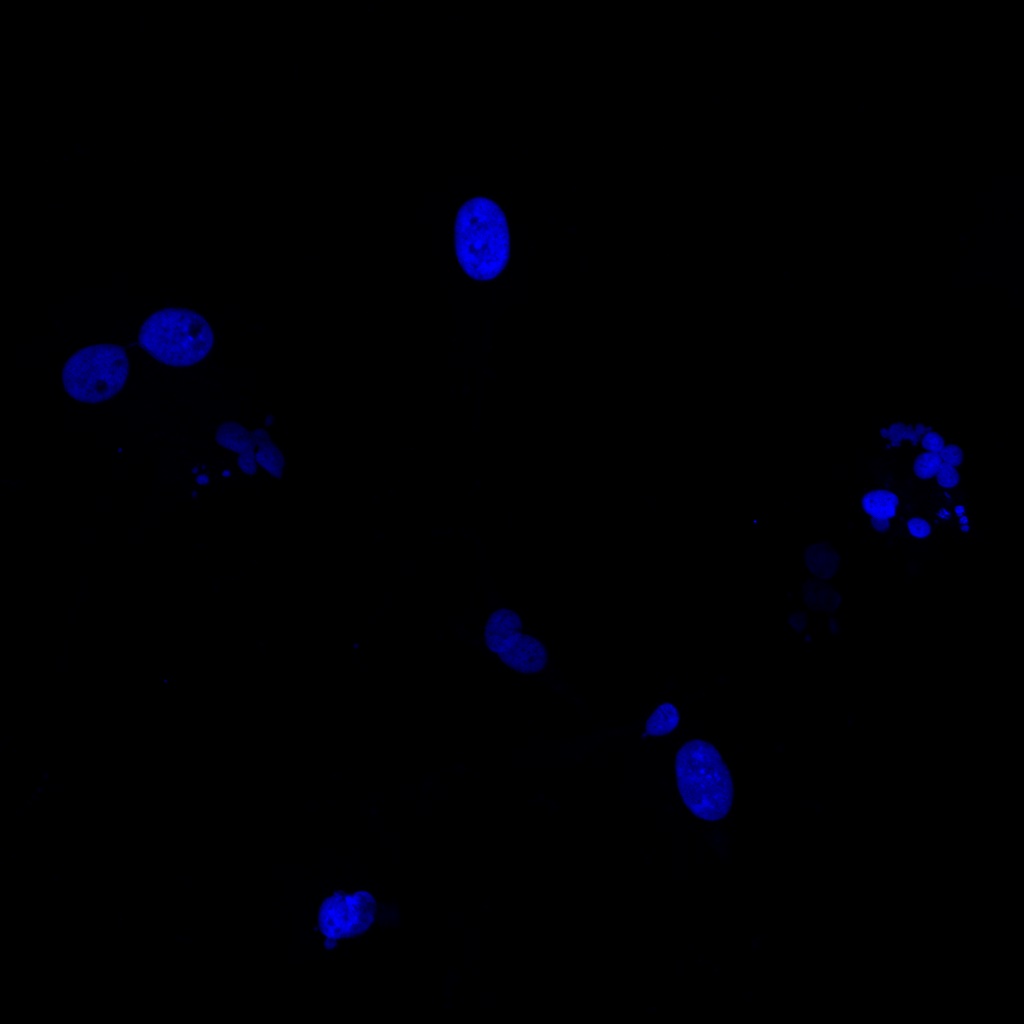

Supplement: Supplementary file 10 — EV figures [file 44321_2025_201_MOESM10_ESM.zip › source data for EV/EV1/EV1 b/U87/U87-IR-2-Image Export-14_c1.jpg]

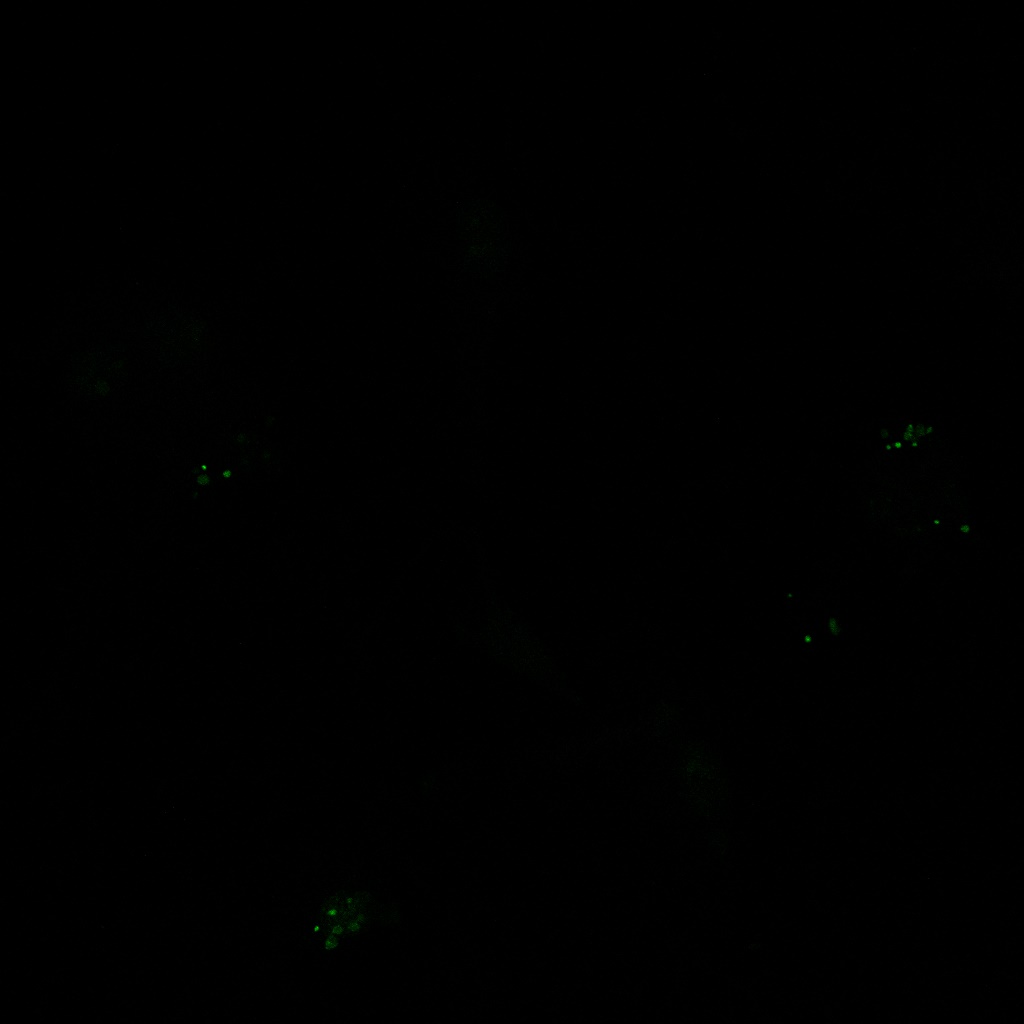

Supplement: Supplementary file 10 — EV figures [file 44321_2025_201_MOESM10_ESM.zip › source data for EV/EV1/EV1 b/U87/U87-IR-2-Image Export-14_c2.jpg]

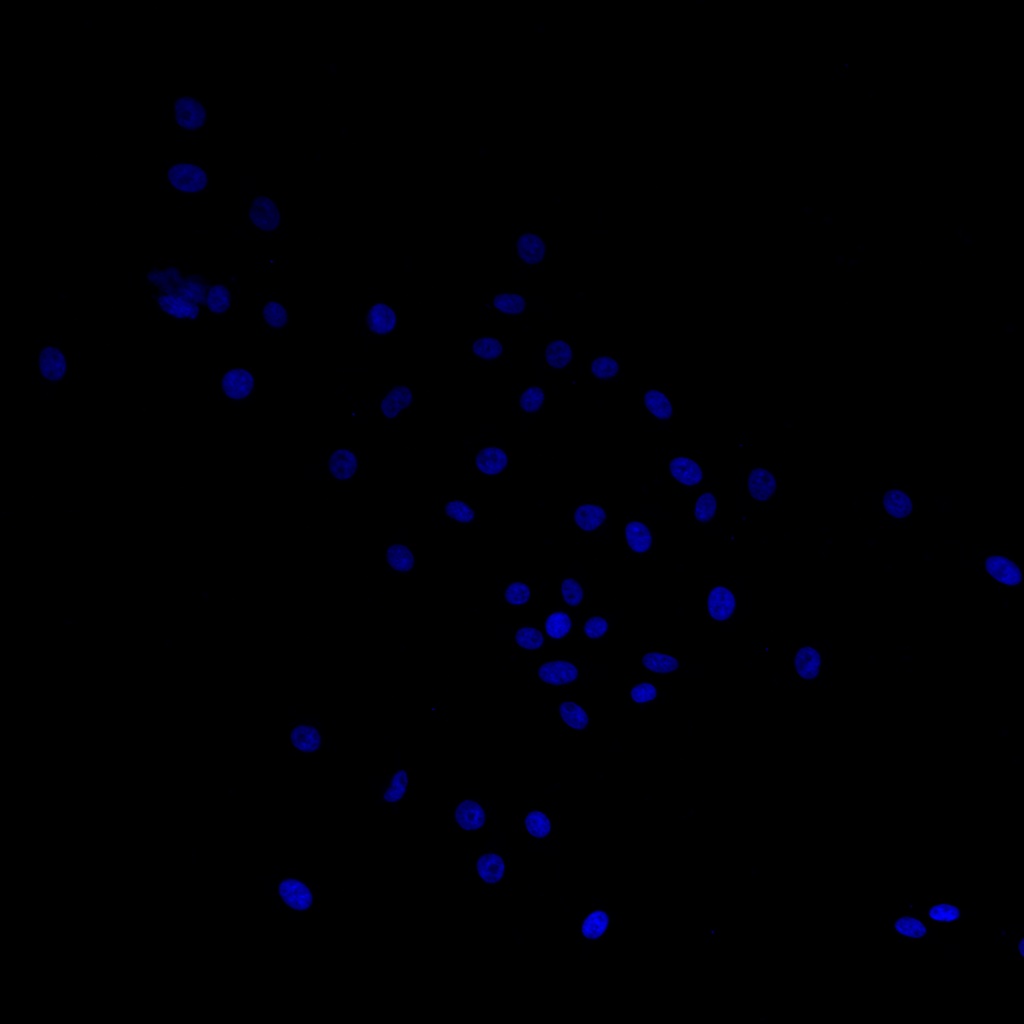

Supplement: Supplementary file 10 — EV figures [file 44321_2025_201_MOESM10_ESM.zip › source data for EV/EV1/EV1 b/U87/U87-mock-3-Image Export-18_c1.jpg]

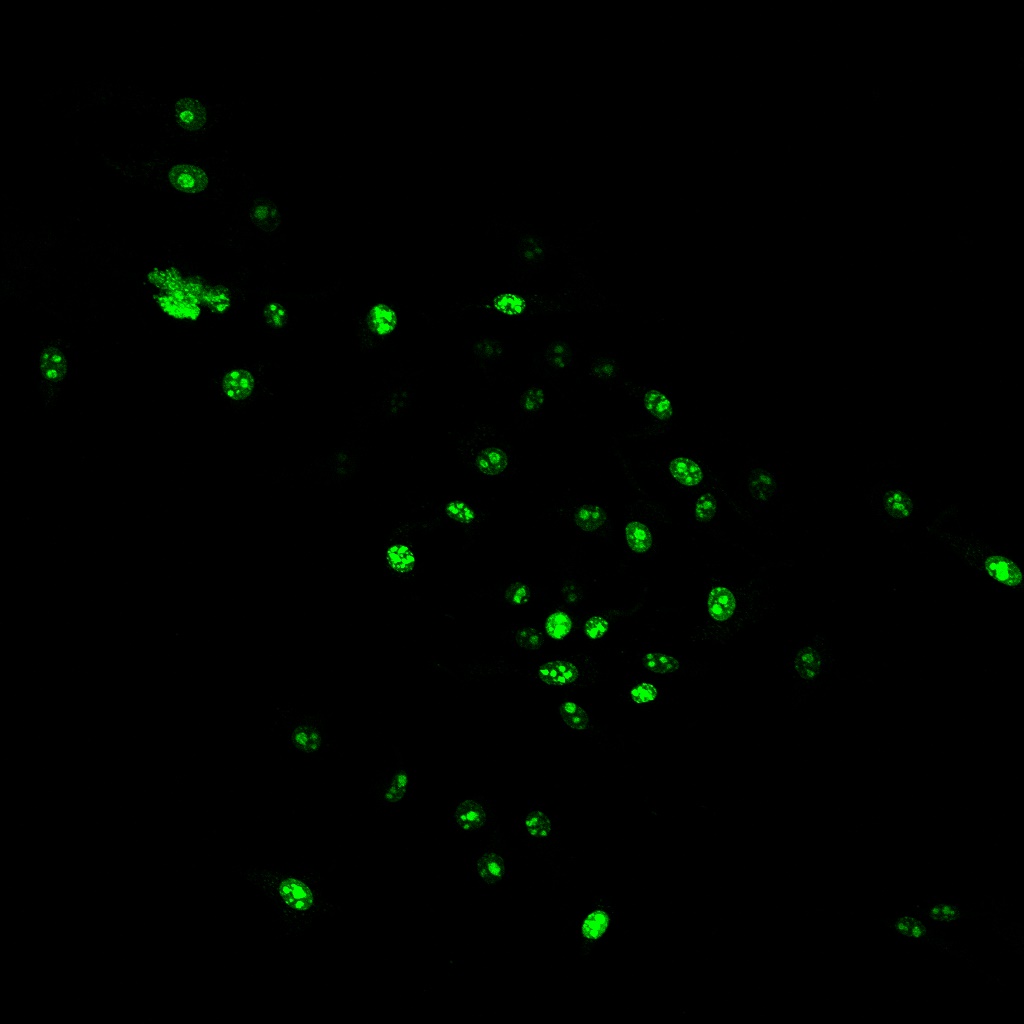

Supplement: Supplementary file 10 — EV figures [file 44321_2025_201_MOESM10_ESM.zip › source data for EV/EV1/EV1 b/U87/U87-mock-3-Image Export-18_c2.jpg]

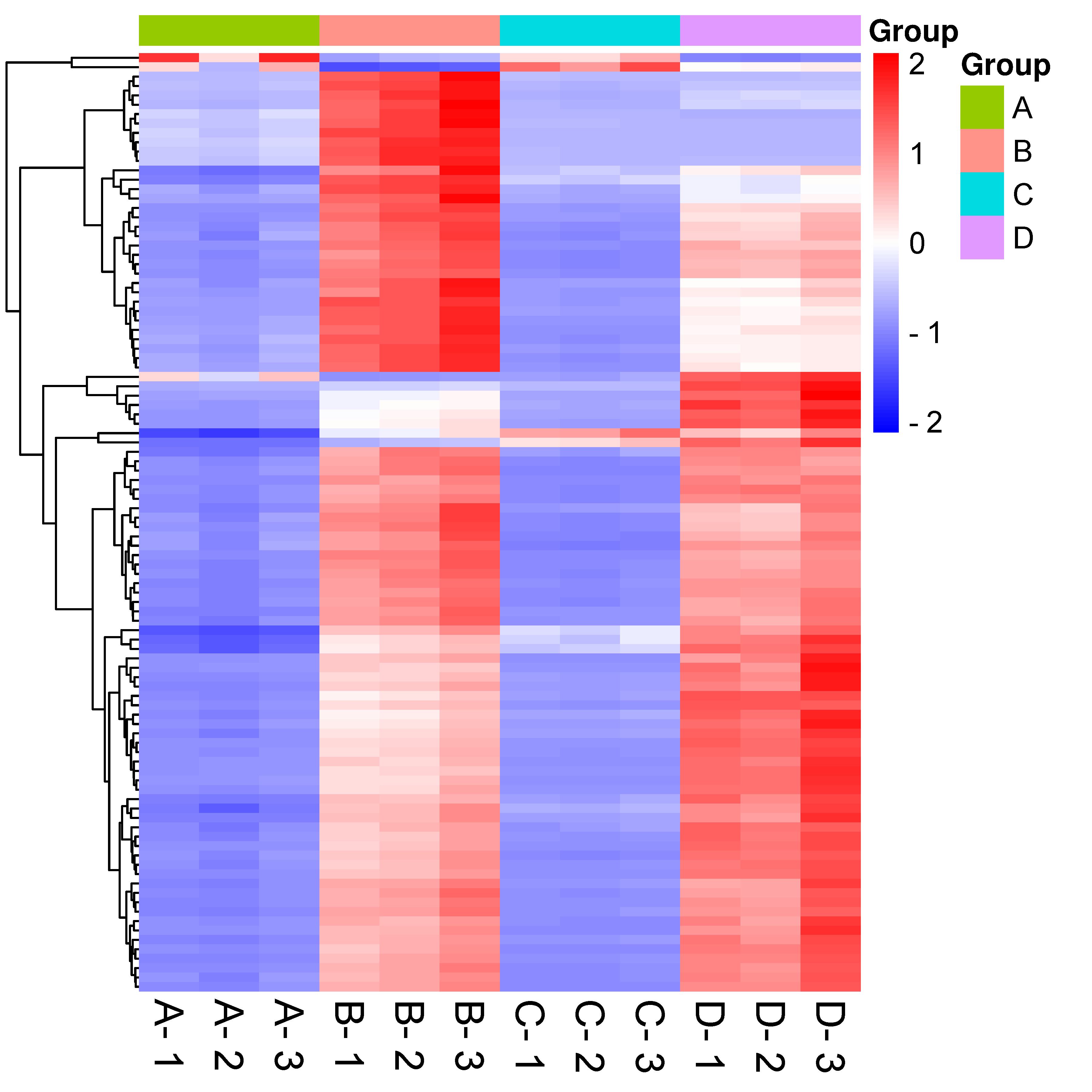

Supplement: Supplementary file 10 — EV figures [file 44321_2025_201_MOESM10_ESM.zip › source data for EV/EV1/EV1 c heatmap/LN229_A172_top100_heatmap_46d1840a9eb1d199.tiff]

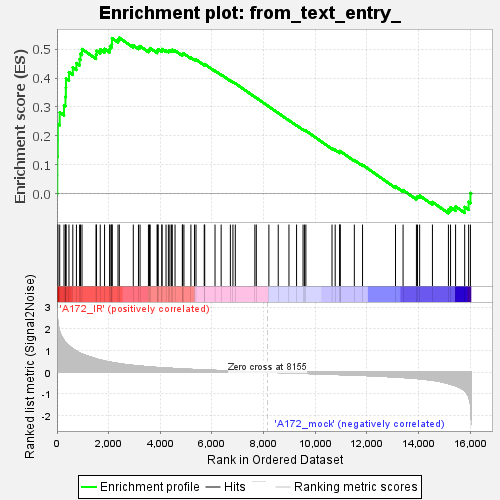

Supplement: Supplementary file 10 — EV figures [file 44321_2025_201_MOESM10_ESM.zip › source data for EV/EV1/EV1 e/A172_enplot_from_NFKB__135.png]

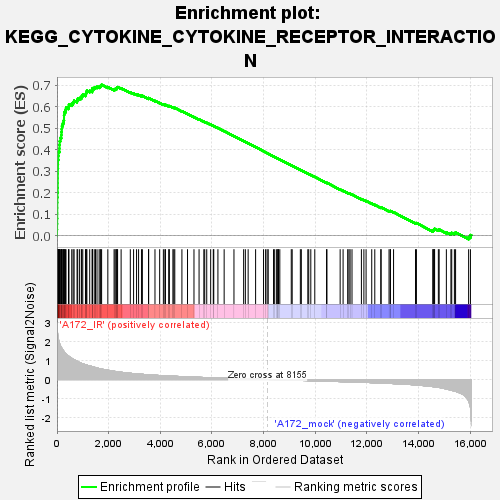

Supplement: Supplementary file 10 — EV figures [file 44321_2025_201_MOESM10_ESM.zip › source data for EV/EV1/EV1 e/A172_enplot_KEGG_CYTOKINE_CYTOKINE_RECEPTOR_INTERACTION_125.png]

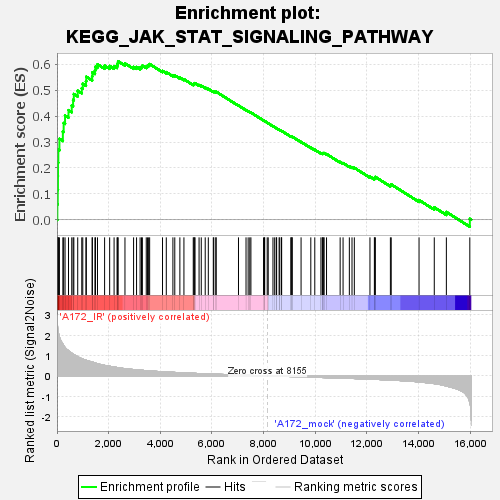

Supplement: Supplementary file 10 — EV figures [file 44321_2025_201_MOESM10_ESM.zip › source data for EV/EV1/EV1 e/A172_enplot_KEGG_JAK_STAT_SIGNALING_PATHWAY_130.png]

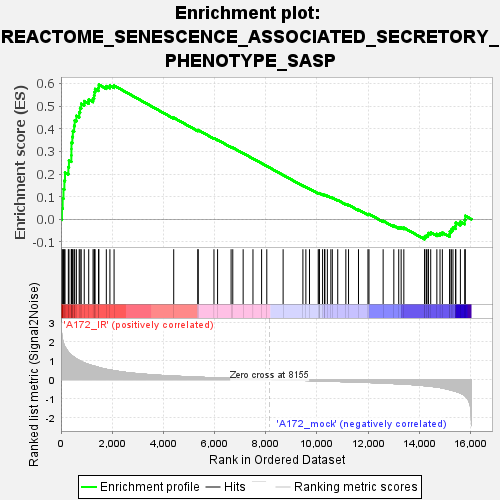

Supplement: Supplementary file 10 — EV figures [file 44321_2025_201_MOESM10_ESM.zip › source data for EV/EV1/EV1 e/A172_SASP_REACTOME_4271.png]

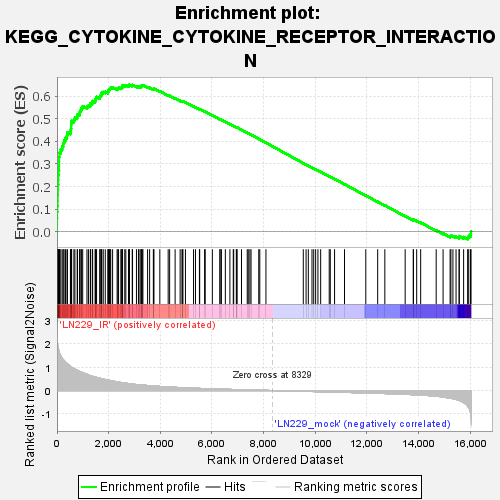

Supplement: Supplementary file 10 — EV figures [file 44321_2025_201_MOESM10_ESM.zip › source data for EV/EV1/EV1 e/LN229_enplot_KEGG_CYTOKINE_CYTOKINE_RECEPTOR_INTERACTION_130.png]

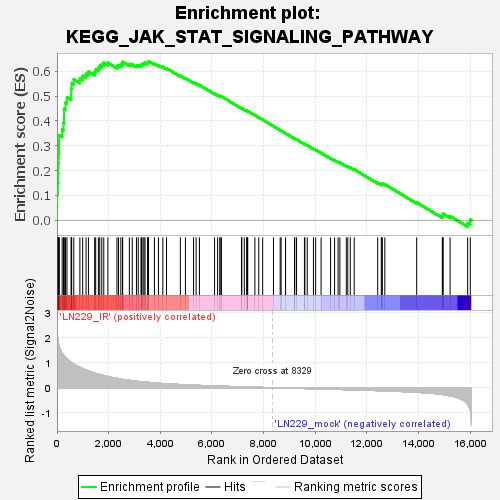

Supplement: Supplementary file 10 — EV figures [file 44321_2025_201_MOESM10_ESM.zip › source data for EV/EV1/EV1 e/LN229_enplot_KEGG_JAK_STAT_SIGNALING_PATHWAY_125.png]

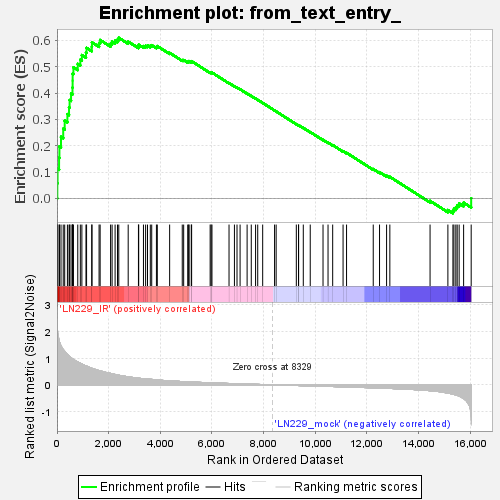

Supplement: Supplementary file 10 — EV figures [file 44321_2025_201_MOESM10_ESM.zip › source data for EV/EV1/EV1 e/LN229_NFkB_enplot_fromNFKB145.png]

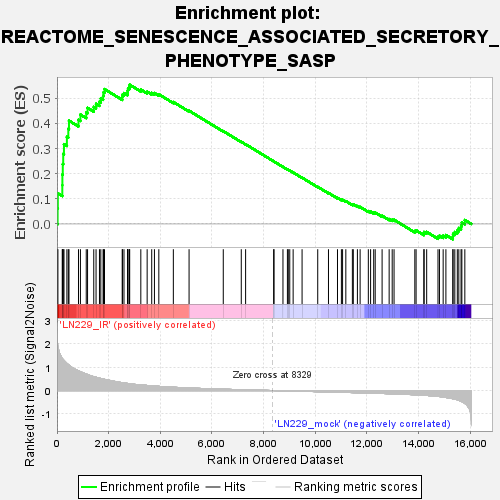

Supplement: Supplementary file 10 — EV figures [file 44321_2025_201_MOESM10_ESM.zip › source data for EV/EV1/EV1 e/LN229_SASP_REACTOME_293.png]

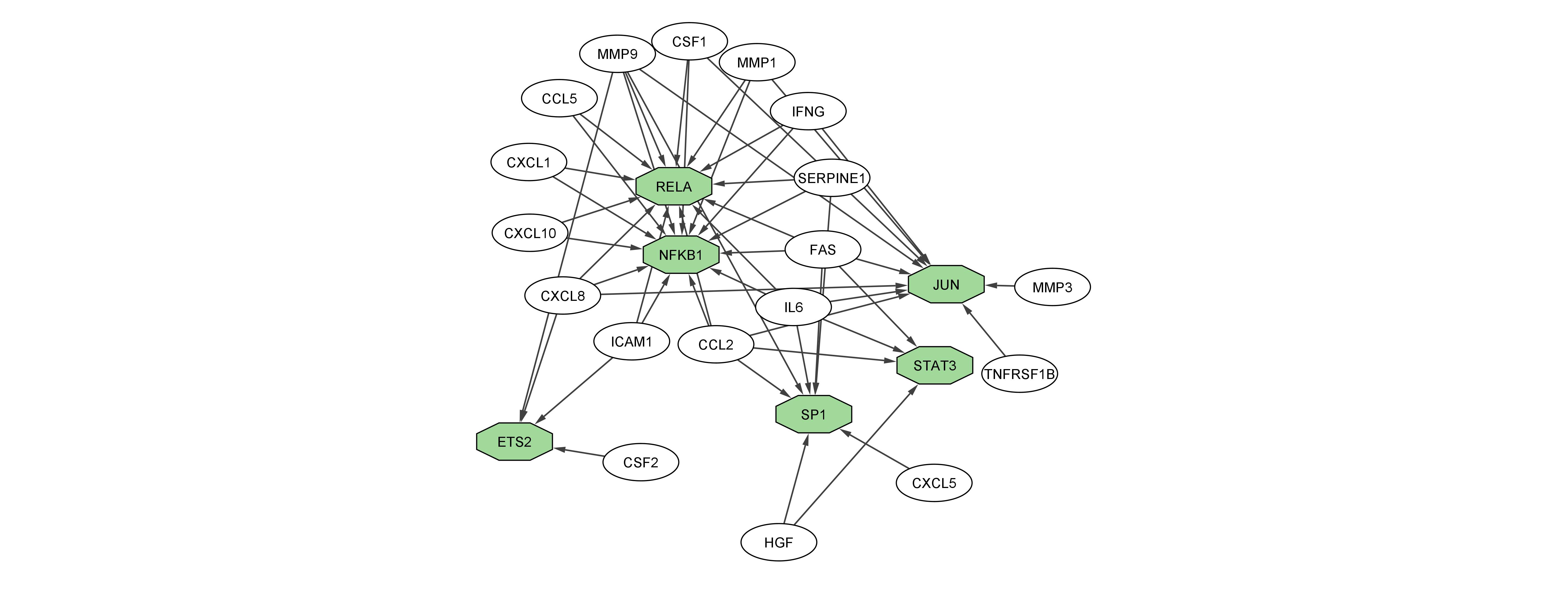

Supplement: Supplementary file 10 — EV figures [file 44321_2025_201_MOESM10_ESM.zip › source data for EV/EV1/EV1 f/Cytoscape network v1.jpeg]
