# Supplementary figures and images for: Targeting cIAP2 in a novel senolytic strategy prevents glioblastoma recurrence after radiotherapy (part 4 of 4)
Source: EMBO Mol Med. 2025 Feb 19;17(4):645–78. doi: 10.1038/s44321-025-00201-x (PMC11982261; doi:10.1038/s44321-025-00201-x)

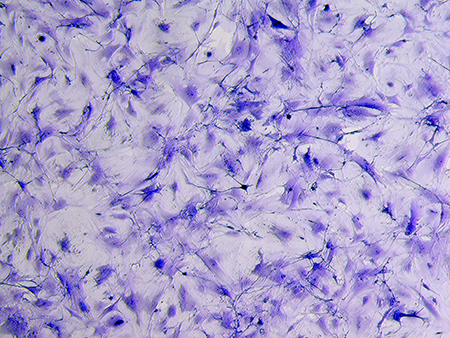

Supplement: Supplementary file 10 — EV figures [file 44321_2025_201_MOESM10_ESM.zip › source data for EV/EV5/EV5c CV/GBM148/IR/IR-Biri-Day0.jpg]

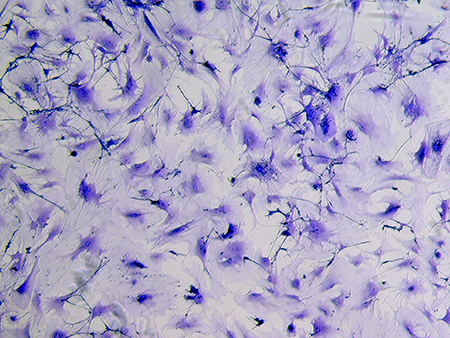

Supplement: Supplementary file 10 — EV figures [file 44321_2025_201_MOESM10_ESM.zip › source data for EV/EV5/EV5c CV/GBM148/IR/IR-Biri-Day3.jpg]

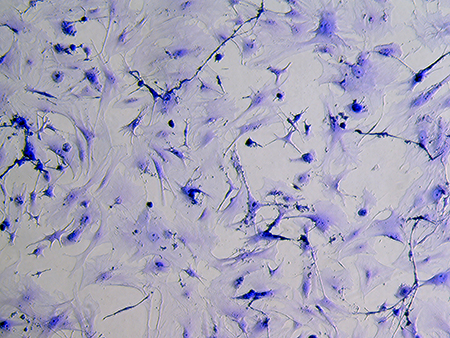

Supplement: Supplementary file 10 — EV figures [file 44321_2025_201_MOESM10_ESM.zip › source data for EV/EV5/EV5c CV/GBM148/IR/IR-Biri-Day6.jpg]

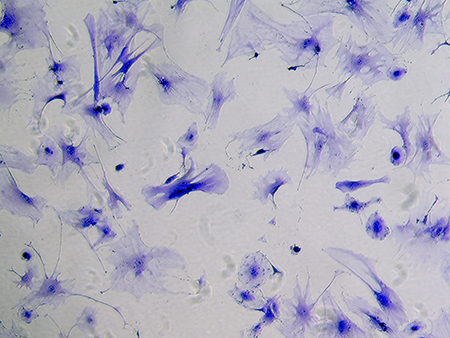

Supplement: Supplementary file 10 — EV figures [file 44321_2025_201_MOESM10_ESM.zip › source data for EV/EV5/EV5c CV/GBM148/IR/IR-Biri-Day9.jpg]

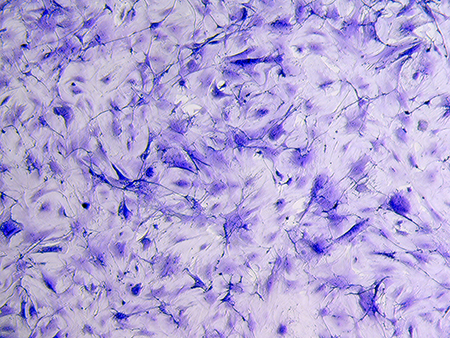

Supplement: Supplementary file 10 — EV figures [file 44321_2025_201_MOESM10_ESM.zip › source data for EV/EV5/EV5c CV/GBM148/IR/IR-DMSO-Day0.jpg]

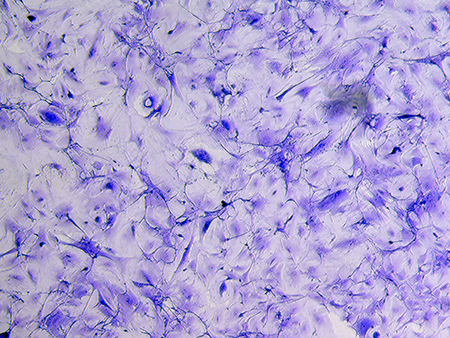

Supplement: Supplementary file 10 — EV figures [file 44321_2025_201_MOESM10_ESM.zip › source data for EV/EV5/EV5c CV/GBM148/IR/IR-DMSO-Day3.jpg]

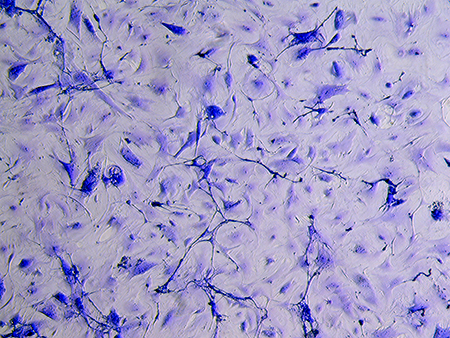

Supplement: Supplementary file 10 — EV figures [file 44321_2025_201_MOESM10_ESM.zip › source data for EV/EV5/EV5c CV/GBM148/IR/IR-DMSO-Day6.jpg]

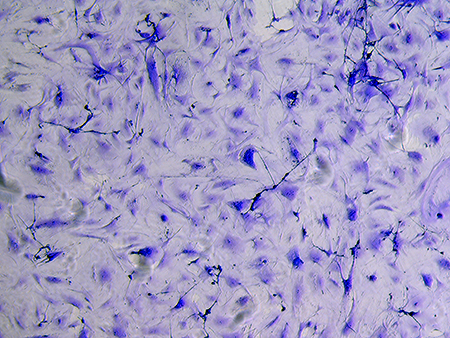

Supplement: Supplementary file 10 — EV figures [file 44321_2025_201_MOESM10_ESM.zip › source data for EV/EV5/EV5c CV/GBM148/IR/IR-DMSO-Day9.jpg]

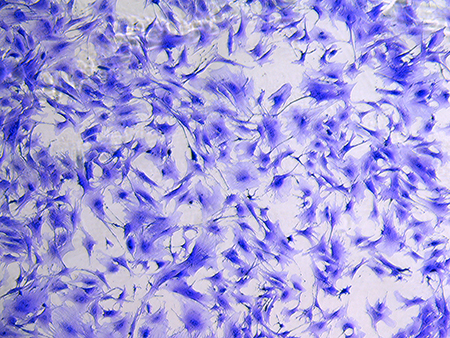

Supplement: Supplementary file 10 — EV figures [file 44321_2025_201_MOESM10_ESM.zip › source data for EV/EV5/EV5c CV/GBM148/mock/B-Day0.jpg]

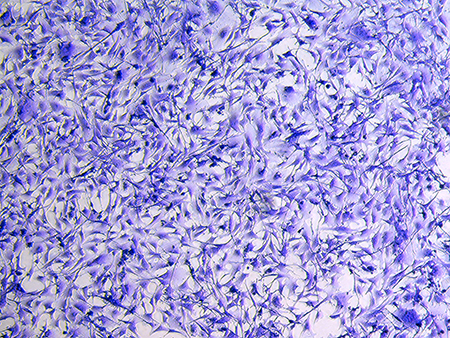

Supplement: Supplementary file 10 — EV figures [file 44321_2025_201_MOESM10_ESM.zip › source data for EV/EV5/EV5c CV/GBM148/mock/B-Day3.jpg]

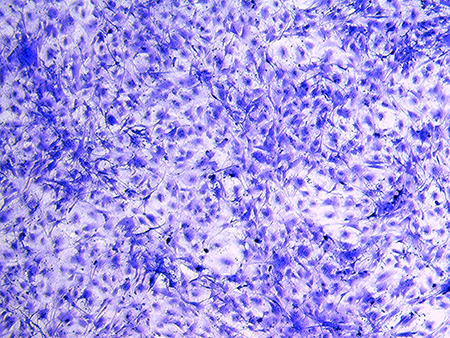

Supplement: Supplementary file 10 — EV figures [file 44321_2025_201_MOESM10_ESM.zip › source data for EV/EV5/EV5c CV/GBM148/mock/B-Day6.jpg]

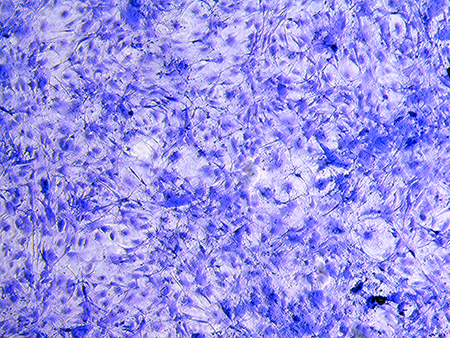

Supplement: Supplementary file 10 — EV figures [file 44321_2025_201_MOESM10_ESM.zip › source data for EV/EV5/EV5c CV/GBM148/mock/B-Day9.jpg]

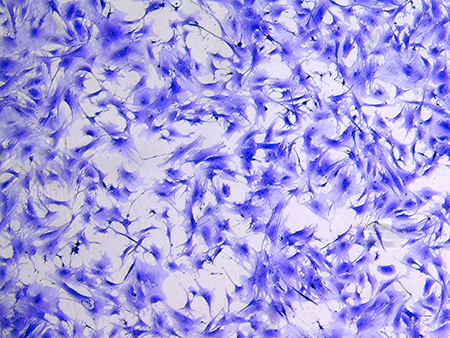

Supplement: Supplementary file 10 — EV figures [file 44321_2025_201_MOESM10_ESM.zip › source data for EV/EV5/EV5c CV/GBM148/mock/D-Day0.jpg]

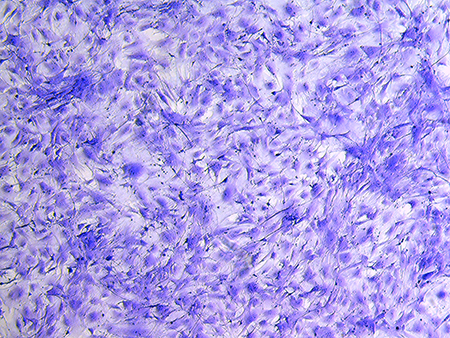

Supplement: Supplementary file 10 — EV figures [file 44321_2025_201_MOESM10_ESM.zip › source data for EV/EV5/EV5c CV/GBM148/mock/D-Day3.jpg]

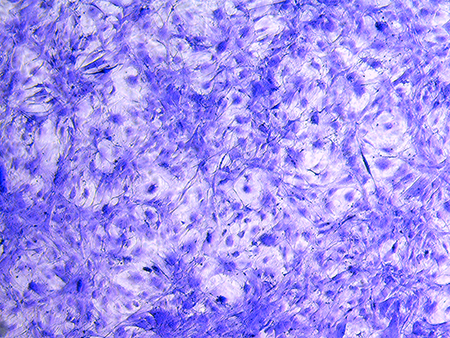

Supplement: Supplementary file 10 — EV figures [file 44321_2025_201_MOESM10_ESM.zip › source data for EV/EV5/EV5c CV/GBM148/mock/D-Day6.jpg]

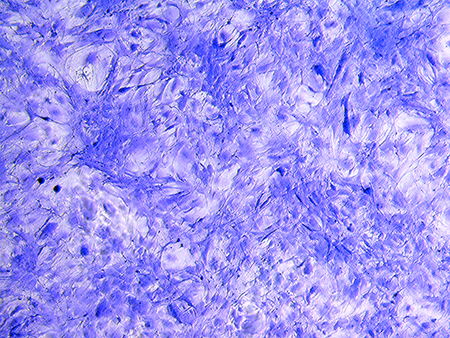

Supplement: Supplementary file 10 — EV figures [file 44321_2025_201_MOESM10_ESM.zip › source data for EV/EV5/EV5c CV/GBM148/mock/D-Day9.jpg]

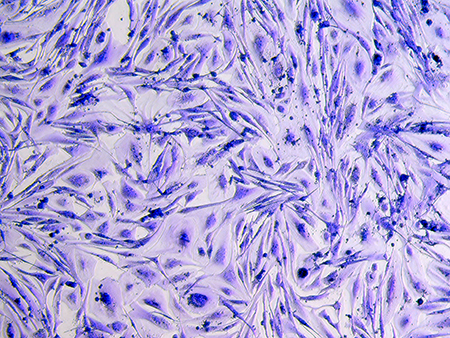

Supplement: Supplementary file 10 — EV figures [file 44321_2025_201_MOESM10_ESM.zip › source data for EV/EV5/EV5c CV/GBM245/IR/Biri-Day0.jpg]

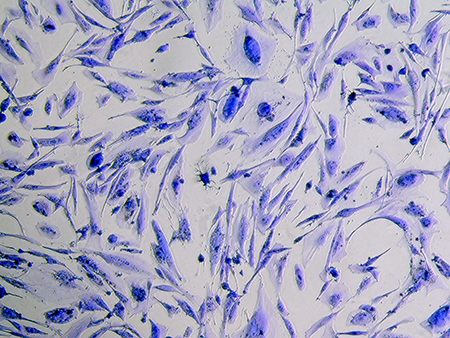

Supplement: Supplementary file 10 — EV figures [file 44321_2025_201_MOESM10_ESM.zip › source data for EV/EV5/EV5c CV/GBM245/IR/Biri-Day3.jpg]
