# Supplementary figures and images for: Targeting cIAP2 in a novel senolytic strategy prevents glioblastoma recurrence after radiotherapy (part 3 of 4)
Source: EMBO Mol Med. 2025 Feb 19;17(4):645–78. doi: 10.1038/s44321-025-00201-x (PMC11982261; doi:10.1038/s44321-025-00201-x)

EV2

a

LN229

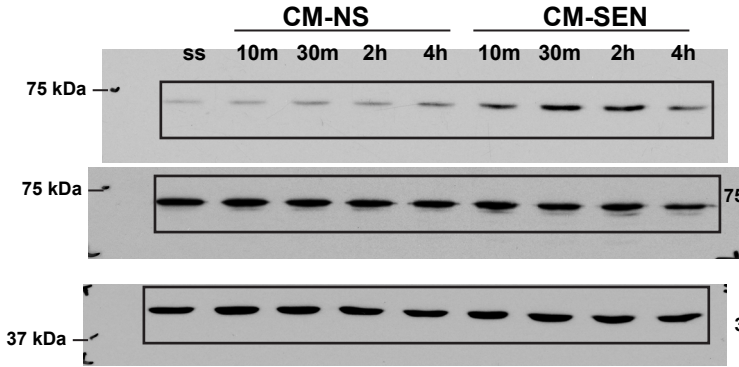

A172

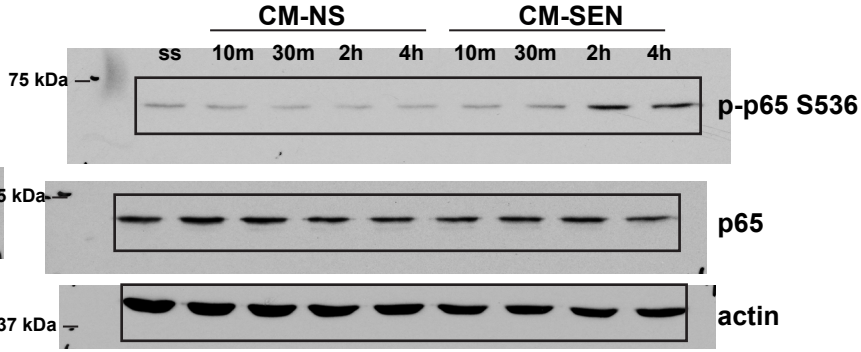

U118

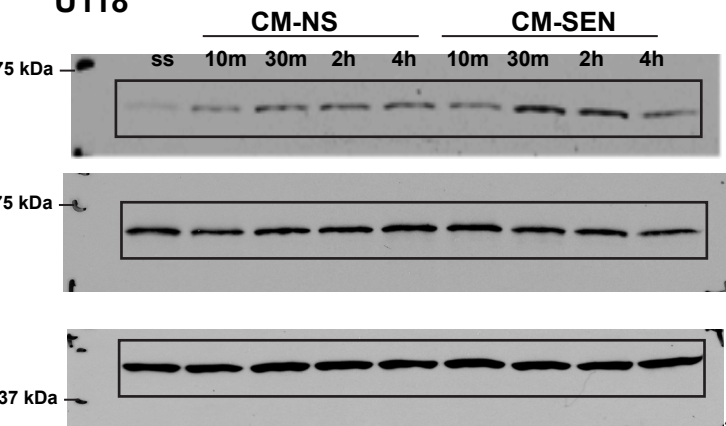

U87

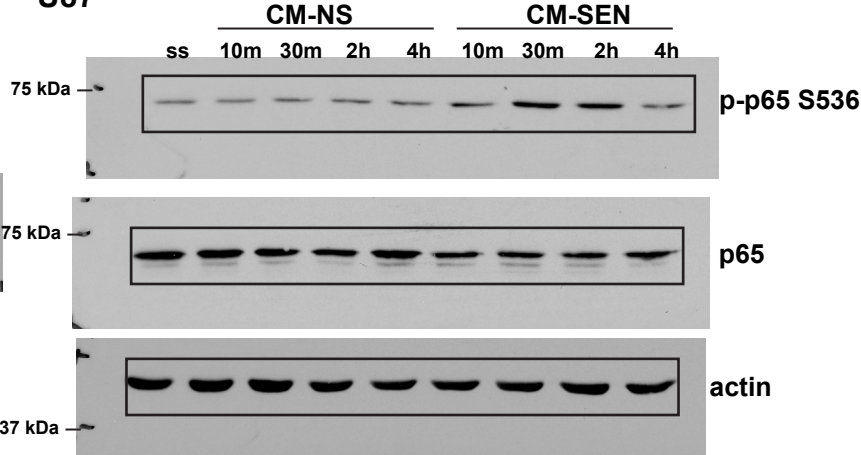

Supplement: Supplementary file 10 — EV figures [file 44321_2025_201_MOESM10_ESM.zip › source data for EV/EV2/EV2a.pdf]

Fig EV3

**C**

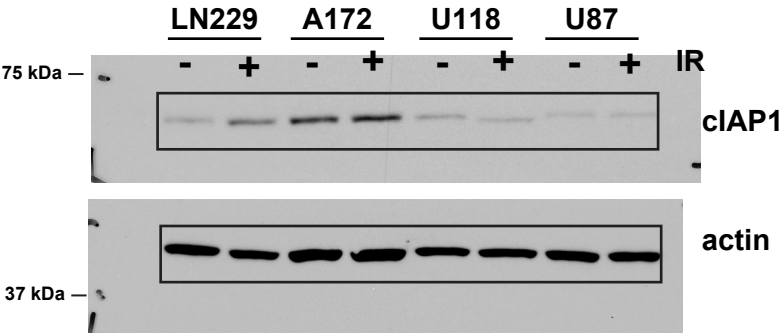

**e**

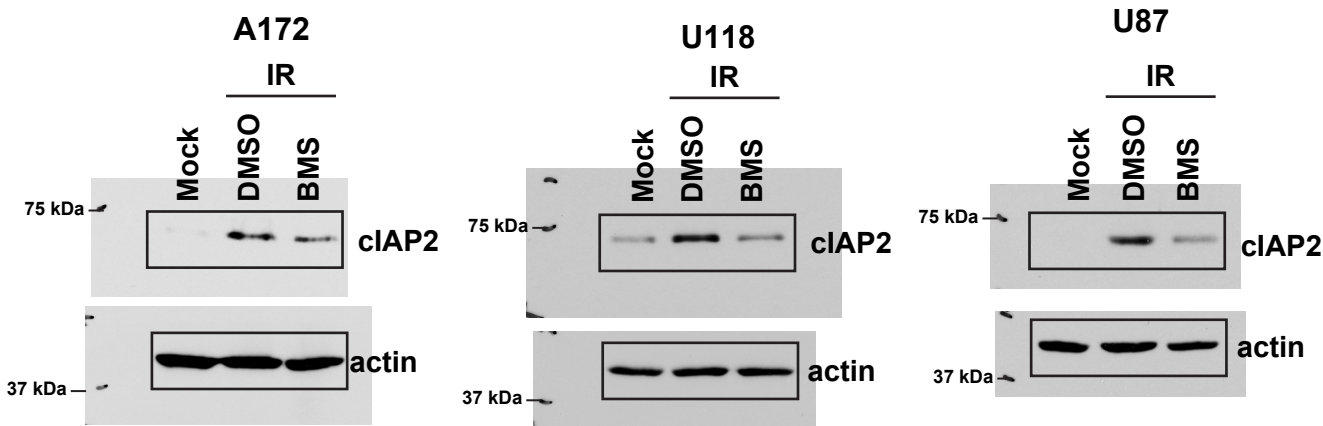

**f**

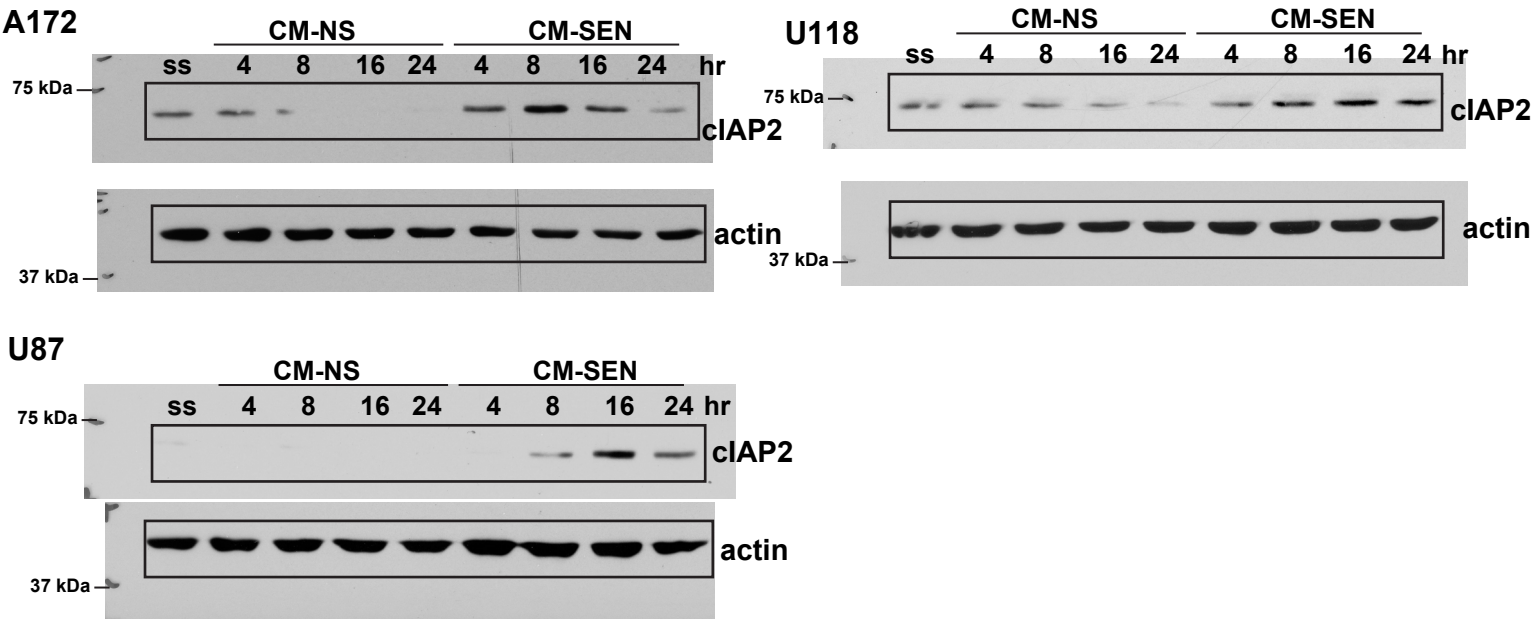

**h**

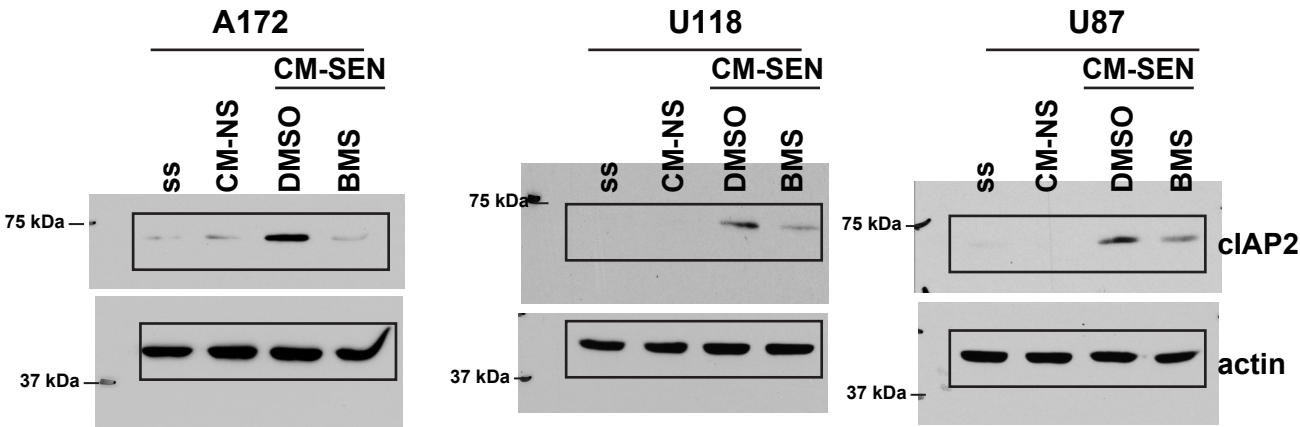

Supplement: Supplementary file 10 — EV figures [file 44321_2025_201_MOESM10_ESM.zip › source data for EV/EV3/EV3 WB.pdf]

# EV4

a

229

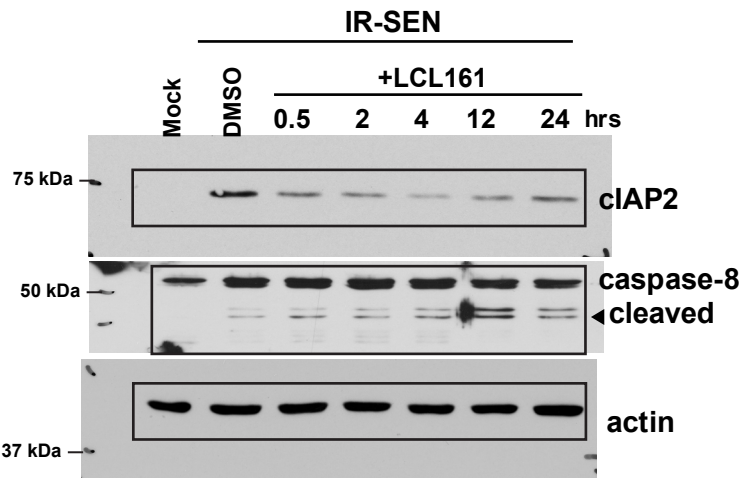

A172

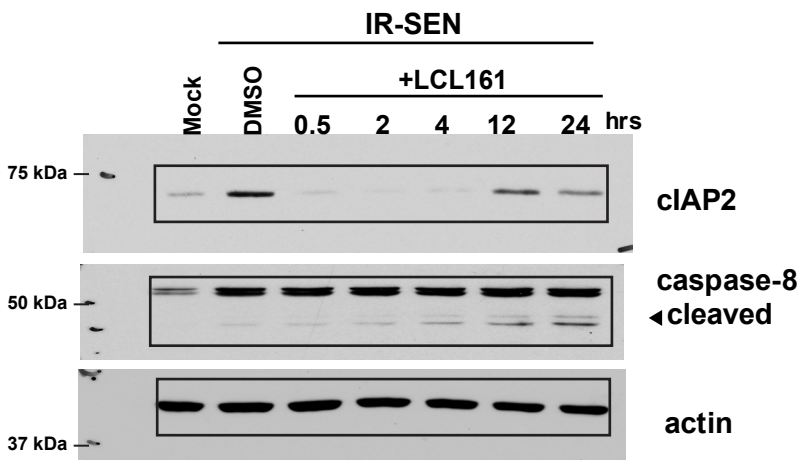

U118

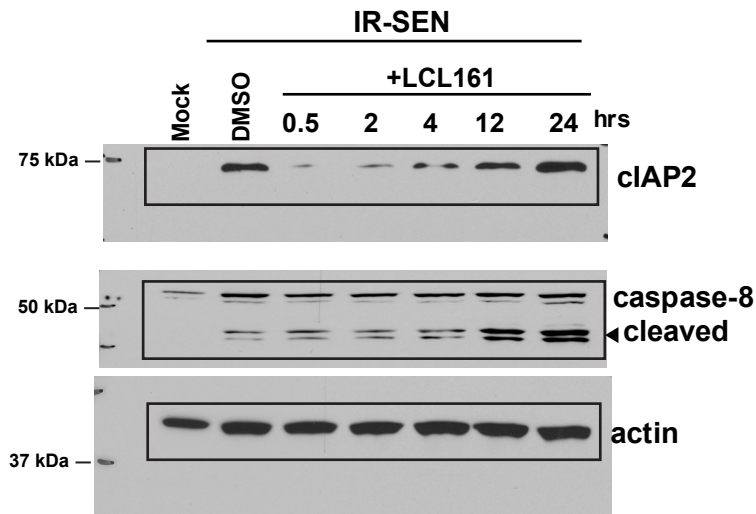

U87

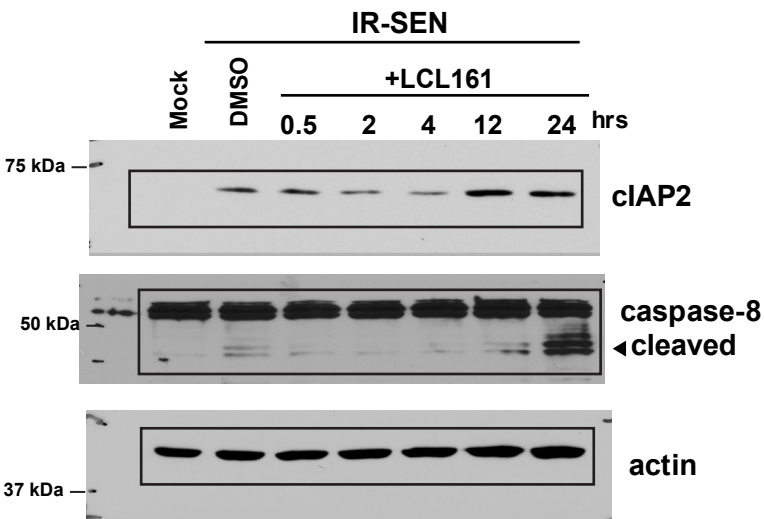

Supplement: Supplementary file 10 — EV figures [file 44321_2025_201_MOESM10_ESM.zip › source data for EV/EV4/EV4a WB.pdf]

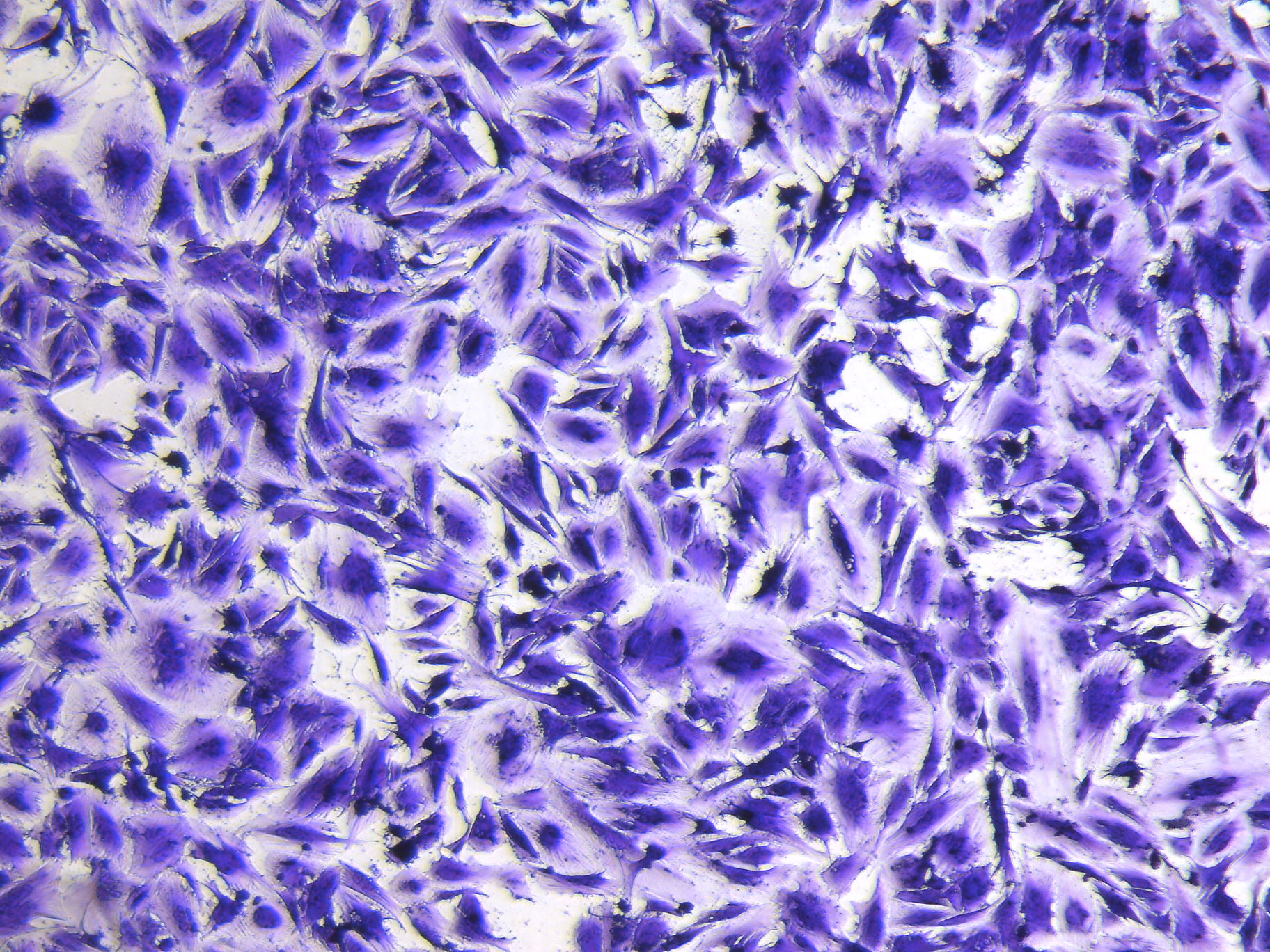

Supplement: Supplementary file 10 — EV figures [file 44321_2025_201_MOESM10_ESM.zip › source data for EV/EV4/EV4b CV/229/IR/DMSO D0.JPG]

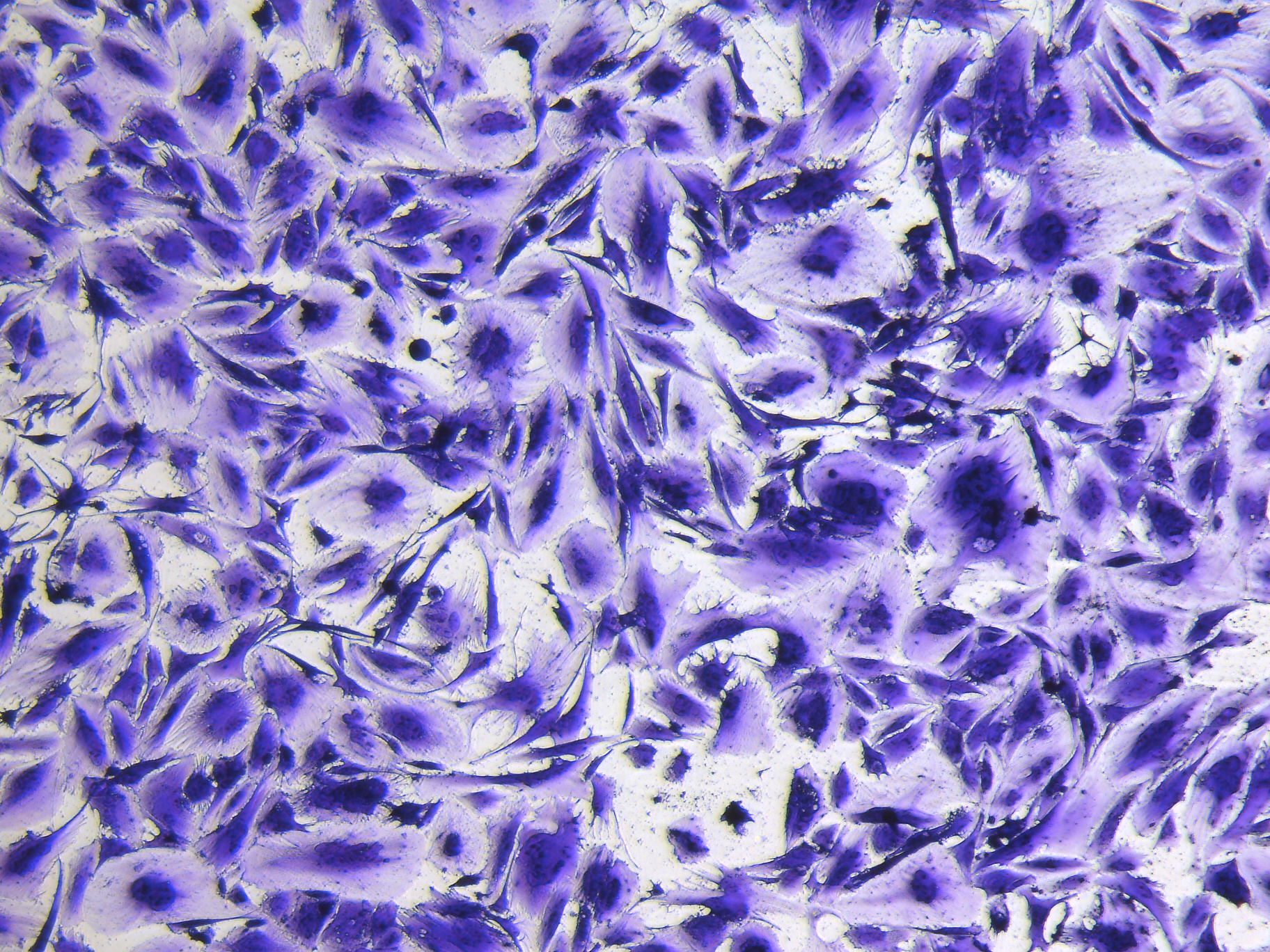

Supplement: Supplementary file 10 — EV figures [file 44321_2025_201_MOESM10_ESM.zip › source data for EV/EV4/EV4b CV/229/IR/DMSO D3.JPG]

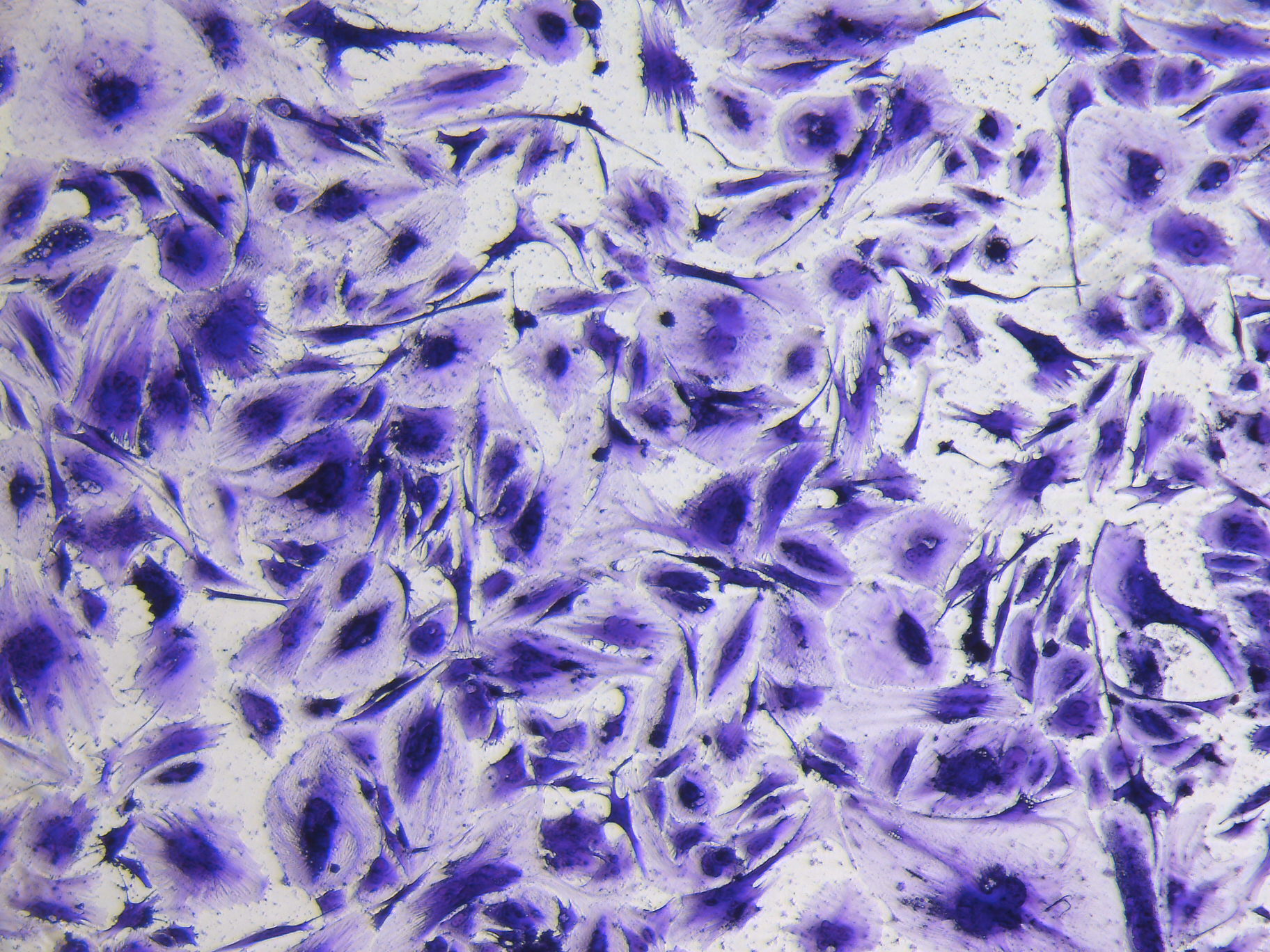

Supplement: Supplementary file 10 — EV figures [file 44321_2025_201_MOESM10_ESM.zip › source data for EV/EV4/EV4b CV/229/IR/DMSO D6.JPG]

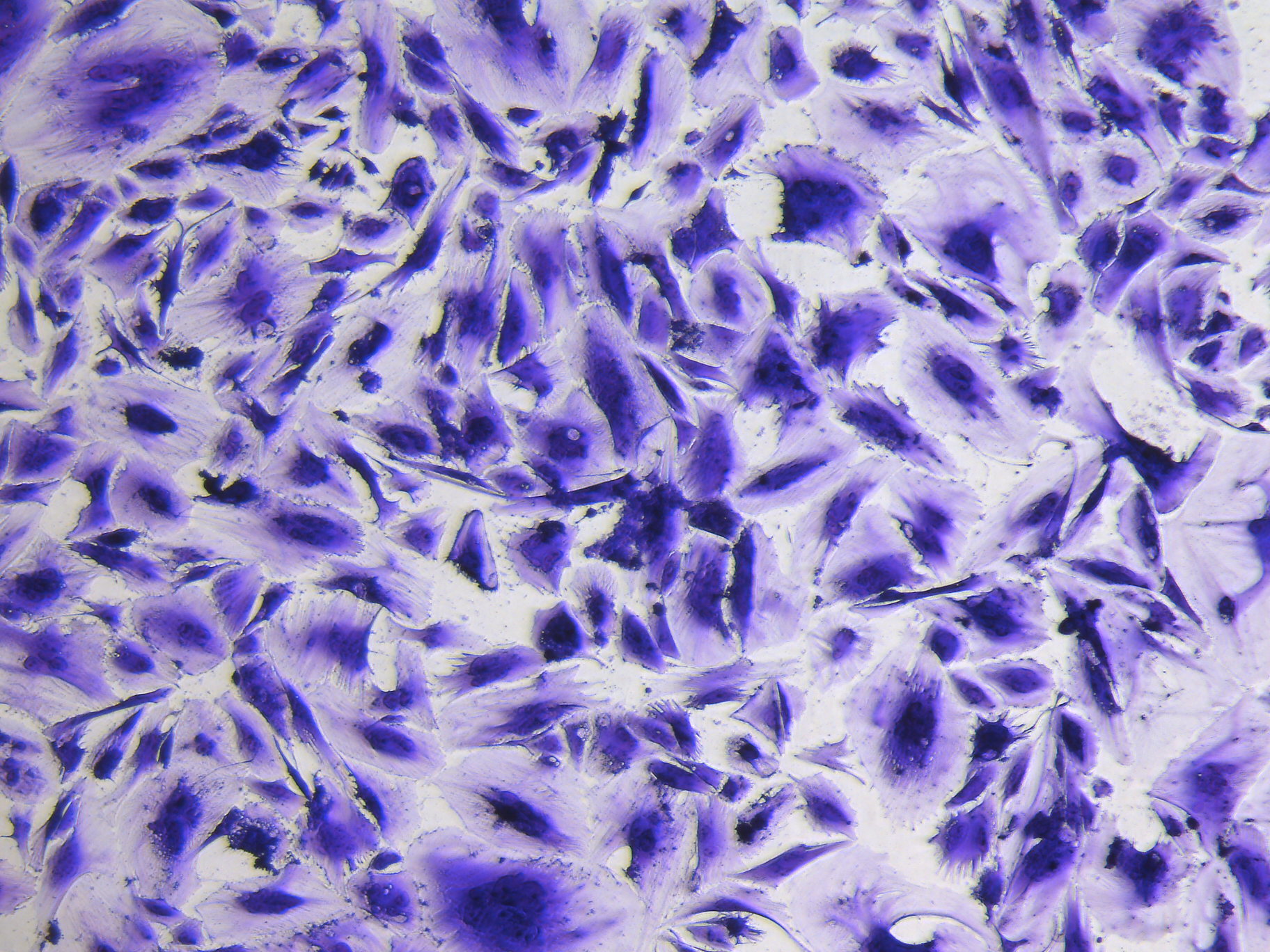

Supplement: Supplementary file 10 — EV figures [file 44321_2025_201_MOESM10_ESM.zip › source data for EV/EV4/EV4b CV/229/IR/DMSO D9.JPG]

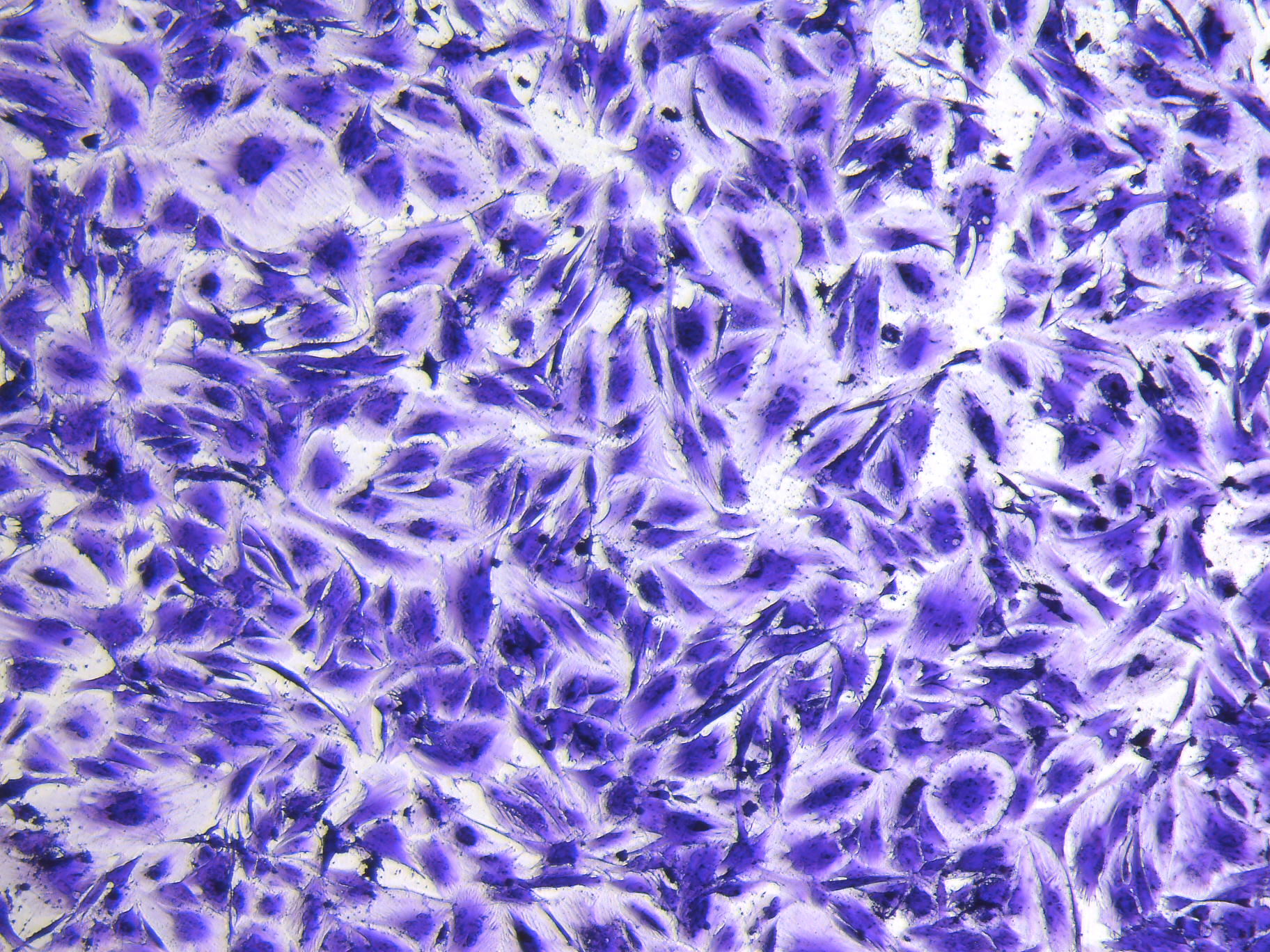

Supplement: Supplementary file 10 — EV figures [file 44321_2025_201_MOESM10_ESM.zip › source data for EV/EV4/EV4b CV/229/IR/LCL D0.JPG]

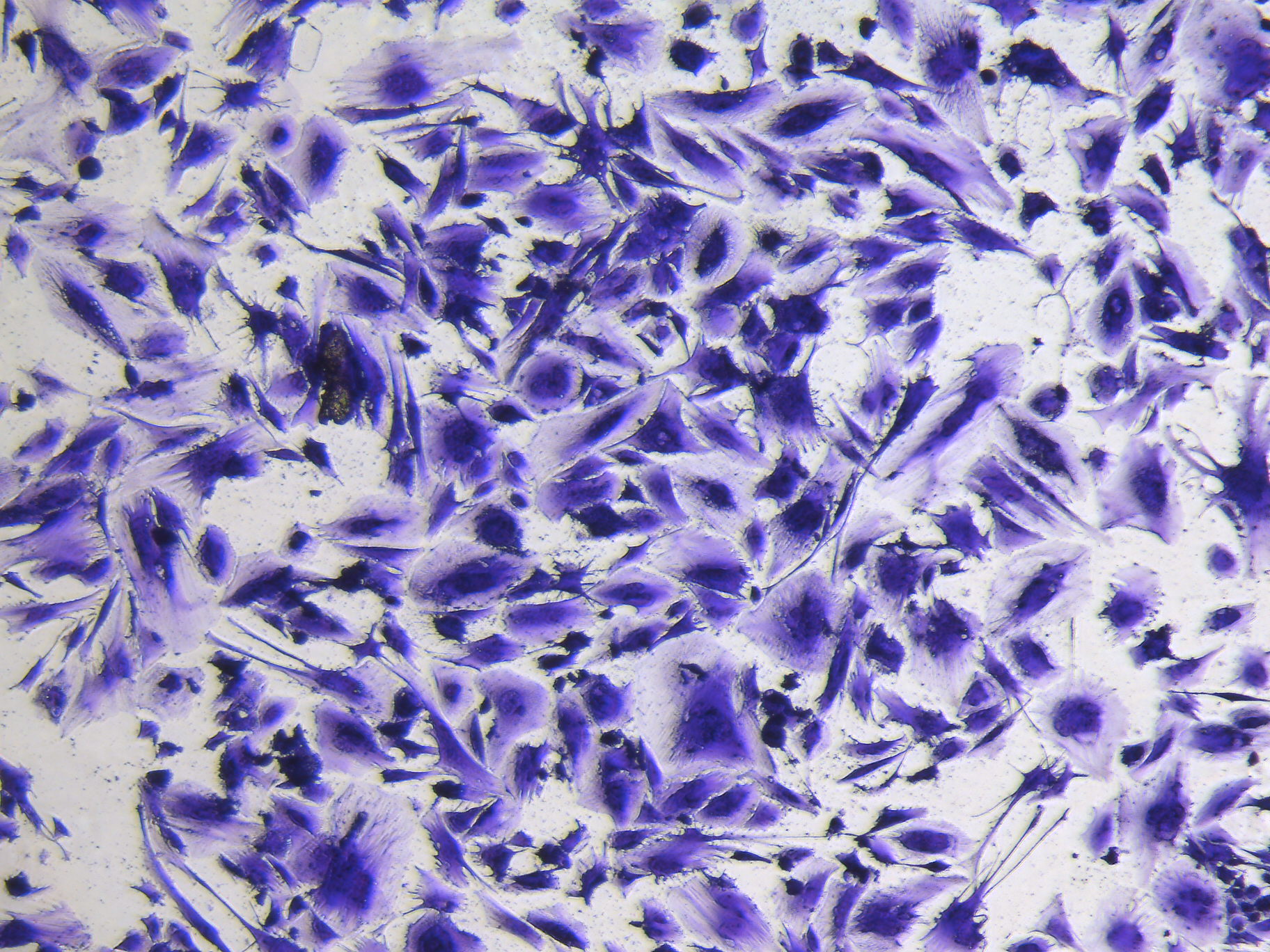

Supplement: Supplementary file 10 — EV figures [file 44321_2025_201_MOESM10_ESM.zip › source data for EV/EV4/EV4b CV/229/IR/LCL D3.JPG]

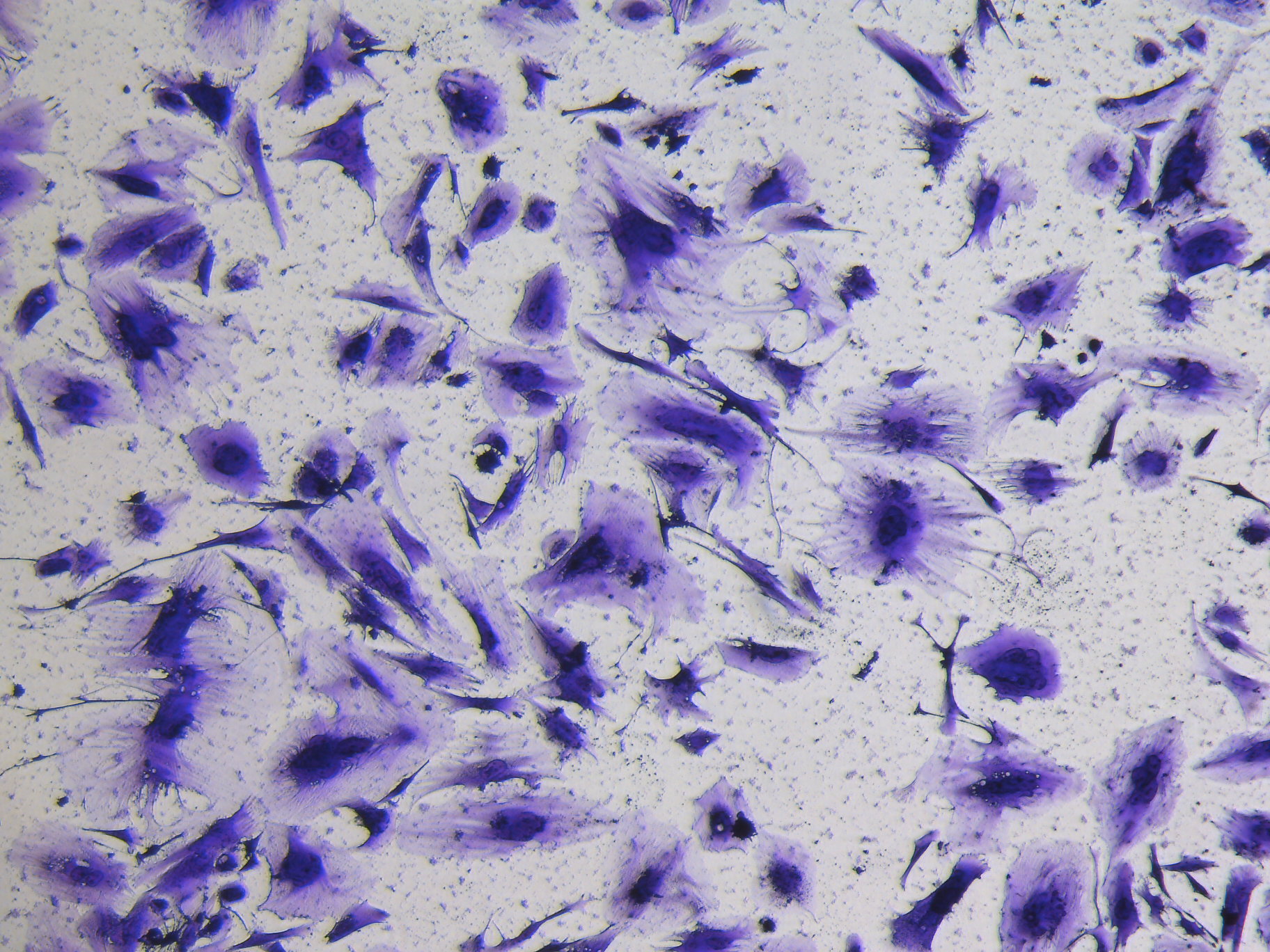

Supplement: Supplementary file 10 — EV figures [file 44321_2025_201_MOESM10_ESM.zip › source data for EV/EV4/EV4b CV/229/IR/LCL D6.JPG]

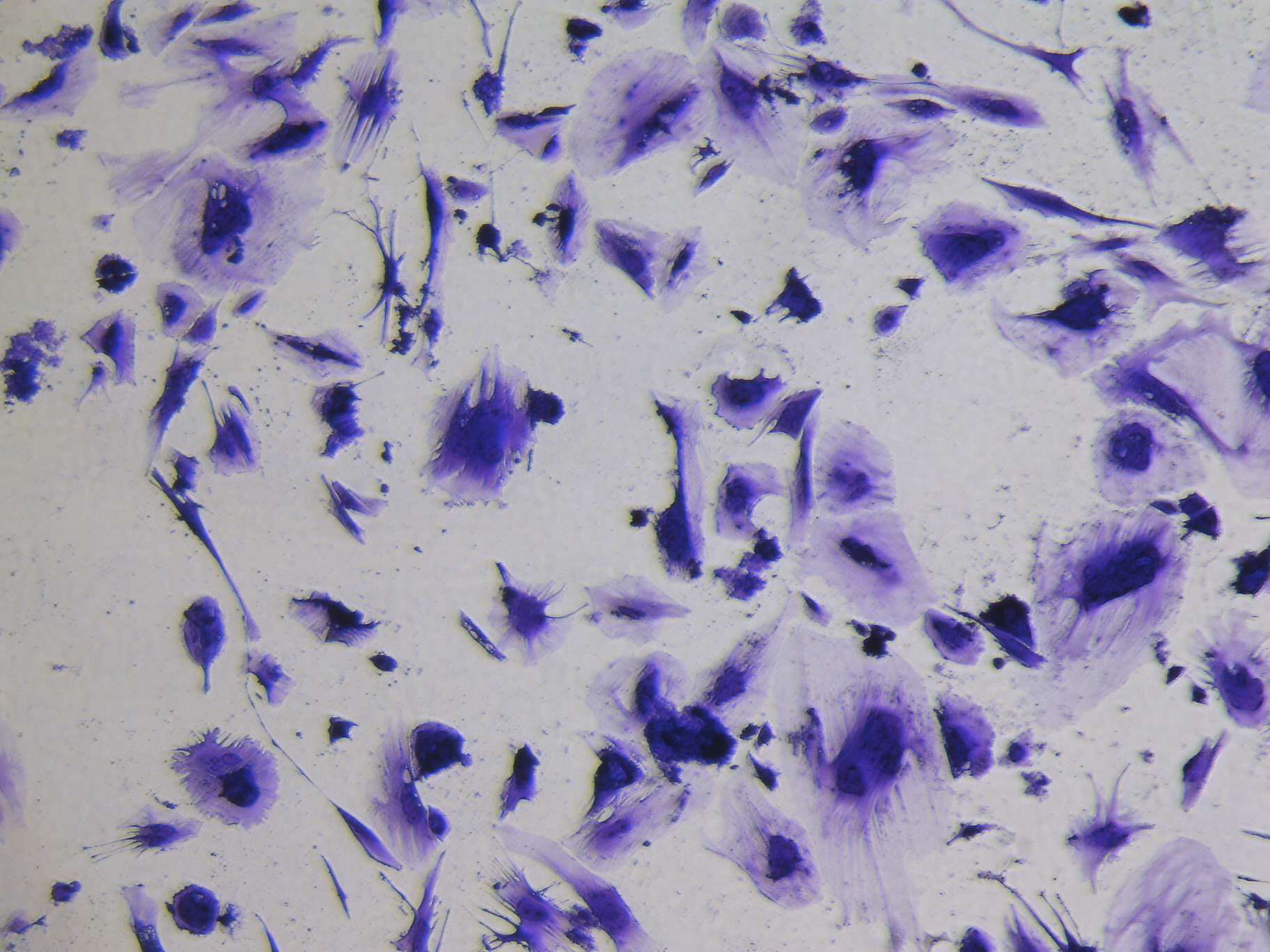

Supplement: Supplementary file 10 — EV figures [file 44321_2025_201_MOESM10_ESM.zip › source data for EV/EV4/EV4b CV/229/IR/LCL D9.JPG]

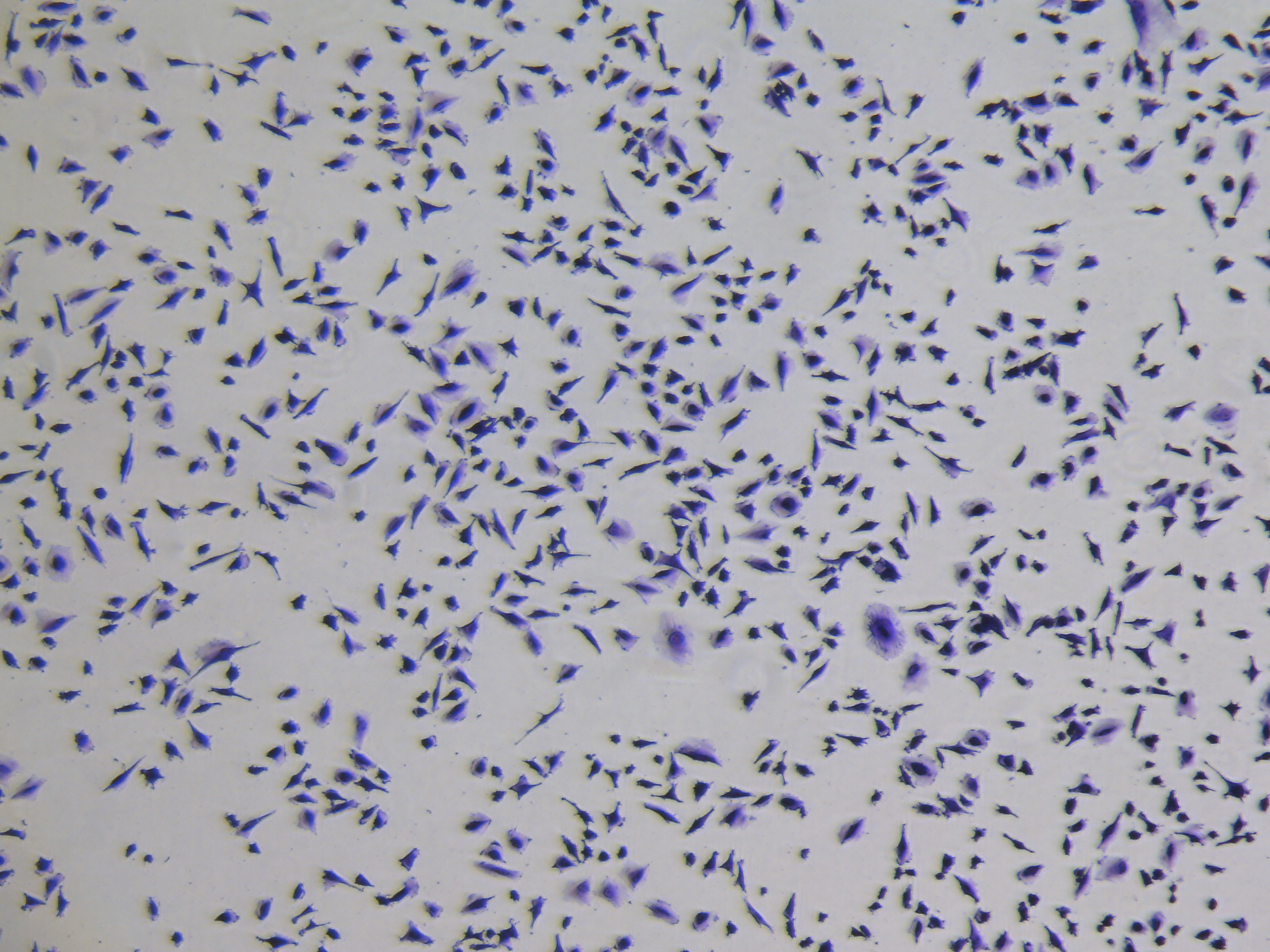

Supplement: Supplementary file 10 — EV figures [file 44321_2025_201_MOESM10_ESM.zip › source data for EV/EV4/EV4b CV/229/mock/DMSO D0.JPG]

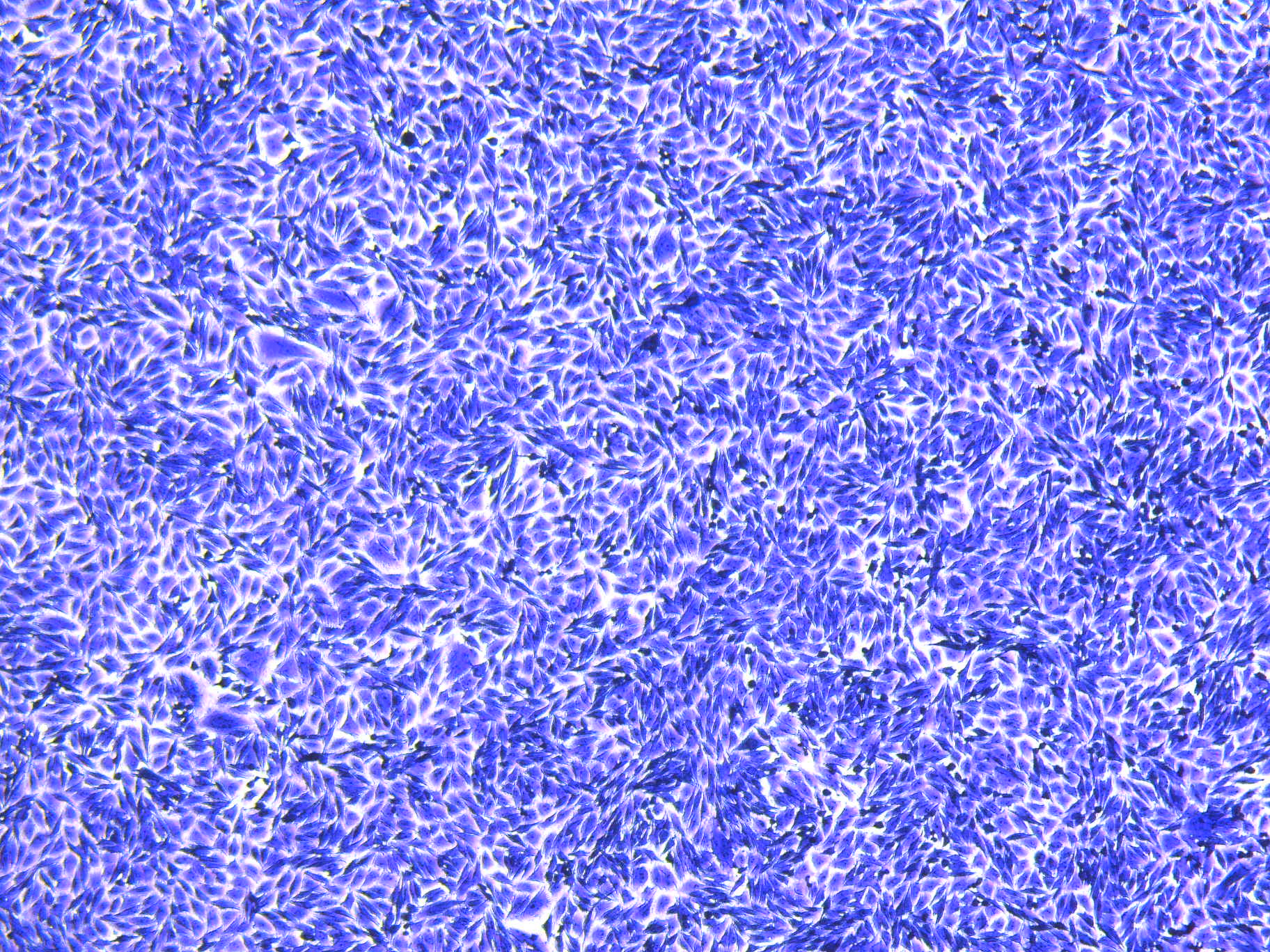

Supplement: Supplementary file 10 — EV figures [file 44321_2025_201_MOESM10_ESM.zip › source data for EV/EV4/EV4b CV/229/mock/DMSO D3.JPG]

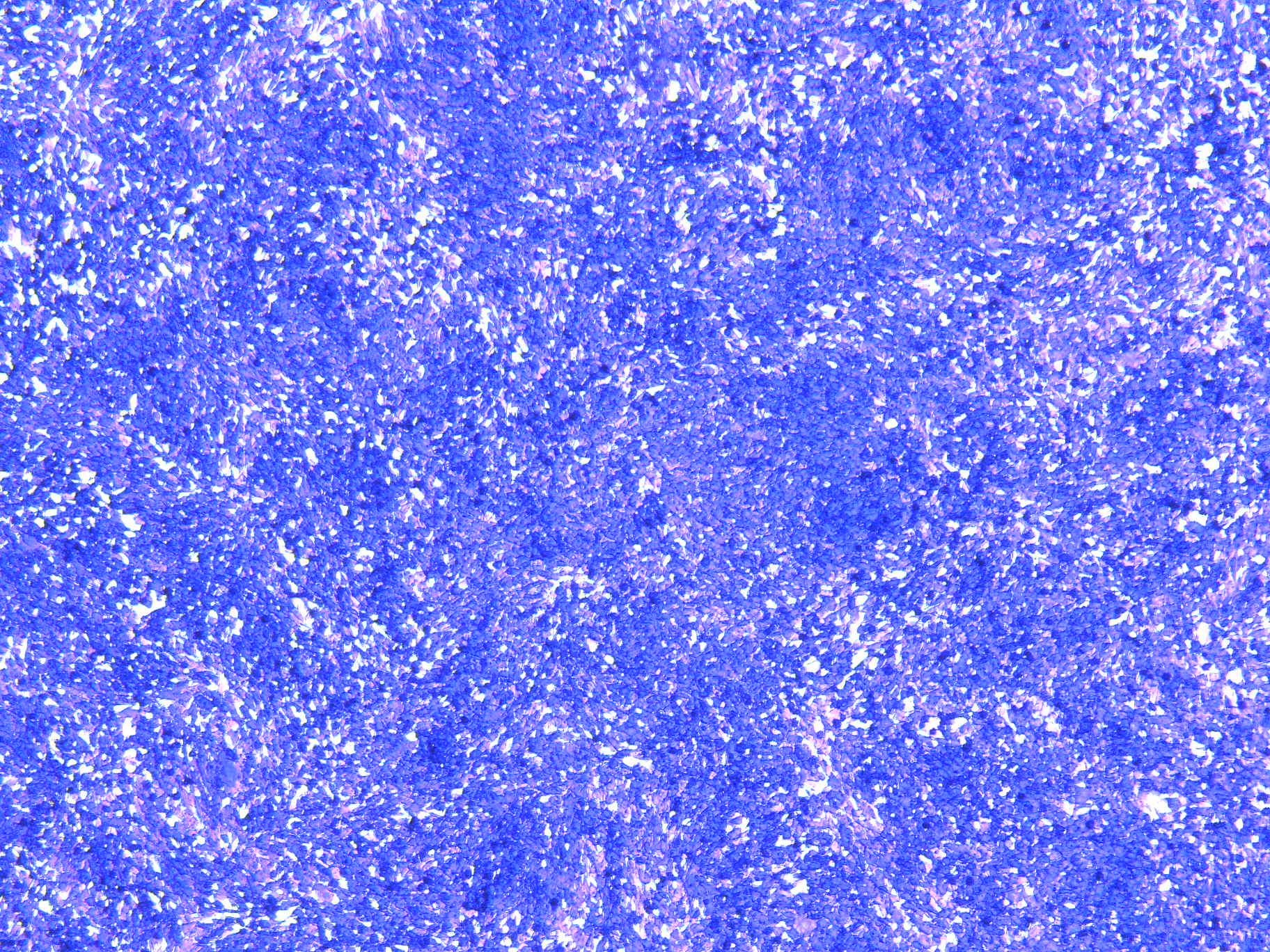

Supplement: Supplementary file 10 — EV figures [file 44321_2025_201_MOESM10_ESM.zip › source data for EV/EV4/EV4b CV/229/mock/DMSO D6.JPG]

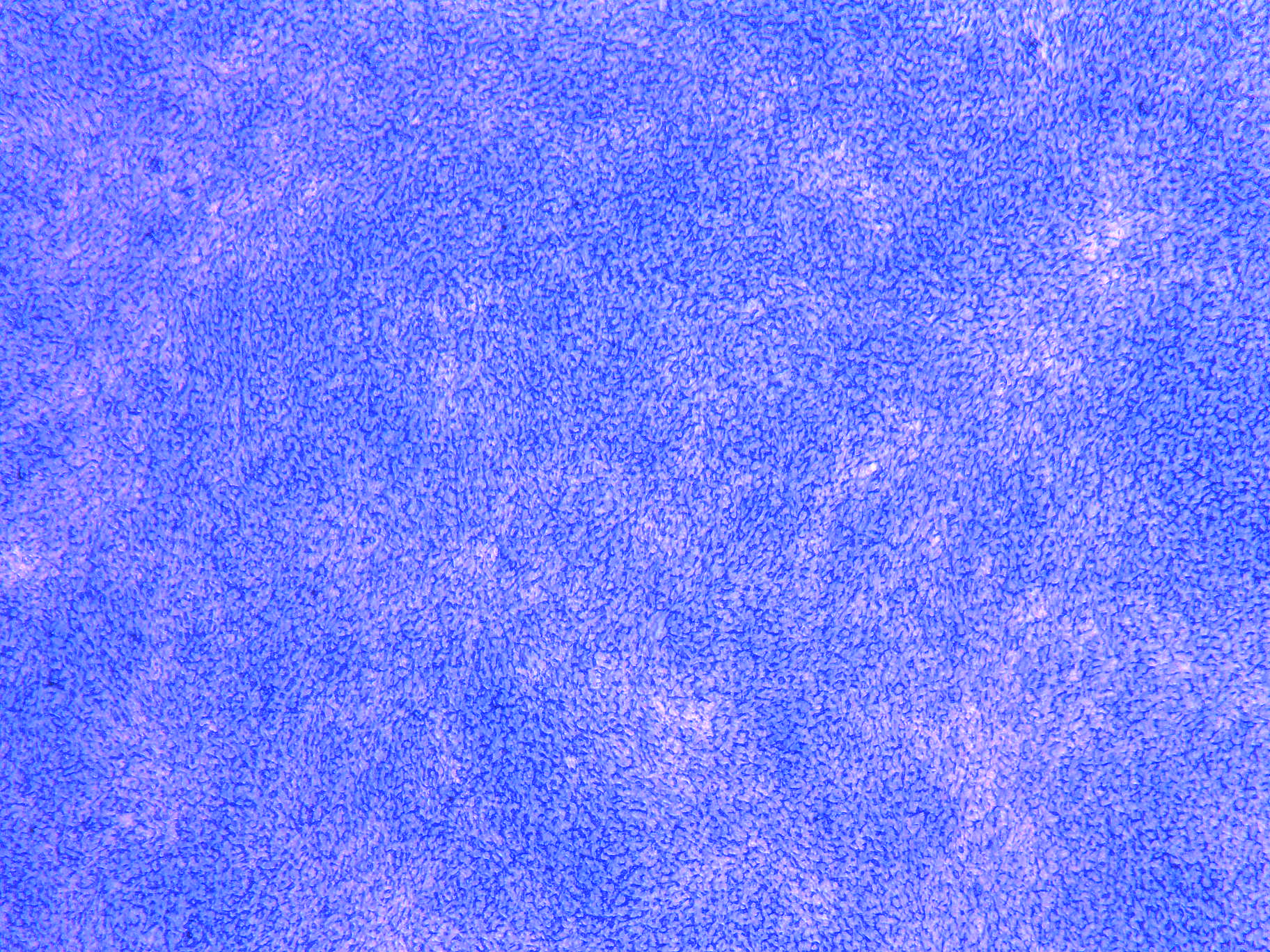

Supplement: Supplementary file 10 — EV figures [file 44321_2025_201_MOESM10_ESM.zip › source data for EV/EV4/EV4b CV/229/mock/DMSO D9.JPG]

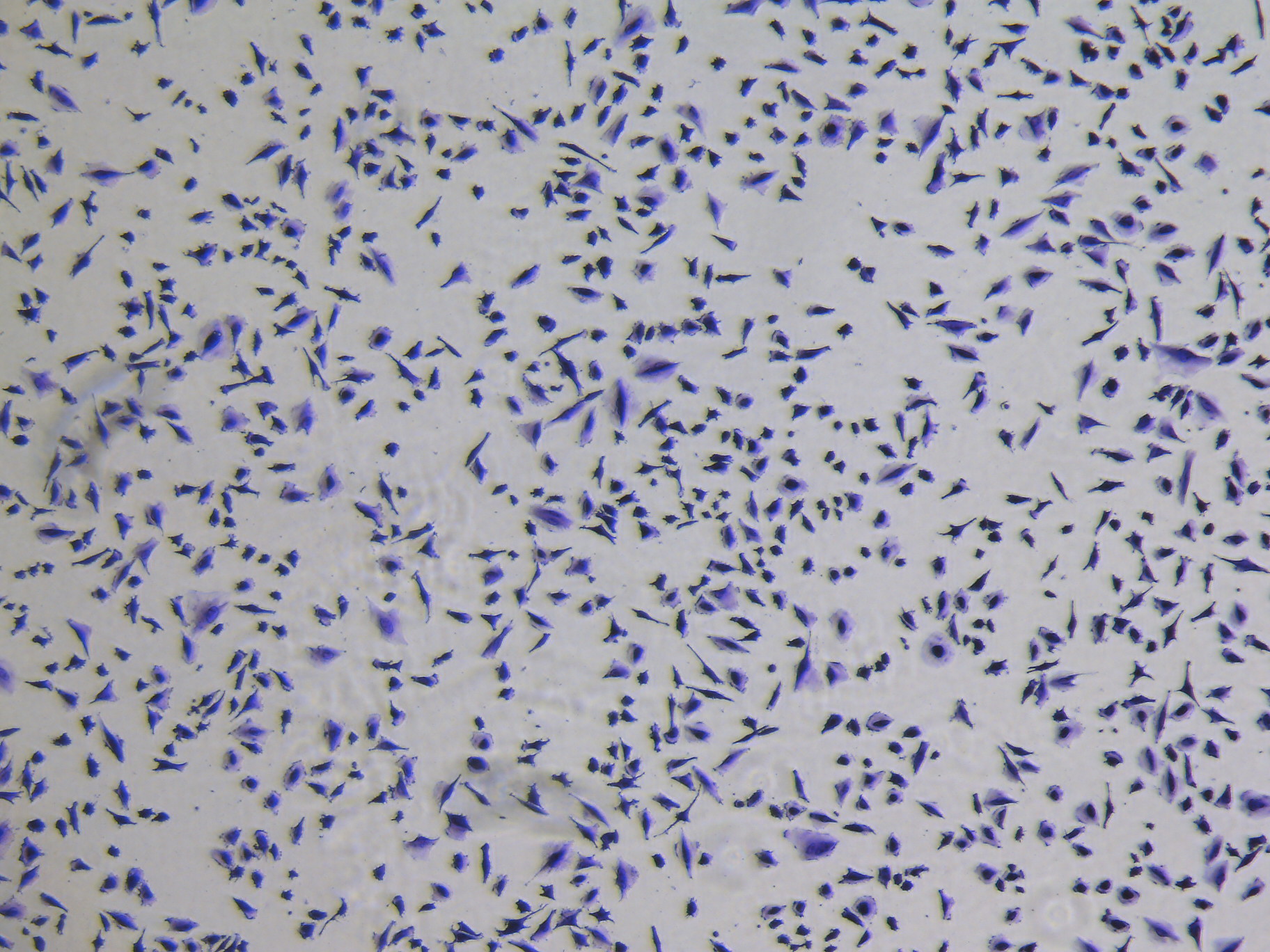

Supplement: Supplementary file 10 — EV figures [file 44321_2025_201_MOESM10_ESM.zip › source data for EV/EV4/EV4b CV/229/mock/LCL D0.JPG]

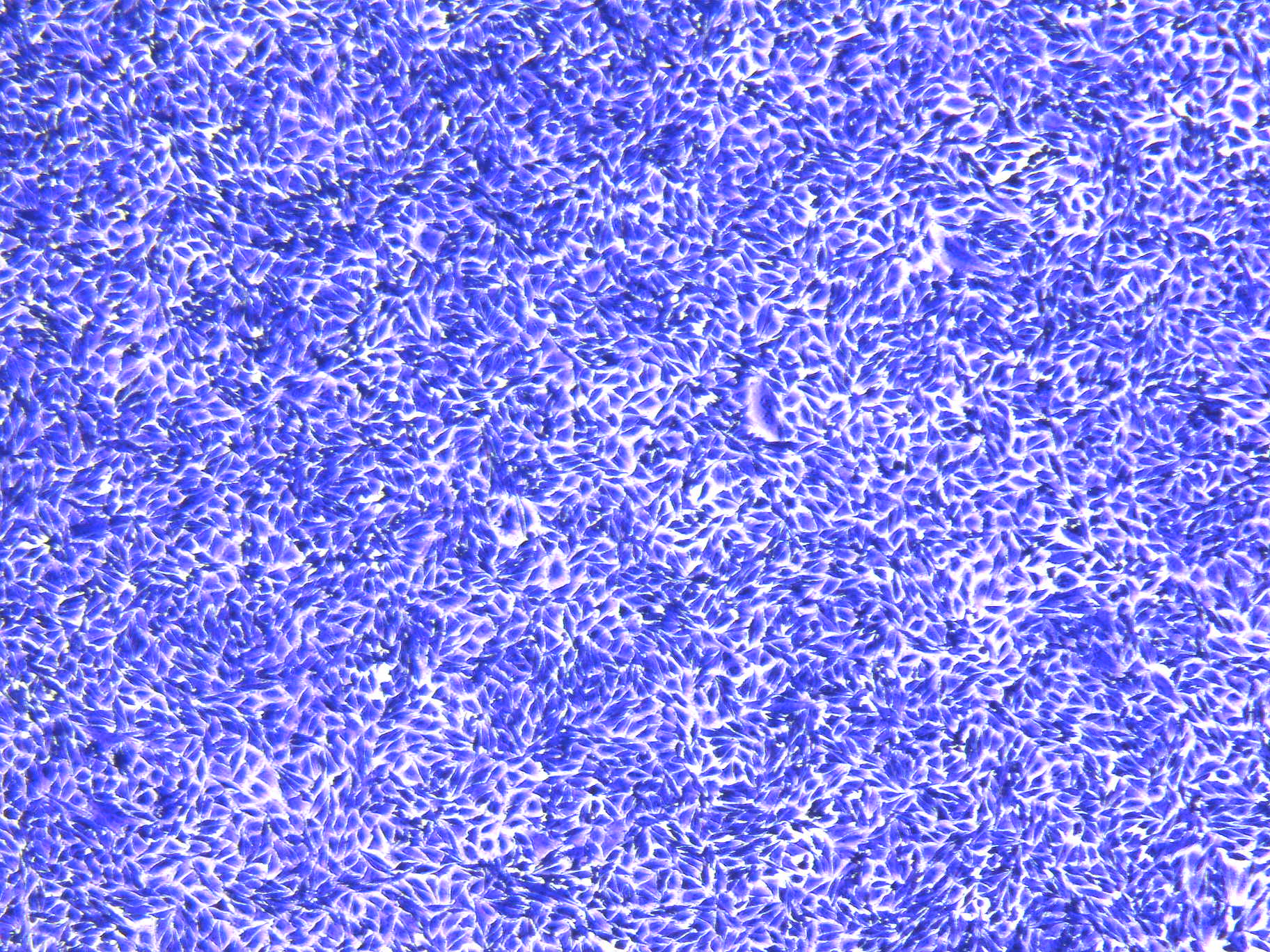

Supplement: Supplementary file 10 — EV figures [file 44321_2025_201_MOESM10_ESM.zip › source data for EV/EV4/EV4b CV/229/mock/LCL D3.JPG]

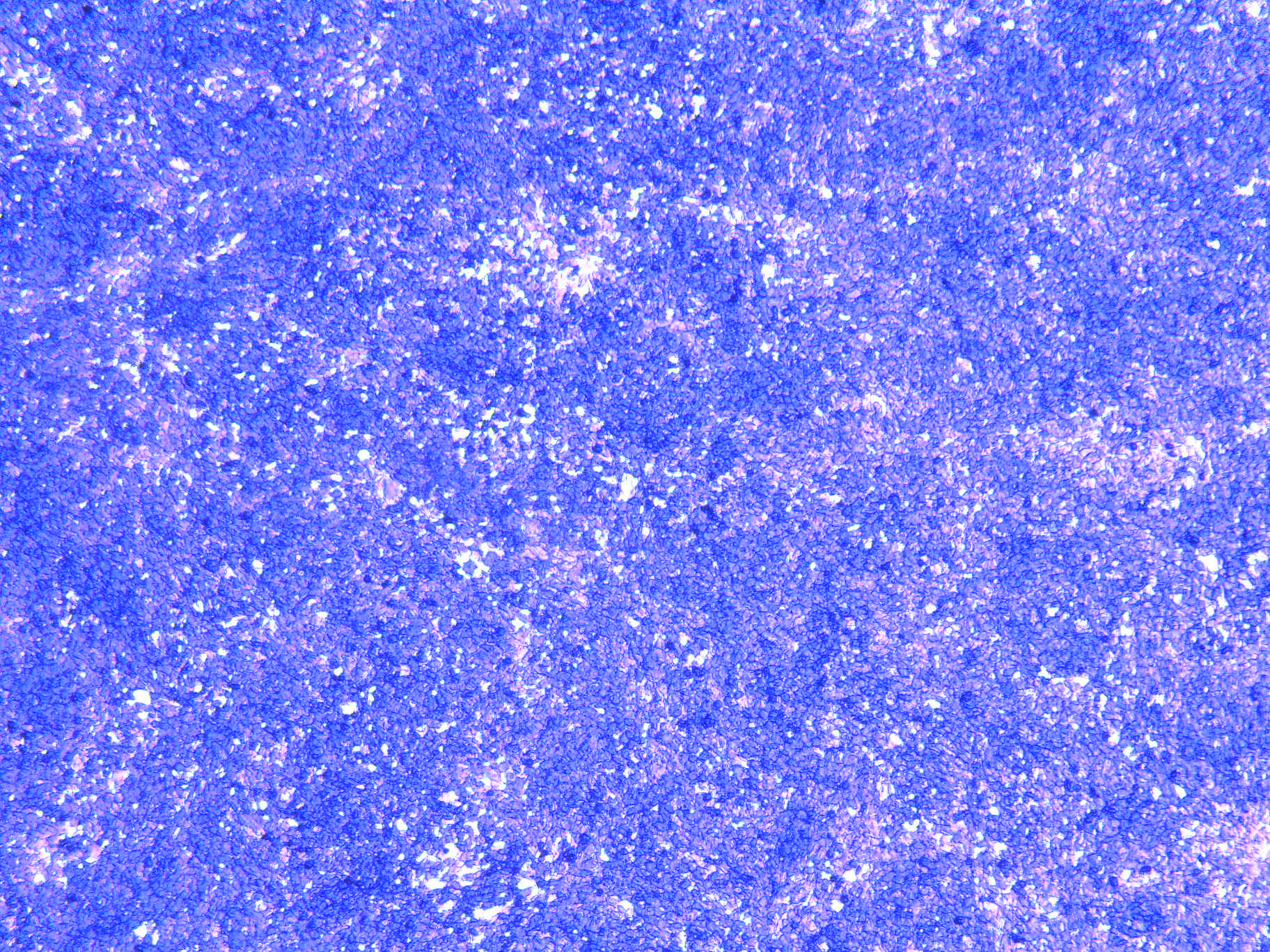

Supplement: Supplementary file 10 — EV figures [file 44321_2025_201_MOESM10_ESM.zip › source data for EV/EV4/EV4b CV/229/mock/LCL D6.JPG]

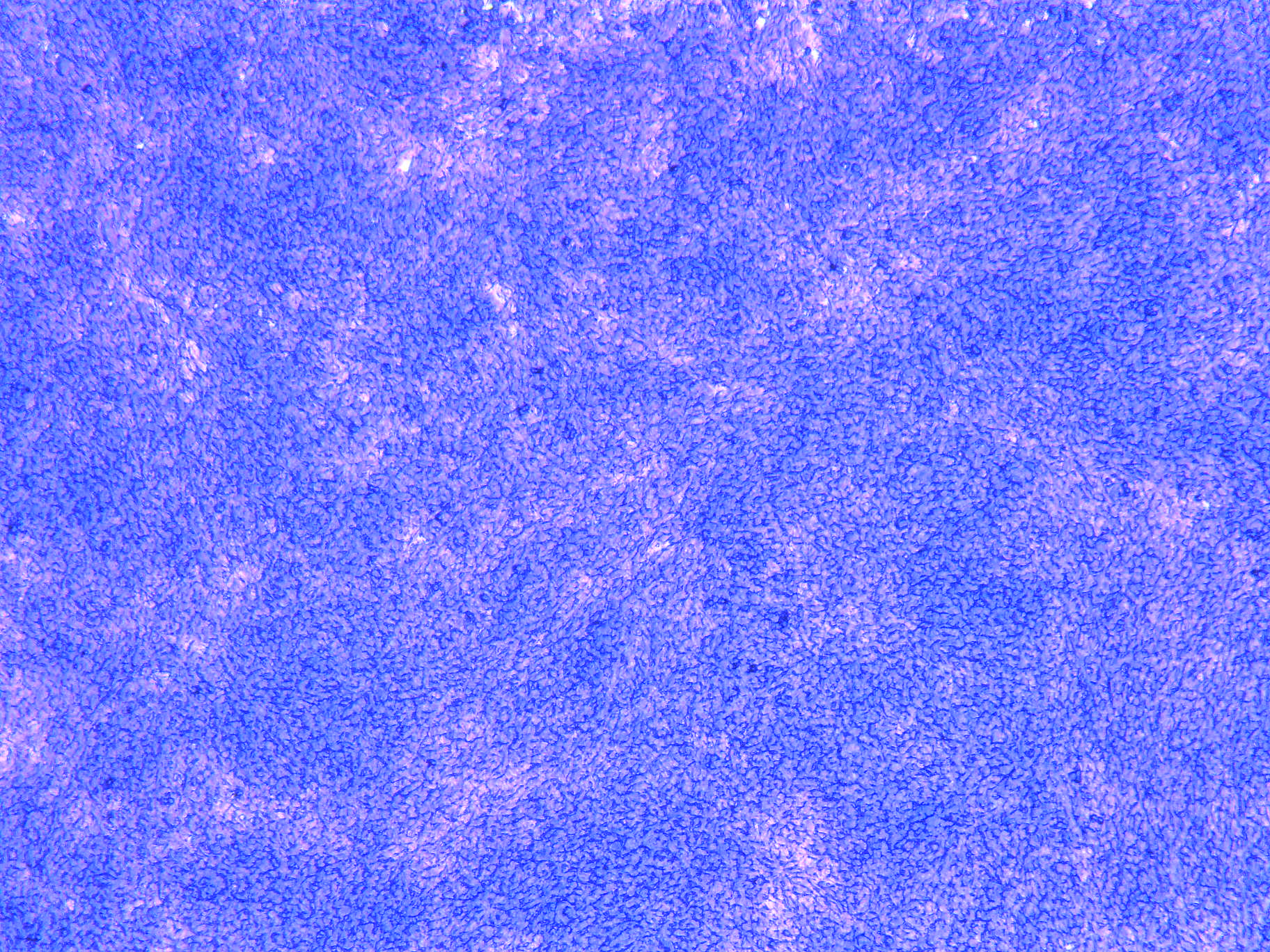

Supplement: Supplementary file 10 — EV figures [file 44321_2025_201_MOESM10_ESM.zip › source data for EV/EV4/EV4b CV/229/mock/LCL D9.JPG]

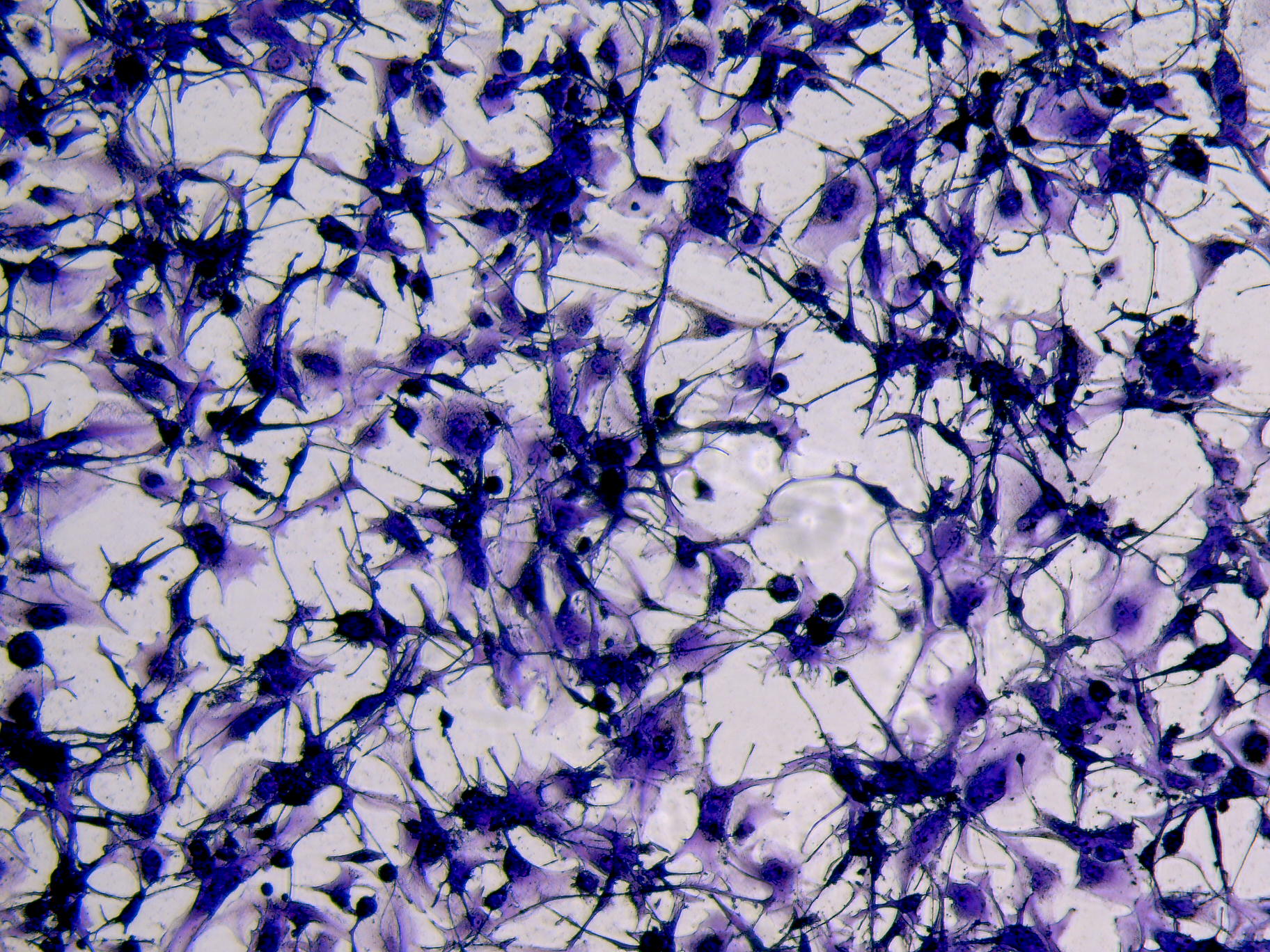

Supplement: Supplementary file 10 — EV figures [file 44321_2025_201_MOESM10_ESM.zip › source data for EV/EV4/EV4b CV/A172/IR/DMSO D0.JPG]

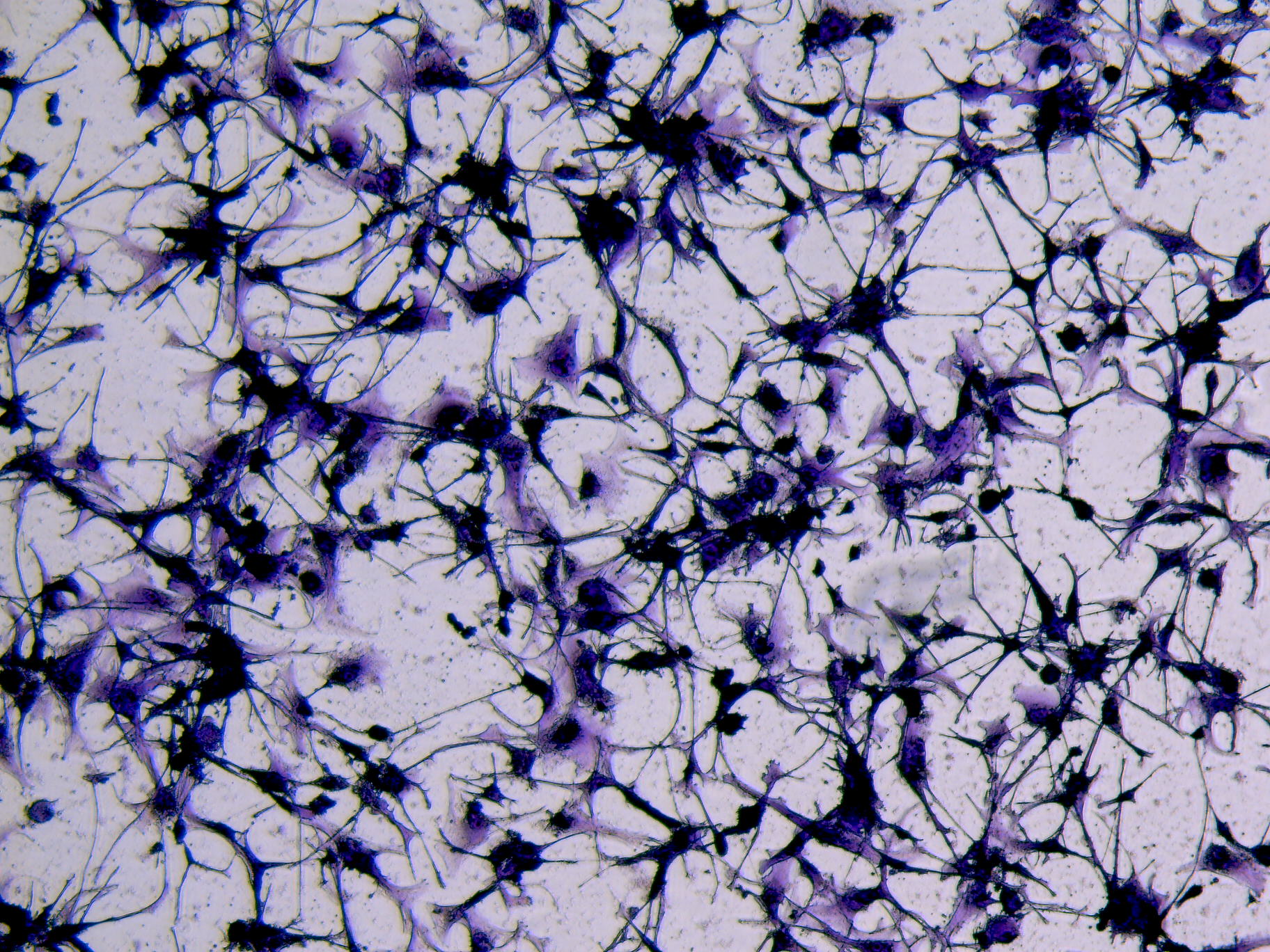

Supplement: Supplementary file 10 — EV figures [file 44321_2025_201_MOESM10_ESM.zip › source data for EV/EV4/EV4b CV/A172/IR/DMSO D3.JPG]

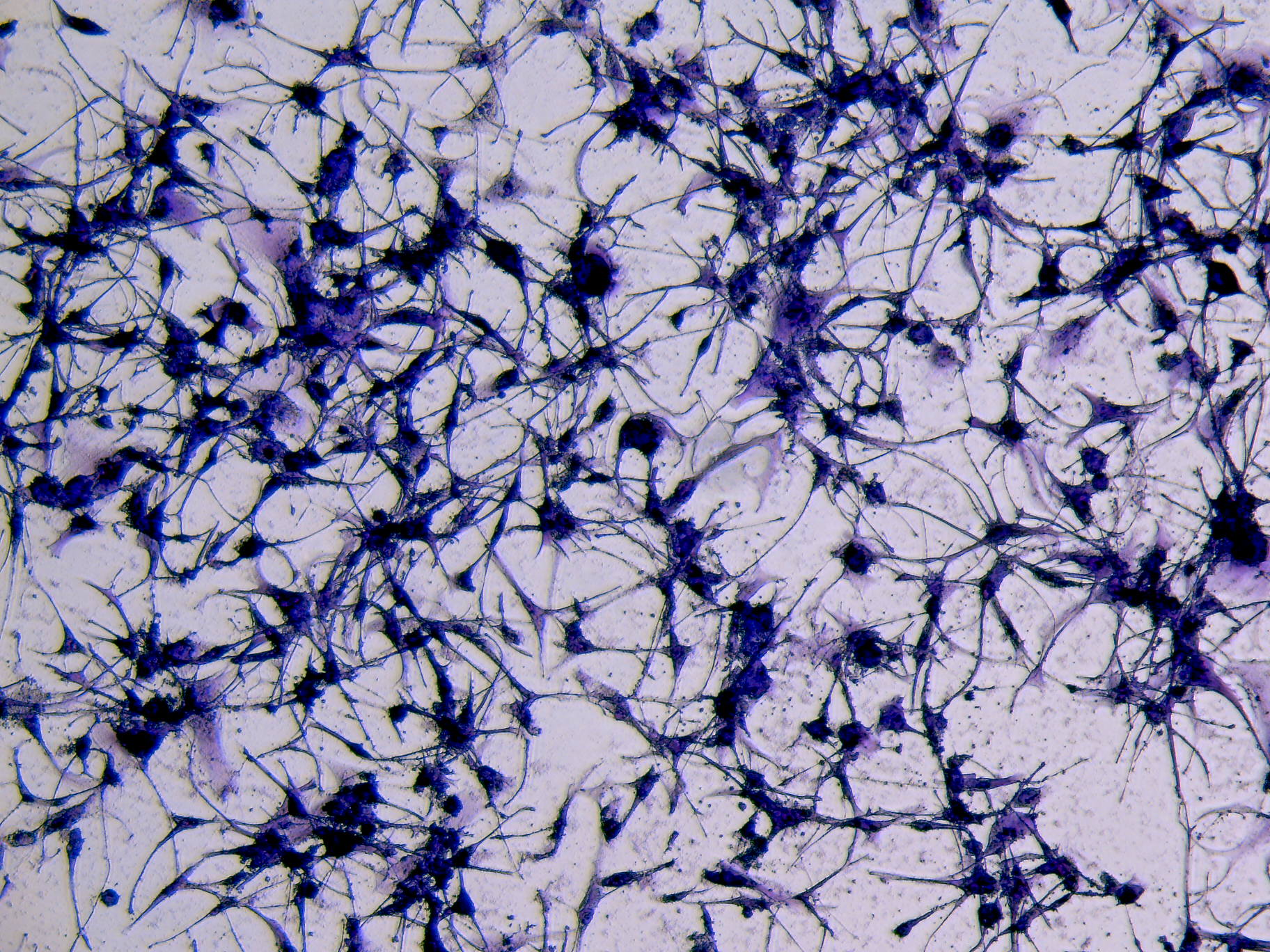

Supplement: Supplementary file 10 — EV figures [file 44321_2025_201_MOESM10_ESM.zip › source data for EV/EV4/EV4b CV/A172/IR/DMSO D6.JPG]

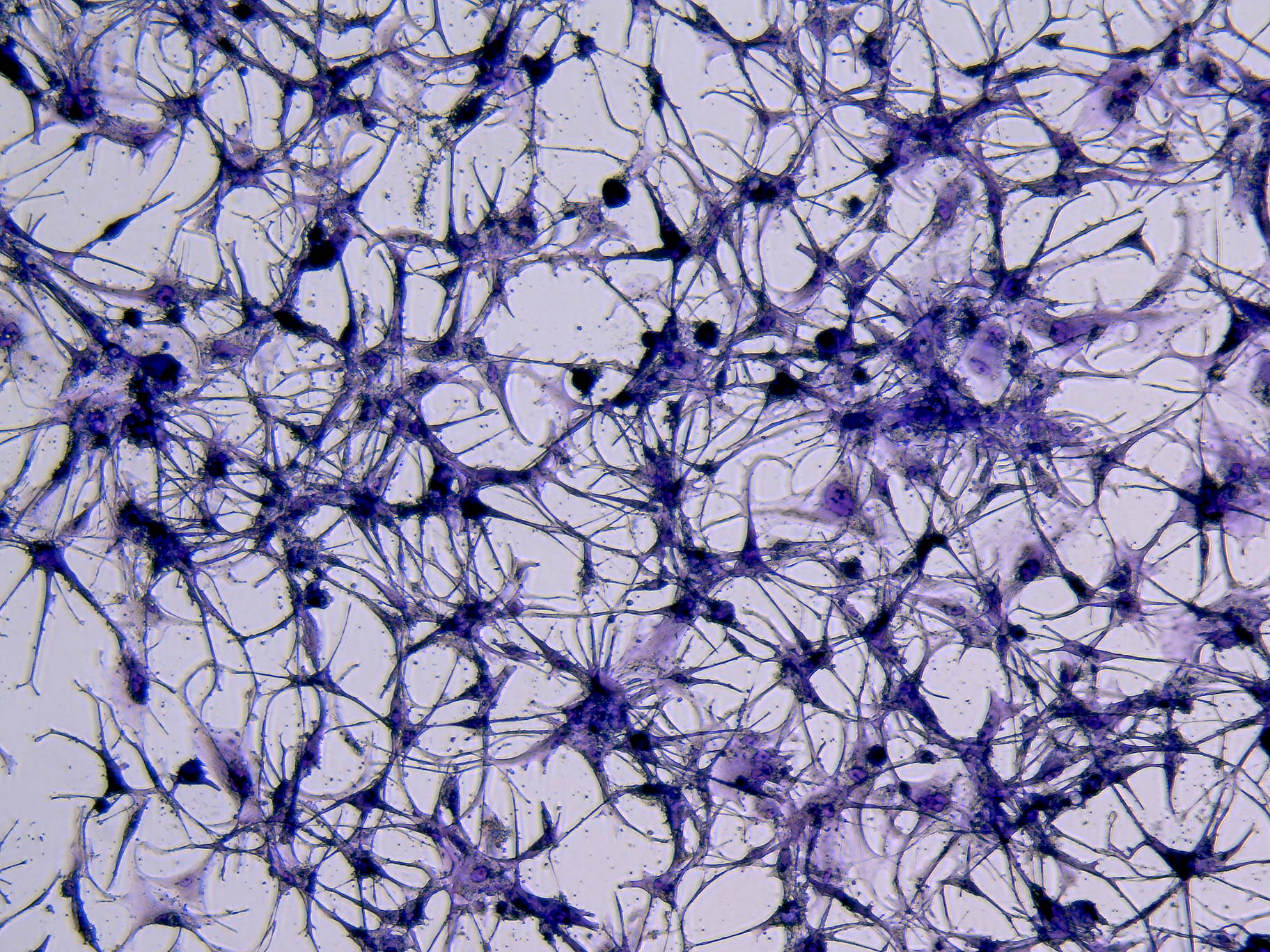

Supplement: Supplementary file 10 — EV figures [file 44321_2025_201_MOESM10_ESM.zip › source data for EV/EV4/EV4b CV/A172/IR/DMSO D9.JPG]

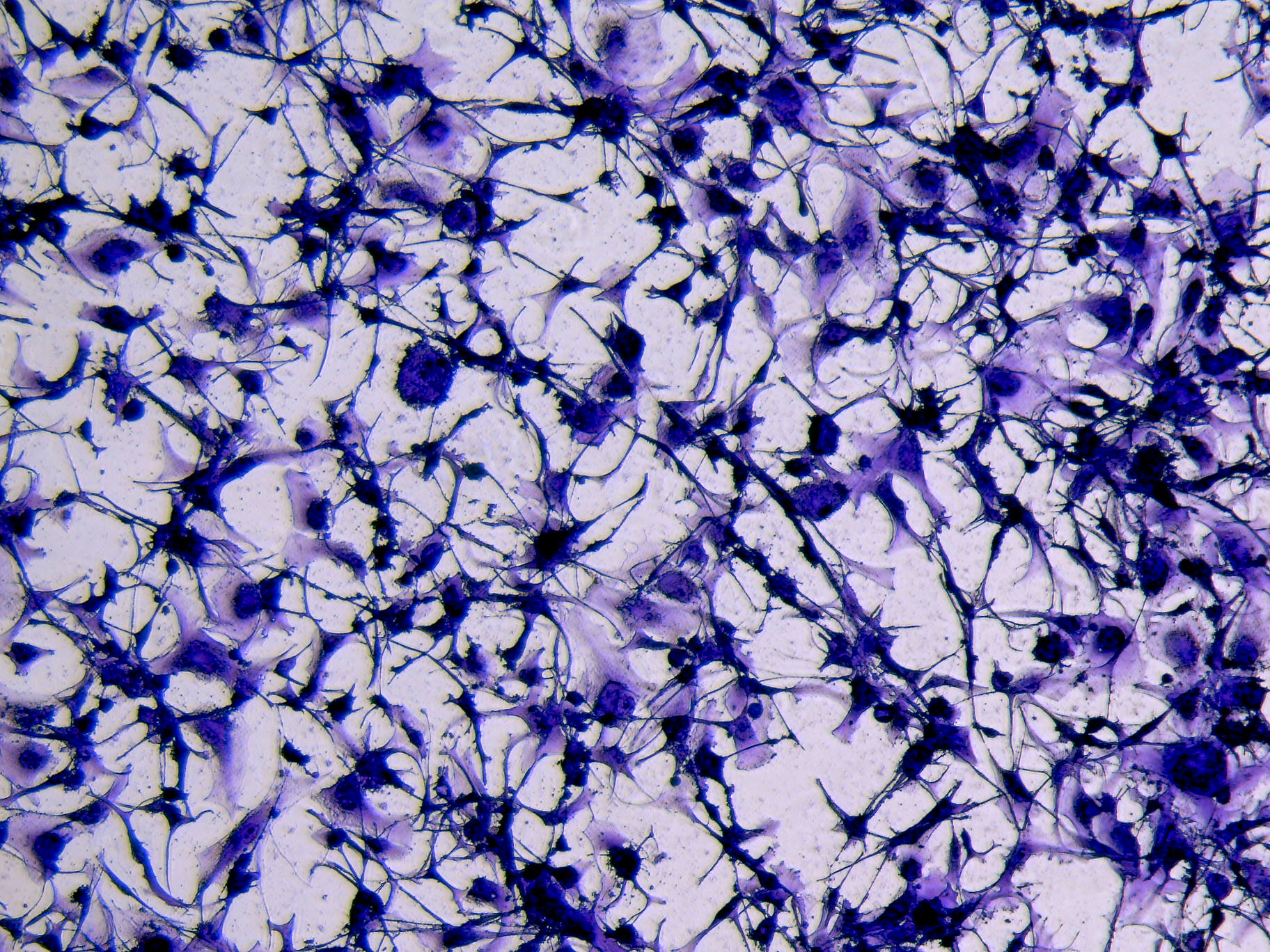

Supplement: Supplementary file 10 — EV figures [file 44321_2025_201_MOESM10_ESM.zip › source data for EV/EV4/EV4b CV/A172/IR/LCL D0.JPG]

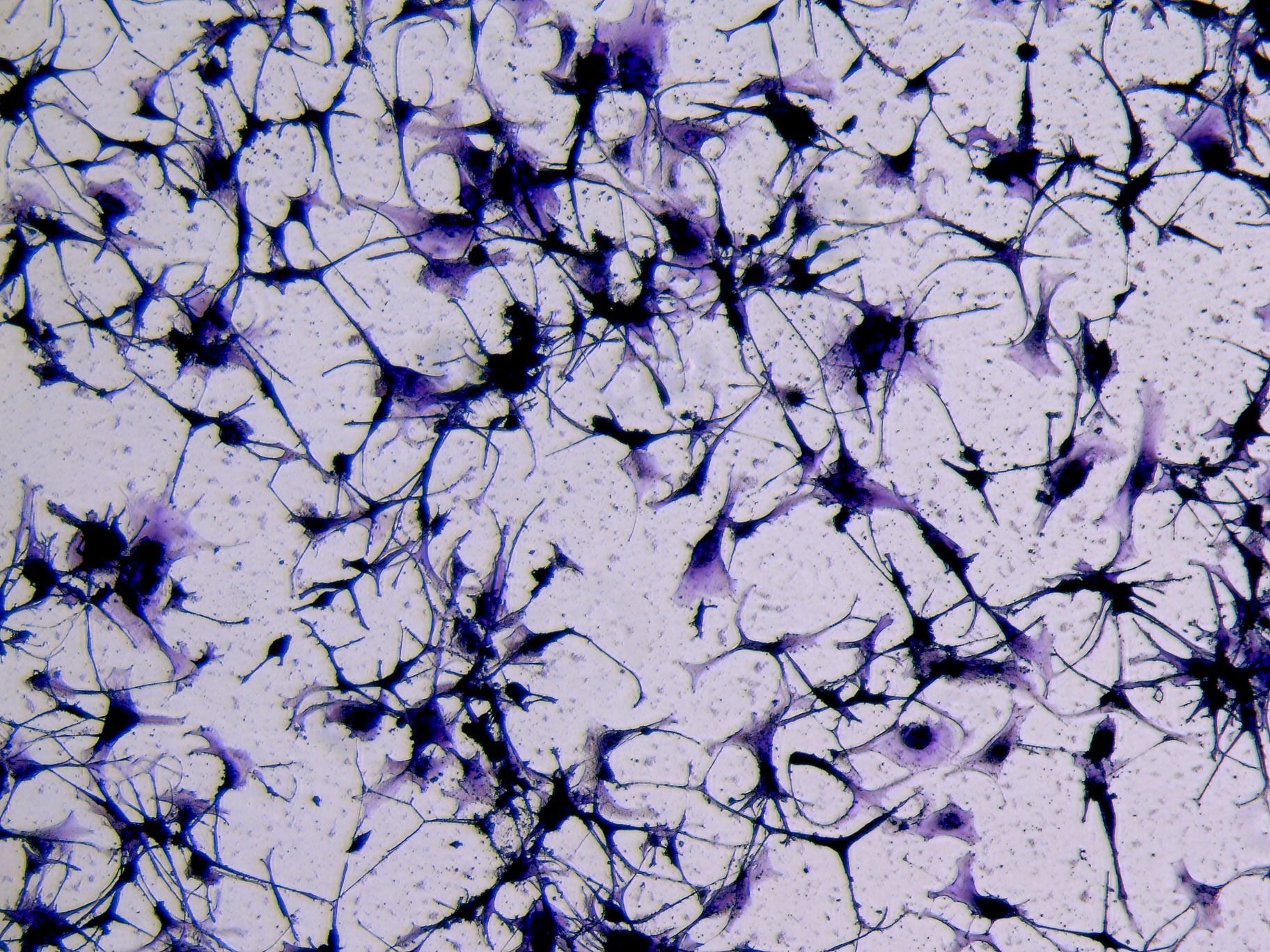

Supplement: Supplementary file 10 — EV figures [file 44321_2025_201_MOESM10_ESM.zip › source data for EV/EV4/EV4b CV/A172/IR/LCL D3.JPG]

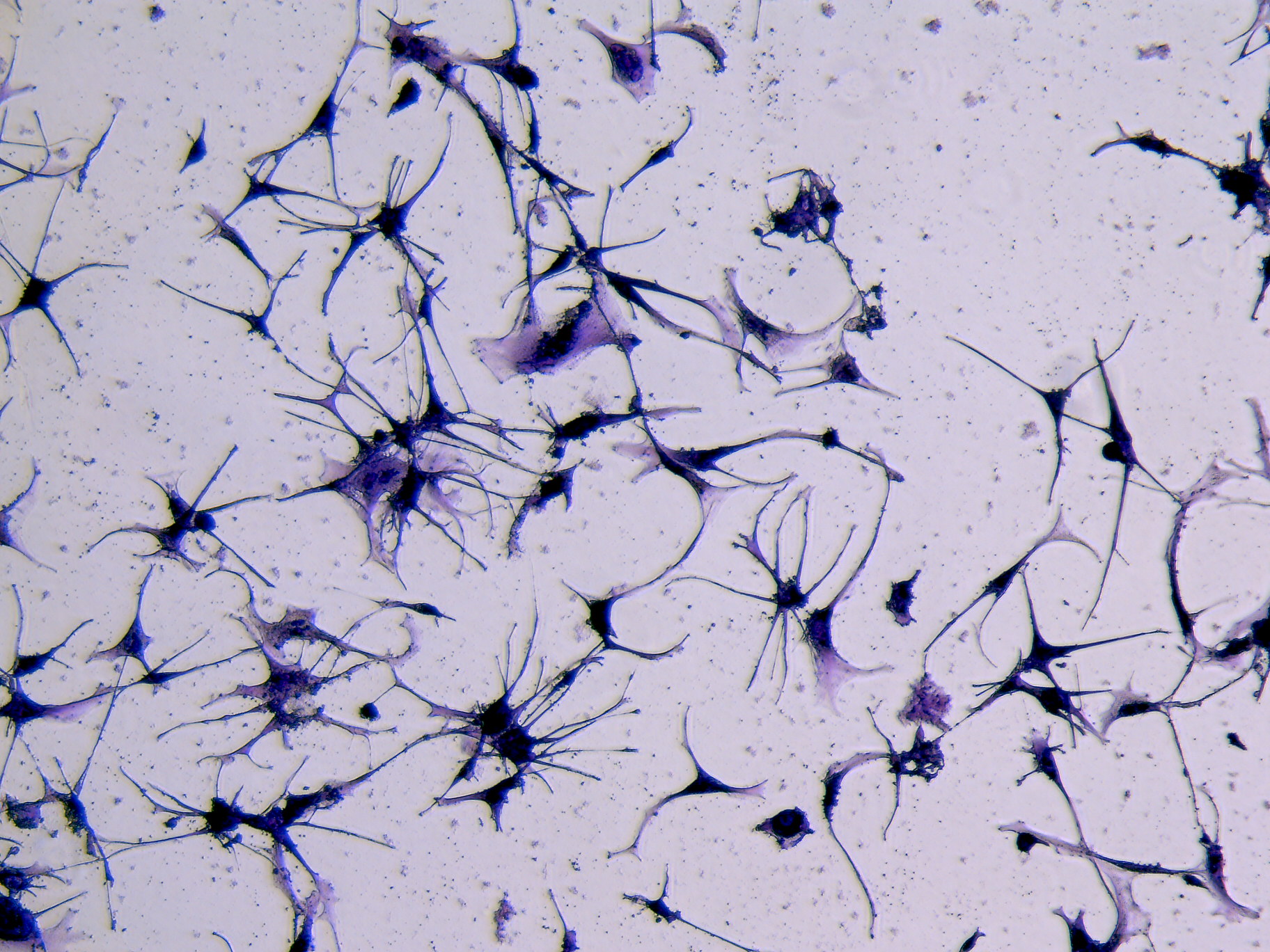

Supplement: Supplementary file 10 — EV figures [file 44321_2025_201_MOESM10_ESM.zip › source data for EV/EV4/EV4b CV/A172/IR/LCL D6.JPG]

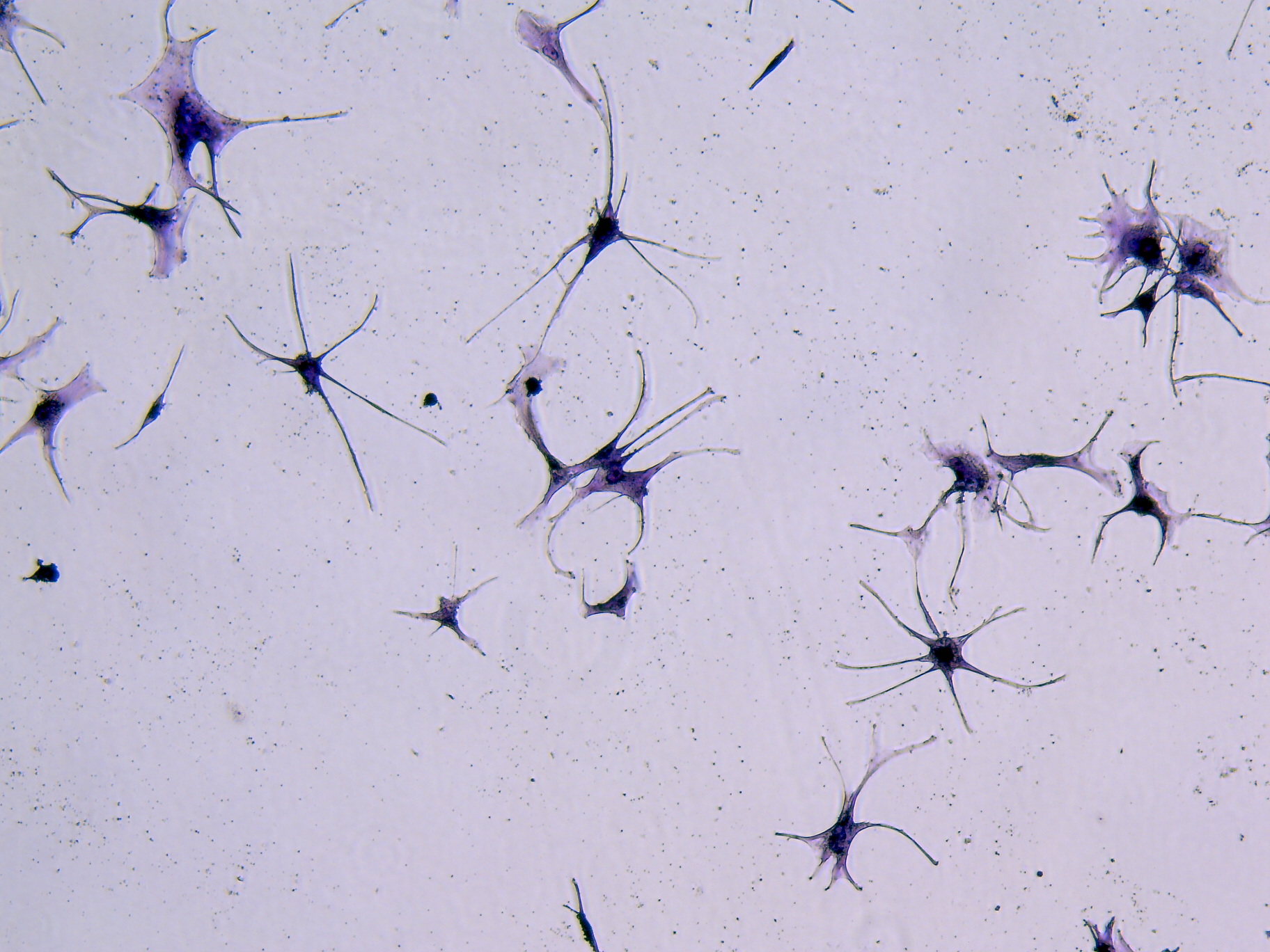

Supplement: Supplementary file 10 — EV figures [file 44321_2025_201_MOESM10_ESM.zip › source data for EV/EV4/EV4b CV/A172/IR/LCL D9.JPG]

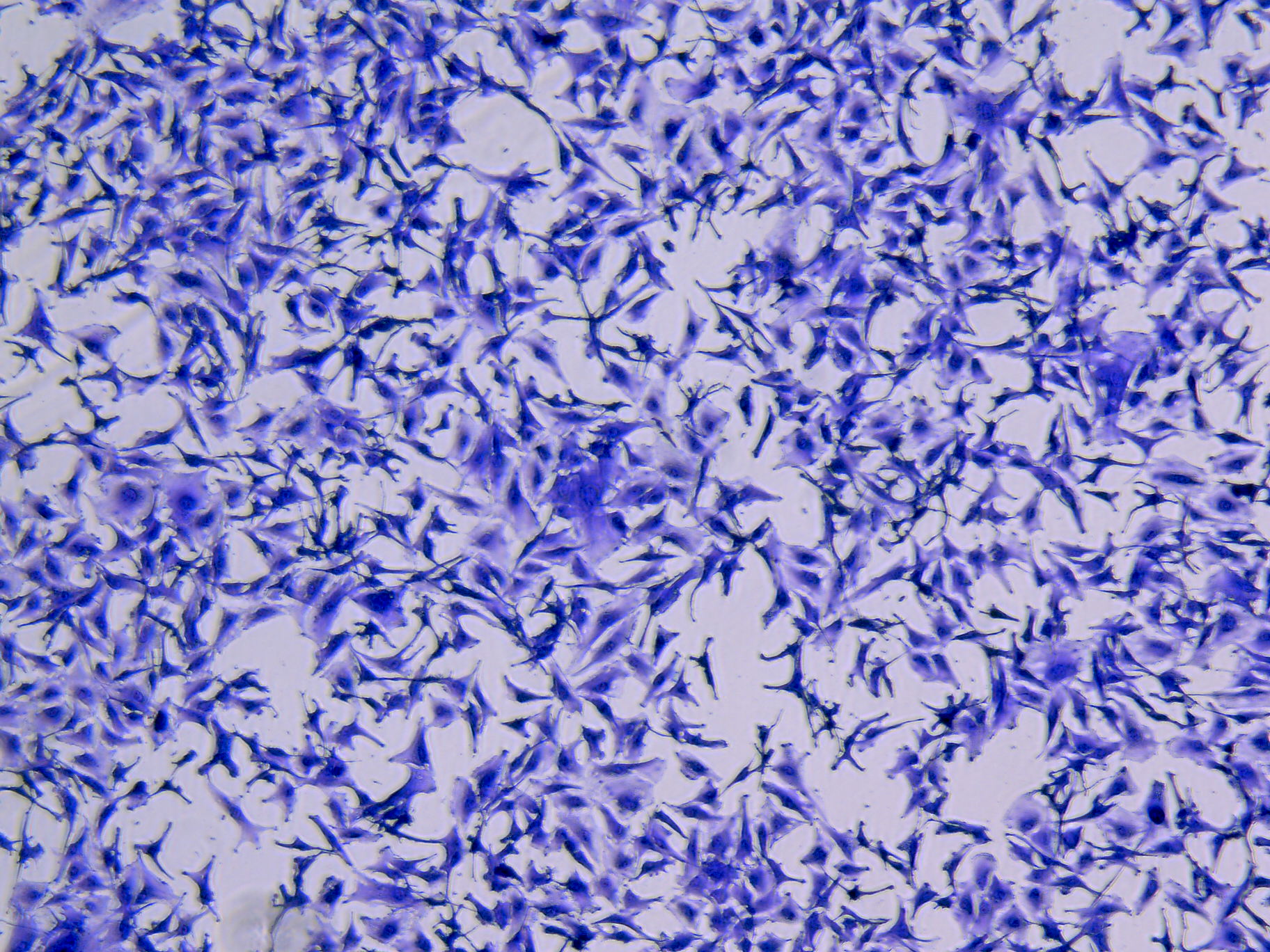

Supplement: Supplementary file 10 — EV figures [file 44321_2025_201_MOESM10_ESM.zip › source data for EV/EV4/EV4b CV/A172/mock/DMSO D0.JPG]

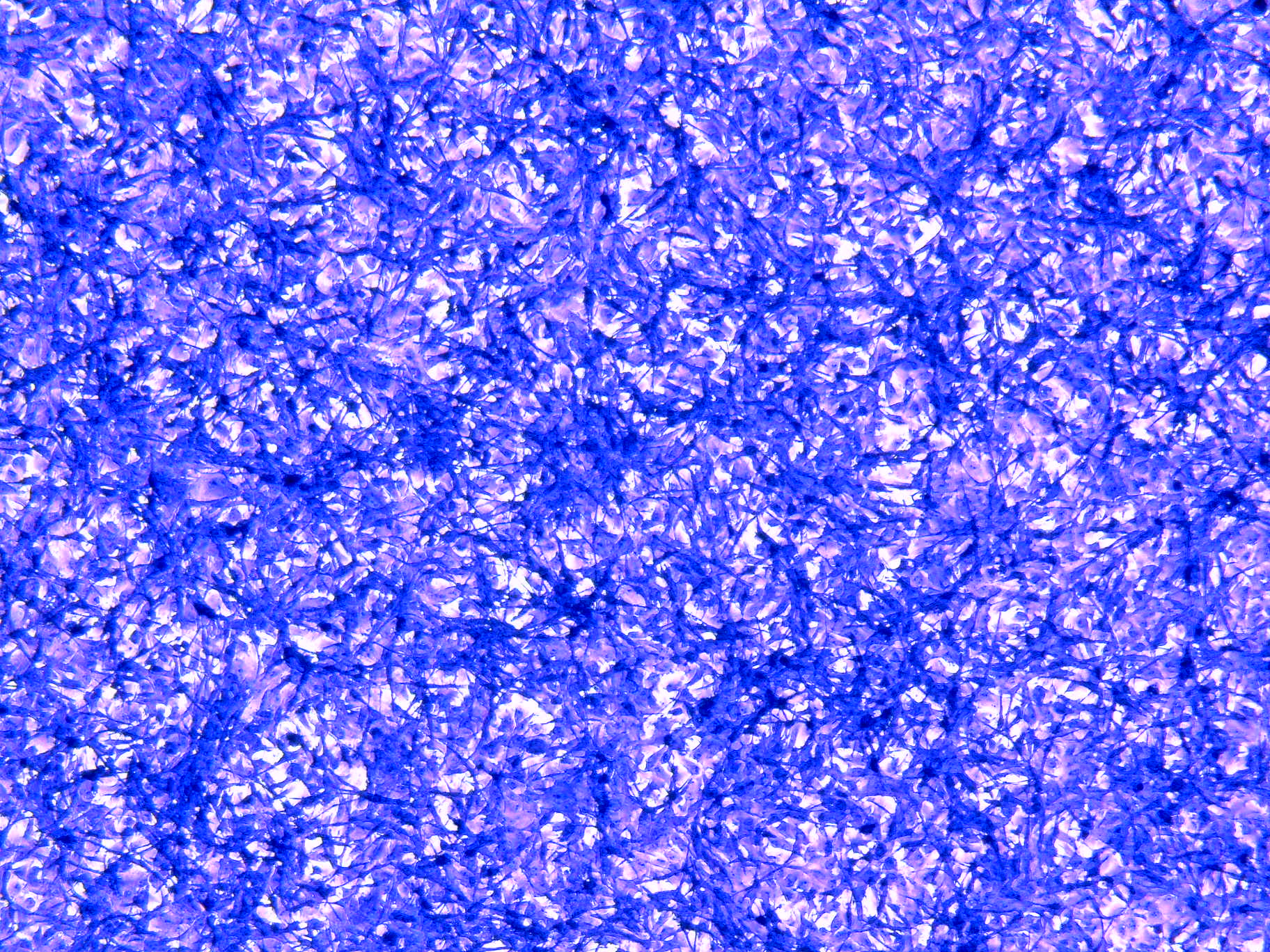

Supplement: Supplementary file 10 — EV figures [file 44321_2025_201_MOESM10_ESM.zip › source data for EV/EV4/EV4b CV/A172/mock/DMSO D3.JPG]

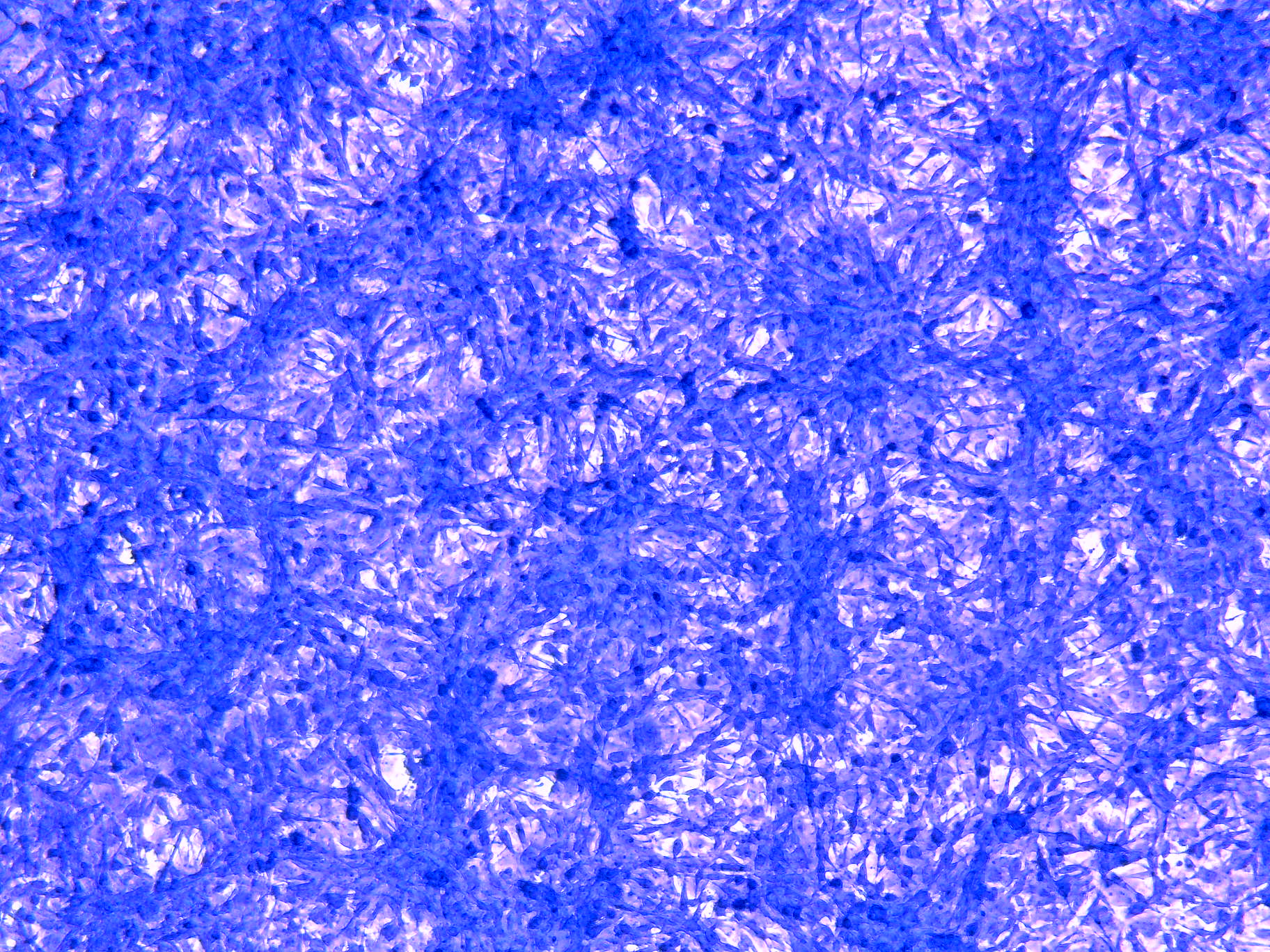

Supplement: Supplementary file 10 — EV figures [file 44321_2025_201_MOESM10_ESM.zip › source data for EV/EV4/EV4b CV/A172/mock/DMSO D6.JPG]

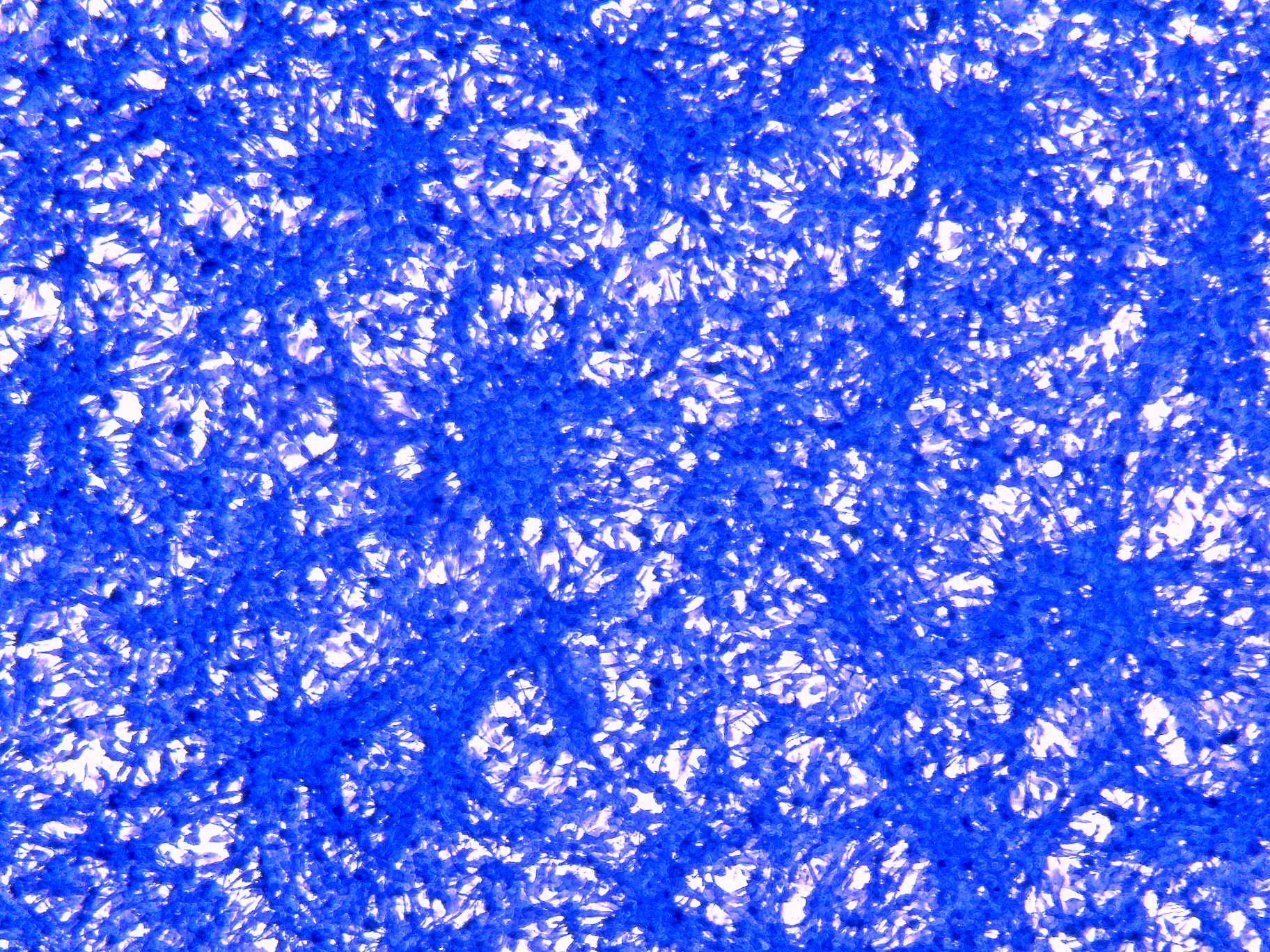

Supplement: Supplementary file 10 — EV figures [file 44321_2025_201_MOESM10_ESM.zip › source data for EV/EV4/EV4b CV/A172/mock/DMSO D9.JPG]

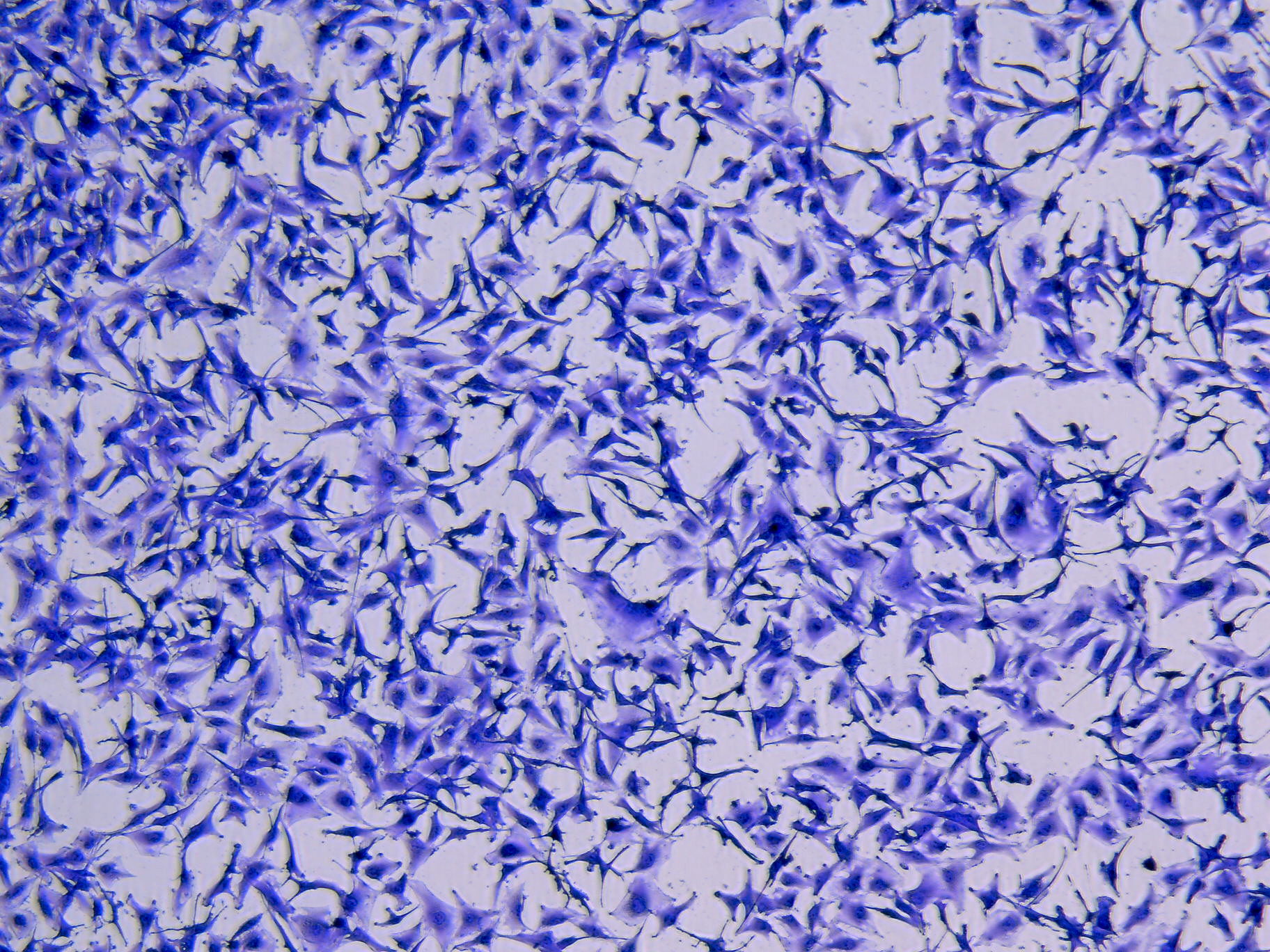

Supplement: Supplementary file 10 — EV figures [file 44321_2025_201_MOESM10_ESM.zip › source data for EV/EV4/EV4b CV/A172/mock/LCL D0.JPG]

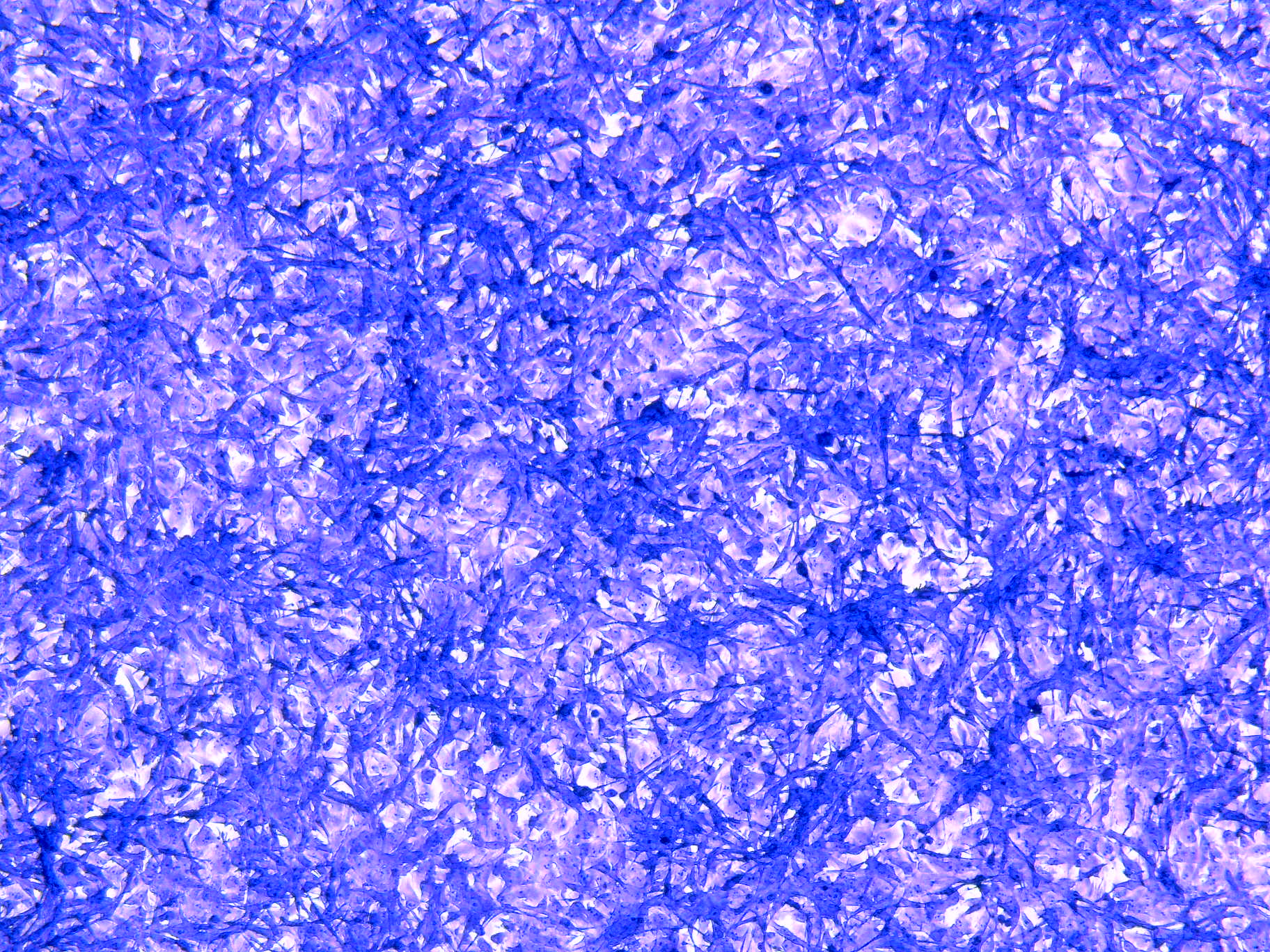

Supplement: Supplementary file 10 — EV figures [file 44321_2025_201_MOESM10_ESM.zip › source data for EV/EV4/EV4b CV/A172/mock/LCL D3.JPG]

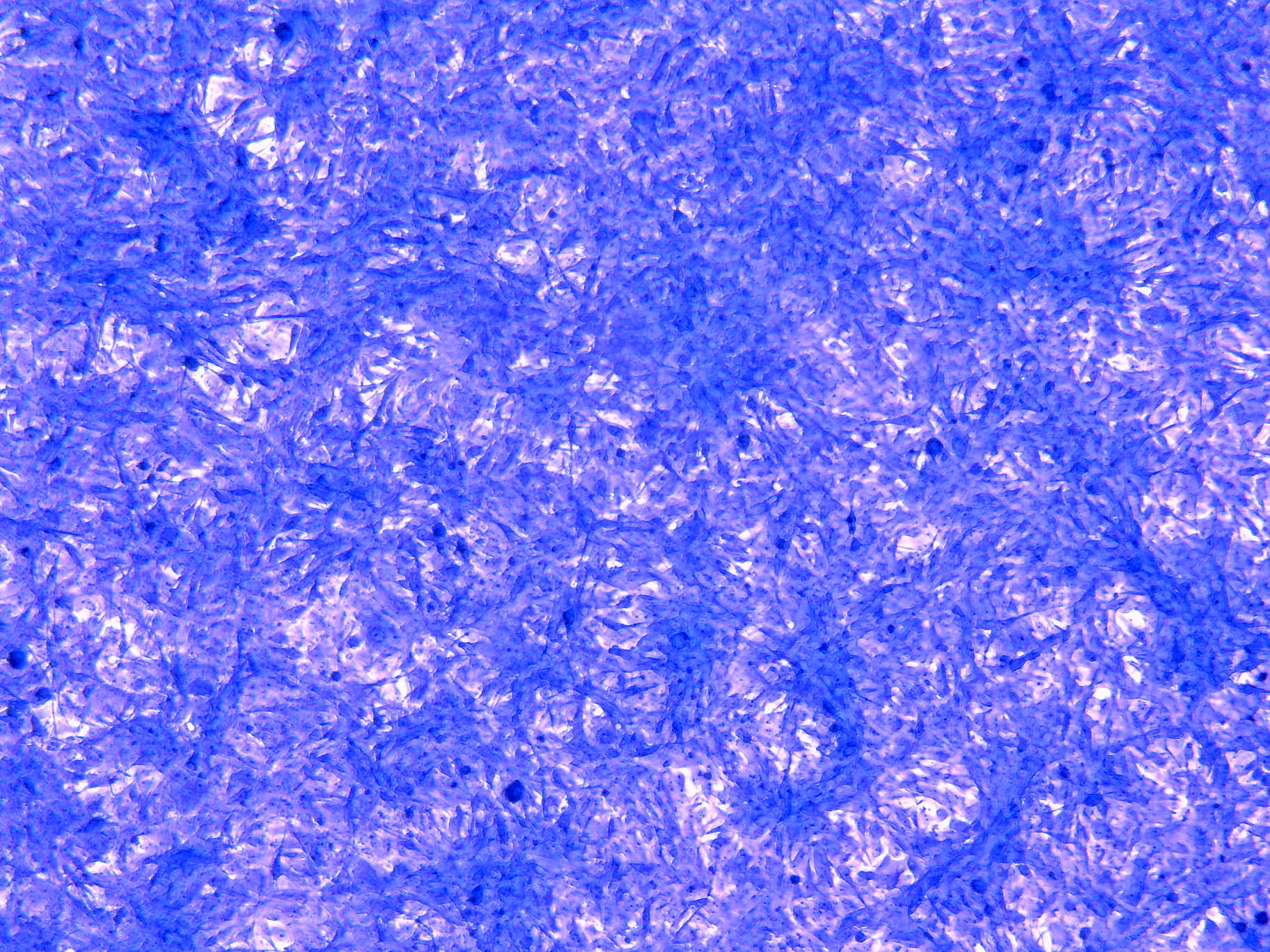

Supplement: Supplementary file 10 — EV figures [file 44321_2025_201_MOESM10_ESM.zip › source data for EV/EV4/EV4b CV/A172/mock/LCL D6.JPG]

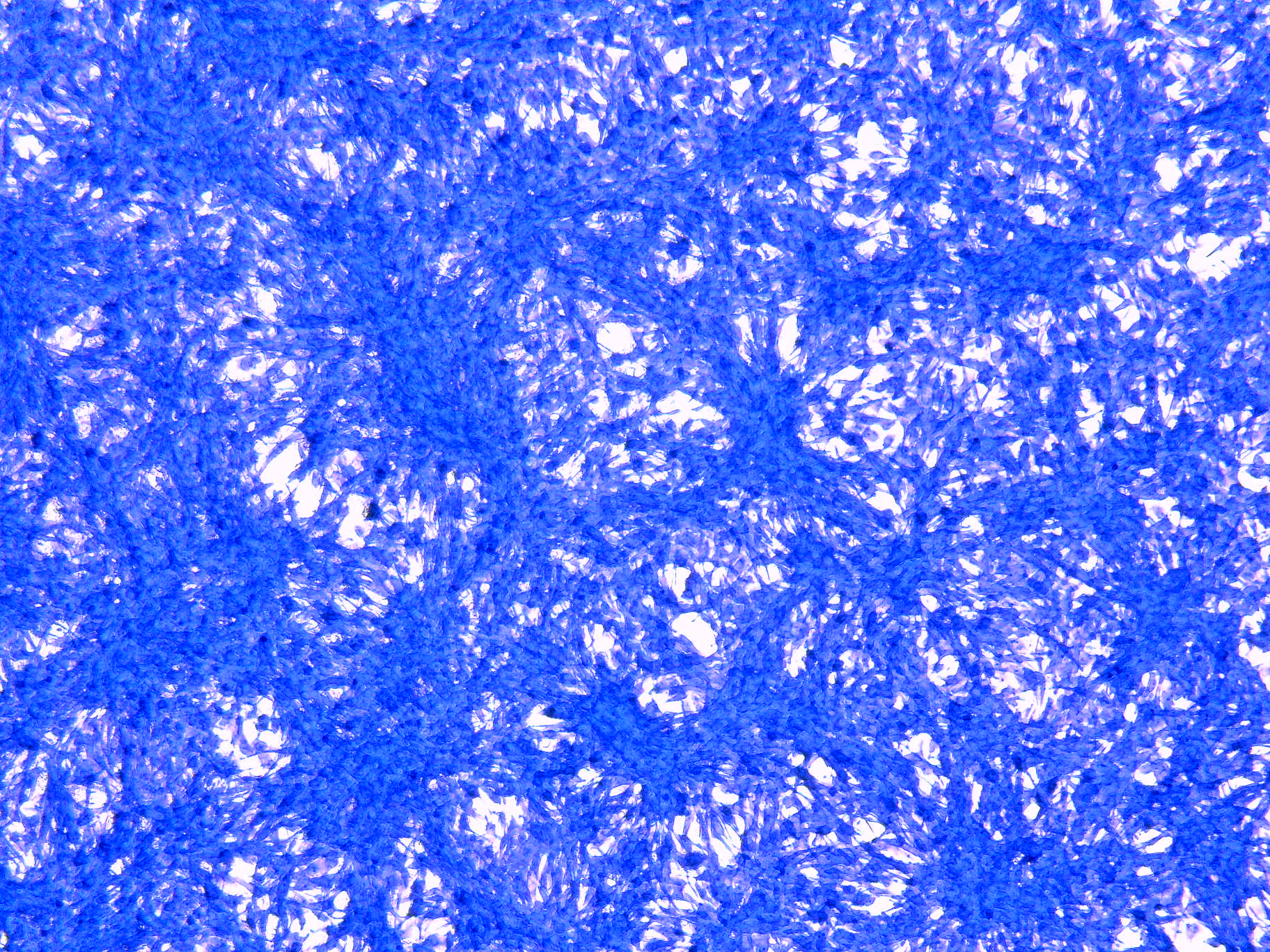

Supplement: Supplementary file 10 — EV figures [file 44321_2025_201_MOESM10_ESM.zip › source data for EV/EV4/EV4b CV/A172/mock/LCL D9.JPG]

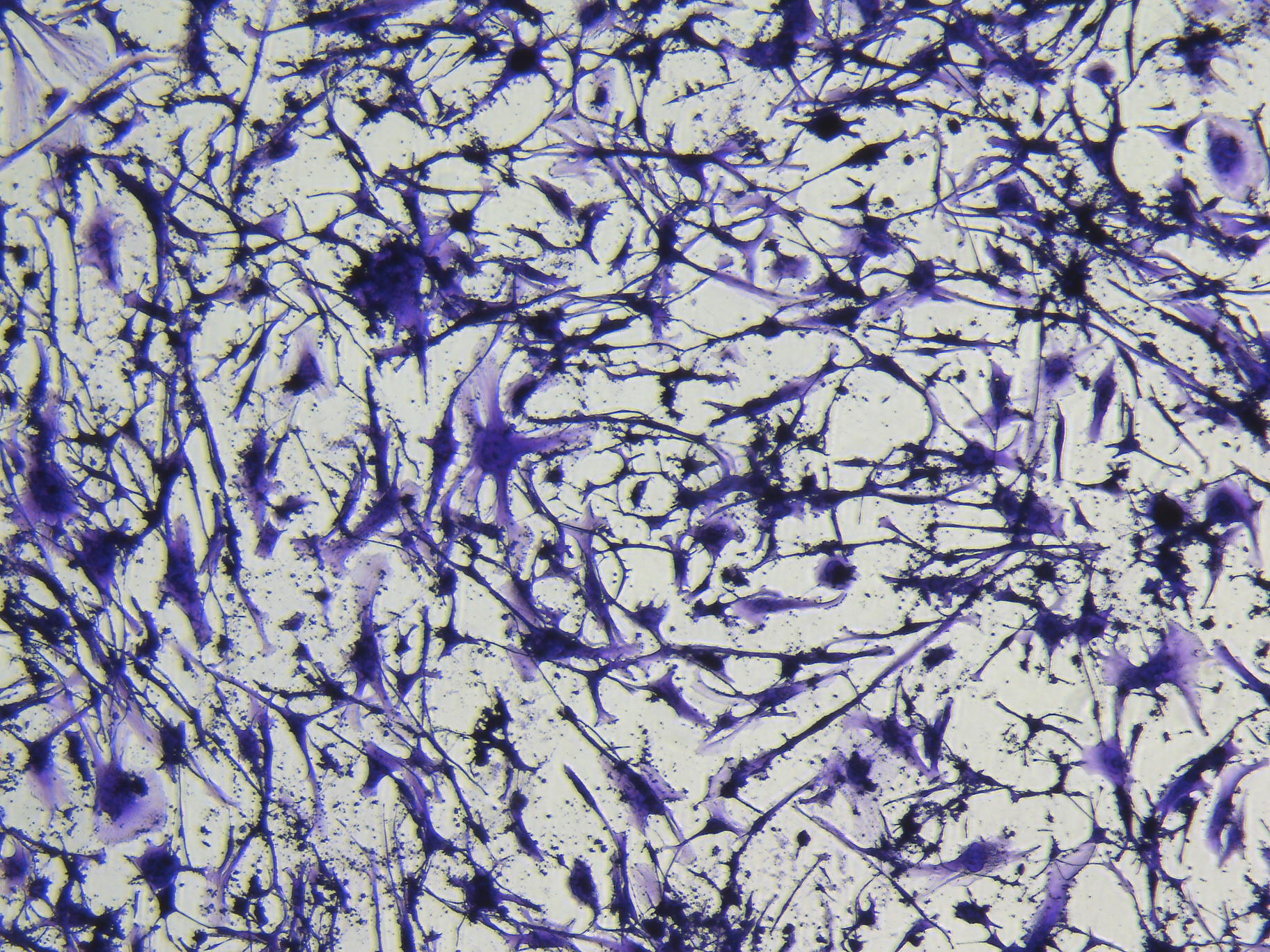

Supplement: Supplementary file 10 — EV figures [file 44321_2025_201_MOESM10_ESM.zip › source data for EV/EV4/EV4b CV/U118/IR/DMSO D0.JPG]

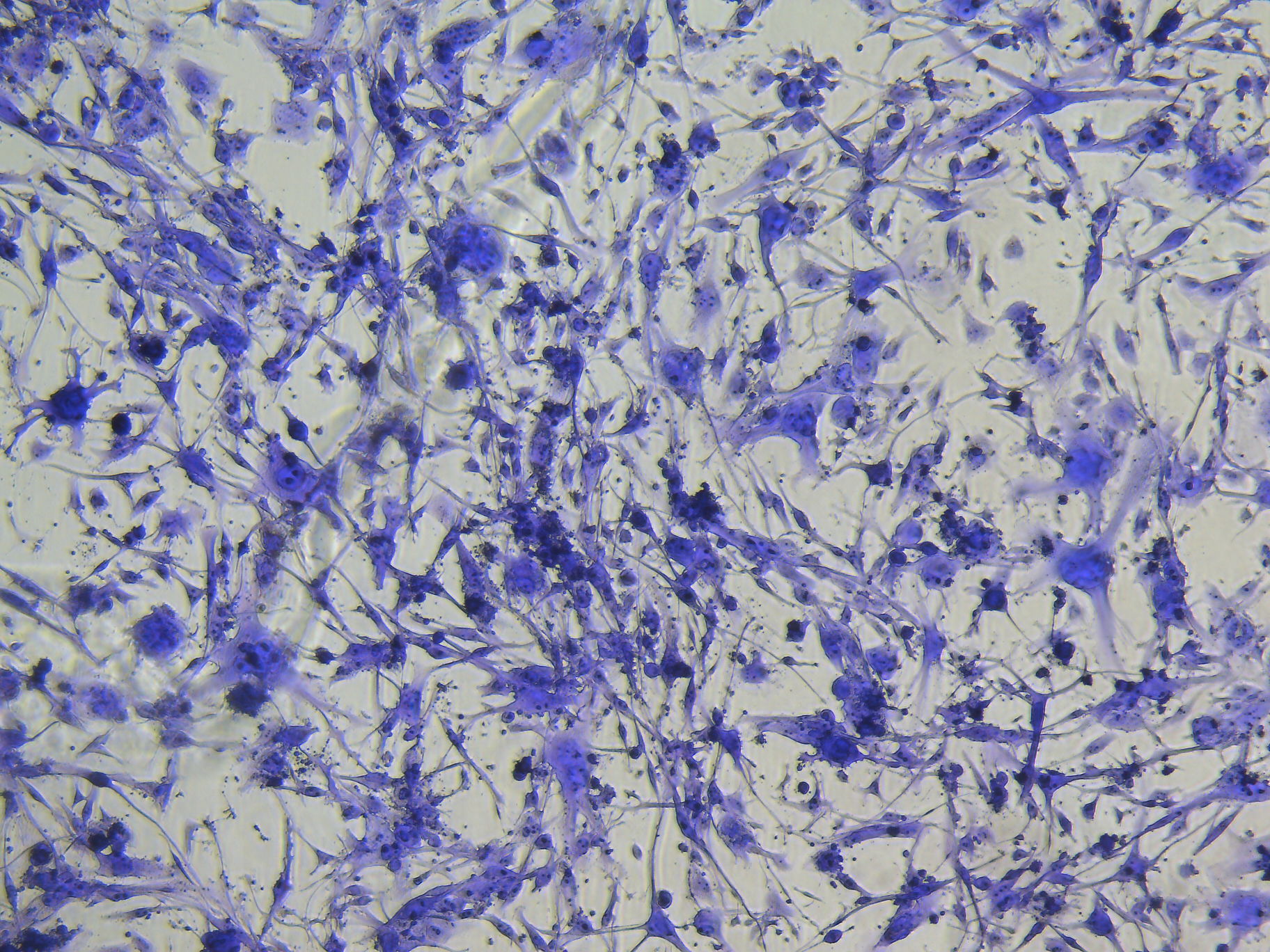

Supplement: Supplementary file 10 — EV figures [file 44321_2025_201_MOESM10_ESM.zip › source data for EV/EV4/EV4b CV/U118/IR/DMSO D3.JPG]

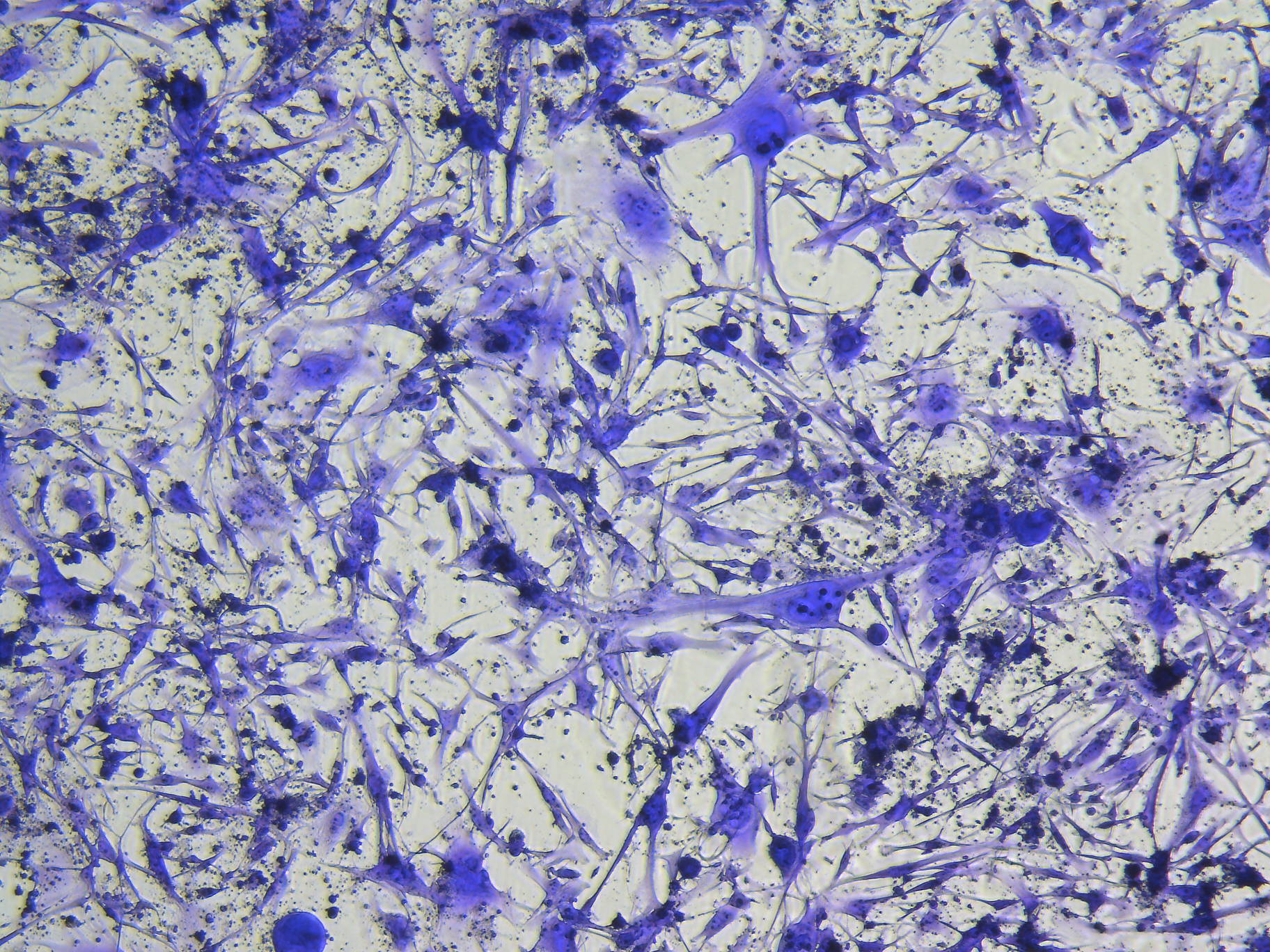

Supplement: Supplementary file 10 — EV figures [file 44321_2025_201_MOESM10_ESM.zip › source data for EV/EV4/EV4b CV/U118/IR/DMSO D6.JPG]

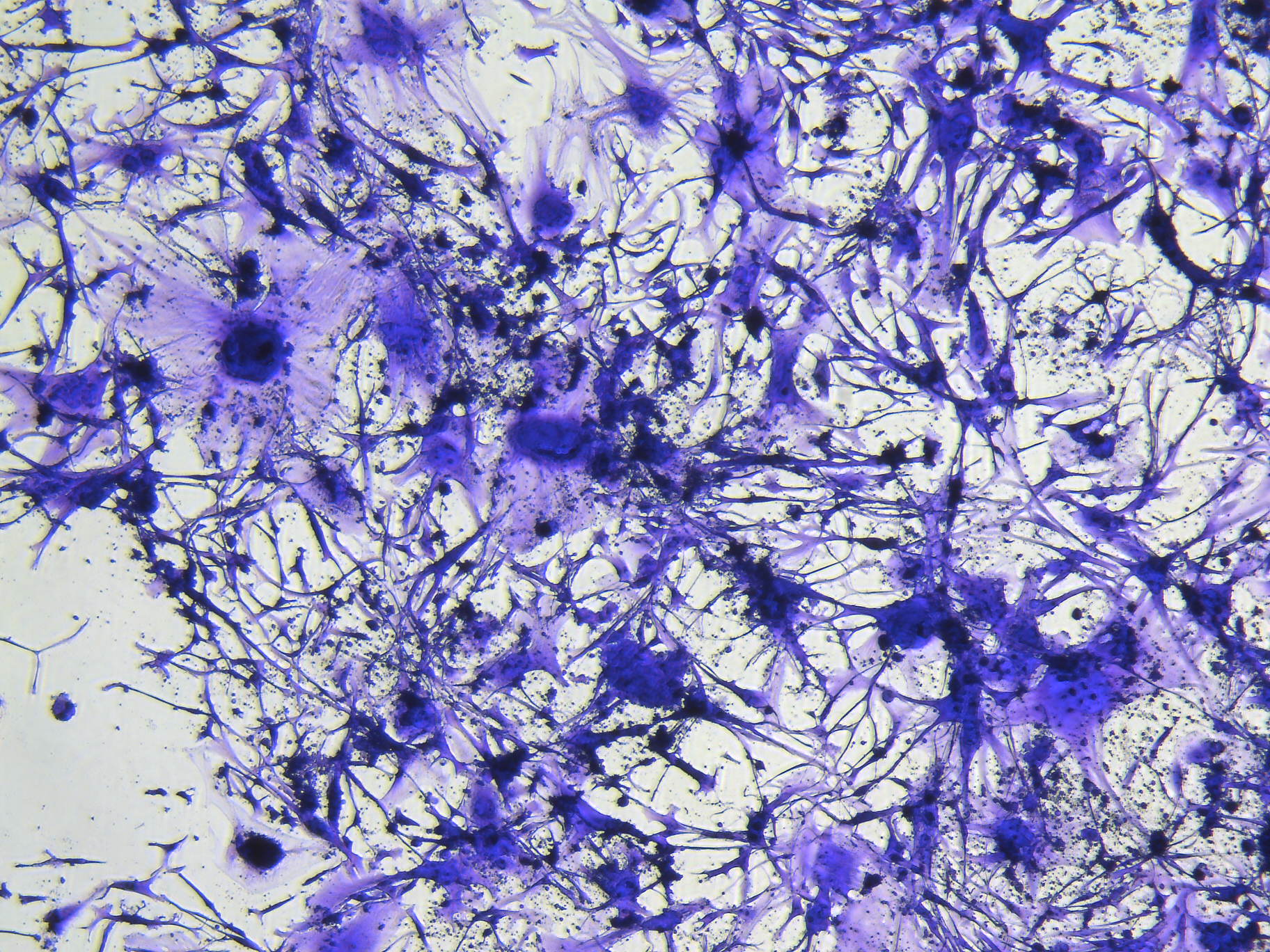

Supplement: Supplementary file 10 — EV figures [file 44321_2025_201_MOESM10_ESM.zip › source data for EV/EV4/EV4b CV/U118/IR/DMSO D9.JPG]

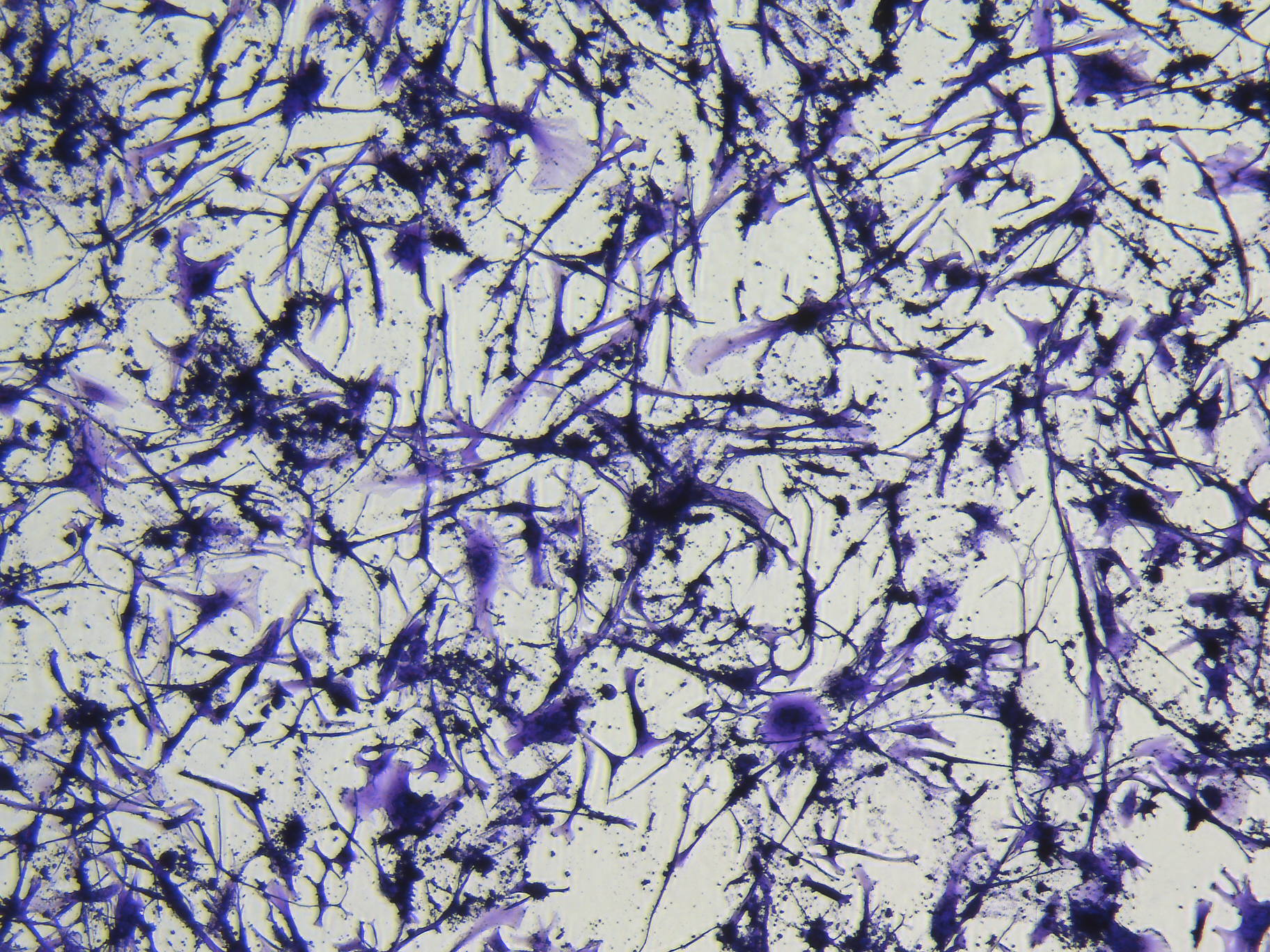

Supplement: Supplementary file 10 — EV figures [file 44321_2025_201_MOESM10_ESM.zip › source data for EV/EV4/EV4b CV/U118/IR/LCL D0.JPG]

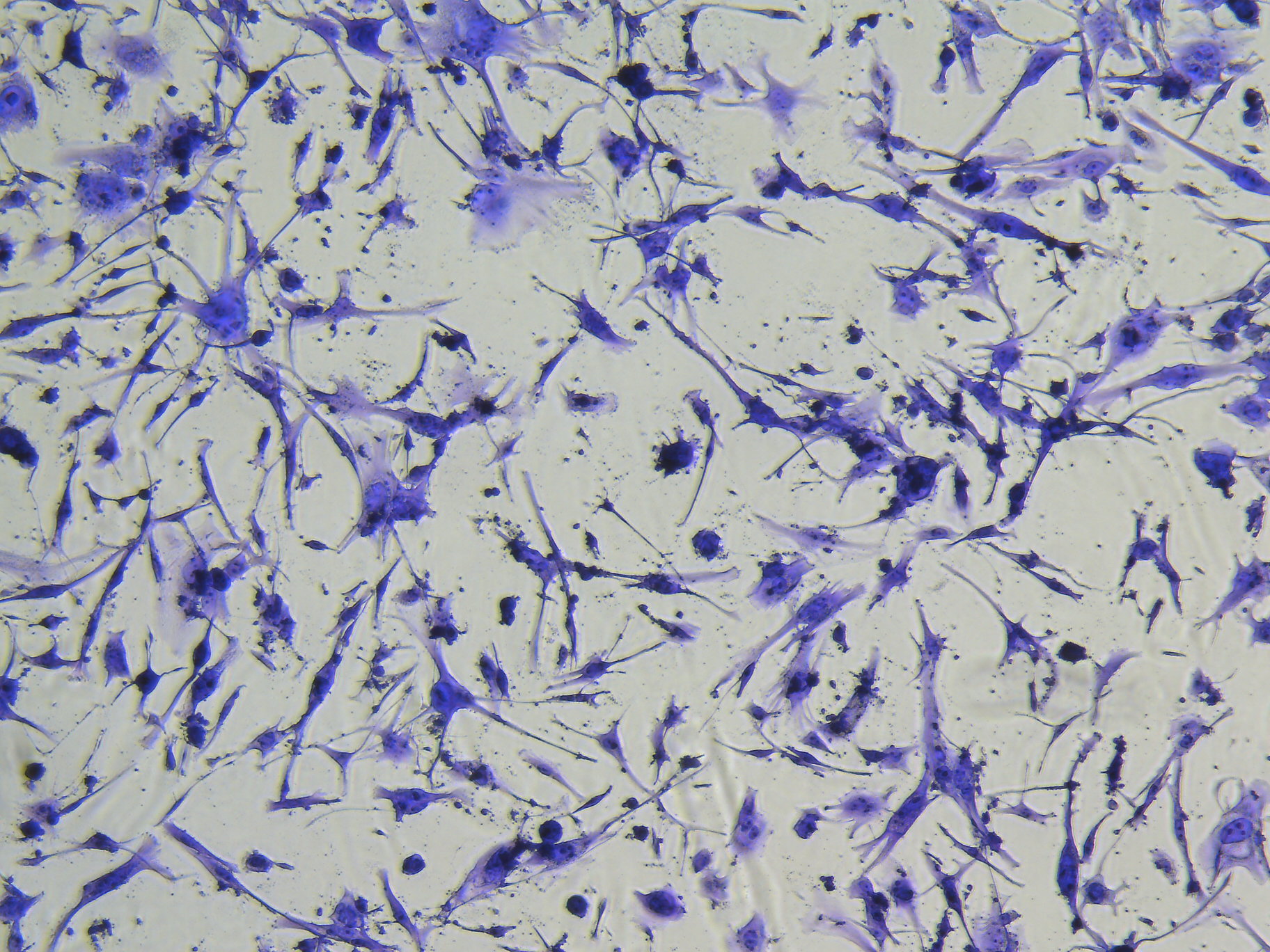

Supplement: Supplementary file 10 — EV figures [file 44321_2025_201_MOESM10_ESM.zip › source data for EV/EV4/EV4b CV/U118/IR/LCL D3.JPG]

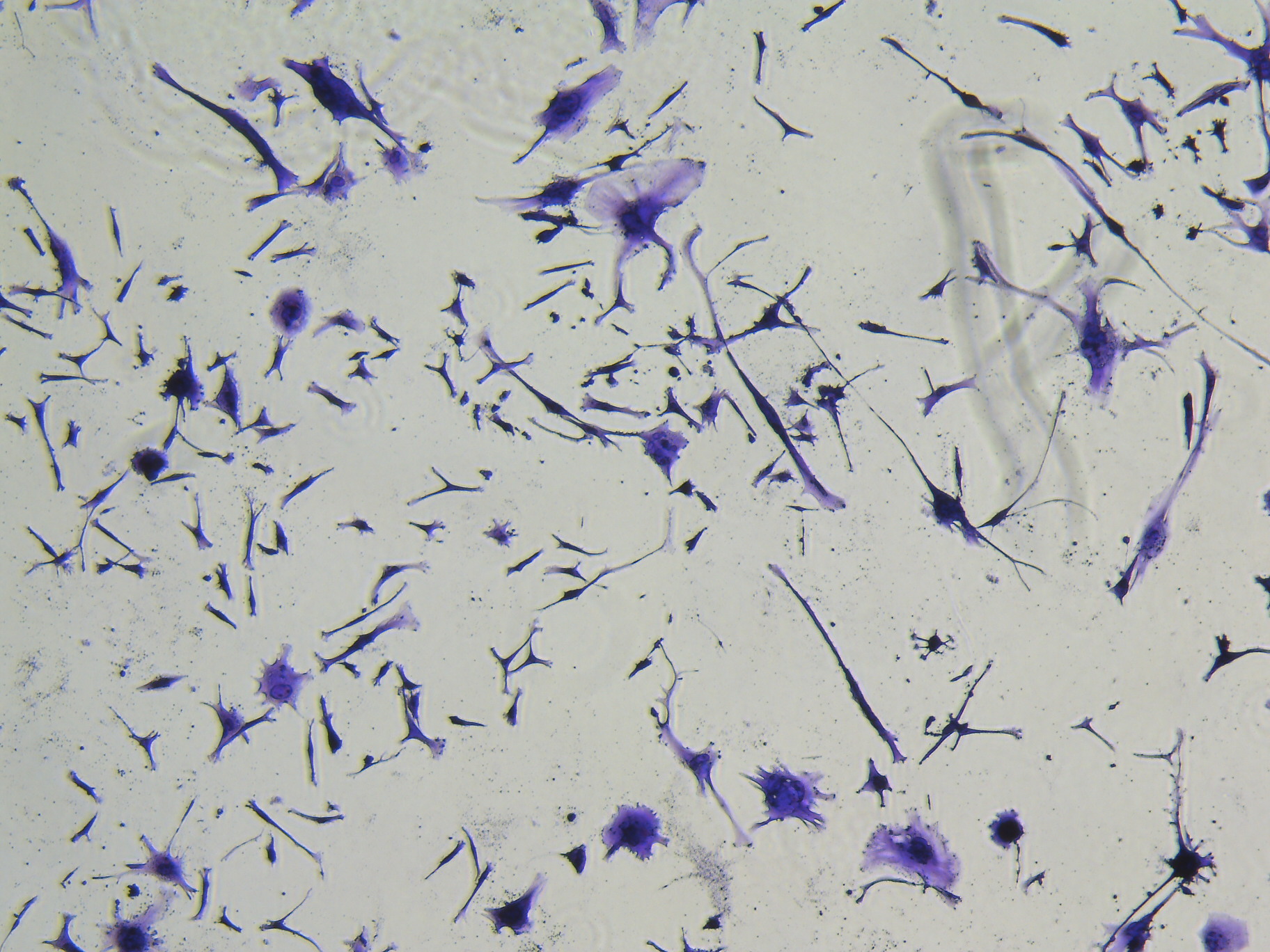

Supplement: Supplementary file 10 — EV figures [file 44321_2025_201_MOESM10_ESM.zip › source data for EV/EV4/EV4b CV/U118/IR/LCL D6.JPG]

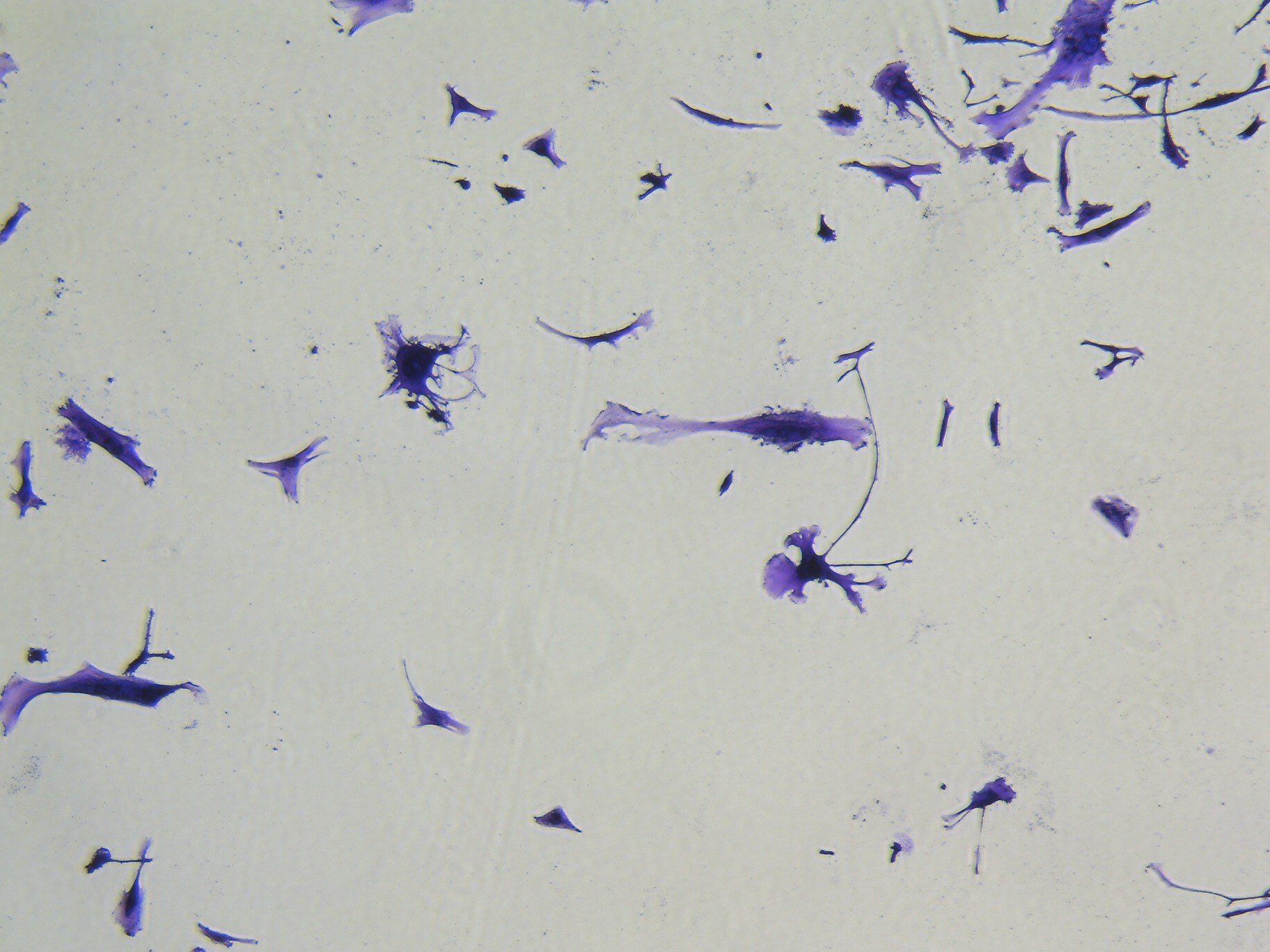

Supplement: Supplementary file 10 — EV figures [file 44321_2025_201_MOESM10_ESM.zip › source data for EV/EV4/EV4b CV/U118/IR/LCL D9.JPG]

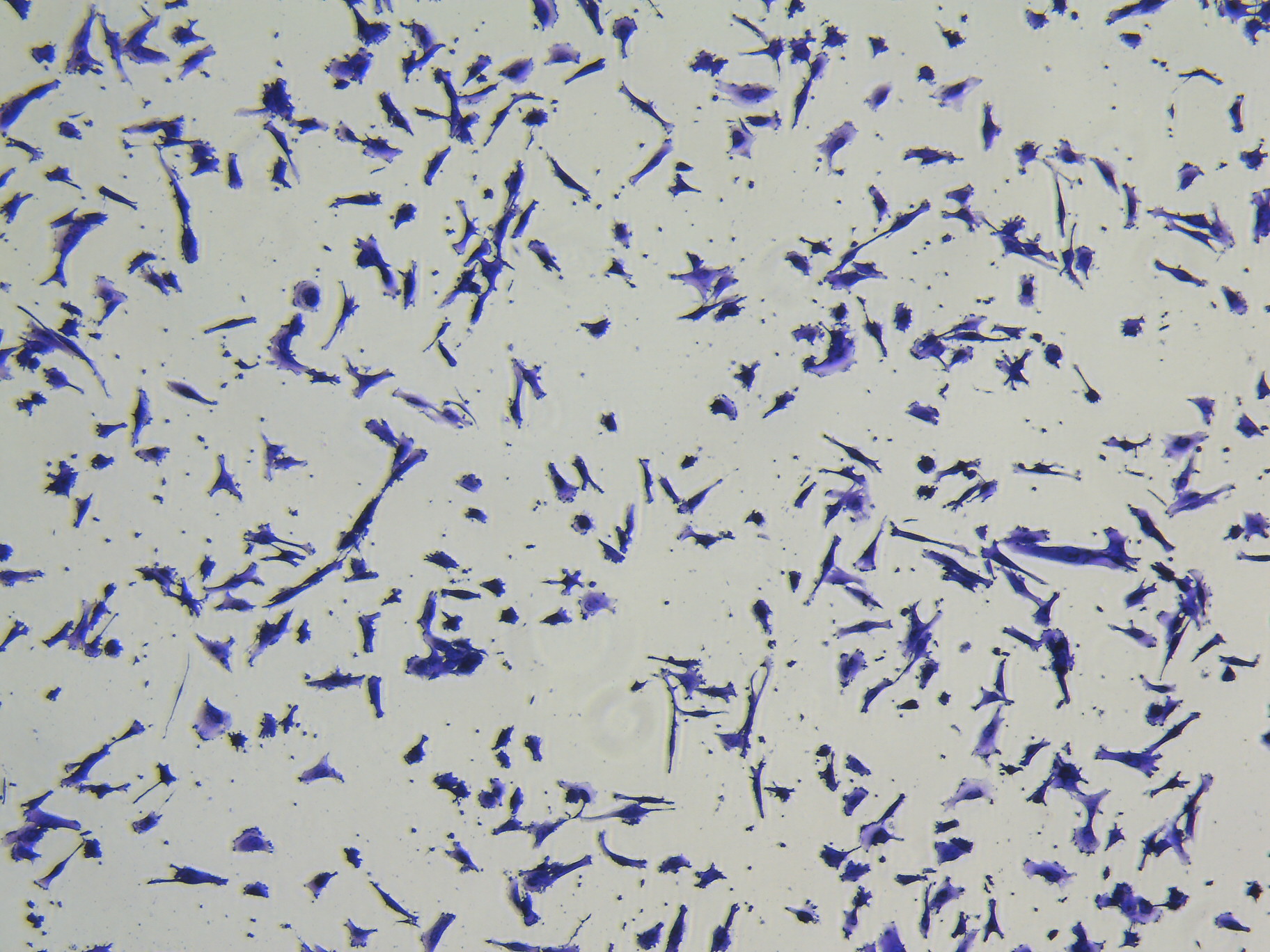

Supplement: Supplementary file 10 — EV figures [file 44321_2025_201_MOESM10_ESM.zip › source data for EV/EV4/EV4b CV/U118/mock/DMSO D0.JPG]

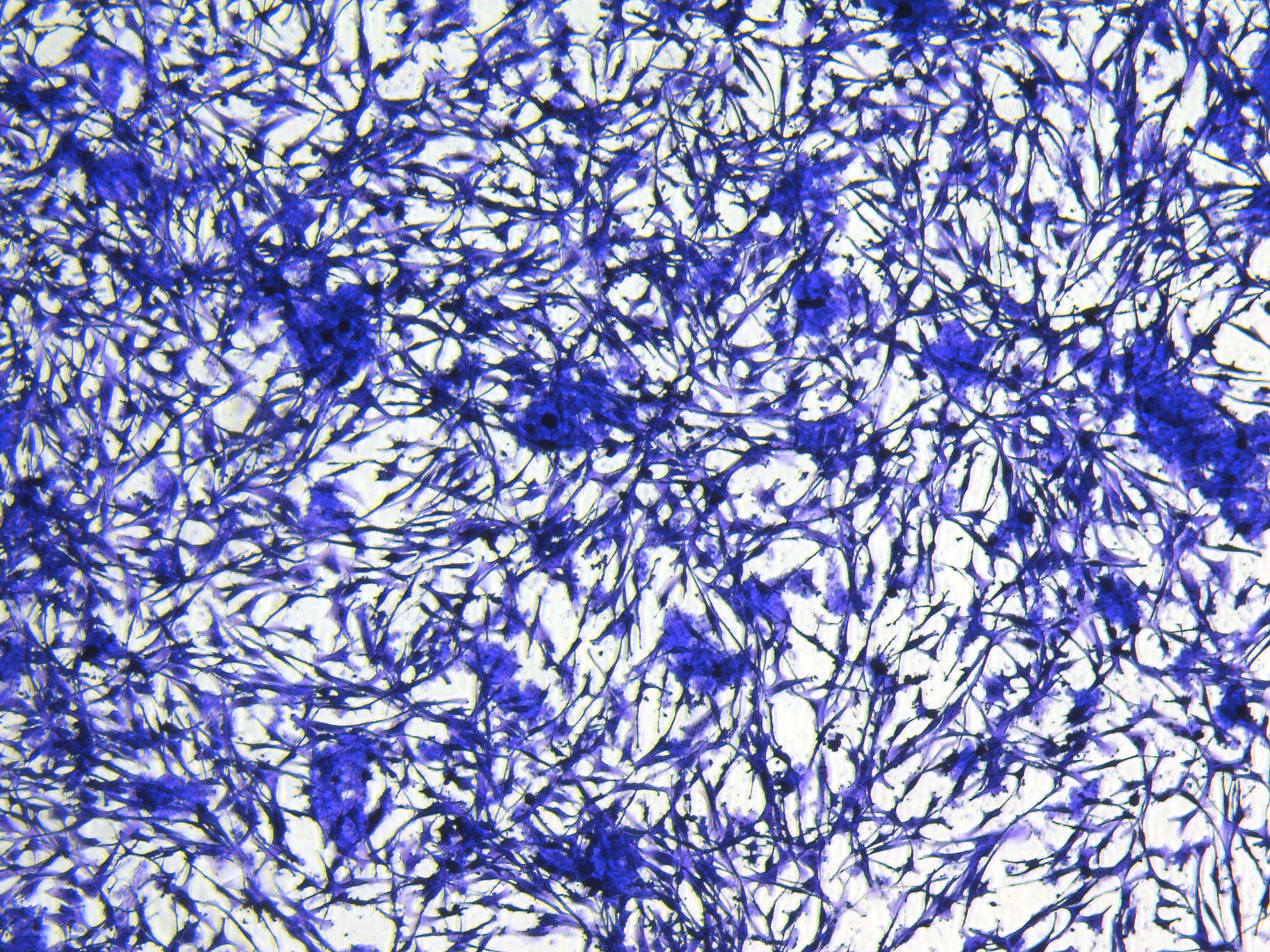

Supplement: Supplementary file 10 — EV figures [file 44321_2025_201_MOESM10_ESM.zip › source data for EV/EV4/EV4b CV/U118/mock/DMSO D3.JPG]

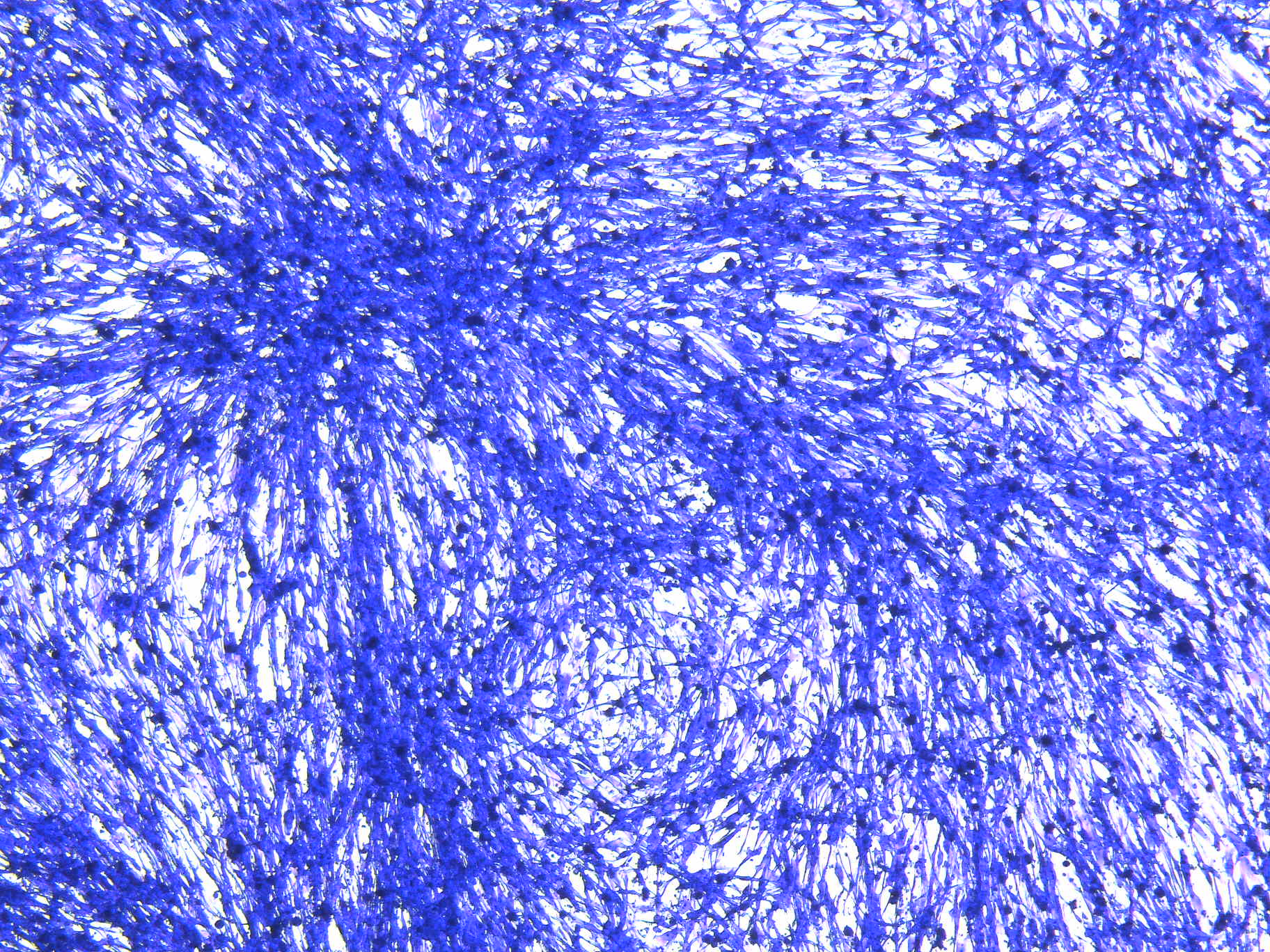

Supplement: Supplementary file 10 — EV figures [file 44321_2025_201_MOESM10_ESM.zip › source data for EV/EV4/EV4b CV/U118/mock/DMSO D6.JPG]

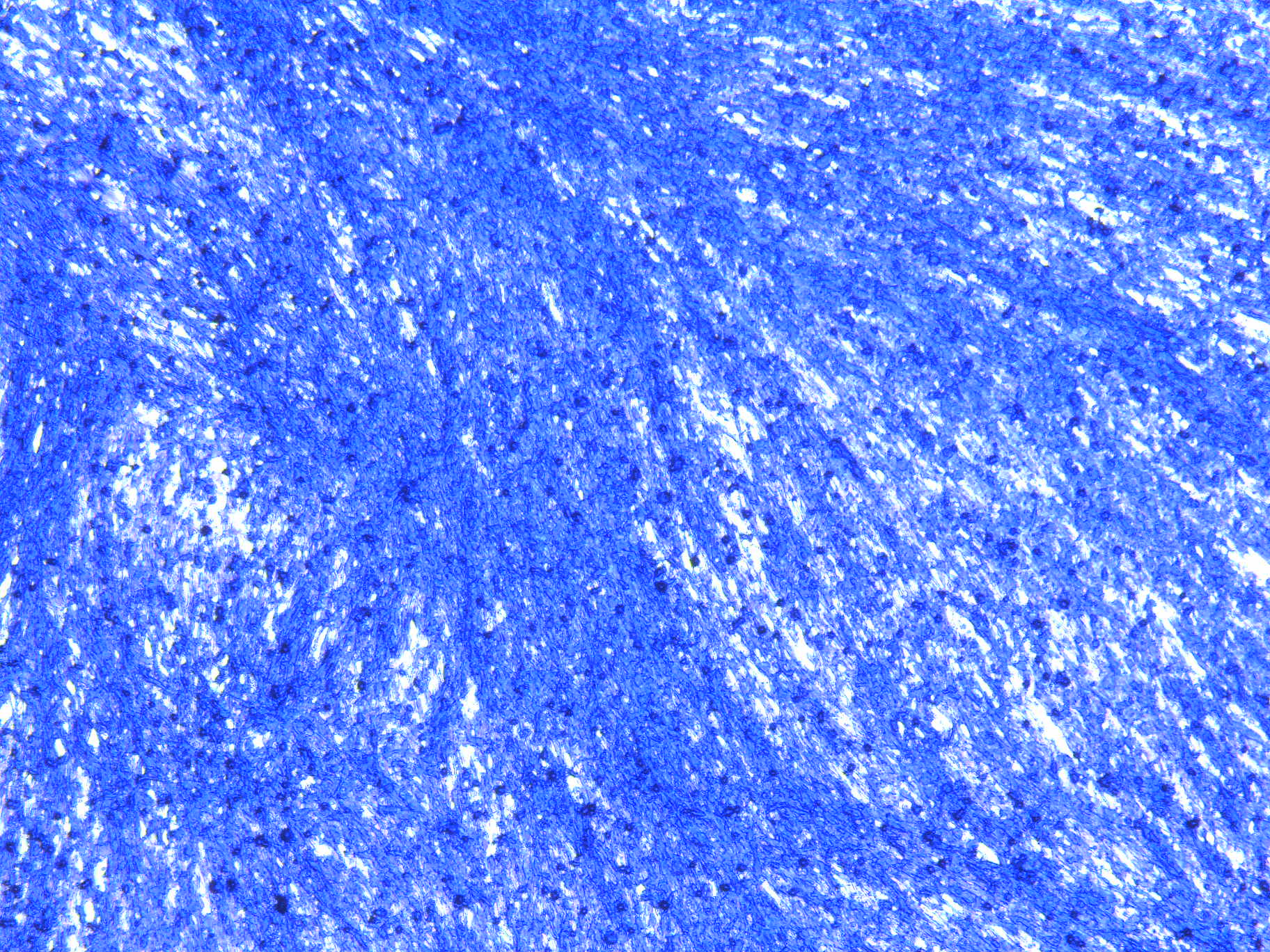

Supplement: Supplementary file 10 — EV figures [file 44321_2025_201_MOESM10_ESM.zip › source data for EV/EV4/EV4b CV/U118/mock/DMSO D9.JPG]

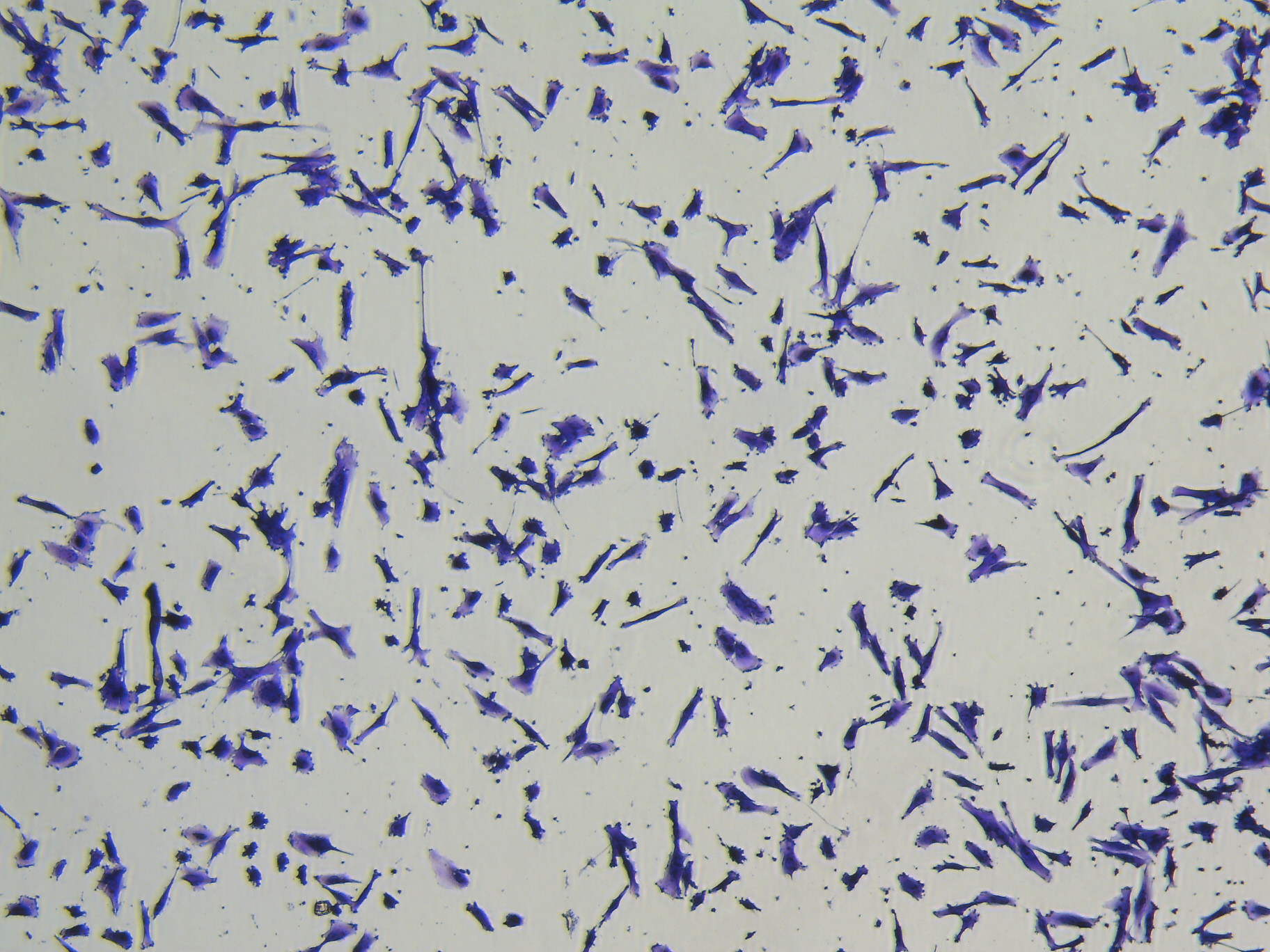

Supplement: Supplementary file 10 — EV figures [file 44321_2025_201_MOESM10_ESM.zip › source data for EV/EV4/EV4b CV/U118/mock/LCL D0.JPG]

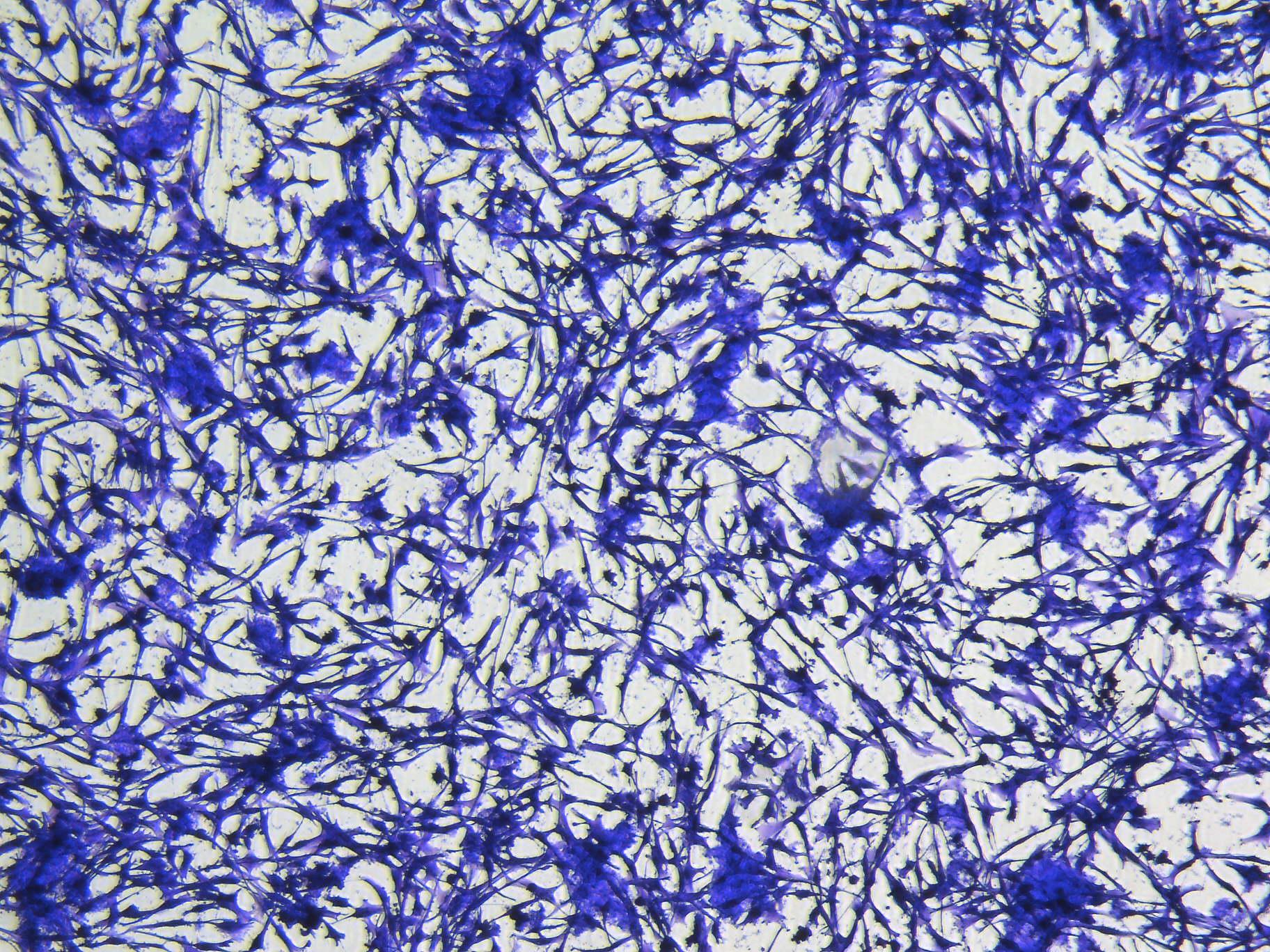

Supplement: Supplementary file 10 — EV figures [file 44321_2025_201_MOESM10_ESM.zip › source data for EV/EV4/EV4b CV/U118/mock/LCL D3.JPG]

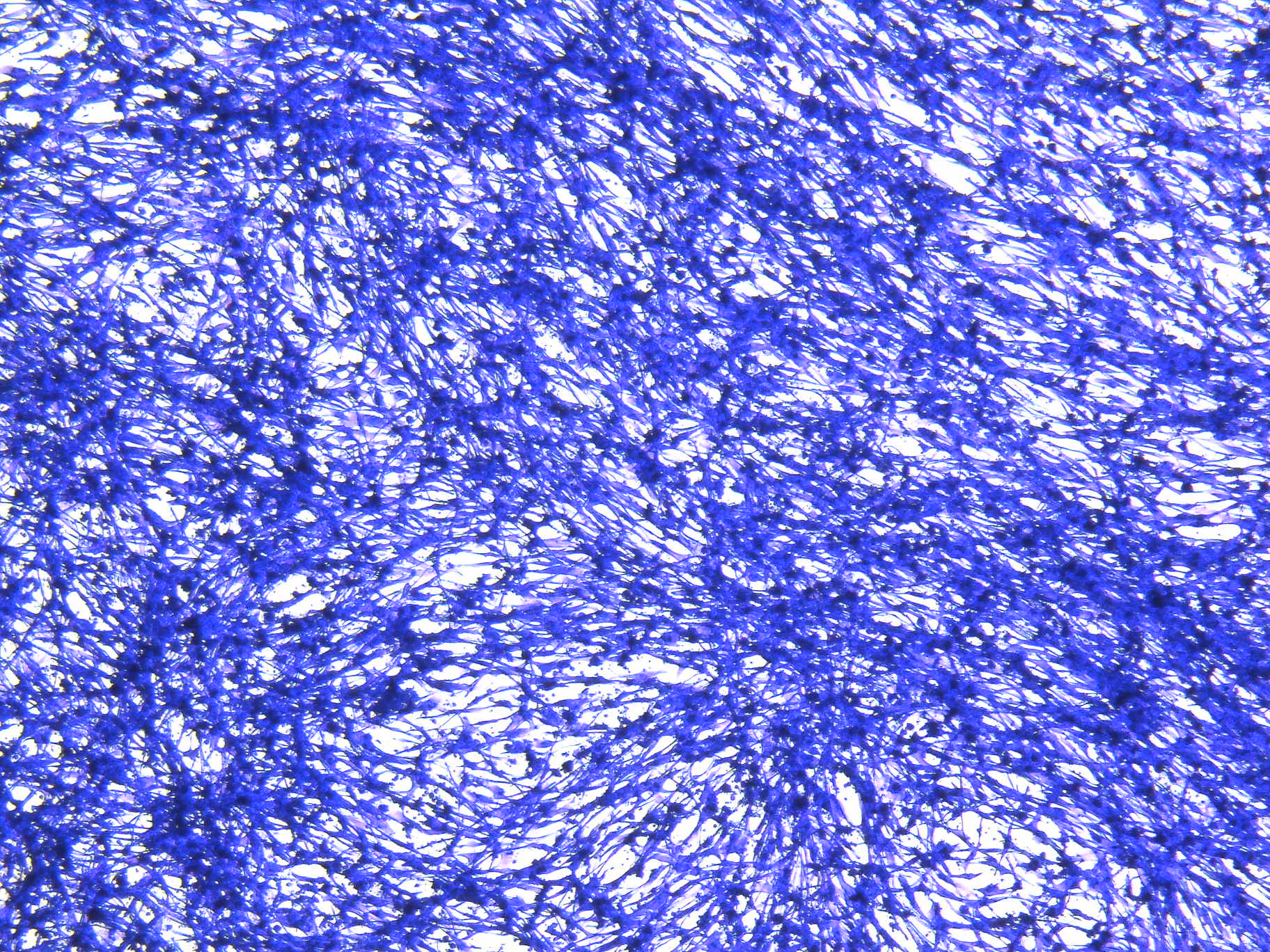

Supplement: Supplementary file 10 — EV figures [file 44321_2025_201_MOESM10_ESM.zip › source data for EV/EV4/EV4b CV/U118/mock/LCL D6.JPG]

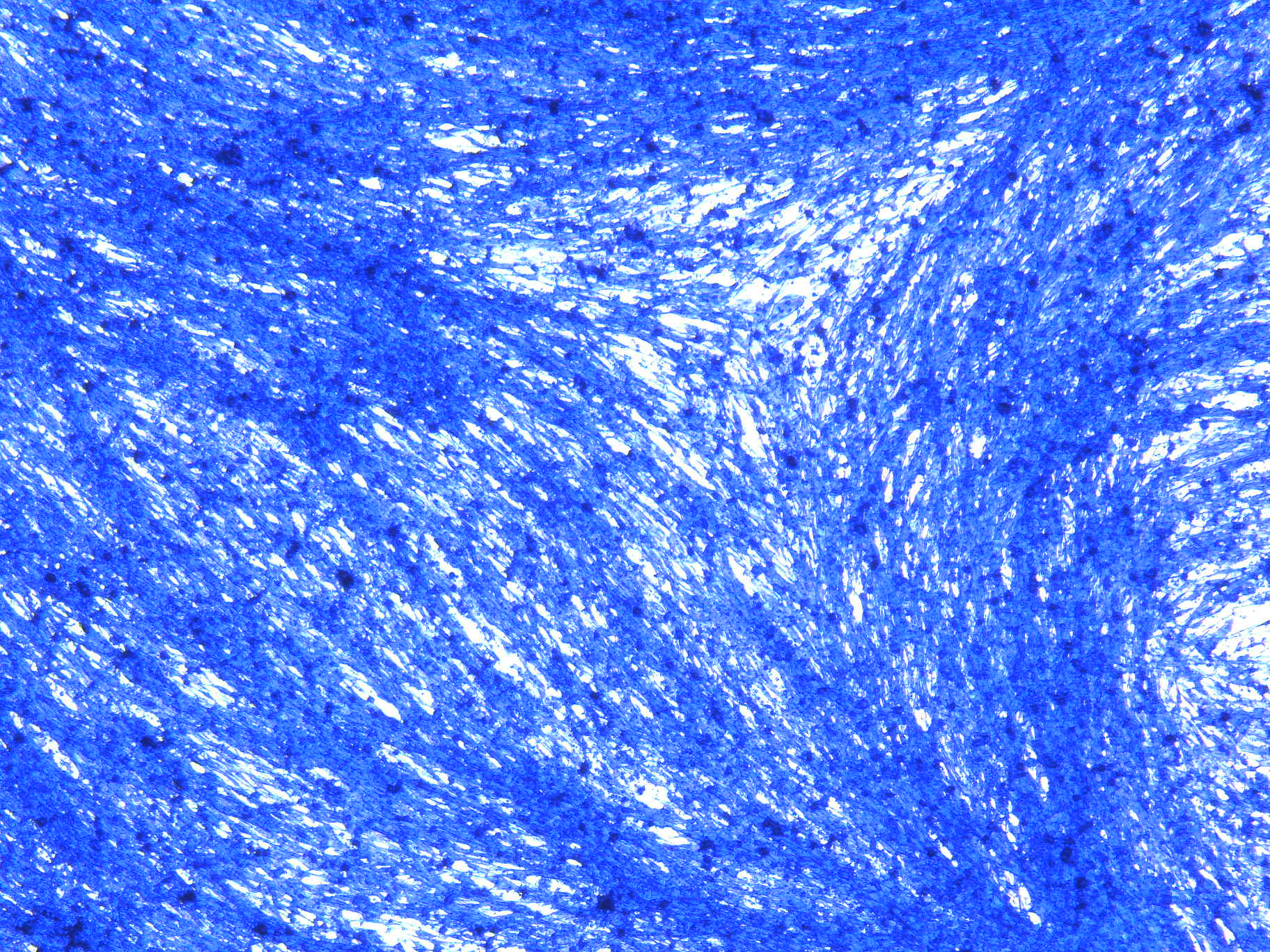

Supplement: Supplementary file 10 — EV figures [file 44321_2025_201_MOESM10_ESM.zip › source data for EV/EV4/EV4b CV/U118/mock/LCL D9.JPG]

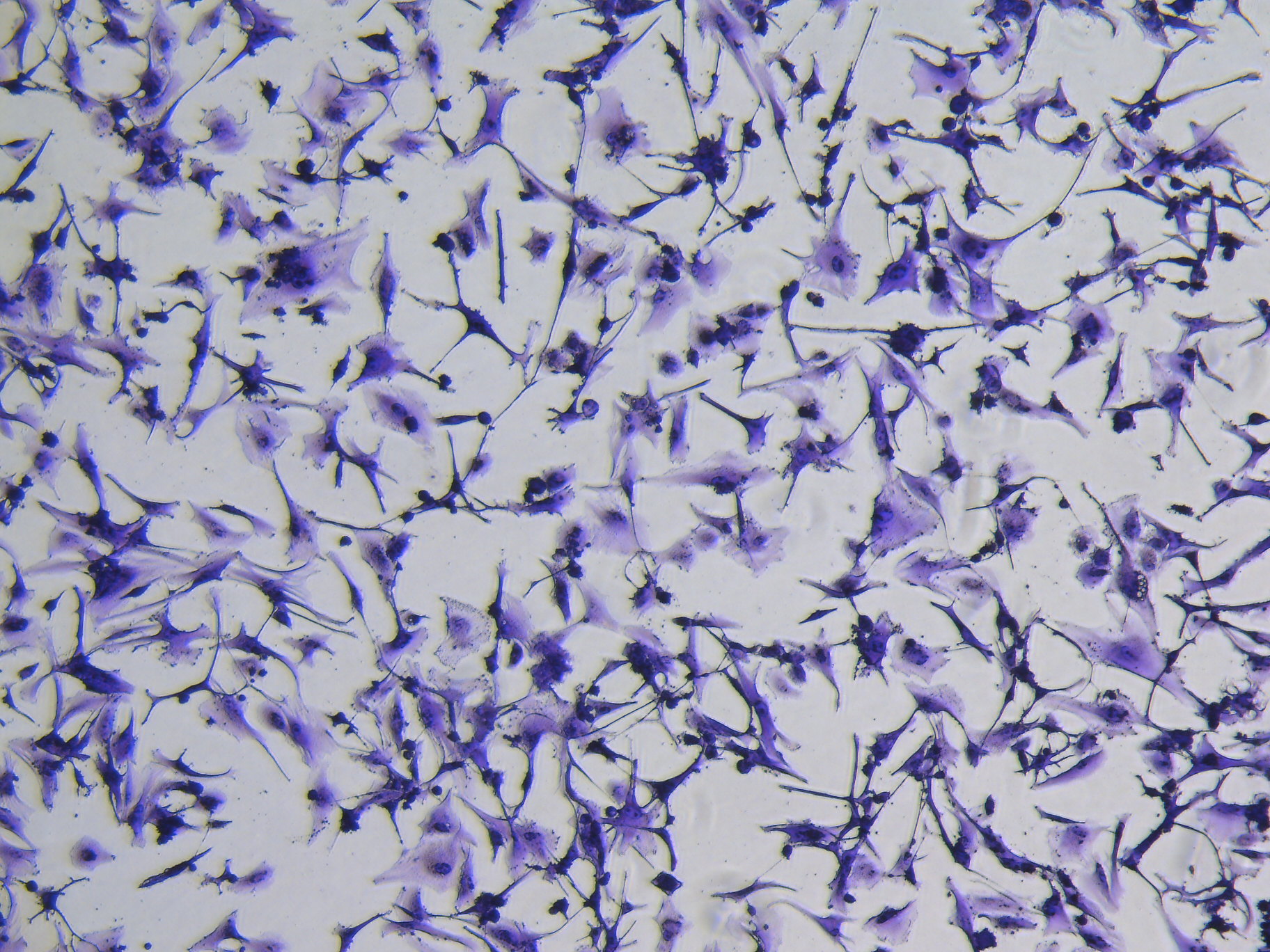

Supplement: Supplementary file 10 — EV figures [file 44321_2025_201_MOESM10_ESM.zip › source data for EV/EV4/EV4b CV/U87/IR/DMSO D0.JPG]

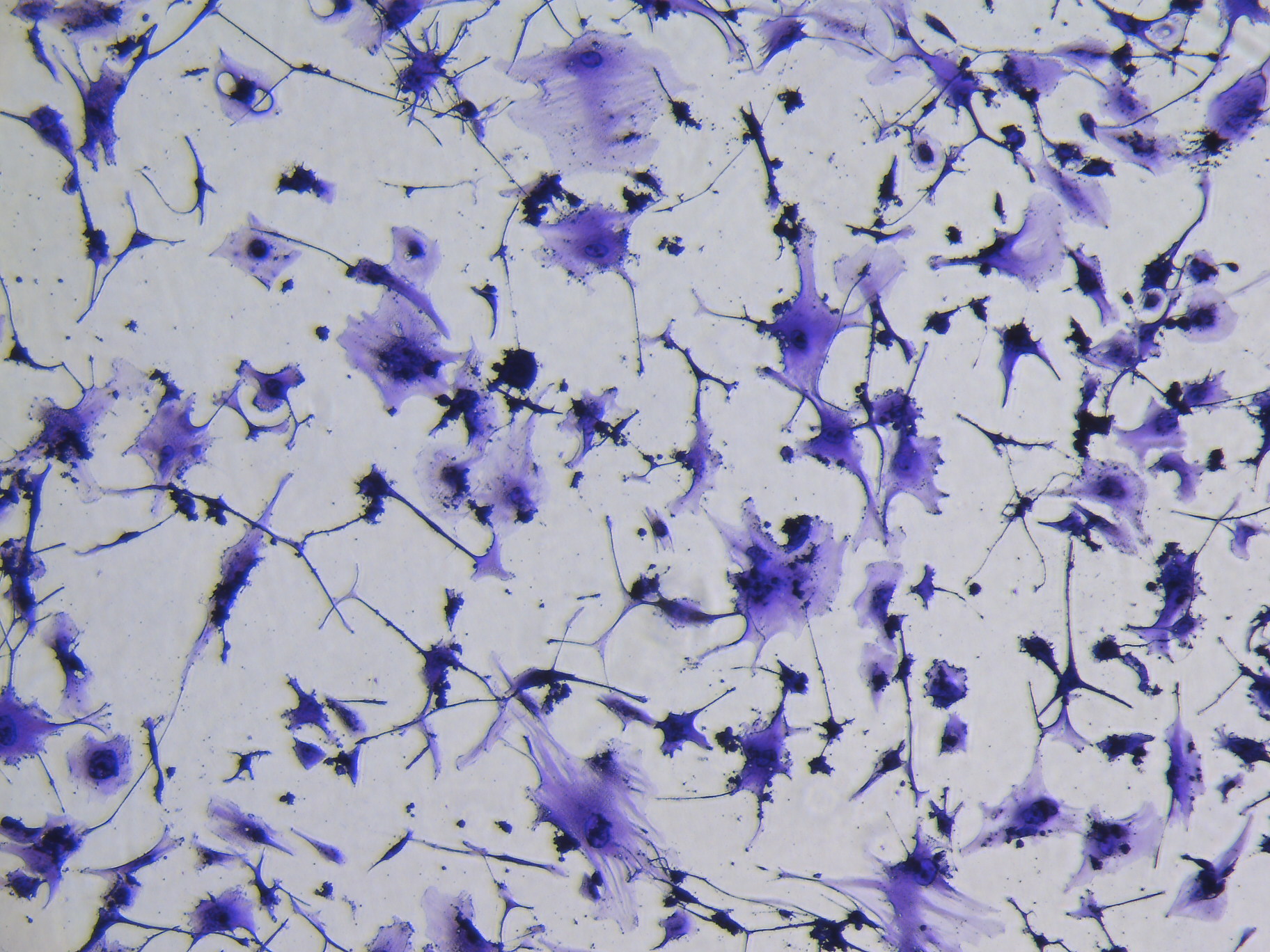

Supplement: Supplementary file 10 — EV figures [file 44321_2025_201_MOESM10_ESM.zip › source data for EV/EV4/EV4b CV/U87/IR/DMSO D3.JPG]

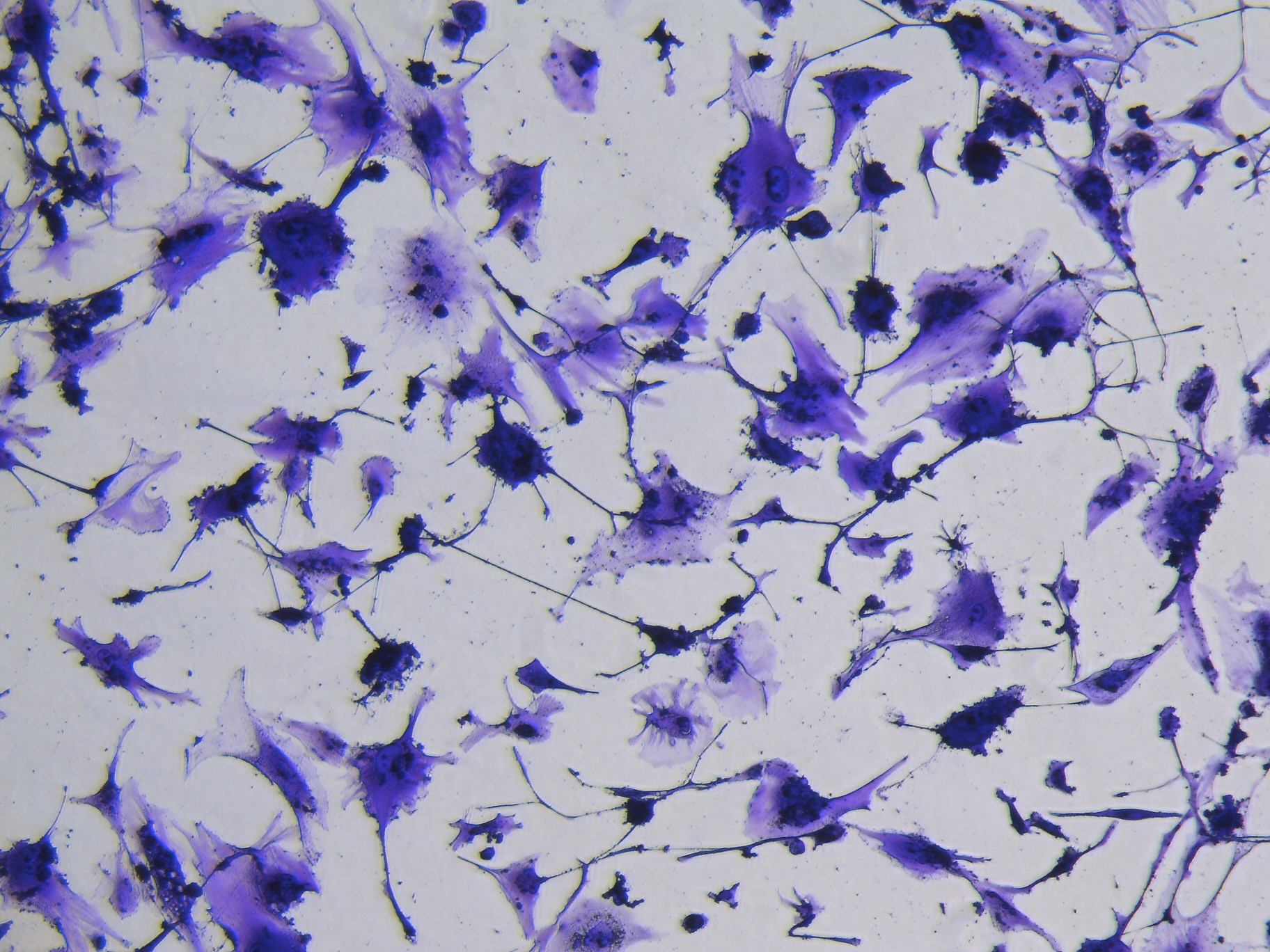

Supplement: Supplementary file 10 — EV figures [file 44321_2025_201_MOESM10_ESM.zip › source data for EV/EV4/EV4b CV/U87/IR/DMSO D6.JPG]

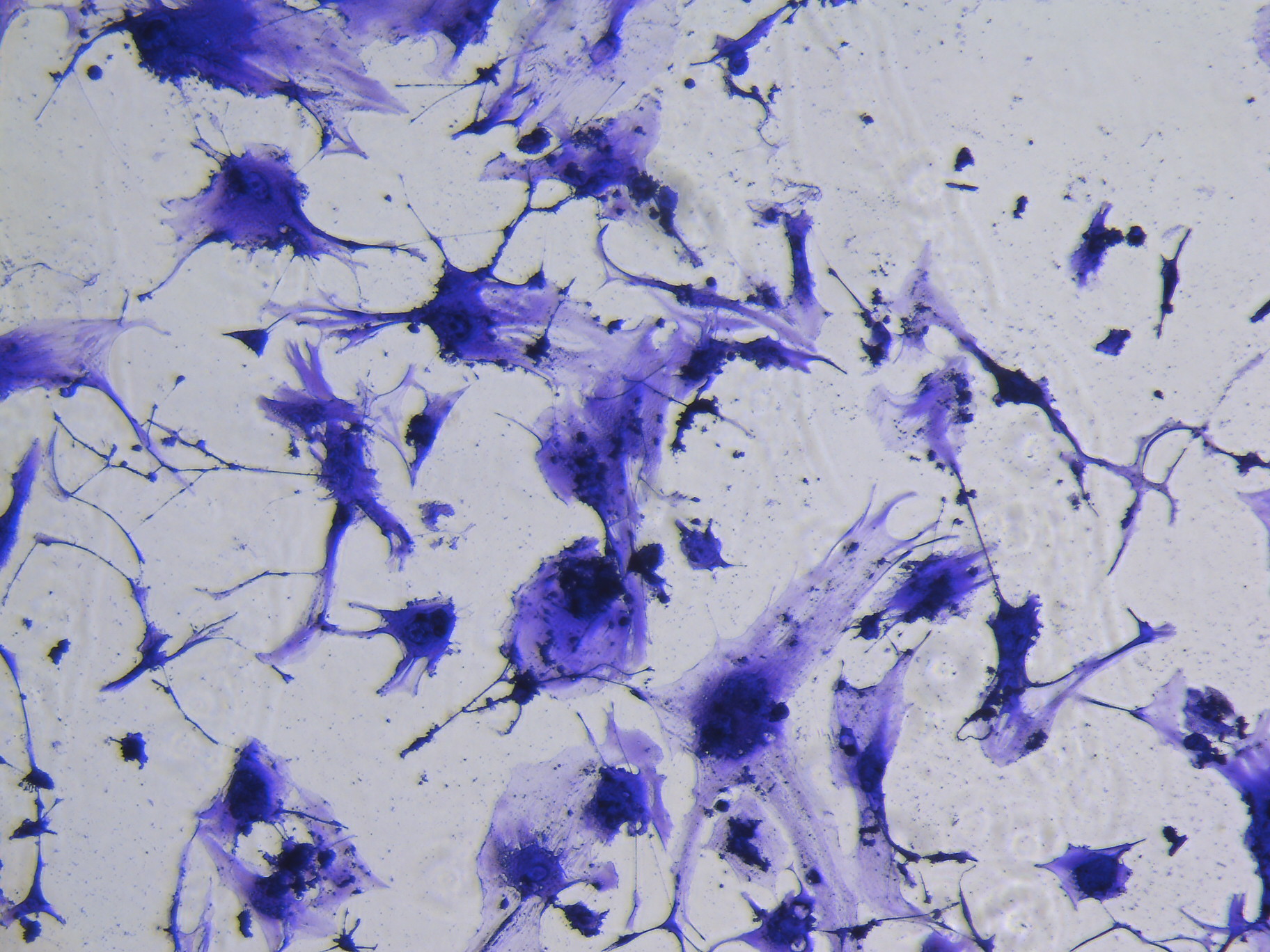

Supplement: Supplementary file 10 — EV figures [file 44321_2025_201_MOESM10_ESM.zip › source data for EV/EV4/EV4b CV/U87/IR/DMSO D9.JPG]

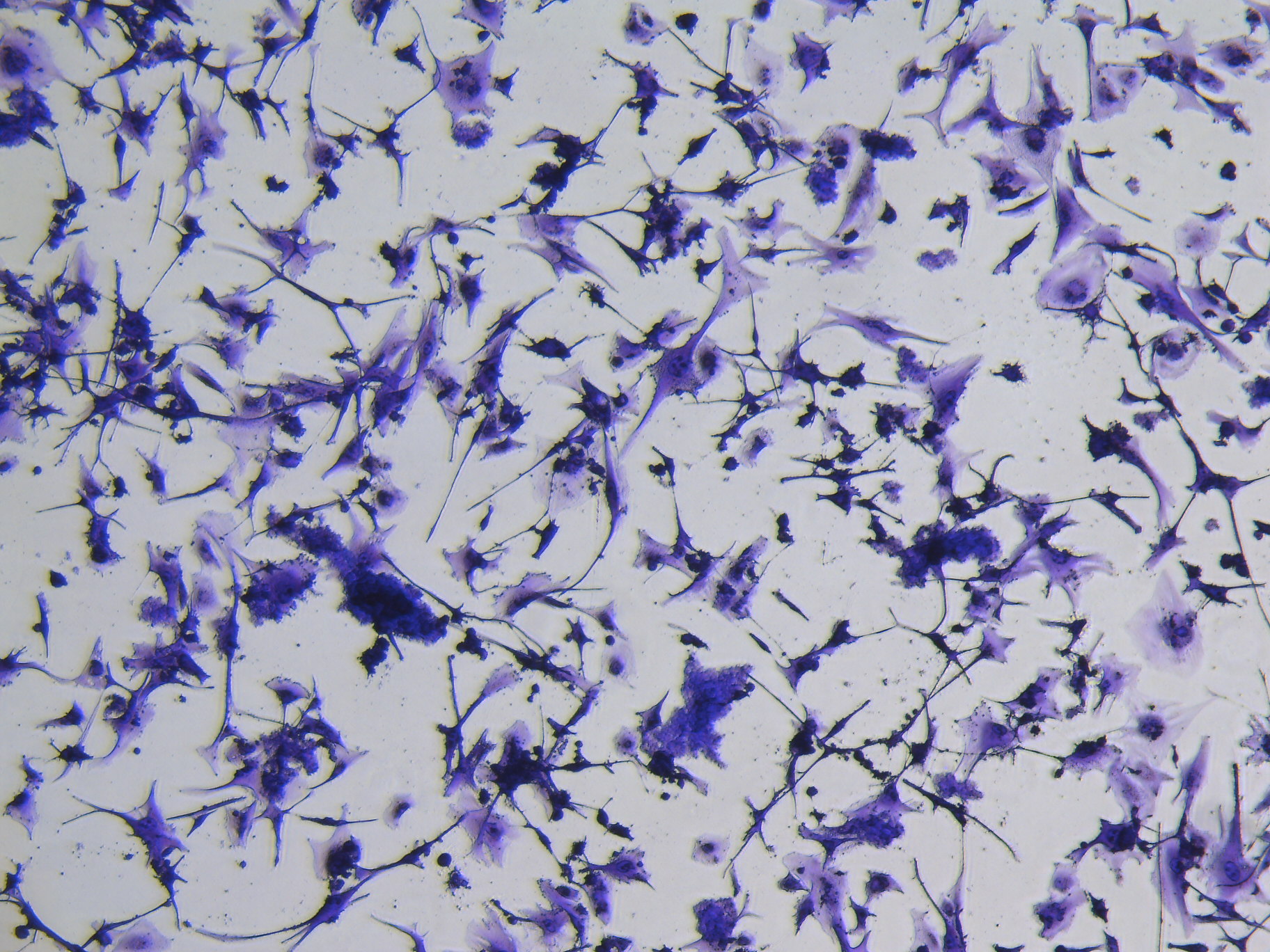

Supplement: Supplementary file 10 — EV figures [file 44321_2025_201_MOESM10_ESM.zip › source data for EV/EV4/EV4b CV/U87/IR/LCL D0.JPG]

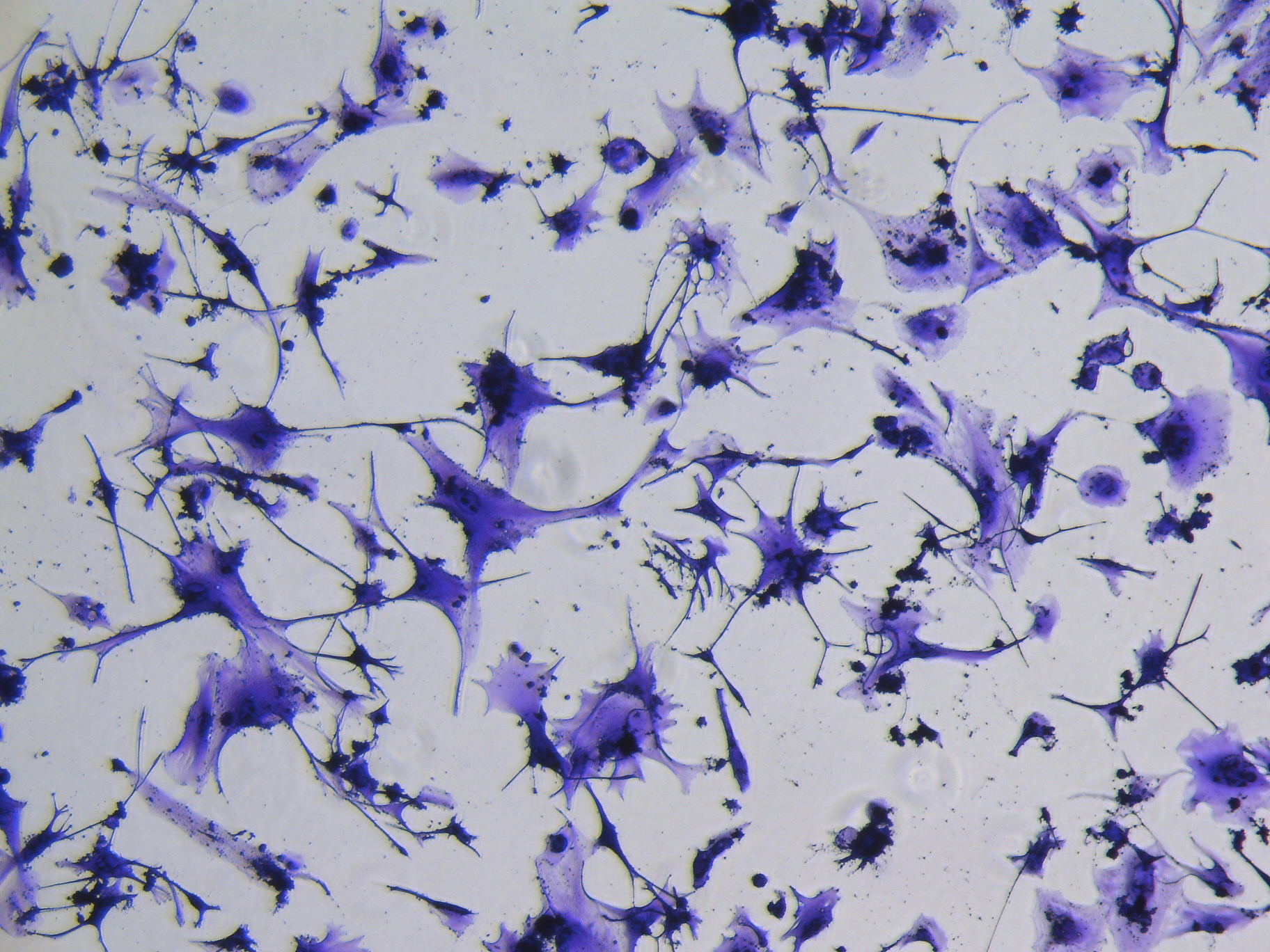

Supplement: Supplementary file 10 — EV figures [file 44321_2025_201_MOESM10_ESM.zip › source data for EV/EV4/EV4b CV/U87/IR/LCL D3.JPG]

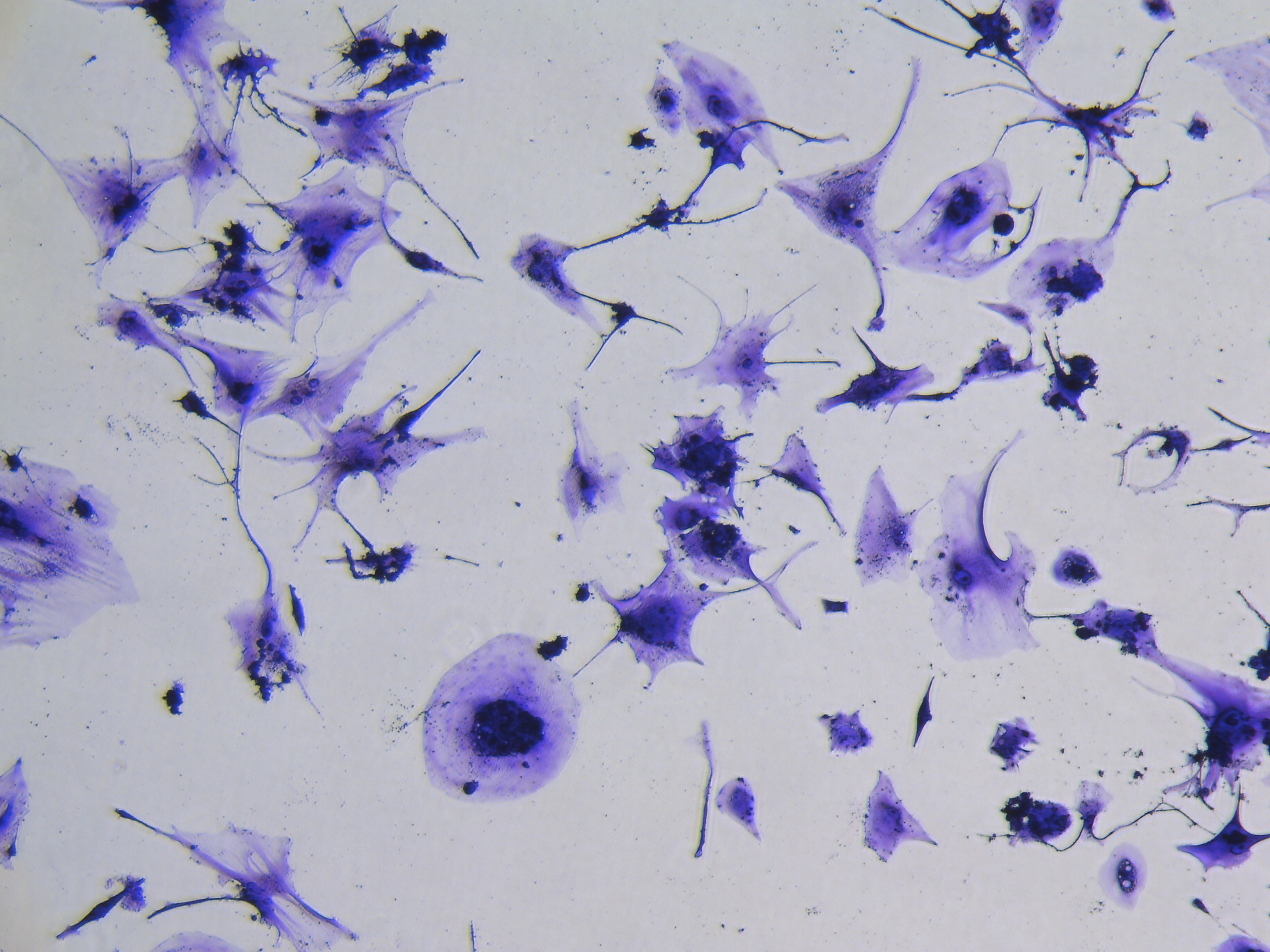

Supplement: Supplementary file 10 — EV figures [file 44321_2025_201_MOESM10_ESM.zip › source data for EV/EV4/EV4b CV/U87/IR/LCL D6.JPG]

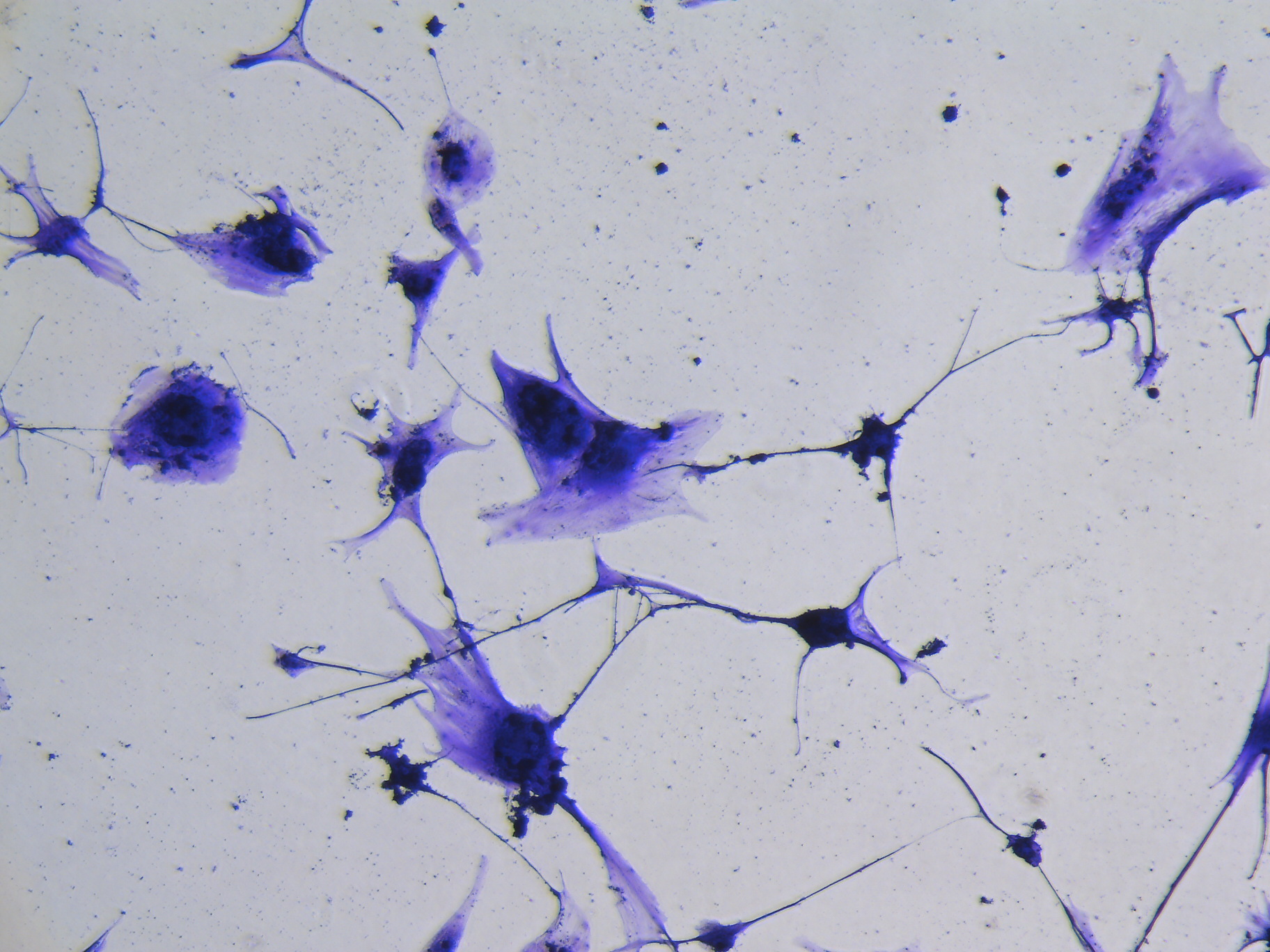

Supplement: Supplementary file 10 — EV figures [file 44321_2025_201_MOESM10_ESM.zip › source data for EV/EV4/EV4b CV/U87/IR/LCL D9.JPG]

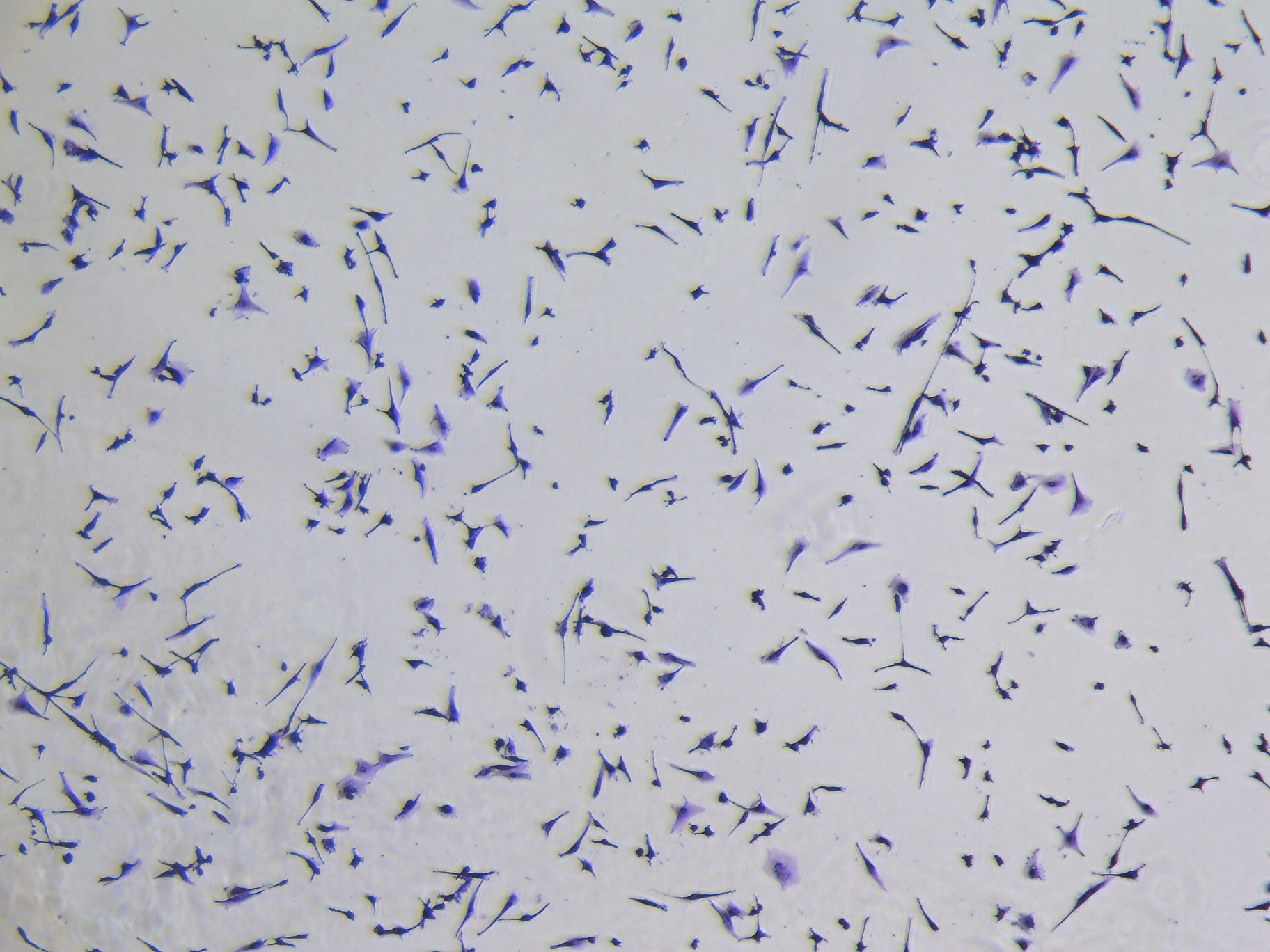

Supplement: Supplementary file 10 — EV figures [file 44321_2025_201_MOESM10_ESM.zip › source data for EV/EV4/EV4b CV/U87/mock/DMSO D0.JPG]

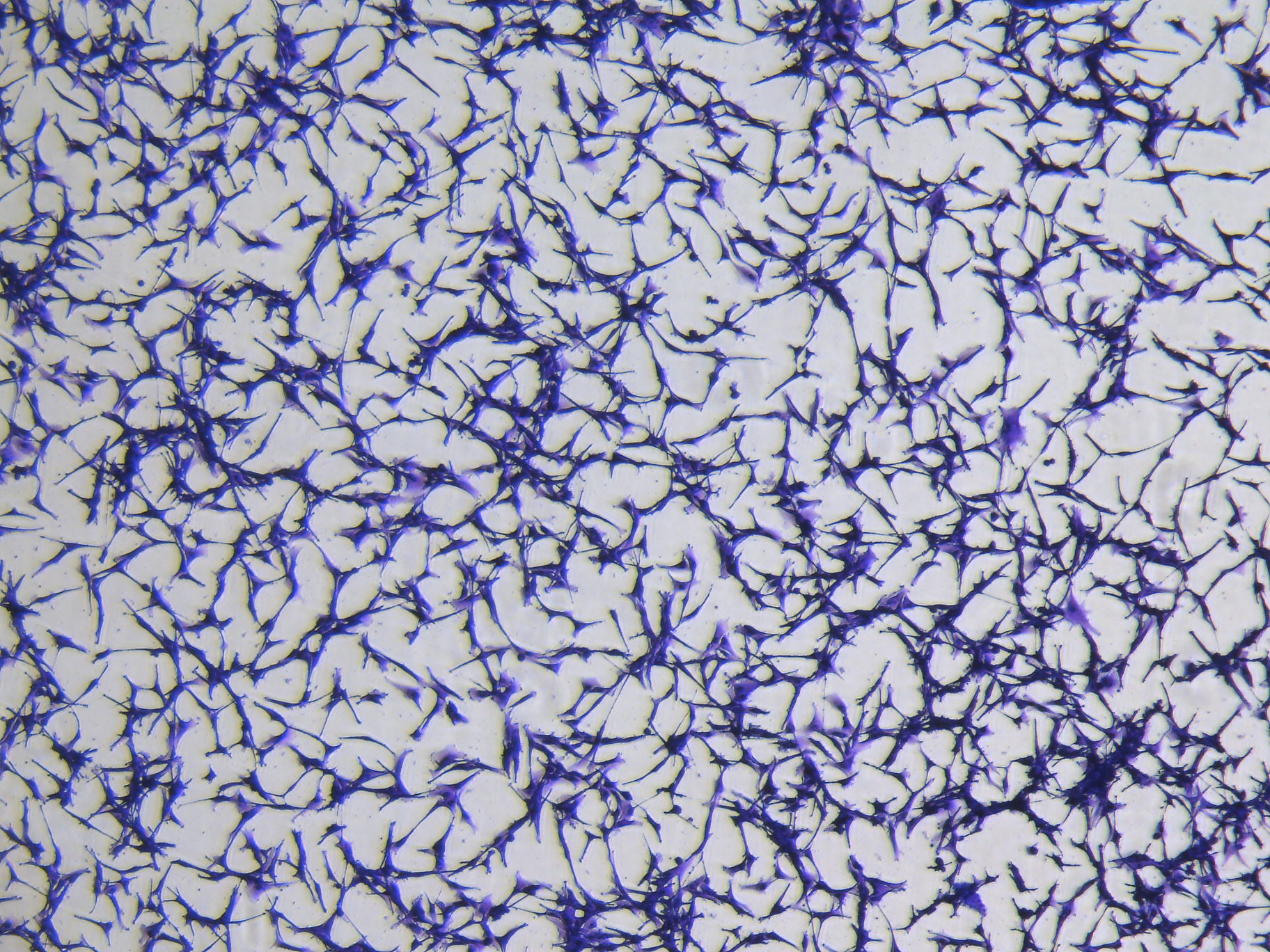

Supplement: Supplementary file 10 — EV figures [file 44321_2025_201_MOESM10_ESM.zip › source data for EV/EV4/EV4b CV/U87/mock/DMSO D3.JPG]

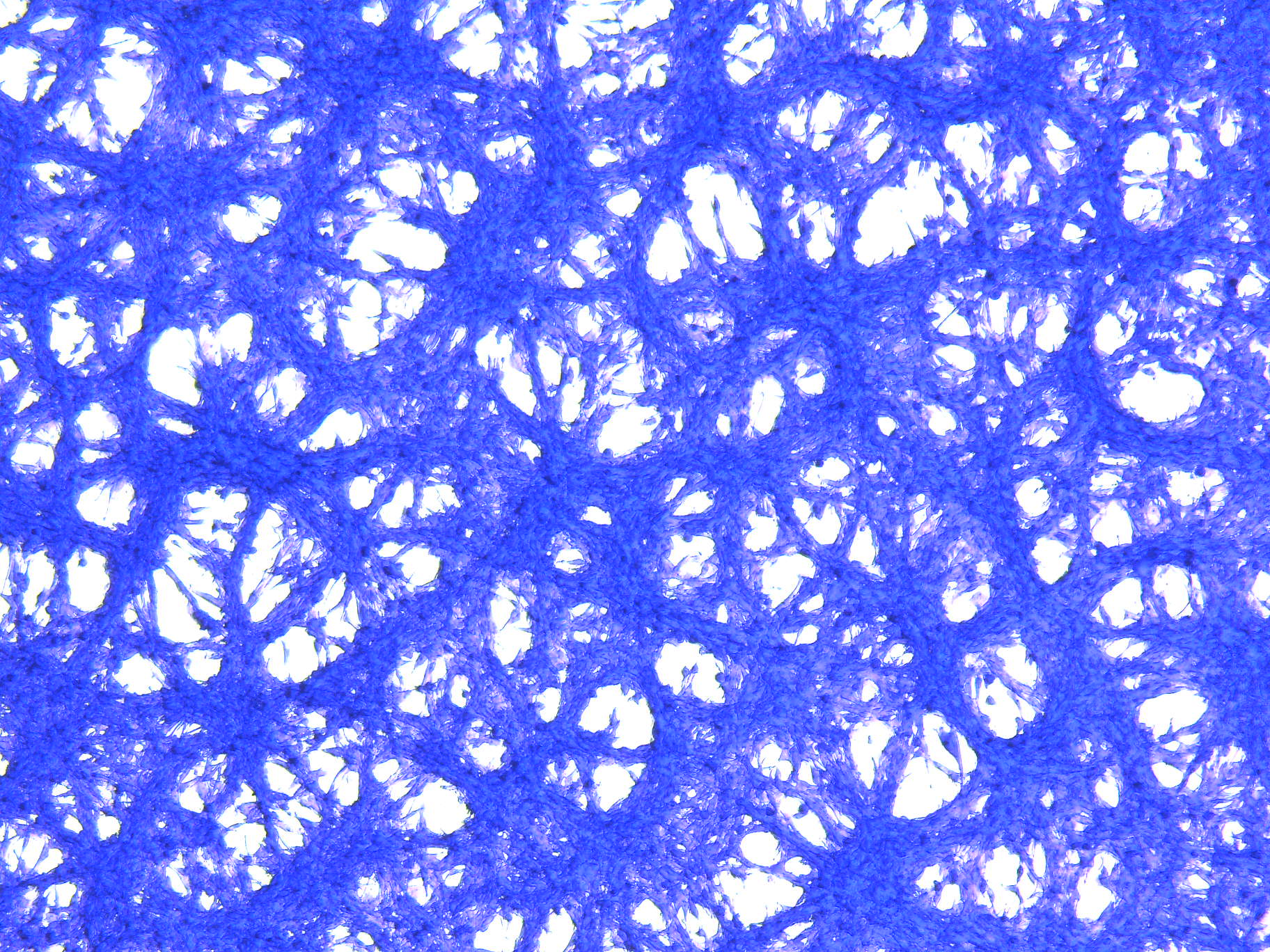

Supplement: Supplementary file 10 — EV figures [file 44321_2025_201_MOESM10_ESM.zip › source data for EV/EV4/EV4b CV/U87/mock/DMSO D6.JPG]

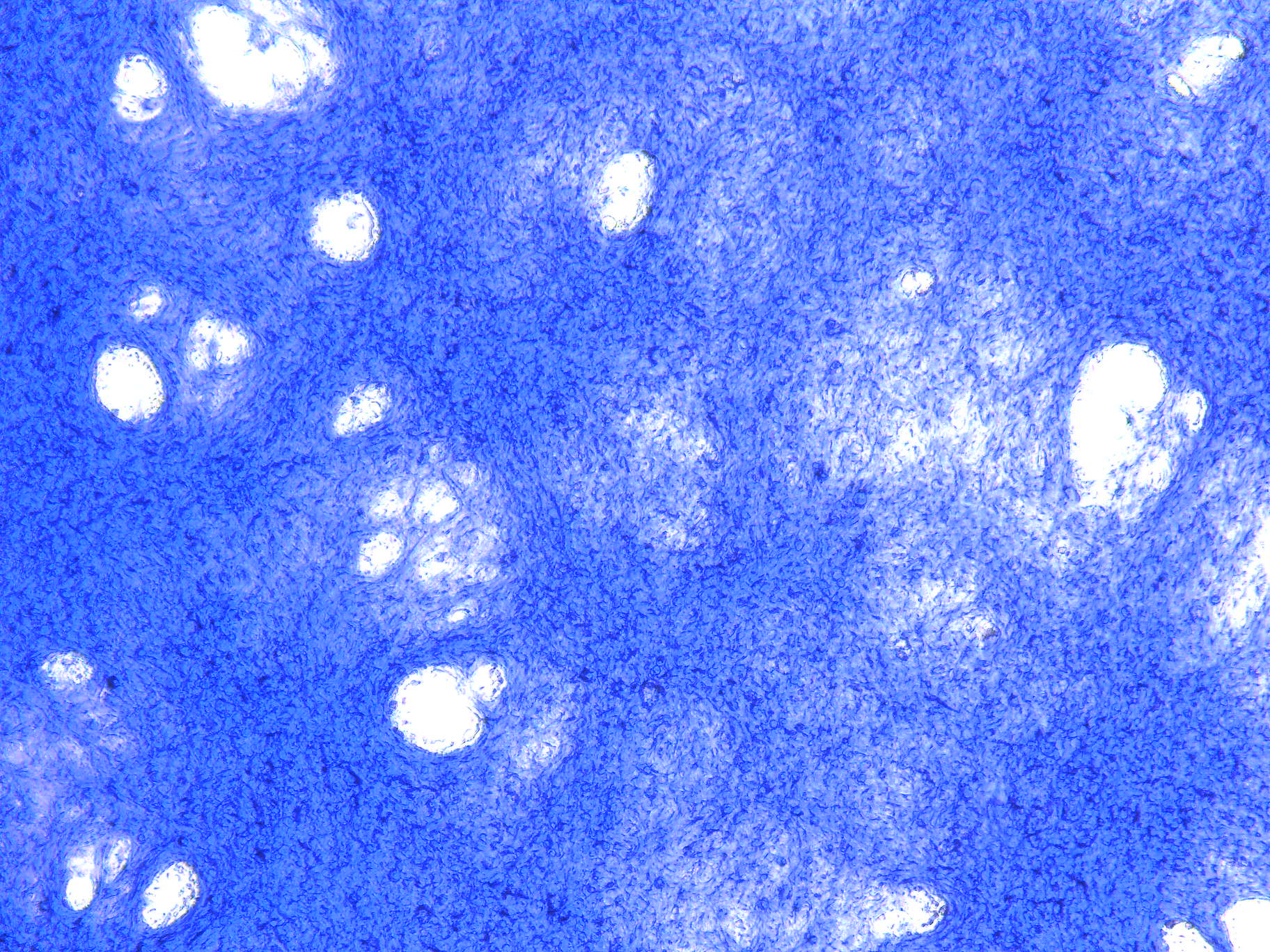

Supplement: Supplementary file 10 — EV figures [file 44321_2025_201_MOESM10_ESM.zip › source data for EV/EV4/EV4b CV/U87/mock/DMSO D9.JPG]

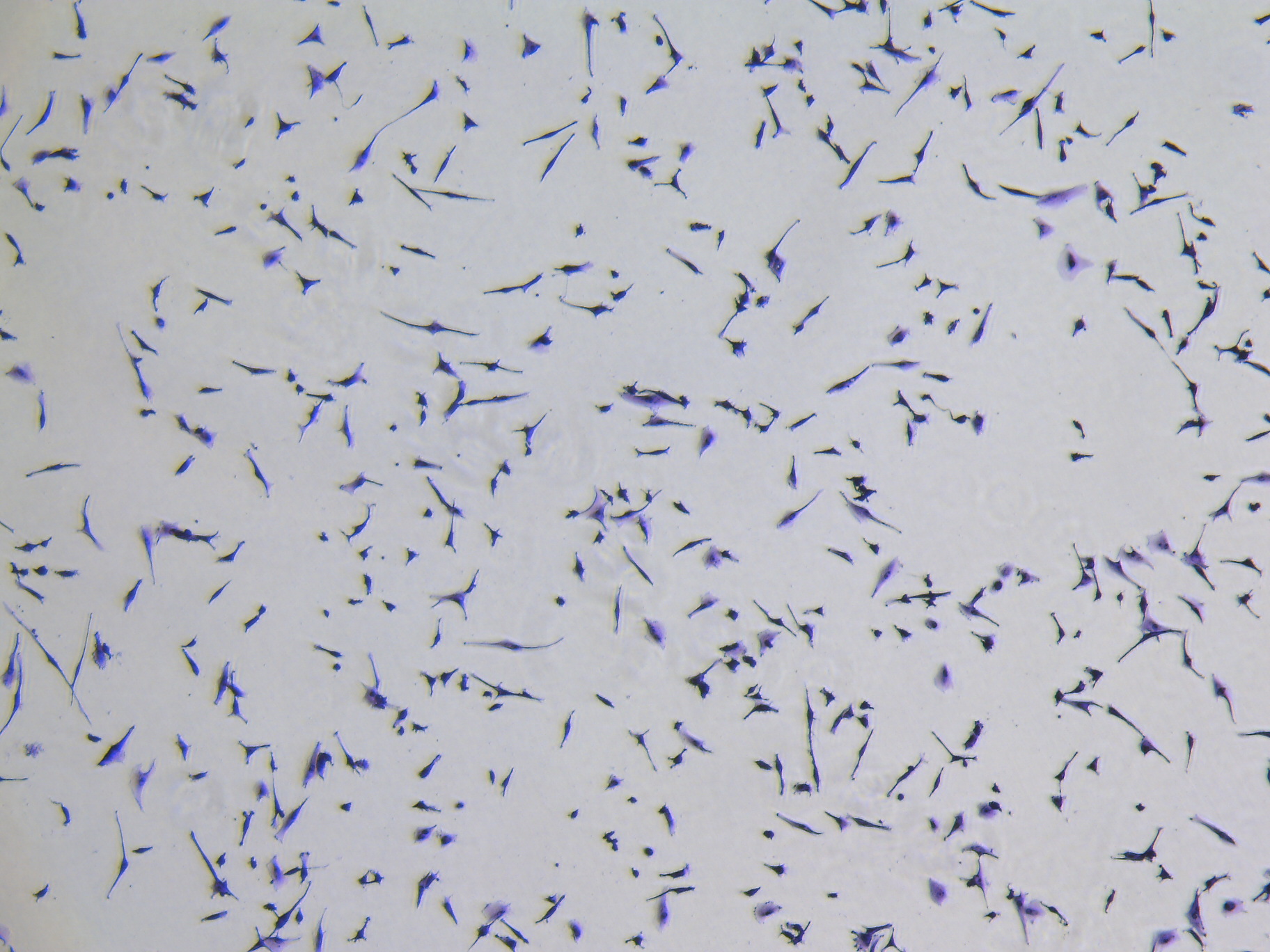

Supplement: Supplementary file 10 — EV figures [file 44321_2025_201_MOESM10_ESM.zip › source data for EV/EV4/EV4b CV/U87/mock/LCL D0.JPG]

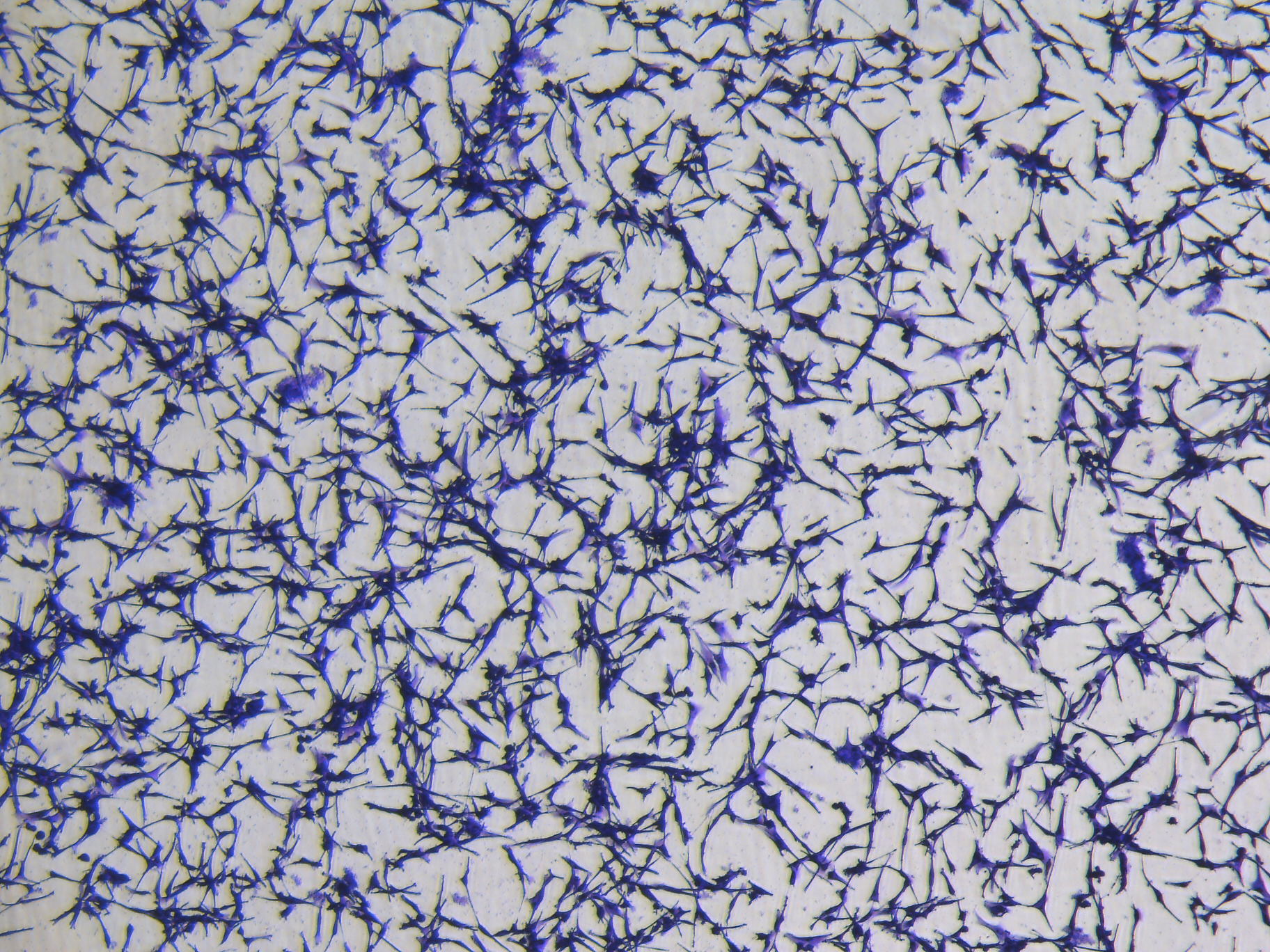

Supplement: Supplementary file 10 — EV figures [file 44321_2025_201_MOESM10_ESM.zip › source data for EV/EV4/EV4b CV/U87/mock/LCL D3.JPG]

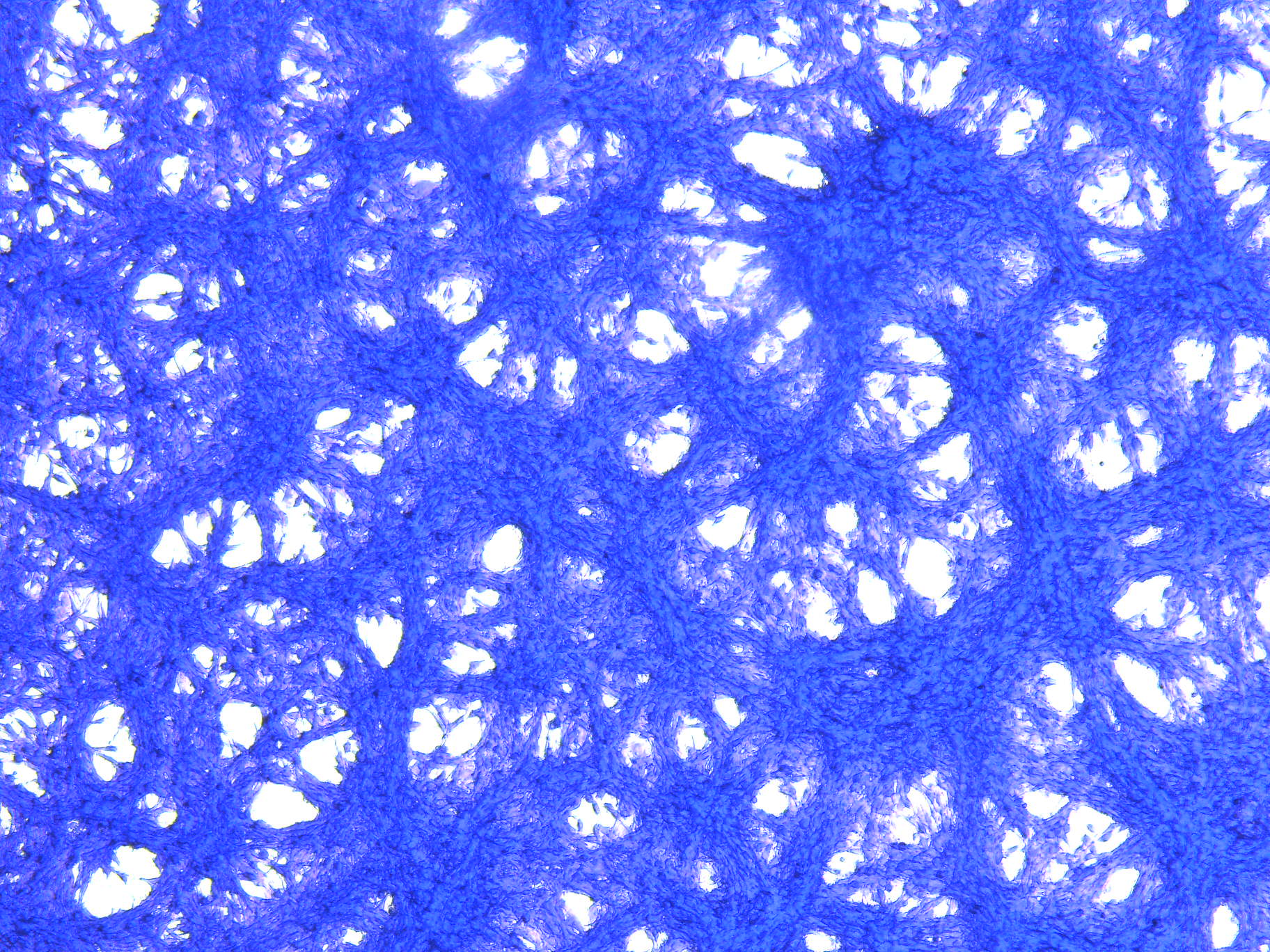

Supplement: Supplementary file 10 — EV figures [file 44321_2025_201_MOESM10_ESM.zip › source data for EV/EV4/EV4b CV/U87/mock/LCL D6.JPG]

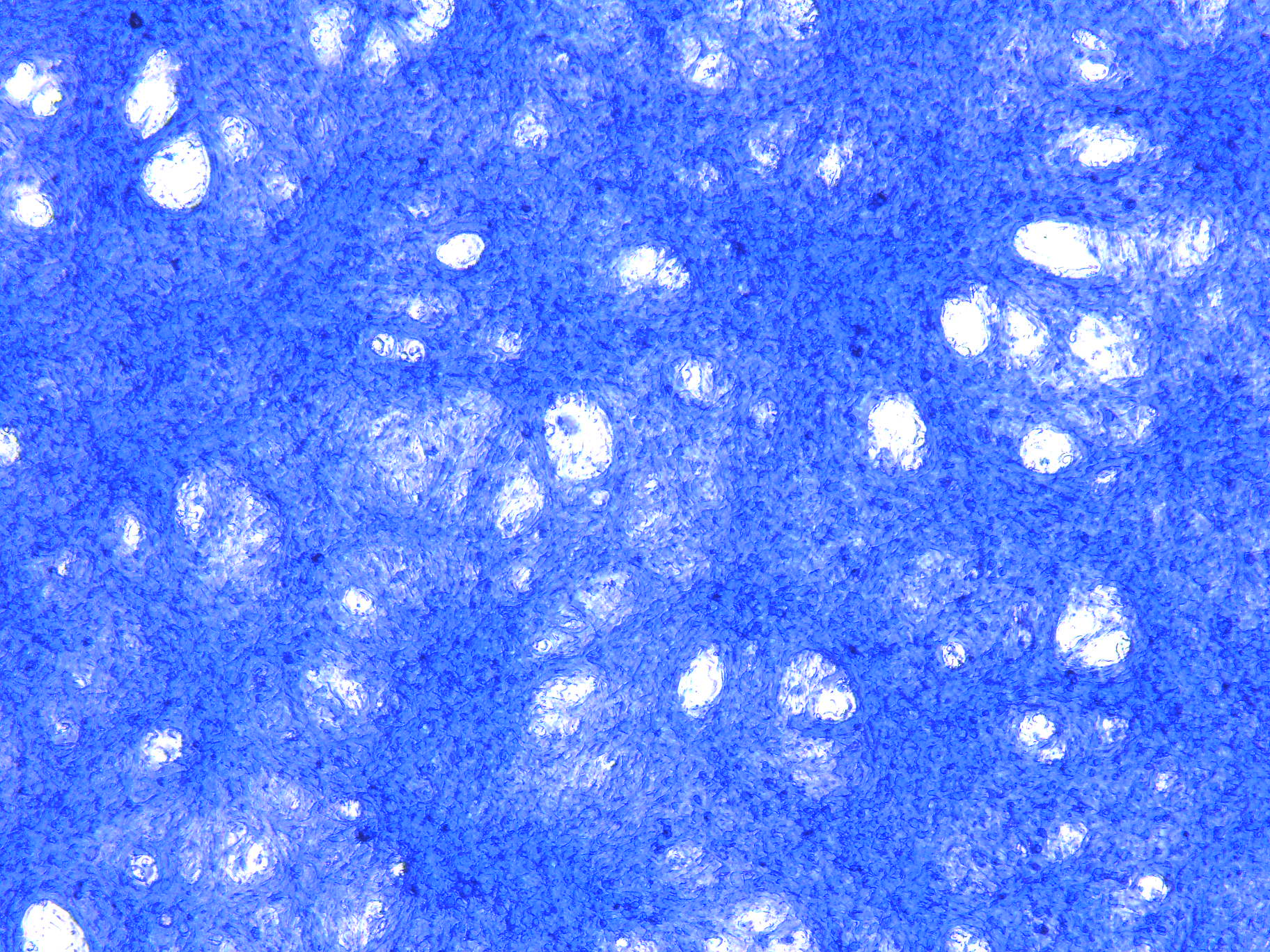

Supplement: Supplementary file 10 — EV figures [file 44321_2025_201_MOESM10_ESM.zip › source data for EV/EV4/EV4b CV/U87/mock/LCL D9.JPG]

EV5

a

GBM6

GBM12

GBM43

GBM123

GBM148

GBM245

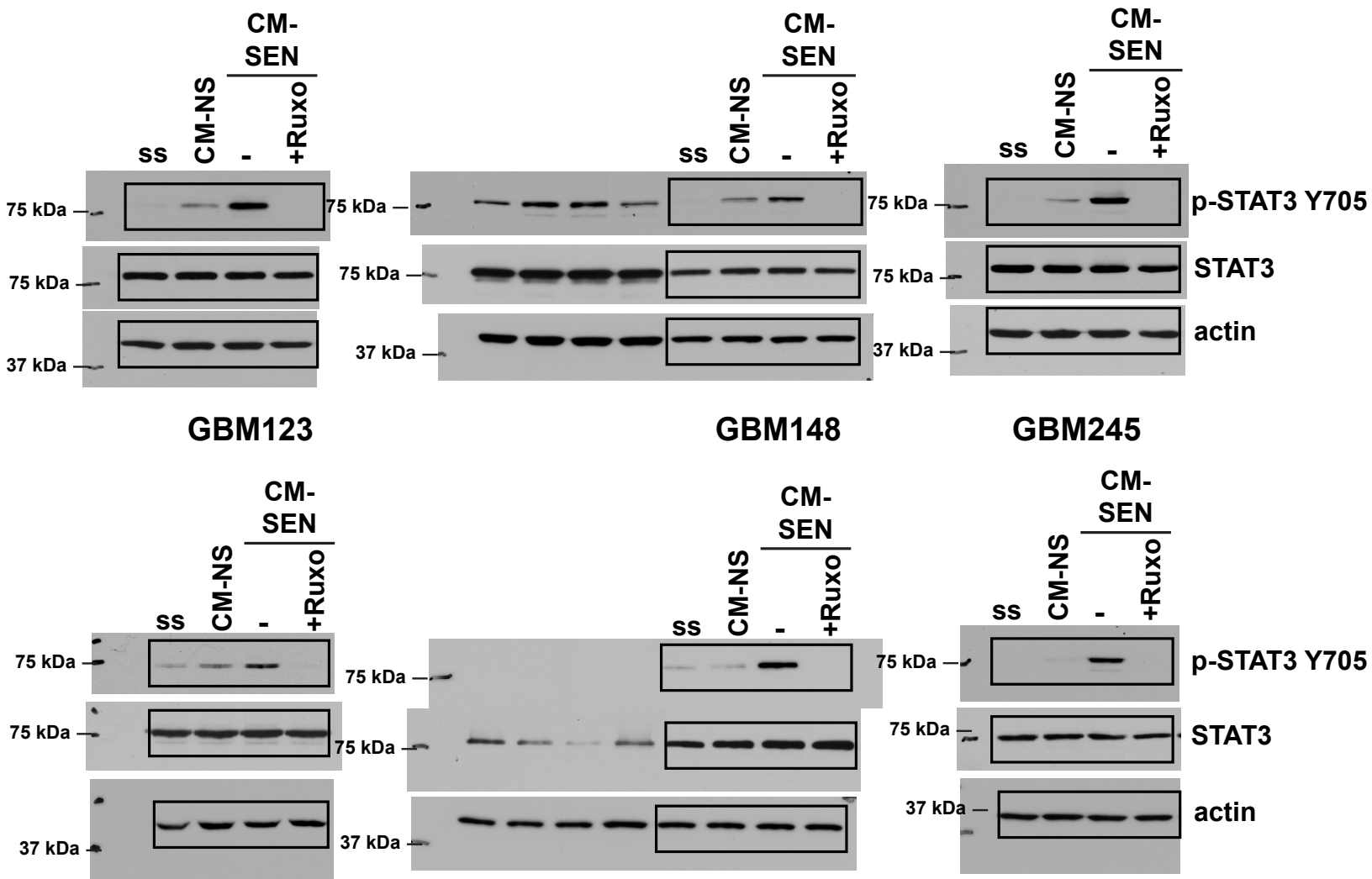

Supplement: Supplementary file 10 — EV figures [file 44321_2025_201_MOESM10_ESM.zip › source data for EV/EV5/EV5a PDX CM RUXO-uncropped.pdf]

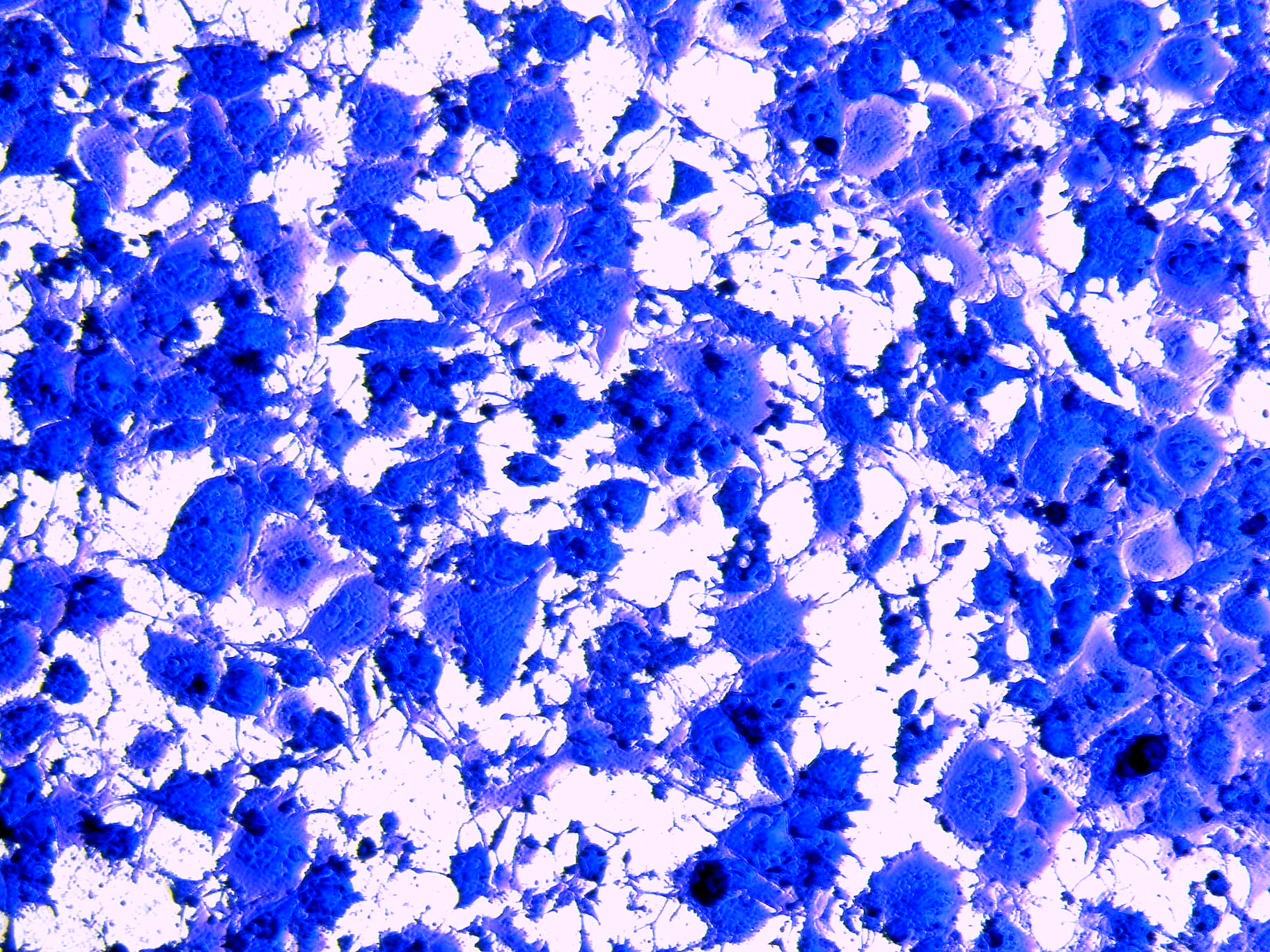

Supplement: Supplementary file 10 — EV figures [file 44321_2025_201_MOESM10_ESM.zip › source data for EV/EV5/EV5c CV/GBM12/IR/Biri-D0.JPG]

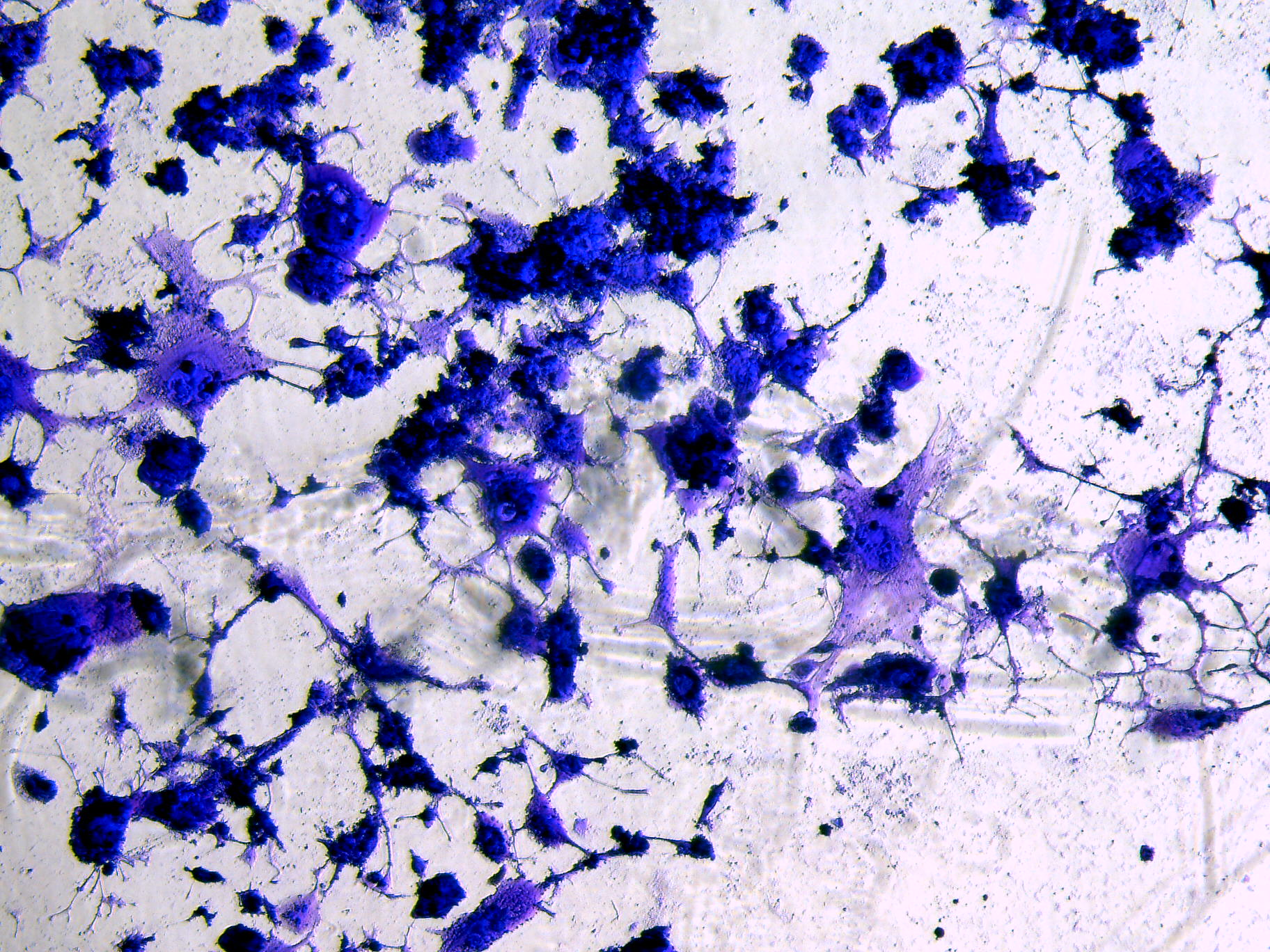

Supplement: Supplementary file 10 — EV figures [file 44321_2025_201_MOESM10_ESM.zip › source data for EV/EV5/EV5c CV/GBM12/IR/Biri-D3.JPG]

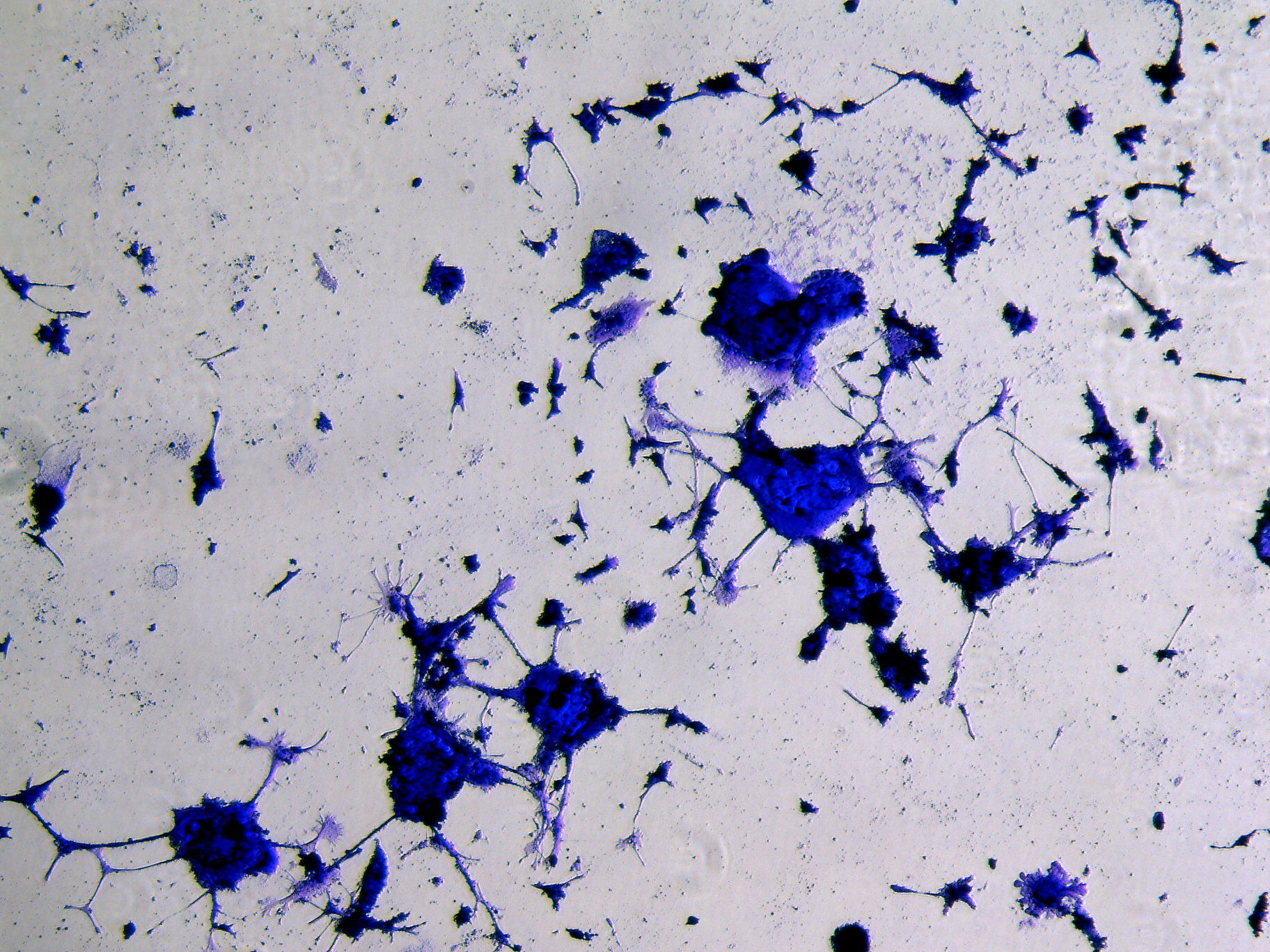

Supplement: Supplementary file 10 — EV figures [file 44321_2025_201_MOESM10_ESM.zip › source data for EV/EV5/EV5c CV/GBM12/IR/Biri-D6.JPG]

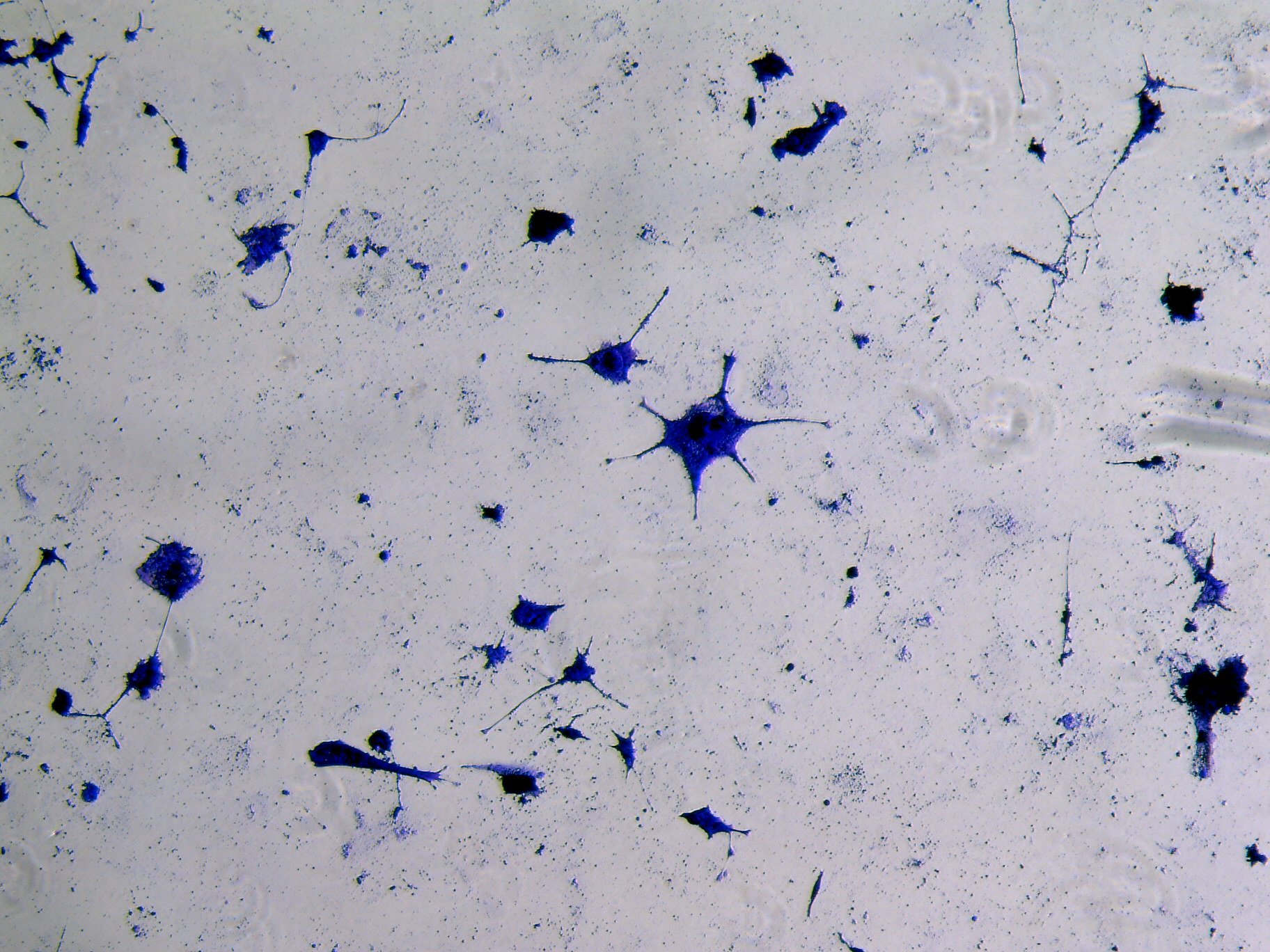

Supplement: Supplementary file 10 — EV figures [file 44321_2025_201_MOESM10_ESM.zip › source data for EV/EV5/EV5c CV/GBM12/IR/Biri-D9.JPG]

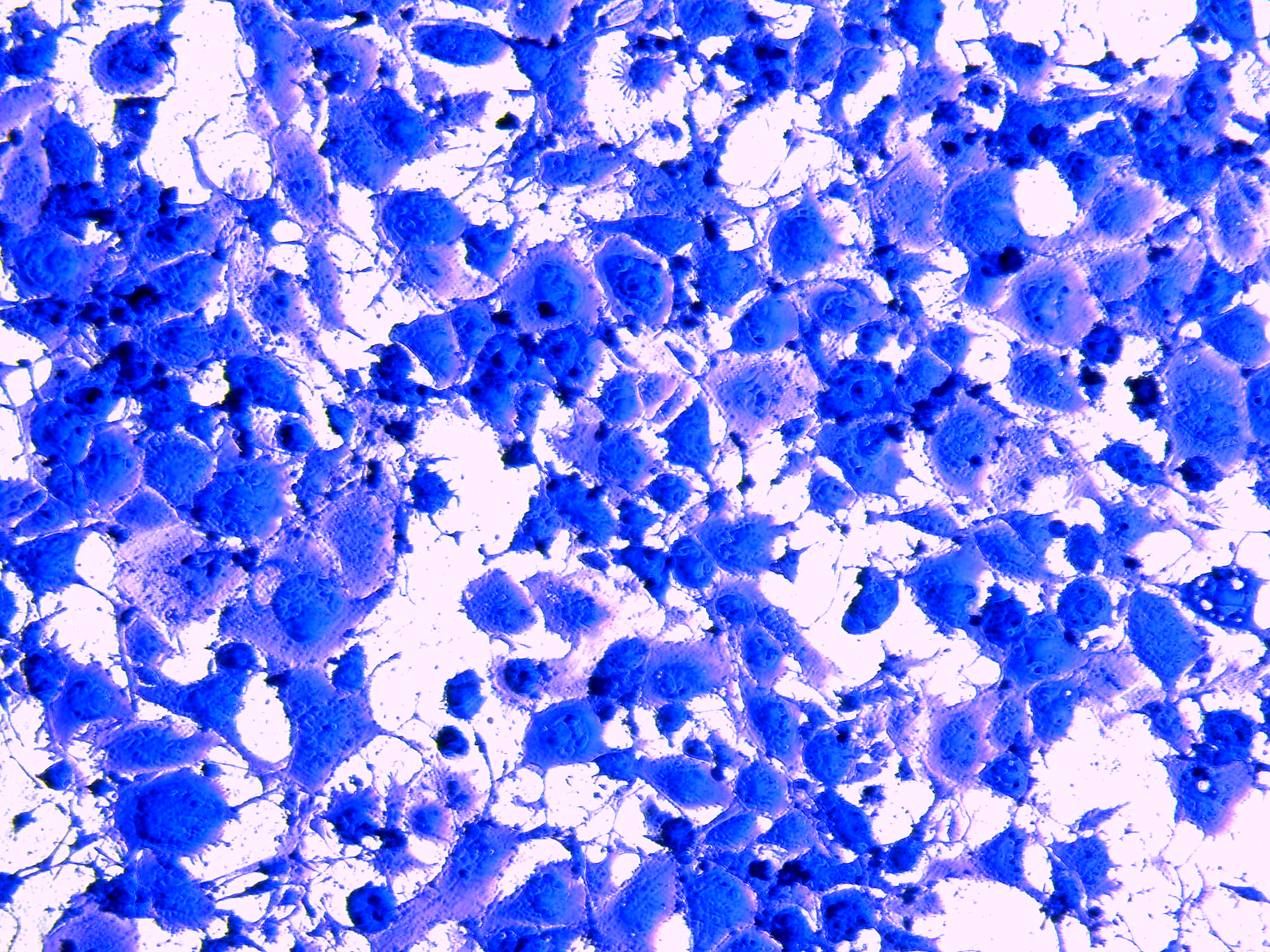

Supplement: Supplementary file 10 — EV figures [file 44321_2025_201_MOESM10_ESM.zip › source data for EV/EV5/EV5c CV/GBM12/IR/DMSO-D0.JPG]

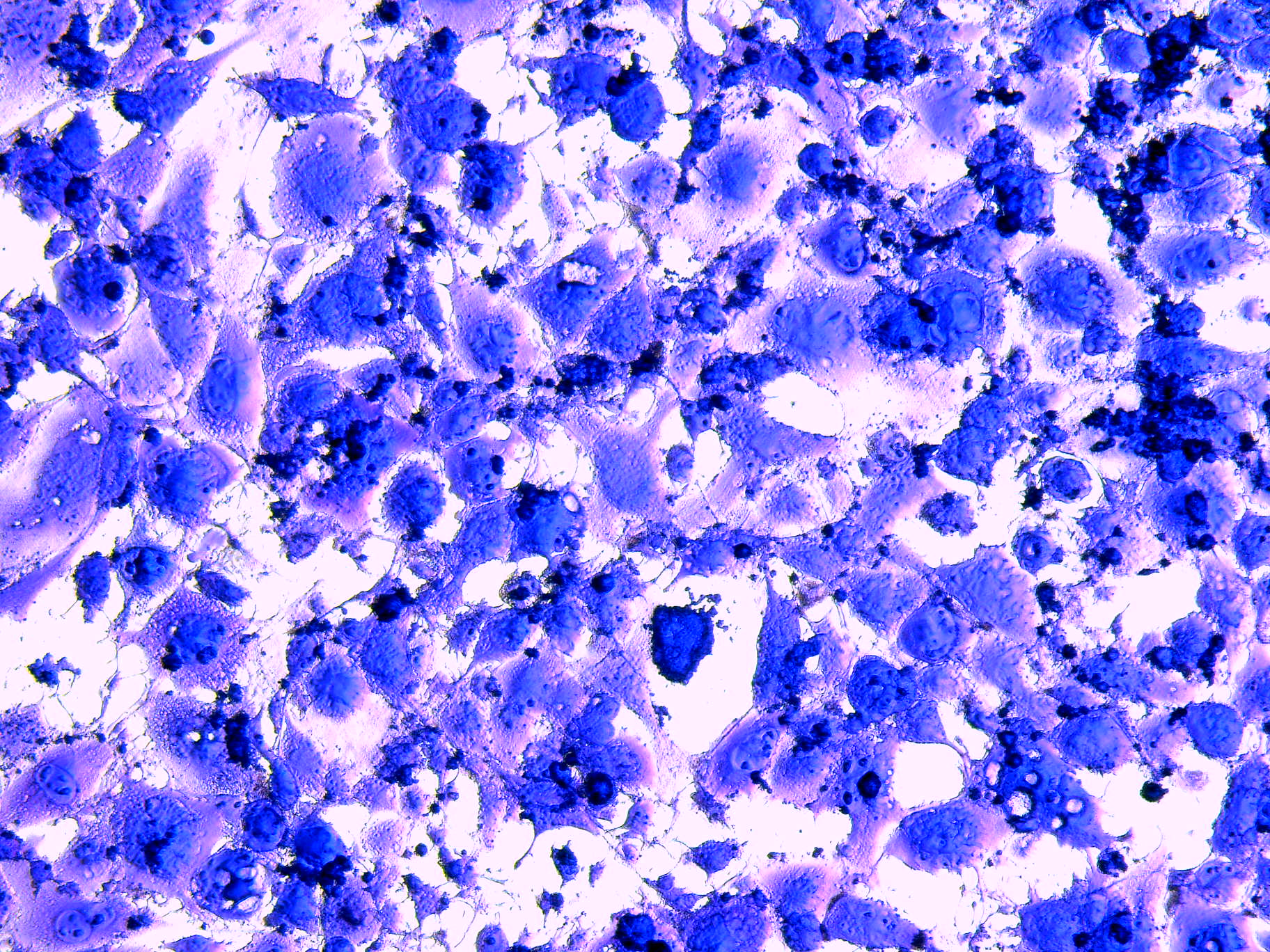

Supplement: Supplementary file 10 — EV figures [file 44321_2025_201_MOESM10_ESM.zip › source data for EV/EV5/EV5c CV/GBM12/IR/DMSO-D3.JPG]

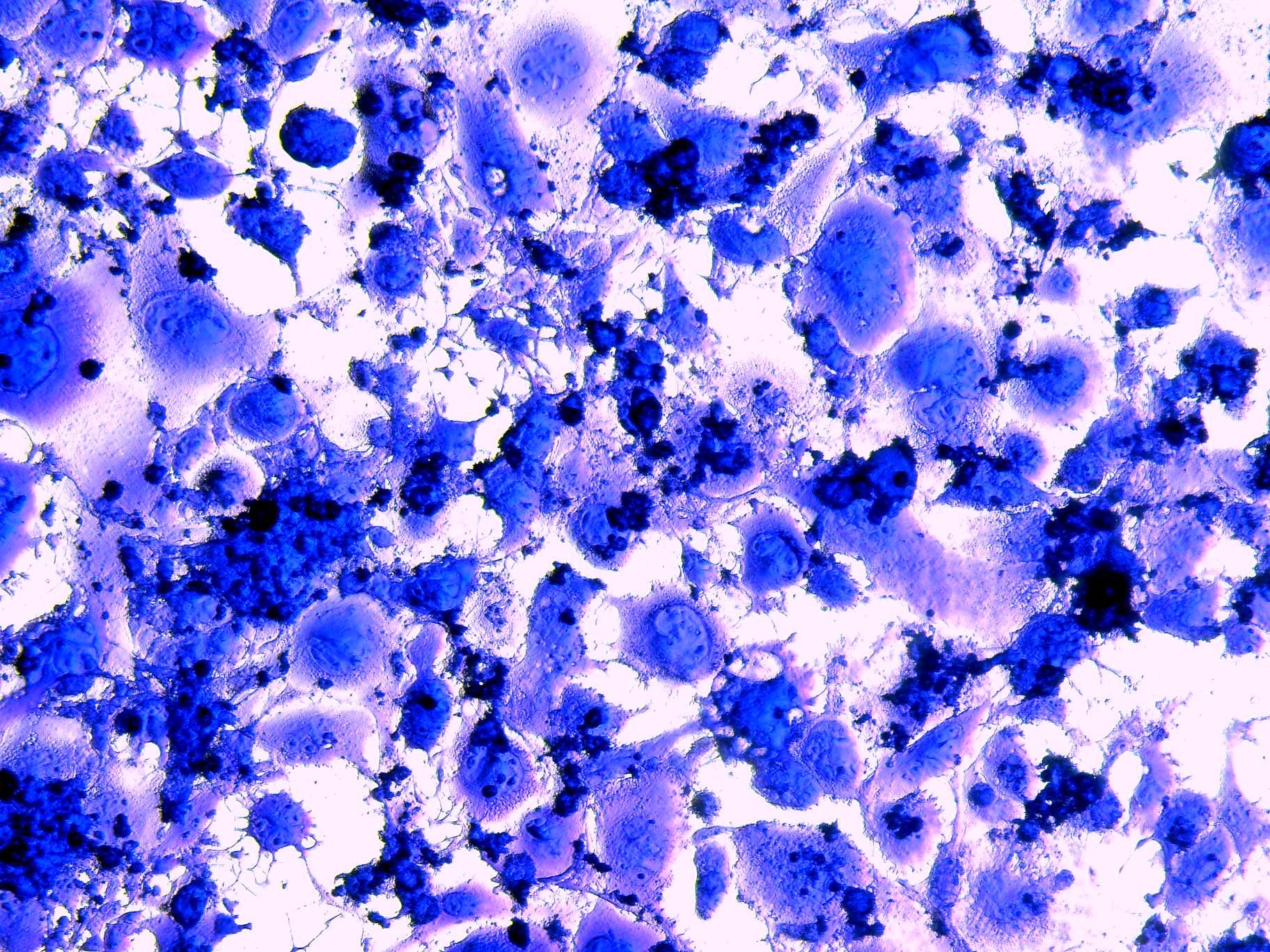

Supplement: Supplementary file 10 — EV figures [file 44321_2025_201_MOESM10_ESM.zip › source data for EV/EV5/EV5c CV/GBM12/IR/DMSO-D6.JPG]

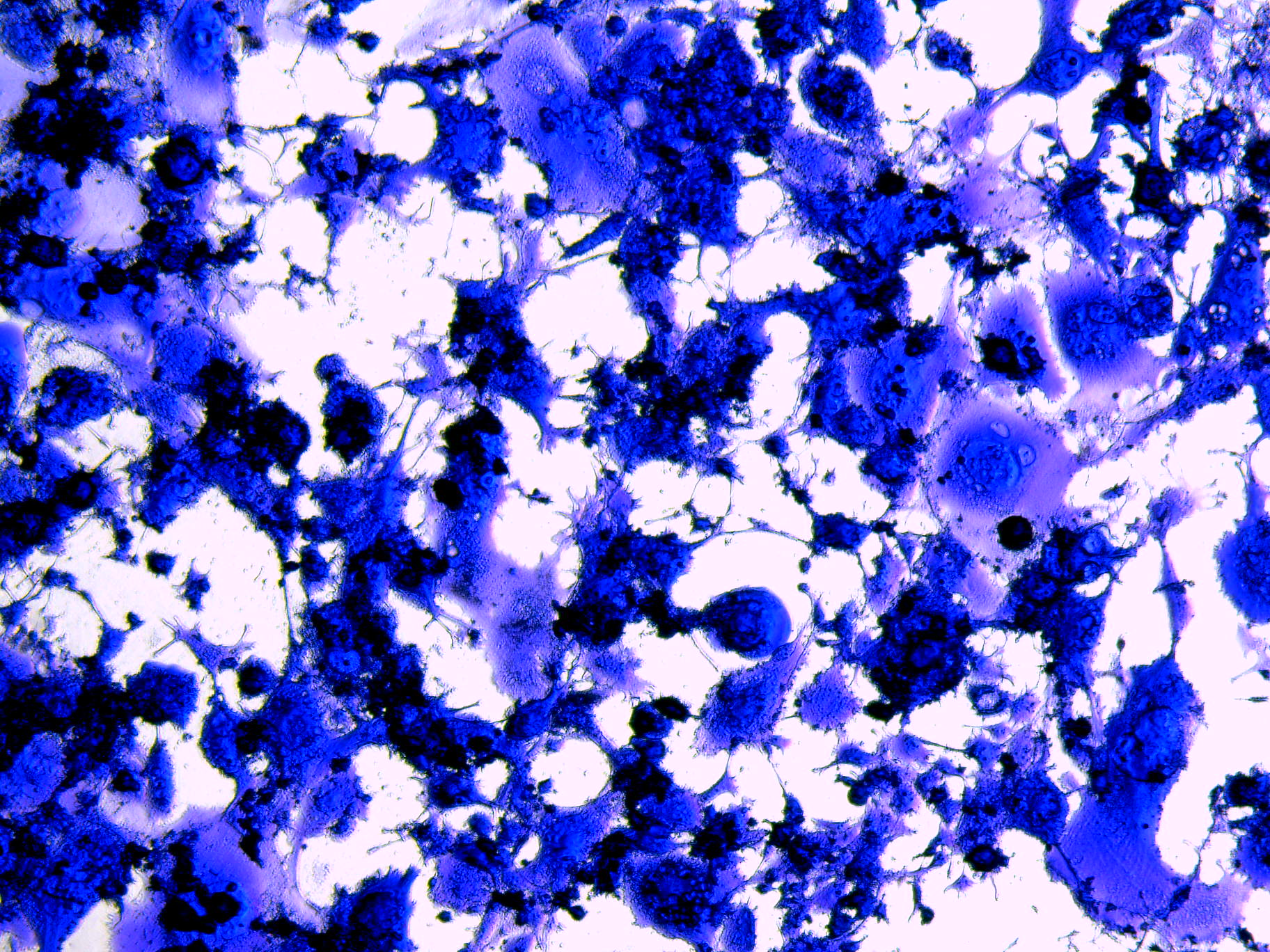

Supplement: Supplementary file 10 — EV figures [file 44321_2025_201_MOESM10_ESM.zip › source data for EV/EV5/EV5c CV/GBM12/IR/DMSO-D9.JPG]

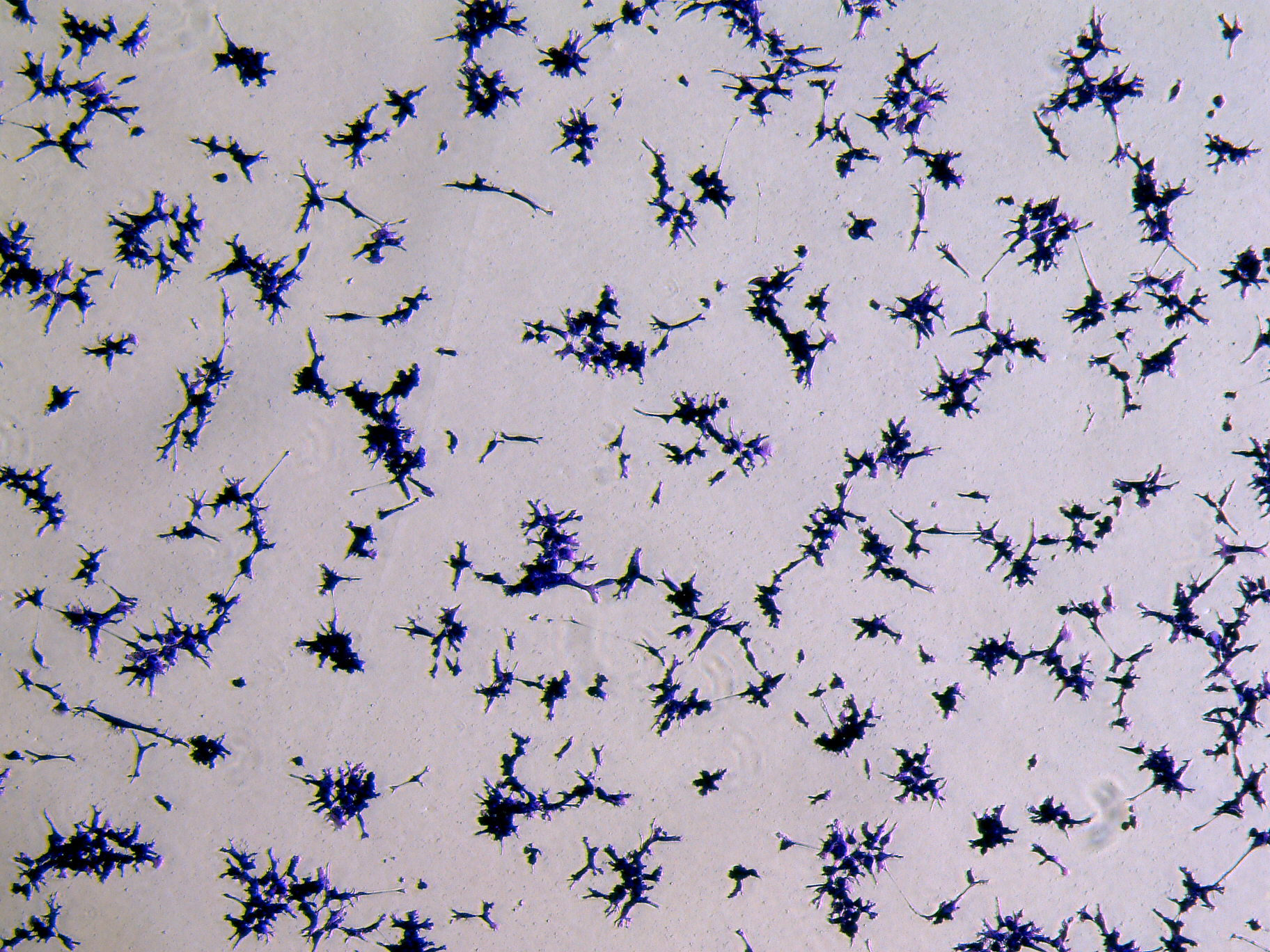

Supplement: Supplementary file 10 — EV figures [file 44321_2025_201_MOESM10_ESM.zip › source data for EV/EV5/EV5c CV/GBM12/mock/Biri-D0.JPG]

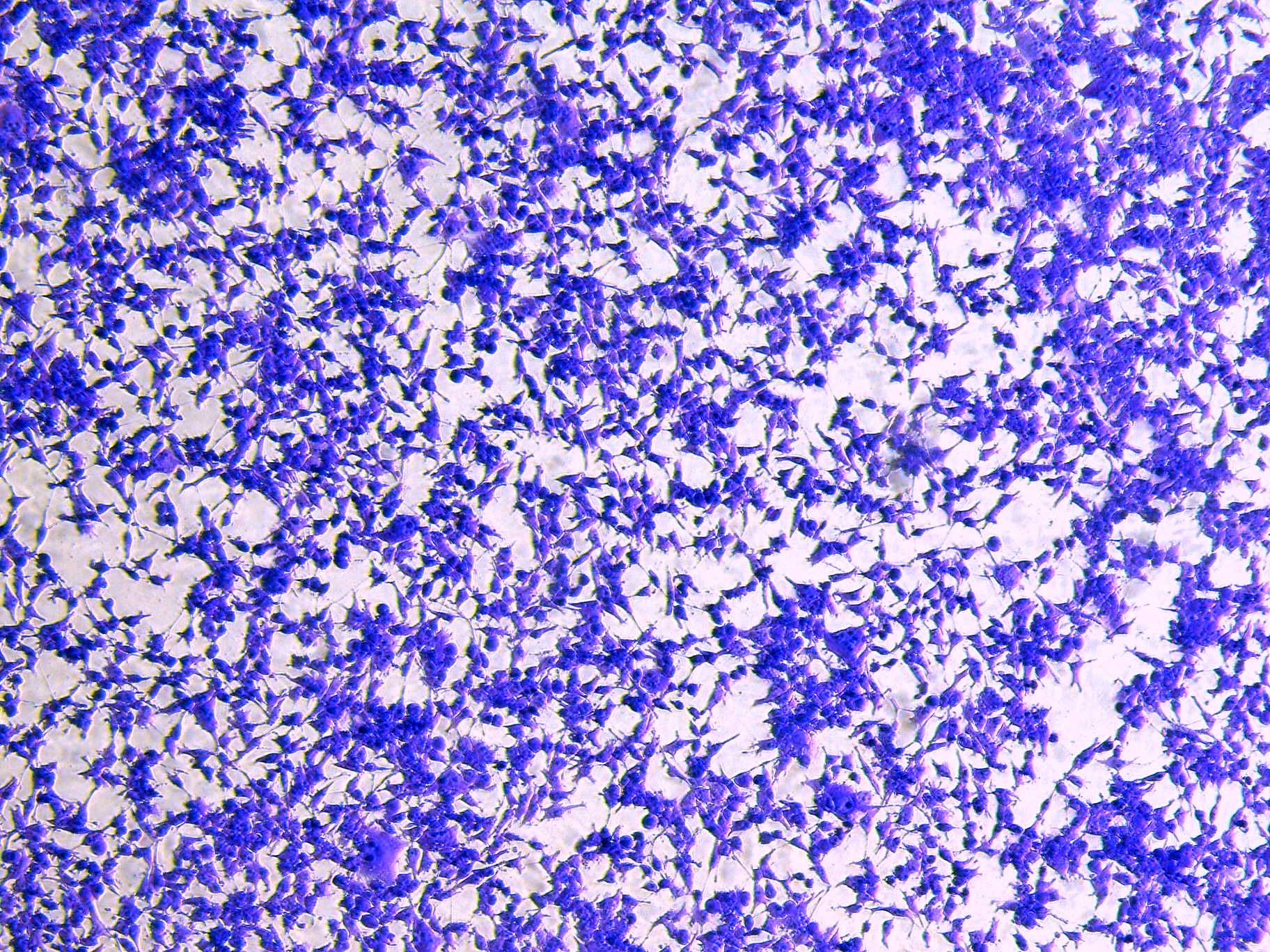

Supplement: Supplementary file 10 — EV figures [file 44321_2025_201_MOESM10_ESM.zip › source data for EV/EV5/EV5c CV/GBM12/mock/Biri-D3.JPG]

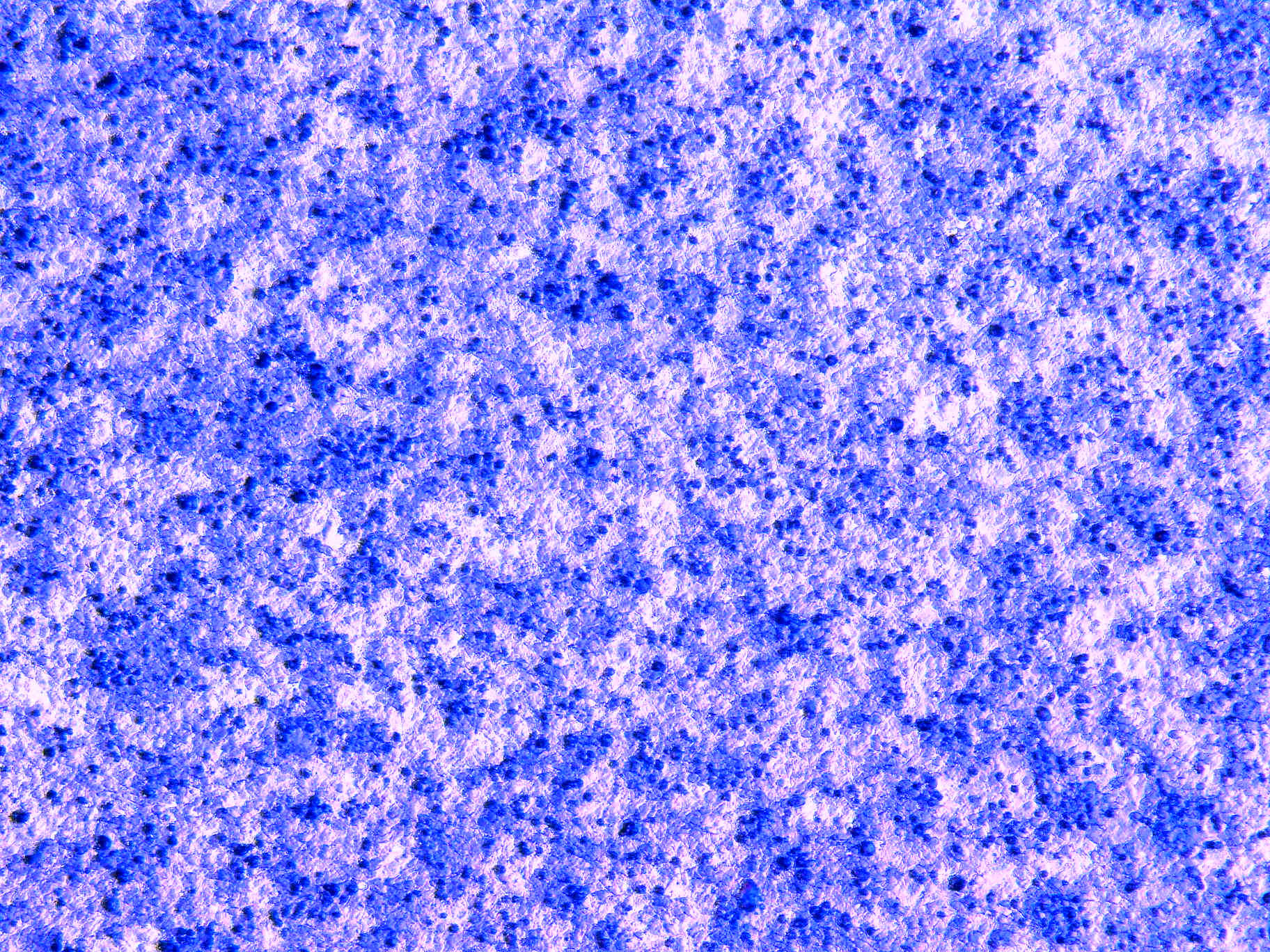

Supplement: Supplementary file 10 — EV figures [file 44321_2025_201_MOESM10_ESM.zip › source data for EV/EV5/EV5c CV/GBM12/mock/Biri-D6.JPG]

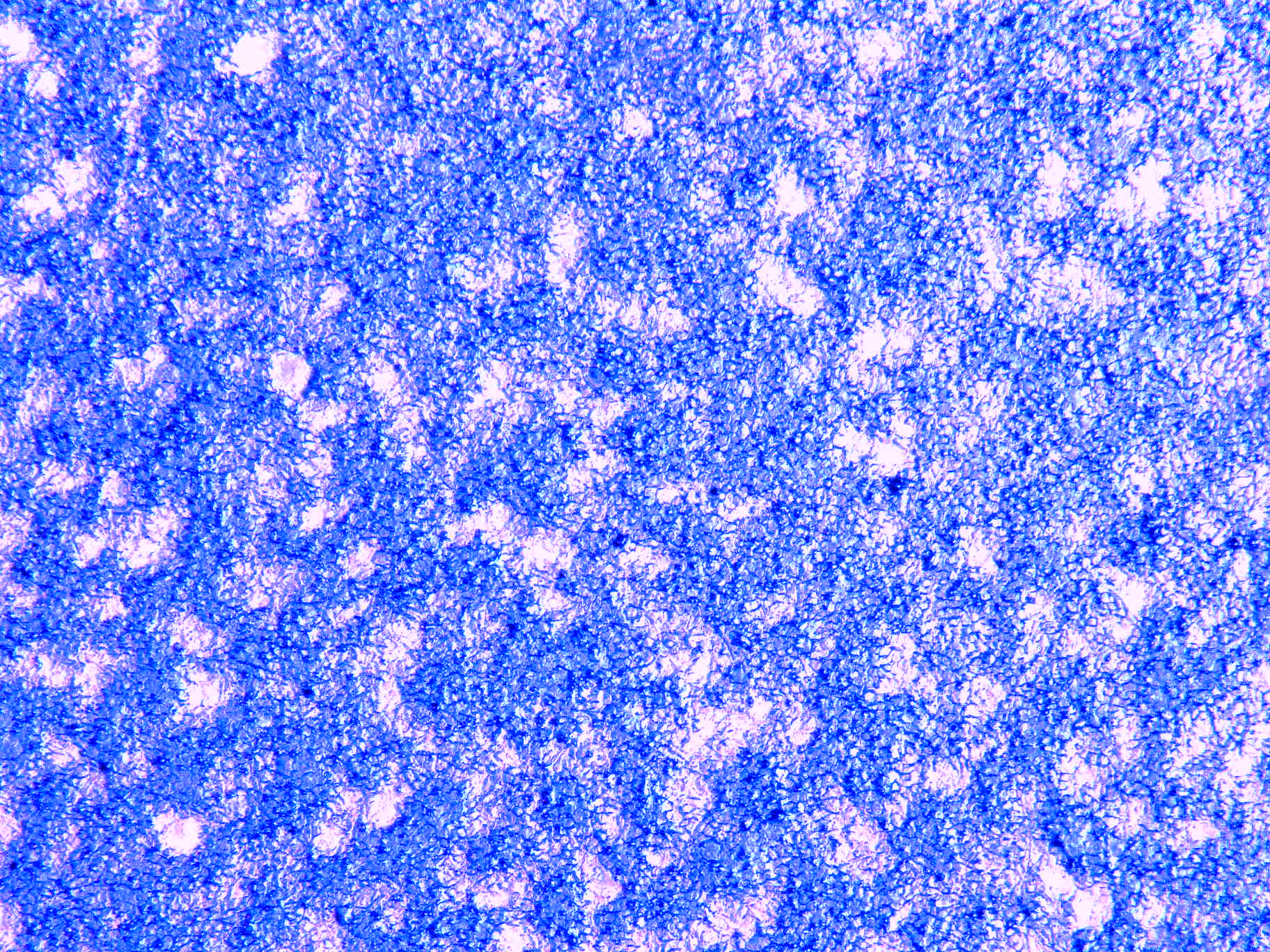

Supplement: Supplementary file 10 — EV figures [file 44321_2025_201_MOESM10_ESM.zip › source data for EV/EV5/EV5c CV/GBM12/mock/Biri-D9.JPG]

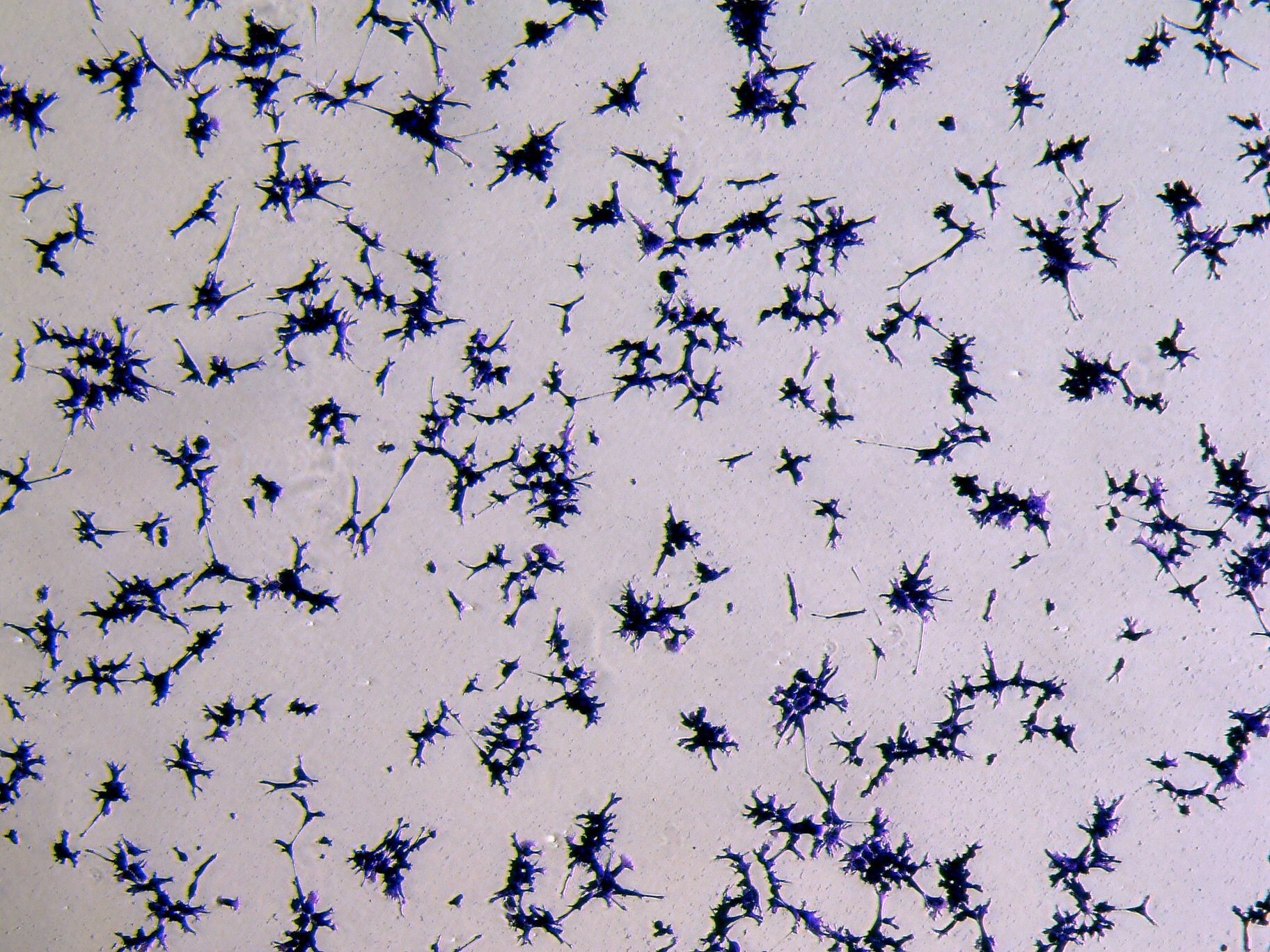

Supplement: Supplementary file 10 — EV figures [file 44321_2025_201_MOESM10_ESM.zip › source data for EV/EV5/EV5c CV/GBM12/mock/DMSO-D0.JPG]

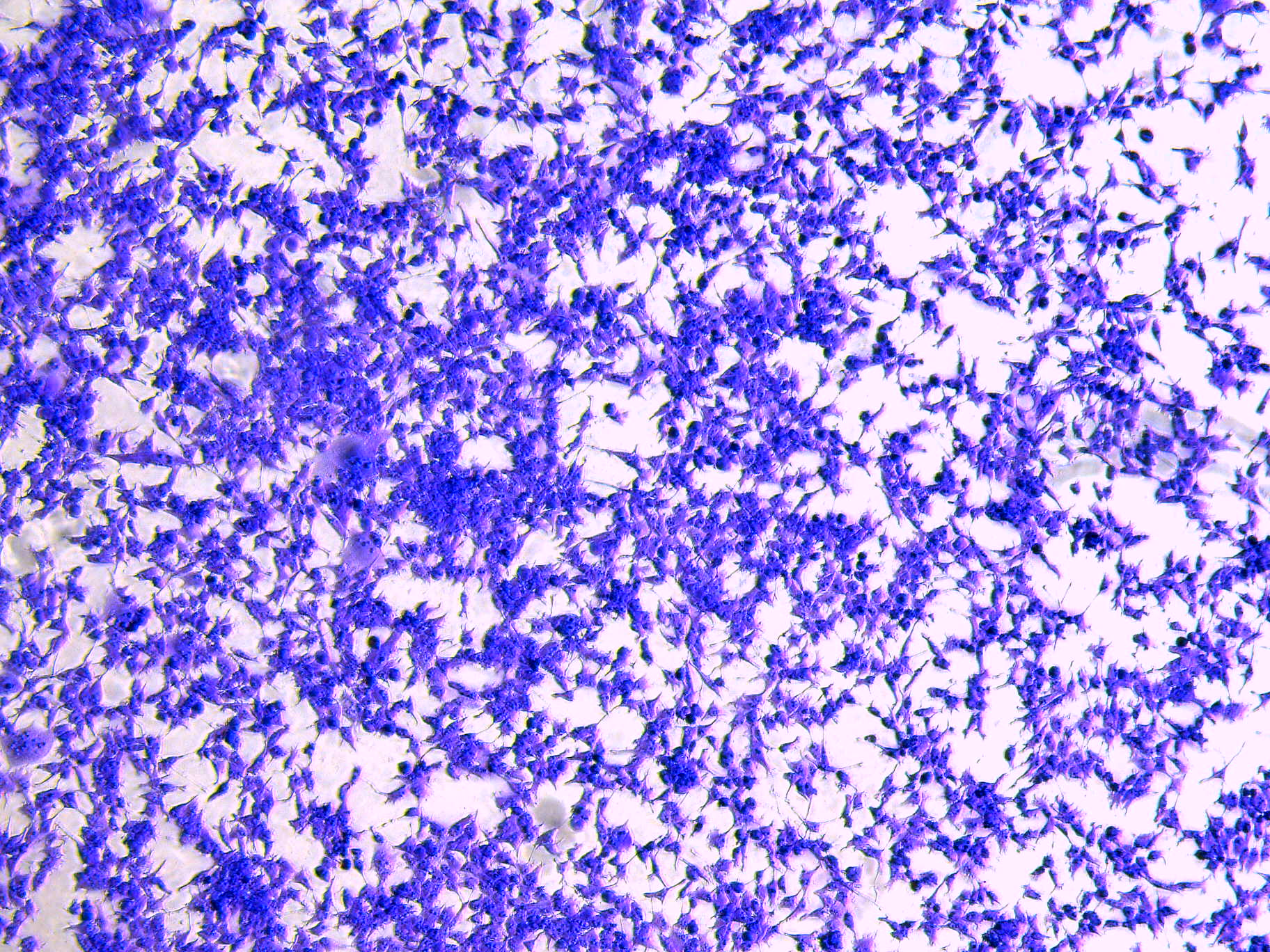

Supplement: Supplementary file 10 — EV figures [file 44321_2025_201_MOESM10_ESM.zip › source data for EV/EV5/EV5c CV/GBM12/mock/DMSO-D3.JPG]

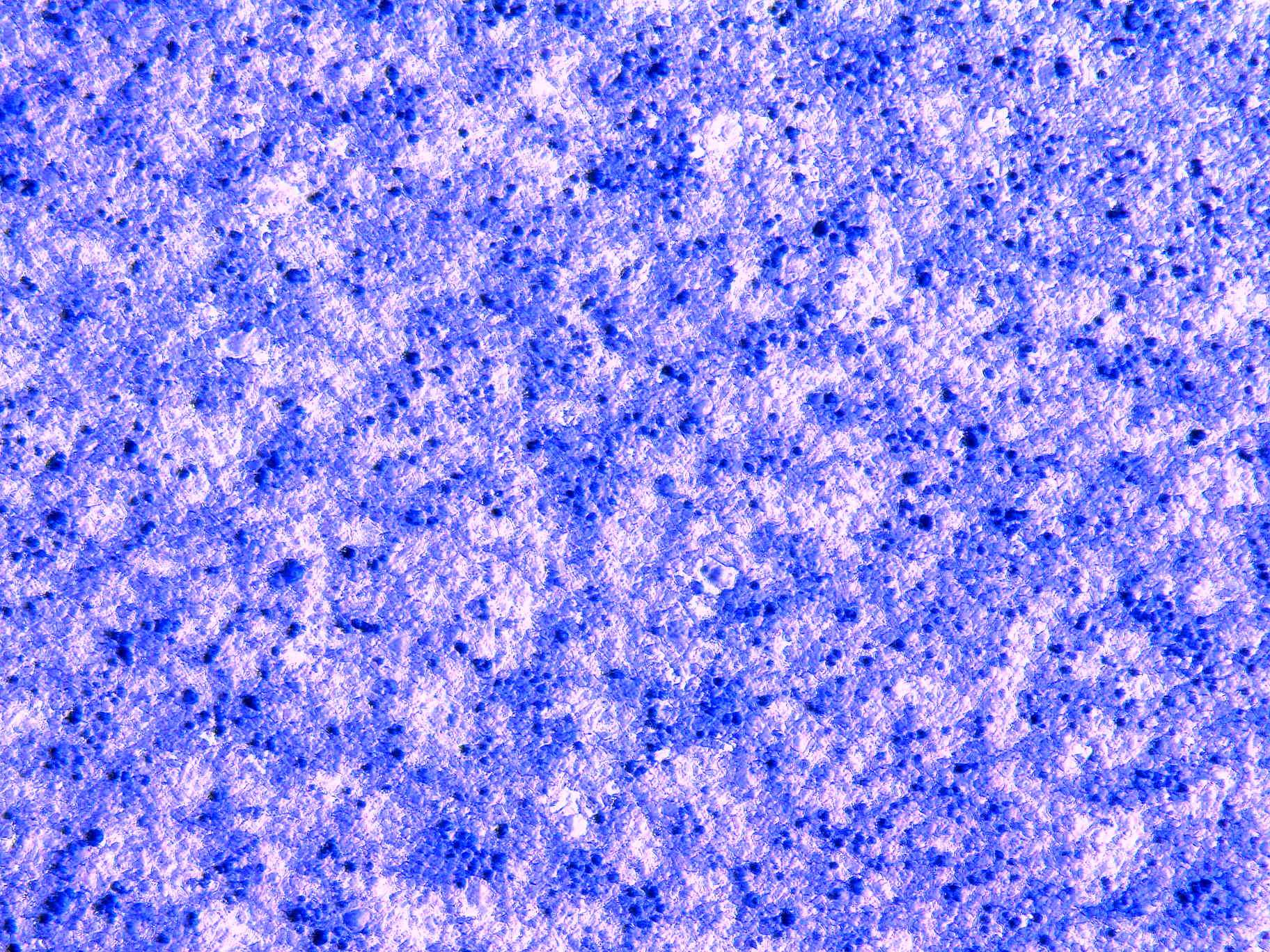

Supplement: Supplementary file 10 — EV figures [file 44321_2025_201_MOESM10_ESM.zip › source data for EV/EV5/EV5c CV/GBM12/mock/DMSO-D6.JPG]

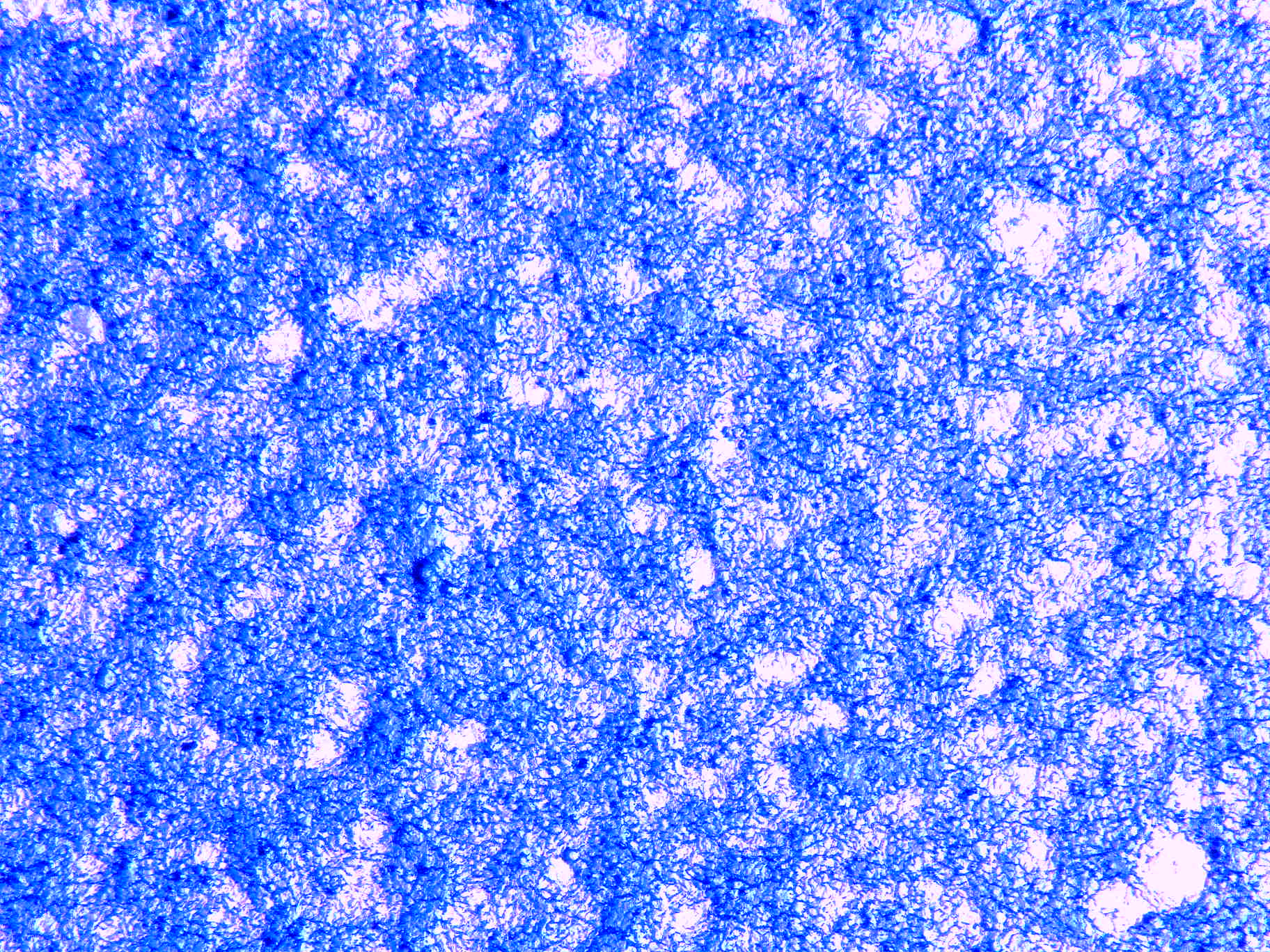

Supplement: Supplementary file 10 — EV figures [file 44321_2025_201_MOESM10_ESM.zip › source data for EV/EV5/EV5c CV/GBM12/mock/DMSO-D9.JPG]

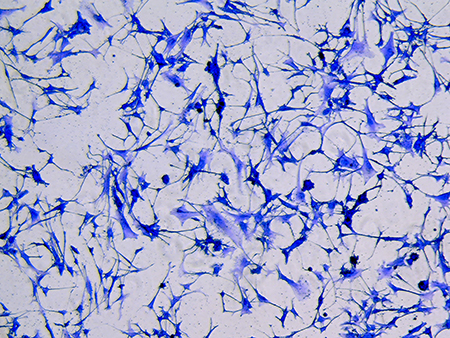

Supplement: Supplementary file 10 — EV figures [file 44321_2025_201_MOESM10_ESM.zip › source data for EV/EV5/EV5c CV/GBM123/IR/IR-Biri-D0.jpg]

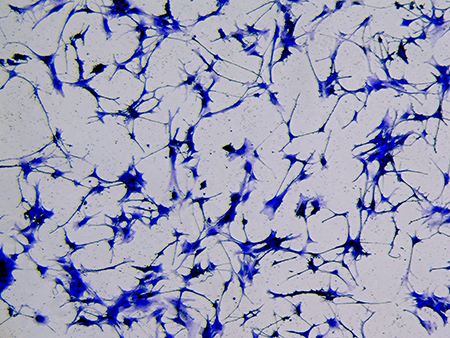

Supplement: Supplementary file 10 — EV figures [file 44321_2025_201_MOESM10_ESM.zip › source data for EV/EV5/EV5c CV/GBM123/IR/IR-Biri-D3.jpg]

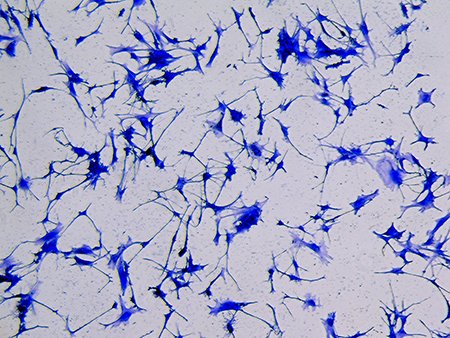

Supplement: Supplementary file 10 — EV figures [file 44321_2025_201_MOESM10_ESM.zip › source data for EV/EV5/EV5c CV/GBM123/IR/IR-Biri-D6.jpg]

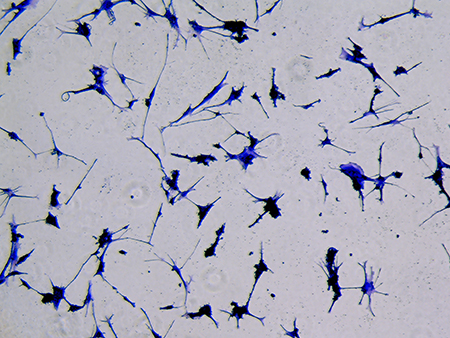

Supplement: Supplementary file 10 — EV figures [file 44321_2025_201_MOESM10_ESM.zip › source data for EV/EV5/EV5c CV/GBM123/IR/IR-Biri-D9.jpg]

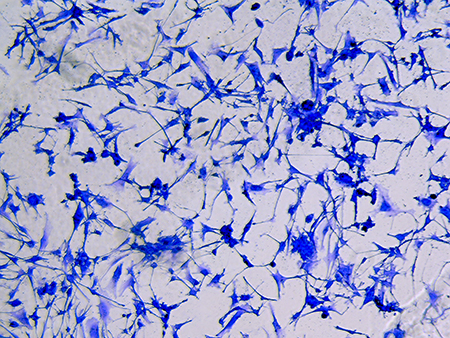

Supplement: Supplementary file 10 — EV figures [file 44321_2025_201_MOESM10_ESM.zip › source data for EV/EV5/EV5c CV/GBM123/IR/IR-DMSO-D0.jpg]

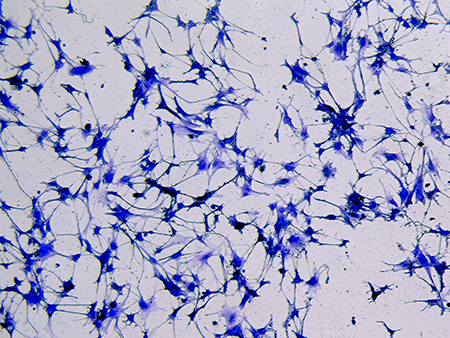

Supplement: Supplementary file 10 — EV figures [file 44321_2025_201_MOESM10_ESM.zip › source data for EV/EV5/EV5c CV/GBM123/IR/IR-DMSO-D3.jpg]

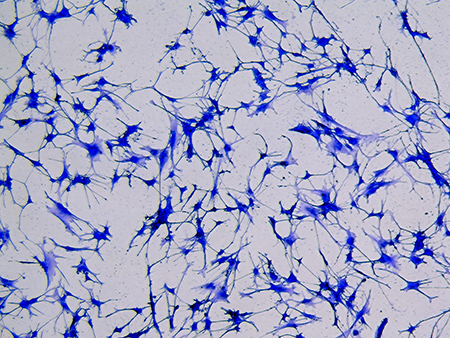

Supplement: Supplementary file 10 — EV figures [file 44321_2025_201_MOESM10_ESM.zip › source data for EV/EV5/EV5c CV/GBM123/IR/IR-DMSO-D6.jpg]

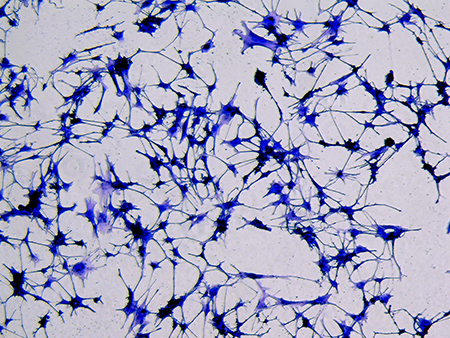

Supplement: Supplementary file 10 — EV figures [file 44321_2025_201_MOESM10_ESM.zip › source data for EV/EV5/EV5c CV/GBM123/IR/IR-DMSO-D9.jpg]

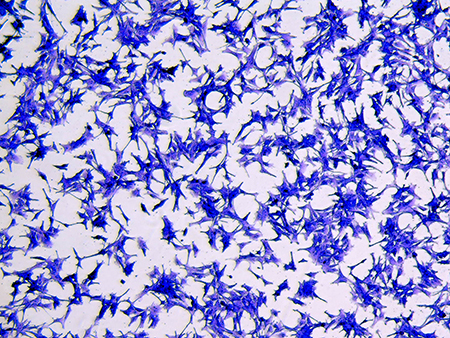

Supplement: Supplementary file 10 — EV figures [file 44321_2025_201_MOESM10_ESM.zip › source data for EV/EV5/EV5c CV/GBM123/mock/Biri-Naive-D0.jpg]

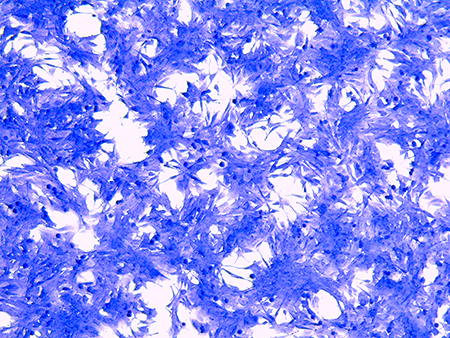

Supplement: Supplementary file 10 — EV figures [file 44321_2025_201_MOESM10_ESM.zip › source data for EV/EV5/EV5c CV/GBM123/mock/Biri-Naive-D3.jpg]

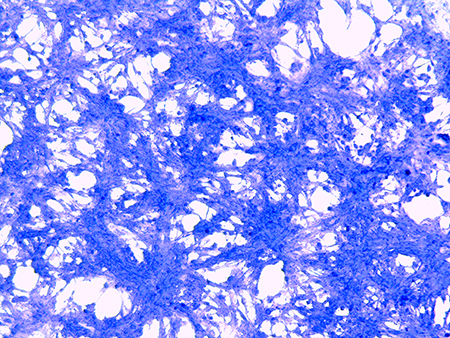

Supplement: Supplementary file 10 — EV figures [file 44321_2025_201_MOESM10_ESM.zip › source data for EV/EV5/EV5c CV/GBM123/mock/Biri-Naive-D6.jpg]

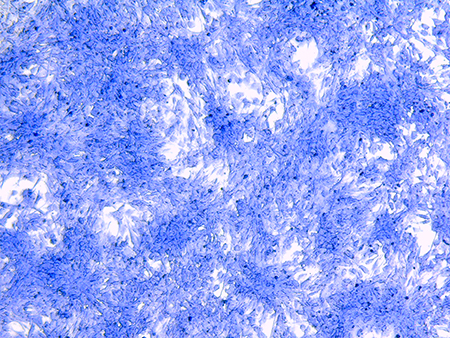

Supplement: Supplementary file 10 — EV figures [file 44321_2025_201_MOESM10_ESM.zip › source data for EV/EV5/EV5c CV/GBM123/mock/Biri-Naive-D9.jpg]

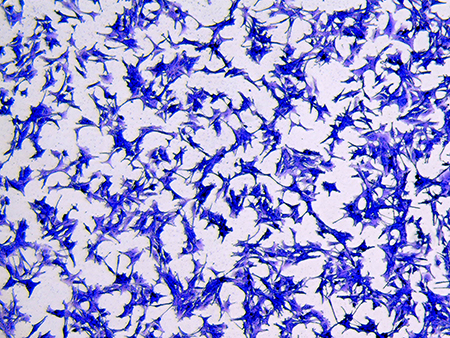

Supplement: Supplementary file 10 — EV figures [file 44321_2025_201_MOESM10_ESM.zip › source data for EV/EV5/EV5c CV/GBM123/mock/DMSO-Naive-D0.jpg]

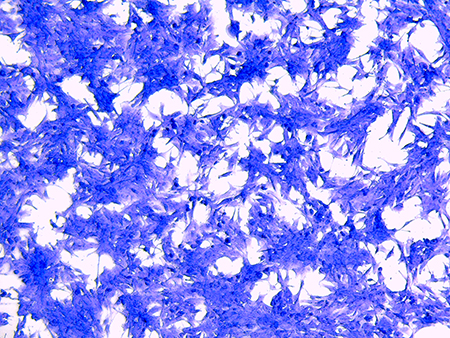

Supplement: Supplementary file 10 — EV figures [file 44321_2025_201_MOESM10_ESM.zip › source data for EV/EV5/EV5c CV/GBM123/mock/DMSO-Naive-D3.jpg]

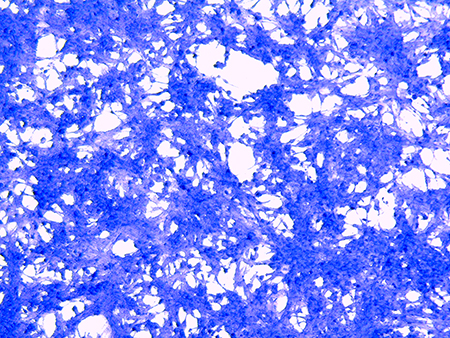

Supplement: Supplementary file 10 — EV figures [file 44321_2025_201_MOESM10_ESM.zip › source data for EV/EV5/EV5c CV/GBM123/mock/DMSO-Naive-D6.jpg]

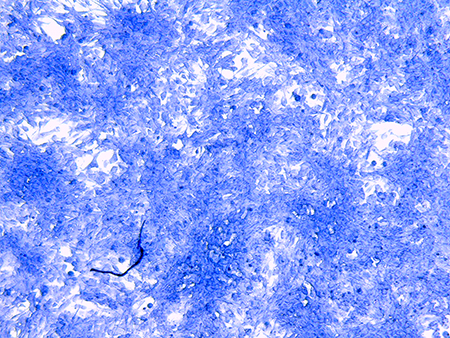

Supplement: Supplementary file 10 — EV figures [file 44321_2025_201_MOESM10_ESM.zip › source data for EV/EV5/EV5c CV/GBM123/mock/DMSO-Naive-D9.jpg]
