# Supplementary material for: Targeting cIAP2 in a novel senolytic strategy prevents glioblastoma recurrence after radiotherapy
Source: EMBO Mol Med. 2025 Feb 19;17(4):645–78. doi: 10.1038/s44321-025-00201-x (PMC11982261; doi:10.1038/s44321-025-00201-x)
Supplement: Supplementary file 11 — Expanded View Figures [file 44321_2025_201_MOESM11_ESM.pdf]

Expanded View Figures

Figure EV1. Senescent GBM cells secrete SASP factors that can potentially activate the JAK-STAT3 and NF-kB pathways.

(**A**) Representative images of GBM cell lines immunofluorescence stained for Lamin B1 (green) and (**B**) Ki67 (green), 10 days after irradiation (IR) with 10 Gy of X-rays or mock-irradiation (Mock). $n = 3$ with at least 100 nuclei scored for each replicate. Nuclei are stained with DAPI (blue). Plots show mean percentages +/− SD of Lamin B1- or Ki67-positive cells. A two-tailed Student's *t* test was performed; Lamin B1 - LN229 $P = 0.00000163109$, A172 $P = 0.00000004724$, U118 $P = 0.00000000002$, U87 $P = 0.00000702223$; Ki67 - LN229 $P = 0.000030$, A172 $p = 0.000002$, U118 $p = 0.000028$, U87 $P = 0.000005$. Scale bar, 50 µm. (**C**) Heatmap of top 100 differentially expressed genes in LN229 or A172 GBM cells mock-irradiated (Mock) or irradiated (IR) with 10 Gy of X-rays and then allowed to recover for 10 days ($n = 3$), as assessed by RNA sequencing. (**D**) Senescence scores of mock-irradiated or irradiated LN229 and A172 cells ($n = 3$) generated by analysis of RNA-seq datasets using the SenCan Classifier tool. Score ranges from 0 (no senescence) to 1 (senescence). Plot shows mean senescence score +/− SD for both GBM cell lines. A two-tailed Student's *t* test was performed; LN229 $P = 0.00002368169637$, A172 $p = 0.00000000000002$. (**E**) Genes involved in SASP, Cytokine-cytokine receptor interaction, JAK-STAT signaling, and NF-kB signaling are significantly enriched in irradiated cells compared to mock-irradiated cells. Normalized Enrichment Scores (NES) are shown in the figure; $P = 0$. (**F**) SASP-transcription factor network generated by the list of 24 common cytokines secreted by all four senescent GBM cell lines cross-referenced against the TRRUST database. The visualization was generated using Cytoscape version 3.9.1. Transcription factors are shown in green. The cytokines not shown had no available information in the database. Source data are available online for this figure.

 © The Author(s)

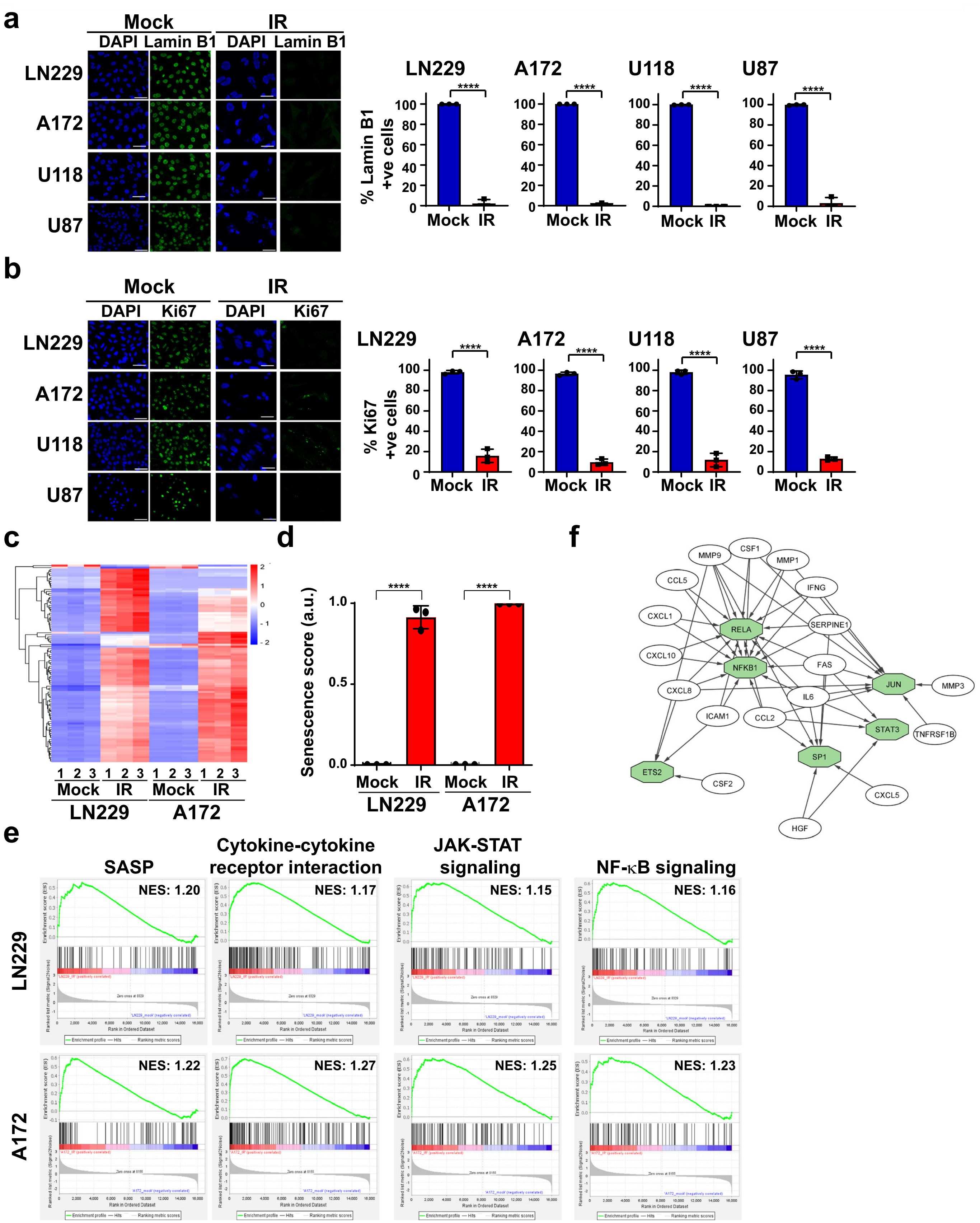
a
Mock
IR
DAPI Lamin B1 DAPI Lamin B1
LN229
A172
U118
U87
% Lamin B1 +ve cells
Mock IR
****
b
Mock
IR
DAPI Ki67 DAPI Ki67
% Ki67 +ve cells
c
1 2 3 1 2 3 1 2 3 1 2 3
Mock IR Mock IR
LN229 A172
d
Senescence score (a.u.)
f
MMP9 CSF1 MMP1 CCL5 IFNG CXCL1 RELA SERPINE1 CXCL10 NFKB1 FAS JUN MMP3 CXCL8 IL6 ICAM1 CCL2 STAT3 TNFRSF1B SP1 ETS2 CSF2 CXCL5 HGF
e
SASP
Cytokine-cytokine receptor interaction
JAK-STAT signaling
NF-κB signaling
NES: 1.20
NES: 1.17
NES: 1.15
NES: 1.16
NES: 1.22
NES: 1.27
NES: 1.25
NES: 1.23

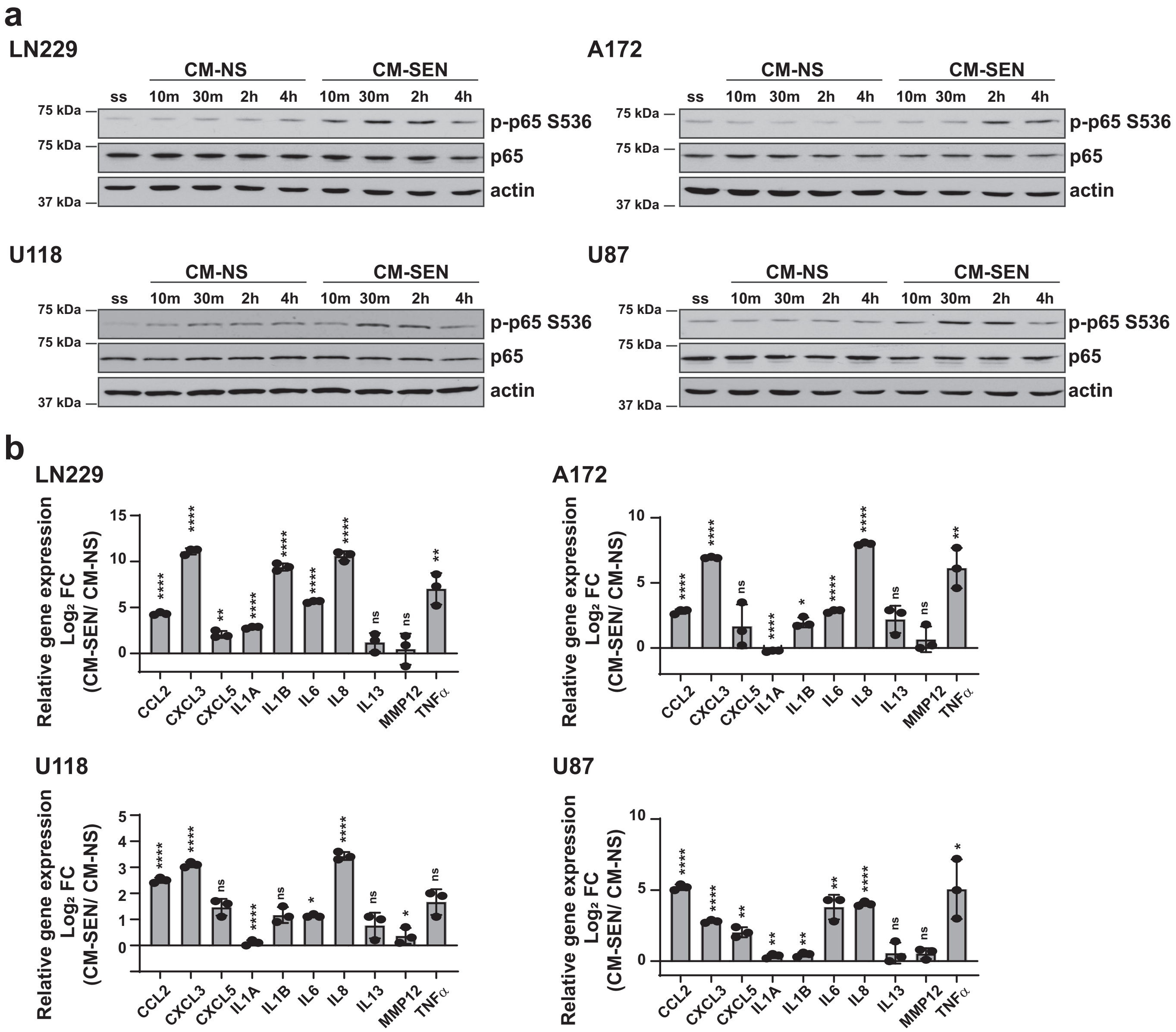

Figure EV2. Senescent GBM cells activate the NF-kB pathway in naive GBM cells.

(**A**) Naive GBM cells were exposed to conditioned media from senescent (CM-SEN) or non-senescent (CM-NS) GBM cell lines for the indicated times, and activation of the NF-kB pathway assessed by western blotting with anti-phopho-p65 (S536) antibody. Actin serves as loading control. Recipient cells were serum starved (ss) before addition of CM. (**B**) Serum starved GBM cells were exposed for 2 h to CM-NS or CM-SEN, and expression of SASP-related genes (relative to expression levels in serum starved cells) was assessed by qRT-PCR ($n = 3$ biological replicates comprising 3 technical replicates each). Plots show mean fold change in gene expression +/− SD of SASP-related genes in CM-SEN-treated cells relative to CM-NS-treated cells. A two-tailed Student's t test was performed; ns, not significant; $*P < 0.05$; $**P < 0.01$; $****P < 0.0001$ (please refer to Appendix Table S4 for the exact P values). Source data are available online for this figure.

 © The Author(s)

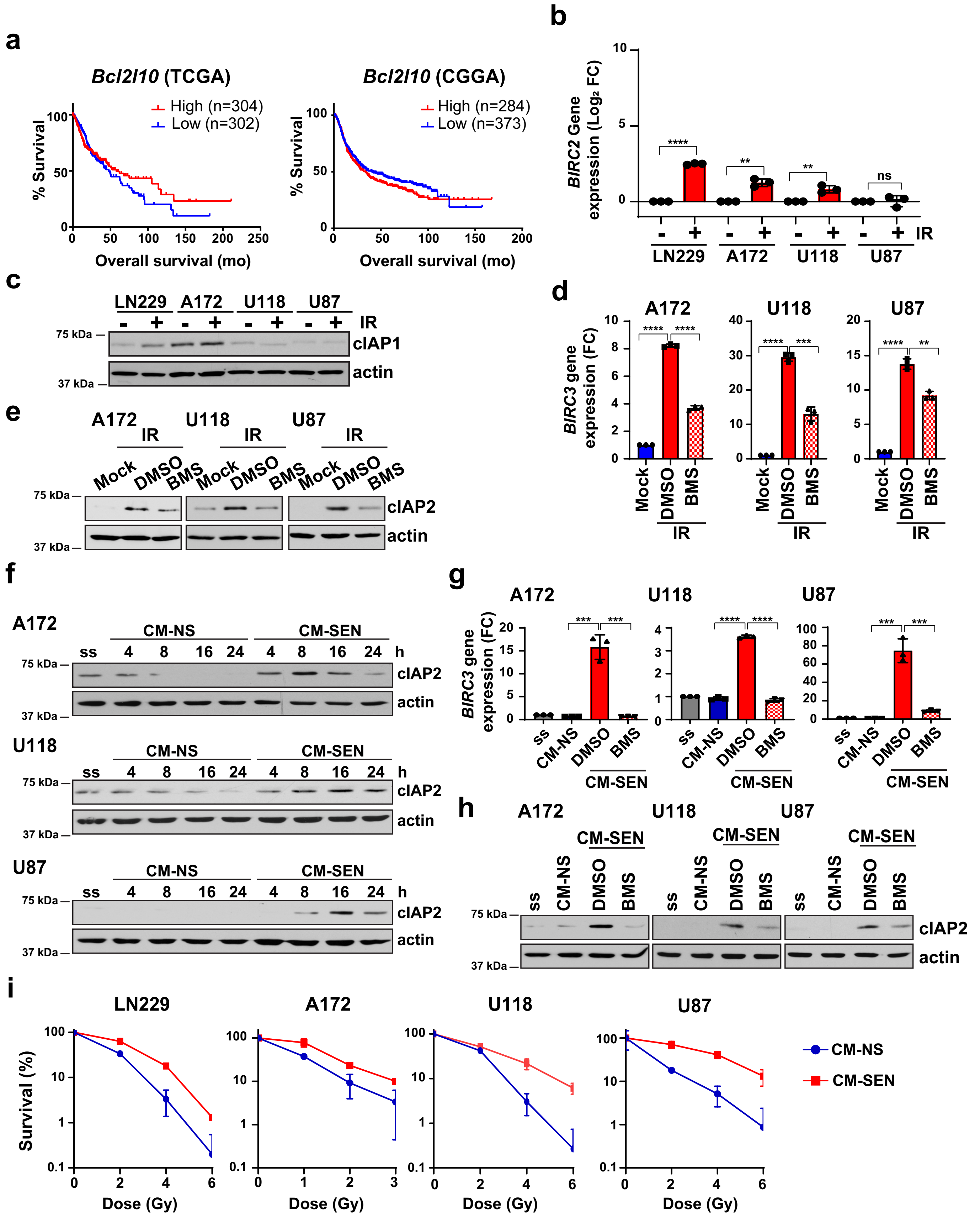
a
Bcl2l10 (TCGA)
High (n=304)
Low (n=302)
% Survival
Overall survival (mo)
Bcl2l10 (CGGA)
High (n=284)
Low (n=373)
b
BIRC2 Gene expression (Log₂ FC)
LN229
A172
U118
U87
IR
c
cIAP1
actin
75 kDa
37 kDa
d
BIRC3 gene expression (FC)
Mock
DMSO
BMS
e
cIAP2
f
CM-NS
CM-SEN
ss
h
g
h
i
LN229
Survival (%)
Dose (Gy)
CM-NS
CM-SEN

Figure EV3. Senescent GBM cells induce *BIRC3* in naive cells and promote resistance to ionizing radiation.

(**A**) Kaplan–Meier curve showing lack of correlation of *Bcl2l10* expression levels with prognosis in GBMLGG patients in TCGA ($n = 606$) and CGGA ($n = 657$) cohorts as evidenced by Hazard Ratio (logrank) of 0.9692 and 0.8629 and P values of 0.1943 and 0.2640, respectively. (**B**) Plot shows mean relative expression of *BIRC2* +/− SD in mock-irradiated vs. irradiated (IR) GBM cells 10 days after exposure to 10 Gy of X-rays, as assessed by qRT-PCR ($n = 3$ biological replicates comprising 3 technical replicates each). A two-tailed Student's t test was performed; exact P values from left to right: 0.0000001, 0.0010112, 0.0044335, 0.9295618. (**C**) Whole cell extracts from mock-irradiated or irradiated GBM cell lines were western blotted with anti-cIAP1 antibody. Actin serves as loading control. (**D**) Senescent GBM cells (10 days after exposure to 10 Gy) were treated with the IKK inhibitor BMS-345541 (BMS) or DMSO as control for 72 h ($n = 3$ biological replicates comprising 3 technical replicates each), and mean relative expression of *BIRC3* +/− SD was assessed by qRT-PCR (a two-tailed Student's t test was performed; A172 $p = 0.00000003$, 0.00000232; U118 $p = 0.00000220$, 0.00026887; U87 $P = 0.00000818$, 0.00118914, respectively) or (**E**) western blotting for cIAP2. (**F**) Naive GBM cells were exposed to conditioned media from senescent (CM-SEN) or non-senescent (CM-NS) cells for the indicated times, and expression of cIAP2 assessed by western blotting. (**G**) Naive GBM cells were treated with BMS-345541 or DMSO as control for 2 h before exposure to CM-SEN ($n = 3$ biological replicates comprising 3 technical replicates each), and mean relative expression of *BIRC3* +/− SD was assessed by qRT-PCR (a two-tailed Student's t test was performed; A172 $P = 0.000627$, 0.000622; U118 $P = 0.000001$, 0.000001; U87 $P = 0.000593$, 0.000936, respectively) or (**H**) western blotting for cIAP2. (**I**) GBM cells were exposed to conditioned media from senescent or non-senescent cells ($n = 3$) and radiation sensitivity measured by the colony survival assay. The mean percentage of surviving colonies +/− SD (y axis) is plotted against the corresponding radiation dose (x axis). Source data are available online for this figure.

 © The Author(s)

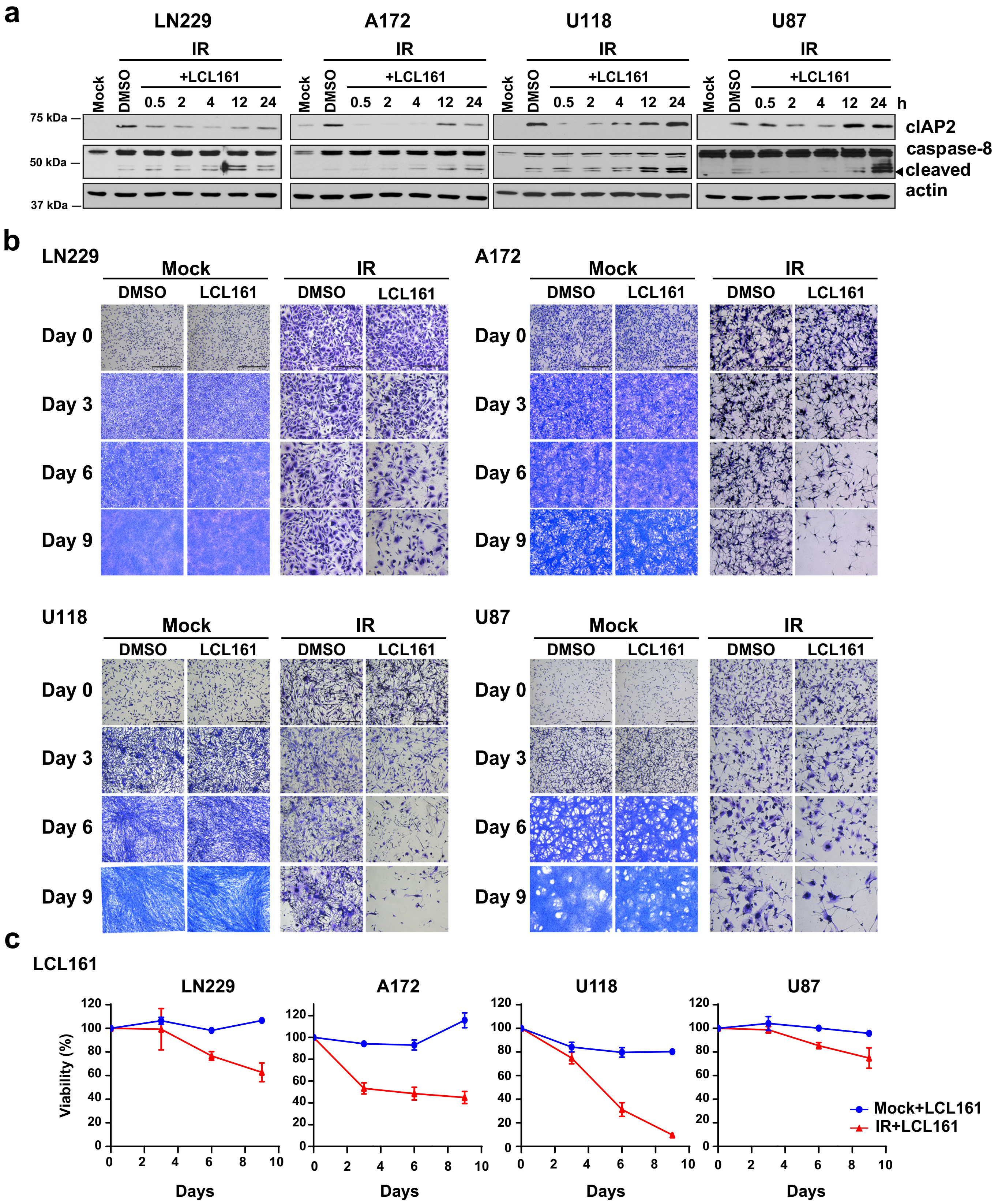
a
LN229
A172
U118
U87
IR
+LCL161
Mock
DMSO
0.5 2 4 12 24 h
75 kDa
50 kDa
37 kDa
cIAP2
caspase-8
cleaved
actin
b
LN229
A172
U118
U87
Mock
IR
DMSO
LCL161
Day 0
Day 3
Day 6
Day 9
c
LCL161
LN229
A172
U118
U87
Viability (%)
Days
Mock+LCL161
IR+LCL161

Figure EV4. The SMAC mimetic LCL161 selectively eliminates senescent GBM cells.

(**A**) GBM cells were irradiated with 10 Gy of X-rays (IR) and treated with the cIAP2 inhibitor LCL161(or DMSO as control) after 10 days for the indicated times, and cIAP2 levels and cleavage of caspase-8 were assessed by western blotting. Actin serves as loading control. (**B**) Mock-irradiated or irradiated GBM cells were treated with LCL161or DMSO as control, and the surviving cells were visualized by staining with crystal violet at the indicated times (scale bar, 500 μm), and (**C**) viability (normalized to that of DMSO-treated cells) was quantified by the MTT assay ($n = 6$–9 replicates per cell line). The drug was replaced every 72 h. Plots show mean viability $+/-$ SD. Source data are available online for this figure.

 © The Author(s)

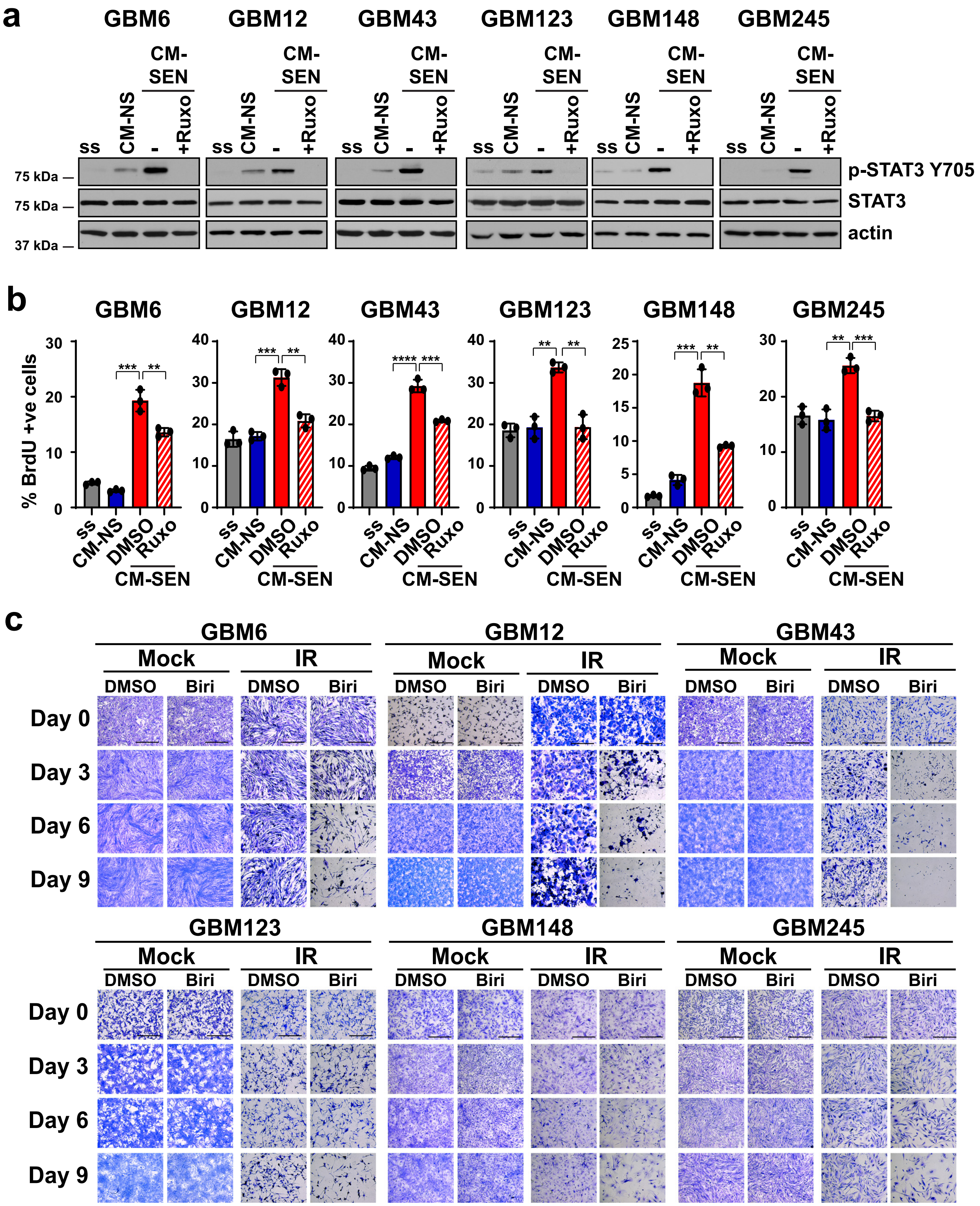
a
GBM6
GBM12
GBM43
GBM123
GBM148
GBM245
CM-SEN
ss
CM-NS
-
+Ruxo
75 kDa
75 kDa
37 kDa
p-STAT3 Y705
STAT3
actin
b
% BrdU +ve cells
ss
CM-NS
DMSO
Ruxo
CM-SEN
c
Mock
IR
DMSO
Biri
Day 0
Day 3
Day 6
Day 9

Figure EV5. Paracrine effects of senescent GBM PDX cultures and sensitivity to birinapant.

(**A**) Naive GBM PDX cultures were exposed to conditioned media from senescent (CM-SEN) or non-senescent (CM-NS) PDX cultures for 30 min, and activation of the JAK-STAT3 pathway assessed by western blotting with anti-phopho-STAT3 (Y705) antibody. Actin serves as loading control. Recipient cells were serum starved (ss) before addition of CM and were untreated or treated with the JAK inhibitor ruxolitinib, as indicated. (**B**) Serum starved (ss) PDX cells were pulsed with BrdU after exposure to CM-NS or CM-SEN in the presence or absence of ruxolitinib ($n = 3$ with at least 100 nuclei scored for each replicate), and immunofluorescence stained with anti-BrdU antibody. Nuclei are stained with DAPI (blue). Plots show mean percentages of BrdU-positive cells +/− SD. A two-tailed Student's *t* test was performed; GBM6 $P = 0.00014$, 0.00919; GBM12 $P = 0.00040$, 0.00214; GBM43 $P = 0.00004$, 0.00069; GBM123 $P = 0.00101$, 0.00148; GBM148 $P = 0.00031$, 0.00130; GBM245 $P = 0.00191$, 0.00072, respectively. (**C**) Mock-irradiated or irradiated PDX cultures were treated with birinapant or DMSO as control. The drug was replaced every 72 h, and the surviving cells were visualized by staining with crystal violet at the indicated times. Scale bar, 500 µm. Source data are available online for this figure.

 © The Author(s)
