# Supplementary material for: Targeting cIAP2 in a novel senolytic strategy prevents glioblastoma recurrence after radiotherapy
Source: EMBO Mol Med. 2025 Feb 19;17(4):645–78. doi: 10.1038/s44321-025-00201-x (PMC11982261; doi:10.1038/s44321-025-00201-x)
Supplement: Supplementary file 8 — Source data Fig. 6 [file Fig6a WB G12 Biri scan.pdf › Fig6/Fig6a WB G12 Biri scan.pdf]

Figure 6

a

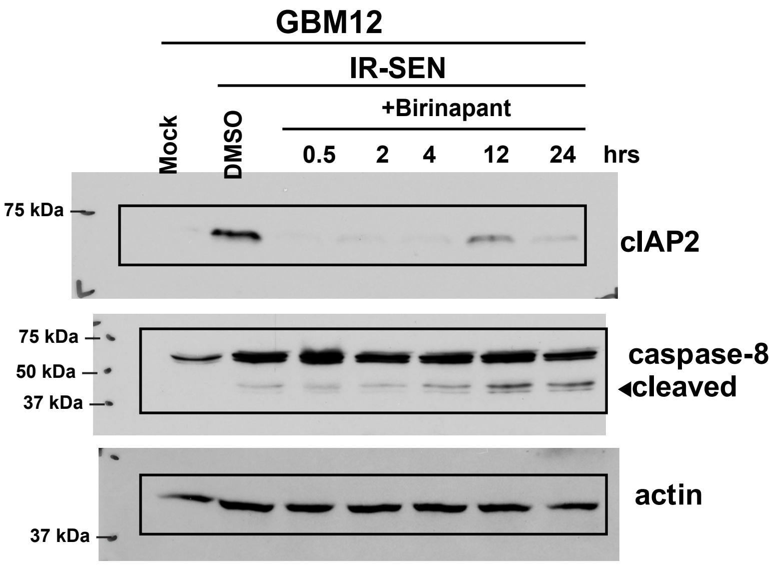

GBM12
IR-SEN
+Birinapant
Mock
DMSO
0.5
2
4
12
24
hrs
75 kDa
cIAP2
75 kDa
50 kDa
37 kDa
caspase-8
cleaved
actin
37 kDa
